# Pyridylic anions are soft nucleophiles in the palladium-catalyzed C(sp^3^)–H allylation of 4-alkylpyridines†

**DOI:** 10.1039/d0sc03304a

**Published:** 2020-12-10

**Authors:** Nour Wasfy, Faizan Rasheed, Raphaël Robidas, Isabelle Hunter, Jiaqi Shi, Brian Doan, Claude Y. Legault, Dan Fishlock, Arturo Orellana

**Affiliations:** Department of Chemistry, York University 4700 Keele Street Toronto ON Canada M3J 1P3 aorellan@yorku.ca; Department of Chemistry, Centre in Green Chemistry and Catalysis, University of Sherbrooke 2500 Boulevard de l’Université Sherbrooke Québec J1K 2R1 Canada claude.legault@usherbrooke.ca; Process Chemistry and Catalysis, Synthetic Molecule Technical Development, F. Hoffmann-La Roche Ltd 4070 Basel Switzerland

## Abstract

We report a mild palladium-catalyzed method for the selective allylation of 4-alkylpyridines in which highly basic pyridylic anions behave as soft nucleophiles. This method exploits alkylidene dihydropyridines, which are semi-stable intermediates readily formed using a ‘soft-enolization’ approach, in a new mechanistic manifold for decarboxylative allylation. Notably, the catalytic generation of pyridylic anions results in a substantially broader functional group tolerance compared to other pyridine allylation methods. Experimental and theoretical mechanistic studies strongly suggest that pyridylic anions are indeed the active nucleophiles in these reactions, and that they participate in an outer-sphere reductive elimination step. This finding establishes a new p*K*_a_ boundary of 35 for soft nucleophiles in transition metal-catalyzed allylations.

## Abstract 2

We report a mild palladium-catalyzed method for the selective allylation of 4-alkylpyridines in which highly basic pyridylic anions behave as soft nucleophiles.

## Graphical abstract

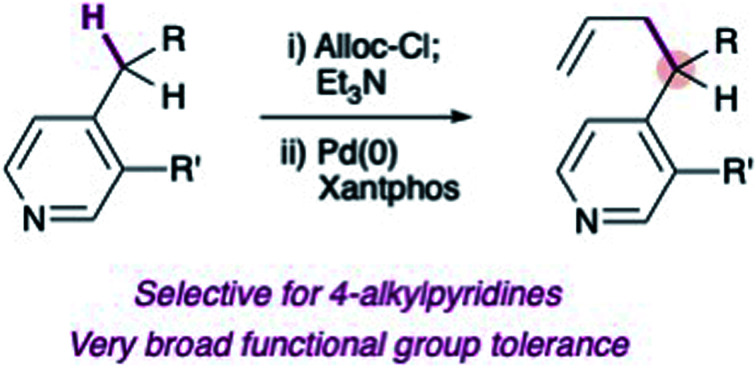

## Introduction and background

**Fig. 1:**
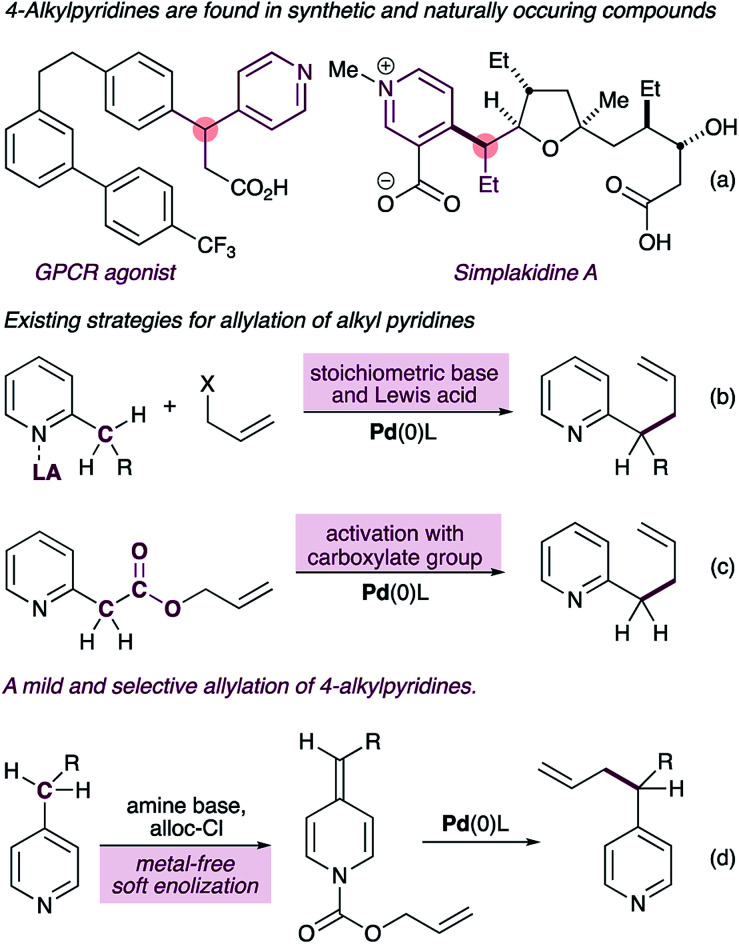
4-Alkylpyridines with stereogenic pyridylic carbons are featured in synthetic and naturally occurring biologically active molecules (a). Current approaches to palladium-catalyzed C(sp^3^)–H allylation of alkylpyridines involve pre-activation with a Lewis acid (b) or a require a pre-existing carboxylate group (c). Our new approach exploits alkylidene dihydropyridines for selective C(sp^3^)–H allylation of 4-alkylpyridines (d).

Pyridines and related aza-arenes bearing a stereogenic carbon at the heterobenzylic position are widely represented in drug candidates,^1,2^ bioactive natural products^3^ and agrochemicals (Fig. 1a). In pharmaceuticals, the stereogenic carbon in these compounds is often responsible for their increased target selectivity,^4^ and therefore transformations that install sterogenicity at the pyridylic position^5,6^ are highly valuable.^7,8^ Due to the high p*K*_a_ value of alkyl pyridines, however, methods to functionalize the pyridylic C(sp^3^)–H require some form of pre-activation of the pyridine ring to enable functionalization. One strategy involves coordination of the pyridine nitrogen to a Lewis acid prior to deprotonation with a stoichiometric base (Fig. 1b).^9–13^ Most of the reported methods using this strategy require superstoichiometric amounts of strong bases (*e.g. n*-BuLi and/or MHMDS), severely limiting the functional group content, with Sawamura's method^11^ being a notable exception. Furthermore, these reactions are limited to 2-alkylpyridines. A second strategy requires a pre-existing carboxylate group to further activate the pyridylic C(sp^3^)–H bond, thus narrowing the substrate scope (Fig. 1c).^14^

Here we report a new mechanistic framework for C(sp^3^)–H allylation of pyridines, which exploits their metal-free soft-enolization^15^ to form alkylidene dihydropyridines^16^ (ADHPs) as semi-stable intermediates that are primed for a palladium-catalyzed decarboxylative allylation reaction (Fig. 1d). This approach imparts complete selectivity for 4-alkylpyridines and stands in contrast to all other methods which are either selective for 2-alkylpyridines or display no selectivity. Importantly, the use of a very mild base for the synthesis of the ADHPs and the catalytic generation of pyridylic anions in the allylation step allows the presence of a very broad range of functionality in the substrates, including enolizable and electrophilic carbonyls (*e.g.* free aldehydes) and acidic N–H groups (*e.g.* carbamates).

## Reaction design and development

**Scheme 1:**
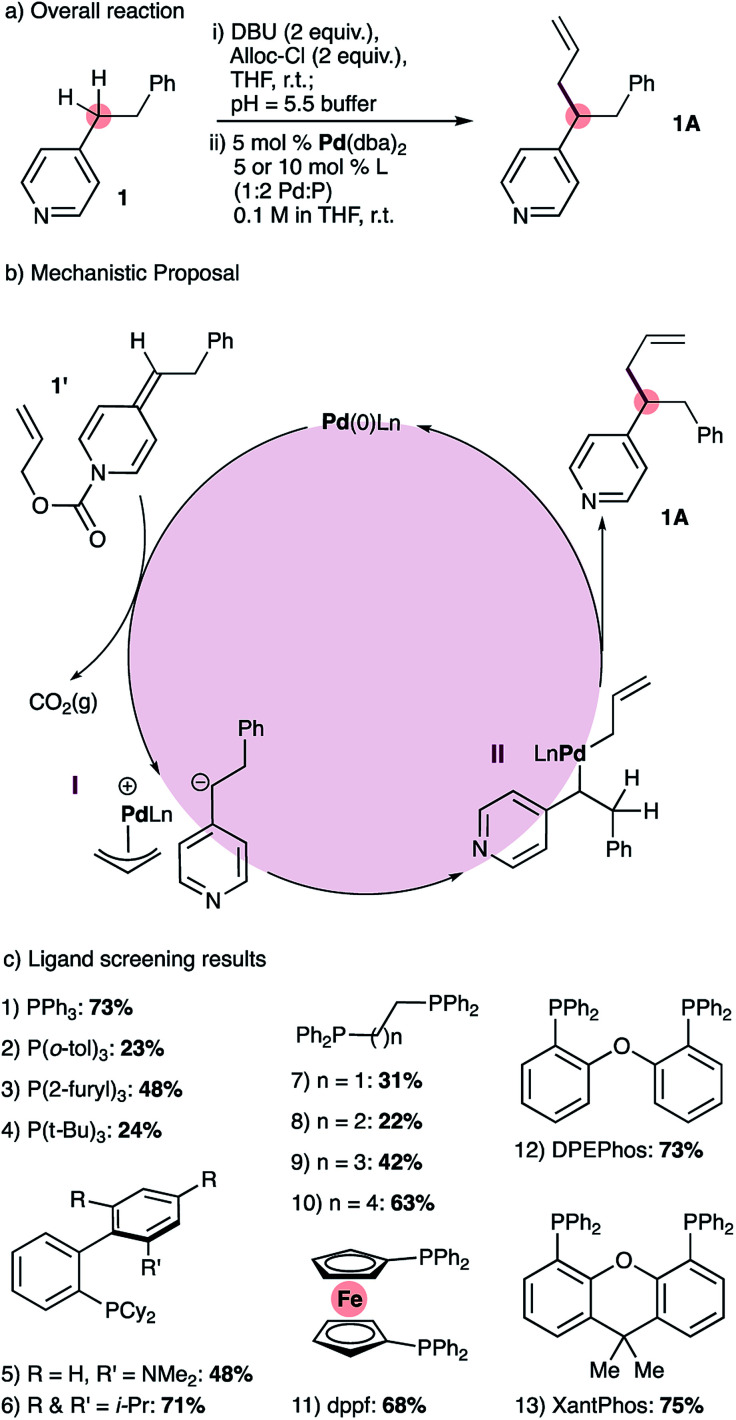
Reaction development: overall reaction (a), mechanistic proposal (b) and ligand screening results (c).

At the outset of this work we envisioned the mechanistic framework outlined in Scheme 1(b). Thus, treatment of ADHPs such as 1′, readily prepared from the corresponding pyridine 1 (Scheme 1(a), see ESI† for full details), with a Pd(0) catalyst would result in ionization of the allyl group, releasing CO_2_(g), a pyridylic anion and a cationic allyl-Pd(ii) intermediate (I). Since 4-alkylpyridines have p*K*_a_ values^17^ above the currently established limit for ‘soft’ nucleophiles in palladium-catalyzed allylation reactions,^12*a*^ it seemed likely that the pyridylic anion would behave as a ‘hard’ nucleophile and form alkyl palladium intermediate II.^18^ Reductive elimination would give the expected allylation product 1A.

Catalyst screening reactions were conducted using Pd(dba)_2_ as the Pd(0) source and a 1 : 2 ratio of palladium to phosphine (Scheme 1(c)). Use of triphenylphosphine provided the desired allylation product 1A in 73% yield, while tri(*o*-tolyl)phosphine provided a lower yield (entries 1 & 2). More electron-rich monodentate ligands gave moderate or low yields of the allylated product (entries 3 & 4). The use of monodentate biaryl ligands (Buchwald ligands) gave the allylated product, but did not prove superior to triphenylphosphine (entries 5 and 6). Tethered alkyl diphenylphosphines with small bite angles (entries 7 & 8) gave low yields of 1A, and the yield increases with wider bite angles (entries 9 & 10). Bidentate ligands with large bite angles (entries 11 to 13) provided consistently good results but none was significantly superior to triphenylphosphine. Nonetheless, we chose XantPhos for future reaction development reasoning that catalysts bearing bidentate ligands should display greater stability than those with monodentate ligands.

**Scheme 2:**
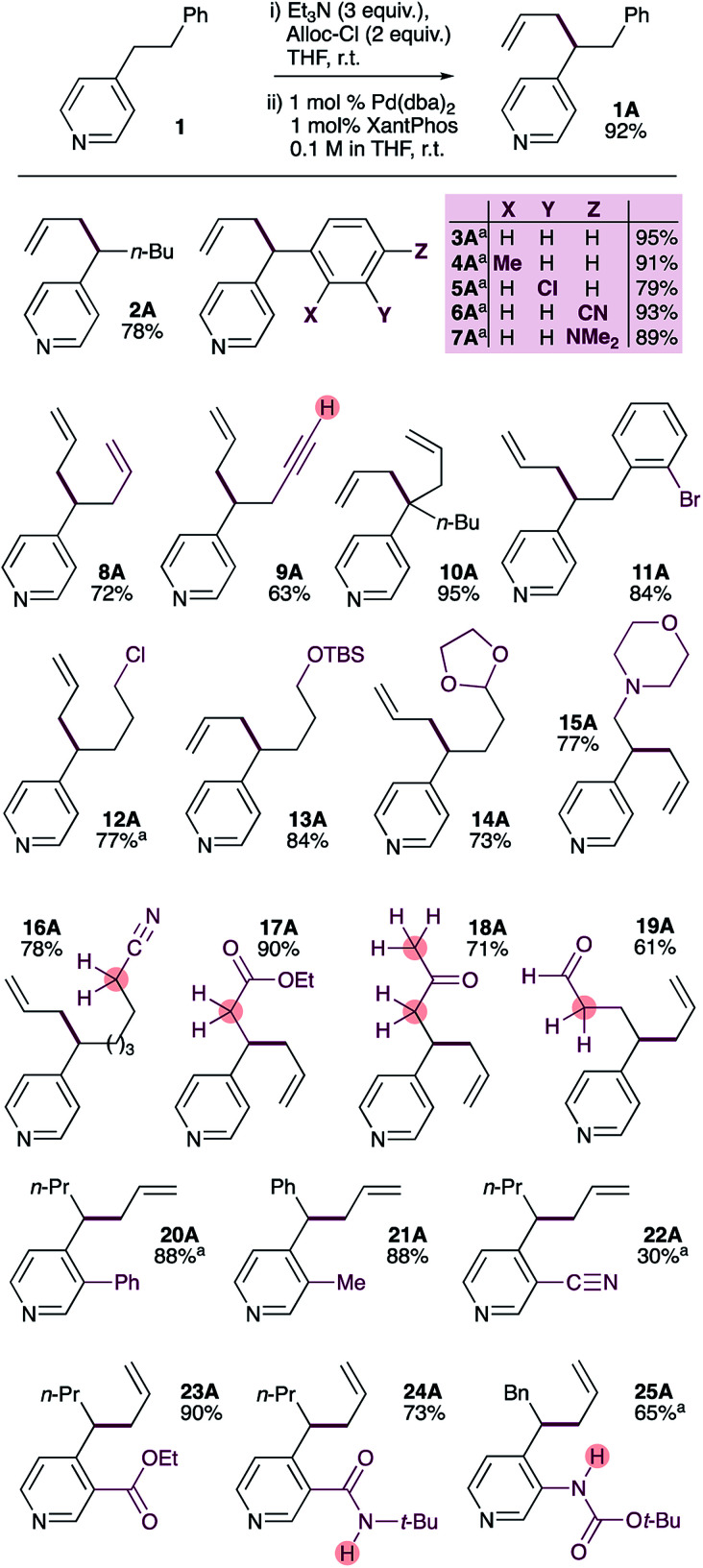
Substrate scope of the selective palladium-catalyzed allylation of 4-alkylpyridines. ^a^ Pd_2_dba_3_·CHCl_3_ used instead of Pd(dba)_2_.

We also noted that during our ligand-screening study, reaction yields reached a plateau of 70–75% with ligands of varying structure, including triphenylphosphine, XPhos, as well as bidentate phosphines with large bite angles such as DPEPhos and XantPhos (Scheme 1c, entries 1, 6, 12 and 13 respectively). Therefore, we suspected that yield losses were a result of the mildly acidic work-up step required to remove DBU, which was used during the formation of ADHP 1′ (Scheme 1a). To mitigate this problem, we developed an alternative high-yielding method for the synthesis of ADHPs that uses triethylamine and avoids aqueous work-up (Scheme 2, see ESI† for full details). With this modification, and using 1% catalyst loading, 4-phenethylpyridine could be allylated in 92% isolated yield, and therefore these conditions were adopted for substrate scope studies.

### Substrate scope

We were pleased to find that the optimized conditions are suitable for a wide range of 4-substituted pyridines (Scheme 2). 4-*n*-Pentylpyridine provided the allylation product 2A in 78% yield. Benzylpyridines bearing methyl-, chloro-, cyano- and dimethylamino-substituents on the benzene ring could also be allylated in uniformly good yields (3A–7A). It should be noted that 4-benzylpyridines have significantly lower p*K*_a_ values than 4-alkylpyridines (26.7 and 35 respectively)^17^ and very likely act as soft nucleophiles in these reactions,^18^ in contrast to the proposed mechanism (Scheme 1) for 4-alkylpyridines. Substrates bearing alkenes or terminal alkynes were also tolerated, giving the corresponding products in good yields (8A and 9A). It is also possible to generate products with a quaternary pyridylic carbon in excellent yield (10A). Interestingly, a substrate bearing an aryl bromide gives allylation product 11A in good yield, with no evidence of oxidative addition across the C(sp^2^)–Br bond. Similarly, a primary alkyl chloride is well tolerated (12A). In contrast to other pyridine allylation methods that use Lewis acids (*e.g.* BF_3_·OEt_2_), our method tolerates acid-sensitive TBS-protected primary alcohols (13A) and acetal-protected aldehydes (14A). Amines do not interfere with the reaction, giving the allylated product 15A in good yield. Due to the relatively low acidity of alkylpyridines (p*K*_a_ = 35) it is common to use strong bases (*e.g. n*-Buli or MHMDS, M = Li, Na or K) alone or in combination with Lewis acid to generate pyridylic nucleophiles,^9,10,12^ which limits functional group tolerance. In contrast, our method avoids the use of aggressive reagents and tolerates the presence of functional groups with activated C–H or N–H groups with p*K*_a_ values well below 35. Indeed, pyridines bearing electrophilic functional groups with acidic α-protons, including nitriles (16, p*K*_a_ ∼ 32), esters (17, p*K*_a_ ∼ 30), ketones (18, p*K*_a_ ∼ 24), and even aldehydes (19), are allylated in good-to-excellent yields.

It is also possible to allylate 4-alkylpyridines bearing substituents at the 3-position. 3-Phenyl-4-*n*-butyl pyridine (20) and 3-methyl-4-benzyl pyridine (21) provide allylated products in excellent yield. Substrates bearing electrophilic and strongly coordinating functional groups at the 3-position are also tolerated, and generally provide the allylated products in good yields (22A–25A). The low yield obtained with 3-cyano-4-butylpyridine (22) reflects its low nucleophilicity, which results in incomplete conversion to the corresponding ADHP. It is notable that the acidic N–H protons in the amide (24, p*K*_a_ ∼ 26) and carbamate (25, p*K*_a_ ∼ 21) groups do not interfere with the reaction.

### Pyridylic selectivity

**Fig. 2:**
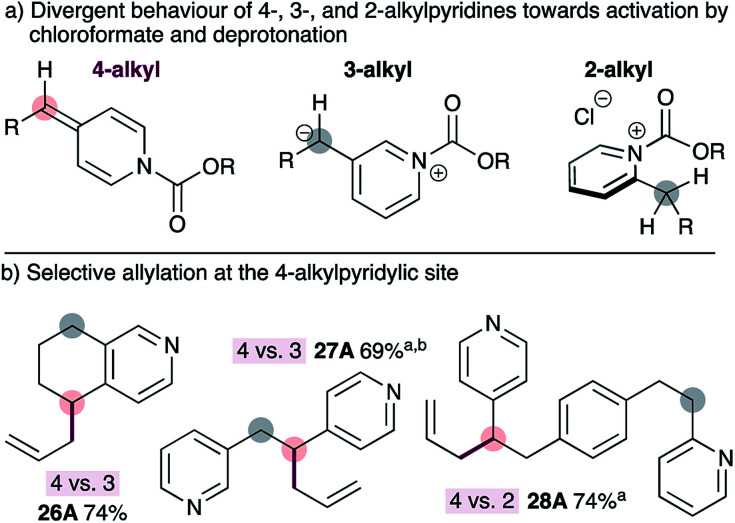
Divergent reactivity of alkylpyridines towards chloroformates enables selective allylation at the 4-pyridylic position in substrates with multiple pyridylic sites. ^a^Pd_2_dba_3_·CHCl_3_ used instead of Pd(dba)_2_. ^b^Dioxane used instead of THF.

To our knowledge, all existing methods for transition metal-catalyzed pyridylic allylation are selective for the 2-pyridylic position in substrates with multiple pyridylic sites,^9–11^ or do not display positional selectivity at all,^12^ unless guided by pre-existing functionality.^13,14^ Therefore, selective allylation of 4-alkylpyridines in substrates bearing multiple pyridylic positions would be a valuable synthetic advance. Our strategy for achieving selective allylation exploits the divergent behaviour of 2-, 3- and 4-alkylpyridines towards activation with chloroformate and subsequent deprotonation with mild base (Fig. 2a). 4-Alkylpyridines are readily activated by chloroformate reagents and the resulting salts are easily deprotonated with mild base to generate ADHPs. 3-Alkylpyridines also form the corresponding pyridinium salts, however deprotonation is not possible with mild base because the resulting 3-pyridylic anion would not be resonance-stabilized by the pyridine nitrogen. The heterobenzylic position in 2-alkylpyridines is inherently activated by the ring, and these substrates also form the required pyridinium salts readily,^19^ however they cannot be deprotonated with mild base. The reasons for this are unclear at present but may reflect an orthogonal relationship between the heterocycle and the alkoxycarbonyl group in the pyridinium salt or developing allylic strain during the necessary deprotonation step. Regardless of the reasons, 4-alkylpyridines can be selectively activated over other positional isomers.

As expected, using standard conditions for the synthesis of ADHPs and subsequent palladium-catalyzed allylation, tetrahydroisoquinoline 26 can be allylated selectively at the 4-position in good isolated yield (Fig. 2b). Similarly, tethered 3- and 4-alkylpyridines (*i.e.*27) can be allylated exclusively at the 4-pyridylic position. Selective allylation at the 4-alkylpyridylic site is also possible in substrates bearing tethered 2- and 4-alkylpyridines such as 28. This is notable since all other transition metal-catalyzed reactions would result in allylation at the 2-alkylpyridylic site or display little or no selectivity. In this case, no products arising from allylation at the 2- or 3-position of the pyridines were observed. Interestingly, however, double allylation of the 4-pyridylic position of substrate 27 was observed when the reaction was conducted in THF but only the monoallylated product 27 was observed when dioxane is used as the solvent.

**Fig. 3:**
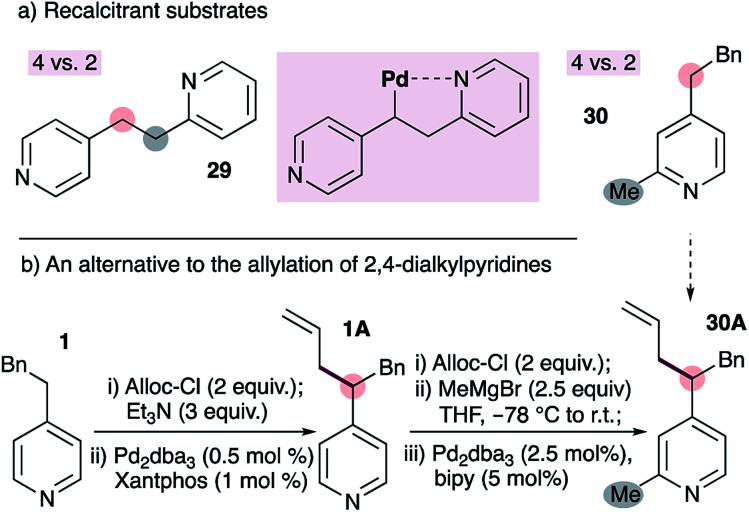
Some limitations of our palladium-catalyzed allylation of 4-alkylpyridines (a), and an alternative to selective allylation of 2,4-dialkylpyridines (b).

We have encountered some limitations to the current method (Fig. 3). The reaction fails with substrate 29, which bears tethered 2- and 4-alkylpyridines (Fig. 3a). We speculated that this may be due to coordination of the 2-alkylpyridine to the metal as shown (inset), however the reasons remain unclear (see below). A second limitation is the inability to allylate pyridines bearing 2- and 4-alkyl groups on the same ring (*e.g.*30 to 30A, Fig. 3). This stems from the inability to form the requisite ADHP, as described in Fig. 2a. This limitation can be circumvented by first installing the allyl group on a 4-substituted pyridine, followed by the addition of a Grignard reagent to the chloroformate-activated pyridine^20^ (Fig. 3b). Notably, the use of allyl chloroformate as the activating group conveniently allows removal with a palladium-bipyridine catalyst, and achieves aromatization of the dihydropyridine to the pyridine with allylpalladium(ii) as the oxidant.^21^

### Mechanistic studies

A number of methods for allylation of alkylpyridines rely on the stoichiometric generation of nucleophilic pyridylic anions through the use of strong base (Fig. 1b), and this limits the functional group content in the substrates. In contrast, our reaction tolerates a wide variety of electrophilic and acidic functional groups (Scheme 2). Our originally proposed mechanism (Scheme 1) invokes pyridylic anions, which would normally react with some of the functional groups tolerated in the substrate scope (*i.e.*9A, 12A, 16–19A and 22–25A). This seeming discrepancy prompted us to gain a better understanding of the reaction by using mechanistic probes.

Indirect evidence for the generation of pyridylic anions was obtained by the allylation of phenethylpyridine using bidentate ligands bearing the 1,2-*trans*-diamine backbone (eqn (1)). When the parent ligand is used (*i.e.* R = H) the reaction fails. In contrast, the use of the *N*-methylated version of the ligand provided the allylated product in 88% isolated yield. These experiments suggest that catalyst deactivation in the first instance may be the result of deprotonation of the amide backbone on the ligand by a basic pyridylic anion. It is worth noting that although some substrates (*i.e.*24 and 25) contain acidic N–H groups that could be deprotonated by pyridylic anions, they provide the allylated product in good yields. Although this undesired substrate deprotonation may lead to reduced yields, it does not affect the nature of the catalyst in any way, allowing the reaction to proceed. Furthermore, the allylated product will predominate if the rate of reductive elimination is faster than the rate of deprotonation.1
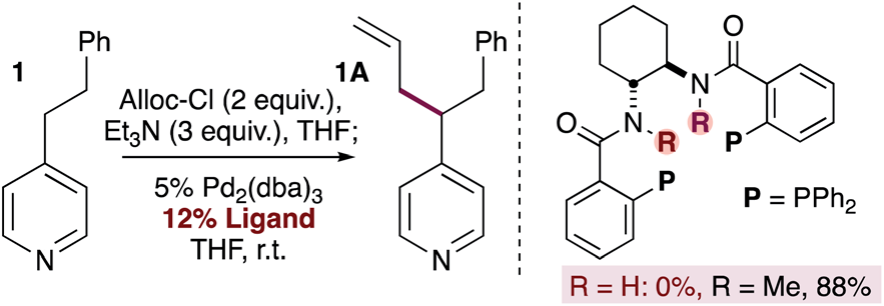


**Scheme 3:**
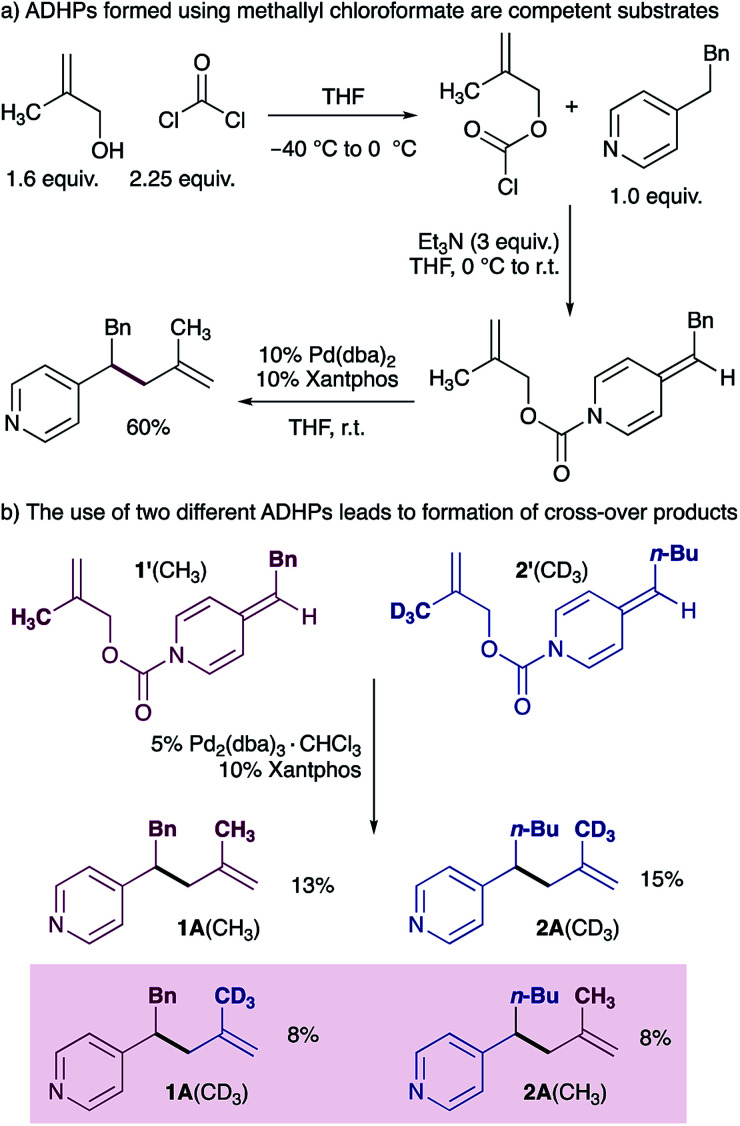
Establishing the presence of ion pairs.

Our mechanistic proposal also invoked the formation of an ion pair (I in Scheme 1b), and we therefore aimed to use a cross-over experiment to establish the participation of solvent separated ion pairs. We chose to use methallyl chloroformate^22^ rather than allyl chloroformate for this experiment due to ease of synthesis of the deuterated methallyl chloroformate (see ESI† for details), and because the reaction of ADHPs prepared from methallyl chloroformate works in good yield under standard conditions (Scheme 3a). A cross-over experiment using ADHP 1′ and ADHP 2’ revealed that all four possible cross-over products are observed, supporting the participation of ion pairs. It is interesting to note that the product distribution suggests that ion separation is slow relative to reductive elimination.

**Scheme 4:**
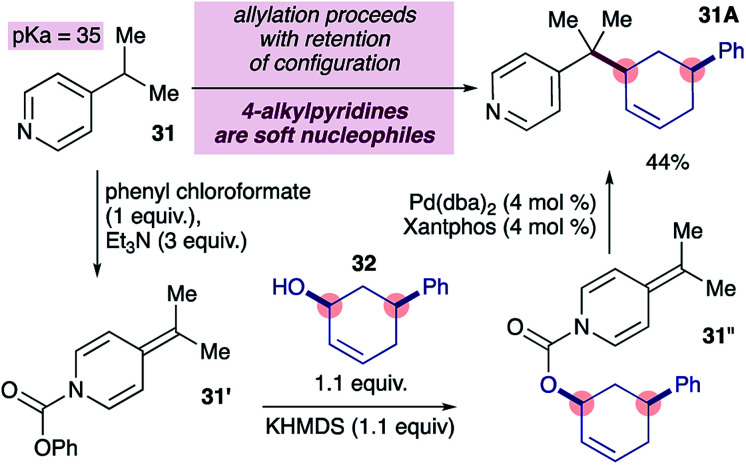
Alkylpyridines undergo outer-sphere reductive elimination with allyl electrophiles in palladium-catalyzed allylation reactions.

We became interested in the mode of reductive elimination in these reactions because this would inform the future development of an enantioselective variant. Following the dogma of palladium-catalyzed allylations^18^ and assuming fast decarboxylation, alkylpyridines are expected to behave as ‘hard’ nucleophiles, undergoing inner-sphere reductive elimination, owing to their high p*K*_a_ values, as suggested in Scheme 1b. Recently however, it has been shown that nucleophiles with p*K*_a_ values of up to 32 can also behave as soft nucleophiles,^12*a*^ at least under some conditions. The p*K*_a_ range for 4-substituted pyridines spans from 26 to 35 (ref. 23) and covers the current limit of 32 for soft nucleophiles, and therefore it is of fundamental interest to determine the behaviour of 4-alkylpyridines (p*K*_a_ = 35 in DMSO) in transition metal-catalyzed allylations. Ideally, the stereochemical probe required (*i.e.* ADHP 31′′ in Scheme 4) would be prepared from the corresponding chloroformate, however we were unable to prepare the required chloroformate cleanly (not shown) using a variety of conditions.^24*a*^ We therefore prepared the required probe by forming the ADHP 31′ using phenylchloroformate, and substituting phenoxide with the potassium alkoxide ion of alcohol 32, as previously reported.^24*b*,*c*^ Subjecting stereochemical probe 31′ to standard conditions provided coupled product 31A in 44% yield.^25^ Notably, the cross-coupling product formed displayed complete retention of configuration (*i.e.* double inversion), consistent with outer-sphere reductive elimination.^26^ This is a remarkable finding because it would establish a new p*K*_a_ boundary for soft nucleophiles in transition metal-catalyzed allylations if pyridylic anions are indeed involved, as suggested by prior experiments (*i.e.*eqn (1)).

The unexpected outcome of the stereochemical probe experiment, together with the broad functional group tolerance of this reaction, raises the possibility that ADHPs themselves could be sufficiently nucleophilic towards allylpalladium intermediates. To test this hypothesis we prepared the known, cationic XantPhos-stabilized allylpalladium(ii) complex^27^ and subjected it to a stoichiometric reaction with ADHP 1′′ prepared using ethyl chloroformate and therefore unable to react with palladium (eqn (2)). Interestingly, this reaction provided the allylated product in 82% yield after hydrolytic work-up. It is worth noting however, that the allylpalladium complex proved insoluble in THF (which is the standard solvent) and therefore the reaction was conducted in DCM. Furthermore, the rate of this reaction is significantly slower than that of the parent reaction despite the fact that it is stoichiometric in palladium.2
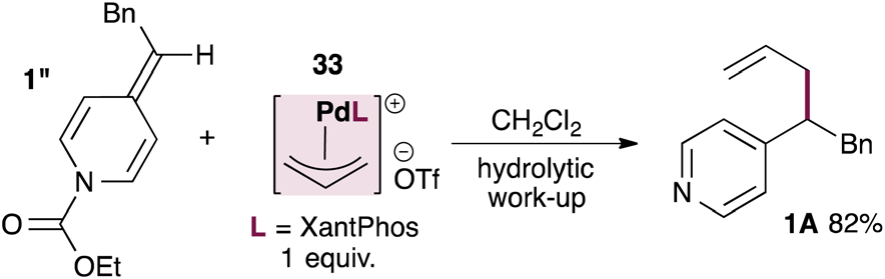


**Scheme 5:**
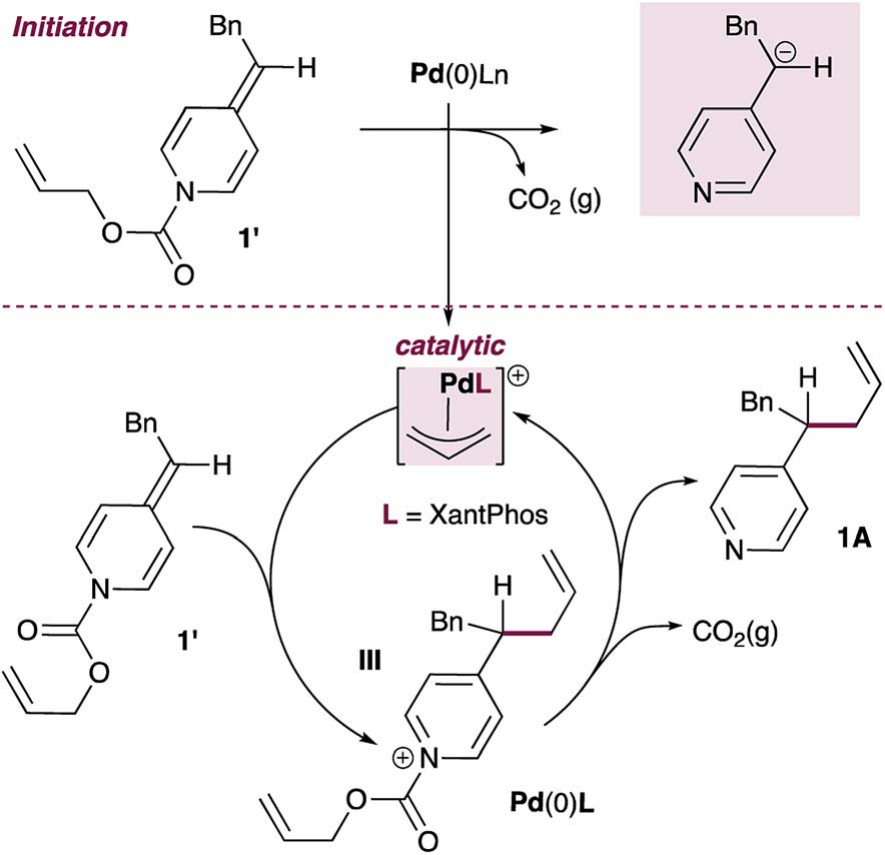
An alternative mechanistic proposal with ADHPs as active nucleophiles in palladium-catalyzed pyridylic allylation.

Given this finding, a different mechanism that accounts for the broad functional group tolerance observed can be proposed (Scheme 5). Under standard conditions, the Pd(0)L catalyst would undergo oxidative addition to ADHP 1′ to generate a cationic allylpalladium(ii) intermediate and a pyridylic anion (inset). The allylpalladium complex could then be attacked by a second ADHP to generate a pyridinium intermediate (III) and a Pd(0)L catalyst. These two partners would combine to regenerate the cationic allylpalladium(ii) intermediate and release allylated product 1A.

The viability of this proposal is predicated on (i) the cationic allylpalladium complex acting as a competent catalyst in the allylation reaction, and (ii) ADHPs outcompeting pyridylic anions in the nucleophilic addition to the allylpalladium(ii) intermediate. A liability in this proposal is that the fate of the pyridylic anion remains unclear.

As shown in eqn (3), the XantPhos-stabilized allylpalladium(ii) complex is indeed catalytically active in these reactions, providing the allylated product in 92% isolated yield, lending some support to the mechanism in Scheme 5.3
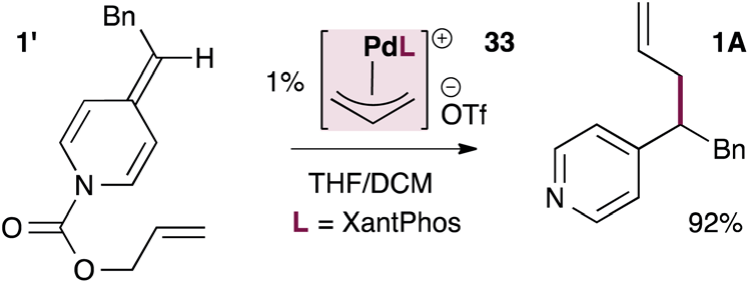


It seemed unlikely however, that a neutral ADHP would be more nucleophilic than a pyridylic anion, casting doubt on the proposal in Scheme 5. Previous experiments have shown that ADHPs prepared from allyl chloroformate generate solvent separated ion pairs and give cross-over products under standard conditions (see Scheme 3). Therefore, it is possible to test the ability of an ADHP to outcompete pyridylic anions by subjecting ADHP 11′ to standard reaction conditions and in the presence of an equal amount of ADHP 1′′, which is prepared using *ethyl* chloroformate and therefore unable to generate allylpalladium(ii) intermediates itself (eqn (4)). Not surprisingly, this reaction only yields the expected allylation product 11A, along with the unreacted ADHP 1′, and with no evidence of allylated pyridine 1A. This strongly suggests that ADHPs are not competent nucleophiles in the presence of pyridylic anions.4
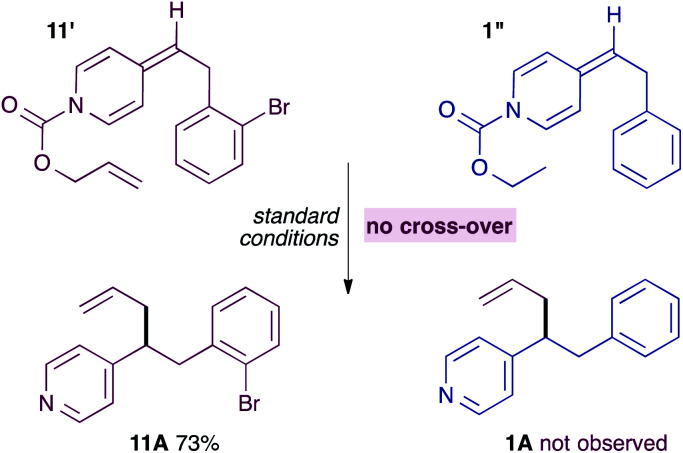


**Fig. 4:**
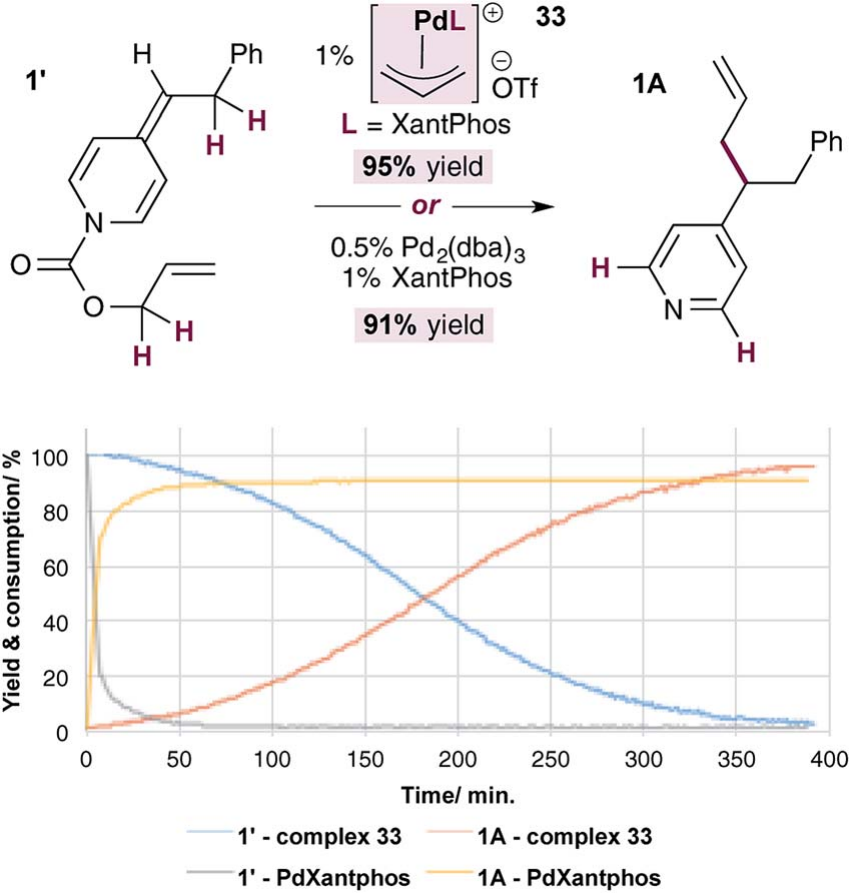
Comparison of the rate of pyridylic allylation using standard conditions (Pd(0) and XantPhos) and cationic complex 33.

Finally, we conducted a head-to-head comparison of the allylation reaction of ADHP 1′, using the standard catalytic system (*i.e.* 1% Pd(0) and XantPhos) and the allylpalladium(ii) complex 33 (Fig. 4). The relative rates of these reactions were compared by monitoring the disappearance of the substrate and appearance of the product using NMR signal of the indicated protons. In both cases the reactions are high yielding, however it is clear that the reaction under standard conditions, likely proceeding through generation of pyridylic anions as nucleophiles, occurs at a significantly faster rate than that using complex 33, in which neutral ADHPs act as nucleophiles. This indicates that the rate of nucleophilic attack on an allylpalladium complex by a pyridylic anion is significantly faster than the rate of attack by a neutral ADHP,^28^ making the catalytic cycle proposed in Scheme 5 highly unlikely.

### Computational study

**Scheme 6:**
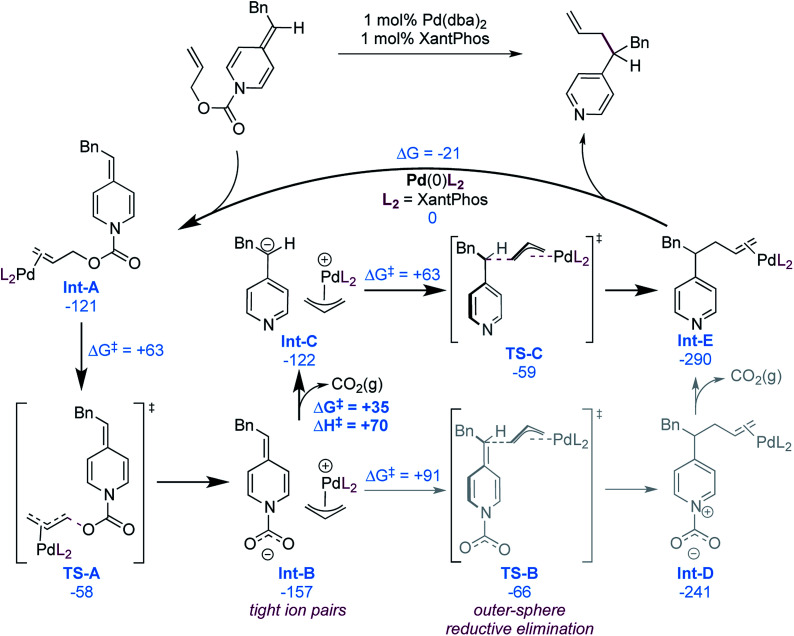
Computationally supported catalytic cycle (free energies in kJ mol^−1^), most probable pathway illustrated in bold.

While our experimental mechanistic probes provided some insight into the mechanism and allowed us to exclude some possibilities, they did not allow us to firmly establish the nature of the nucleophile in these reactions. In view of seemingly conflicting data, specifically the wide substrate scope (particularly acidic functional groups, Scheme 2) and catalyst decomposition (suggesting a strongly basic intermediate, eqn (1)), it remained unclear if a free pyridylic anion or a carboxylate-stabilized pyridylic anion is involved. To gain a better understanding of the mechanism, we therefore performed a computational investigation using DFT (M06-D3/6-311++G(d,p) + SDD(Pd)//wB97XD/6-31+G(d,p) + SDD(Pd))^29^ using substrate 1, as it is a typical and high-yielding example. The mechanistic possibilities supported by the calculations are illustrated in Scheme 6.

**Scheme 7:**
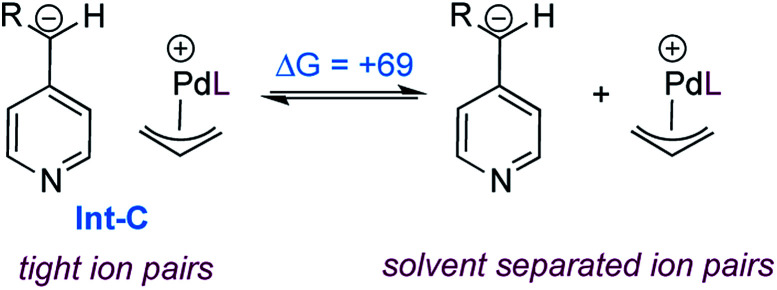
Dissociation of the ion pair Int-C (free energies in kJ mol^−1^).

As expected, formation of the catalyst–substrate complex (Int-A) is found to be exergonic. At this point, oxidative addition occurs (TS-A) and leads to the formation of the cationic allylpalladium(ii) complex and a carboxylate-stabilized pyridylic anion. The proximity of the charges is significantly stabilizing and thus the two species form the tight ion pair Int-B, the separation being endergonic (Scheme 7).

This ion pair (Int-B) can undergo outer-sphere allylation *via*TS-B with a free energy barrier of 91 kJ mol^−1^. Calculation of an inner-sphere transition state resulted in an even higher free energy barrier of 126 kJ mol^−1^ (see ESI†). It is thus not involved in the mechanism, in line with the experimental observations (Scheme 4). The allylation product Int-D can then undergo exergonic decarboxylation to form the final product.

An alternative mechanistic pathway can be envisioned, where Int-B decarboxylates prior to addition, to form the ion pair Int-C. This new intermediate was also found to undergo preferentially the allylation *via* an outer-sphere mechanism (TS-C). The calculations suggest that the decarboxylation is endergonic, with a computed free energy cost of 35 kJ mol^−1^. However, no decarboxylation transition structure could be found by performing relaxed scan of the elongation of the N–CO_2_^−^ bond length, suggesting a fast process. To estimate the upper activation barrier of decarboxylation, we computed the enthalpy of the process, which was found to be +70 kJ mol^−1^. The actual free energy barrier should then be between +35 and +70 kJ mol^−1^, which is lower than the activation barrier from Int-B to TS-B in the previously discussed mechanistic alternative (see above).

We also calculated the free energy to convert the tight ion pair Int-C to its solvent separated ion pair (Scheme 7). While endergonic, the cost of dissociation is close in energy to the allylation step (+69 *vs.* +63 kJ mol^−1^). This would enable possible kinetic competition between ion exchange and allylation, but the kinetic preference for allylation would result in the non-crossover products as the major components in the mixture. This is consistent with the product distribution observed in the cross-over experiment (Scheme 3b). Finally the product to substrate exchange on the Pd(0) catalyst was found to be exergonic, hence no product inhibition is expected to be observed.

## Discussion

The original mechanistic proposal for this study (Scheme 1b) invoked the formation of strongly basic pyridylic anions. Consistent with the widely accepted behaviour of strongly basic anions in transition metal-catalyzed allylation reactions, this proposal also invoked an inner sphere reductive elimination event. The wide functional group tolerance of this reaction seemed at odds with the presence of a strongly basic anion and prompted us to study the mechanism in some detail. Furthermore, the recent finding that substrates with p*K*_a_ values as high as 32 act as soft nucleophiles in transition metal-catalyzed allylation reactions, coupled with the fact 4-alkylpyridines have p*K*_a_ values that are not significantly higher, also raised questions about the reductive elimination step in this reaction.

The results of our mechanistic probe experiments together with a theoretical treatment support a mechanism that proceeds *via* catalyst coordination to substrate (Scheme 6) and oxidative addition, resulting in an ion pair consisting of carboxylate-stabilized pyridylic anion and a cationic allylpalladium(ii) intermediate (Int-A to Int-B*via*TS-A). Two mechanistic pathways were identified from Int-B, one involving pre-decarboxylative allylation (Int-B to Int-E*via*TS-B and Int-D), and the other proceeding *via* decarboxylation followed by allylation (Int-B to In-E*via*Int-C and TS-C). Our calculations show that both mechanistic pathways are reasonable, with TS-B being lower in energy than TS-C, and decarboxylation having a lower energy barrier than either TS-B and TS-C. Assuming reversible decarboxylation, these computations would suggest a Curtin–Hammett regime, with the reaction proceeding *via*TS-B. Alternatively, if decarboxylation is irreversible, then the reaction must proceed through TS-C. Thus, the preferred mechanism hinges the reversibility of decarboxylation.

There remains some debate regarding the reversibility of decarboxylation processes. For example, it has been suggested that slow separation of the carbanion and CO_2_(g) is the key factor responsible for reversibility.^30^ However, using calculations and experimental kinetic isotope effects it has been shown that there is no significant reversibility in simple decarboxylations.^31^ Recently it was also shown that decarboxylation of the potassium salt of 4-pyridylic acetic acid in DMF solution, generating a 4-pyridylic anion, is reversible under a CO_2_(g) atmosphere.^32^ Further, exchange of CO_2_(g) and ^13^CO_2_(g) directly demonstrates that CO_2_(g) diffuses away from the anion, countering the notion that separation of a carbanion and CO_2_(g) is limiting.

In view of the relatively low calculated barrier to decarboxylation (Int-B to Int-C), insignificant reversibility of decarboxylation of pyridylacetic acids^31^ as well as evidence for diffusion of CO_2_(g) from pyridylic anions,^32^ and the suggested presence of strongly basic anions in our reaction (eqn (1)) we favour the mechanistic pathway in which decarboxylation occurs prior to allylation (shown in bold in Scheme 6). If correct, this mechanism is notable because it would implicate highly basic pyridylic anions as reactive intermediates in the presence of other reactive functional groups. Palladium-catalyzed cross-coupling reactions of highly basic and nucleophilic reagents in the presence of electrophilic functional groups are known,^33^ and can be synthetically useful if the rate of transmetallation to palladium is faster than the rate of addition to the electrophilic site.^34^ In our case, the concentration of the pyridylic anion is low throughout the reaction because it is catalytically generated, the decarboxylation is predicted to be endergonic, and ion separation is disfavoured (Scheme 7). Thus, the observed functional group tolerance is likely a reflection of a fast rate of allyation relative to the reaction of the pyridylic anion with an electrophilic or acidic function. It is possible, however, that the reaction proceeds through allylation prior to decarboxylation (Int-B to Int-D*via*TS-B). This mechanistic alternative could be supported by observing Int-D, which we will investigate in future studies. Irrespective of the precise mechanism, our work shows that anions derived from 4-alkylpyridines (p*K*_a_ = 35 in DMSO) behave as soft nucleophiles towards allylpalladium intermediates. This study therefore suggests a new p*K*_a_ limit for soft nucleophiles in transition metal-catalyzed allylation reactions.

## Conclusions

We have developed a practical, mild and selective palladium-catalyzed method for the allylation of 4-alkylpyridines. This method exploits the conversion of 4-alkylpyridines to alkylidene dihydropyridines (ADHPs) under metal-free soft enolization conditions using inexpensive and broadly available reagents. The strategic use of ADHPs as semi-stable substrates provides a new entry into transition metal-catalyzed allylation reactions of pyridines, which complements all known methods in terms of positional selectivity. This reaction tolerates a very broad range of functional groups in the substrates, including electrophilic groups and acidic protons, which is notable given the low acidity of alkylpyridines. In addition, the reaction allows allylation at the 4-alkyl pyridylic position in substrates bearing multiple pyridylic sites. Experimental and computational mechanistic studies revealed that catalytically generated pyridylic anions are the likely nucleophiles in these reactions and that reductive elimination proceeds through an outer-sphere mechanism. Together, these findings are of fundamental importance, suggesting a new p*K*_a_ limit of 35 for soft nucleophiles in transition metal-catalyzed allylation reactions.

## Conflicts of interest

There are no conflicts to declare.

## Supplementary Material

SC-012-D0SC03304A-s001

## References

[cit1] Vitaku E., Smith D. T., Njardarson J. T. (2014). J. Med. Chem..

[cit2] Ma Z., Lin D. C.-H., Sharma R., Liu J., Zhu L., Li A.-R., Kohn T., Wang Y., Liu J., Bartberger M. D., Medina J. C., Zhuang R., Li F., Zhang J., Luo J., Wong S., Tonn G. R., Houze J. B. (2016). Bioorg. Med. Chem. Lett..

[cit3] Campagnuolo C., Fattorusso C., Fattorusso E., Ianaro A., Pisano B., Taglialatela-Scafati O. (2003). Org. Lett..

[cit4] Loverling F., Bikker J., Humblet C. J. (2009). Med. Chem..

[cit5] Zhai D. D., Zhang X.-Y., Liu Y.-F., Zheng L., Guan B.-T. (2018). Angew. Chem., Int. Ed..

[cit6] Gao K., Yamamoto K., Nogi K., Yorimitsu H. (2017). Synlett.

[cit7] Nadin A., Hattotuwagama C., Churcher I. (2012). Angew. Chem., Int. Ed..

[cit8] Foley D. J., Nelson A., Marsden S. P. (2016). Angew. Chem., Int. Ed..

[cit9] Trost B. M., Thaisrivongs D. A. (2008). J. Am. Chem. Soc..

[cit10] Liu X. J., You S. L. (2017). Angew. Chem., Int. Ed..

[cit11] Murakami R., Sano K., Iwai T., Taniguchi T., Monde M., Sawamura M. (2018). Angew. Chem., Int. Ed..

[cit12] Sha S. C., Zhang J., Carroll P., Walsh P. J. (2013). J. Am. Chem. Soc..

[cit13] Waetzig S. R., Tunge J. A. (2007). J. Am. Chem. Soc..

[cit14] Moon P. J., Wei Z., Lundgren R. J. (2018). J. Am. Chem. Soc..

[cit15] Zhang Z., Collum D. B. (2017). J. Org. Chem..

[cit16] Lansakara A. I., Mariappan S. V., Pigge F. C. (2016). J. Org. Chem..

[cit17] Bordwell F. (1988). Acc. Chem. Res..

[cit18] Trost B. M., Van Vranken D. L. (1996). Chem. Rev..

[cit19] Krow G. R., Lee Y. B., Raghavachari R., Szczepanski S. W., Alston P. V. (1991). Tetrahedron.

[cit20] Yamaguchi R., Nakazono Y., Matsuki T., Hata E., Kawanisi M. (1987). Bull. Chem. Soc. Jpn..

[cit21] Minami I., Takahashi K., Shimizu I., Kimura T., Tsuji J. (1986). Tetrahedron.

[cit22] The use of methallyl and allyl chloroformates in the cross-over experiment is complicated by the different rate of oxidative addition across the allyl and methallyl fragments. We chose to use methallyl because CD_3_-methallyl alcohol is readily prepared

[cit23] For our purposes, the p*K*_a_ range of 26–35 is defined 4-benzylpyridine and 4-methylpyridine. It does not take into account acidifying effects due to electron-withdrawing groups

[cit24] Comins D. L. (1983). Tetrahedron Lett..

[cit25] The relatively low yield is partly due to material losses during preparation of the stereochemical probe, which involves somewhat unstable intermediates

[cit26] For a discussion on stereochemical aspects of palladium-catalyzed allylic substitutions see: Hartwig J., Organotransition Metal Chemistry: From Bonding to Catalysis, University Science Books, Sausalito, CA, 2009; ch. 20, pp. 974–977

[cit27] Johns A. M., Utsunomiya M., Incarvito C. D., Hartwig J. F. (2006). J. Am. Chem. Soc..

[cit28] This inference is possible since the rate of oxidative addition to a neutral allyl carbamate (as in ADHPs prepared with allylchloroformate) should be slower than the rate of oxidative addition to an allylic electrophile with a cationic leaving group (*i.e.*III in Scheme 3)

[cit29] He G., Lu G., Guo Z., Liu P., Chen G. (2016). Nat. Chem..

[cit30] Kluger R. (2015). Acc. Chem. Res..

[cit31] Gonzales-James O. M., Singleton D. A. (2010). J. Am. Chem. Soc..

[cit32] Kong D., Moon P. J., Lui E. K. J., Bsharat O., Lundgren R. J. (2020). Science.

[cit33] Dai C., Fu G. C. (2001). J. Am. Chem. Soc..

[cit34] Hua X., Masson-Makdissi J., Sullivan R. J., Newman S. G. (2017). Org. Lett..

