# Supplementary material for: Pyridylic anions are soft nucleophiles in the palladium-catalyzed C(sp3)–H allylation of 4-alkylpyridines
Source: Chem Sci. 2020 Dec 10;12(4):1503–12. doi: 10.1039/d0sc03304a (PMC8179045; doi:10.1039/d0sc03304a)
Supplement: SC-012-D0SC03304A-s001 [file SC-012-D0SC03304A-s001.pdf]

Electronic Supplementary Material (ESI) for Chemical Science.
This journal is © The Royal Society of Chemistry 2020

Pyridylic Anions are Soft Nucleophiles in the Palladium-Catalyzed C(sp^3)-H Allylation of 4-Alkylpyridines.

Nour Wasfy,[†] Faizan Rasheed,[†] Raphaël Robidas,[§] Isabelle Hunter,[†] Jiaqi Shi,[†] Brian Doan,[†] Claude Legault,*[§] Dan Fishlock[‡] and Arturo Orellana*[†]

[†] Department of Chemistry, York University. 4700 Keele Street, Toronto ON, Canada M3J 1P3

[§]Department of Chemistry, Centre in Green Chemistry and Catalysis, University of Sherbrooke. 2500 Boulevard de l'Universite, Sherbrooke, QC, Canada, J1K 2R1

[‡] Process Chemistry and Catalysis, Small Molecule Technical Development. F. Hoffmann-La Roche Ltd. 4070 Basel Switzerland.

claude.legault@usherbrooke.ca

aorellan@yorku.ca

Contents

I.	General Experimental	3
II.	Preparation of Substrates	4-49
III.	Reaction Development	50-52
IV.	Palladium-Catalyzed Allylation of 4-Alkylpyridines	53-86
V.	Cross-Over Experiment	87-95
VI.	Stereochemical Probe	96-100
VII.	Allylation Using [(Xantphos)Pd(η3-allyl)]OTf	101-102
VIII.	Anionic vs Neutral Cross-Over Experiment	103
IX.	NMR Reaction Progress Experiments	104-107
X.	^{1}H-, ^{2}H-, ^{13}C-, ^{29}Si- and ^{31}P – NMR spectra	108-213
XI.	Computational Method	214
XII.	Computational Study	215
XIII.	Extended Reaction Map	216-217
XIV.	Cartesian Coordinates of Computed Structures	218-254
XV.	References	255-256

General Experimental.

All reactions were conducted in flame- or oven-dried glassware under an atmosphere of argon using anhydrous solvents unless specified otherwise. Tetrahydrofuran (THF), diethylether (Et_2O), dichloromethane (DCM) and toluene were dried using an INERT® PureSolv solvent purification system. Commercial reagents were used as received. Thin-layer chromatography was performed on SiliCycle® silica gel 60 F254 plates. Visualization was carried out using UV light (254 nm) and/or $KMnO_4$, $(NH_4)_2Ce(NO_3)_6$, vanillin, or anisaldehyde stains. Flash column chromatography[1] was carried out using SiliCycle® Silia*Flash*® silica gel (230-400 mesh, 40- 63 µ, 60 Å pore size). Hexanes (ACS grade) and ethyl acetate (ACS grade) were used as received. ^{1}H-NMR and ^{13}C-NMR spectra were recorded on a Bruker 400 AV, Bruker DRX 600 or Bruker 300 AV spectrometer in chloroform-d (99.8 % deuterated). Spectra recorded using chloroform were calibrated to 7.26 ppm ^{1}H and 77.16 ppm ^{13}C. ^{29}Si-NMR spectra were obtained at 60 MHz and chemical shifts are reported relative to a TMS external standard. ^{31}P-NMR spectra were obtained at 122 MHz and chemical shifts are reported relative to an 85% H_3PO_4 external standard. Chemical shifts (δ) are reported in ppm and multiplicities are indicated by s (singlet), d (doublet), t (triplet), q (quartet), p (quintet), sext (sextet), td (triplet of doublets), tt (triplet of triplets), dd (doublet of doublets), dddd (doublet of doublet of doublet of doublets),[2] m (multiplet), and br (broad). Coupling constants *J* are reported in Hertz (Hz). Infrared (IR) spectra were recorded as thin films (neat) using AlphaPlatinum ATR, Bruker, diamond crystal FT-IR instrument.

Preparation of Substrates: Procedures and Structural Data.

Procedure 1: *Alkylation of 4-methylpyridine*

A flame-dried round-bottomed flask equipped with a stir bar was charged with freshly distilled diisopropylamine (1.1 equiv.) in THF and cooled to −78 °C. To this solution, *n*-BuLi (1.0 equiv., 1.6 M in hexanes) was added dropwise and the mixture was stirred for 15 min to form LDA. A solution of 4-methylpyridine (1.0 equiv.) in THF was introduced at the same temperature. The deprotonated picoline was transferred via cannula to a round-bottomed flask containing the electrophile (1.0 equiv.) in THF. The reaction was kept at −78 °C for 1 h and then allowed to warm slowly to room temperature overnight. The reaction was quenched with water, and the aqueous layer was extracted three times with EtOAc. The combined organics were then washed with brine, dried using $MgSO_4$ and concentrated *in vacuo*. Unless otherwise stated, the product was purified by the following method. The crude mixture was dissolved in minimal DCM and was treated with pentane. The resulting suspension was filtered through a cotton plug and the filtrate was concentrated *in vacuo* to obtain the desired product.

Procedure 2: *Palladium-catalyzed arylation of 4-picoline*[3]

A solution of 4-methylpyridine in dry THF was prepared in a flame-dried round-bottomed flask equipped with a stir bar. TMPZnCl•LiCl in THF (1.3M, 1.2 equiv.) was added at room temperature and the resulting solution was allowed to stir for 1 h. $Pd(OAc)_2$ (2.0 mol%), SPhos (4.0 mol%) and the corresponding aryl bromide (0.80 equiv.) were stirred in a separate flame-dried round-bottomed flask for 30 min and added dropwise to the reaction mixture. The resulting mixture was stirred for 1 h at 50 °C, then quenched with a mixture of saturated aqueous NH_4Cl and NH_4OH (10:1), extracted with ethyl acetate and dried over anhydrous $MgSO_4$. After filtration, the solvent was evaporated under reduced pressure and purified using flash chromatography to furnish the desired benzyl pyridine.

Procedure 3: *Alkylation of pyridines by Knochel's method*[4]

A flame-dried round-bottomed flask equipped with a stir bar was charged with the appropriate pyridine (1.0 equiv.) as a solution in THF and cooled to 0 °C. BF_3•OEt_2 (1.1 equiv.) was added dropwise and the mixture was stirred for 15 min. The reaction mixture was then cooled to –50 °C and a THF solution of the required alkyl Grignard reagent (1.3 equiv.) was added. After 30 min, chloranil (2.0 equiv.) was added, the mixture was warmed up to room temperature and stirred for 2 additional hours. The reaction was quenched with $NH_4OH_{(aq.)}$ (1.0 mL/mmol of pyridine), filtered through a pad of Celite® and extracted thrice with diethyl ether. The combined organics were washed with brine and dried with $MgSO_4$. The product was concentrated *in vacuo* and purified using flash column chromatography.

4-(2-Phenylethyl)pyridine (**1**, CAS 2116-64-5)

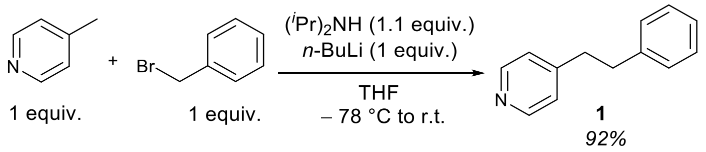

Substrate **1** was synthesized according to procedure 1 using 4-picoline (0.52 g, 5.6 mmol, 1.0 equiv.) and benzyl bromide (0.96 g, 5.6 mmol, 1.0 equiv.). The product was obtained as a yellow solid (0.94 g, 5.1 mmol) in 92% yield. Spectral data obtained is consistent with that previously reported.[5]

Data for **1**

^{1}H-NMR (400 MHz, $CDCl_3$)

δ 8.48 (d, *J* = 5.6 Hz, 2 H), 7.30-7.19 (m, 3 H), 7.15 (d, *J* = 7.6 Hz, 2 H), 7.08 (d, *J* = 5.6 Hz, 2 H), 2.93 (br, 4 H).

^{13}C-NMR (76 MHz, $CDCl_3$)

δ 150.4, 149.7, 140.6, 128.4, 128.3, 126.2, 123.9, 37.0, 36.5.

4-Pentylpyridine (**2**, CAS 2961-50-4)

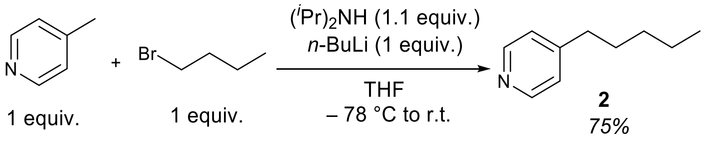

Substrate **2** was synthesized according to procedure 1 using 4-picoline (0.75 g, 8.0 mmol, 1.0 equiv.) and 1-bromobutane as the electrophile (1.11 g, 8.00 mmol, 1.0 equiv.). The product was purified by flash chromatography and obtained as a yellow oil (0.90 g, 6.0 mmol) in 75% yield. Spectral data is consistent with that previously reported.[6]

Chromatography: 20% EtOAc in hexane (R_f = 0.27).

Data for **2**

^{1}H-NMR (400 MHz, $CDCl_3$)

δ 8.47 (d, *J* = 5.6 Hz, 2 H), 7.09 (d, *J* = 5.6 Hz, 2 H), 2.59 (t, *J* = 7.6 Hz, 2 H), 1.62 (m, 2 H), 1.36-1.27 (m, 4 H), 0.89 (t, *J* = 7.2 Hz, 3 H).

^{13}C-NMR (101 MHz, $CDCl_3$)

δ 152.0, 149.8, 124.1, 35.4, 31.5, 30.2, 22.6, 14.1.

4-[(2-Methylphenyl)methyl]pyridine (**4**, CAS 36995-46-7)

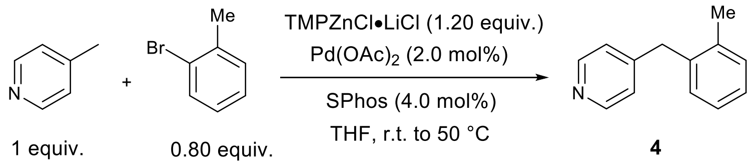

4
87%

Using procedure 2, 4-picoline (447mg, 4.80 mmol, 1.0 equiv.) and 2-bromotoluene (657 mg, 3.8 mmol, 0.80 equiv.) provided benzylpyridine **4** (765 mg, 3.31 mmol) as a colorless oil in 87% yield. Spectral data is consistent with that previously reported.[7]

Chromatography: 60% EtOAc in hexanes (R_f = 0.3).

Data for **4**

^{1}H-NMR (400 MHz, $CDCl_3$)
δ 8.51 (d, *J* = 5.6 Hz, 2 H), 7.22-7.20 (m, 3 H), 7.14-7.12 (m, 1 H), 7.07 (d, *J* = 5.6 Hz, 2 H), 4.01 (s, 2 H), 2.23 (s, 3 H).

^{13}C-NMR (101 MHz, $CDCl_3$)
δ 150.0, 149.8, 136.8, 136.7, 130.7, 130.2, 127.3, 126.4, 124.2, 39.0, 19.7.

4-[(3-Chlorophenyl)methyl]pyridine (**5**, CAS 134869-90-2)

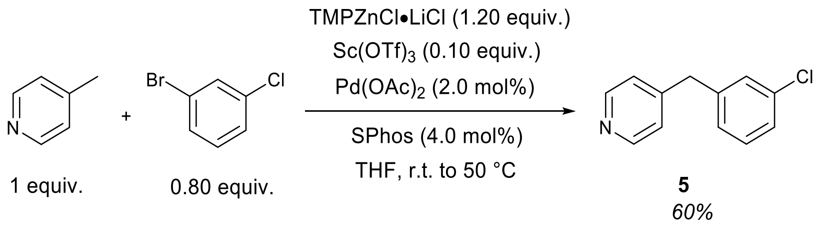

Using procedure 2, 4-picoline (559 mg, 6.00 mmol, 1.0 equiv.) was deprotonated using TMPZnCl•LiCl in THF (6 mL, 7.2mmol, 1.3M, 1.2 equiv.). $Sc(OTf)_3$ (30 mg, 0.60 mmol, 0.10 equiv.) was introduced, followed by the addition of $Pd(OAc)_2$ (27 mg, 2 mol%), SPhos (99 mg, 4 mol%) and 1-bromo-3-chlorobenzene (919 mg, 4.8 mmol, 0.80 equiv.). Benzylpyridine **5** was obtained as a yellow oil in 60% yield (733 mg, 2.88 mmol). Spectral data is consistent with that previously reported.[8]

Chromatography: 80% EtOAc in hexanes (R_f = 0.4).

Data for **5**

^{1}H-NMR (300 MHz, $CDCl_3$)
δ 8.50 (d, *J* = 6Hz, 2 H), 7.22-7.21 (m, 2 H), 7.14 (s, 1 H), 7.08-7.02 (m, 3 H), 3.92 (s, 2 H).

^{13}C-NMR (76 MHz, $CDCl_3$)
δ 150.0, 149.0, 140.9, 134.5, 130.0, 129.1, 127.2, 126.9, 124.1, 40.8.

4-(4-Pyridinylmethyl)benzonitrile (**6**, CAS 146040-04-2)

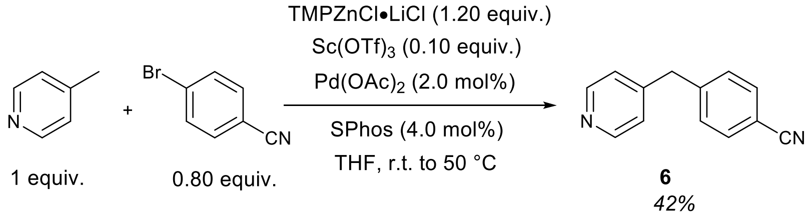

Using procedure 2, 4-picoline (652 mg, 7.00 mmol, 1.0 equiv.) was deprotonated using TMPZnCl•LiCl in THF (7 mL, 8.4 mmol, 1.3M, 1.2 equiv.). $Sc(OTf)_3$ (30 mg, 0.60 mmol, 0.10 equiv.) was introduced, followed by the addition of $Pd(OAc)_2$ (31 mg, 2 mol%), SPhos (115 mg, 4 mol%) and 4-bromobenzonitrile (1.02 g, 5.6 mmol, 0.80 equiv.). Benzylpyridine **6** was obtained as a yellow oil in 42% yield (489 mg, 2.35 mmol). Spectral data is consistent with that reported in the literature.[3]

Chromatography: 80% EtOAc in hexanes (R_f = 0.3).

Data for **6**

^{1}H-NMR (400 MHz, $CDCl_3$)
δ 8.49 (d, J = 5.2 Hz, 2 H), 7.57 (d, J = 8.0 Hz, 2 H), 7.26 (d, J = 8.4 Hz, 2 H), 7.06 (d, J = 5.6 Hz, 2 H), 4.00 (s, 2 H).

^{13}C-NMR (101 MHz, $CDCl_3$)
δ 150.0, 148.1, 144.3, 132.4, 129.7, 124.0, 118.6, 110.5, 41.0.

N,N-Dimethyl-4-(4-pyridinylmethyl)benzenamine (**7**, CAS 131416-55-2)

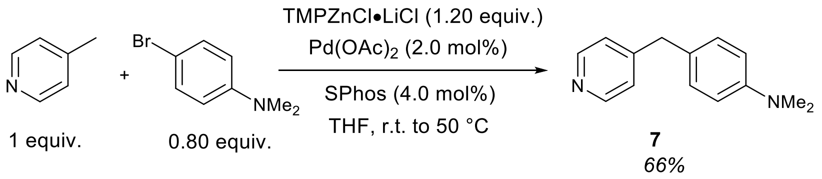

Using procedure 2, 4-picoline (559 mg, 6.00 mmol, 1.00 equiv.) and 4-bromo-*N,N*-dimethylaniline (821 mg, 4.8 mmol, 0.80 equiv.) provided benzylpyridine **7** (581 mg, 3.17 mmol) as a colorless oil in 66% yield. Spectral data is consistent with that previously reported.[3]

Chromatography: 50% EtOAc in hexanes (R_f = 0.3).

Data for **7**

^{1}H-NMR (400 MHz, $CDCl_3$)

δ 8.36 (d, *J* = 5.2 Hz, 2 H), 6.98 (d, *J* = 5.6 Hz, 2 H), 6.93 (d, *J* = 8.4 Hz, 2 H), 6.58 (d, *J* = 8.4 Hz, 2 H), 3.74 (s, 2 H), 2.80 (s, 6 H).

^{13}C-NMR (101 MHz, $CDCl_3$)

δ 150.9, 149.5, 149.3, 149.2, 129.5, 126.5, 123.9, 112.7, 40.4, 40.1.

4-(But-3-en-1-yl)pyridine (**8**, CAS 45814-04-8)

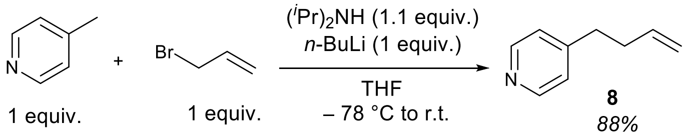

Substrate **8** was synthesized according to procedure 1 using 4-picoline (0.98 g, 10.5 mmol, 1.0 equiv.) and allyl bromide (1.30 g, 10.5 mmol, 1.0 equiv.). The product was obtained as a yellow oil (1.2 g, 9.3 mmol) in 88% yield. Spectral data is consistent with that previously reported.[9]

Data for **8**

^{1}H-NMR (400 MHz, $CDCl_3$)
δ 8.51 (d, J = 5.6 Hz, 2 H), 7.13 (d, J = 5.6 Hz, 2 H),
5.84 (dddd, J = 17.2, 10.0, 6.8, 6.8 Hz, 1 H), 5.06-4.99 (m, 2 H),
2.75-2.71 (m, 2 H), 2.44-2.38 (m, 2 H).

^{13}C-NMR (101 MHz, $CDCl_3$)
δ 150.1, 149.2, 136.5, 123.5, 115.2, 34.0, 33.7.

4-(3-Butyn-1-yl)pyridine (**9**, CAS 103440-64-8)

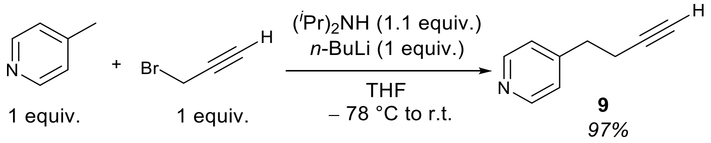

Substrate **9** was synthesized according to procedure 1 using 4-picoline (0.71 g, 7.6 mmol, 1.0 equiv.) and propargyl bromide (1.08 g, 7.6 mmol, 1.0 equiv.). The product was obtained as a white solid (0.98 g, 7.4 mmol) in 97% yield.

Data for **9**

**^{1}H-NMR**	(400 MHz, $CDCl_3$)
	δ 8.52 (dd, J = 4.4, 1.6 Hz, 2 H), 7.16 (d, J = 6.0 Hz, 2 H),
	2.84 (t, J = 7.2 Hz, 2 H), 2.52 (dt, J = 7.2, 2.8 Hz, 2 H), 1.99 (t, J = 2.8 Hz, 1 H).
**^{13}C-NMR**	(101 MHz, $CDCl_3$)
	δ 150.0, 149.2, 124.0, 82.9, 69.8, 34.1, 19.5.
**IR**	Alpha-Platinum ATR, Bruker, diamond crystal
	υ = 3180, 2955, 1602, 1558 cm^{-1}
**HRMS**	ESI
	Calculated mass for $(M+H)^+$ of C_9H_9N is 132.0808 found 132.0806.

4-(2-Bromophenethyl)pyridine (**11**)

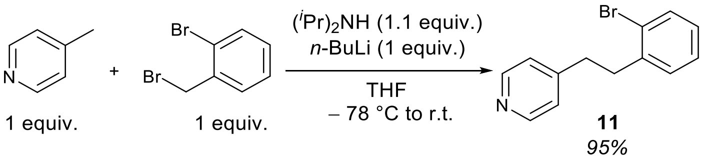

11
95%

Substrate **11** was synthesized according to procedure 1 using 4-picoline (0.36 g, 3.9 mmol, 1.0 equiv.) and 2-bromobenzyl bromide (0.98 g, 3.9 mmol, 1.0 equiv.). The product was obtained as a yellow oil (0.97 g, 3.7 mmol) in 95% yield.

Data for **11**

^{1}H-NMR (400 MHz, $CDCl_3$)

δ 8.49 (d, *J* = 6.0 Hz, 2 H), 7.55 (d, *J* = 8.0 Hz, 1 H), 7.22-7.19 (m, 1 H), 7.13-7.06 (m, 4 H), 3.06-3.02 (m, 2 H), 2.93-2.89 (m, 2 H).

^{13}C-NMR (101 MHz, $CDCl_3$)

δ 149.8, 149.5, 139.6, 132.6, 130.2, 127.8, 127.3, 124.1, 123.6, 36.8, 35.0.

IR Alpha-Platinum ATR, Bruker, diamond crystal

υ = 3066, 3023, 2930, 1599, 1559 cm^{-1}

HRMS ESI

Calculated mass for $(M+H)^+$ of $C_{13}H_{12}BrN$ is 262.0226 found 262.0223.

4-(4-Chlorobutyl)pyridine (**12**, CAS 5264-17-5)

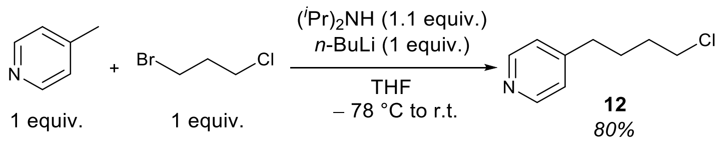

Substrate **12** was synthesized according to procedure 1 using 4-picoline (0.36 g, 3.9 mmol, 1.0 equiv.) and 1-bromo-3-chloropropane (0.98 g, 3.9 mmol, 1.0 equiv.). The product was obtained as a yellow oil (0.97 g, 3.7 mmol) in 80% yield. Spectral data is consistent with that previously reported.[10]

Chromatography: 50% EtOAc in hexanes (R_f = 0.47).

Data for **12**

^{1}H-NMR (400 MHz, $CDCl_3$)

δ 8.50 (d, *J* = 6 Hz, 2 H), 7.12 (d, *J* = 6 Hz, 2 H), 3.56 (m, 2 H), 2.65 (m, 2 H), 1.83-1.79 (m, 4 H).

^{13}C-NMR (101 MHz, $CDCl_3$)

δ 150.8, 149.8, 123.9, 44.7, 34.4, 31.9, 27.4.

4-(4-((*tert*-Butyldimethylsilyl)oxy)butyl)pyridine (13)

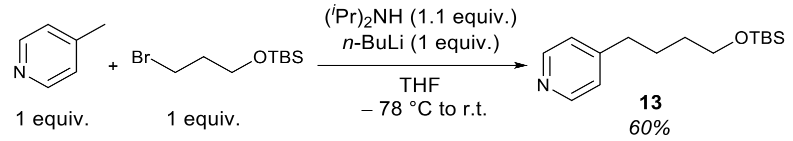

Substrate **13** was synthesized according to procedure 1 using 4-picoline (0.85 g, 9.1 mmol, 1.0 equiv.) and (3-bromopropoxy)-*tert*-butyldimethylsilane (2.30 g, 9.1 mmol, 1.0 equiv.). The product was purified by flash chromatography and obtained as a yellow oil (1.60 g, 5.48 mmol) in 60% yield.

Chromatography: 40% EtOAc in hexane (R_f. = 0.56).

Data for **13**

^{1}H-NMR (400 MHz, $CDCl_3$)
δ 8.48 (d, *J* = 5.6 Hz, 2 H), 7.10 (d, *J* = 5.6 Hz, 2 H), 3.62 (t, *J* = 6.4 Hz, 2 H), 2.62 (t, *J* = 7.6 Hz, 2 H), 1.78-1.66 (m, 2 H), 1.57-1.52 (m, 2 H), 0.91 (s, 9 H), 0.06 (s, 6 H).

^{13}C-NMR (76 MHz, $CDCl_3$)
δ 151.2, 149.4, 123.6, 62.4, 34.7, 31.9, 26.3, 25.7, 18.1, -5.5.

^{29}Si-NMR (57 MHz, $CDCl_3$)
δ 18.74.

IR Alpha-Platinum ATR, Bruker, diamond crystal
υ = 2894, 2857, 1602 cm^{-1}

HRMS ESI
Calculated mass for $(M+H)^+$ of $C_{15}H_{27}NOSi$ is 266.1935 found 266.1928.

4-(3-(1,3-Dioxolan-2-yl)propyl)pyridine (**14**, CAS 639089-29-5)

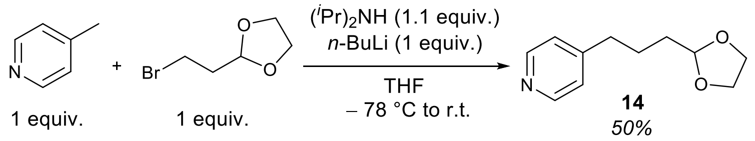

14
50%

Substrate **14** was synthesized according to procedure 1 using 4-picoline (1.06 g, 11.4 mmol, 1.0 equiv.) and 2-(2-bromoethyl)-1,3-dioxolane (2.06 g, 12.5 mmol, 1.1 equiv.). The product was purified by flash chromatography and obtained as a yellow oil (1.10 g, 5.69 mmol) in 50% yield. Spectral data is consistent with that previously reported.[11]

Chromatography: 100% EtOAc ($R_{f.}$ = 0.50).

Data for **14**

^{1}H-NMR (400 MHz, $CDCl_3$)

δ 8.48 (d, *J* = 5.6 Hz, 2 H), 7.11 (d, *J* = 5.6 Hz, 2 H), 4.87 (t, *J* = 4.4 Hz, 1 H), 3.99-3.93 (m, 2 H), 3.92-3.81 (m, 2 H), 2.65 (t, *J* = 7.2 Hz, 2 H), 1.79-1.68 (m, 4 H).

^{13}C-NMR (101 MHz, $CDCl_3$)

δ 151.1, 149.8, 123.9, 104.2, 65.0, 35.0, 33.3, 24.6.

4-[2-(4-Morpholinyl)ethyl]pyridine (**15**, CAS 28487-18-5)

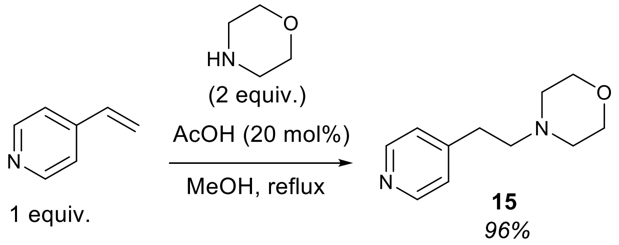

To a 25 mL round-bottomed flask equipped with a stir bar was added 4-vinylpyridine (0.60 mL, 5.5 mmol, 1.0 equiv.), morpholine (0.96 mL, 11.0 mmol, 2.0 equiv.) and MeOH (7 mL). AcOH (0.06 mL, 1.1 mmol, 20 mol%) was then added and the mixture was heated to reflux overnight. The reaction mixture was then diluted with EtOAc and neutralized using a saturated solution of $NaHCO_3$. The aqueous phase was extracted three times with EtOAc and the combined organics were washed with brine, dried using $MgSO_4$, and concentrated *in vacuo*. The product was purified using flash chromatography and obtained as a yellow solid (1.2 g, 5.3 mmol) in 96% yield. Spectral data is consistent with that previously reported.[12]

Chromatography: 5% Et_3N : 3.3% MeOH : 91.7% EtOAc (R_f. = 0.33).

Data for **15**

^{1}H-NMR (400 MHz, $CDCl_3$)
δ 8.49 (dd, J = 4.4, 1.6 Hz, 2 H), 7.14 (d, J = 6.0 Hz, 2 H), 3.73 (t, J = 4.4 Hz, 4 H), 2.80 (dd, J = 8.4, 5.6 Hz, 2 H), 2.61 (dd, J = 8.4, 5.6 Hz, 2 H), 2.51 (t, J = 4.4 Hz, 4 H).

^{13}C-NMR (101 MHz, $CDCl_3$)
δ 149.6, 149.1, 124.0, 66.8, 59.2, 53.5, 32.5.

4-Pyridineheptanenitrile (**16**, CAS 154939-07-8)

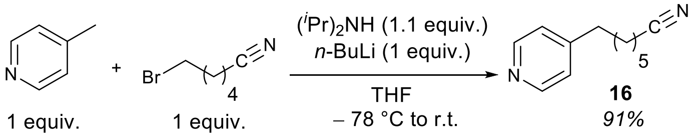

Substrate **16** was synthesized according to procedure 1 using 4-picoline (0.50 g, 5.4 mmol, 1.0 equiv.) and 6-bromohexanenitrile (0.95 g, 5.4 mmol, 1.0 equiv.). The product was obtained as a yellow oil (0.92 g, 4.9 mmol) in 91% yield. Spectral data is consistent with that previously reported.[13]

Data for **16**

^{1}H-NMR (400 MHz, $CDCl_3$)

δ 8.49 (dd, *J* = 5.0, 1.6 Hz, 2 H), 7.09 (d, *J* = 5.6 Hz, 2 H), 2.61 (t, *J* = 7.6 Hz, 2 H), 2.34 (t, *J* = 7.2 Hz, 2 H), 1.69-1.62 (m, 4 H), 1.53-1.45 (m, 2 H), 1.40-1.37 (m, 2 H).

^{13}C-NMR (101 MHz, $CDCl_3$)

δ 151.1, 149.5, 123.7, 119.6, 34.9, 29.8, 28.2, 28.1, 25.1, 17.0.

Ethyl 3-(pyridin-4-yl)propanoate (**17**, CAS 52809-19-5)

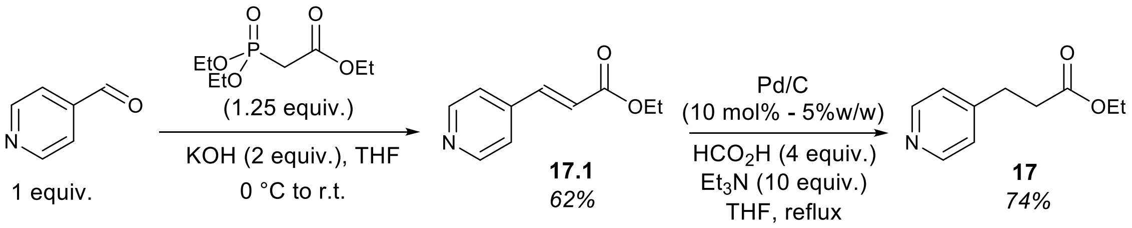

17.1 *62%* **17** *74%*

Horner-Wadsworth-Emmons Reaction - A flame-dried round-bottomed flask equipped with a stir bar was charged with finely-ground KOH (0.56 g, 10 mmol, 2.0 equiv.) and THF (10 mL), and cooled to 0 °C. Triethyl phosphonoacetate (1.40 g, 6.25 mmol, 1.25 equiv.) in THF (7 mL) was added and the mixture was stirred for 30 min. A solution of 4-Pyridinecarboxaldehyde (0.54 g, 5.0 mmol, 1.0 equiv.) in THF (7 mL) was added and the reaction mixture was warmed to room temperature and allowed to stir overnight. The reaction mixture was diluted with diethyl ether and dried with $MgSO_4$. Upon filtration through a pad of Celite® and concentration, crude product **17.1** (CAS: 24489-96-1) was obtained as a white solid. Purification using a short plug of silica using EtOAc as eluent (0.55 g, 3.1 mmol) in provided **17.1** in 62% yield. Spectral data is consistent with that previously reported.[14]

Data for **17.1**

^{1}H-NMR (400 MHz, $CDCl_3$)

δ 8.64 (d, *J* = 5.6 Hz, 2 H), 7.59 (d, *J* = 16.0 Hz, 1 H), 7.36 (d, *J* = 5.6 Hz, 2 H), 6.58 (d, *J* = 16.0 Hz, 1 H), 4.28 (q, *J* = 7.2 Hz, 2 H), 1.34 (t, *J* = 7.2 Hz, 3 H).

^{13}C-NMR (101 MHz, $CDCl_3$)

δ 166.2, 150.8, 141.8, 123.1, 122.0, 61.2, 14.4.

Ethyl 3-(pyridin-4-yl)propanoate (**17**, CAS 52809-19-5)

Hydrogenation - A flame-dried round-bottomed flask equipped with a stir bar was charged with a THF (17 mL) solution of α,β-unsaturated ester **17.1** (0.90 g, 5.1 mmol, 1.0 equiv.), triethylamine (7.1 mL, 51 mmol, 10.0 equiv.), 5% w/w Pd/C (1.1 g, 0.51 mmol, 10 mol%) and formic acid (0.77 mL, 20 mmol, 4.0 equiv.). The flask was then equipped with a condenser and the reaction mixture was heated to reflux for 4 h. The reaction mixture was then diluted with EtOAc and filtered through a pad of Celite® to remove the palladium catalyst. The filtrate was concentrated *in vacuo* and subsequently flushed through a plug of silica to afford product **17** as a yellow oil (0.68 g, 3.8 mmol) in 74% yield. Spectral data is consistent with that previously reported.[15]

Data for **17**

^{1}H-NMR (400 MHz, $CDCl_3$)

δ 8.51 (d, *J* = 5.6 Hz, 2 H), 7.14 (d, *J* = 5.6 Hz, 2 H), 4.13 (q, *J* = 7.2 Hz, 2 H), 2.95 (t, *J* = 7.6 Hz, 2 H), 2.64 (t, *J* = 7.6 Hz, 2 H), 1.23 (t, *J* = 7.2 Hz, 3 H).

^{13}C-NMR (101 MHz, $CDCl_3$)

δ 172.1, 149.8, 149.4, 123.6, 60.6, 34.4, 30.0, 14.1.

4-(Pyridin-4-yl)butan-2-one (**18**, CAS 35250-71-6)

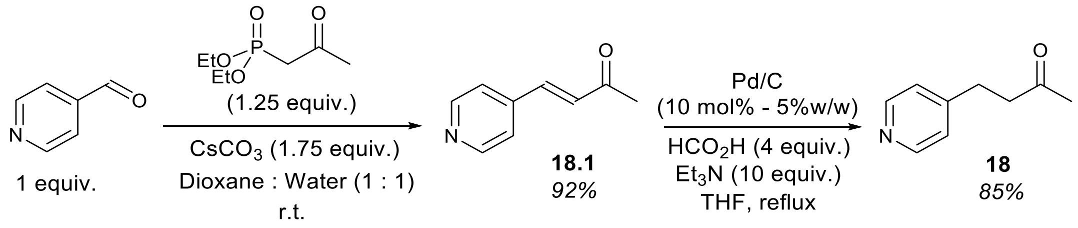

Horner-Wadsworth-Emmons Reaction – A round bottomed flask equipped with a stir-bar was charged with Cs_2CO_3 (2.85 g, 8.75 mmol, 1.75 equiv.) and a 1:1 mixture of 1,4-dioxane and distilled water (10 mL). Diethyl (2-oxopropyl)phosphonate (1.04 g, 6.25 mmol, 1.25 equiv.) was added and the resulting mixture was stirred for 30 min. 4-Pyridinecarboxaldehyde (0.54 g, 5.0 mmol, 1.0 equiv.) was added neat. Upon completion of the reaction, as indicated by TLC, the mixture was diluted with EtOAc and washed thrice with brine. The organic layer was dried with $MgSO_4$ and concentrated *in vacuo*. The crude mixture was flushed through a plug of silica gel using EtOAc to afford **18.1** (CAS: 10416-53-2) as brown solid (0.68 g, 4.60 mmol) in 92% yield. Spectral data is consistent with that previously reported.[16]

Data for **18.1**

^{1}H-NMR (400 MHz, $CDCl_3$)
δ 8.66 (d, J = 5.6 Hz, 2 H), 7.41 (d, J = 16.4 Hz, 1 H), 7.38 (d, J = 5.6 Hz, 2 H), 6.84 (d, J = 16.4 Hz, 1 H), 2.41 (s, 3 H).

^{13}C-NMR (101 MHz, $CDCl_3$)
δ 197.9, 150.9, 141.9, 140.3, 130.9, 122.1, 28.1.

4-(Pyridin-4-yl)butan-2-one (**18**, CAS 35250-71-6)

Hydrogenation – A flame-dried round-bottomed flask equipped with a stir bar was charged with THF solution (17 mL) of α,β-unsaturated ketone **18.1** (0.44 g, 3.0 mmol, 1.0 equiv.), triethylamine (4.0 mL, 30 mmol, 10.0 equiv.), 5% w/w Pd/C (0.64 g, 0.29 mmol, 10 mol%) and formic acid (0.55 mL, 12 mmol, 4.0 equiv.). The flask was then equipped with a condenser and heated to reflux for 4 h. The reaction mixture was then diluted with EtOAc and filtered through a pad of Celite®. The filtrate was concentrated *in vacuo* and subsequently passed through a plug of silica gel to afford **18** as a yellow oil (0.38 g, 2.6 mmol) in 85% yield. Spectral data is consistent with that reported in the literature.[6]

Data for **18**

^{1}H-NMR (400 MHz, $CDCl_3$)
δ 8.49 (d, *J* = 5.2 Hz, 2 H), 7.11 (d, *J* = 5.2 Hz, 2 H), 2.89 (t, *J* = 7.2 Hz, 2 H), 2.77 (t, *J* = 7.2 Hz, 2 H), 2.16 (s, 3 H).

^{13}C-NMR (101 MHz, $CDCl_3$)
δ 206.9, 150.1, 149.9, 124.2, 43.7, 30.1, 28.9.

4-(Pyridin-4-yl)butanal (**19**, CAS 192643-84-8)

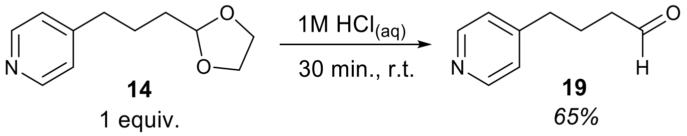

Acetal **14** (0.35 g, 1.8 mmol, 1.0 equiv.) was dissolved in 1 M $HCl_{(aq)}$ (10 mL), the resulting solution was stirred for 30 min at room temperature and subsequently washed twice with DCM. The aqueous solution was then neutralized using saturated aqueous $NaHCO_3$ solution and extracted three times with DCM. The combined organics were washed with brine, dried over $MgSO_4$, filtered and concentrated *in vacuo* to afford **19** as a colorless oil (0.17 g, 1.2 mmol) in 65% yield which was used without further purification. Spectral data is consistent with that previously reported.[10]

Data for **19**

^{1}H-NMR (400 MHz, $CDCl_3$)

δ 9.77 (s, 1 H), 8.49 (d, *J* = 5.2 Hz, 2 H), 7.10 (d, *J* = 5.2 Hz, 2 H), 2.64 (t, *J* = 7.6 Hz, 2 H), 2.47 (t, *J* = 7.2 Hz, 2 H), 1.96 (tt, *J* = 7.6, 7.2 Hz, 2 H).

^{13}C-NMR (101 MHz, $CDCl_3$)

δ 201.3, 150.0, 149.4, 123.6, 42.6, 33.9, 22.1.

4-Butyl-3-phenylpyridine (20)

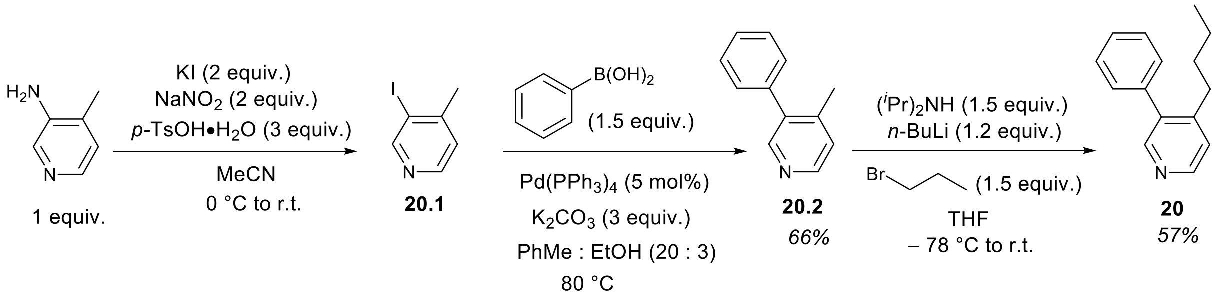

Synthesis of 3-iodo-4-methylpyridine **20.1** (CAS: 38749-96-1)[17] – To a solution of 3-amino-4-methylpyridine (0.40 g, 3.7 mmol, 1.0 equiv.) and *p*-TsOH•H_2O (2.11 g, 11.1 mmol, 3.0 equiv.) in MeCN (15 mL) was added dropwise a solution of $NaNO_2$ (0.51 g, 7.4 mmol, 2.0 equiv.) and KI (1.23 g, 7.40 mmol, 2.0 equiv.) in distilled water (5 mL) at 0 °C over 15 min. After 1 h the reaction mixture was warmed to room temperature, and then treated with saturated aqueous $NaHCO_3$ (30 mL) and saturated aqueous $NaHSO_3$ (30 mL). The aqueous phase was extracted with EtOAc three times and the combined organics were washed with brine, dried with $MgSO_4$ and concentrated *in vacuo* to afford **20.1** as a yellow oil, which was used in the next step without further purification.

Suzuki Coupling – A flame-dried round-bottomed flask equipped with a stir bar was charged with **20.1**, phenylboronic acid (0.60 g, 4.9 mmol, 1.5 equiv.), K_2CO_3 (1.37 g, 9.90 mmol, 3.0 equiv.) and $Pd(PPh_3)_4$ (0.19 g, 0.16 mmol, 5 mol%) and kept under an atmosphere of argon. To this mixture was added freshly distilled toluene (20 mL) and anhydrous EtOH (3 mL), and the resulting solution was heated to 80 °C. After 3 h, the reaction mixture was allowed to cool to room temperature, diluted with EtOAc and washed with brine three times. The organic layer was dried with $MgSO_4$ and concentrated *in vacuo*. The crude mixture was purified by flash chromatography to afford **20.2** as a colorless oil (0.37 g, 2.2 mmol) in 66% yield. Spectral data is consistent with that previously reported.[18]

Chromatography: 30% EtOAc in Toluene (R_f. = 0.33).

Data for **20.2**

^{1}H-NMR (400 MHz, $CDCl_3$)

δ 8.44 (br, 2 H), 7.47-7.34 (m, 3 H), 8.37 (d, *J* = 7.6 Hz, 2 H), 7.19 (d, *J* = 4.8 Hz, 1 H), 2.29 (s, 3 H).

^{13}C-NMR (101 MHz, $CDCl_3$)

δ 149.8, 148.2, 144.4, 137.8, 137.6, 129.2, 128.3, 127.5, 125.1, 19.7.

4-Butyl-3-phenylpyridine (**20**)

*Alkylation of **20.2*** – Substrate **20** was synthesized according to procedure 1 using pyridine **20.2** (0.30 g, 1.8 mmol, 1.0 equiv.) and 1-bromopropane (0.33 g, 2.7 mmol, 1.5 equiv.). Purification by flash column chromatography provided pyridine **20** as a colorless oil (0.32 g, 1.0 mmol) in 57% yield.

Chromatography: 40% EtOAc in hexanes ($R_{f.}$ = 0.40).

Data for **20**

^{1}H-NMR (400 MHz, $CDCl_3$)
δ 8.47 (d, *J* = 4.8 Hz, 1 H), 8.41 (s, 1 H), 7.45-7.36 (m, 3 H), 7.30-7.28 (m, 2 H), 7.19 (d, *J* = 4.8 Hz, 1 H), 2.58 (t, *J* = 7.6 Hz, 2 H), 1.46 (p, *J* = 7.6 Hz, 2 H), 1.22 (sext, *J* = 8 Hz, 2 H), 0.79 (t, *J* = 7.2 Hz, 3 H).

^{13}C-NMR (101 MHz, $CDCl_3$)
δ 150.1, 149.1, 148.4, 137.9, 137.5, 129.3, 128.3, 127.5, 123.8, 32.2, 31.9, 22.3, 13.7.

IR Alpha-Platinum ATR, Bruker, diamond crystal
υ = 2957, 2929, 2861, 1597 cm^{-1}

HRMS ESI
Calculated mass for $(M+H)^+$ of $C_{15}H_{17}N$ is 212.1434 found 212.1430.

3-Methyl-4-(phenylmethyl)pyridine (**21**, CAS 24015-80-3)

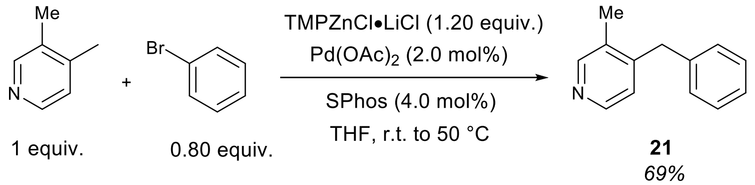

Using procedure 2, 3,4-lutidine (643 mg, 6.00 mmol, 1.00 equiv.) and bromobenzene (754 mg, 4.8 mmol, 0.80 equiv.) provided benzylpyridine **21** (758 mg, mmol) as a colorless oil in 69% yield. Spectral data is consistent with that previously reported.[7]

Chromatography: 60% EtOAc in hexanes (R_f = 0.3).

Data for **21**

^{1}H-NMR (400 MHz, $CDCl_3$)
δ 8.35-8.33 (m, 2H), 7.73-7.21 (m, 3H), 7.10 (d, *J* = 5.6Hz, 2H), 6.95 (d, *J* = 5.2Hz, 1H), 3.94 (s, 2H), 2.23 (s, 3H).

^{13}C-NMR (76 MHz, $CDCl_3$)
δ 150.8, 148.2, 147.8, 138.3, 132.3, 129.0, 128.8, 126.7, 124.5, 38.9, 16.5.

4-Butyl-3-pyridinecarbonitrile (**22**, CAS 1713287-19-4)

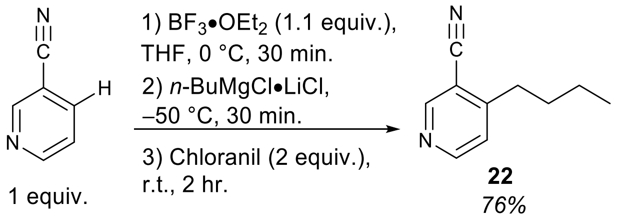

Substrate **22** was synthesized according to procedure 2 using 3-cyanopyridine (0.78 g, 7.5 mmol, 1.0 equiv.) and *n*-BuMgCl•LiCl (4.87 mL, 9.74 mmol, 2.0 M, 1.3 equiv.). The product was purified by flash chromatography and obtained as a colorless oil (0.91 g, 5.7 mmol) in 76% yield.

Chromatography: 20% EtOAc in hexanes (R_f. = 0.40).

Data for **20**

^{1}H-NMR (400 MHz, $CDCl_3$)
δ 8.77 (s, 1 H), 8.64 (d, *J* = 5.2 Hz, 1 H), 7.26 (d, *J* = 5.2 Hz, 1 H), 2.82 (t, *J* = 7.6 Hz, 2 H), 1.70-1.62 (m, 2 H), 1.44-1.35 (m, 2 H), 0.97 (t, *J* = 7.2 Hz, 3 H).

^{13}C-NMR (101 MHz, $CDCl_3$)
δ 155.6, 153.1, 152.7, 124.1, 116.1, 110.6, 34.0, 32.1, 22.4, 13.9.

IR Alpha-Platinum ATR, Bruker, diamond crystal
υ = 2958, 2932, 2872, 2228, 1589, 1553 cm^{-1}

HRMS ESI
Calculated mass for $(M+H)^+$ of $C_{10}H_{12}N_2$ is 161.1073 found 161.1077.

Ethyl 4-butylnicotinate (23)

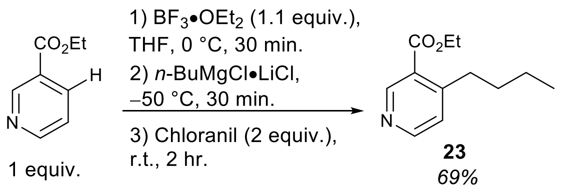

Substrate **23** was prepared according to procedure 2 using 3-ethyl nicotinate (0.76 g, 5.0 mmol, 1.0 equiv.) and *n*-BuMgCl•LiCl (11 mL, 7.9 mmol, 1.6 equiv., 0.7 M in THF). The product was purified by flash chromatography and obtained as a colorless oil (0.71 g, 3.5 mmol) in 69% yield.

Chromatography: 3.33% Et_3N in hexanes (R_f. = 0.20).

Data for **23**

^{1}H-NMR (400 MHz, $CDCl_3$)
δ 9.03 (s, 1 H), 8.56 (d, *J* = 5.1 Hz, 1 H), 7.17 (d, *J* = 5.1 Hz, 1 H), 4.39 (q, *J* = 7.2 Hz, 2 H), 2.97 (t, *J* = 7.8 Hz, 2 H), 1.63-1.55 (m, 2 H), 1.45-1.36 (m, 5 H), 0.94 (t, *J* = 7.2 Hz, 3 H).

^{13}C-NMR (101 MHz, $CDCl_3$)
δ 166.3, 153.7, 152.1, 151.7, 126.1, 125.4, 61.3, 33.6, 33.0, 22.8, 14.3, 14.0.

IR Alpha-Platinum ATR, Bruker, diamond crystal
υ = 2959, 2932, 2872, 1719, 1590, 1556 cm^{-1}

HRMS ESI
Calculated mass for $(M+H)^+$ of $C_{12}H_{17}NO_2$ is 208.1332 found 208.1326.

***N*-(*tert*-Butyl)-4-butylnicotinamide** (**24**, CAS 331969-16-5)

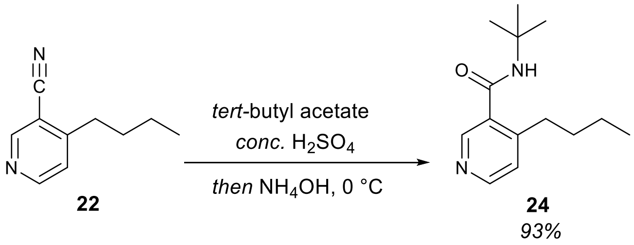

To a round-bottomed flask containing a solution of 3-cyano-4-butylpyridine (0.40 g, 2.5 mmol, 1.0 equiv.) in *tert*-butyl acetate (5 mL) was added concentrated H_2SO_4 (1 mL), and the mixture was stirred at room temperature overnight. The solution was then diluted with water and carefully neutralized by adding NH_4OH (3 mL) at 0 °C. The aqueous phase was extracted three times with a 1:1 mixture of hexane-EtOAc and the combined organics were washed with brine, dried with $MgSO_4$, filtered, and concentrated *in vacuo*. The residue was purified using flash column chromatography on silica gel to afford the product as a white solid (0.54 g, 2.3 mmol) in 93% yield. Spectral data is consistent with that previously reported.[19]

Chromatography: 70%-90% EtOAc in hexanes (R_f. = 0.34).

Data for **24**

^{1}H-NMR (400 MHz, $CDCl_3$)
δ 8.50 (s, 1 H), 8.48 (d, *J* = 5.2 Hz, 1 H), 7.14 (d, *J* = 5.2 Hz, 1 H), 5.64 (br, 1 H), 2.76 (t, *J* = 7.6 Hz, 2 H), 1.59 (p, *J* = 7.6 Hz, 2 H), 1.47 (s, 9 H), 1.37 (sext, *J* = 8 Hz, 2 H), 0.93 (t, *J* = 7.2 Hz, 3 H).

^{13}C-NMR (101 MHz, $CDCl_3$)
δ 167.4, 150.7, 150.3, 147.5, 133.8, 124.9, 52.4, 32.9, 32.5, 29.0, 22.9, 14.1.

***tert*-Butyl (4-phenethylpyridin-3-yl)carbamate (25)**

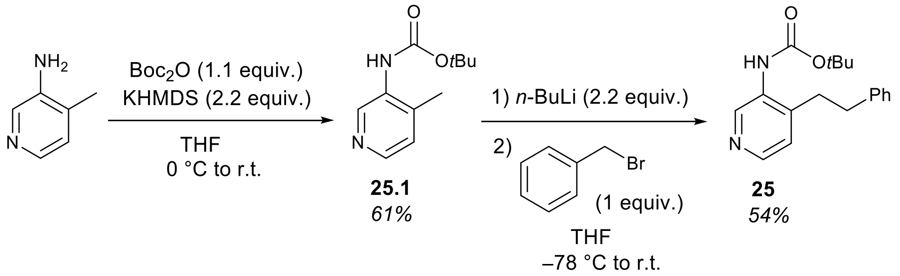

<u>*Boc-protection of 3-amino-4-methylpyrdine* (**25.1** CAS 180253-66-1)</u> – A flame-dried round-bottomed flask equipped with a stir bar was charged with 3-amino-4-methylpyridine (0.50 g, 4.6 mmol, 1.0 equiv.) and anhydrous THF (10 mL), and cooled to 0 °C. A solution of KHMDS (20.2 mL, 10.2 mmol, 2.2 equiv., 0.5 M in toluene) was introduced drop-wise, the reaction mixture was stirred for 30 min at 0 °C and then allowed to warm to ambient temperature. A solution of di-*tert*-butyl dicarbonate (1.10 g, 5.09 mmol, 1.1 equiv.) in dry THF (10 mL) was added, and the reaction was monitored by TLC. Upon completion, the reaction mixture was concentrated *in vacuo* then quenched with of 0.1 M $HCl_{(aq)}$ (20 mL). The aqueous phase was extracted with DCM three times and the combined organics were washed with brine, dried with $MgSO_4$ and concentrated *in vacuo*. The crude mixture was purified by silica gel chromatography to afford **25.1** as yellow solid (0.58 g, 2.8 mmol) in 61% yield. The spectral data was consistent with that previously reported.[20]

Chromatography: 60% EtOAc in hexanes (R_f. = 0.40).

Data for **25.1**

<u>**^{1}H-NMR**</u> (400 MHz, $CDCl_3$)
δ 8.82 (br, 1 H), 8.21 (d, *J* = 4.8 Hz, 1 H), 7.06 (d, *J* = 4.8 Hz, 1 H), 6.52 (br, 1 H), 2.24 (s, 3 H), 1.49 (s, 9 H).

<u>**^{13}C-NMR**</u> (101 MHz, $CDCl_3$)
δ 152.9, 145.1, 143.9, 138.0, 133.4, 125.0, 80.9, 28.2, 17.3.

***tert*-Butyl (4-phenethylpyridin-3-yl)carbamate** (**25**)

*Alkylation of **25.1*** – A flame-dried round-bottomed flask equipped with a stir bar was cooled to –78 °C and charged with *n*-BuLi (1.90 mL, 4.75 mmol, 2.2 equiv., 2.5 M in hexanes) and dry THF (20 mL) under an atmosphere of argon. A solution of **25.1** (0.45 g, 2.2 mmol, 1.0 equiv.) in dry THF (5 mL) was then added dropwise and the resulting mixture was allowed to stir for 1 h. Benzyl bromide (0.37 g, 2.2 mmol, 1.0 equiv.) was introduced and the solution was maintained at –78 °C for 1 h, then allowed to warm to ambient temperature. Upon completion as indicated by TLC, the reaction was quenched with water and the aqueous phase was extracted with EtOAc three times. The combined organics were washed with brine, dried with $MgSO_4$ and concentrated *in vacuo*. The crude mixture was purified by flash chromatography to afford **25** as a white solid (0.35 g, 1.2 mmol) in 54% yield.

Chromatography: 50-65% EtOAc in Hexane ($R_{f.}$ = 0.30).

Data for **25**

^{1}H-NMR (400 MHz, $CDCl_3$)
δ 8.78 (br, 1 H), 8.29 (d, *J* = 4.8 Hz, 1 H), 7.31-7.21 (m, 3 H), 7.12 (d, *J* = 7.6 Hz, 2 H), 7.07 (d, *J* = 4.8 Hz, 1 H), 5.94 (br, 1 H), 2.95-2.85 (m, 4 H), 1.49 (s, 9 H).

^{13}C-NMR (101 MHz, $CDCl_3$)
δ 153.3, 145.9, 145.5, 141.9, 140.6, 132.9, 128.8, 128.5, 126.7, 124.0, 81.0, 35.5, 32.9, 28.4.

IR Alpha-Platinum ATR, Bruker, diamond crystal
υ = 3179, 2971, 2931, 1705, 1600, 1566, 1530 cm^{-1}

HRMS ESI
Calculated mass for $(M+H)^+$ of $C_{18}H_{22}N_2O_2$ is 299.1754 found 299.1753.

M.P. 127.2-129.1 °C

3-(2-(pyridin-4-yl)ethyl)pyridine (**27**)

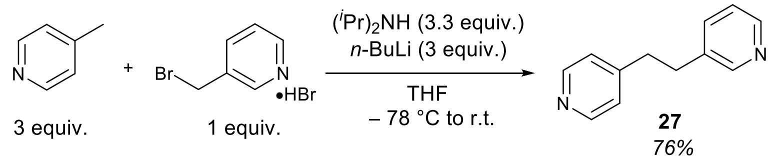

Substrate **27** was synthesized according to procedure 1 using 4-picoline (0.78 g, 8.4 mmol, 3.0 equiv.) and 3-(bromomethyl)pyridine•HBr salt as the electrophile (0.70 g, 2.8 mmol, 1.0 equiv.). The product was purified by flash chromatography and was obtained as an orange oil (0.39 g, 2.1 mmol) in 76% yield.

Chromatography: 5% Et_3N : 25% Hexane : 70% EtOAc (R_f = 0.25).

^{1}H-NMR (400 MHz, $CDCl_3$)
δ 8.48 (d, *J* = 5.6 Hz, 2 H), 8.45 (d, *J* = 4.8 Hz, 1 H), 8.42 (s, 1 H), 7.42 (d, *J* = 7.6 Hz, 1 H), 7.19 (dd, *J* = 7.6, 4.8 Hz, 1 H), 7.06 (d, *J* = 5.6 Hz, 2 H), 2.93 (br, 4 H).

^{13}C-NMR (101 MHz, $CDCl_3$)
δ 149.8, 149.8, 149.5, 147.7, 135.8, 135.7, 123.8, 123.2, 36.5, 33.5.

IR Alpha-Platinum ATR, Bruker, diamond crystal
υ = 3026, 2928, 1599, 1575, 1558 cm^{-1}

HRMS ESI
Calculated mass for $(M+H)^+$ of $C_{12}H_{12}N_2$ is 185.1073 found 185.1072.

2-(4-(2-(Pyridin-4-yl)ethyl)phenethyl)pyridine (**28**)

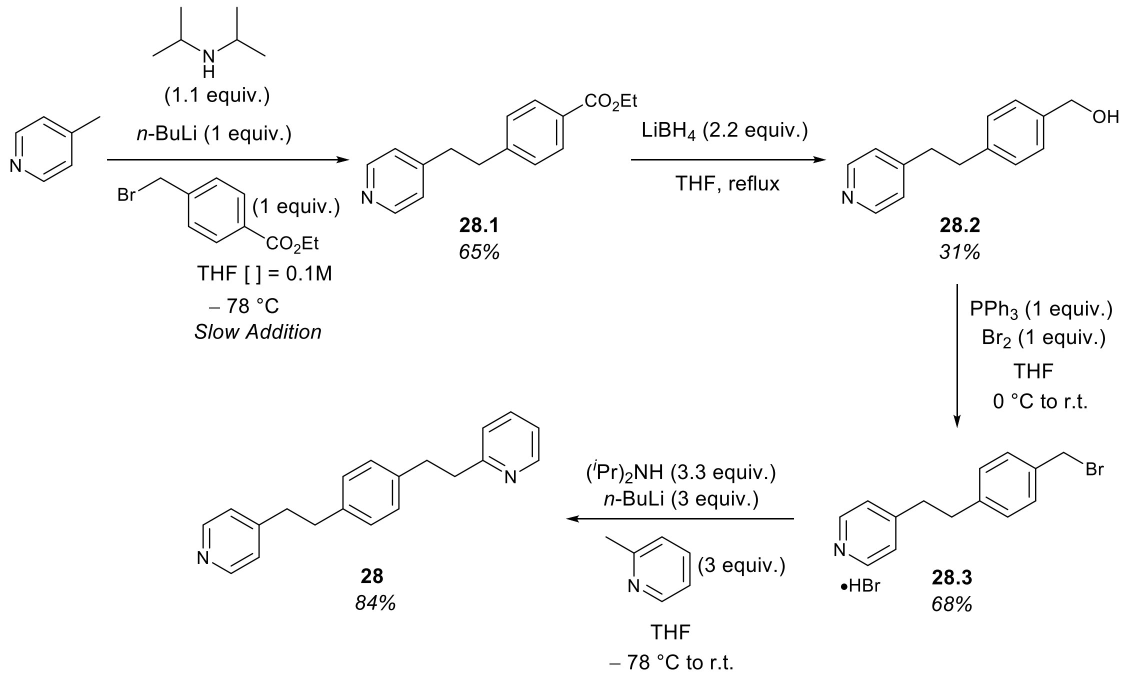

*Synthesis of **28.1*** (CAS: 1802629-62-4) – **28.1** was synthesized according to procedure 1 using 4-picoline (0.80 g, 8.6 mmol, 1.0 equiv.) and ethyl-4-bromomethylbenzoate as the electrophile (2.08 g, 8.60 mmol, 1.0 equiv.). The crude mass was purified by flash chromatography to afford **28.1** as a white solid (1.43 g, 5.58 mmol) in 65% yield. Spectral data is consistent with that reported in the literature.[21]

Chromatography: 80% EtOAc in hexanes (R_f. = 0.46).

Data for **28.1**

^{1}H-NMR (400 MHz, $CDCl_3$)
δ 8.48 (dd, *J* = 4.8, 1.6 Hz, 2 H), 7.95 (d, *J* = 8.4 Hz, 2 H), 7.20 (d, *J* = 8.4 Hz, 2 H), 7.05 (d, *J* = 6.4 Hz, 2 H), 4.36 (q, *J* = 7.2 Hz, 2 H), 3.01-2.91 (m, 4 H), 1.39 (t, *J* =7.2 Hz, 3 H).

^{13}C-NMR (101 MHz, $CDCl_3$)
δ 166.6, 150.0, 149.9, 146.0, 129.9, 128.8, 128.6, 124.0, 61.0, 36.7, 36.6, 14.5.

2-(4-(2-(Pyridin-4-yl)ethyl)phenethyl)pyridine (**28**)

*Reduction of **28.1*** – A flame-dried round-bottomed flask equipped with a stir bar was charged with a solution of ester **28.1** (1.43 g, 5.58 mmol, 1.0 equiv.) in dry THF (50 mL). $LiBH_4$ (0.15 g, 6.70 mmol, 1.20 equiv.) was then added and the reaction was heated to reflux and left to stir overnight. A second portion of $LiBH_4$ (0.15 g, 6.70 mmol, 1.2 equiv.) was added, and after 2 h the reaction was quenched with a saturated solution of $NaHCO_3$ and extracted with EtOAc three times. The combined organics were washed with brine, dried using $MgSO_4$ and concentrated *in vacuo*. The crude mixture was purified by flash chromatography to afford **28.2** as a white solid (0.36 g, 1.7 mmol) in 31% yield.

Chromatography: 100% EtOAc (R_f. = 0.34).

Data for **28.2**

^{1}H-NMR (400 MHz, $CDCl_3$)
δ 8.46 (dd, J = 4.8, 1.6 Hz, 2 H), 7.29 (d, J = 8.0 Hz, 2 H), 7.14 (d, J = 8.0 Hz, 2 H), 7.05 (dd, J = 4.8, 1.6 Hz, 2 H), 4.67 (s, 2 H), 2.94-2.91 (m, 4 H), 1.97 (br, 1 H).

^{13}C-NMR (101 MHz, $CDCl_3$)
δ 150.6, 149.8, 140.2, 139.1, 128.8, 127.4, 124.1, 65.2, 37.2, 36.4.

IR Alpha-Platinum ATR, Bruker, diamond crystal
υ = 3158, 2879, 1603, 1558 cm^{-1}

HRMS ESI
Calculated mass for $(M+H)^+$ of $C_{14}H_{15}NO$ is 214.1226 found 214.1216.

M.P. 175.7-177.1 °C

2-(4-(2-(Pyridin-4-yl)ethyl)phenethyl)pyridine (**28**)

Bromination of ***28.2*** – A flame-dried round-bottomed flask equipped with a stir bar was charged with triphenylphosphine (0.32 g, 1.2 mmol, 1.0 equiv.) and dry THF (5 mL), and cooled to 0 °C. Bromine (0.06 mL, 1.2 mmol, 1.0 equiv.) was then added dropwise and the mixture was left to stir for 20 min. A solution of alcohol **28.2** (0.26 g, 1.2 mmol, 1.0 equiv.) in THF (7 mL) was introduced and the reaction mixture was allowed to warm to room temperature overnight. The pyridinium salt (**28.3**) precipitated out of the mixture and was isolated as a white solid after vacuum filtration (0.29 g, 0.83 mmol) in 68% yield. This salt was used in the next step without further purification.

Data for **28.3**

^{1}H-NMR (400 MHz, $CDCl_3$)
δ 8.64 (d, J = 6.8 Hz, 2 H), 7.62 (d, J = 6.8 Hz, 2 H), 7.33 (d, J = 8.0 Hz, 2 H), 7.06 (d, J = 8.0 Hz, 2 H), 4.67 (s, 2 H), 3.23 (t, J = 7.4 Hz, 2 H), 3.06 (t, J = 7.4 Hz, 2 H).

^{13}C-NMR (101 MHz, $CDCl_3$)
δ 162.8, 140.2, 138.6, 137.0, 129.8, 128.9, 127.1, 37.9, 35.6, 33.0.

IR Alpha-Platinum ATR, Bruker, diamond crystal
υ = 3370, 3091, 3052, 1634, 1594 cm^{-1}

HRMS ESI
Calculated mass for $(M+H)^+$ of $C_{14}H_{15}Br_2N$ is 355.9644 found $(C_{14}H_{15}BrN+H-HBr)^+$ 276.0400.

2-(4-(2-(Pyridin-4-yl)ethyl)phenethyl)pyridine (**28**)

*Alkylation of **28.3** using 2-methylpyridine* – **28** was synthesized according to procedure 1 using 2-picoline (0.23 g, 2.5 mmol, 3.0 equiv.) and **28.3** (0.30 g, 0.82 mmol, 1.0 equiv.) as the electrophile. The product was purified using flash chromatography and obtained as a white solid (0.20 g, 0.69 mmol) in 84% yield.

Chromatography: 3% MeOH in EtOAc ($R_{f.}$ = 0.44).

Data for **28**

^{1}H-NMR (400 MHz, $CDCl_3$)
δ 8.56 (d, J = 4.4 Hz, 1 H), 8.48 (dd, J = 4.4, 1.6 Hz, 2 H), 7.56 (td, J = 7.6, 1.6 Hz, 1 H), 7.13-7.04 (m, 8 H), 3.10-3.01 (m, 4 H), 2.89 (br, 4 H).

13C-NMR (101 MHz, $CDCl_3$)
δ 161.4, 150.7, 149.8, 149.5, 139.6, 138.4, 136.4, 128.7, 128.5, 124.1, 123.1, 121.3, 40.4, 37.2, 36.2, 35.7.

IR Alpha-Platinum ATR, Bruker, diamond crystal
υ = 2922, 2856, 1587, 1566 cm^{-1}

HRMS ESI
Calculated mass for $(M+H)^+$ of $C_{20}H_{20}N_2$ is 289.1699 found 289.1688.

M.P. 91.5-92.6 °C

2-(2-(Pyridin-4-yl)ethyl)pyridine (29)

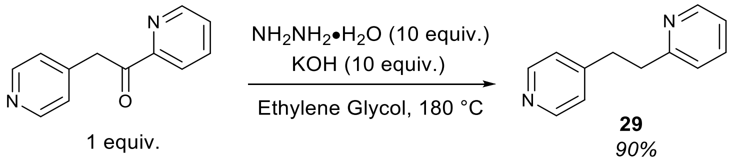

A round-bottomed flask equipped with a stir bar and a condenser was charged with a solution of 1-(pyridin-2-yl)-2-(pyridin-4-yl)ethenone (0.43 g, 2.16 mmol, 1 equiv.) in 10 mL ethylene glycol. Hydrazine monohydrate (1.1 mL, 21.6 mmol, 1 equiv.) was introduced at room temperature, the resulting solution was heated to 180 °C for 1 hr., and then cooled to ~80 °C. Potassium hydroxide pellets (1.21 g, 21.6 mmol, 10 equiv.) were then carefully added in small portions and the reaction mixture was again heated to 180 °C for 1 hr. Upon completion of the reaction, as indicated by TLC, the mixture was allowed to cool to ambient temperature and diluted with Et_2O. The phases were separated and the organic phase was washed with brine three times, dried over $MgSO_4$, filtered and concentrated *in vacuo* to afford **29** as an orange oil (0.36 g, 1.94 mmol) in 90% yield which was used without further purification.

Data for **29**

^{1}H-NMR (400 MHz, $CDCl_3$)
δ 8.51 (d, *J* = 4.4 Hz, 1 H), 8.47 (d, *J* = 5.6 Hz, 2 H), 7.52 (t, *J* = 7.6 Hz, 1 H), 7.09-7.00 (m, 4 H), 3.08-2.99 (m, 4 H).

^{13}C-NMR (101 MHz, $CDCl_3$)
δ 160.0, 150.3, 149.5, 149.3, 136.3, 123.8, 122.9, 121.3, 38.6, 34.8.

IR Alpha-Platinum ATR, Bruker, diamond crystal
υ = 3024, 2928, 1590, 1568 cm^{-1}

HRMS ESI
Calculated mass for $(M+H)^+$ of $C_{12}H_{12}N_2$ is 185.1073 found 185.1071.

4-Isopropylpyridine (**31**, CAS 696-30-0)

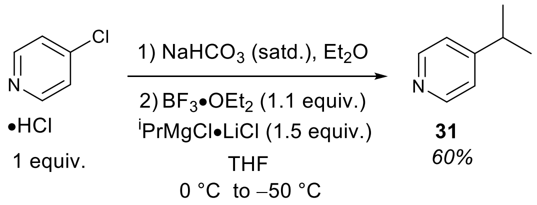

4-Chloropyridine hydrochloride (2 g, 13.3 mmol, 1 equiv.) was dissolved in saturated sodium bicarbonate (30 mL) solution to release the free pyridine. The aqueous solution was extracted with Et_2O three times and the combined organics were washed with brine, dried over $MgSO_4$ and concentrated *in vacuo* at 0 °C. The resulting oil was then dissolved in anhydrous THF (24 mL) and transferred to a flame-dried round-bottomed flask equipped with a stir bar. The solution was cooled to 0 °C, and BF_3•OEt_2 was added drop-wise. After stirring for 15 min, the flask was cooled to –50 °C and a solution of isopropylmagnesium chloride•lithium chloride complex (12.7 mL, 16.5 mmol, 1.2 equiv.) was added dropwise. The mixture was stirred for 30 min., and then quenched with NH_4OH (12 mL). The aqueous layer was extracted with Et_2O three times and the combined organics were washed with brine, dried over $MgSO_4$ and concentrated *in vacuo* at 0 °C. The crude mixture was purified using flash chromatography to afford pyridine **31** (0.97 g, 8.04 mmol) as a yellow oil in 60% yield. Spectral data is consistent with that reported in the literature.[22]

Chromatography: 40% Et_2O in Pentane (R_f. = 0.32).

Data for **31**

^{1}H-NMR (400 MHz, $CDCl_3$)
δ 8.50 (d, J = 6.1 Hz, 2 H), 7.14 (d, J = 6.1 Hz, 2 H), 2.88 (sept, J = 7 Hz, 1 H), 1.26 (d, J = 7 Hz, 6 H).

^{13}C-NMR (76 MHz, $CDCl_3$)
δ 157.6, 149.9, 122.1, 33.7, 23.2.

***cis*-5-Phenyl-2-cyclohexen-1-ol** (**32**, CAS 26114-87-4)

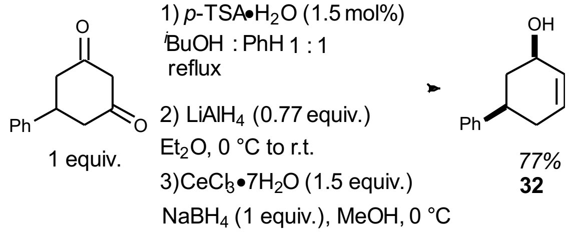

A round-bottomed flask equipped with a stir bar was charged with 5-phenyl-1,3-cyclohexanedione (200 mg, 1.1 mmol, 1.00 equiv.), *p*-toluic acid (2 mg, 0.016 mmol, 0.015 equiv.) and a benzene : isobutylalcohol mixture (1 : 1, 4.66 mL). The flask was fitted with a Dean-Stark trap and heated to reflux for 3 h. The solution was then cooled to room temperature and concentrated under reduced pressure to afford a viscous oil which was dissolved in diethyl ether (8 mL) and washed with 2M NaOH three times. The organic layer was washed with brine, dried over $MgSO_4$ and concentrated under reduced pressure. The resulting oil was dissolved in anhydrous diethyl ether (12 mL) and transferred to a flame-dried round-bottomed flask. The solution was cooled to 0°C, $LiAlH_4$ (31 mg, 0.82 mmol, 0.77 equiv.) was added portion-wise and the resulting mixture was stirred at room temperature for 2 h. The reaction was quenched with 10% aq. HCl, extracted with diethyl ether, washed with brine, dried over $MgSO_4$ and concentrated under reduced pressure. The resulting oil was then dissolved in methanol (16 mL), $CeCl_3$•$7H_2O$ (394 mg, 1.60 mmol, 1.5 equiv.) was added, and then $NaBH_4$ was added portion-wise (40 mg, 1.06 mmol, 1 equiv.). This reaction mixture was stirred for 3 h at 0°C, then concentrated under reduced pressure and washed with 1 M HCl (3x) and brine. It was then dried over $MgSO_4$ and concentrated *in vacuo*. Purification by chromatography furnished *cis*-5-phenyl-2-cyclohexen-1-ol (142 mg, 0.85 mmol) as a white solid in 77% yield. Spectral data is consistent with that reported in the literature.[23]

Chromatography: 20% EtOAc in Hexanes (R_f. = 0.40).

Data for **32**

^{1}H-NMR (300 MHz, $CDCl_3$)

δ 7.33-7.29 (m, 2 H), 7.23-7.18 (m, 3 H), 5.84-5.81 (m, 1 H), 5.77-5.73 (m, 1 H), 4.47 (br, 1 H), 2.96-2.83 (m, 1 H), 2.37-2.21 (m, 2 H), 2.19-2.04 (m, 1 H), 1.78-1.68 (m, 1 H), 1.58 (d, J = 5.1Hz, 1 H).

^{13}C-NMR (101 MHz, $CDCl_3$)

δ 145.6, 131.1, 128.7, 128.6, 126.8, 126.4, 68.6, 39.6, 39.3, 33.8.

***cis*-5-Phenyl-2-cyclohexen-1-ol** (**32**, CAS 26114-87-4)

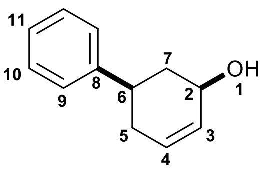

Proton No.	^{1}H δ (ppm) (mult: *J* (Hz))[a, b,c,d]	COSY Correlation
H-1	1.58 (d, *J* = 5.1Hz, 1H)	H-2, H-7a, H-7b
H-2	4.47 (s, 1H)	H-1, H-5a H-7a, H-7b
H-3	5.77-5.73 (m, 1H)	H-4
H-4	5.84-5.81 (m, 1H)	H-4, H-5b, H-7b
H-5	H-5a: 2.19-2.04 (m, 1H)<br>H-5b: part of the m at 2.37-2.21	H-2, H-4, H-6, H-7a, H-7b
H-6	2.96-2.83 (m, 1H)	H-5a, H-5b, H-7a, H-7b
H-7	H-7a: 1.78-1.68 (m, 1H)<br>H-7b: part of the m at 2.37-2.21	H-1, H-2, H-4, H-5a, H-5b, H-6
H-9	Part of the m at 7.23-7.18	H-10, H-11
H-10	7.33-7.29 (m, 2H)	H-9, H-11
H-11	Part of the m at 7.23-7.18	H-9, H-19

[a] Recorded at 600 MHz. [b] Assignments based on HSQC-DEPT and HMBC data

[c] Methylene protons are designated H-Xa and H-Xb arbitrarily

[d] Only those correlations which could be unambiguously assigned are reported.

***cis*-5-Phenyl-2-cyclohexen-1-ol** (**32**, CAS 26114-87-4)

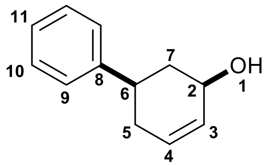

Proton No.	^{1}H δ (ppm) (mult: *J* (Hz))[a, b,c,d]	NOESY Correlation
H-1	1.58 (d, *J* = 5.1Hz, 1H)	H-2
H-2	4.47 (s, 1H)	H-1, H-6, H-7a, H-7b
H-3	5.77-5.73 (m, 1H)	H-4, H-7a
H-4	5.84-5.81 (m, 1H)	H-3, H-5a, H-5b, H-7b
H-5	H-5a: 2.19-2.04 (m, 1H)<br>H-5b: part of the m at 2.37-2.21	H-2, H-4, H-6, H-7a, H-7b, H-9
H-6	2.96-2.83 (m, 1H)	H-2, H-5a, H-5b, H-7a, H-7b, H-9
H-7	H-7a: 1.78-1.68 (m, 1H)<br>H-7b: part of the m at 2.37-2.21	H-2, H-3, H-4, H-5a, H-5b, H-6, H-9
H-9	Part of the m at 7.23-7.18	H-5a, H-5b, H-7a, H-7b, H-10, H-11
H-10	7.33-7.29 (m, 2H)	H-9, H-11
H-11	Part of the m at 7.23-7.18	H-9, H-10

[a] Recorded at 600 MHz. [b] Assignments based on HSQC-DEPT and HMBC data

[c] Methylene protons are designated H-Xa and H-Xb arbitrarily

[d] Only those correlations which could be unambiguously assigned are reported.

[(Xantphos)Pd(η3-allyl)]OTf (33)

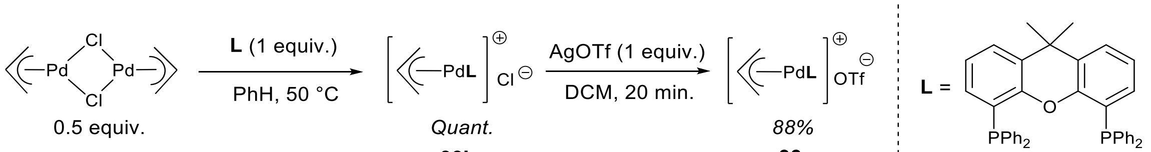

33' 33

A flame-dried round-bottomed flask equipped with a stir bar was charged with [Pd(η^3-allyl)Cl]$_2$ (100 mg, 0.27 mmol, 0.5 equiv.) and benzene (7.5 mL). Xantphos (316 mg, 0.55 mmol, 1 equiv.) was suspended in benzene (7.5 mL) in a second round-bottomed flask. Both flasks were heated to 50 °C until the solids dissolved, then the solution of palladium in benzene was transferred via cannula to the flask containing xantphos. After stirring for 30 min at 50 °C, the resulting suspension was allowed to cool to ambient temperature, and the precipitate was isolated by filtration in air. The precipitate was washed two times with 15 mL of Et_2O and dried *in vacuo* to give the [(Xantphos)Pd(allyl)Cl] (419 mg, 0.55 mmol) as an orange solid in quantitative yield. 0.3 g of the complex was then taken inside a glovebox, dissolved in DCM (24 mL) and transferred to a 50 mL vial equipped with a stir bar. To it was added AgOTf (101 mg, 0.39 mmol, 1 equiv.) and the mixture was stirred for 20 min. The suspension was then filtered through Celite® and concentrated *in vacuo.* The crude product was recrystallized by dissolving the crude complex in a minimum amount of DCM, then layering it with Et_2O and cooling to –35 °C overnight. The resulting pure complex was isolated by filtration, and was washed with Et_2O twice. After drying under reduced pressure, complex **33** was obtained as a yellow solid (303 mg, 0.35 mmol) in 88% yield. Spectral data is consistent with that reported in the literature.[24]

Data for **33**

^{1}H-NMR (300 MHz, CD_2Cl_2)
δ 7.67 (dd, J = 7.7, 1.2 Hz, 2 H), 7.45-7.33 (m, 12 H), 7.20-7.11 (m, 10 H), 6.58 (t, J = 7.8 Hz, 2 H), 6.58 (sept, J = 7.2 Hz, 1 H), 3.75 (d, J = 6.9 Hz, 2 H), 3.42 (m, 2 H), 1.65 (br, 3 H), 1.54 (s, 3 H).

^{13}C-NMR (101 MHz, CD_2Cl_2)
δ 155.6 (t, J = 3.5 Hz), 134.8, 133.3 (t, J = 6 Hz), 132.9 (t, J = 6 Hz), 132.7, 132.5, 131.4 (d, J = 6 Hz), 129.8 (m), 129.5 (t, J = 6 Hz), 129.0, 125.4 (t, J = 4 Hz), 123.1, 122.5 (t, J = 5.1 Hz), 118.3, 117.9 (m), 79.9 (t, J = 15.2 Hz), 36.6, 30.2, 25.8.

^{31}P-NMR (122 MHz, CD_2Cl_2)
δ 4.27.

Methallyl alcohol-*d3* (34)

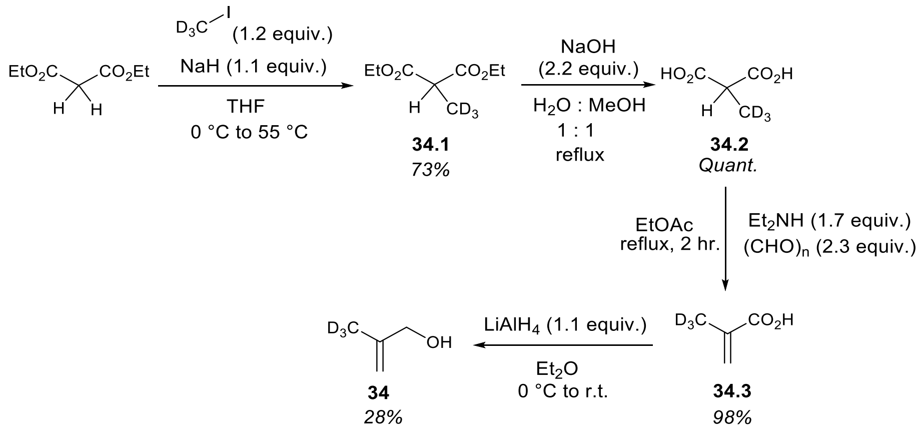

<u>*Alkylation of diethylmalonate* (**34.1**, CAS: 54840-57-2)</u> – An oven-dried 50 mL vial equipped with a stir bar was brought inside a glove box and charged with dry NaH (380 mg, 15.8 mmol, 1.1 equiv.). It was then brought out, kept under an atmosphere of argon and anhydrous THF (24 mL) was introduced to form a suspension of NaH. The vial was cooled to 0 °C and then a solution of diethylmalonate (2.3 g, 14.4 mmol, 1 equiv.) in THF (24 mL) was added slowly. The clear solution was stirred at ambient temperature for 1 h, and then cooled again to 0 °C. Methyl iodide-d3 was then added dropwise and the mixture was allowed to warm up to ambient temperature. The solution was stirred for two hours at room temperature and heated to 55 °C overnight. The solution was cooled to room temperature then quenched with saturated aqueous ammonium chloride. The aqueous layer was extracted with EtOAc three times and the combined organics were dried with $MgSO_4$, concentrated *in vacuo* and purified using flash chromatography to deliver the product as a colorless oil (1.86 g, 10.5 mmol) in 73% yield. Spectral data is consistent with that reported in the literature.[25]

Chromatography: 100% hexanes – 4% EtOAc in hexanes (R_f. = 0.3 in 10% EtOAc in hexanes).

Data for **34.1**

<u>^{1}H-NMR</u> (400 MHz, $CDCl_3$)

δ 4.20 (m, 4 H), 3.4 (s, 1H), 1.2 (t, *J* = Hz, 6 H).

<u>^{13}C-NMR</u> (76 MHz, $CDCl_3$)

δ 170.2, 61.3, 46.0, 14.0.

Methallyl alcohol-*d3* (34)

Ester Hydrolysis (**34.2**, CAS: 42522-59-8) – Sodium hydroxide pellets (0.94 g, 23.56 mmol, 2.2 equiv.) were added to a solution of *d3*-methyl diethylmalonate (1.89 g, 10.71 mmol, 1 equiv.) in a 1:1 MeOH:H_2O (30 mL), and the resulting mixture was refluxed for 4 hours. After being cooled to ambient temperature, the mixture was diluted with distilled water and the aqueous layer was extracted once with EtOAc. The aqueous layer was then acidified to pH = 1 using 1M HCl, and then extracted thrice with EtOAc. The combined organics were dried with $MgSO_4$ then concentrated *in vacuo* to provide the diacid **34.2** as a white solid (1.27 g, 10.71 mmol) in quantitative yield which was used in the next step without further purification.

Formation of the α,β-Unsaturated Acid (**34.3**)[26] – A solution of the crude *d3*-methyl malonic acid (1.27 g, 10.71 mmol, 1 equiv.) in EtOAc (43 mL) was cooled to 0 °C. Diethylamine (1.85 mL, 17.88 mmol, 1.7 equiv.) was added dropwise to form a white suspension. Paraformaldehyde (0.72 g, 23.87 mmol, 2.25 equiv.) was added and the mixture was refluxed for two hours, then cooled to 0 °C and quenched with 1M HCl. The aqueous layer was extracted thrice with Et_2O and the combined organics were washed with brine, dried with $MgSO_4$ then concentrated *in vacuo* to provide the α,β-unsaturated acid as a yellow oil (0.873 g, 9.78 mmol) in 93% yield. This material was used in the next step without further purification.

$LiAlH_4$ Reduction – A flame-dried round-bottomed flask equipped with a stir bar was charged with the α,β-unsaturated acid (0.937 g, 9.87 mmol, 1 equiv.) in diethyl ether (15 mL) and cooled to 0 °C. A solution of Lithium Aluminum Hydride (1.37 g, 10.86 mmol, 1.1 equiv.) in diethyl ether (15 mL) was added dropwise and the mixture was allowed to stir for 4 hours at room temperature. Once complete, the mixture was cooled to 0 °C and quenched with distilled H_2O (1.5 mL) followed by 1M $NaOH_{(aq.)}$ (1.5 mL) and distilled H_2O (4.5 mL). The mixture was then transferred to a separatory funnel and Rochelle's salt was added. The aqueous layer was extracted thrice with Et_2O and the combined organics were given a brine wash, dried with $MgSO_4$ then concentrated by distillation under atmospheric pressure using a Vigreux column. The crude product was purified using flash chromatography and the pure product obtained after atmospheric distillation as a 2.5 M solution in diethyl ether (2.76 mmol) in 28% yield.

Chromatography: 40% Et_2O in Pentane (R_f. = 0.42).

Data for **34**

^{1}H-NMR (300 MHz, $CDCl_3$)

δ 4.98 (q, *J* = 1.5 Hz, 1 H), 4.87 (s, 1 H), 4.05 (dt, *J* = 6.3, 1.2 Hz, 2 H).

^{2}H-NMR (46 MHz, $CDCl_3$)

δ 1.73.

^{13}C-NMR (151 MHz, $CDCl_3$)

δ 144.5, 110.0, 67.0, 18.3 (m).

HRMS DART

Calculated mass for $(M+H)^+$ of $C_4H_6D_3O$ is 75.0764,found $(C_4H_6D_3O–H)^+$ 75.0778.

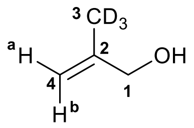

Carbon No.	^{13}C δ (ppm)	^{1}H δ (ppm) (mult; *J* (Hz)) [a, b]	HMBC Correlations
1	67.0	H-1 : 4.05 (dt, *J* = 6.3, 1.2 Hz, 2 H)	H-4a, H-4b
2	144.5	--	H-1
3	18.3 (m)	--	H-1, H-4a
4	110.0	4.98 (q, *J* = 1.5 Hz, 1 H), 4.87 (s, 1 H)	H-1

[a] Recorded at 600 MHz.

[b] Only the correlations that could be unambiguously assigned are reported.

***trans*-1,2-Bis[2-(diphenylphosphino)benzamido]cyclohexane** (**L1**)

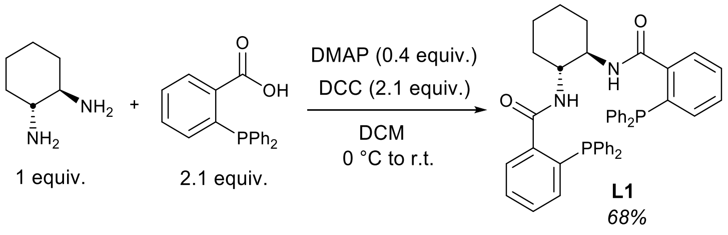

A flame-dried round-bottomed flask equipped with a stir bar was charged with 2-(diphenylphosphino)benzoic acid (307 mg, 1.0 mmol, 2.1 equiv.) and dry DCM (9.5 mL). *trans*-1,2-Bis(methylamino)cyclohexane (55 mg, 0.50 mmol, 1 equiv.) was then introduced and the solution was cooled to 0 °C. DMAP (23 mg, 0.2 mmol, 0.4 equiv.) and DCC (208 mg, 1 mmol, 2.1 equiv.) were then added sequentially and the mixture was stirred at room temperature overnight. The solution was then diluted with DCM and filtered through a pad of Celite® and concentrated in vacuo. The residue was dissolved in Et_2O, filtered through a pad of Celite®, concentrated in vacuo then purified using flash chromatography to afford a white solid (224 mg, 0.34 mmol) in 68% yield. Spectral data is consistent with that reported in the literature.[27]

Chromatography: 40% EtOAc in Hexanes (R_f. = 0.40).

Data for **L1**

^{1}H-NMR (300 MHz, $CDCl_3$)

δ 7.60 (m, 2 H), 7.35-7.20 (m, 24 H), 6.93 (m, 2 H), 6.33 (d, *J* = Hz, 2 H), 3.79 (m, 2 H), 1.88 (d, *J* = 12 Hz, 2 H), 1.66 (d, *J* = 8 Hz, 2 H), 1.23 (m, 2 H), 0.99 (m, 2 H).

^{13}C-NMR (76 MHz, $CDCl_3$)

δ 169.4, 141.0 (d, *J* = 24.3 Hz), 137.8 (dd, *J* = 5.3, 11.4 Hz), 136.7 (d, *J* = 22.0 Hz), 134.4, 134.1, 133.9, 130.3, 128.9, 128.7-128.5 (m), 127.7 (d, *J* = 3.8 Hz), 54.0, 32.1, 24.8.

***trans*-1,2-Bis[2-(diphenylphosphino)-*N*-methyl-benzamido]cyclohexane (L2)**

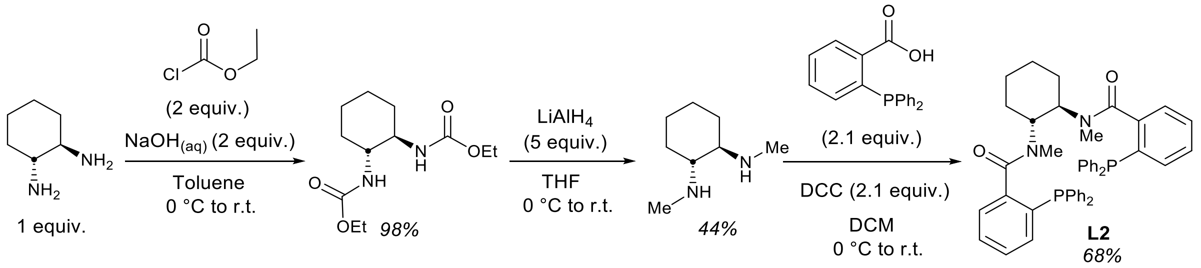

<u>Carbamate Formation</u> – A solution of *trans*-1,2-diaminocyclohexane (0.61 mL, 5 mmol, 1 equiv.) in toluene (10 mL) was cooled to 0 °C and to it was added ethyl chloroformate dropwise (0.96 mL, 10 mmol, 2 equiv.) followed by 1M $NaOH_{(aq)}$ (10 mL, 10 mmol, 2 equiv.). The mixture was allowed to warm to room temperature while stirring for 3 hours and was then filtered through a cotton plug. The mixture was extracted with EtOAc three times and the combined organics were washed with brine, dried with MgSO4, and concentrated *in vacuo* to afford a white solid (1.267 g, 4.9 mmol) in 98% yield. The crude product was used in the next step without further purification.

<u>Carbamate Reduction</u> – A flame-dried round-bottomed flask equipped with a stir bar was charged with Lithium Aluminum Hydride (949mg, 25 mmol, 5 equiv.) and cooled to 0 °C and to it was added a solution of the crude 1,2-bis(ethoxycarbonylamino)cyclohexane (1.267 g, 5 mmol, 1 equiv.) in dry THF (10 mL). The mixture was stirred under argon for at least 2 hours and monitored by TLC. Once complete, the mixture was quenched with distilled H_2O (1 mL) followed by 15 % $NaOH_{(aq.)}$ (1 mL). The mixture was stirred for 15 min then $MgSO_4$ was added to the mixture and stirred for additional 15 min. The mixture was filtered through a pad of Celite® then HCl in dioxane (1.5 mL, 4M) was added to the filtrate while stirring. The mixture was extracted with distilled H_2O and the combined aqueous layer was basified using NaOH pellet then extracted with DCM three times. The combined organics were washed with brine, dried with $MgSO_4$, and concentrated *in vacuo* to provide a colorless oil (312 mg, 2.19 mmol) in 44% yield which was used in the next step without further purification.

***trans*-1,2-Bis[2-(diphenylphosphino)-*N*-methyl-benzamido]cyclohexane** (**L2**)

<u>*DCC Coupling*</u> – A flame-dried round-bottomed flask equipped with a stir bar was charged with *trans*-1,2-bis(methylamino)cyclohexane (312 mg, 2.19 mmol, 1 equiv.), 2-(diphenylphosphino) benzoic acid (1.357 g, 4.43 mmol, 2.1 equiv.), DCC (914 mg, 4.43 mmol, 2.1 equiv.), and few crystal of DMAP in DCM (4 mL). The mixture was stirred at room temperature overnight and then concentrated *in vacuo*. The resulting crude was purified by flash chromatography to afford **L2** as a white powder (1.02 g, 1.49 mmol) in 68% yield.[28]

Chromatography: 20% EtOAc in hexanes (R_f. = 0.3).

Data for **L2**

<u>^{1}H-NMR</u> (400 MHz, $CDCl_3$)
δ 7.37-7.20 (m, 26 H), 7.16-7.10 (m, 2 H), 4.95-4.85 (m, 2 H), 2.79 (s, 6 H), 1.94-1.86 (m, 2 H), 1.85-1.77 (m, 2 H), 1.63-1.50 (m, 2 H), 1.50-1.37(m, 2 H).

<u>^{13}C-NMR</u> (101 MHz, $CDCl_3$)
δ 171.1, 171.0, 143.8, 143.5, 136.8 (d, *J* = 10.1 Hz), 136.4 (, *J* = 11.1 Hz), 134.4 (m), 134.0, 133.8, 133.4, 133.2, 129.1, 128.8, 128.7, 128.49, 128.46 (m), 126.3, 52.0, 31.6, 28.9, 25.0.

Reaction Development.

Part A: Procedure for Alkylidene Dihydropyridine Formation

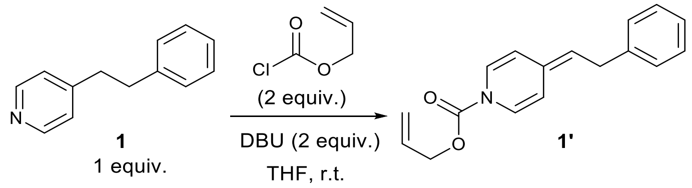

4-Phenethylpyridine **1** (0.10 g, 0.59 mmol, 1.0 equiv.) and diazabicyclo[5.4.0]undec-7-ene (0.17 g, 1.09 mmol, 2.0 equiv.) were dissolved in dry THF (6 mL) in a flame-dried round-bottomed flask equipped with a stir bar. Allyl chloroformate (0.13 g, 1.09 mmol, 1.0 equiv.) was added dropwise and the solution was left to stir for 30 min. The reaction mixture was then diluted with EtOAc and washed with pH 5.5 phosphate buffer thrice. The organic phase was then washed with brine, dried over $MgSO_4$ and concentrated *in vacuo* to afford the corresponding alkylidene dihydropyridine (ADHP) intermediate **1'** as a bright yellow oil.

Data for **1'**

^{1}H-NMR (300 MHz, $CDCl_3$, 45°C)
δ 7.28-7.16 (m, 5 H), 6.96 (d, J = 5.7 Hz, 1 H), 6.81 (d, J = 6.3 Hz, 1 H), 6.01-5.88 (m, 2 H), 5.68 (d, J = 7.2 Hz, 1 H), 5.35 (d, J = 17.4 Hz, 1 H), 5.28 (d, J = 10.2 Hz, 1 H), 4.97 (t, J = 7.8 Hz, 1 H), 4.71 (d, J = 5.7 Hz, 2 H), 3.38 (d, J = 7.8 Hz, 2 H).

^{13}C-NMR (76 MHz, $CDCl_3$, 52°C)
δ 150.4, 141.2, 131.6, 128.5, 128.3, 128.2, 125.8, 124.6, 122.4, 118.7, 115.6, 114.4, 109.0, 67.3, 33.1.

IR Alpha-Platinum ATR, Bruker, diamond crystal
υ = 3026, 1719, 1673, 1300, 1285, 1192, 1127, 1102, 966, 937 cm^{-1}

HRMS ESI
Calculated mass for $(M+H)^+$ of $C_{17}H_{17}NO_2$ is 268.1332, found 268.1328.

Part B: Ligand Screen

To expedite the ligand-screening process, pyridine **1** was subjected to alkylidene dihydropyridine formation conditions described in part A of the reaction development section (Pg. 49). The resulting ADHP **1′** was weighed and split into equal portions by mass; each portion was subjected to $Pd(dba)_2$ (5 mol%) and a phosphine ligand (1:2 Pd:P) in THF at room temperature under an atmosphere of argon. Each reaction mixture was left to stir overnight then concentrated *in vacuo*. The yields (reported over two steps) of allylation (**1A**) and elimination (**1B**) products were determined by quantitative ^{1}H-NMR using 1,4-bis(trichloromethyl)benzene as an internal standard. Triphenylphosphine (entry 2) and bidentate ligands with large bite angles (entries 12-14) provided the allylation product **1A** in good yields. Since complexes with bidentate ligands should display greater stability than those with monodentate ligands, Xantphos was our ligand of choice in future reaction development.

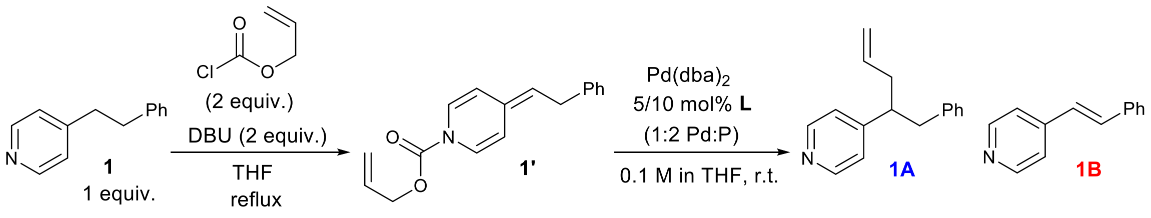

1) no ligand 0%, 25%

2) PPh_3 73%, 0%

3) P(*o*-tol)$_3$ 23%, 0%

4) P(2-furyl)$_3$ 48%, 17%

5) P(t-Bu)$_3$ 24%, 13%

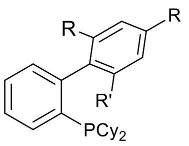

6) R & R' = *i-Pr*: 71%, 0%

7) R = H, R' = NMe_2: 48%, 0%

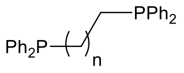

8) n = 1: 31%, 15%

9) n = 2: 22%, 41%

10) n = 3: 42%, 0%

11) n = 4: 63%, 0%

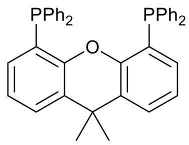

12) XantPhos: 75%, 0%

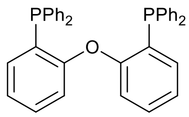

13) DPEPhos: 73%, 0%

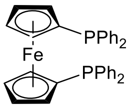

14) dppf: 68%, 0%

Since the yields obtained in the ligand screen were based off quantitative ^{1}H NMR, the allylation of **1** was repeated on a 200 mg scale and using a 5 mol% of Pd-Xantphos catalytic system to investigate the isolated yield of the reaction. The resulting product **1A** was purified using flash chromatography and isolated in 78% yield. We hypothesized that the yield could be further improved by eliminating the work-up steps after both the ADHP formation and allylation reactions. Therefore, we implemented the following adjustments that provided the allylation product **1A** in 92% isolated yield using 1 mol% catalyst loading:

1) 3 equivalents of triethylamine were used as base for the ADHP formation instead of DBU which allowed *i)* the full conversion of the starting pyridine **1** to the ADHP intermediate **1′**, and *ii)* isolation of **1′** by trituration with diethyl ether thereby avoiding the buffer workup previously required to remove DBU,
2) No work-up was done after the allylation step; the reaction mixture was concentrated and directly purified using flash chromatography.

This improved protocol (detailed in **procedure 4**) was used to conduct substrate scope studies.

Palladium-Catalyzed Allylation of 4-Alkylpyridines: Procedure and Structural Data.

Procedure 4: General procedure for Pd-catalyzed pyridylic allylation of 4-alkylpyridines

Synthesis of the ADHP intermediate: Triethylamine (3.0 equiv.) was added to a solution of the appropriate pyridine (1.0 equiv.) in dry THF (0.1 M) in a flame-dried round-bottomed flask equipped with a stir bar. The resulting solution was stirred at room temperature and allyl chloroformate (2.0 equiv.) was added dropwise. Upon disappearance of the starting pyridine, the mixture was concentrated *in vacuo*. The resulting crude mass was suspended in diethyl ether and filtered through a plug of cotton to remove the triethylammonium chloride salt. The filtrate was concentrated *in vacuo* to afford the desired ADHP which was used in the next step without further purification.

Palladium-catalyzed decarboxylative allylation: To an oven-dried round-bottomed flask equipped with a stir bar was added Xantphos (1 mol%) and $Pd(dba)_2$ (1 mol%). Dry THF was introduced and the resulting solution was allowed to stir for 10 minutes at room temperature under an inert atmosphere of argon. A solution of the ADHP in dry THF was then added to the flask containing the catalyst and the mixture was stirred at room temperature and monitored by TLC. Upon completion, the reaction mixture was concentrated *in vacuo* and purified using flash chromatography, eluting with the indicated solvent mixture to afford the desired product.

4-(2-Phenylethyl)pyridine coupled product (**1A**)

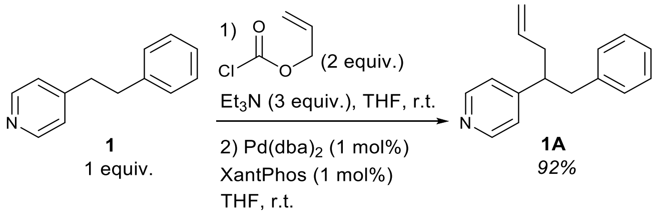

Using procedure 4, pyridine **1** (0.20 g, 1.09 mmol, 1.0 equiv.) provided product **1A** (0.23 g, 1.01 mmol) in 93% yield as a yellow oil.

Chromatography: 20% EtOAc in hexane (R_f. = 0.30).

Data for **1A**

^{1}H-NMR (400 MHz, $CDCl_3$)
δ 8.45 (d, *J* = 5.6 Hz, 2 H), 7.24-7.13 (m, 3 H), 7.01-7.00 (m, 4 H), 5.63 (dddd, *J* =17.2, 10.4, 6.8, 6.8 Hz, 1 H), 4.97 (d, *J* = 17.2 Hz, 1 H), 4.96 (d, *J* = 10.4 Hz, 1 H), 2.99 (dd, *J* = 12.4, 6.8 Hz, 1 H), 2.97-2.89 (m, 1 H), 2.83 (dd, *J* = 12.4, 7.4 Hz, 1 H), 2.50-2.36 (m, 2 H).

^{13}C-NMR (101 MHz, $CDCl_3$)
δ 153.5, 149.8, 139.5, 135.8, 129.2, 128.4, 126.3, 123.5, 117.2, 47.4, 42.1, 39.4.

IR Alpha-Platinum ATR, Bruker, diamond crystal
υ = 3064, 3026, 2923, 1640, 1597, 1557, 1495, 1453, 1413, 910 cm^{-1}

HRMS ESI
Calculated mass for $(M+H)^+$ of $C_{13}H_{17}N$ is 224.1434, found 224.1424.

4-Pentylpyridine coupled product (2A)

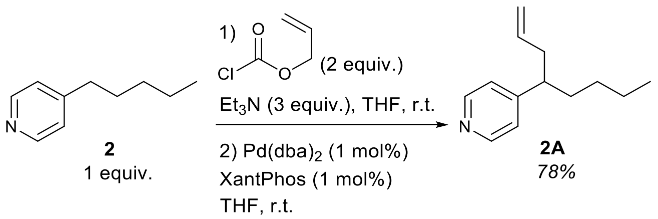

Using procedure 4, pyridine **2** (0.30 g, 2.0 mmol, 1.0 equiv.) provided product **2A** (0.29 g, 1.6 mmol) in 78% yield as a yellow oil.

Chromatography: 20% EtOAc in Hexane ($R_{f.}$ = 0.40).

Data for **2A**

^{1}H-NMR (400 MHz, $CDCl_3$)

δ 8.48 (d, *J* = 5.6 Hz, 2 H), 7.05 (d, *J* = 5.6 Hz, 2 H),
5.59 (dddd, *J* = 17.2, 10.0, 7.2, 7.2 Hz, 1 H), 4.92 (d, *J* = 17.2 Hz, 1 H),
4.91 (d, *J* = 10.0 Hz, 1 H), 2.61-2.54 (m, 1 H), 2.40-2.26 (m, 2 H),
1.73-1.64 (m, 1 H), 1.56-1.44 (m, 1 H), 1.31-1.08 (m, 4 H),
0.81 (t, *J* = 7.2 Hz, 3 H).

^{13}C-NMR (101 MHz, $CDCl_3$)

δ 154.4, 149.6, 136.0, 123.2, 116.5, 45.3, 40.5, 34.9, 29.4, 22.6, 13.9.

IR Alpha-Platinum ATR, Bruker, diamond crystal

υ = 3069, 2957, 2927, 2858, 1640, 1597, 1557, 1492, 1465, 1378, 912 cm^{-1}

HRMS ESI

Calculated mass for $(M+H)^+$ of $C_{13}H_{19}N$ is 190.1590, found 190.1582.

4-Benzylpyridine coupled product (**3A**, CAS 856856-59-2)

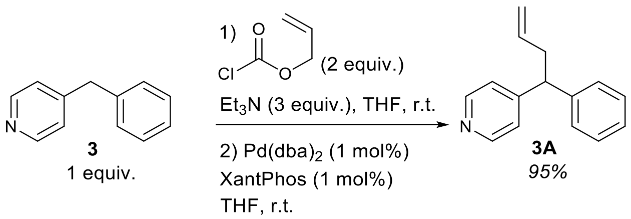

Using procedure 4, pyridine **3** (0.20 g, 1.81 mmol, 1 equiv.) provided product **3A** (0.23 g, 1.08 mmol) in 93% yield as a yellow oil. Spectral data is consistent with that reported in the literature.[29]

Chromatography: 15% Acetone in Hexane (R_f. = 0.25).

Data for **3A**

^{1}H-NMR (400 MHz, $CDCl_3$)

δ 8.49 (d, J = 5.8 Hz, 2 H), 7.32-7.29 (m, 2 H), 7.23 (m, 3 H), 7.15 (d, J = 5.8 Hz, 2 H), 5.68 (dddd, J = 17.0, 10.4, 7.2, 7.2 Hz, 1 H), 5.04 (d, J = 17 Hz, 1 H), 4.98 (d, J = 10.4 Hz, 1 H), 3.99 (t, J = 7.8 Hz, 1 H), 2.81 (t, J = 7.2 Hz, 2 H).

^{13}C-NMR (101 MHz, $CDCl_3$)

δ 153.4, 150.0, 142.7, 135.9, 128.8, 128.1, 126.9, 123.4, 117.2, 50.7, 39.3.

4-[(2-Methylphenyl)methyl]pyridine coupled product (4A)

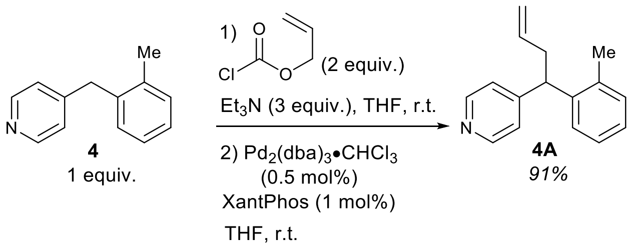

Using procedure 4, pyridine **4** (108 mg, 0.59 mmol, 1.0 equiv.) provided product **4A** (120 mg, 0.54 mmol) in 91% yield as a yellow oil.

Chromatography: 75% EtOAc in Hexanes ($R_{f.}$ = 0.3).

Data for **4A**

^{1}H-NMR (300 MHz, $CDCl_3$)
δ 8.45 (d, *J* = 7.0 Hz, 2 H), 7.26-7.14 (m, 2 H), 7.13 (d, *J* = 3.6 Hz, 2 H), 7.07 (d, *J* = 7.0 Hz, 2 H), 5.77-5.63 (m, 1 H), 5.04-4.95 (m, 2 H), 4.16 (t, *J* = 7.8 Hz, 1 H), 2.79-2.72 (m, 2 H), 2.22 (s, 3 H).

^{13}C-NMR (76 MHz, $CDCl_3$)
δ 153.1, 149.8, 140.5, 136.3, 135.9, 130.7, 126.9, 126.7, 126.3, 123.6, 117.2, 46.3, 39.6, 19.9.

IR Alpha-Platinum ATR, Bruker, diamond crystal
υ = 3021, 1594, 1411, 993, 848, 748, 640, 559 cm^{-1}

HRMS DART
Calculated mass for $(M+H)^+$ of $C_{16}H_{18}N$ is 224.1434, found 224.1441.

4-[(3-Chlorophenyl)methyl]pyridine coupled product (5A)

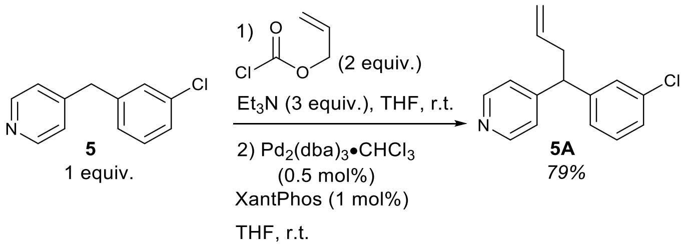

Using procedure 4, pyridine **5** (80 mg, 0.39 mmol, 1.0 equiv.) provided product **5A** (92 mg, 0.31 mmol) in 79% yield as a yellow oil.

Chromatography: 80% EtOAc in Hexane ($R_{f.}$ = 0.4).

Data for **5A**

^{1}H-NMR (400 MHz, $CDCl_3$)

δ 8.50 (d, J = 6.0 Hz, 2H), 7.22-7.17 (m, 3 H), 7.11 (d, J = 6.4Hz, 2 H), 7.08-7.06 (m, 1 H), 5.68-5.59 (m, 1 H), 5.04-4.96 (m, 2 H), 3.94 (t, J = 8.0 Hz, 1 H), 2.79-2.75 (m, 2 H).

^{13}C-NMR (101 MHz, $CDCl_3$)

δ 152.4, 150.0, 144.7, 135.2, 134.5, 129.9, 128.1, 127.1, 126.2, 123.2, 117.5, 50.2, 39.0.

IR Alpha-Platinum ATR, Bruker, diamond crystal

υ = 3024, 1592, 1475, 1412, 993, 915, 694 cm^{-1}

HRMS DART

Calculated mass for $(M+H)^+$ of $C_{15}H_{15}NCl$ is 224.0888, found 224.0892.

4-(4-Pyridinylmethyl)benzonitrile coupled product (6A)

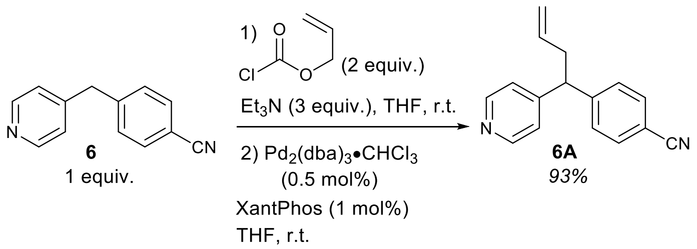

Using procedure 4, pyridine **6** (115 mg, 0.59 mmol, 1.0 equiv.) provided product **6A** (121 mg, 0.55 mmol) in 93% yield as an orange oil.

Chromatography: 100 % EtOAc in Hexane (R_f. = 0.3).

Data for **6A**

^{1}H-NMR (300 MHz, $CDCl_3$)

δ 8.42 (d, J = 6 Hz, 2 H), 7.58 (d, J = 8.1 Hz, 2 H), 7.30 (d, J = 8.4 Hz, 2 H), 7.10 (d, J = 6.0 Hz, 2 H), 5.70-5.56 (m, 1 H), 5.05-4.98 (m, 2 H), 4.03 (t, J = 7.8 Hz, 1 H), 2.82-2.76 (m, 2 H).

^{13}C-NMR (76 MHz, $CDCl_3$)

δ 151.6, 150.2, 148.0, 134.7, 132.5, 128.8, 123.2, 118.6, 117.6, 110.9, 50.5, 38.8.

IR Alpha-Platinum ATR, Bruker, diamond crystal

υ = 3027, 2226, 1593, 1412, 993, 872, 815, 561 cm^{-1}

HRMS DART

Calculated mass for $(M+H)^+$ of $C_{16}H_{15}N_2$ is 235.1230, found 235.1235.

***N,N*-Dimethyl-4-(4-pyridinylmethyl)benzenamine coupled product (7A)**

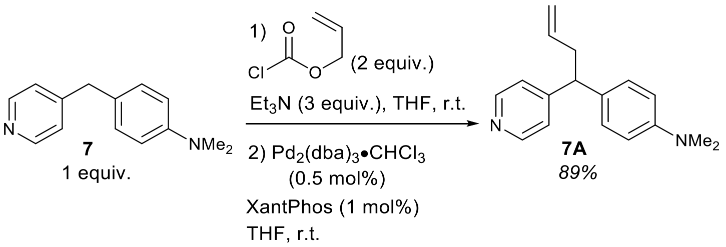

Using procedure 4, pyridine **7** (118 mg, 0.59 mmol, 1.0 equiv.) provided product **7A** (139 mg, 0.53 mmol) 89% yield as an orange oil.

Chromatography: 90% EtOAc in Hexane ($R_{f.}$ = 0.30).

Data for **7A**

^{1}H-NMR (300 MHz, $CDCl_3$)

δ 8.45 (d, *J* = 6.0 Hz, 2 H), 7.10 (d, *J* = 6.3 Hz, 2 H), 6.66 (d, *J* = 8.7 Hz, 2 H), 5.68 (d, *J* = 8.7 Hz, 2 H), 5.75-5.61 (m, 1 H), 5.04-4.93 (m, 2 H), 3.87 (t, *J* = 7.8 Hz, 1 H), 2.90 (s, 6 H), 2.77-2.72 (m, 2 H).

^{13}C-NMR (76 MHz, $CDCl_3$)

δ 154.2, 149.8, 149.4, 136.3, 130.4, 128.5, 123.3, 116.7, 112.7, 49.7, 40.6, 39.4.

IR Alpha-Platinum ATR, Bruker, diamond crystal

υ = 2977, 1594, 1518, 1346, 912, 811, 552 cm^{-1}

HRMS DART

Calculated mass for $(M+H)^+$ of $C_{17}H_{21}N_2$ is 253.1699, found 253.1695.

4-(But-3-en-1-yl)pyridine coupled product (8A)

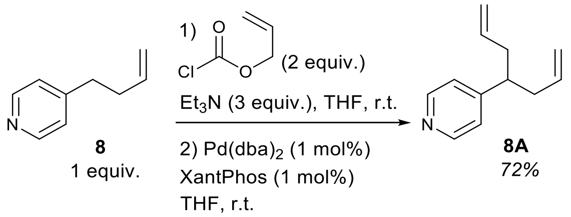

Using procedure 4, pyridine **8** (0.20 g, 1.5 mmol, 1.0 equiv.) provided product **8A** (0.19 g, 1.1 mmol) in 72% yield as a yellow oil.

Chromatography: 25% EtOAc in hexanes (R_f. = 0.30).

Data for **8A**

^{1}H-NMR (400 MHz, $CDCl_3$)
δ 8.50 (d, *J* = 5.4 Hz, 2 H), 7.07 (d, *J* = 5.4 Hz, 2 H), 5.62 (dddd, *J* = 16.4, 10.8, 7.2, 7.2 Hz, 2 H), 4.97 (dd, *J* = 16.4, 1.2 Hz, 2 H), 4.96 (d, *J* = 10.8 Hz, 2 H), 2.75-2.68 (m, 1 H), 2.47-2.30 (m, 4 H).

^{13}C-NMR (101 MHz, $CDCl_3$)
δ 153.4, 149.6, 135.5, 123.2, 116.9, 44.9, 39.4.

IR Alpha-Platinum ATR, Bruker, diamond crystal
υ = 3075, 2923, 1640, 1597, 1413, 993, 911, 784 cm^{-1}

HRMS ESI
Calculated mass for $(M+H)^+$ of $C_{12}H_{15}N$ is 174.1277, found 174.1275.

4-(3-Butyn-1-yl)pyridine coupled product (9A)

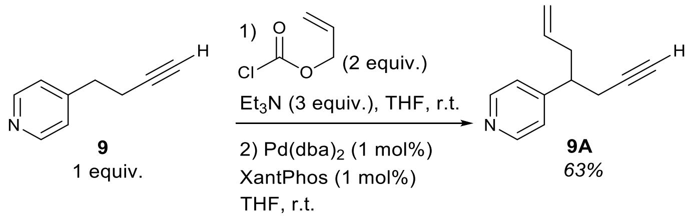

Using procedure 4, pyridine **9** (0.20 g, 1.5 mmol, 1.0 equiv.) provided product **9A** (0.16 g, 0.96 mmol) in 63% yield as a yellow oil.

Chromatography: 33% EtOAc in toluene (R_f. = 0.35).

Data for **9A**

^{1}H-NMR (400 MHz, $CDCl_3$)

δ 8.53 (d, *J* = 6.0 Hz, 2 H), 7.14 (d, *J* = 6.0 Hz, 2 H), 5.63 (dddd, *J* = 17.2, 10.0, 6.8, 6.8 Hz, 1 H), 5.03 (d, *J* = 17.2, 1 H), 5.03 (d, *J* = 10.0, 1 H), 2.90-2.83 (m, 1 H), 2.62-2.39 (m, 4 H), 1.96 (t, *J* = 2.8 Hz, 1 H).

^{13}C-NMR (101 MHz, $CDCl_3$)

δ 152.3, 150.0, 135.1, 123.2, 117.8, 81.6, 70.6, 43.7, 38.6, 24.5.

IR Alpha-Platinum ATR, Bruker, diamond crystal

υ = 3298, 2912, 1640, 1599, 1557, 1414, 994, 917, 816 cm^{-1}

HRMS ESI

Calculated mass for $(M+H)^+$ of $C_{12}H_{13}N$ is 172.1121, found 172.1121.

4-(Oct-1-en-4-yl)pyridine coupled product (10A)

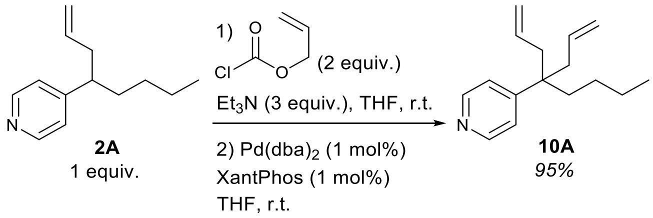

Using procedure 4, pyridine **2A** (0.25 g, 1.32 mmol, 1.0 equiv.) provided product **10A** (0.290 g, 1.25 mmol) in 95% yield as a colorless oil.

Chromatography: 14% EtOAc in hexanes ($R_{f.}$ = 0.30).

Data for **10A**

^{1}H-NMR (600 MHz, $CDCl_3$)

δ 8.52 (d, *J* = 6.0 Hz, 2 H), 7.20 (d, *J* = 6.0 Hz, 2 H), 5.51 (ddt, *J* = 16.2, 10.8, 7.2 Hz, 2 H), 5.00 (d, *J* = 10.8 Hz, 2 H), 5.01 (d, *J* = 16.2 Hz, 2 H), 2.42 (d, *J* = 7.2 Hz, 4 H), 1.64-1.61 (m, 2 H), 1.21 (sext, *J* = 7.2 Hz, 2 H), 1.03-0.96 (m, 2 H), 0.82 (t, *J* = 7.2 Hz, 3 H).

^{13}C-NMR (101 MHz, $CDCl_3$)

δ 155.9, 149.6, 133.6, 122.1, 118.1, 43.5, 41.0, 37.1, 25.4, 23.1, 13.9.

IR Alpha-Platinum ATR, Bruker, diamond crystal

υ = 3076, 2956, 2931, 2862, 1639, 1595, 1455, 1410, 995, 912, 730 cm^{-1}

HRMS ESI

Calculated mass for $(M+H)^+$ of $C_{16}H_{23}N$ is 230.1903, found 230.1898.

4-(2-Bromophenethyl)pyridine coupled product (11A)

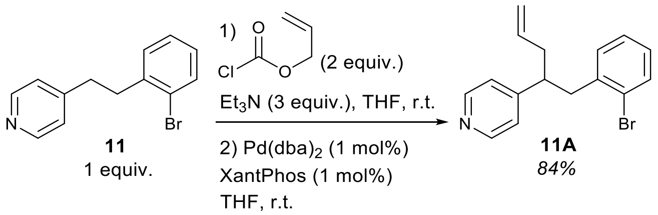

Using procedure 4, pyridine **11** (0.20 g, 0.76 mmol, 1.0 equiv.) provided product **11A** (0.19 g, 0.64 mmol) in 84% yield as a colorless oil.

Chromatography: 50% EtOAc in toluene ($R_{f.}$ = 0.30).

Data for **11A**

^{1}H-NMR (400 MHz, $CDCl_3$)

δ 8.45 (d, *J* = 5.2 Hz, 2 H), 7.51 (d, *J* = 7.6 Hz, 1 H), 7.09-7.01 (m, 4 H), 6.85 (d, *J* = 7.6 Hz, 1 H), 5.68 (dddd, *J* = 16.8, 10.0, 6.8, 6.8 Hz, 1 H), 5.00 (d, *J* = 16.8 Hz, 1 H), 4.96 (d, *J* = 10.0 Hz, 1 H), 3.16 (dd, *J* = 13.2, 6.8 Hz, 1 H), 3.12-3.05 (m, 1 H), 2.88 (dd, *J* = 13.2, 8.0 Hz, 1 H), 2.51-2.46 (m, 2 H).

^{13}C-NMR (101 MHz, $CDCl_3$)

δ 153.1, 149.9, 138.9, 135.7, 133.1, 131.5, 128.2, 127.3, 124.8, 123.5, 117.3, 45.3, 42.5, 39.3.

IR Alpha-Platinum ATR, Bruker, diamond crystal

υ = 3068, 2924, 1639, 1597, 1493, 1470, 1438, 1023, 913, 746 cm^{-1}

HRMS ESI

Calculated mass for $(M+H)^+$ of $C_{16}H_{16}BrN$ is 302.0539, found 302.0540.

4-(4-Chlorobutyl)pyridine coupled product (12A)

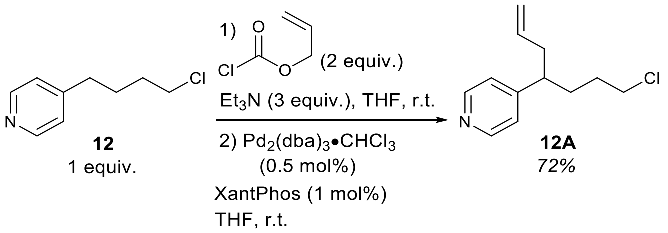

Using procedure 4, pyridine **12** (0.17 g, 1 mmol, 1.0 equiv.) provided product **12A** (0.15 g, 0.72 mmol) in 72% yield as a yellow oil.

Chromatography: 50% EtOAc in toluene (R_f. = 0.30).

Data for **12A**

^{1}H-NMR (300 MHz, $CDCl_3$)

δ 8.50 (d, *J* = 5.7 Hz, 2 H), 7.06 (dd, *J* = 4.5, 1.5 Hz, 2 H)
5.59 (dddd, *J* = 16.5, 9.6, 7.2, 7.2 Hz, 1 H), 4.98-4.92 (m, 2 H),
3.45 (t, *J* = 6.3 Hz, 2 H), 2.61-2.58 (m, 1 H), 2.38-2.32 (m, 2 H),
1.88-1.83 (m, 1 H), 1.72-1.45 (m, 3 H).

^{13}C-NMR (76 MHz, $CDCl_3$)

δ 153.4, 149.8, 135.4, 123.1, 117.0, 44.7, 40.5, 32.2, 30.2.

IR Alpha-Platinum ATR, Bruker, diamond crystal

υ = 3070, 2926, 1598, 817 cm^{-1}

HRMS DART

Calculated mass for $(M+H)^+$ $C_{12}H_{17}NCl$ of is 210.1044, found 210.1046.

4-(4-((*tert*-Butyldimethylsilyl)oxy)butyl)pyridine coupled product (13A)

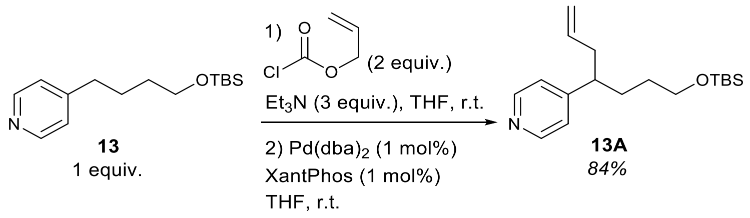

Using procedure 4, pyridine **13** (0.40 g, 1.5 mmol, 1.0 equiv.) provided product **13A** (0.38 g, 1.3 mmol) in 84% yield as a colorless oil.

Chromatography: 40% EtOAc in hexanes (R_f. = 0.40).

Data for **13A**

^{1}H-NMR (400 MHz, $CDCl_3$)
δ 8.50 (d, *J* = 5.6 Hz, 2 H), 7.07 (d, *J* = 5.6 Hz, 2 H), 5.61 (dddd, *J* = 17.2, 10.4, 7.2, 7.2 Hz, 1 H), 4.95 (d, *J* = 17.2 Hz, 1 H), 4.94 (d, *J* = 10.4 Hz, 1 H), 3.54 (t, *J* = 6.4 Hz, 2 H), 2.65-2.58 (m, 1 H), 2.40-2.31 (m, 2 H), 2.81-2.74 (m, 1 H), 1.65-1.57 (m, 1 H), 1.41-1.31 (m, 2 H), 0.87 (s, 9 H), 0.01 (d, *J* = 2.0 Hz, 6 H).

^{13}C-NMR (101 MHz, $CDCl_3$)
δ 154.2, 149.7, 135.9, 123.3, 116.7, 62.8, 45.1, 40.6, 31.4, 30.5, 25.9, 18.3, 5.34.

^{29}Si-NMR (57 MHz, $CDCl_3$)
δ 18.78.

IR Alpha-Platinum ATR, Bruker, diamond crystal
υ = 3070, 2951, 2928, 2856, 1641, 1597, 1471, 1413, 1253, 1096, 912, 773 cm^{-1}

HRMS ESI
Calculated mass for $(M+H)^+$ of $C_{18}H_{31}NOSi$ is 306.2248, found 306.2240.

4-(3-(1,3-Dioxolan-2-yl)propyl)pyridine coupled product (**14A**)

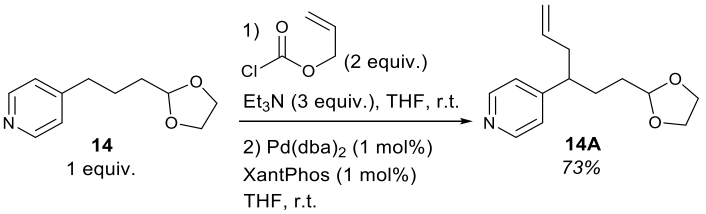

Using procedure 4**,** pyridine **14** (0.25 g, 1.29 mmol, 1.0 equiv.) provided product **14A** (0.22 g, 0.94 mmol) in 73% yield as a colorless oil.

Chromatography: 80% EtOAc in toluene ($R_{f.}$ = 0.40).

Data for **14A**

^{1}H-NMR (400 MHz, $CDCl_3$)

δ 8.51 (br, 2 H), 7.08 (d, *J* = 5.2 Hz, 2 H), 5.60 (dddd, *J* = 16.8, 9.6, 6.8, 6.8 Hz, 1 H), 4.97 (d, *J* = 16.8 Hz, 1 H), 4.95 (d, *J* = 9.6 Hz, 1 H), 4.79 (t, *J* = 4.8 Hz, 1 H), 3.94-3.80 (m, 4 H), 2.43-2.28 (m, 2 H), 1.87-1.82 (m, 1 H),1.72-1.61 (m, 1 H), 1.58-1.40 (m, 2 H).

^{13}C-NMR (101 MHz, $CDCl_3$)

δ 153.9, 149.98, 135.8, 123.4, 117.0, 104.3, 65.0, 45.3, 40.6, 31.8, 29.4.

IR Alpha-Platinum ATR, Bruker, diamond crystal

υ = 3071, 2882, 1640, 1598 cm^{-1}

HRMS ESI

Calculated mass for $(M+H)^+$ of $C_{14}H_{19}NO_2$ is 234.1489, found 234.1487.

4-[2-(4-Morpholinyl)ethyl]pyridine coupled product (15A)

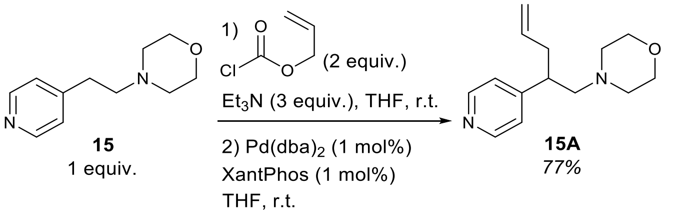

Using procedure 4, pyridine **15** (0.19 g, 1.0 mmol, 1.0 equiv.) provided product **15A** (0.18 g, 0.77 mmol) in 77% yield as a yellow oil.

Chromatography: 5% Et_3N : 60% EtOAc : 35% hexane (R_f. = 0.40).

Data for **15A**

<u>^{1}H-NMR</u> (400 MHz, $CDCl_3$)

δ 8.50 (d, *J* = 5.6 Hz, 2 H), 7.09 (d, *J* = 5.6 Hz, 2 H), 5.62 (dddd, *J* = 17.2, 10.8, 7.2, 7.2 Hz, 1 H), 4.95 (d, *J* = 17.2 Hz, 1 H), 4.94 (d, *J* = 10.8 Hz, 1 H), 3.62 (t, *J* = 4.4 Hz, 4 H), 2.91-2.84 (m, 1 H), 2.58-2.48 (m, 3 H), 2.40 (t, *J* = 4.4 Hz, 4 H), 2.35-2.27 (m, 1 H).

<u>^{13}C-NMR</u> (101 MHz, $CDCl_3$)

δ 153.0, 149.8, 135.8, 123.5, 117.0, 67.1, 63.7, 54.1, 42.8, 38.1.

<u>IR</u> Alpha-Platinum ATR, Bruker, diamond crystal

υ = 3071, 2854, 1640, 1558 cm^{-1}

<u>HRMS</u> ESI

Calculated mass for $(M+H)^+$ of $C_{14}H_{20}N_2O$ is 233.1648, found 133.1646.

4-Pyridineheptanenitrile coupled product (16A)

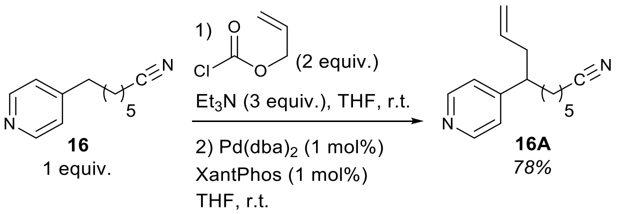

Using procedure 4, pyridine **16** (0.19 g, 1.0 mmol, 1.0 equiv.) provided product **16A** (0.18 g, 0.78 mmol) in 78% yield as a yellow oil.

Chromatography: 67% EtOAc in hexanes (R_f. = 0.30).

Data for **16A**

^{1}H-NMR (400 MHz, $CDCl_3$)

δ 8.50 (d, *J* = 5.2 Hz, 2 H), 7.06 (d, *J* = 5.2 Hz, 2 H), 5.60 (dddd, *J* = 17.2, 10.4, 7.2, 7.2 Hz, 1 H), 4.95 (d, *J* = 17.2 Hz, 1 H), 4.94 (d, *J* = 10.4 Hz, 1 H), 2.63-2.55 (m, 1 H), 2.40-2.31 (m, 2 H), 2.28 (t, *J* = 7.2 Hz, 2 H), 1.72-1.67 (m, 1 H), 1.62-1.54 (m, 3 H),1.44-1.24 (m, 2 H), 1.20-1.11 (m, 2 H).

^{13}C-NMR (101 MHz, $CDCl_3$)

δ 154.0, 149.8, 135.7, 123.1, 119.6, 116.8, 45.2, 40.5, 34.8, 28.5, 26.5, 25.1, 17.1.

IR Alpha-Platinum ATR, Bruker, diamond crystal

υ = 3071, 2854, 1640, 1558 cm^{-1}

HRMS ESI

Calculated mass for $(M+H)^+$ of $C_{15}H_{20}N_2$ is 229.1699, found 229.1686.

Ethyl 3-(pyridin-4-yl)propanoate coupled product (17A)

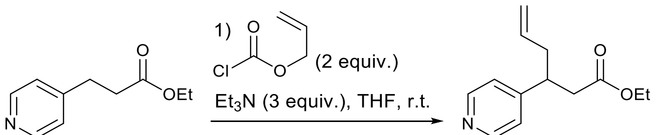

Using procedure 4, pyridine **17** (0.18 g, 1.0 mmol, 1.0 equiv.) provided product **17A** (0.20 g, 0.90 mmol) in 90% yield as a yellow oil.

Chromatography: 60% EtOAc in hexanes (R_f. = 0.25).

Data for **17A**

^{1}H-NMR (400 MHz, $CDCl_3$)

δ 8.51 (d, *J* = 5.2 Hz, 2 H), 7.12 (d, *J* = 5.2 Hz, 2 H),
5.62 (ddt, *J* = 16.8, 9.2, 7.2 Hz, 1 H),
5.01 (d, *J* = 16.8 Hz, 1 H), 5.00 (d, *J* = 9.2 Hz, 1 H), 4.03 (q, *J* = 7.2 Hz, 2 H),
3.24-3.17 (m, 1 H), 2.72-2.67 (m, 1 H), 2.59-2.53 (m, 1 H), 2.38 (t, *J* = 7.2 Hz, 2 H),
1.14 (t, *J* = 7.2 Hz, 3 H).

^{13}C-NMR (101 MHz, $CDCl_3$)

δ 171.6, 152.5, 149.9, 134.8, 122.9, 117.7, 60.5, 41.1, 39.9, 39.5, 14.1.

IR Alpha-Platinum ATR, Bruker, diamond crystal

υ = 3071, 2931, 2245, 1640, 1598 cm^{-1}

HRMS ESI

Calculated mass for $(M+H)^+$ of $C_{13}H_{17}NO_2$ is 220.1332, found 220.1326.

4-(Pyridin-4-yl)butan-2-one coupled product (18A)

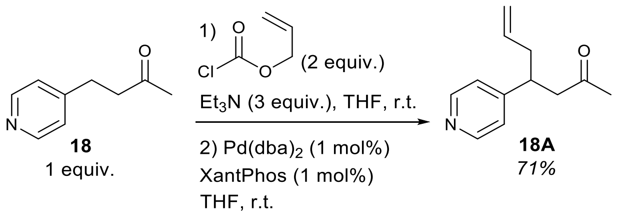

Using procedure 4, pyridine **18** (0.15 g, 1.0 mmol, 1.0 equiv.) provided product **18A** (0.13 g, 0.71 mmol) in 71% yield as a yellow oil.

Chromatography: 100% EtOAc (R_f. = 0.40).

Data for **18A**

^{1}H-NMR (400 MHz, $CDCl_3$)

δ 8.50 (d, *J* = 5.2 Hz, 2 H), 7.11 (d, *J* = 5.2 Hz, 2 H), 5.60 (ddt, *J* =16.2, 9.2, 7.2 Hz, 1 H), 4.98 (d, *J* = 16.8 Hz, 1 H), 4.97 (d, *J* = 9.2 Hz, 1 H), 3.27 (m, 1 H), 2.83-2.69 (m, 2 H), 2.35 (t, *J* = 7.2 Hz, 2 H), 2.07 (s, 3 H).

^{13}C-NMR (101 MHz, $CDCl_3$)

δ 206.5, 153.2, 150.0, 135.2, 123.1, 117.7, 48.5, 40.1, 39.8, 30.7.

IR Alpha-Platinum ATR, Bruker, diamond crystal

υ = 3070, 2980, 1729, 1641, 1599 cm^{-1}

HRMS ESI

Calculated mass for $(M+H)^+$ of $C_{12}H_{15}NO$ is 190.1226, found 190.1226.

4-(Pyridin-4-yl)butanal coupled product (19A)

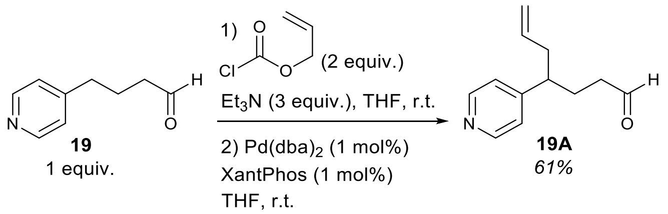

Using procedure 4, pyridine **19** (0.10 g, 0.67 mmol, 1.0 equiv.) provided product **19A** (0.08 g, 0.41 mmol) in 61% yield as a yellow oil.

Chromatography: 70% EtOAc in hexanes (R_f. = 0.30).

Data for **19A**

^{1}H-NMR (400 MHz, $CDCl_3$)

δ 9.67 (s, 1 H), 8.51 (d, *J* = 5.2 Hz, 2 H), 7.05 (d, *J* = 5.2 Hz, 2 H), 5.60 (dddd, *J* =17.2, 10.0, 6.8, 6.8 Hz, 1 H), 4.96 (d, *J* = 17.2 Hz, 1 H), 4.95 (d, *J* = 10.0 Hz, 1 H), 2.67-2.60 (m, 1 H), 2.39-2.34 (m, 2 H), 2.31-2.27 (m, 2 H), 2.12-2.04 (m, 1 H), 1.87-1.80 (m, 1 H).

^{13}C-NMR (101 MHz, $CDCl_3$)

δ 201.4, 152.9, 149.9, 135.2, 123.1, 117.2, 44.4, 41.6, 40.4, 27.1.

IR Alpha-Platinum ATR, Bruker, diamond crystal

υ = 3074, 2924, 1713, 1640, 1598 cm^{-1}

HRMS ESI

Calculated mass for $(M+H)^+$ of $C_{12}H_{15}NO$ is 190.1226, found 190.1220.

4-Butyl-3-phenylpyridine coupled product (20A)

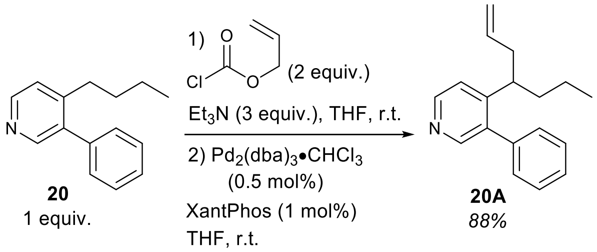

Using procedure 4, pyridine **20** (75 mg, 0.35 mmol, 1.0 equiv.) provided product **20A** (78 mg, 0.31 mmol) in 88% yield as a colorless oil.

Chromatography: 30% EtOAc in hexanes (R_f. = 0.25).

Data for **20A**

^{1}H-NMR (400 MHz, $CDCl_3$)

δ 8.53 (d, J = 5.2 Hz, 1 H), 8.41 (s, 1 H), 7.44-7.36 (m, 3 H), 7.26 (d, J = 8.4 Hz, 2 H), 7.22 (d, J = 5.2 Hz, 1 H), 5.54 (ddt, J = 17.2, 10.4, 7.2 Hz, 1 H), 4.90 (d, J = 10.4 Hz, 1 H), 4.89 (d, J = 17.2 Hz, 1 H), 2.93-2.85 (m, 1 H), 2.28 (t, J = 7.2 Hz, 2 H), 1.60-1.48 (m, 2 H), 1.01-1.01 (m, 2 H), 0.73 (t, J = 7.6 Hz, 3 H).

^{13}C-NMR (101 MHz, $CDCl_3$)

δ 152.1, 150.2, 148.6, 138.3, 138.0, 136.0, 129.8, 128.2, 127.4, 121.0, 116.5, 40.8, 39.6, 38.0, 20.4, 14.0.

IR Alpha-Platinum ATR, Bruker, diamond crystal

υ = 3072, 2925, 2724, 1721, 1640, 1598 cm^{-1}

HRMS ESI

Calculated mass for $(M+H)^+$ of $C_{17}H_{19}N$ is 238.1590, found 238.1589.

3-Methyl-4-(phenylmethyl)pyridine coupled product (21A)

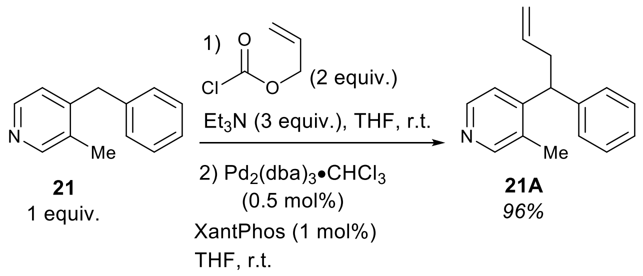

Using procedure 4, pyridine **21** (75 mg, 0.41 mmol, 1.0 equiv.) provided product **21A** (91 mg, 0.39 mmol) in 96% yield as a yellow oil.

Chromatography: 75% EtOAc in Hexanes ($R_{f.}$ = 0.3).

Data for **21A**

^{1}H-NMR (300 MHz, $CDCl_3$)

δ 8.40 (d, *J* = 6.8 Hz, 1 H), 8,31 (s, 1 H), 7.28-7.18 (m, 6 H), 5.75-5.62 (m, 1 H), 5.04-4.94 (m, 2 H), 4.13 (t, *J* = 10.4 Hz, 1 H), 2.78 (dd, *J* = 9, 6 Hz, 2 H), 2.20 (s, 3 H).

^{13}C-NMR (76 MHz, $CDCl_3$)

δ 151.2, 150.9, 147.8, 142.2, 135.9, 131.9, 128.6, 128.2, 126.6, 121.6, 117.0, 46.6, 39.5, 16.6.

IR Alpha-Platinum ATR, Bruker, diamond crystal

υ = 3025, 1589, 1402, 913, 699, 557 cm^{-1}

HRMS ESI

Calculated mass for $(M+H)^+$ of $C_{16}H_{18}N$ is 224.1434, found 224.1436.

4-Butyl-3-pyridinecarbonitrile coupled product (22A)

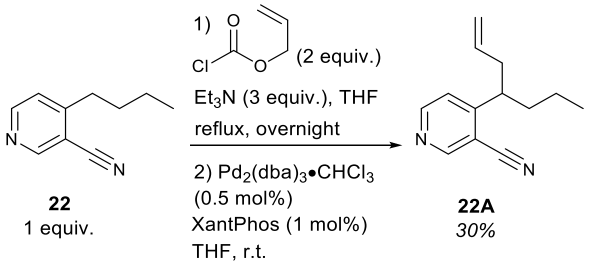

Using procedure 4, pyridine **22** (0.30 g, 1.87 mmol, 1.0 equiv.) in THF (18 mL) reacted with Et_3N (0.45 g, 3.7 mmol, 3.0 equiv.) and Alloc-Cl (0.57 g, 5.6 mmol, 2.0 equiv.) under reflux overnight. The reaction mixture was cooled to room temperature and concentrated under reduced pressure. The resulting crude mass was suspended in diethyl ether and filtered through a plug of cotton to remove the triethylammonium chloride salt. The filtrate was concentrated *in vacuo* to afford the corresponding ADHP (~50% conversion) as a yellow oil. The crude ADHP was then reacted with a $Pd_2(dba)_3$•$CHCl_3$ (10 mg, 0.009 mmol, 0.5 mol%) - XantPhos (11 mg, 0.018 mmol, 1 mol%) catalytic system in THF at room temperature to furnish product **22A** (0.11 g, 0.56 mmol) in 30% yield as a yellow oil.

Chromatography: 20% EtOAc in hexanes (R_f. = 0.25).

Data for **22A**

^{1}H-NMR (400 MHz, $CDCl_3$)

δ 8.80 (s, 1 H), 8.69 (d, *J* = 5.4 Hz, 1 H), 7.25 (d, *J* = 5.4 Hz, 1 H), 5.64 (dddd, *J* = 17.2, 10.0, 7.2, 7.2 Hz, 1 H), 4.97 (d, *J* = 10.0 Hz, 1 H), 4.94 (d, *J* = 17.2 Hz, 1 H), 3.21-3.13 (m, 1 H), 2.53-2.47 (m, 1 H), 2.40-2.33 (m, 1 H), 1.80-1.72 (m, 1 H), 1.67-1.62 (m, 1 H), 1.29-1.15 (m, 2 H), 0.88 (t, *J* = 7.2 Hz, 3 H).

^{13}C-NMR (101 MHz, $CDCl_3$)

δ 158.2, 153.1, 152.6, 134.6, 121.9, 117.7, 116.2, 111.2, 43.4, 39.9, 37.1, 20.4, 13.9.

IR Alpha-Platinum ATR, Bruker, diamond crystal

υ = 3061, 2956, 2930, 1640, 1587 cm^{-1}

HRMS ESI

Calculated mass for $(M+H)^+$ of $C_{13}H_{16}N_2$ is 201.1386, found 201.1385.

N-(*tert*-Butyl)-4-butylnicotinamide coupled product (23A)

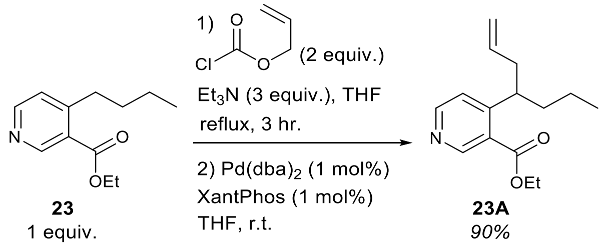

Using procedure 4 with some modification, pyridine **23** (0.15 g, 0.72 mmol, 1.0 equiv.) in THF (7 mL) was reacted with Et_3N (0.22 g, 2.16 mmol, 3.0 equiv.) and Alloc-Cl (0.17 g, 1.4 mmol, 2.0 equiv.) under reflux for 3 h. The reaction mixture was cooled to room temperature and concentrated under reduced pressure. The resulting crude mass was suspended in diethyl ether and filtered through a plug of cotton to remove triethylammonium chloride salt. The filtrate was concentrated *in vacuo* to afford the corresponding alkylidene dihydropyridine as a yellow oil. The alkylidene dihydropyridine was reacted with a $Pd(dba)_2$ (4 mg, 0.007 mmol, 1 mol%) - XantPhos (4 mg, 0.007 mmol, 1 mol%) catalytic system in THF at room temperature to furnish product **23A** (0.16 g, 0.65 mmol) in 90% yield as a yellow oil.

Chromatography: 20% EtOAc in hexanes (R_f. = 0.30).

Data for **23A**

^{1}H-NMR (400 MHz, $CDCl_3$)
δ 8.93 (s, 1 H), 8.60 (d, *J* = 5.2 Hz, 1 H), 7.24 (d, *J* = 5.2 Hz, 1 H), 5.65 (dddd, *J* = 16.8, 9.6, 6.8, 6.8 Hz, 1 H), 4.92 (d, *J* = 16.8 Hz, 1 H), 4.92 (d, *J* = 9.6 Hz, 1 H), 4.39 (q, *J* = 7.2 Hz, 2 H), 3.78-3.71 (m, 1 H), 2.41-2.29 (m, 2 H), 1.68-1.53 (m, 2 H), 1.40 (t, *J* = 7.2 Hz, 3 H), 1.39-1.13 (m, 2 H), 0.85 (t, *J* = 7.6 Hz, 3 H).

^{13}C-NMR (101 MHz, $CDCl_3$)
δ 166.8, 155.9, 152.0, 151.2, 136.0, 127.6, 122.1, 116.8, 61.5, 40.7, 39.3, 37.5, 20.6, 14.4, 14.2.

IR Alpha-Platinum ATR, Bruker, diamond crystal
υ = 3251, 3070, 2961, 1727, 1654, 1590 cm^{-1}

HRMS ESI
Calculated mass for $(M+H)^+$ of $C_{15}H_{21}NO_2$ is 248.1645, found 248.1641.

Ethyl 4-butylnicotinate coupled product (24A)

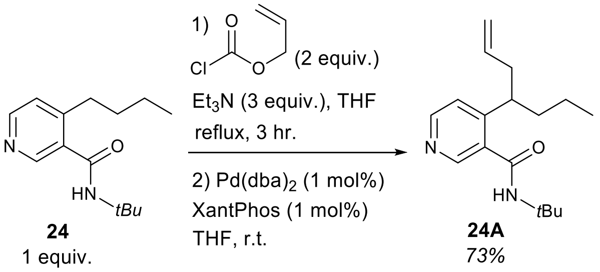

Using procedure 4, pyridine **24** (0.15 g, 0.64 mmol, 1.0 equiv.) in THF (6 mL) reacted with Et_3N (0.19 g, 1.92 mmol, 3.0 equiv.) and Alloc-Cl (0.15 g, 1.28 mmol, 2.0 equiv.) under reflux for 3 h. The reaction mixture was then cooled to room temperature and concentrated under reduced pressure. The resulting crude mass was suspended in diethyl ether and filtered through a plug of cotton to remove triethylammonium chloride salt. The filtrate was concentrated *in vacuo* to afford the corresponding alkylidene dihydropyridine as a yellow solid. The crude alkylidene dihydropyridine was then reacted with $Pd(dba)_2$ (4 mg, 0.006 mmol, 1 mol%) - XantPhos (4 mg, 0.006 mmol, 1 mol%) catalytic system in THF at room temperature to furnish product **24A** (0.13 g, 0.47 mmol) in 73% yield as a colorless oil.

Chromatography: 80% EtOAc in hexanes (R_f. = 0.30).

Data for **24A**

^{1}H-NMR (400 MHz, $CDCl_3$)
δ 8.52 (d, *J* = 5.2 Hz, 1 H), 8.48 (s, 1 H), 7.18 (d, *J* = 5.2 Hz, 1 H), 5.67-5.58 (m, 1 H), 4.93 (d, *J* = 16.0 Hz, 1 H), 4.92 (d, *J* = 11.2 Hz, 1 H), 3.22-3.16 (m, 1 H), 2.42-2.29 (m, 2 H), 1.69-1.57 (m, 2 H), 1.46 (s, 9 H), 1.26-1.11 (m, 2 H), 0.85 (t, *J* = 7.2 Hz, 3 H).

^{13}C-NMR (101 MHz, $CDCl_3$)
δ 167.1, 152.6, 150.3, 147.2, 136.2, 136.1, 121.8, 116.8, 52.3, 40.7, 40.3, 37.7, 28.8, 20.6, 14.0.

IR Alpha-Platinum ATR, Bruker, diamond crystal
υ = 3079, 2959, 2228, 1641, 1687 cm^{-1}

HRMS ESI
Calculated mass for $(M+H)^+$ of $C_{17}H_{26}N_2O$ is 275.2118, found 275.2109.

***tert*-Butyl (4-phenethylpyridin-3-yl)carbamate coupled product (25A)**

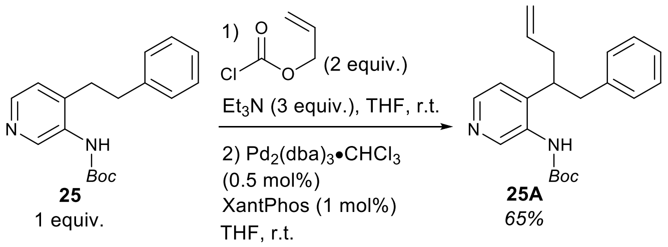

Using procedure 4, pyridine **25** (100 mg, 0.33 mmol, 1.0 equiv.) provided product **25A** (73 mg, 0.21 mmol) in 65% yield as a yellow oil.

Chromatography: 60% Et_2O in toluene (R_f. = 0.40).

Data for **25A**

^{1}H-NMR (400 MHz, $CDCl_3$)

δ 8.59 (br, 1 H), 8.37 (d, J = 5.2 Hz, 1 H), 7.23-7.16 (m, 4 H), 6.96 (d, J = 6.8 Hz, 2 H), 5.65 (dddd, J = 17.2, 10.0, 7.2, 7.2 Hz, 1 H), 5.51 (br, 1 H) 5.02 (d, J = 17.2 Hz, 1 H), 5.00 (d, J = 10.0 Hz, 1 H), 3.14 (br, 1 H), 3.04 (dd, J = 13.2, 6.0 Hz, 1 H), 2.70 (dd, J = 13.2, 8.0 Hz, 1 H), 2.53-2.39 (m, 2 H), 1.46 (s, 9 H).

^{13}C-NMR (101 MHz, $CDCl_3$)

δ 153.5, 147.2, 146.6, 139.4, 135.6, 132.7, 129.1, 128.7, 126.7, 121.4, 117.6, 80.9, 42.1, 41.2, 38.9, 28.4.

IR Alpha-Platinum ATR, Bruker, diamond crystal

υ = 3070, 2959, 1719, 1641, 1589 cm^{-1}

HRMS ESI

Calculated mass for $(M+H)^+$ of $C_{21}H_{26}N_2O_2$ is 339.2067, found 339.2071.

5,6,7,8-Tetrahydroisoquinoline coupled product (26A)

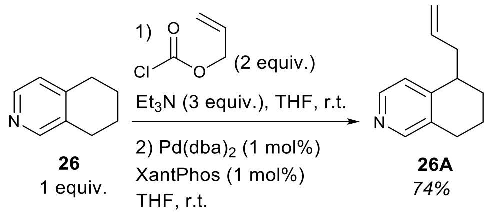

Using procedure 4, pyridine **26** (0.30 g, 2.25 mmol, 1.0 equiv.) provided product **26A** (0.29 g, 1.67 mmol) in 74% yield as a yellow oil.

Chromatography: 25% EtOAc in toluene (R_f. = 0.25).

Data for **26A**

^{1}H-NMR (400 MHz, $CDCl_3$)
δ 8.32 (br, 2 H), 7.11 (d, *J* = 5.2 Hz, 1 H),
5.65 (dddd, *J* = 17.2, 10.8, 7.2, 7.2 Hz, 1 H), 5.09 (d, *J* = 15.6 Hz, 1 H),
5.08 (d, *J* = 11.2 Hz, 1 H), 2.89-2.83 (m, 1 H), 2.75 (br, 2 H),
2.54-2.48 (m, 1 H), 2.38-2.30 (m, 1 H), 1.94-1.83 (m, 2 H), 1.80-1.67 (m, 2H).

^{13}C-NMR (101 MHz, $CDCl_3$)
δ 150.6, 149.3, 146.8, 136.5, 133.1, 123.1, 117.0, 40.4, 36.9, 27.0, 26.7, 19.7.

IR Alpha-Platinum ATR, Bruker, diamond crystal
υ = 3074, 2933, 2861, 1639, 1592 cm^{-1}

HRMS ESI
Calculated mass for $(M+H)^+$ of $C_{12}H_{15}N$ is 174.1277 found 174.1273.

3-(2-(Pyridin-4-yl)ethyl)pyridine coupled product (27A)

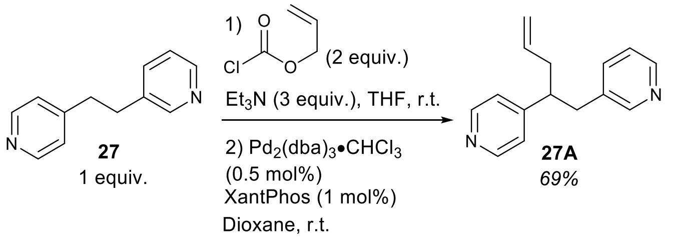

Using procedure 4, pyridine **27** (0.199 g, 1.08 mmol, 1.0 equiv.) and using 1,4-dioxane as solvent in the coupling step (21 mL) provided product **27A** (0.167 g, 0.75 mmol) in 69% yield as a yellow oil.

Chromatography: 5% Et_3N : 20% EtOAc : 75% toluene (R_f. = 0.34).

Data for **27A**

^{1}H-NMR (400 MHz, $CDCl_3$)

δ 8.49 (d, *J* = 8.1 Hz, 2 H), 8.42 (dd, *J* = 4.8, 1.5 Hz, 1 H), 8.31 (d, *J* = 2.0, 1 H), 7.25 (dt, *J* = 7.8, 1.8 Hz, 1 H), 7.13 (dd, *J* = 7.7, 4.8 Hz, 1 H), 7.01 (d, *J* = 6.1 Hz, 2 H), 5.62 (dddd, *J* = 24, 12, 8, 8 Hz, 1 H), 5.02 (d, *J* = 8 Hz, 1 H), 5.01 (d, *J* = 24 Hz, 1 H), 3.02 (dd, *J* = 13.2, 5.5 Hz, 1H), 2.91 (m, 1 H), 2.81 (dd, *J* = 13.2, 8.8 Hz, 1 H), 2.50-2.43 (m, 2 H).

^{13}C-NMR (101 MHz, $CDCl_3$)

δ 152.6, 150.5, 150.0, 147.9, 136.5, 135.3, 134.8, 123.4, 123.3, 117.7, 47.1, 39.5, 39.0.

IR Alpha-Platinum ATR, Bruker, diamond crystal

υ = 3026, 2978, 1640 cm^{-1}

HRMS ESI

Calculated mass for $(M+H)^+$ of $C_{15}H_{17}N_2$ is 225.1386, found 225.1393.

2-(4-(2-(Pyridin-4-yl)ethyl)phenethyl)pyridine coupled product (28A)

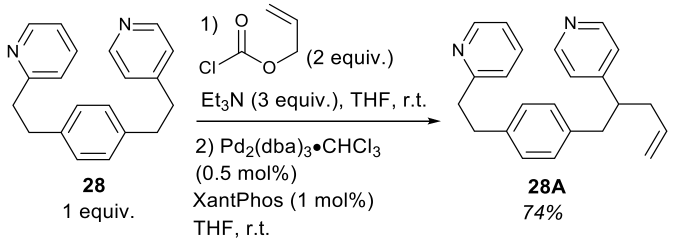

Using procedure 4, pyridine **28** (50 mg, 0.17 mmol, 1.0 equiv.) provided product **28A** (42 mg, 0.12 mmol) in 74% yield as a yellow oil.

Chromatography: 3% MeOH in DCM ($R_{f.}$ = 0.30).

Data for **28A**

^{1}H-NMR (600 MHz, $CDCl_3$)
8.54 (d, J = 4.2 Hz, 1 H), 8.44 (d, J = 4.8 Hz, 2 H), 7.54 (td, J = 7.8, 1.8 Hz, 1 H), 7.10 (dd, J = 4.2, 1.8 Hz, 1 H), 7.01 (d, J = 7.8 Hz, 1 H)*, 7.01 (d, J = 7.8 Hz, 2 H),* 6.99 (d, J = 4.8 Hz, 2 H), 6.88 (d, J = 7.8 Hz, 2 H), 5.54 (dddd, J = 16.8, 10.2, 7.2, 7.2 Hz, 1 H), 4.96 (d, J = 16.8 Hz, 1 H), 4.95 (d, J = 10.2 Hz, 1 H), 3.05-2.87 (m, 6.0 H), 2.78 (dd, J = 12.6, 7.2 Hz, 1 H), 2.46-2.35 (m, 2 H).

^{13}C-NMR (151 MHz, $CDCl_3$)
δ 161.2, 153.4, 149.5, 149.3, 139.3, 136.8, 136.2, 135.7, 129.0, 128.3, 123.4, 122.9, 121.1, 117.0, 47.2, 41.5, 40.1, 39.2, 35.5.

IR Alpha-Platinum ATR, Bruker, diamond crystal
υ = 3069.5, 3007.1, 2922.1, 1596.0, 1473.4, 992.8, 817.6, 748.5 cm^{-1}

HRMS ESI
Calculated mass for $(M+H)^+$ of $C_{23}H_{24}N_2$ is 329.2012, found 329.2003.

* Overlapping doublets with identical chemical shifts and coupling constants.

2-(4-(2-(Pyridin-4-yl)ethyl)phenethyl)pyridine coupled product (28A)

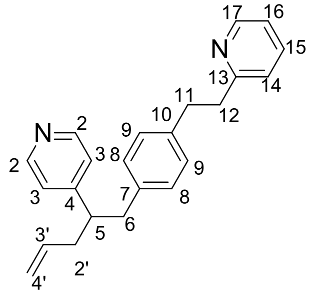

Carbon No.	^{13}C δ (ppm)[a]	^{1}H δ (ppm) (mult; *J* (Hz))[b,c,d]	HMBC Correlations
2	149.5	H-2 : 8.44 (d, *J* = 4.8 Hz)	H-3
3	123.4	H-3 : 6.99 (d, *J* = 4.8 Hz)	H-2, H-5
4	153.4	--	H-2, H-2'a, H-2'b, H-6a, H-6b, H-5
5	47.2	H-5 : part of the m at 2.99-2.87	H-2'a, H-2'b, H-6a, H-6b, H-3
6	41.5	H-6a : part of the m at 2.99-2.87<br>H-6b : 2.78 (dd, *J* = 12.6, 7.2 Hz)	H-5, H-8
7	136.8	--	H-8, H-6a, H-6b
8	129.0	H-8 : 6.88 (d, *J* = 7.8 Hz)	H-6a, H-6b
9	128.3	H-9 : 7.01 (d, *J* = 7.8 Hz)	H-11a, H-11b
10	139.8	--	
11	35.5	H-11a : part of the m at 2.99-2.87<br>H-11b : part of the m at 2.99-2.87	H-9, H-12a, H-12b
12	40.1	H-12a : part of the m at 3.05-2.96.<br>H-12b : part of the m at 3.05-2.96	H-11a, H-11b, H-9
13	161.2	--	H-14, H-15
14	122.9	H-14 : Overlapping with the d at 7.01 (d, *J* = 7.8 Hz)	H-16
15	136.2	H-15 : 7.55 (td, *J* = 7.8, 1.8 Hz)	H-17
16	121.1	H-16 : 7.10 (dd, *J* = 4.2, 1.8 Hz)	H-17, H-14
17	149.3	H-17 : 8.54 (d, *J* = 4.2 Hz)	H-15, H16
2'	39.2	H-2'a : part of the m at 2.46-2.35<br>H-2'b : part of the m at 2.46-2.35	H-4'a-H-4'b, H-3
3'	135.7	H-3' : 5.61 (dddd, *J* = 16.8, 10.2, 7.2, 7.2 Hz)	H-2'a, H-2'b
4'	117.0	H-4'a : 4.96 (d, *J* = 16.8 Hz)<br>H-4'b : 4.95 (d, *J* = 10.2 Hz)	H-2'a, H-2'b

[a] Recorded at 150 MHz. [b] Recorded at 600 MHz.
[c] Assignments based on HSQC-DEPT and HMBC data
[d] Methylene protons are designated H-Xa and H-Xb arbitrarily
[e] Only those correlations which could be unambiguously assigned are reported.

2-(4-(2-(Pyridin-4-yl)ethyl)phenethyl)pyridine coupled product (28A)

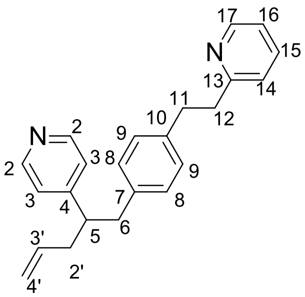

Proton No.	^{1}H δ (ppm) (mult; *J* (Hz))[a,b,c, d]	COSY Correlations
H-2	8.44 (d, *J* = 4.8 Hz)	H-3
H-3	6.99 (d, *J* = 4.8 Hz)	H-2
H-5	part of the m at 2.99-2.87	
H-6a	part of the m at 2.99-2.87	H-6b
H-6b	2.78 (dd, *J* = 12.6, 7.2 Hz)	H-6a
H-8	6.88 (d, *J* = 7.8 Hz)	H-9
H-9	7.01 (d, *J* = 7.8 Hz)	H-8
H-11a	part of the m at 2.99-2.87	
H-11b	part of the m at 2.99-2.87	
H-12a	part of the m at 3.05-2.96.	
H-12b	part of the m at 3.05-2.96	
H-14	Overlapping with the d at 7.01 (d, *J* = 7.8 Hz)	H-15, H-16
H-15	7.54 (td, *J* = 7.8, 1.8 Hz)	H-16, H-14
H-16	7.10 (dd, *J* = 4.2, 1.8 Hz)	H-15, H-14
H-17	8.54 (d, *J* = 4.2 Hz)	H-15
H-2'a	part of the m at 2.46-2.35	
H-2'b	part of the m at 2.46-2.35	
H-3'	5.61 (dddd, *J* = 16.8, 10.2, 7.2, 7.2 Hz)	H-2'a, H-2'b, H-4'a, H-4'b
H-4'a	4.96 (d, *J* = 16.8 Hz)	H-3'
H-4'b	4.95 (d, *J* = 10.2 Hz)	H-3'

[a] Recorded at 600 MHz. [b] Assignments based on HSQC-DEPT and HMBC data
[c] Methylene protons are designated H-Xa and H-Xb arbitrarily
[d] Only those correlations which could be unambiguously assigned are reported.

2-(2-(Pyridin-4-yl)ethyl)pyridine Alkylidene Dihydropyridine Intermediate (**29'**)

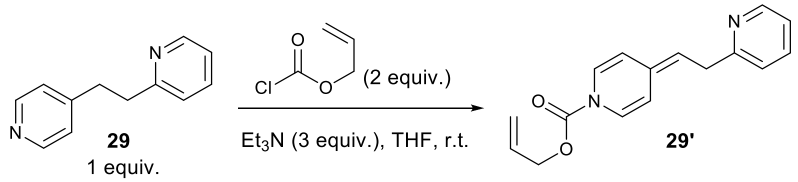

Using procedure 4, pyridine **29** (100 mg, 0.54 mmol, 1.0 equiv.) was reacted with triethylamine (0.23 mL, 1.63 mmol, 3 equiv.) and allyl chloroformate (0.12 mL, 1.09 mmol, 2 equiv.) to provide alkylidene dihydropyridine **29'** as a bright yellow oil.

Data for **29'**

^{1}H-NMR (300 MHz, $CDCl_3$, at 40°C)
δ 8.51 (d, J = 4.2 Hz, 1 H), 7.57 (td, J = 7.7, 1.8 Hz, 1 H), 7.15 (d, J = 7.8 Hz, 1 H), 7.09 (m, 1 H), 6.90 (br dd, J = 45.3, 6.3 Hz, 2 H), 6.01-5.71 (m, 3 H), 5.35 (d, J = 17.1 Hz, 1 H), 5.28 (d, J = 10.5 Hz, 1 H), 5.07 (t, J = 7.8 Hz, 1 H), 4.72 (dd, J = 5.7, 1.2 Hz, 2 H), 3.58 (d, J = 7.8 Hz, 2 H).

^{13}C-NMR (76 MHz, $CDCl_3$, at 52°C)
δ 161.3, 150.6, 149.5, 136.5, 131.8, 129.5, 124.9, 122.7, 122.5, 121.2, 119.0, 115.8, 112.4, 109.2, 67.6, 36.2.

IR Alpha-Platinum ATR, Bruker, diamond crystal
υ = 2921, 1721, 1673, 1589, 1301, 1196, 980, 967 cm^{-1}

HRMS ESI
Calculated mass for $(M+H)^+$ of $C_{16}H_{16}N_2O_2$ is 269.1285, found 269.1277.

2-Methyl-4-(1-phenylpent-4-en-2-yl)pyridine (30A)

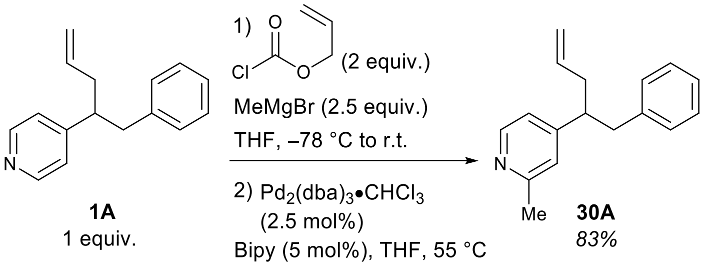

A flame-dried round bottomed flask equipped with a stir bar was charged with pyridine **1A** (0.1 g, 0.55 mmol, 1 equiv.) and dry THF (5.5 mL) then cooled to –78 °C. Allyl chloroformate was introduced dropwise and the mixture was allowed to stir for 1 hour. Methylmagnesium bromide was added dropwise. The solution was stirred for 1 hour at –78 °C and room temperature for another hour, then quenched with a saturated aqueous ammonium chloride solution. The aqueous layer was extracted three times with EtOAc and the combined organics were given a brine wash, dried with $MgSO_4$ and concentrated *in vacuo*. A solution of the crude product in THF (3 mL) was then added to a solution of $Pd_2(dba)_3$•$CHCl_3$ (14.3 mg, 0.014 mmol, 2.5 mol%) and 2,2'-bipyridine (4.3 mg, 0.028 mmol, 5 mol%) in THF (2.5 mL) then heated to 55 °C overnight. The mixture was then concentrated *in vacuo* then purified using flash chromatography to afford product **30A** (0.11 g, 0.45 mmol) in 83% yield as a colourless oil.

Chromatography: 10%-15% EtOAc in Hexanes on Et_3N deactivated silica (R_f. = 0.2).

Data for **30A**

^{1}H-NMR (300 MHz, $CDCl_3$)

δ 8.35 (d, *J* = 4 Hz, 1 H), 7.24-7.12 (m, 3 H), 7.02 (d, *J* = 10 Hz, 2 H), 6.86 (s, 1 H), 6.83 (d, *J* = 8 Hz, 1 H), 5.63 (dddd, *J* = 18, 10.5, 9, 9 Hz, 1 H), 5.0-4.949 (m, 2 H), 2.98-2.79 (m, 3 H), 2.49 (s, 3 H), 2.43-2.36 (m, 2 H).

^{13}C-NMR (76 MHz, $CDCl_3$)

δ 158.1, 153.7, 148.9, 139.5, 135.8, 129.1, 128.3, 126.2, 122.9, 120.4, 117.0, 47.2, 41.9, 39.2, 24.4.

IR Alpha-Platinum ATR, Bruker, diamond crystal

υ = 3027, 2923, 1640, 1601 cm^{-1}

HRMS DART

Calculated mass for $(M+H)^+$ of $C_{17}H_{20}N$ is 238.1590, found 238.1595.

Palladium Catalyzed Allylation using Trost Ligand L2

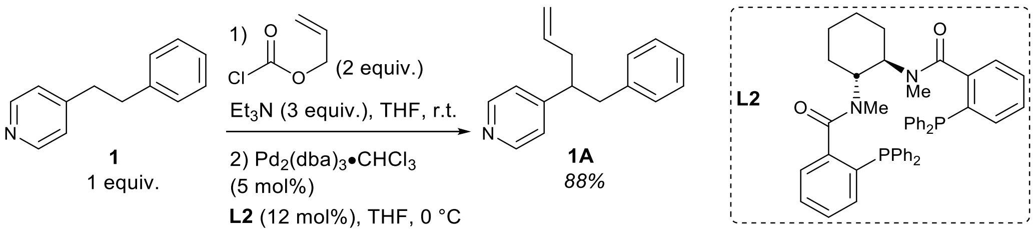

Using procedure 4, pyridine **1** (0.1 g, 0.55 mmol, 1.0 equiv.) and using 5 mol% $Pd_2(dba)_3$•$CHCl_3$ (28 mg, 0.027 mmol) and 12% Trost ligand **L2** (47 mg, 0.065 mmol) catalytic system provided product **1A** (107 mg, 0.48 mmol) in 88% yield as a yellow oil.

Chromatography: 20% EtOAc in hexane (R_f. = 0.30).

Data for **1A**

^{1}H-NMR (400 MHz, $CDCl_3$)

δ 8.45 (d, J = 5.6 Hz, 2 H), 7.24-7.13 (m, 3 H), 7.01-7.00 (m, 4 H), 5.63 (dddd, J =17.2, 10.4, 6.8, 6.8 Hz, 1 H), 4.97 (d, J = 17.2 Hz, 1 H), 4.96 (d, J = 10.4 Hz, 1 H), 2.99 (dd, J = 12.4, 6.8 Hz, 1 H), 2.97-2.89 (m, 1 H), 2.83 (dd, J = 12.4, 7.4 Hz, 1 H), 2.50-2.36 (m, 2 H).

^{13}C-NMR (101 MHz, $CDCl_3$)

δ 153.5, 149.8, 139.5, 135.8, 129.2, 128.4, 126.3, 123.5, 117.2, 47.4, 42.1, 39.4.

IR Alpha-Platinum ATR, Bruker, diamond crystal

υ = 3064, 3026, 2923, 1640, 1597, 1557, 1495, 1453, 1413, 910 cm^{-1}

HRMS ESI

Calculated mass for $(M+H)^+$ of $C_{13}H_{17}N$ is 224.1434, found 224.1424.

Cross-over Experiment.

Part A: Synthesis of Reference Compounds.

Procedure 5: General procedure for allylation of 4-alkylpyridines using methallyl chloroformate

Synthesis of the methallyl chloroformate: A flame-dried round-bottomed flask equipped with a stir bar was cooled to –40 °C and charged with phosgene solution (15% in toluene, 2.24 equiv.). A solution of methallyl alcohol (1.6 equiv.) in THF (1.4 M) was then introduced dropwise and the temperature was maintained at –40 °C for one hour, then warmed to 0 °C for 10 minutes. The solution was then purged for 20 minutes with a stream of argon into a 1M aqueous sodium hydroxide trap to quench the excess phosgene. The resulting solution of methallyl chloroformate was maintained at 0 °C and used in the next step without isolation.

Synthesis of the ADHP intermediate: Triethylamine (3.0 equiv.) was added to a solution of the appropriate pyridine (1.0 equiv.) in dry THF (0.1 M) in a flame-dried flask equipped with a stir bar. The resulting solution was cooled to 0 °C and methallyl chloroformate (1.6 equiv.) was added dropwise. The mixture was allowed to stir for 45 min then concentrated *in vacuo* (on a rotary evaporator inside a fumehood). The resulting crude mass was suspended in diethyl ether and filtered through a plug of cotton to remove the triethylammonium chloride salt. The filtrate was concentrated *in vacuo* to afford an oil which was used in the next step without further purification.

Palladium-catalyzed decarboxylative allylation: An oven-dried round-bottomed flask equipped with a stir bar was charged with Xantphos (10 mol%) and $Pd(dba)_2$ (10 mol%). Dry THF was added and the resulting solution was allowed to stir for 10 min at room temperature under an atmosphere of argon. A solution of the ADHP in dry THF was then added to the flask containing the catalyst and the mixture was stirred at room temperature overnight. The mixture was then concentrated *in vacuo* and purified using flash chromatography, eluting with the indicated solvent mixture to afford the desired product.

4-(2-Phenylethyl)pyridine coupled product 1A (CH_3)

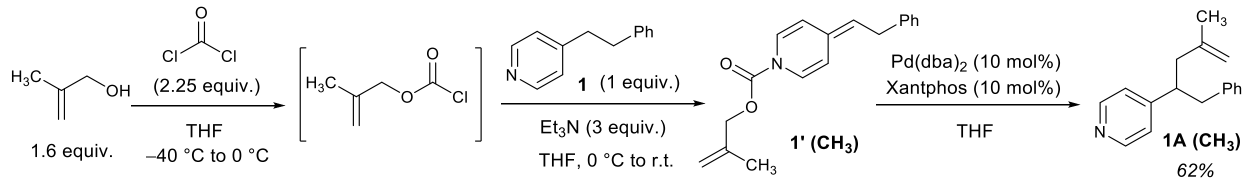

Using <u>procedure 5</u>, pyridine **1** (0.1 g, 0.55 mmol, 1.0 equiv.) provided product **1A (CH_3)** (80 mg, 0.34 mmol) in 62% yield as a colorless oil.

Chromatography: 25% EtOAc in Hexanes (R_f. = 0.28).

Data for **1A (CH_3)**

<u>^{1}H-NMR</u> (400 MHz, $CDCl_3$)

δ 8.46 (d, *J* = 6 Hz, 2 H), 7.23-7.13 (m, 3 H), 7.02-6.95 (m, 4 H), 4.73 (s, 1 H), 4.63 (s, 1 H), 3.11-2.96 (m, 2 H), 2.78 (dd, *J* = 8.8, 13.2 Hz, 1 H), 2.51-2.35 (m, 2 H), 1.59 (s, 3 H).

<u>^{13}C-NMR</u> (101 MHz, $CDCl_3$)

δ 153.7, 149.7, 142.7, 139.6, 129.1, 128.3, 126.3, 123.4, 113.1, 45.6, 43.6, 42.6, 22.4.

<u>IR</u> Alpha-Platinum ATR, Bruker, diamond crystal

υ = 3026, 2931, 1649, 1597 cm^{-1}

<u>HRMS</u> DART

Calculated mass for $(M+H)^+$ of $C_{17}H_{20}N$ is 238.1590, found 238.1590.

4-(2-Phenylethyl)pyridine coupled product 1A (CD_3)

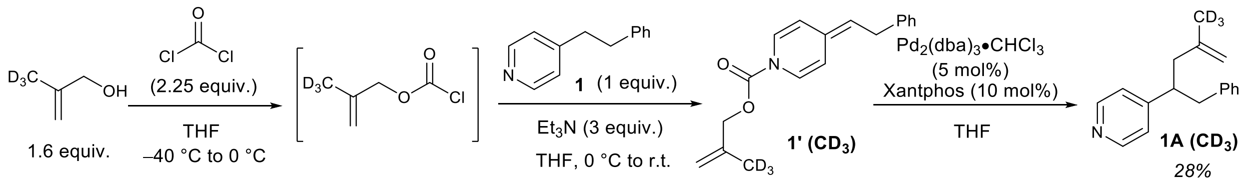

Following procedure 5 and using *d3*-methallyl alcohol (66 mg, 0.87 mmol, 1.6 equiv.), pyridine **1** (100 mg, 0.55 mmol, 1.0 equiv.) provided product **1A (CD_3)** (37.3 mg, 0.15 mmol) in 28% yield as a colorless oil.

Chromatography: 100% Hexanes – 30% EtOAc in Hexanes ($R_{f.}$ = 0.28 in 25% EtOAc in Hexanes).

Data for **1A (CD_3)**

^{1}H-NMR (400 MHz, $CDCl_3$)
δ 8.44 (d, *J* = 6 Hz, 2 H), 7.21-7.14 (m, 3 H), 7.00-6.95 (m, 4 H), 4.71 (s, 1 H), 4.61 (s, 1 H), 3.07-2.95 (m, 2 H), 2.80 (dd, *J* = 8.6, 13.4 Hz, 1 H), 2.46-2.33 (m, 2 H).

^{2}H-NMR (61 MHz, $CDCl_3$)
δ 1.62 (s, 3 D).

^{13}C-NMR (151 MHz, $CDCl_3$)
δ 153.7, 149.7, 142.7, 139.6, 129.2, 128.4, 126.3, 123.4, 113.1, 45.7, 43.6, 42.6, 21.6 (sept, *J* = 18.1 Hz).

IR Alpha-Platinum ATR, Bruker, diamond crystal
υ = 3068, 2928, 2266, 1643, 1597 cm^{-1}

HRMS DART
Calculated mass for $(M+H)^+$ of $C_{17}H_{17}D_3N$ is 241.17786, found 241.17808.

4-Pentylpyridine coupled product 1A (CD_3)

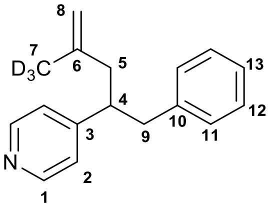

Carbon No.	^{13}C δ (ppm)[a]	^{1}H δ (ppm) (mult: *J* (Hz))[a, b]	HMBC Correlation
1	149.7	H-1: 8.44 (d, J = 6 Hz, 2 H)	H-2
2	123.4	Part of the multiplet at : 7.00-6.95 (m, 4 H)	H-1, H-4
3	153.7	--	H-1, H-4, H-5, H9
4	45.7	Part of the multiplet at: 3.07-2.95 (m, 2 H)	H-1, H-2, H-5, H-8, H-9
5	43.6	H-5: 2.46-2.33 (m, 2 H)	H4, H-8, H-9
6	142.7	--	H-4, H-5, H-8
7	21.6 (sept, *J* = 18.1 Hz)	--	H-5, H-8
8	113.1	H-8: 4.71 (s, 1 H), 4.61 (s, 1 H)	H-5
9	42.6	Part of the multiplet at: 3.07-2.95 (m, 2 H), 2.80 (dd, *J* = 8.6, 13.4 Hz, 1 H)	H-4, H-5, H-11
10	139.6	--	H-4, H-9, H-12
11	129.2	Part of the multiplet at : 7.00-6.95 (m, 4 H)	H-9, H-12, H-13
12	128.4	Part of the multiplet at: 7.21-7.14 (m, 3 H)	H-13
13	126.3	Part of the multiplet at: 7.21-7.14 (m, 3 H)	H-11, H-12

[a] Recorded at 151 MHz.

[b] Only those correlations which could be unambiguously assigned are reported.

4-Pentylpyridine coupled product 2A (CH_3)

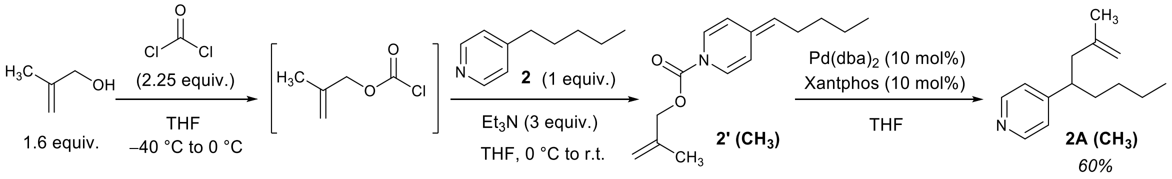

Using procedure 5, pyridine **2** (0.1 g, 0.67 mmol, 1.0 equiv.) provided product **2A (CH_3)** (82 mg, 0.40 mmol) in 60% yield as a colorless oil.

Chromatography: 13% EtOAc in Hexanes (R_f. = 0.31).

Data for **2A (CH_3)**

^{1}H-NMR (400 MHz, $CDCl_3$)

δ 8.49 (d, J = 6 Hz, 2 H), 7.07 (d, J = 6 Hz, 2 H), 4.66 (s, 1 H), 4.55 (s, 1 H), 2.69 (m, 1 H), 2.36-2.21 (m, 2 H), 1.65-1.45 (m, 5 H), 1.35-1.05 (m, 4 H), 0.82 (t, J = 6.9 Hz, 3 H).

^{13}C-NMR (101 MHz, $CDCl_3$)

δ 154.9, 149.8, 143.1, 123.4, 112.7, 44.9, 43.6, 35.5, 29.7, 22.8, 22.5, 14.1.

IR Alpha-Platinum ATR, Bruker, diamond crystal

υ = 3070, 2929, 1598 cm^{-1}

HRMS DART

Calculated mass for $(M+H)^+$ of $C_{14}H_{22}N$ is 204.1747, found 204.1748.

4-Pentylpyridine coupled product 2A (CD_3)

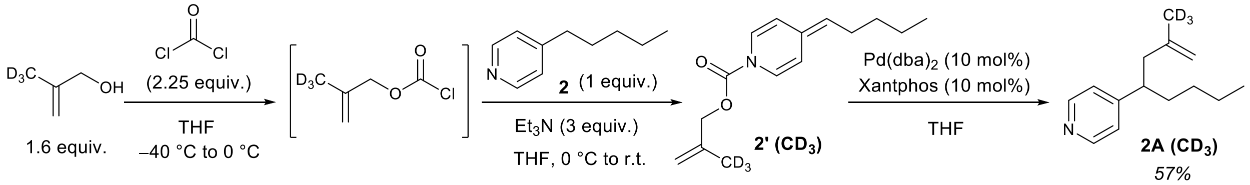

Following procedure 5 and using *d3*-methallyl alcohol (80.6 mg, 1.07 mmol, 1.6 equiv.), pyridine **2** (100 mg, 0.67 mmol, 1.0 equiv.) provided product **2A (CD_3)** (79 mg, 0.38 mmol) in 57% yield as a colorless oil.

Chromatography: 15% EtOAc in Hexanes ($R_{f.}$ = 0.35).

Data for **2A (CD_3)**

^{1}H-NMR (300 MHz, $CDCl_3$)
δ 8.49 (d, *J* = 6 Hz, 2 H), 7.07 (d, *J* = 6 Hz, 2 H), 4.70 (d, *J* = 21.3 Hz, 1 H), 4.52 (d, *J* = 21.3 Hz, 1 H), 2.70 (m, 1 H), 2.39-2.23 (m, 2 H), 1.70-1.41 (m, 2 H), 1.35-1.05 (m, 4 H), 0.82 (t, *J* = 6.9 Hz, 3 H).

^{2}H-NMR (46 MHz, $CDCl_3$)
δ 1.62 (s, 3 D).

^{13}C-NMR (151 MHz, $CDCl_3$)
δ 154.9, 149.8, 143.0, 123.3, 112.7, 44.8, 43.6, 35.5, 29.7, 22.7, 21.8 (sept, *J* = 19.6 Hz), 14.0.

IR Alpha-Platinum ATR, Bruker, diamond crystal
υ = 3071, 2929, 2226, 1597 cm^{-1}

HRMS DART
Calculated mass for $(M+H)^+$ of $C_{14}H_{19}D_3N$ is 207.1935, found 207.1940.

4-Pentylpyridine coupled product 2A (CD_3)

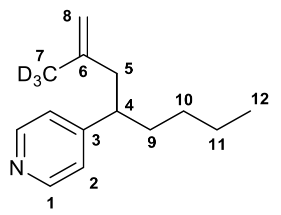

Carbon No.	^{13}C δ (ppm)[a]	^{1}H δ (ppm) (mult: *J* (Hz))[a, b]	HMBC Correlation
1	149.8	H-1: 8.49 (d, *J* = 6 Hz, 2 H)	H-2
2	123.3	H-2: 7.07 (d, *J* = 6 Hz, 2 H)	H-1, H-4
3	154.9	--	H-4, H-5, H9
4	43.6	2.70 (m, 1 H)	H-1, H-2, H-5, H-5
5	44.8	2.39-2.23 (m, 2 H)	H4, H-8
6	143.0	--	H-4, H-5, H-8
7	21.8 (sept, *J* = 19.6 Hz)	--	H-5, H-8
8	112.7	4.70 (d, *J* = 21.3 Hz, 1 H),<br>4.52 (d, *J* = 21.3 Hz, 1 H)	H-5
9	35.5	1.70-1.41 (m, 2 H)	H-4, H-5, H-10, H-11
10	29.7	Part of the multiplet at: 1.35-1.05 (m, 4 H)	H-4, H-9, H-11, H-12
11	22.7	Part of the multiplet at: 1.35-1.05 (m, 4 H)	H-9, H-10, H-12
12	14.0	0.82 (t, *J* = 6.9 Hz, 3 H)	H-10, H-11

[a] Recorded at 151 MHz.

[b] Only those correlations which could be unambiguously assigned are reported.

Cross-Over Experiment.

Part B: Cross-Over Experiment Procedure and Analysis.

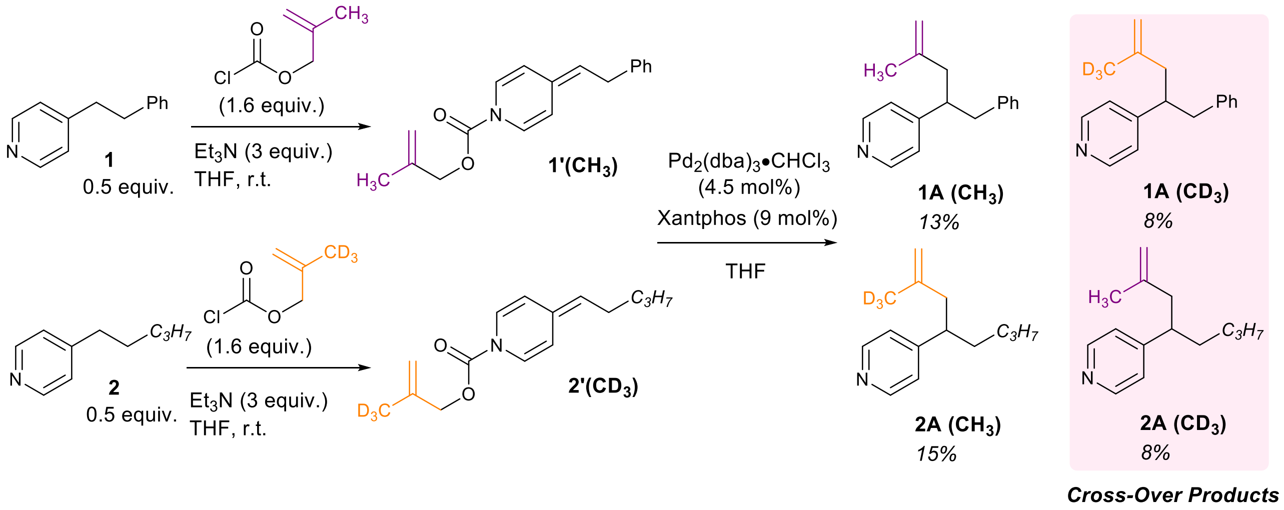

*ADHP **1'(CH₃)** Formation* – Using procedure 5, pyridine **1** (123 mg, 0.67 mmol, 1.0 equiv.) was reacted with triethylamine (0.28 mL, 2.01 mmol, 3 equiv.) and methallyl chloroformate (1.07 mmol, 1.6 equiv.) to provide alkylidene dihydropyridine **1'(CH_3)** as a bright yellow oil.

*ADHP **2'(CD_3)** Formation* – Using procedure 5, pyridine **2** (100 mg, 0.67 mmol, 1.0 equiv.) was reacted with triethylamine (0.28 mL, 2.01 mmol, 3 equiv.) and *d3*-methallyl chloroformate (1.07 mmol, 1.6 equiv.) to provide alkylidene dihydropyridine **2'(CD_3)** as a pink oil.

Cross-Over Experiment – A flame-dried round bottomed flask equipped with a stir bar was charged with $Pd_2(dba)_3 \cdot CHCl_3$ (64 mg, 0.067 mmol, 4.5 mol%), Xantphos (78 mg, 0.134 mmol, 10 mol%) and dry THF (6 mL) and the mixture was stirred for 15 min. ADHP intermediates **1'(CH_3)** and **2'(CD_3)** were then added as a solution in THF (7 mL) and the mixture was left to stir overnight. The solution was then concentrated *in vacuo* and using flash chromatography two sets of products were separated: (**1A (CH_3)** + **1A (CD_3)** and (**2A (CH_3)** + **2A (CD_3)**). The ratio of deuterated to methylated products was determined using quantitative ^{1}H NMR for **1A (CH_3) + 1A (CD_3)** using the CH_3 peak of **1A (CH_3)** as the internal standard and quantitative ^{2}H NMR for **2A (CH_3) + 2A (CD_3)** using CD_3CN as an internal standard.

Determining Product Ratio – The product set **1A (CH_3)** + **1A (CD_3)** was isolated with a total mass of 64.6 mg (0.27 mmol). ^{1}H NMR was used to determine the molar ratio of the products; the CH_3 singlet of **1A (CH_3)** at 1.68 ppm was calibrated to 3 H and compared to the total integration of the multiplet at 6.92 ppm (which accounts for 2 phenyl protons and 2 pyridine protons for each of the compounds). This established the product ratio to be 1 : 0.69 of **1A (CH_3)** : **1A (CD_3)**; **1A (CH_3)** (37.9 mg, 0.16 mmol) and **1A (CD_3)** (26.5 mg, 0.11 mmol).

The product set **2A (CH_3)** + **2A (CD_3)** was isolated with a total mass of 63.8 mg (0.21 mmol). Quantitative ^{2}H NMR was performed using known amounts of CD_3CN as an internal standard which revealed the mixture contains 40.9 mg (0.20 mmol) of **2A (CD_3)**. By considering the total isolated mass and the mass of **2A (CD_3)** obtained through quantitative ^{2}H NMR, it was deduced that **2A (CH_3)** was present in 22.9 mg (0.11 mmol). The products **2A (CH_3)** + **2A (CD_3)**, therefore, existed in a 1 : 0.41 molar ratio.

Stereochemical Probe.

4-Isopropylpyridine coupled product (31A)

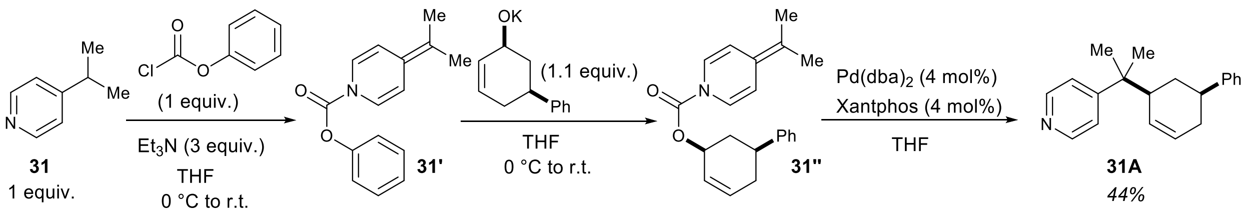

<u>*ADHP **31'** Formation*</u> – Using <u>procedure 4</u>, pyridine **31** (340mg, 2.80 mmol, 1.0 equiv.) was reacted with triethylamine (1.17 mL, 8.40 mmol, 3 equiv.) and phenyl chloroformate (438mg, 2.80 mmol, 1.0 equiv.) to provide ADHP **31'** as a white solid.

<u>*ADHP **31"** Formation*</u> – A flame-dried round bottomed flask equipped with a stir bar was charged with *cis*-5-phenyl-2-cyclohexen-1-ol (520 mg, 3.0 mmol, 1.1 equiv.) and dry THF (30 mL) and cooled to 0°C under an argon atmosphere. KHMDS (6.2 mL, 3.3 mmol, 1.1 equiv., 0.50 M in toluene) was then added dropwise and the mixture was allowed to stir at 0°C for 30 minutes. This potassium alkoxide was added dropwise to another flame-dried flask containing a solution of intermediate **24'** in dry THF (27 mL) at 0°C and the mixture was allowed to warm to room temperature over 1.5 hours. The solution was then *in vacuo* and the resulting crude mass was suspended in toluene and filtered through a plug of cotton to remove the phenyl alkoxide salt. The filtrate was concentrated *in vacuo* to afford ADHP **31"** as a yellow oil which was used in the next step without further purification.

Palladium-catalyzed decarboxylative allyation – Using procedure 4, ADHP **31”** was mixed with Xantphos (4 mol%) and $Pd(dba)_2$ (4 mol%) in dry THF (27 mL) and allowed to stir at room temperature overnight. This afforded product **31A** (70 mg, 1.23 mmol) as a yellow solid in 44% yield.

Chromatography: 25% EtOAc in Hexane (R_f = 0.30).

Data for **31A**

^{1}H-NMR (600 MHz, $CDCl_3$)
δ 8.50 (d, *J* = 6.0 Hz, 2 H), 7.28 (t, *J* = 7.5 Hz, 2 H), 7.25 (d, *J* = 4.7 Hz, 2 H), 7.19-7.15 (m, 3 H), 5.81-5.77 (m, 1 H), 5.48 (d, *J* = 10.4 Hz, 1 H), 2.83-2.77 (m, 1 H), 2.76-2.64 (m, 1 H), 2.19-2.03 (m, 2 H), 1.69-1.68 (m, 1 H), 1.43 (q, *J* = 12.4 Hz, 1 H), 1.30 (s, 3 H), 1.26 (s, 3 H).

^{13}C-NMR (151 MHz, $CDCl_3$)
δ 158.5, 149.6, 146.8, 128.7, 128.4, 127.8, 126.8, 126.2, 121.6, 47.4, 40.8, 40.4, 33.6, 32.1, 24.8, 24.2.

IR Alpha-Platinum ATR, Bruker, diamond crystal
υ = 2893, 1592, 1442, 1148, 822, 760, 699, 578 cm^{-1}

HRMS DART
Calculated mass for $(M+H)^+$ of $C_{20}H_{24}N$ is 278.1903, found 278.1910.

M.P. 103.8 – 104.6 °C

4-Isopropylpyridine coupled product (31A)

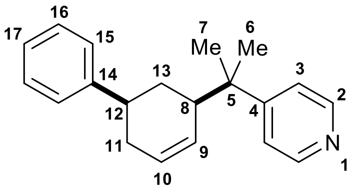

Proton No.	^{1}H δ (ppm) (mult: *J* (Hz))[a, b,c]	COSY Correlation
H-2	8.50 (d, *J* = 6.0 Hz, 2H)	H-3
H-3	7.25 (d, *J* = 4.7 Hz, 2H)	H-2
H-6	1.30 (s, 1H)	H-7
H-7	1.26 (s, 1H)	H-6
H-8	2.76-2.64 (m, 1H)	H-10, H-11, H-13a, H-13b
H-9	5.48 (d, *J* = 10.4 Hz, 1H)	H-10
H-10	5.81-5.77 (m, 1H)	H-9, H-11
H-11	2.19-2.03 (m, 2H)	H-10, H-12
H-12	2.83-2.77 (m, 1H)	H-11, H-13b
H-13	H-13a : 1.69-1.68 (m, 1H)<br>H-13b : 1.43 (q, *J* = 12.4 Hz, 1H)	H-8
H-15	part of the multiplet at: 7.19-7.15 (m, 3 H)	H-17, H-16
H-16	7.28 (t, *J* = 7.5 Hz, 2H)	H-17, H-15
H-17	Part of the multiplet at: 7.19-7.15 (m, 3 H)	H-16, H-15

[a] Recorded at 600 MHz. [b] Methylene protons are designated H-Xa and H-Xb arbitrarily

[c] Only those correlations which could be unambiguously assigned are reported.

4-Isopropylpyridine coupled product 31A

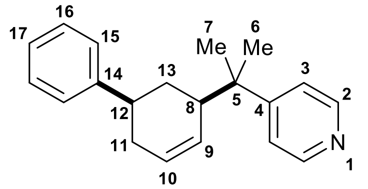

Carbon No.	^{13}C δ (ppm)[a]	^{1}H δ (ppm) (mult: *J* (Hz))[a, b,c,d]	HMBC Correlation
2	149.6	H-2 : 8.50 (d, *J* = 6.0 Hz, 2H)	H-3
3	121.6	H-3 : 7.25 (d, 4.7 Hz, 2H)	H-16, H-15, H-6, H-7
4	158.5	--	H-2, H-6, H-7
5	40.4	--	H-2, H-3, H-6, H-7
6	24.2	H-6 : 1.30 (s, 1H)	H-7
7	24.8	H-7 : 1.26 (s, 1H)	H-6
8	47.4	H-8 : 2.76-2.64 (m, 1H)	H-10, H-12, H-13a, H-13b, H-6, H-7
9	127.8	H-9 : 5.48 (d, *J* = 10.4 Hz, 1H)	H-13a, H-13b
10	128.7	H-10 : 5.81-5.77 (m, 1H)	H-11
11	33.6	H-11 : 2.19-2.03 (m, 2H)	H-10, H-12, H-13a, H-13b
12	40.8	H-12 : 2.83-2.77 (m, 1H)	H-15, H-10, H-8, H-11, H-13a, H-13b
13	29.7	H-13a : 1.69-1.68 (m, 1H)<br>H-13b : 1.43 (q, *J* = 12.4 Hz, 1H)	H-9, H-12, H-11
14	146.8	--	H-16, H-15, H-12, H-13a, H-13b
15	126.8	Part of the multiplet at: 7.19-7.15 (m, 3 H)	H-16, H-17, H-12
16	128.4	H-16: 7.28 (t, *J* = 7.5 Hz, 2H)	H-17
17	126.2	Part of the multiplet at: 7.19-7.15 (m, 3 H)	H-16, H-15

[a] Recorded at 600 MHz. [b] Assignments based on HSQC-DEPT and HMBC data

[c] Methylene protons are designated H-Xa and H-Xb arbitrarily

[d] Only those correlations which could be unambiguously assigned are reported.

4-Isopropylpyridine coupled product 31A

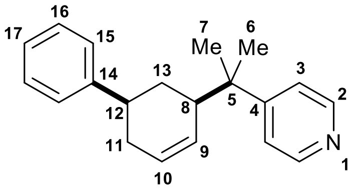

Proton No.	^{1}H δ (ppm) (mult: *J* (Hz))[a, b,c,d]	NOESY Correlation
H-3	7.25 (d, *J* = 4.7 Hz, 2H)	H-6, H-7, H-8
H-6	1.30 (s, 1H)	H-8, H-3, H-9
H-7	1.26 (s, 1H)	H-8, H-3, H-9
H-8	2.76-2.64 (m, 1H)	H-6, H-7, H-13a, H-13b
H-9	5.48 (d, *J* = 10.4 Hz, 1H)	H-6, H-7
H-10	5.81-5.77 (m, 1H)	H-11
H-11	2.19-2.03 (m, 2H)	H-10, H-12, H-15
H-12	2.83-2.77 (m, 1H)	H-13a, H-13b, H-11
H-13	H-13a : 1.69-1.68 (m, 1H)<br>H-13b : 1.43 (q, *J* = 12.4 Hz, 1H)	H-8, H-15
H-15	Part of the multiplet at: 7.19-7.15 (m, 3 H)	H-13, H-11, H-12

[a] Recorded at 600 MHz. [b] Assignments based on HSQC and HMBC data

[c] Methylene protons are designated H-Xa and H-Xb arbitrarily

[d] Only those correlations which could be unambiguously assigned are reported.

Stoichiometric Allylation Using Complex 33.

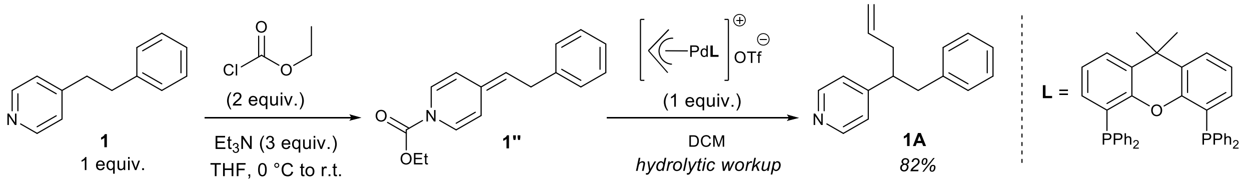

Using procedure 4, pyridine **1** (10.5 mg, 0.06 mmol, 1.0 equiv.) was reacted with triethylamine (24 *µ*L, 0.17 mmol, 3 equiv.) and ethyl chloroformate (11 *µ*L, 0.12 mmol, 2 equiv.) to provide alkylidene dihydropyridine **29'** as a bright yellow oil which was used without further purification. A 5 mL oven-dried scintillation vial was brought inside the glovebox and charged with Complex 33 (48 mg, 0.06 mmol, 1 equiv.) and then brought out and kept under an inert atmosphere of argon. The complex was then dissolved in DCM (0.7 mL) and to it was added alkylidene dihydropyridine **1''** in DCM (0.5 mL). After an overnight reaction, the mixture was concentrated *in vacuo* then stirred in aqueous saturated sodium bicarbonate. The aqueous solution was extracted with EtOAc (3x) and the combined organics were washed with brine, dried over $MgSO_4$ and concentrated *in vacuo*. The yield of the product was determined using quantitative ^1H NMR using 1,4-bis(trichloromethyl)benzene as an internal standard.

Catalytic Allylation Using Complex 33.

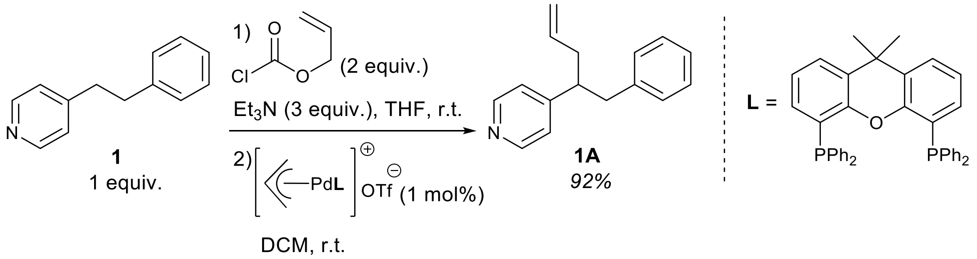

Using procedure 4 and using Complex **33** as catalyst (4.8 mg, 0.06 mmol, 1 mol%), pyridine **1** (100 mg, 0.55 mmol, 1.0 equiv.) provided product **1A** (113 mg, 0.51 mmol) in 92% yield as a clear oil.

Chromatography: 30% EtOAc in hexane (R_f. = 0.30).

Data for **1A**

^{1}H-NMR (400 MHz, $CDCl_3$)

δ 8.45 (d, *J* = 5.6 Hz, 2 H), 7.24-7.13 (m, 3 H), 7.01-7.00 (m, 4 H), 5.63 (dddd, *J* =17.2, 10.4, 6.8, 6.8 Hz, 1 H), 4.97 (d, *J* = 17.2 Hz, 1 H), 4.96 (d, *J* = 10.4 Hz, 1 H), 2.99 (dd, *J* = 12.4, 6.8 Hz, 1 H), 2.97-2.89 (m, 1 H), 2.83 (dd, *J* = 12.4, 7.4 Hz, 1 H), 2.50-2.36 (m, 2 H).

^{13}C-NMR (101 MHz, $CDCl_3$)

δ 153.5, 149.8, 139.5, 135.8, 129.2, 128.4, 126.3, 123.5, 117.2, 47.4, 42.1, 39.4.

IR Alpha-Platinum ATR, Bruker, diamond crystal

υ = 3064, 3026, 2923, 1640, 1597, 1557, 1495, 1453, 1413, 910 cm^{-1}

HRMS ESI

Calculated mass for $(M+H)^+$ of $C_{13}H_{17}N$ is 224.1434, found 224.1424.

Competition cross-over experiment between neutral ADHP 1" and a pyridylic anion of 11.

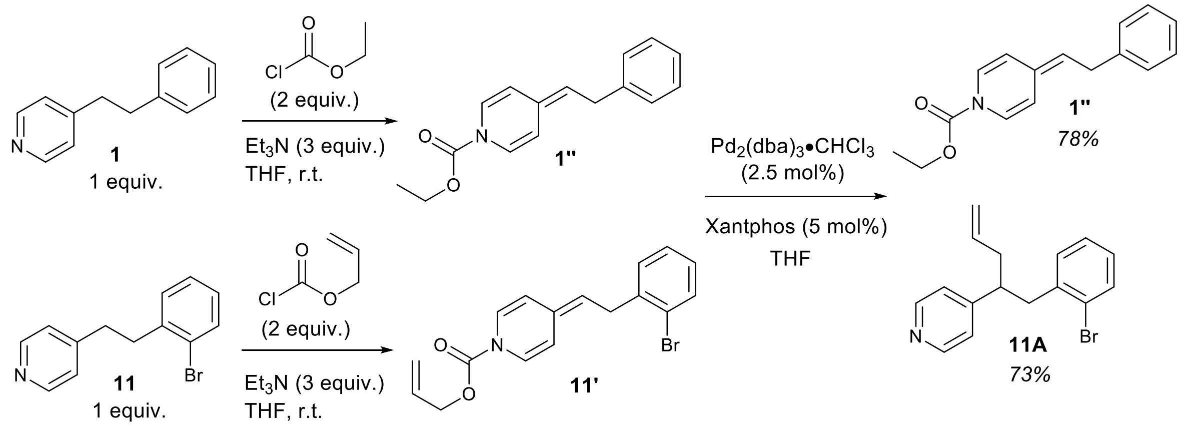

ADHP 1" Formation – Using procedure 4, pyridine **1** (150 mg, 0.82 mmol, 1.0 equiv.) was reacted with triethylamine (0.34 mL, 2.46 mmol, 3 equiv.) and ethyl chloroformate (0.16 mL, 1.64 mmol, 2 equiv.) to provide alkylidene dihydropyridine **1"** as a bright yellow oil which was used without further purification.

ADHP 11' Formation – Using procedure 4, pyridine **11** (215 mg, 0.82 mmol, 1.0 equiv.) was reacted with triethylamine (0.34 mL, 2.46 mmol, 3 equiv.) and allyl chloroformate (0.17 mL, 1.64 mmol, 2 equiv.) to provide ADHP **11'** as a bright yellow oil which was used without further purification.

Cross-Over Experiment – A flame-dried round bottomed flask equipped with a stir bar was charged with $Pd_2(dba)_3$•$CHCl_3$ (21.2 mg, 0.02 mmol, 2.5 mol%), Xantphos (23.7 mg, 0.04 mmol, 5 mol%) and dry THF (3 mL) and the mixture was stirred for 15 min. ADHP intermediates **1"** and **11'** were then added as a solution in THF (7 mL) and the mixture was left to stir overnight. The solution was then diluted with EtOAc and washed with a saturated aqueous solution of sodium bicarbonate, the aqueous layer was extracted twice more with EtOAc and the combined organics were washed with brine, dried over $MgSO_4$ and concentrated *in vacuo*. Product yield and ADHP recovery were determined using quantitative ^{1}H NMR using 1,4-bis(trichloromethyl)benzene as an internal standard.

NMR Reaction Progress Experiments.

*ADHP **1'** Formation* – Using procedure 4, pyridine **1** (50 mg, 0.27 mmol, 1.0 equiv.) was reacted with triethylamine (0.11 mL, 0.82 mmol, 3 equiv.) and allyl chloroformate (0.06 mL, 0.55 mmol, 2 equiv.) to provide alkylidene dihydropyridine **1'** as a bright yellow oil which was used without further purification.

Preparation of Pd-Xantphos Stock Solution – A flame-dried 5mL scintillation vial was charged with $Pd_2(dba)_3$•$CHCl_3$ (5.2 mg, 0.005 mmol), Xantphos (9 mg, 0.016 mmol) and CD_2Cl_2 (3.90 mL) and the solution was stirred for 15 min before use.

*Preparation of Complex **33** Stock Solution* – A flame-dried 5 mL scintillation vial was brought inside the glovebox and charged with complex 33 (8.4 mg, 0.01 mmol) then brought outside and kept under an atmosphere of argon. CD_2Cl_2 (3.68 mL) was then added and the solution was stirred for 15 min before use.

NMR Reaction Progress Experiment – An oven-dried NMR tube was charged with AHDP 1' (14.4 mg, 0.05 mmol) in CD_2Cl_2 (0.79 mL). 0.21 mL of the appropriate complex was introduced and the mixture was quickly mixed and immediately placed in the NMR. The NMR tube was spun inside the probe throughout the experiment to ensure proper mixing of the sample. The reaction was monitored for approximately 6.5 hours, recording a scan every minute with relaxation delay set to 60 seconds. The collected scans were then analyzed using Bruker Dynamics Center 2.5.5 to obtain the decay of the intermediate and rise of the allylated product.

NMR Reaction Progress Experiment – Pd-Xantphos Catalytic System

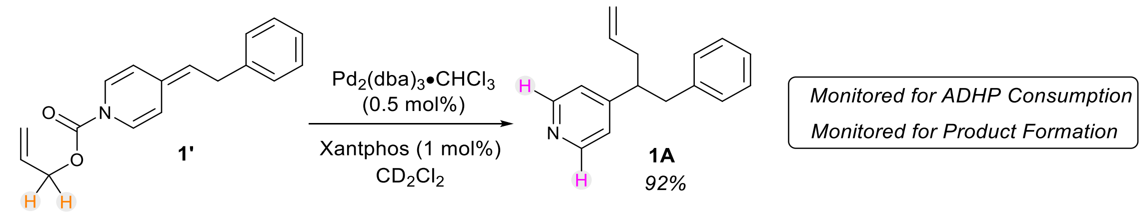

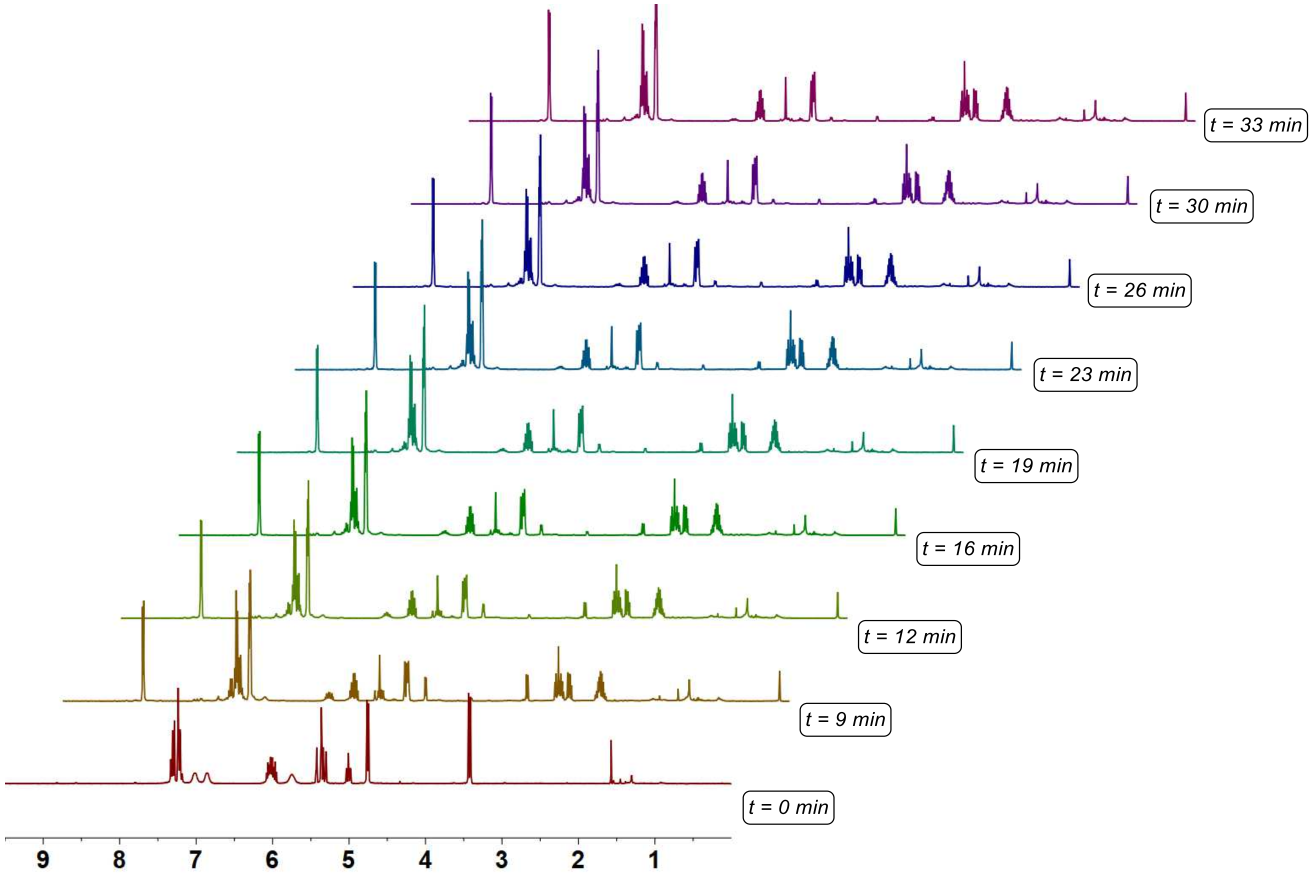

Note: Product yield was determined by quantitative ^{1}H NMR after reaction completion using1,4-bis(trichloromethyl)benzene as an internal standard.

NMR reaction progress experiment – [(Xantphos)Pd(η3-allyl)]OTf Catalytic System

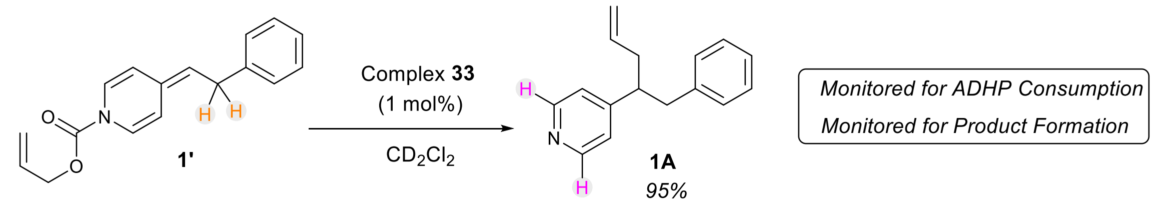

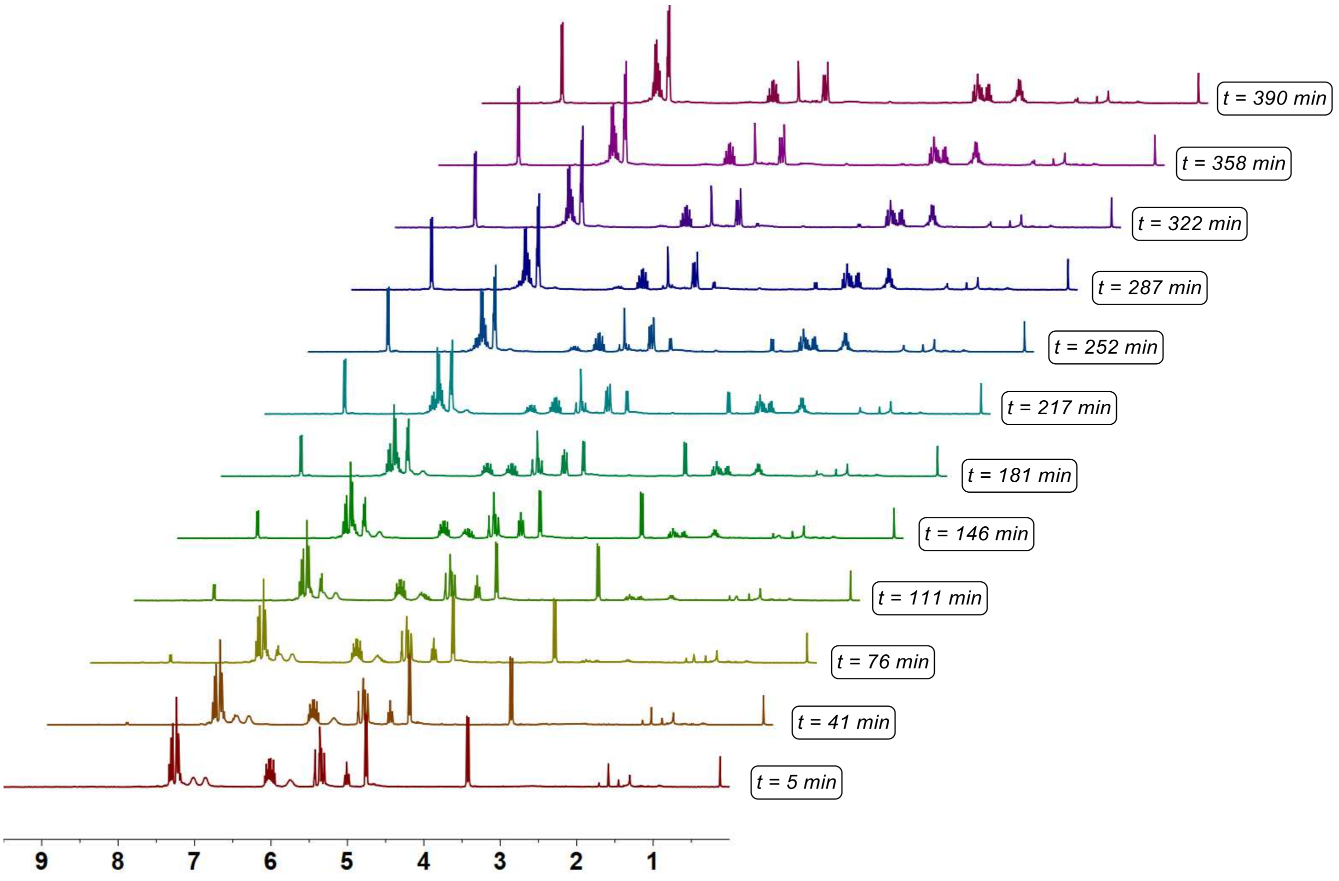

Note: Product yield was determined by using the integration values of the ADHP in the first scan as an internal standard.

Comparison of the reaction progress profiles for the Allylation of **1’** Using Pd-Xantphos and [(Xantphos)Pd(η3-allyl)]OTf (**33**) Catalytic Systems

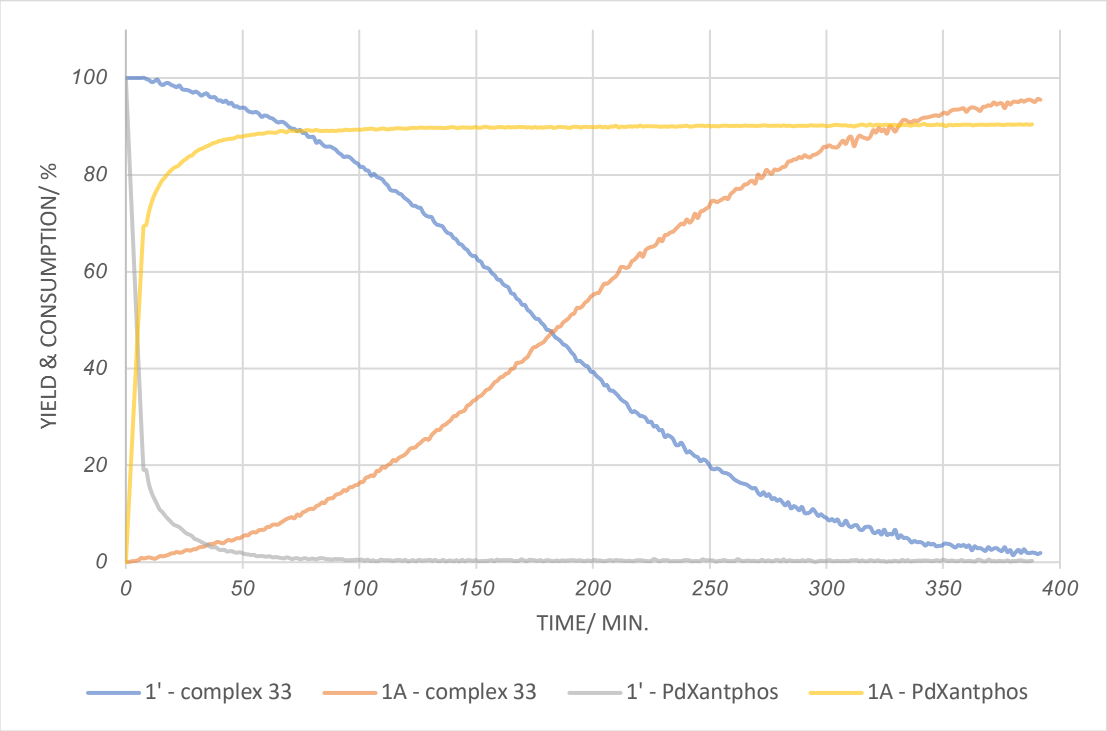

^{1}H (400 MHz, $CDCl_3$) and ^{13}C (76 MHz, $CDCl_3$) – NMR spectra of **1**

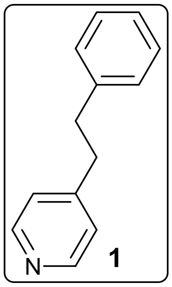

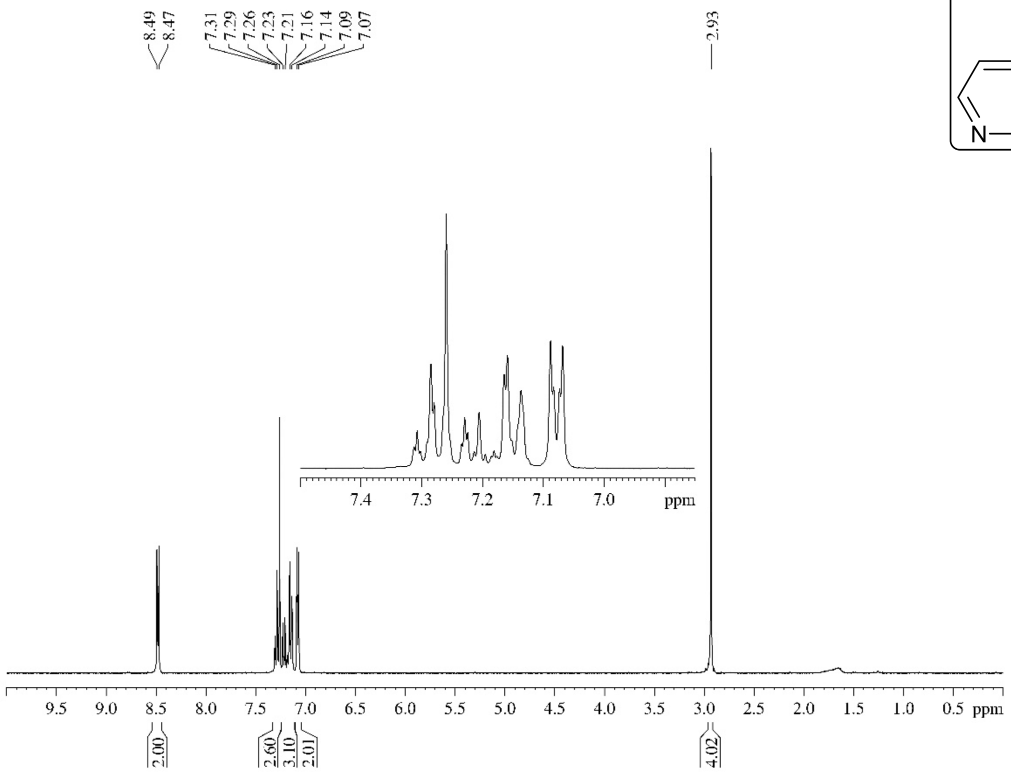

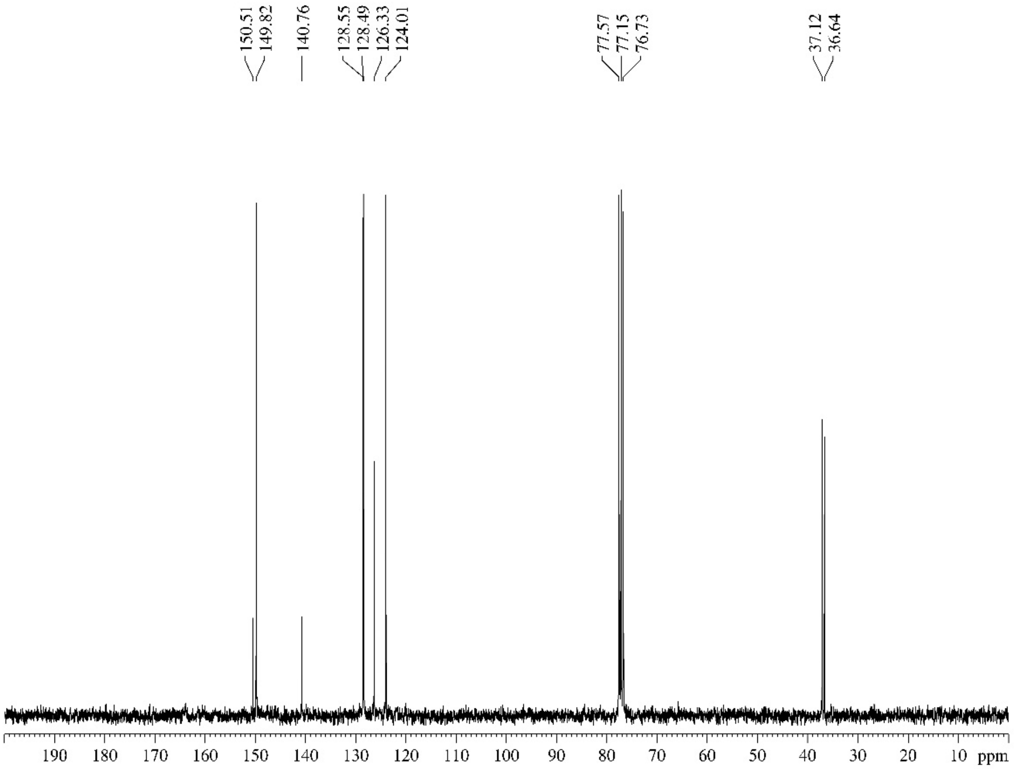

^{1}H (400 MHz, $CDCl_3$) and ^{13}C (101 MHz, $CDCl_3$) – NMR spectra of **2**

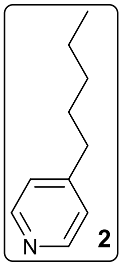

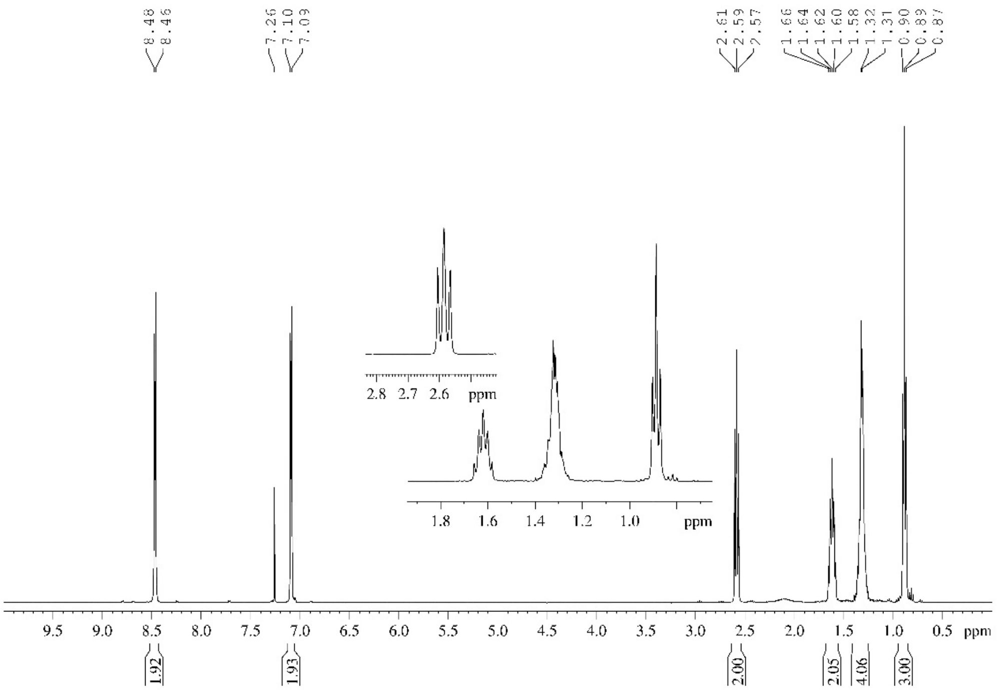

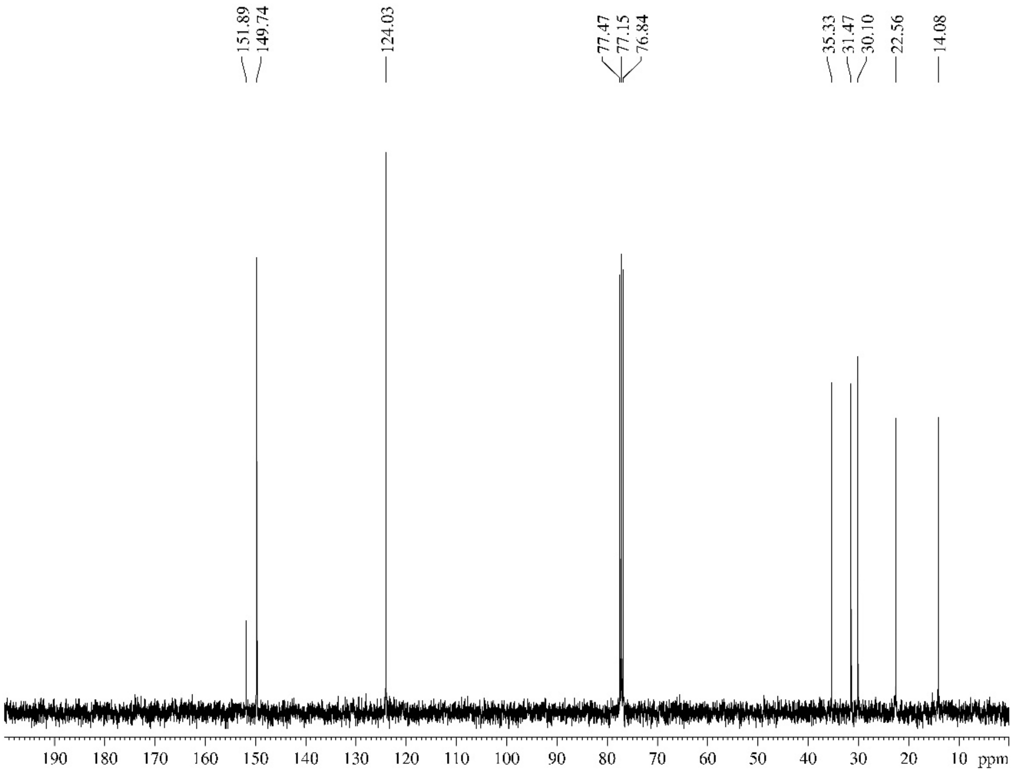

^{1}H (400 MHz, $CDCl_3$) and ^{13}C (101 MHz, $CDCl_3$) – NMR spectra of **4**

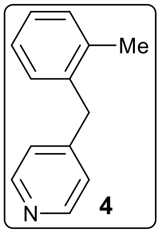

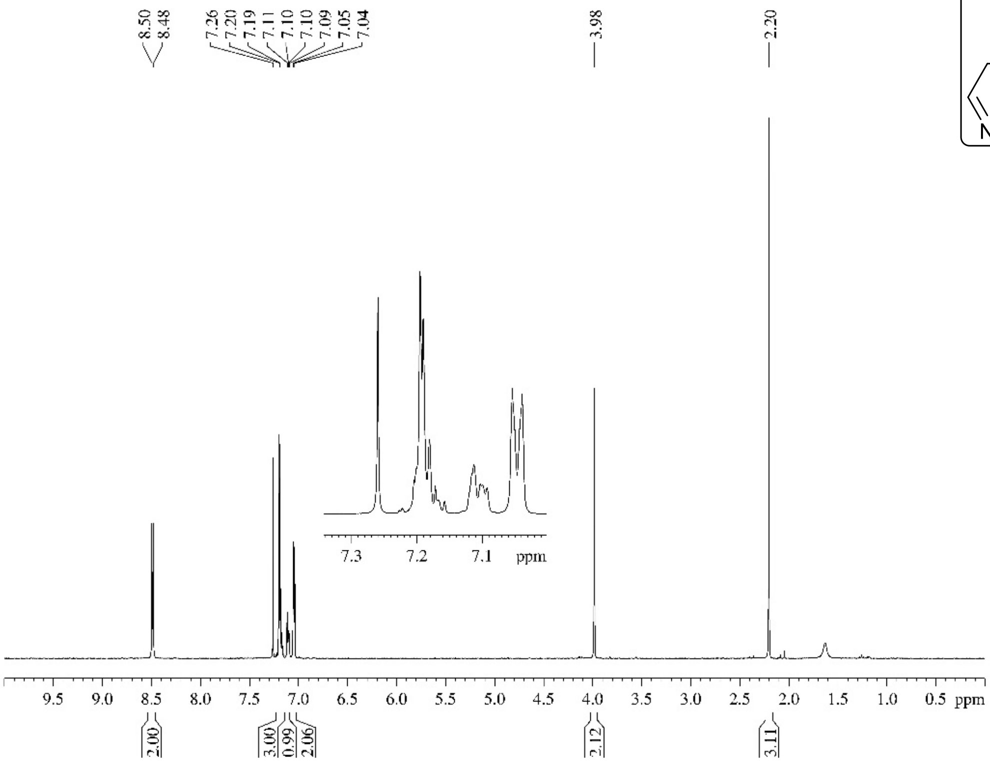

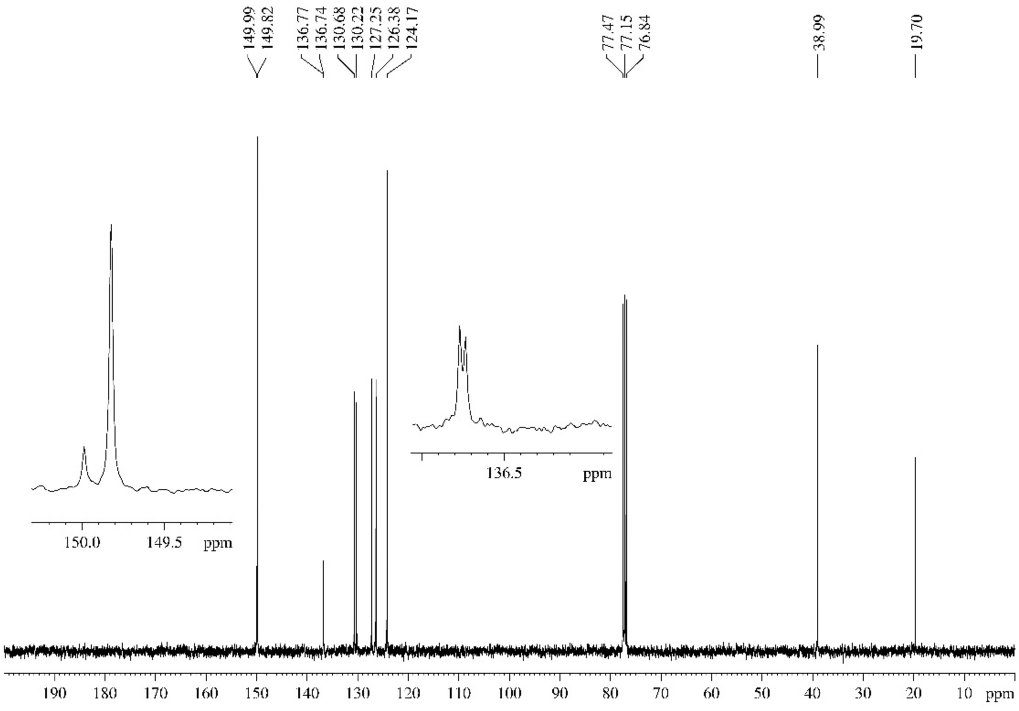

^{1}H (300 MHz, $CDCl_3$) and ^{13}C (76 MHz, $CDCl_3$) – NMR spectra of **5**

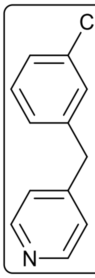

5

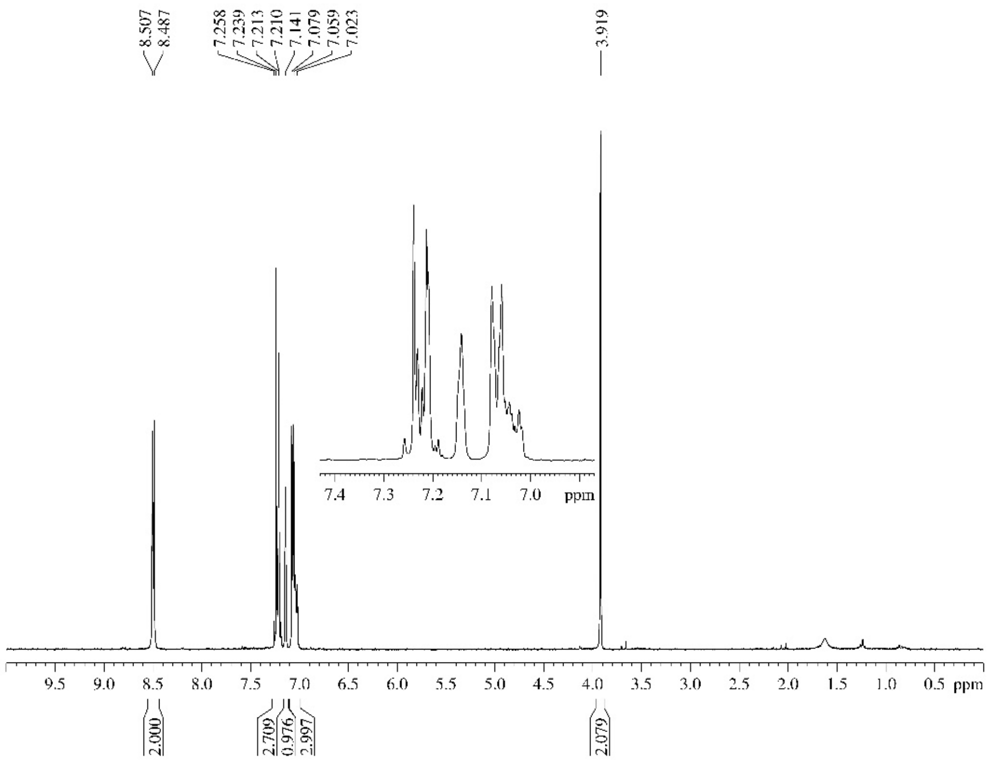

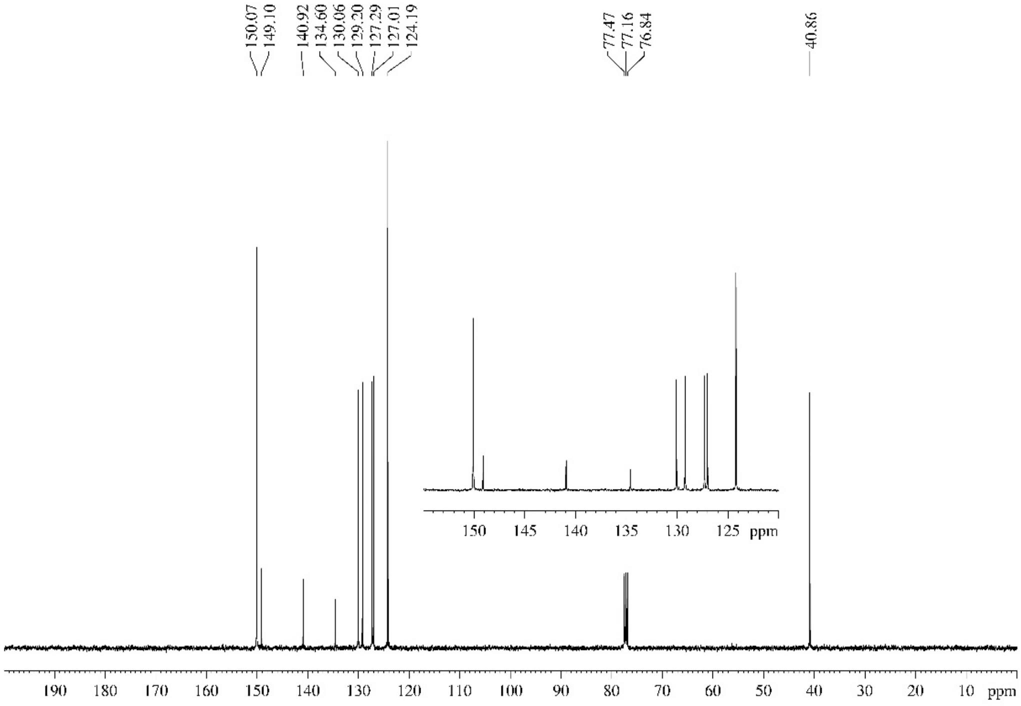

^{1}H (400 MHz, $CDCl_3$) and ^{13}C (101 MHz, $CDCl_3$) – NMR spectra of **6**

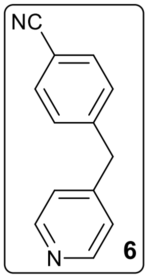

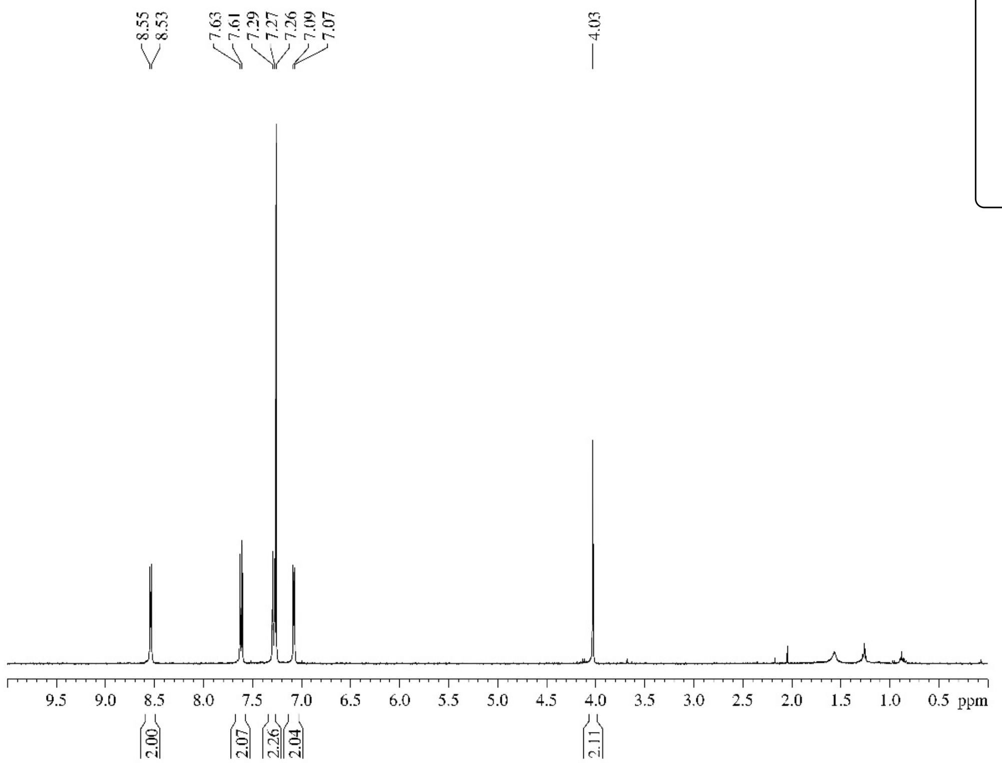

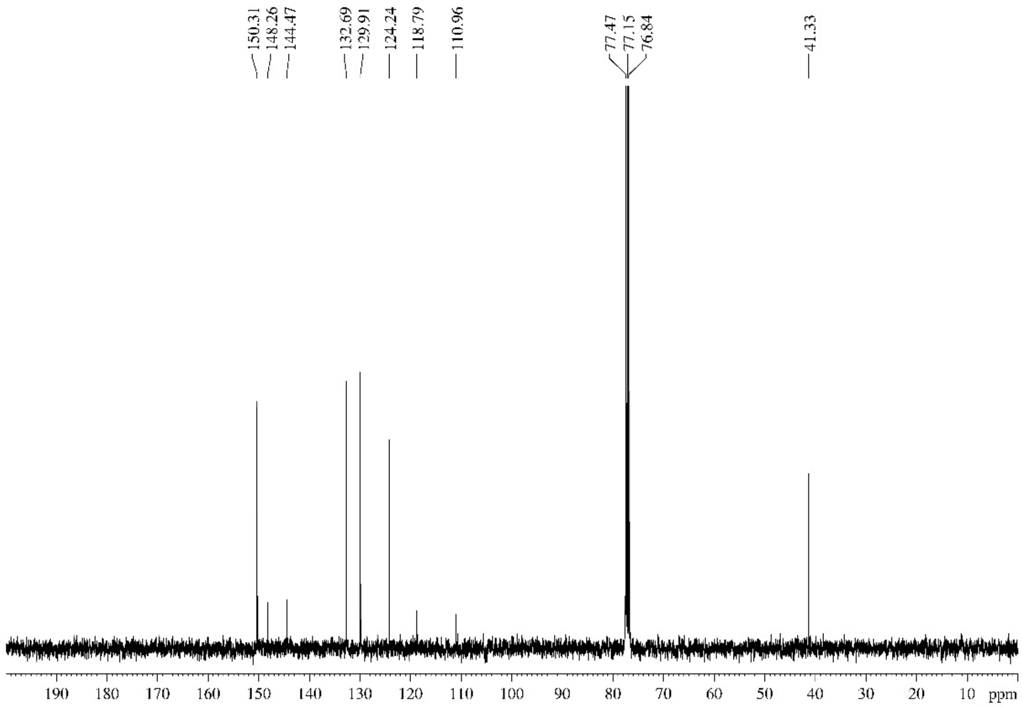

^{1}H (400 MHz, CDCl$_3$) and ^{13}C (101 MHz, CDCl$_3$) – NMR spectra of 7

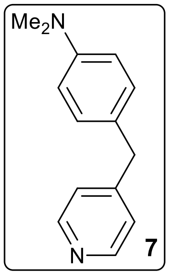

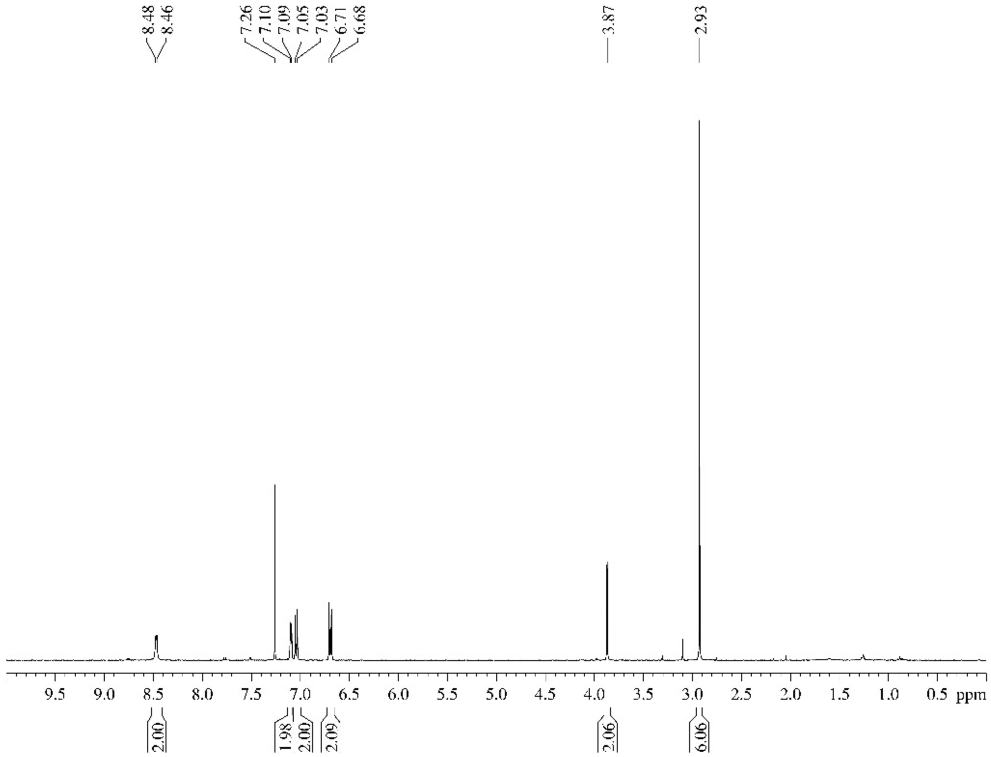

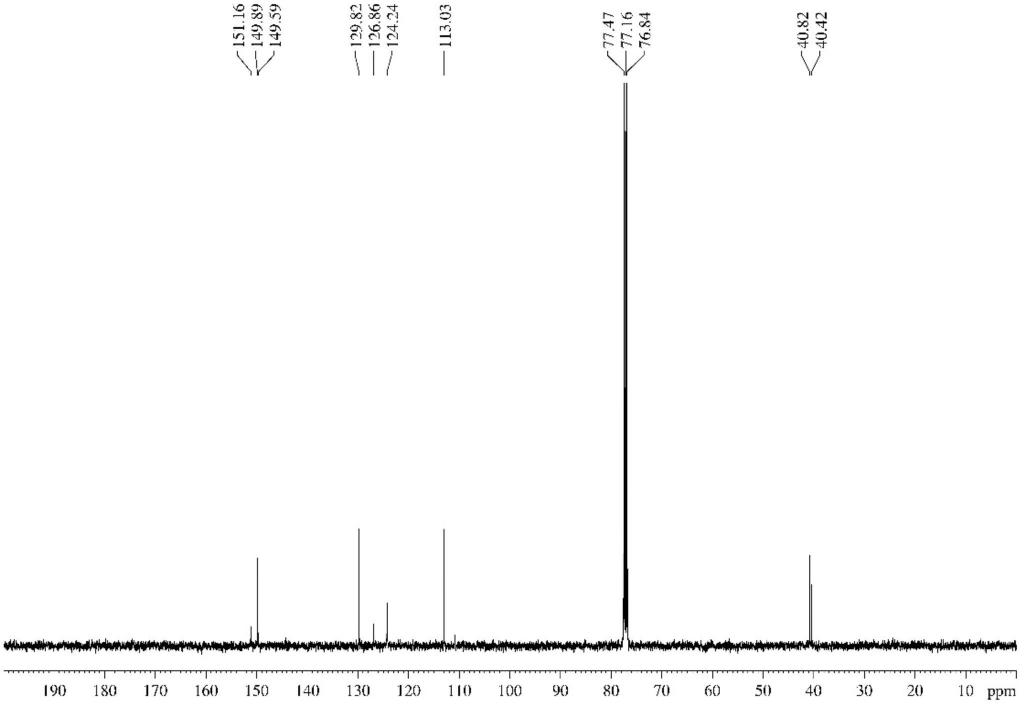

^{1}H (400 MHz, $CDCl_3$) and ^{13}C (101 MHz, $CDCl_3$) – NMR spectra of **8**

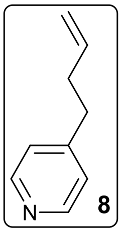

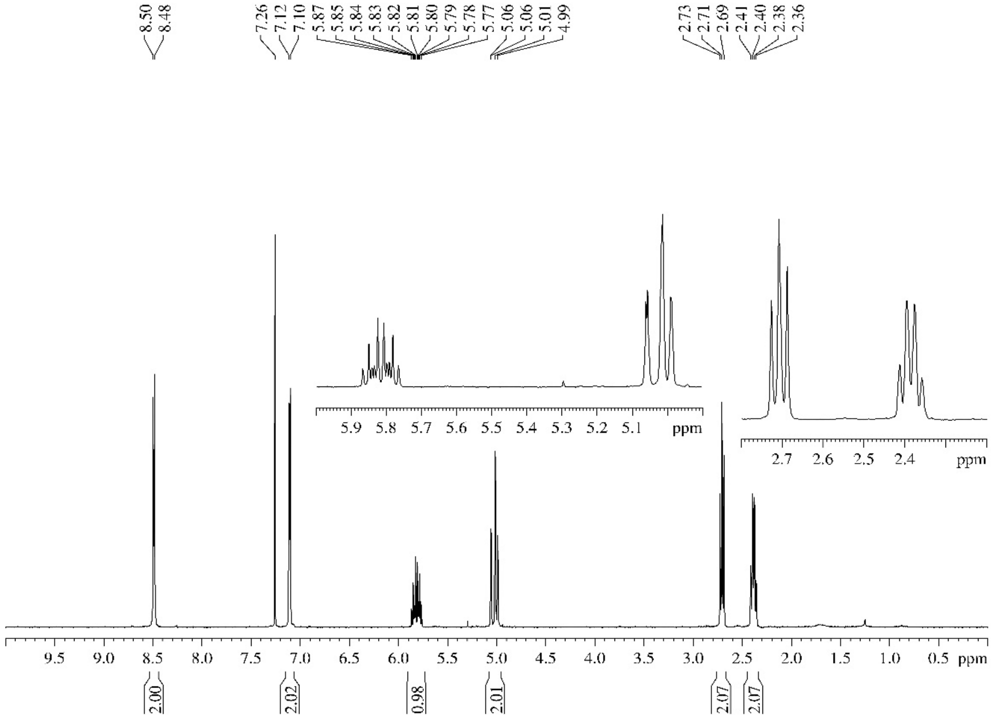

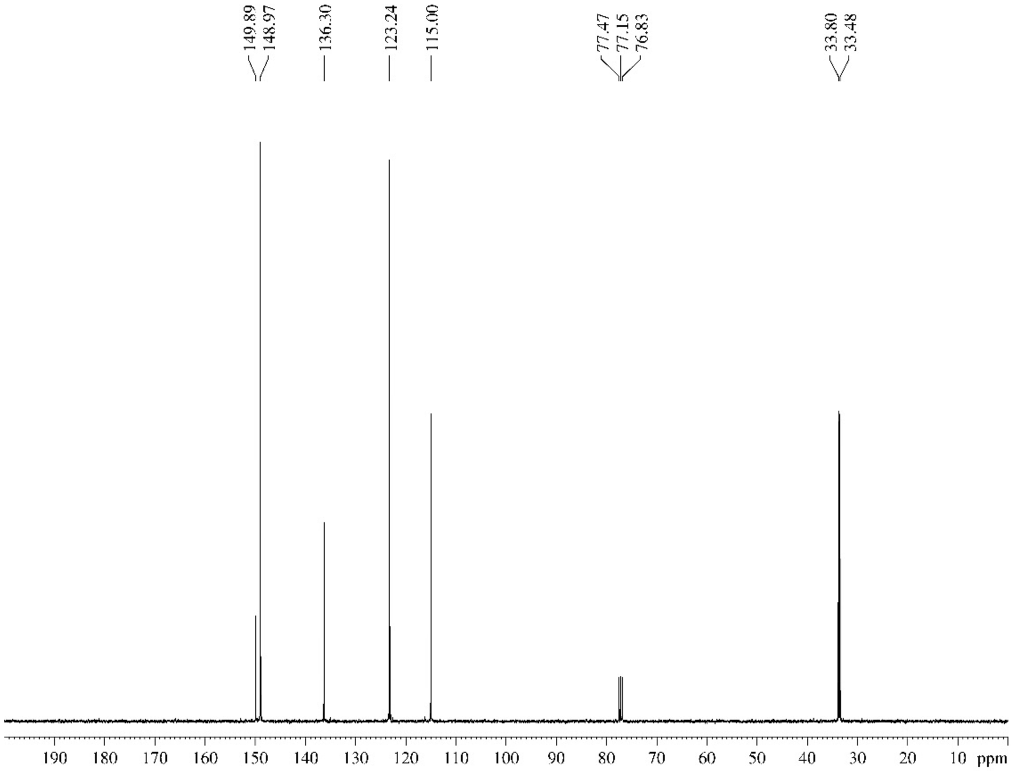

^{1}H (400 MHz, $CDCl_3$) and ^{13}C (101 MHz, $CDCl_3$) – NMR spectra of **9**

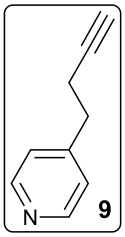

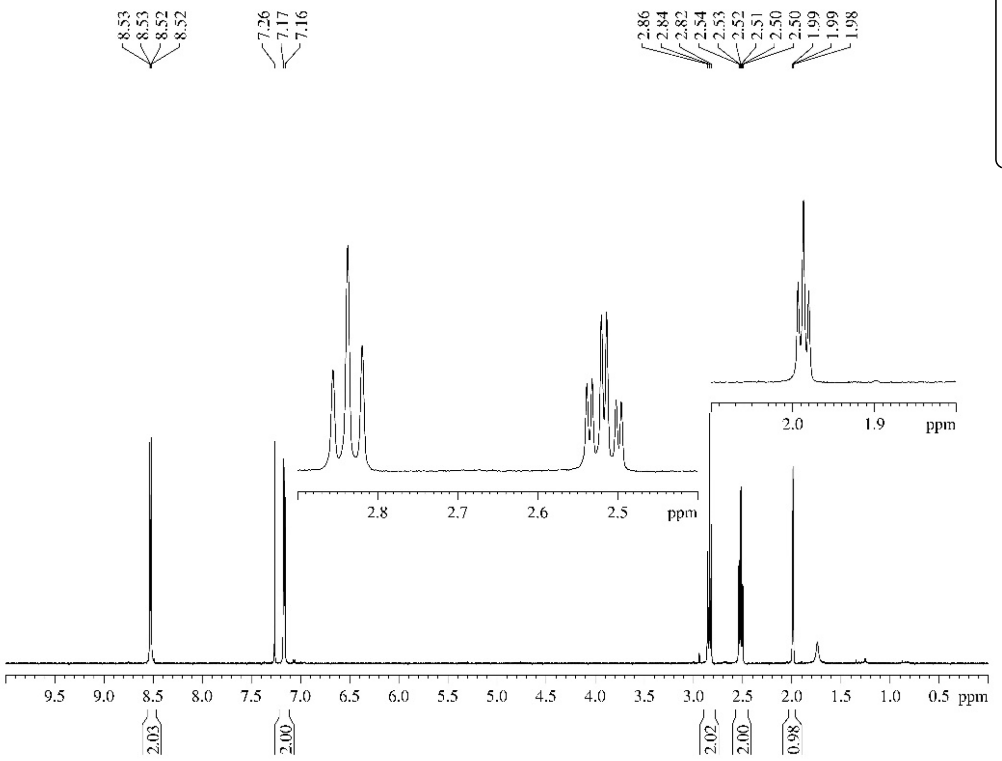

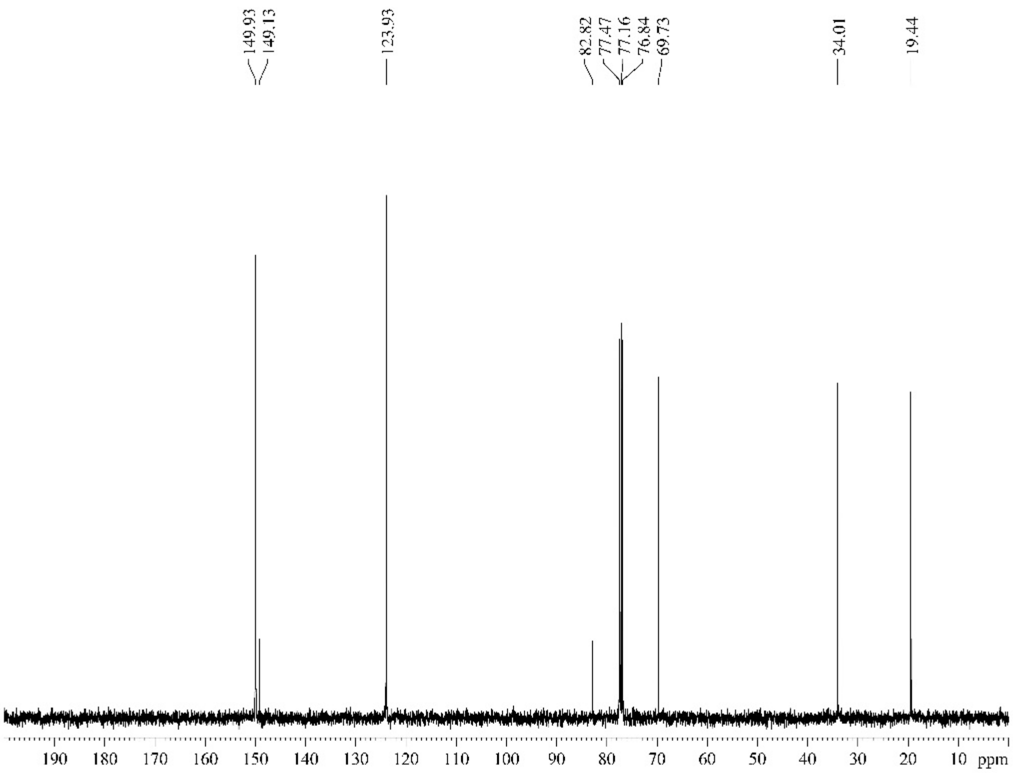

^{1}H (400 MHz, $CDCl_3$) and ^{13}C (101 MHz, $CDCl_3$) – NMR spectra of **11**

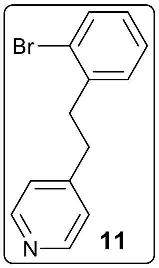

11

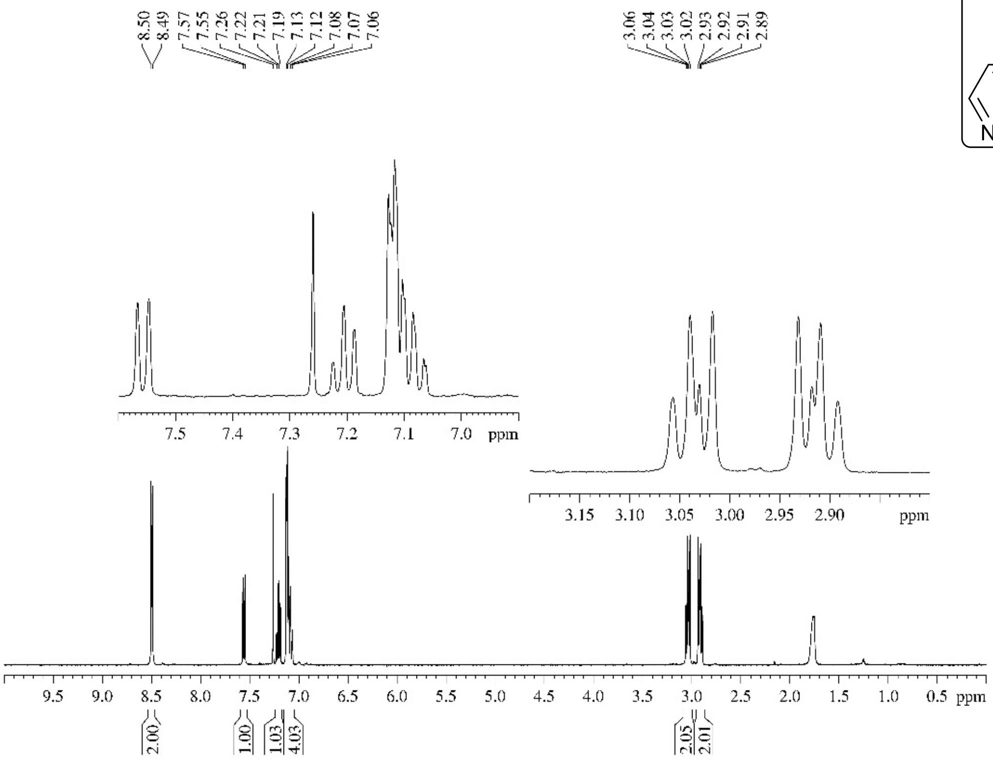

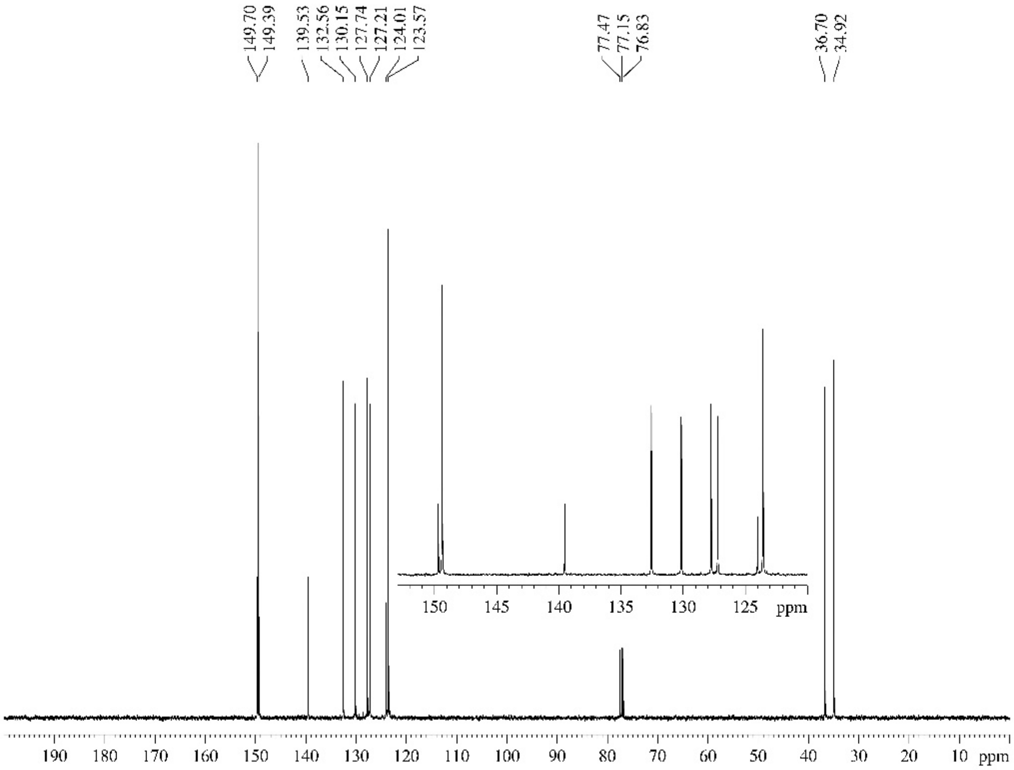

^{1}H (400 MHz, $CDCl_3$) and ^{13}C (101 MHz, $CDCl_3$) – NMR spectra of **12**

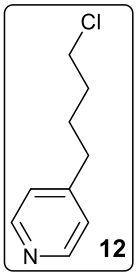

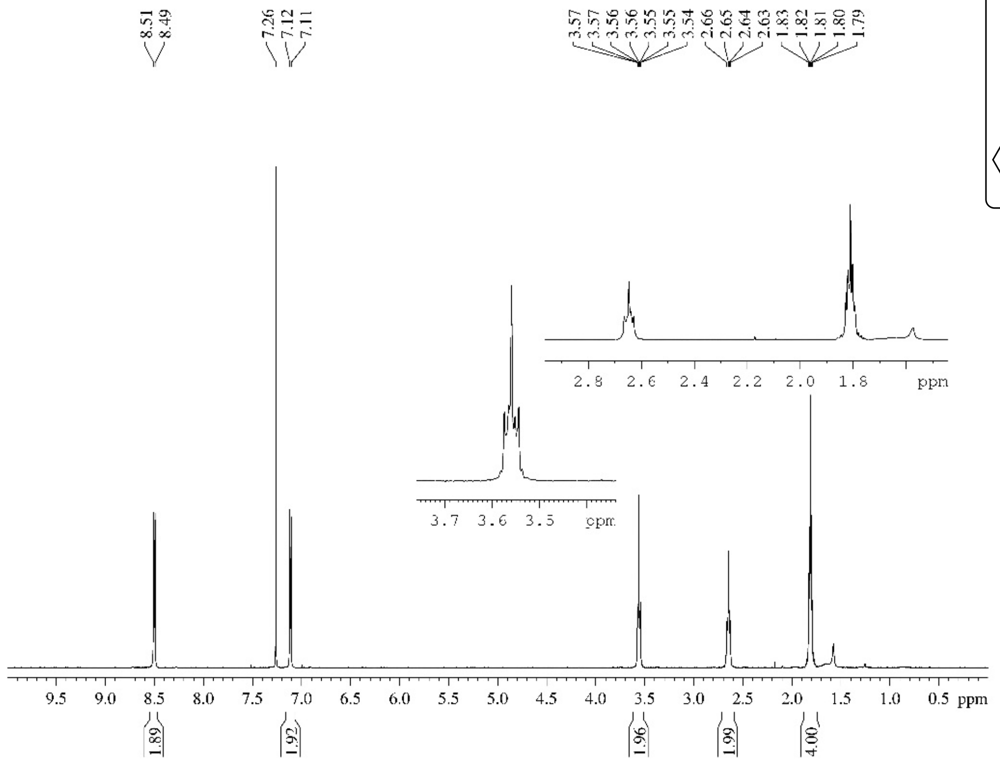

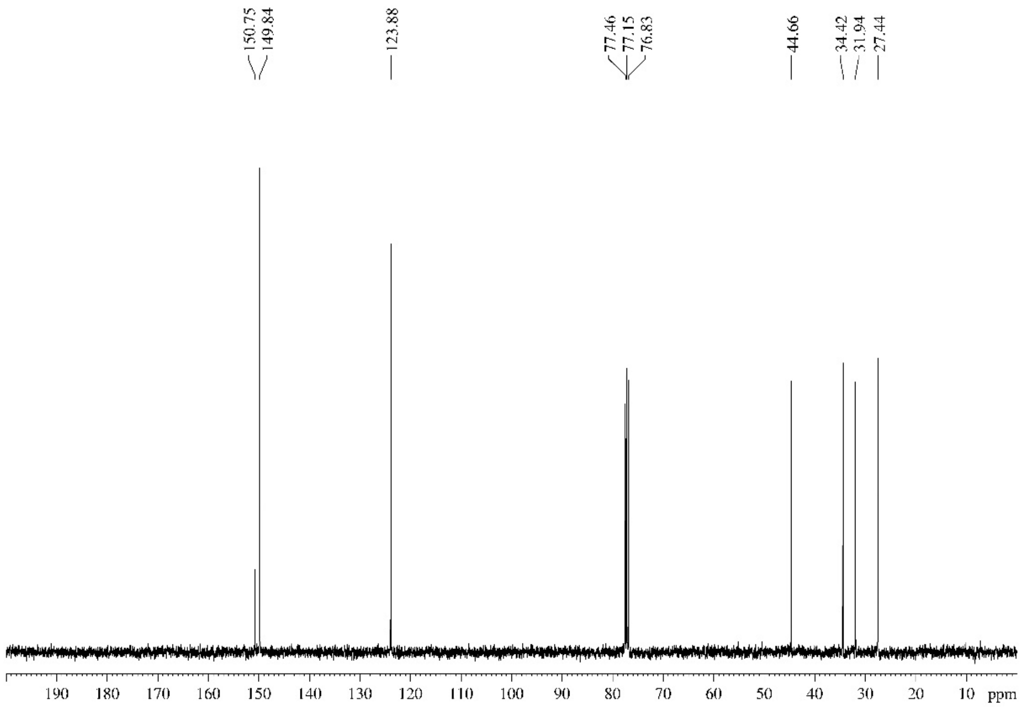

^{1}H (400 MHz, $CDCl_3$) and ^{13}C (76 MHz, $CDCl_3$) – NMR spectra of **13**

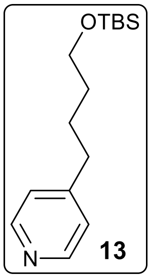

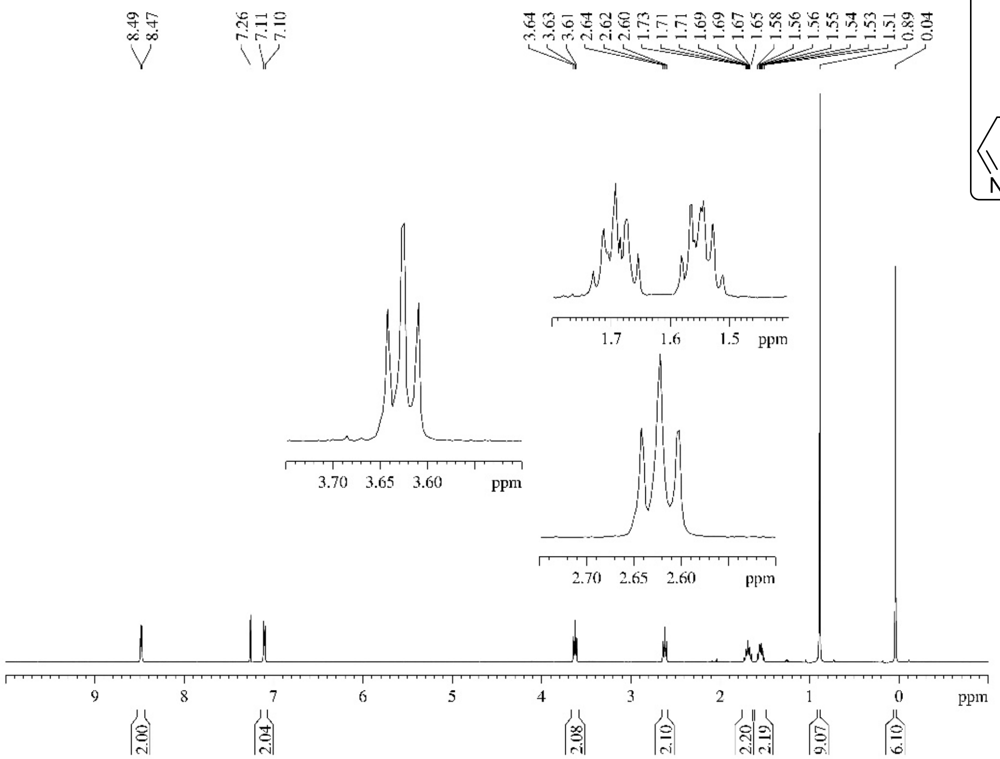

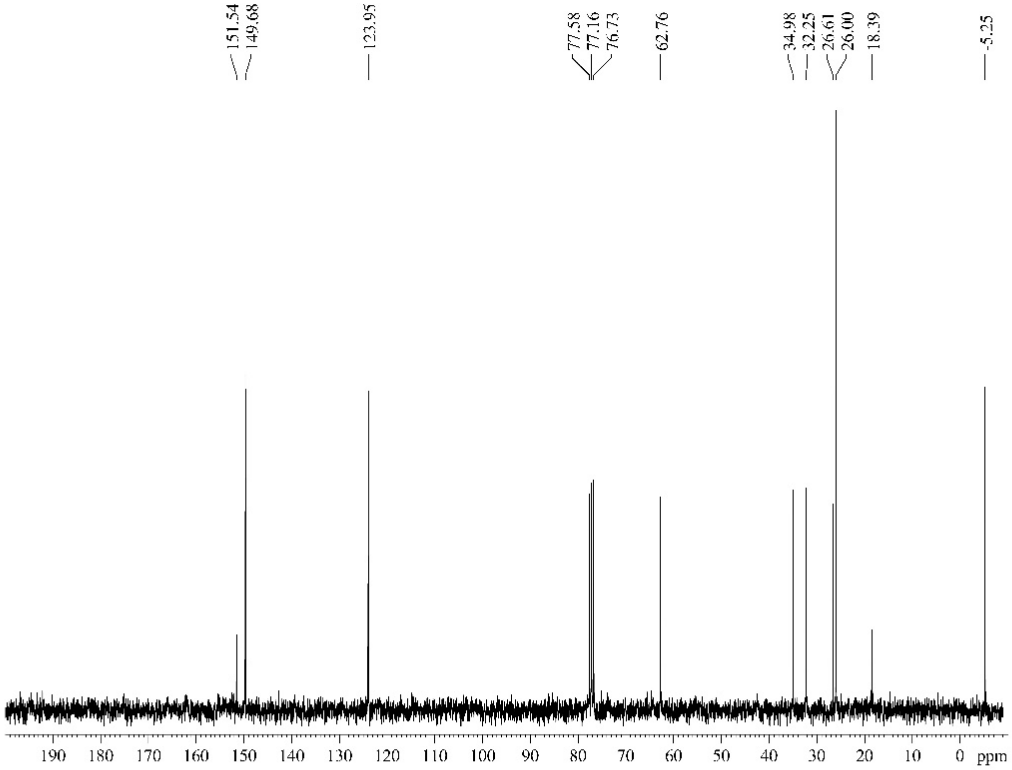

^{29}Si (57 MHz, $CDCl_3$) – NMR spectrum of **13**

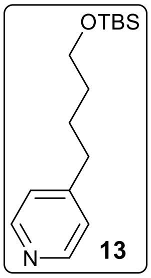

13

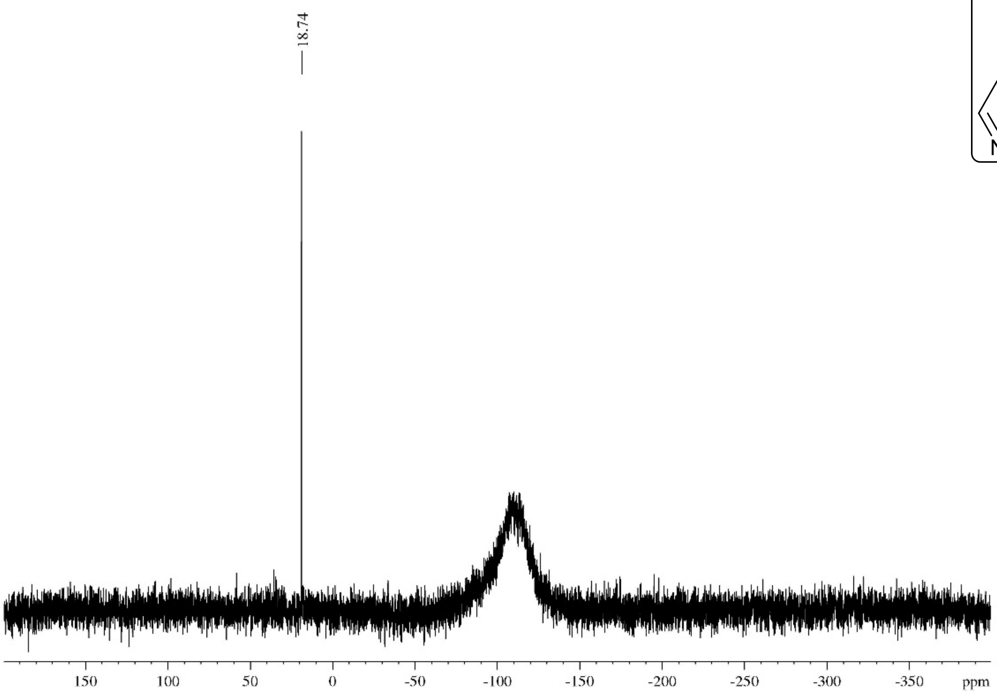

^{1}H (400 MHz, $CDCl_3$) and ^{13}C (101 MHz, $CDCl_3$) – NMR spectra of **14**

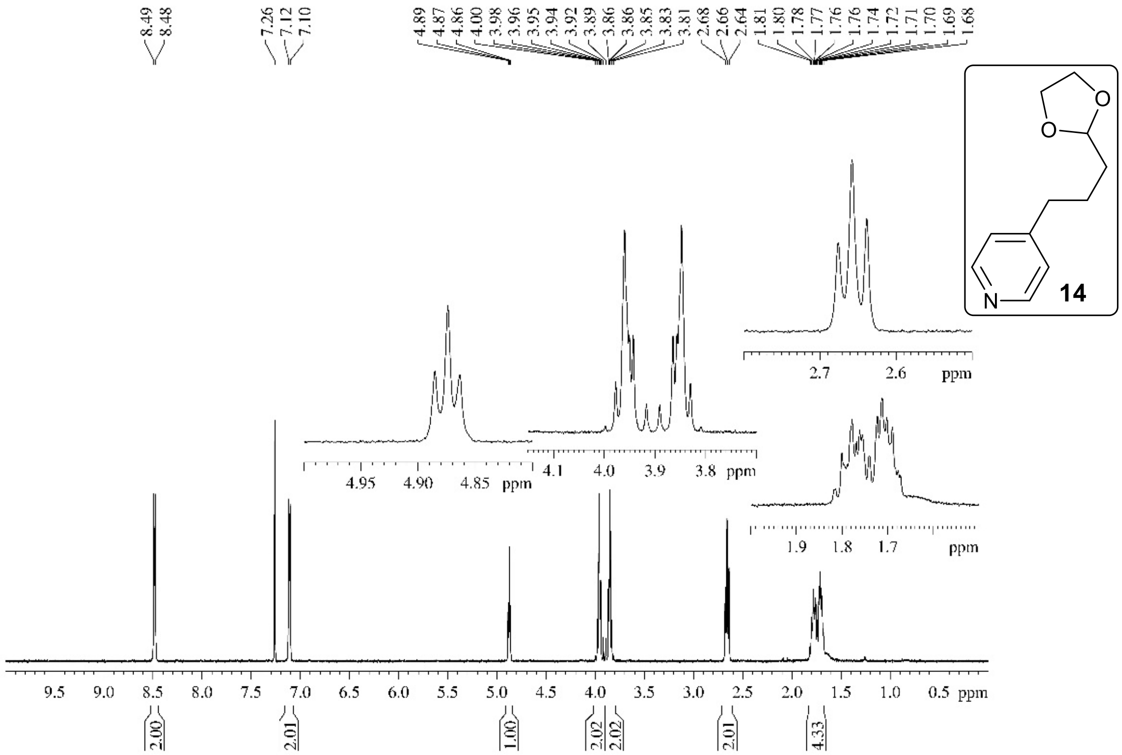

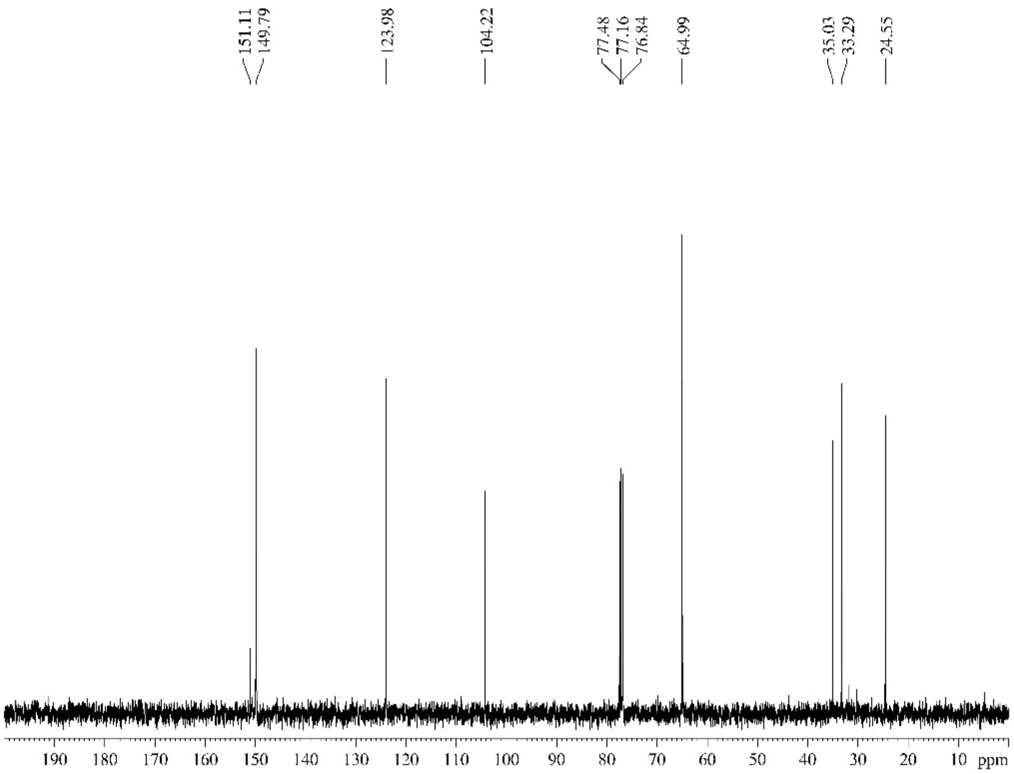

^{1}H (400 MHz, $CDCl_3$) and ^{13}C (101 MHz, $CDCl_3$) – NMR spectra of **15**

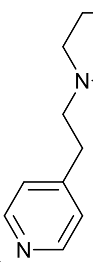

15

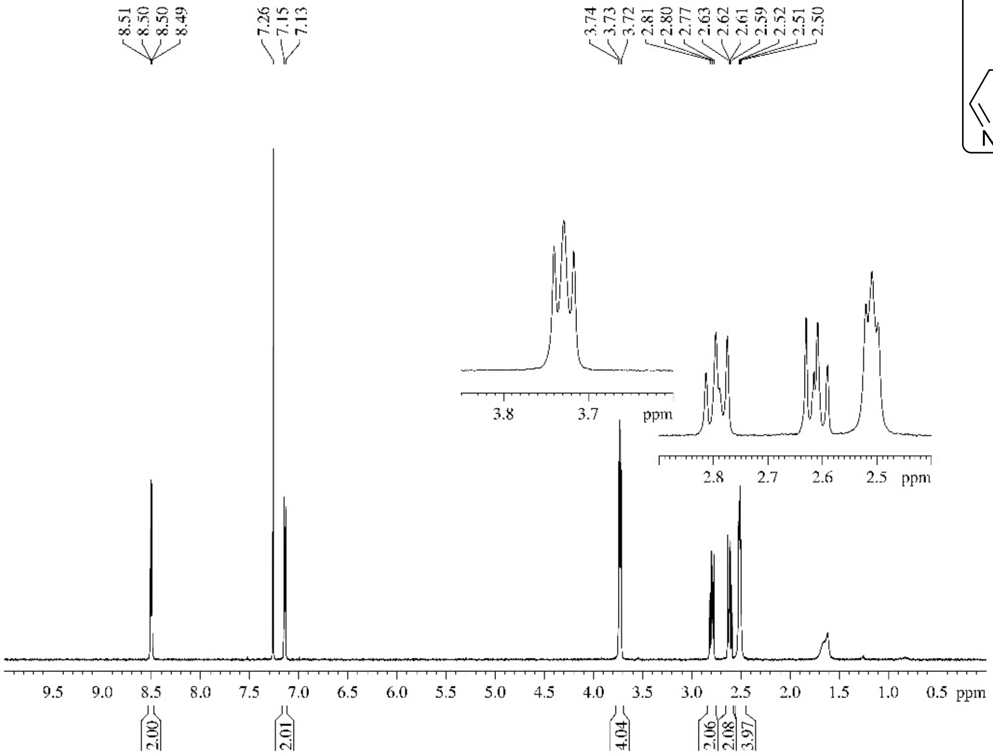

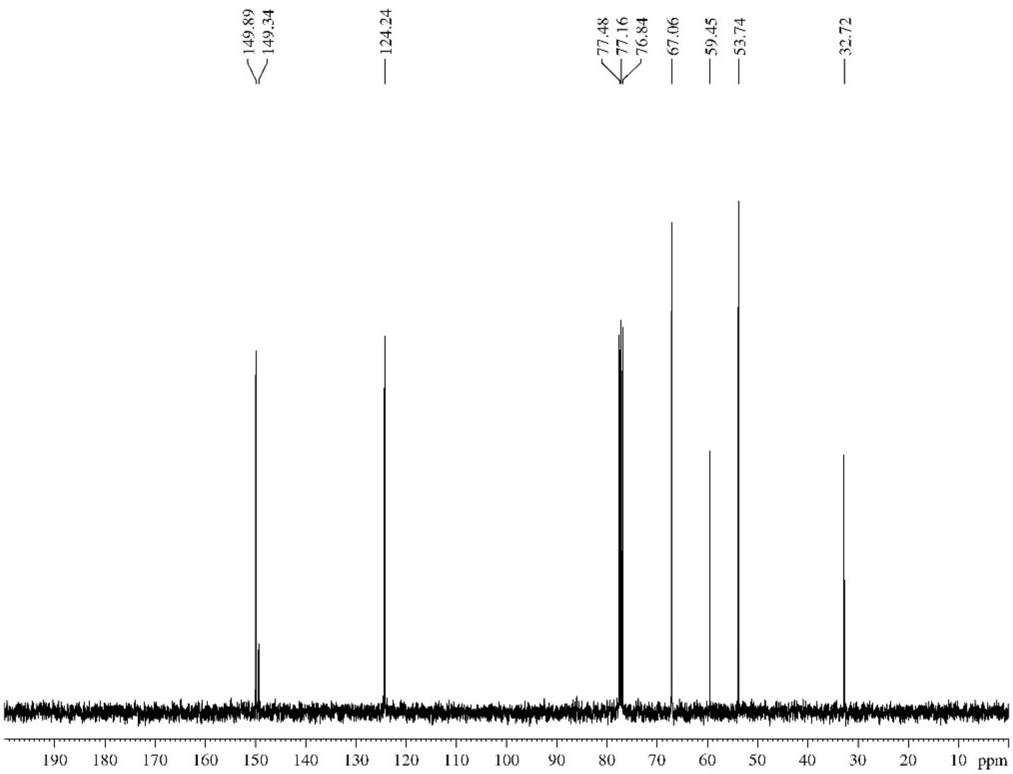

^1H (400 MHz, $CDCl_3$) and ^{13}C (101 MHz, $CDCl_3$) – NMR spectra of **16**

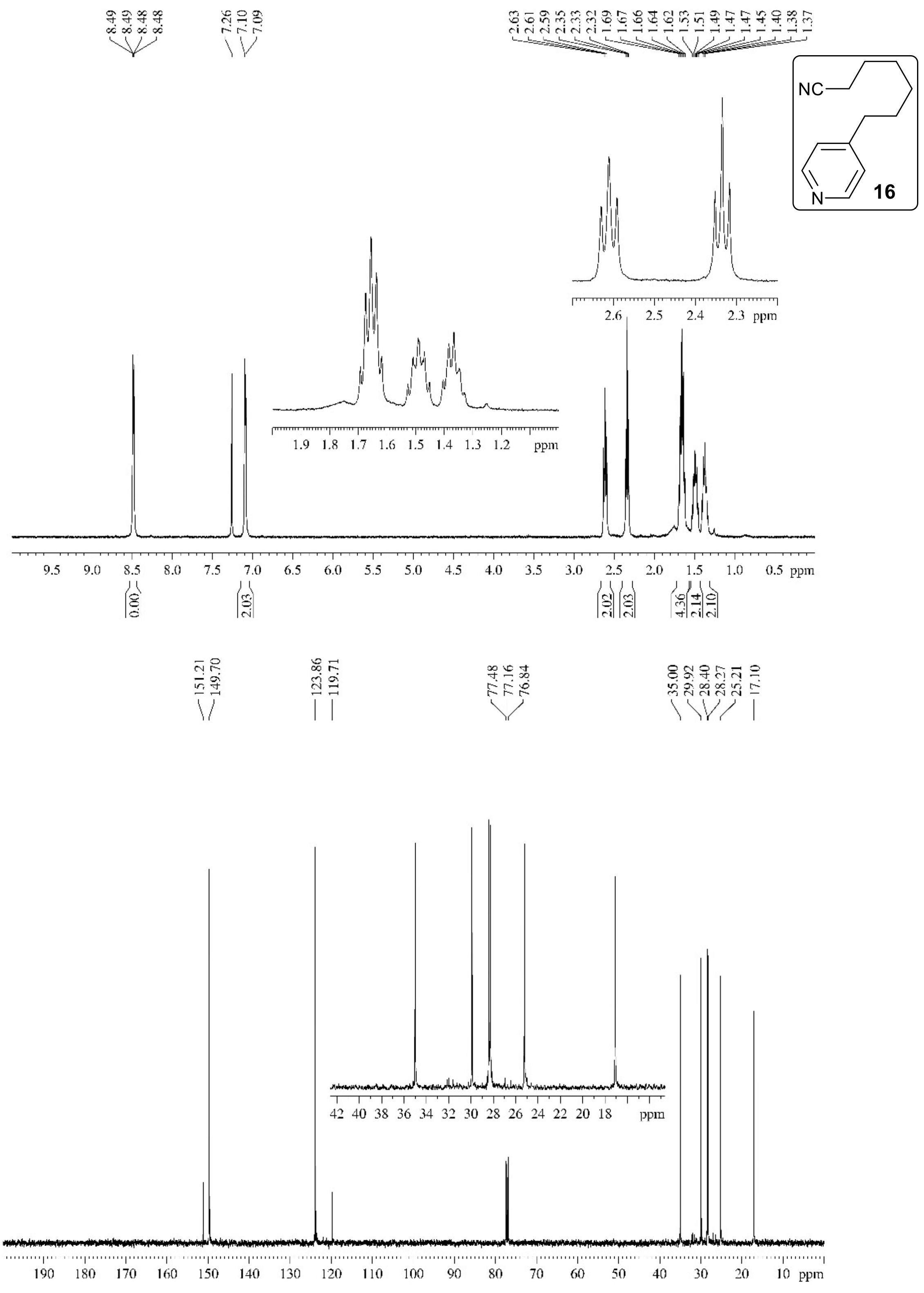

^{1}H (400 MHz, $CDCl_3$) and ^{13}C (101 MHz, $CDCl_3$) – NMR spectra of **17.1**

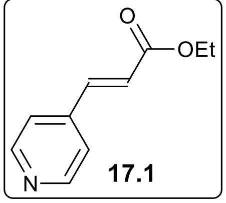

17.1

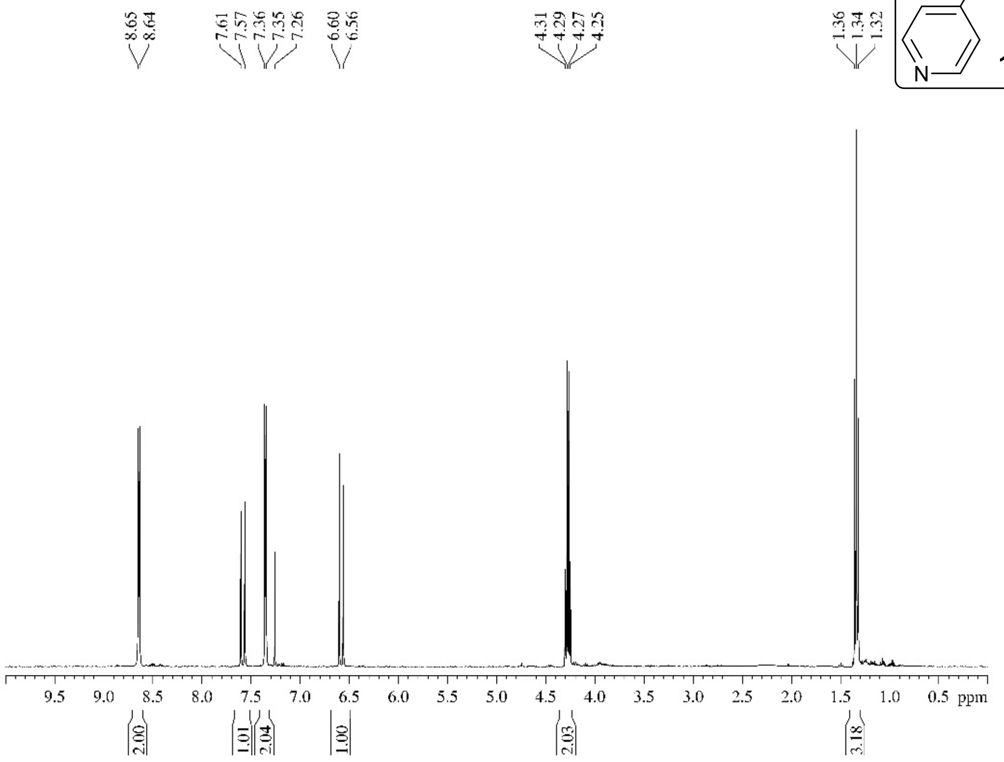

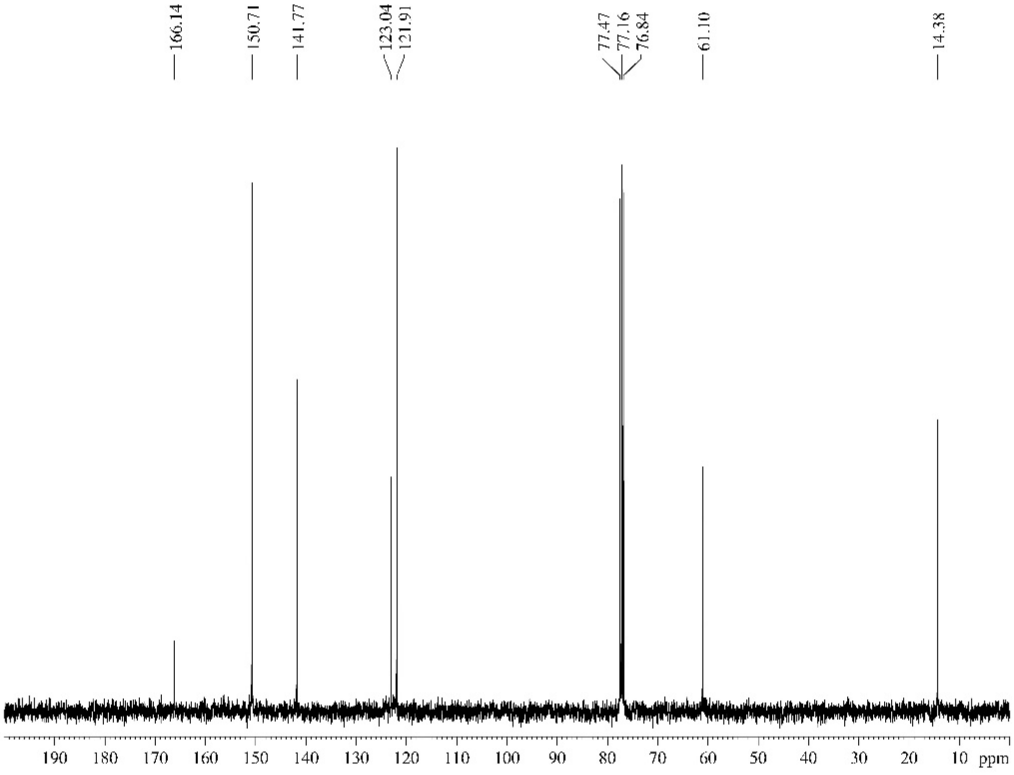

^{1}H (400 MHz, $CDCl_3$) and ^{13}C (101 MHz, $CDCl_3$) – NMR spectra of **17**

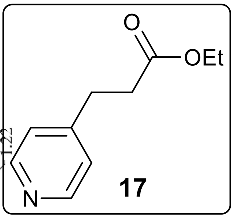

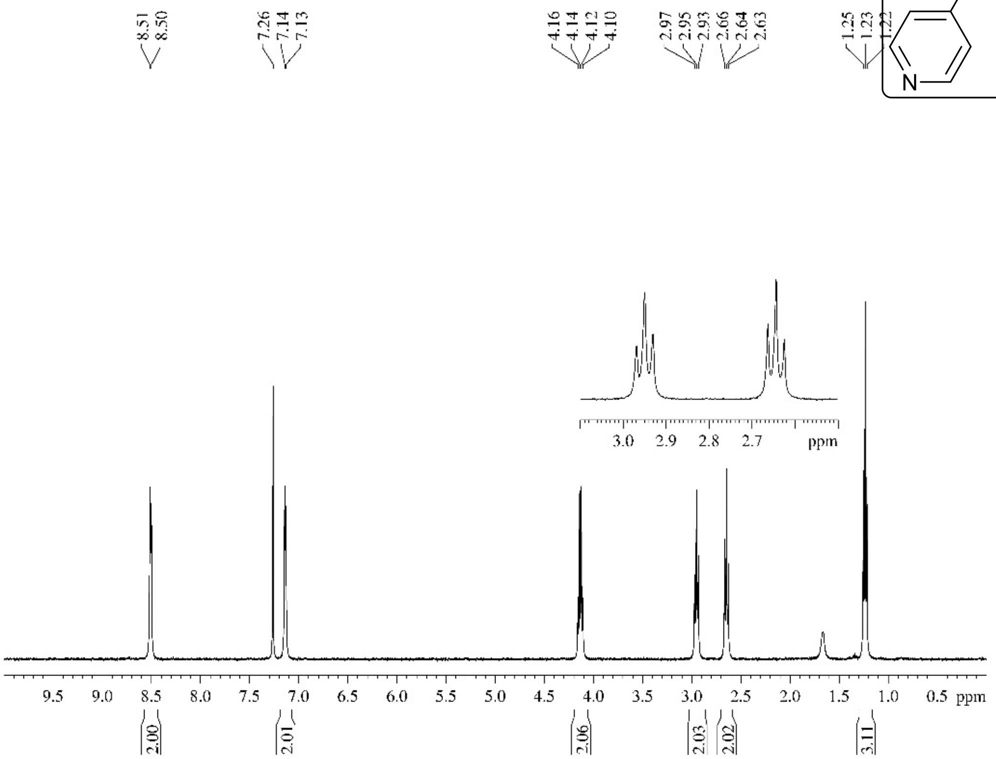

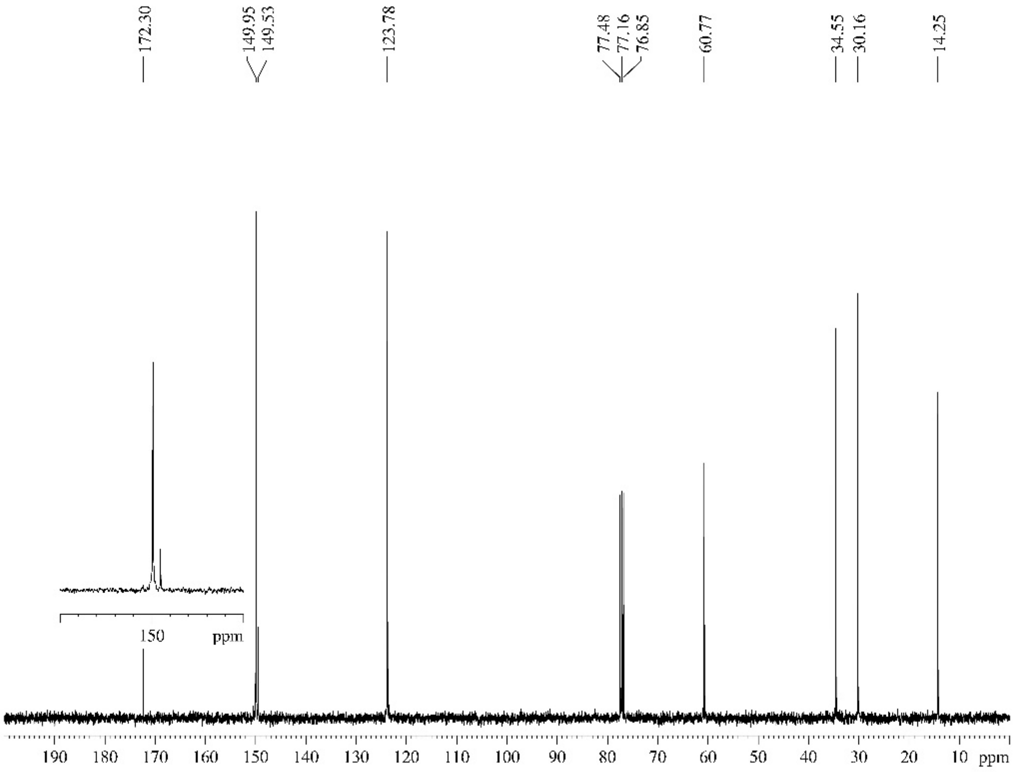

^{1}H (400 MHz, $CDCl_3$) and ^{13}C (101 MHz, $CDCl_3$) – NMR spectra of **18.1**

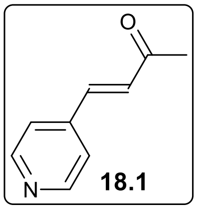

18.1

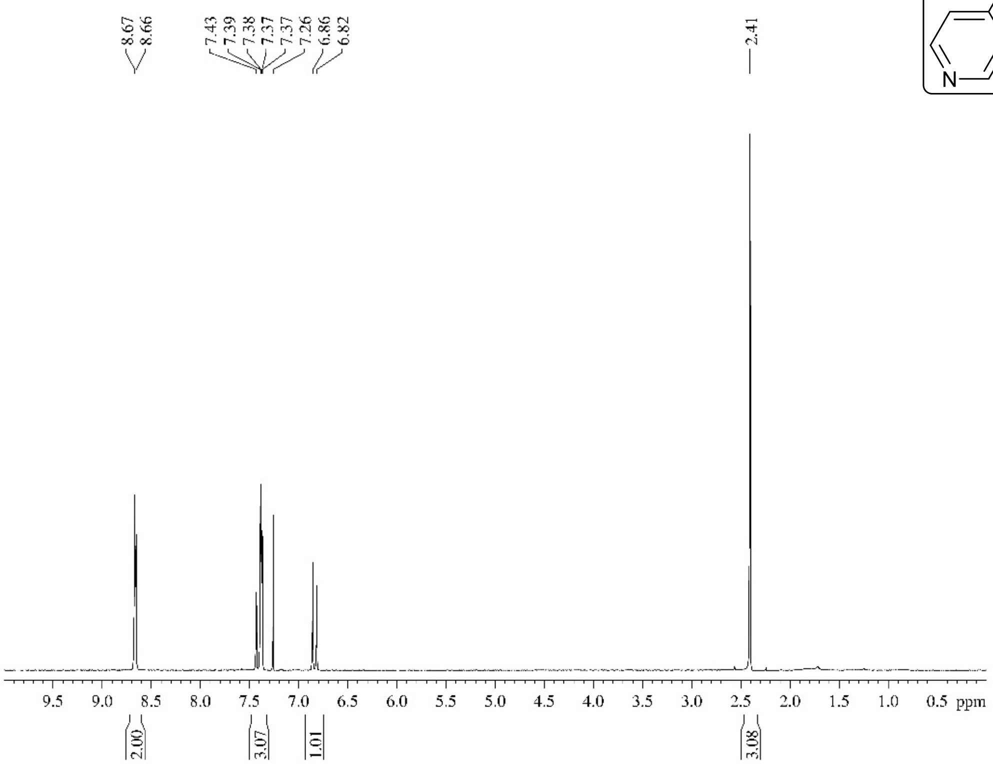

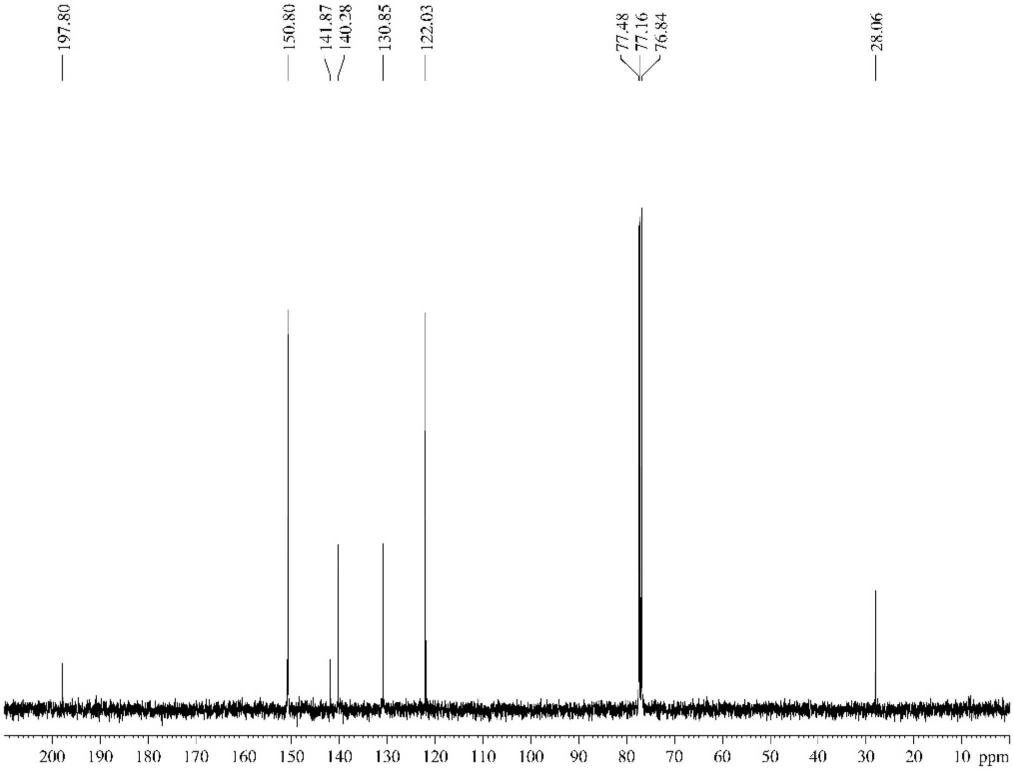

^{1}H (400 MHz, $CDCl_3$) and ^{13}C (101 MHz, $CDCl_3$) – NMR spectra of **18**

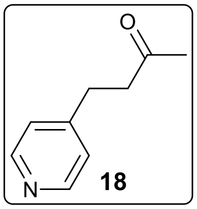

18

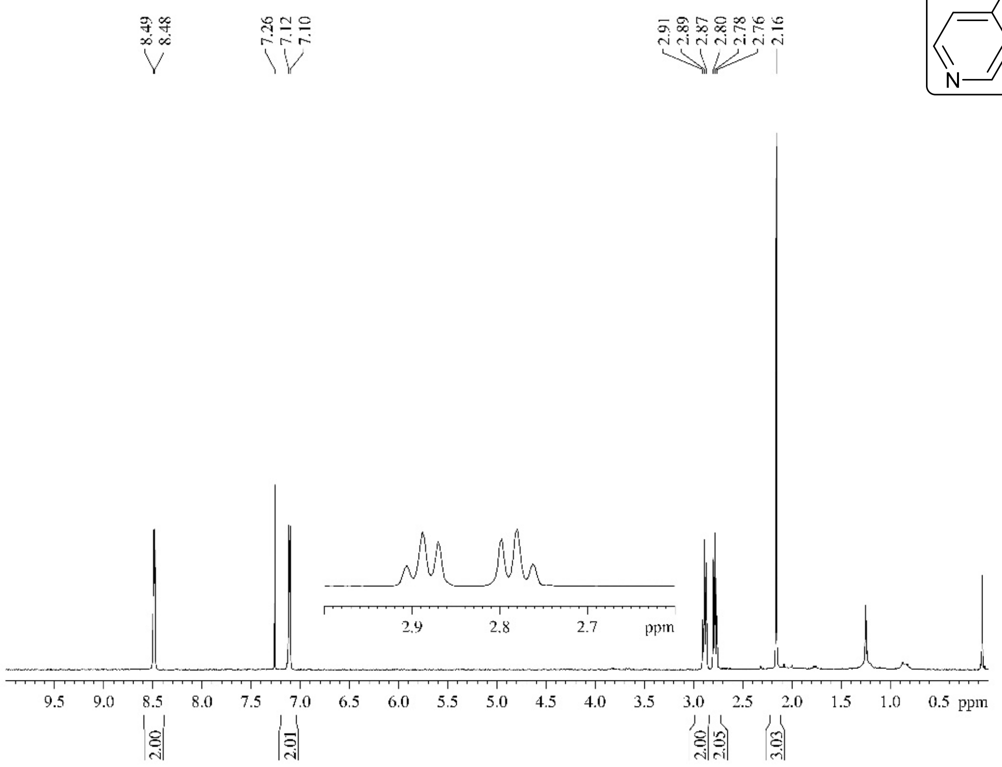

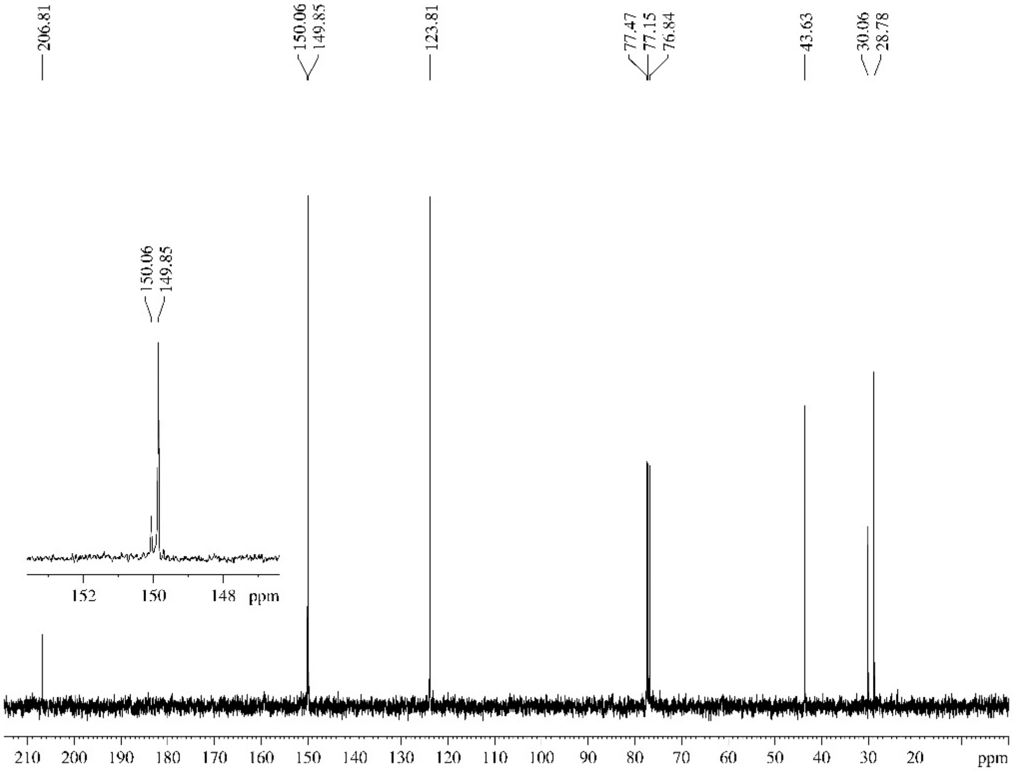

^{1}H (400 MHz, $CDCl_3$) and ^{13}C (101 MHz, $CDCl_3$) – NMR spectra of **19**

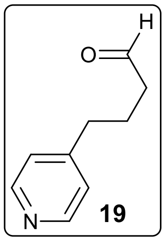

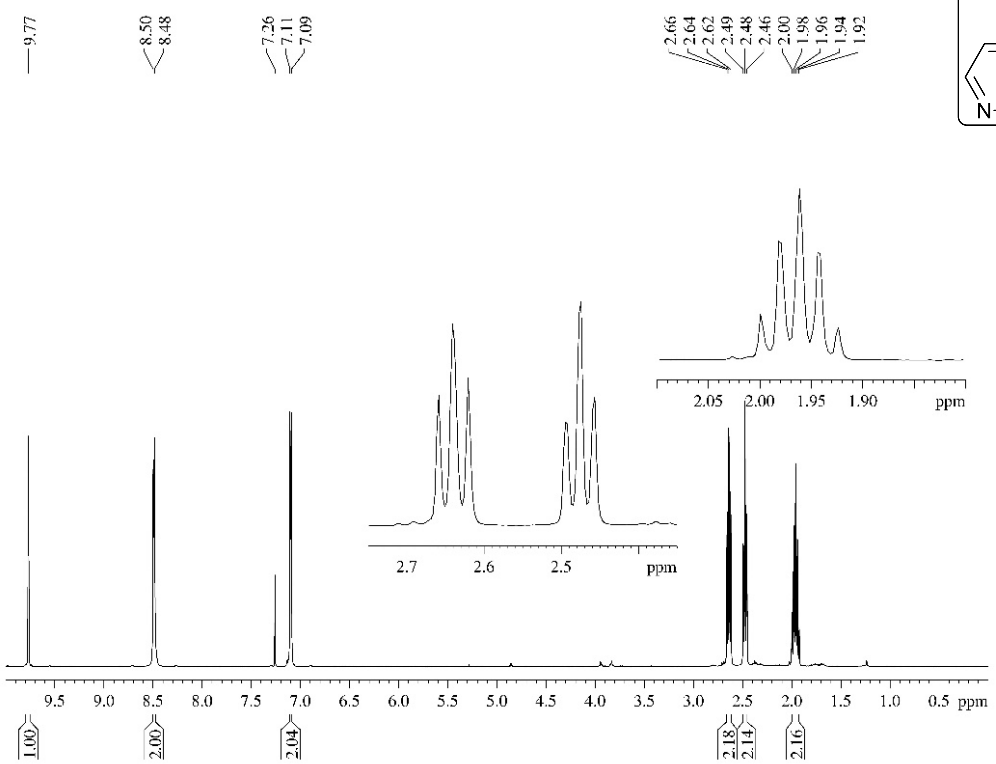

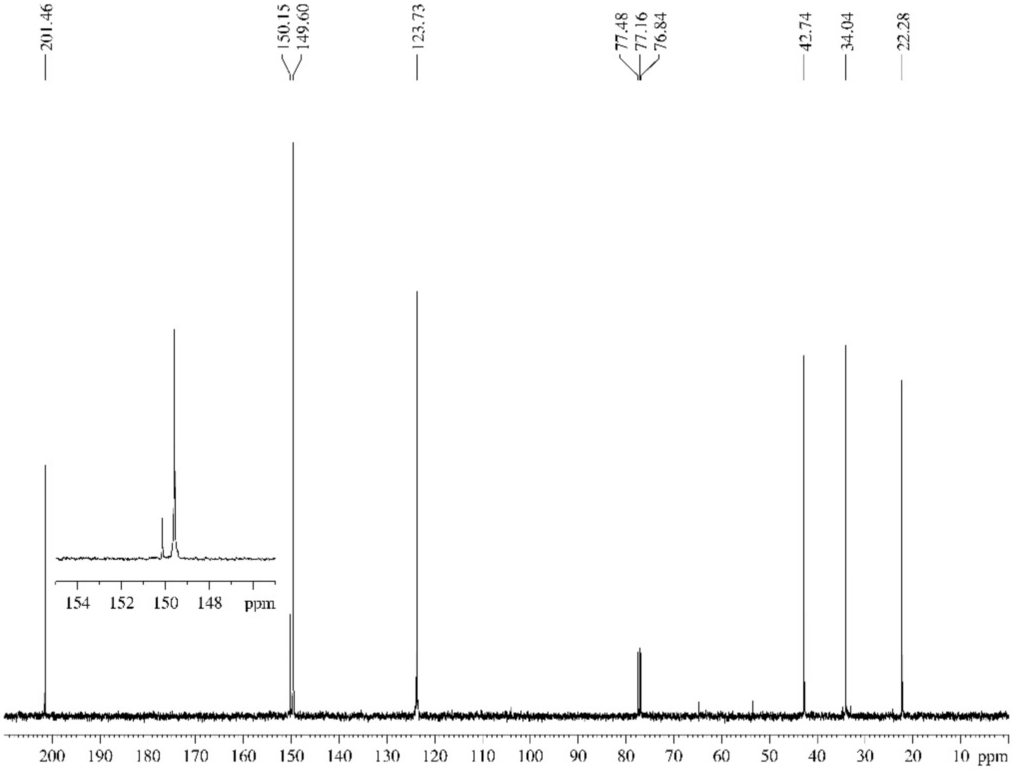

^1H (400 MHz, $CDCl_3$) and ^{13}C (101 MHz, $CDCl_3$) – NMR spectra of **20.2**

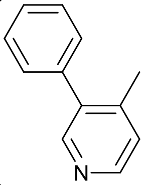

20.2

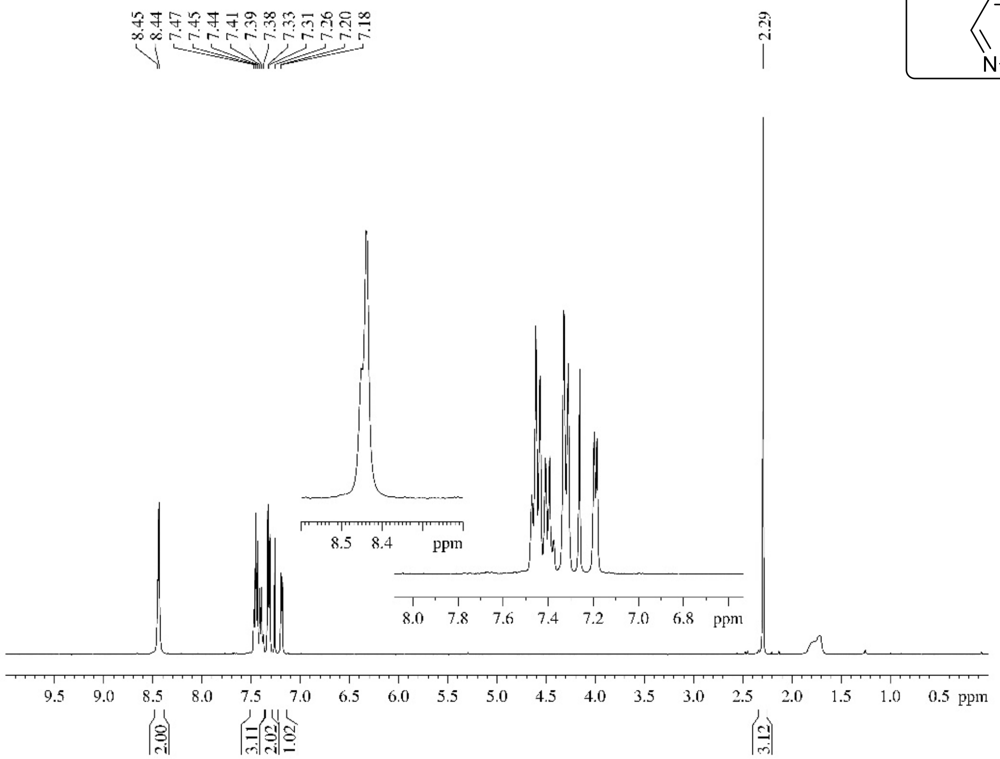

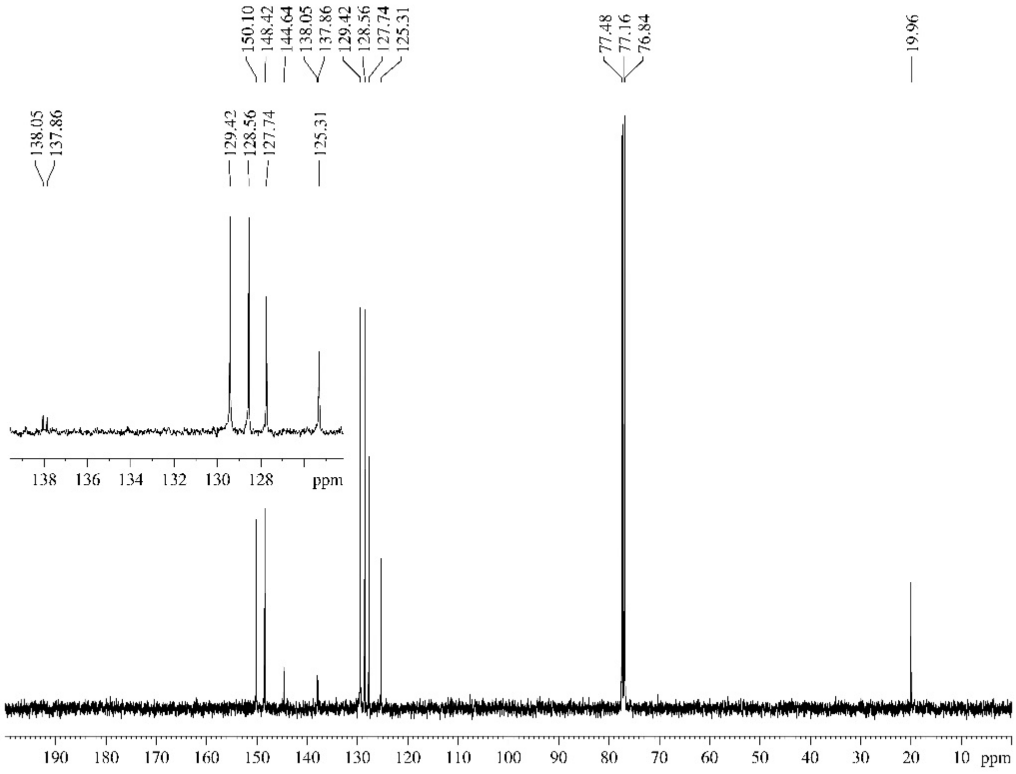

^{1}H (400 MHz, $CDCl_3$) and ^{13}C (101 MHz, $CDCl_3$) – NMR spectra of **20**

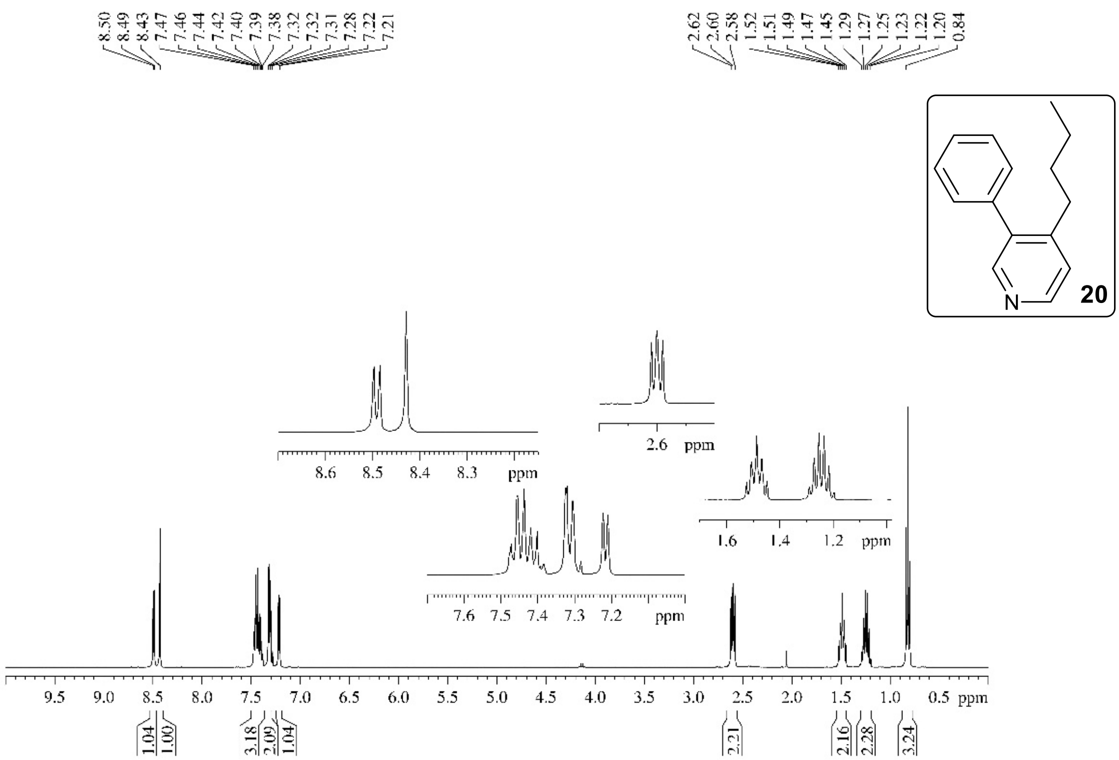

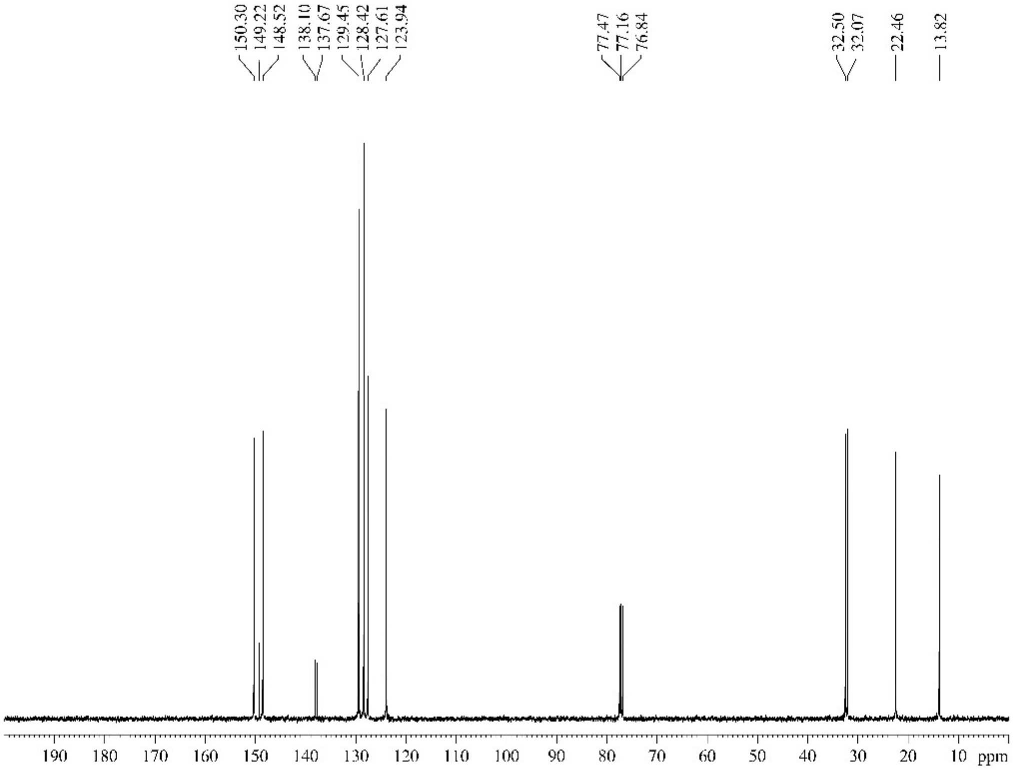

^{1}H (400 MHz, $CDCl_3$) and ^{13}C (76 MHz, $CDCl_3$) – NMR spectra of **21**

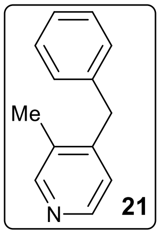

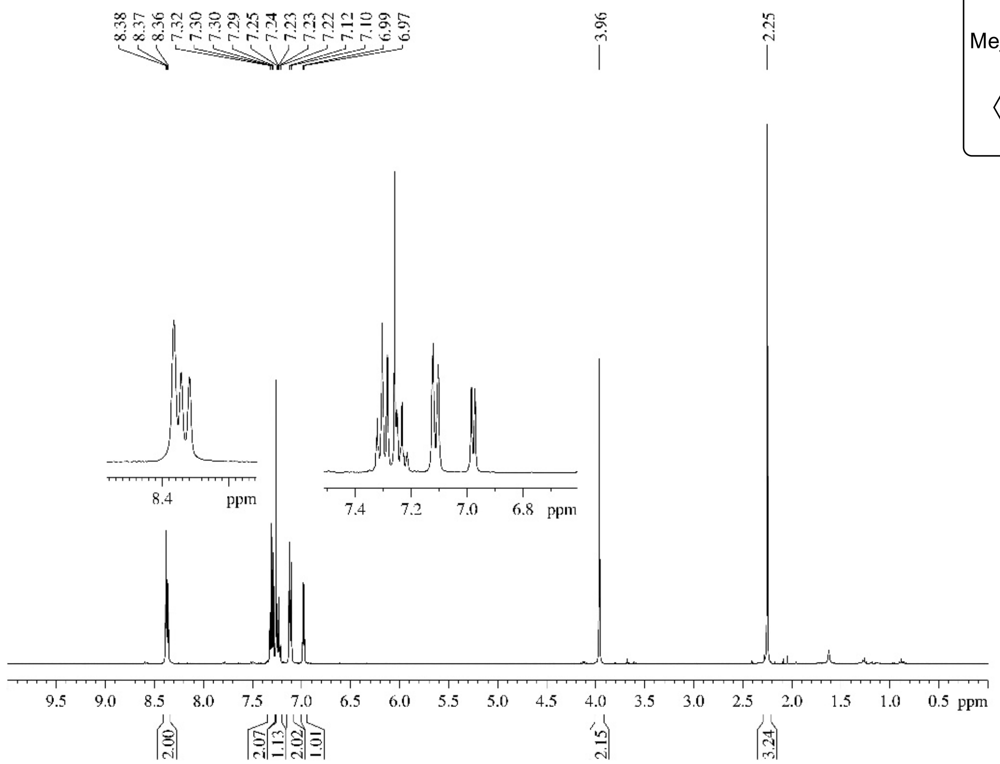

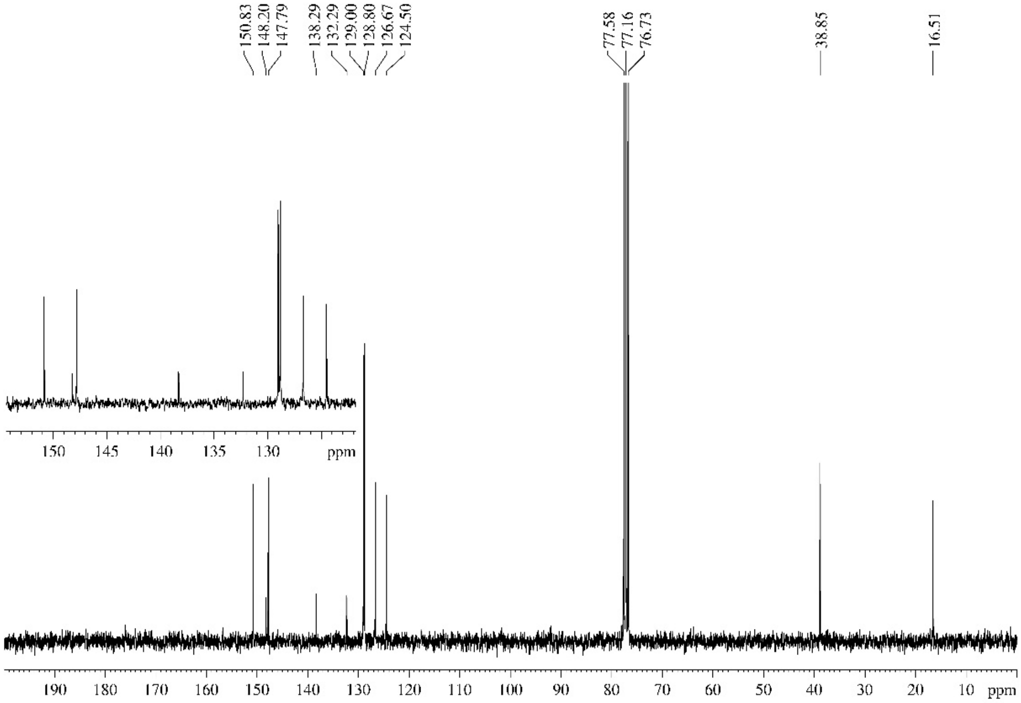

^{1}H (400 MHz, $CDCl_3$) and ^{13}C (101 MHz, $CDCl_3$) – NMR spectra of **22**

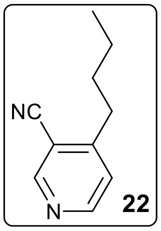

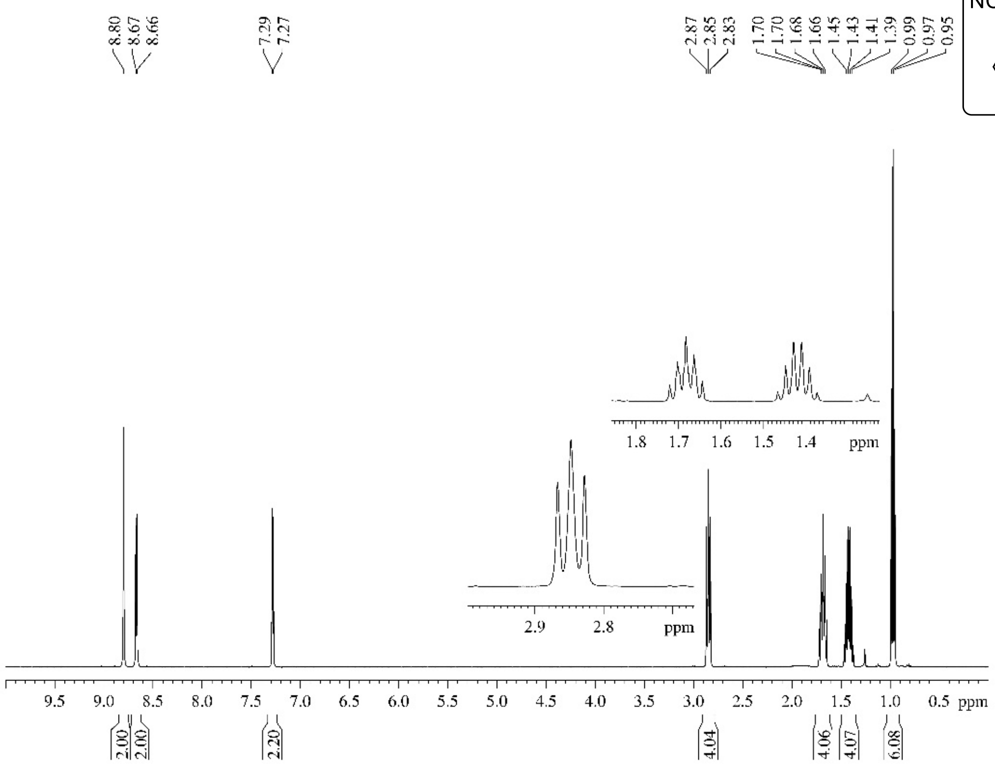

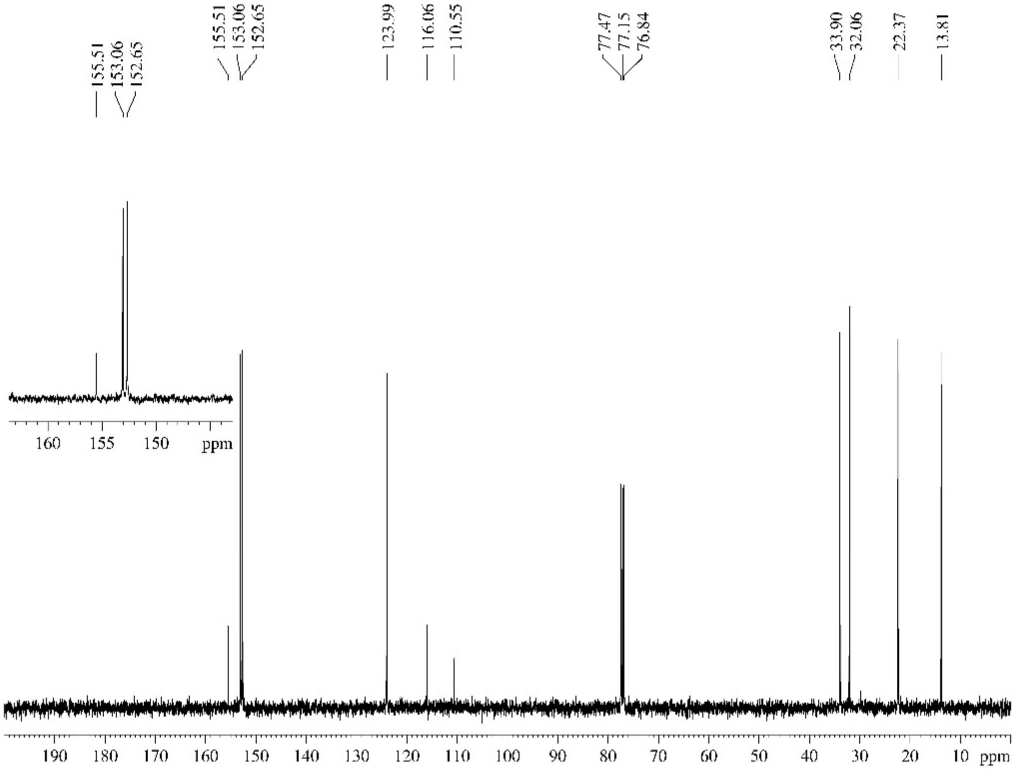

^1H (400 MHz, $CDCl_3$) and ^{13}C (101 MHz, $CDCl_3$) – NMR spectra of **23**

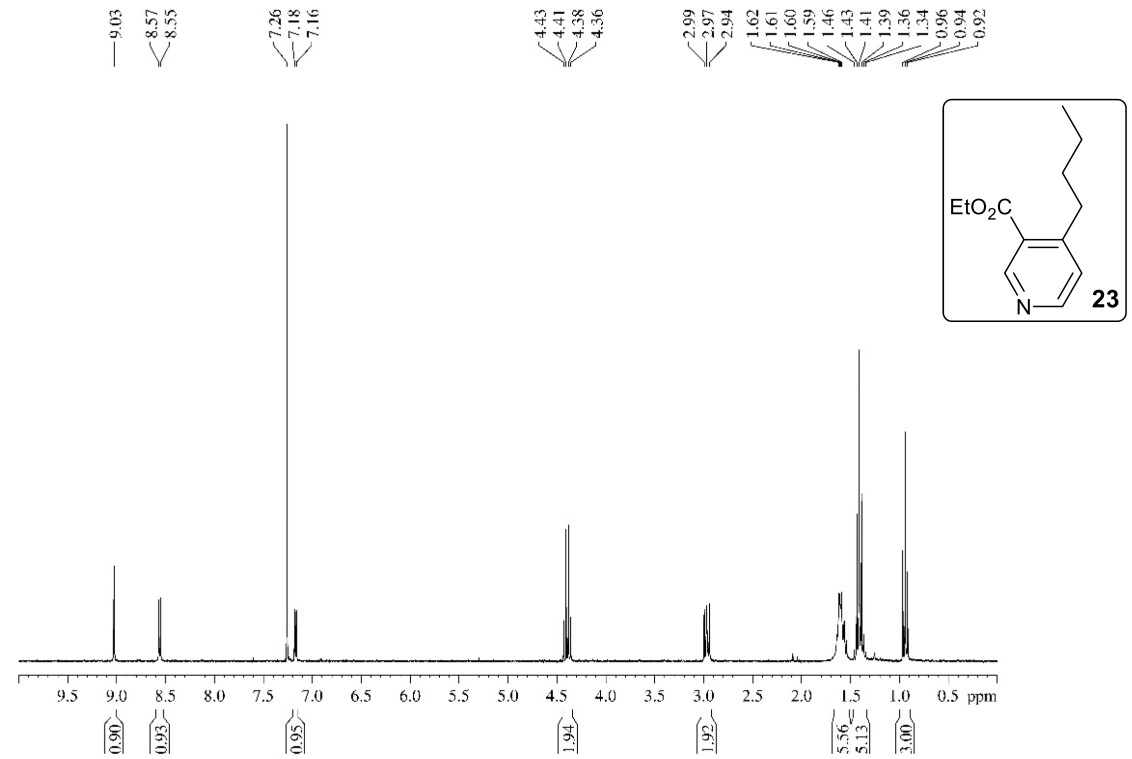

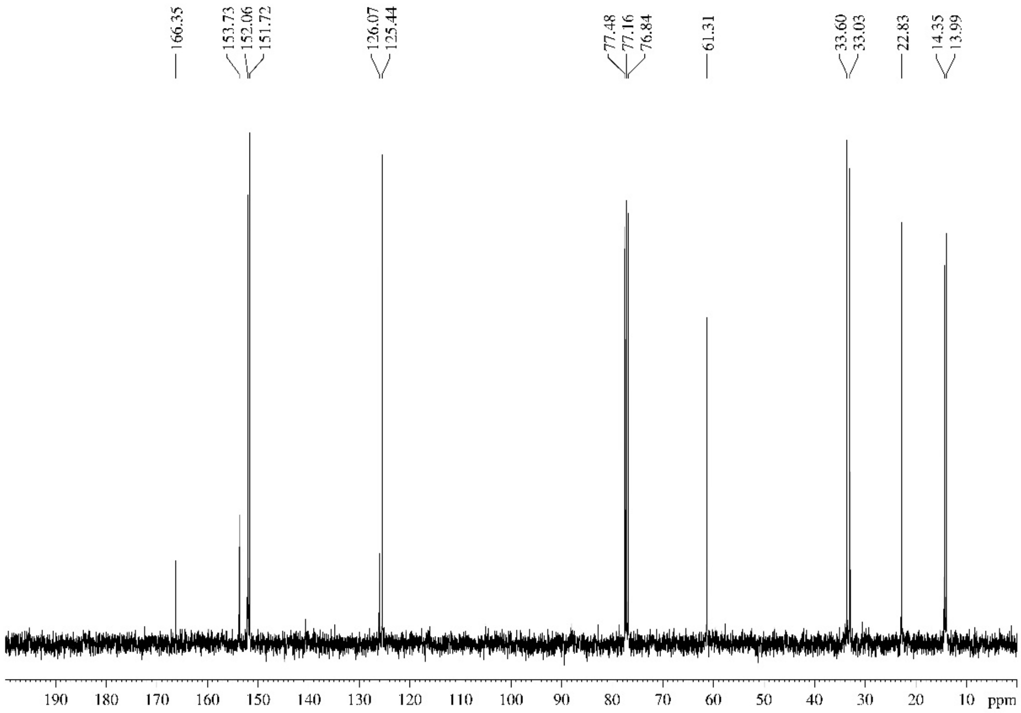

^{1}H (400 MHz, $CDCl_3$) and ^{13}C (101 MHz, $CDCl_3$) – NMR spectra of **24**

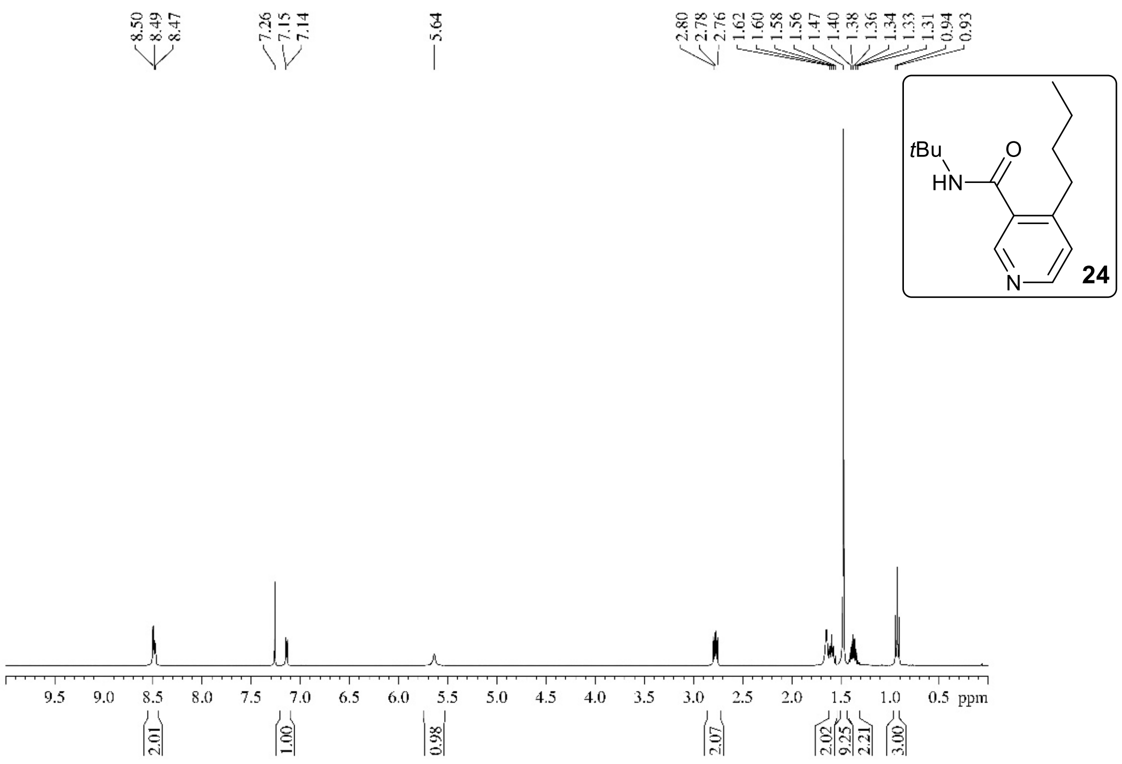

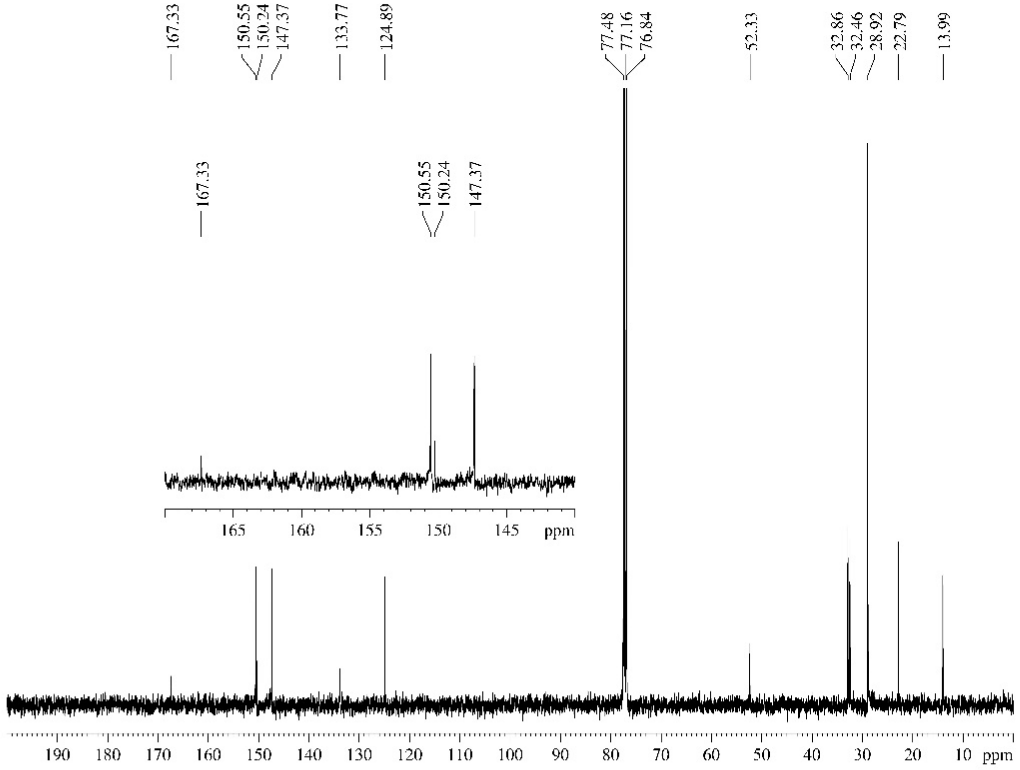

^{1}H (400 MHz, $CDCl_3$) and ^{13}C (101 MHz, $CDCl_3$) – NMR spectra of **25.1**

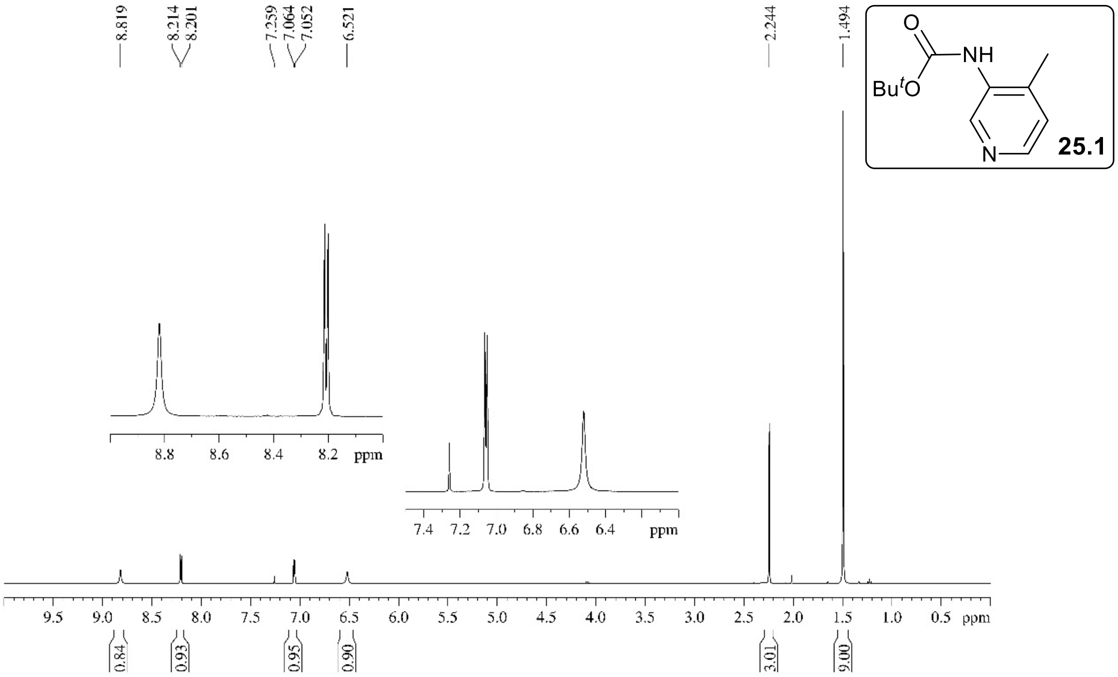

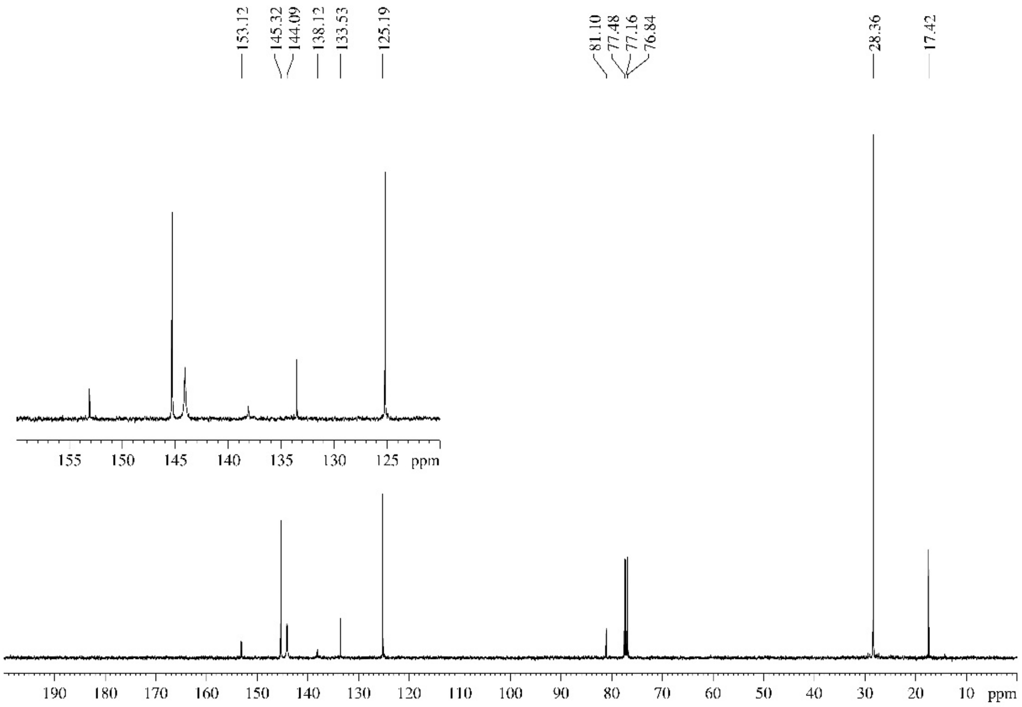

^{1}H (400 MHz, $CDCl_3$) and ^{13}C (101 MHz, $CDCl_3$) – NMR spectra of **25**

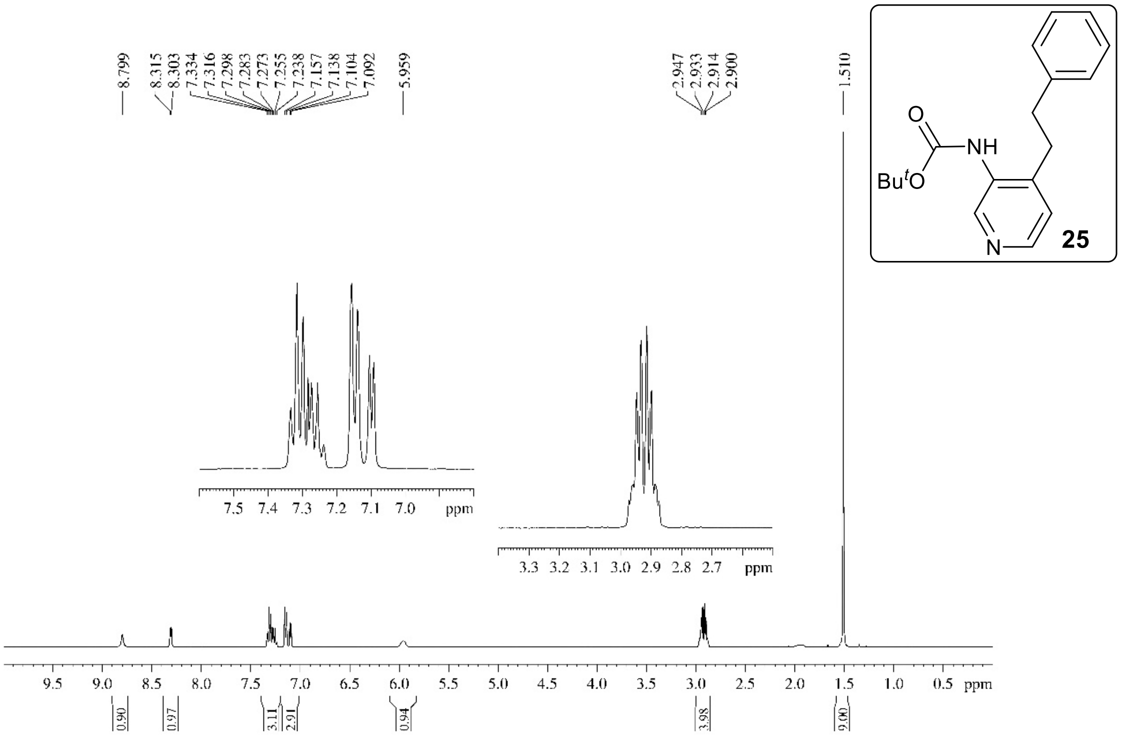

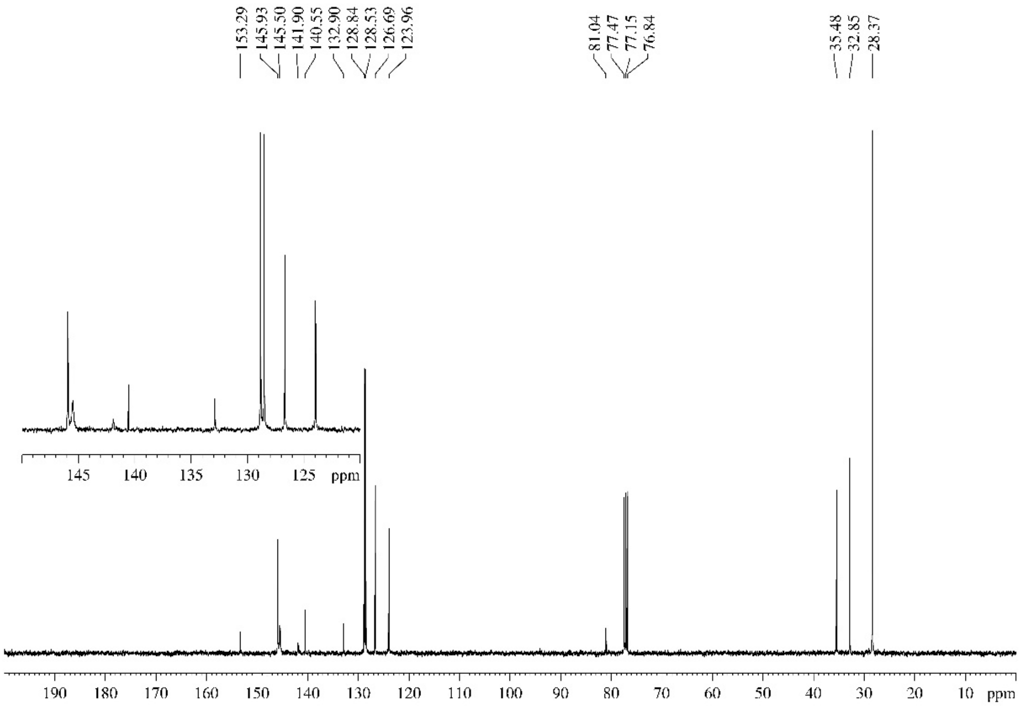

^{1}H (400 MHz, CDCl$_3$) and ^{13}C (101 MHz, CDCl$_3$) – NMR spectra of **27**

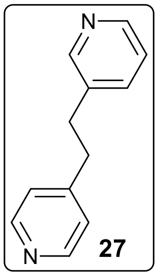

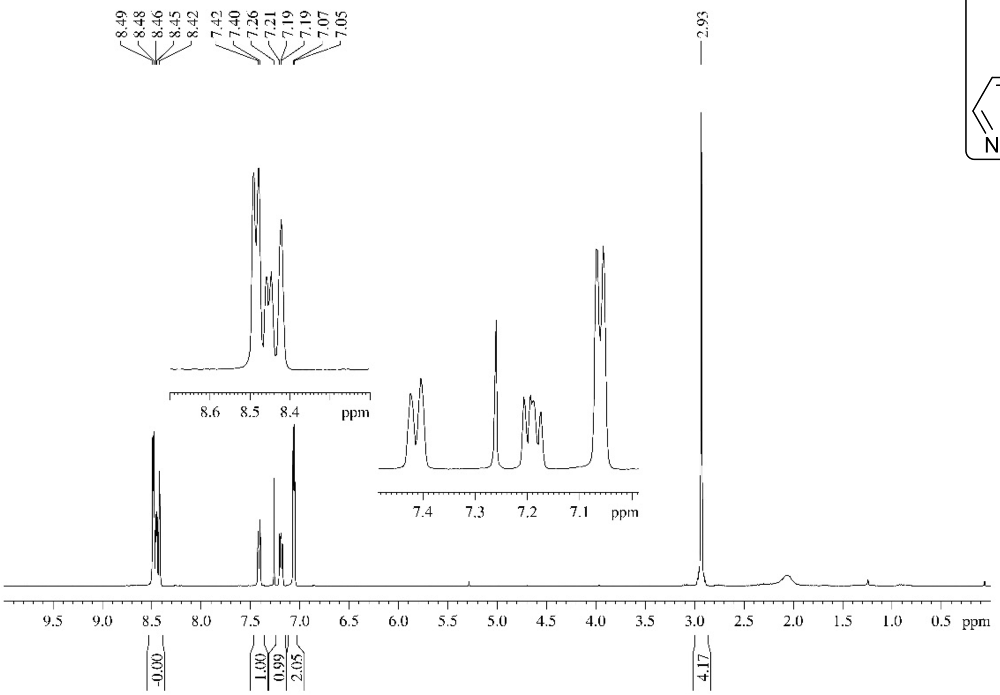

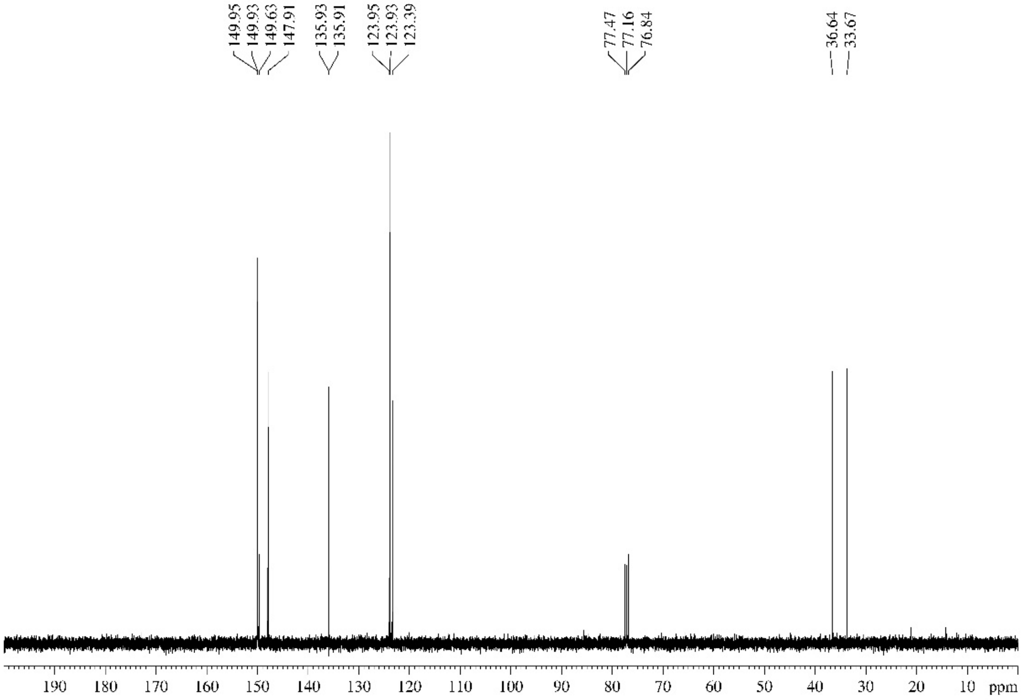

^{1}H (400 MHz, $CDCl_3$) and ^{13}C (101 MHz, $CDCl_3$) – NMR spectra of **28.1**

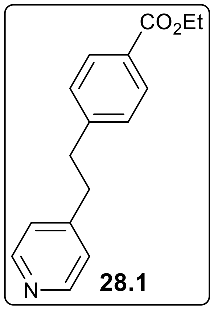

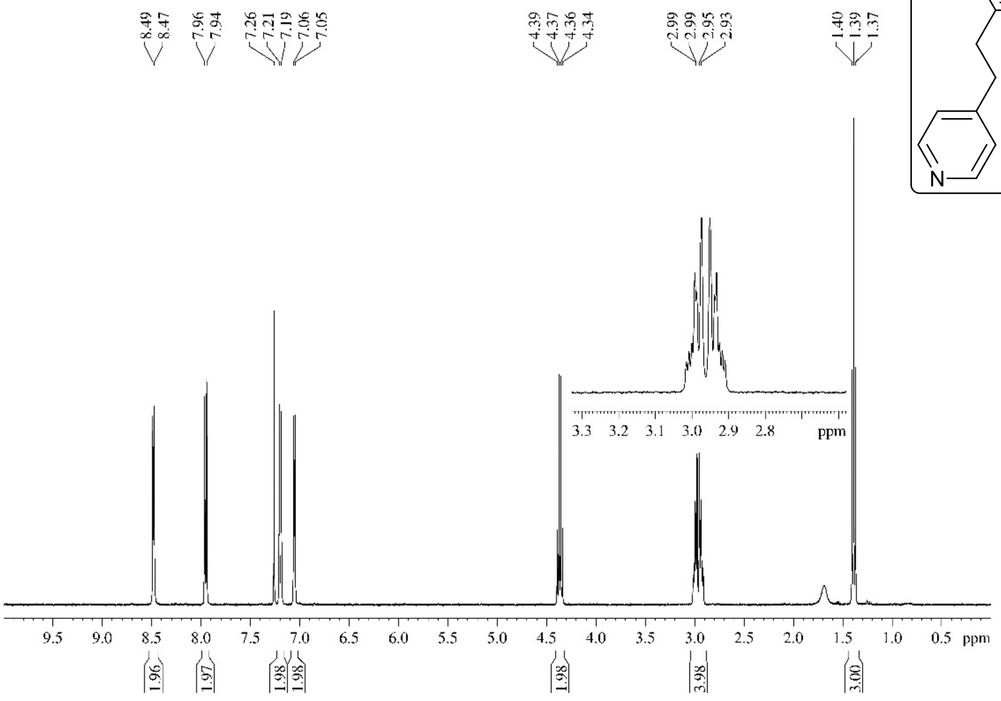

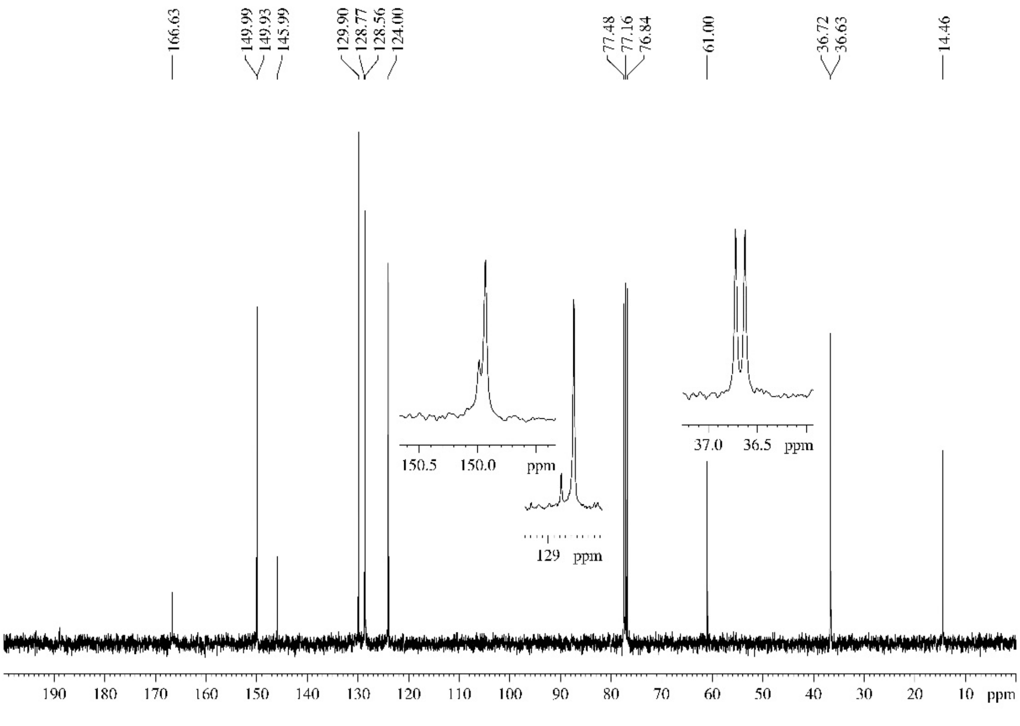

^{1}H (400 MHz, $CDCl_3$) and ^{13}C (101 MHz, $CDCl_3$) – NMR spectra of **28.2**

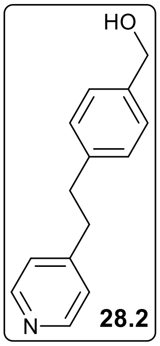

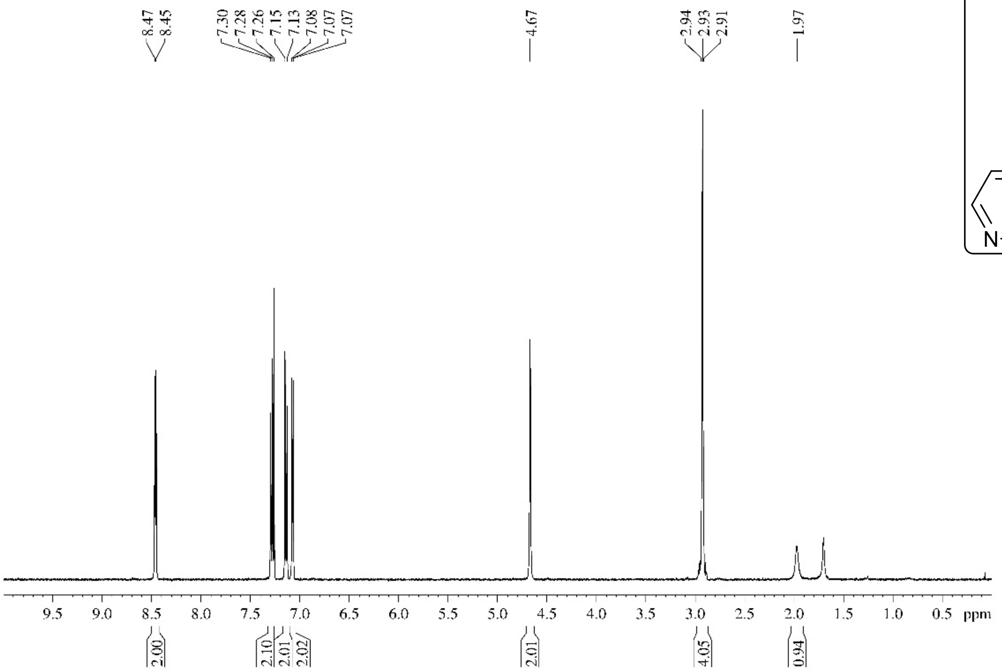

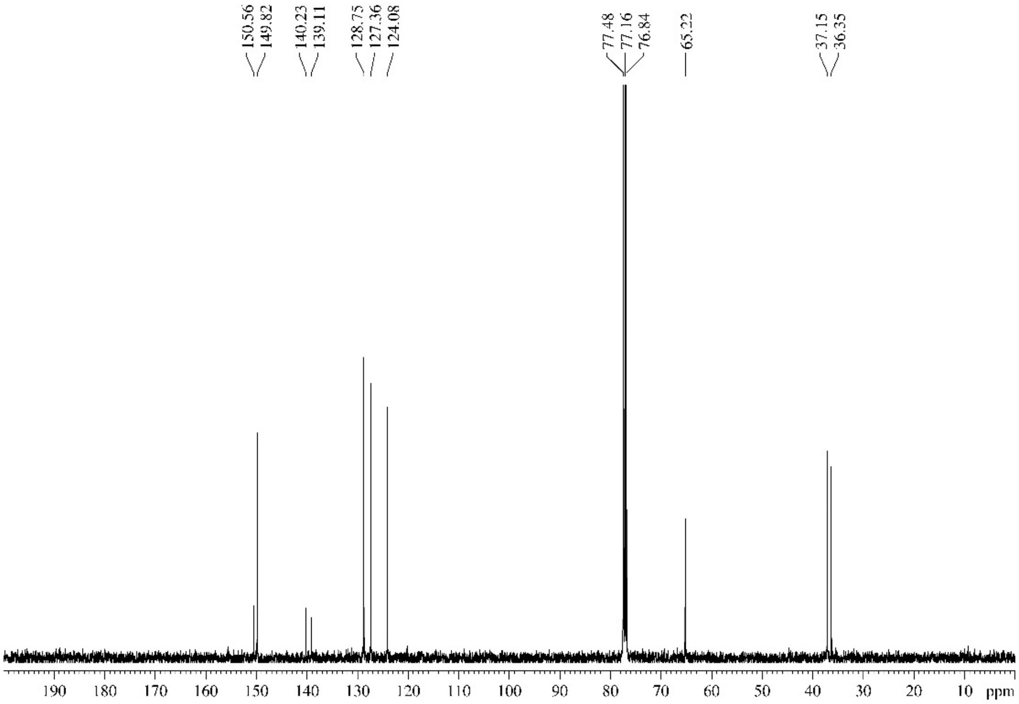

^{1}H (400 MHz, $CDCl_3$) and ^{13}C (101 MHz, $CDCl_3$) – NMR spectra of **28.3**

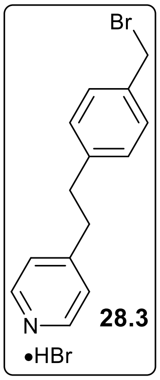

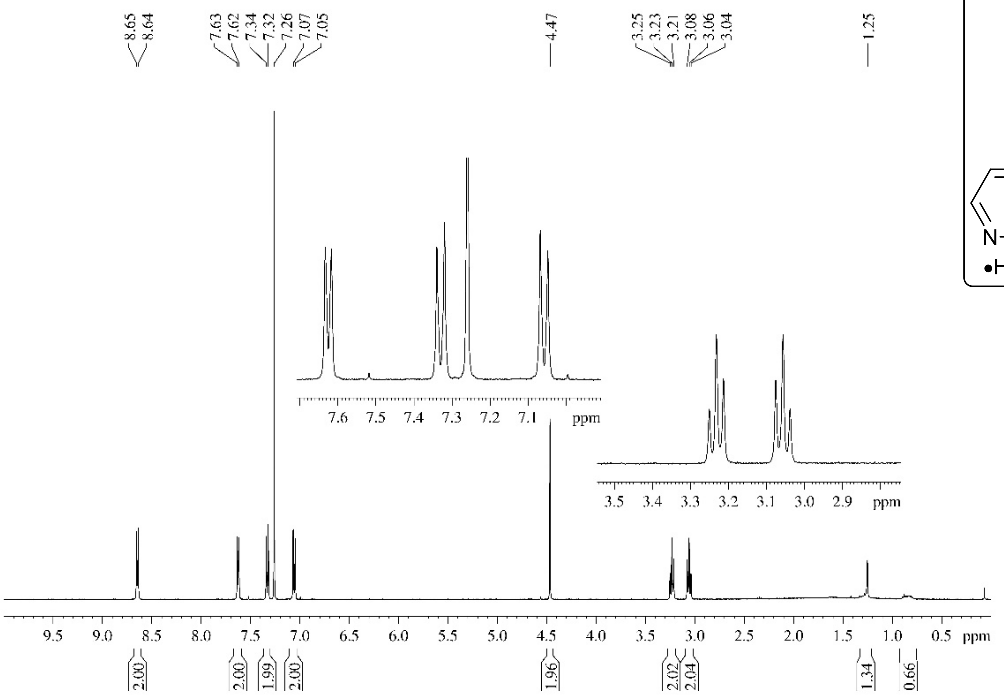

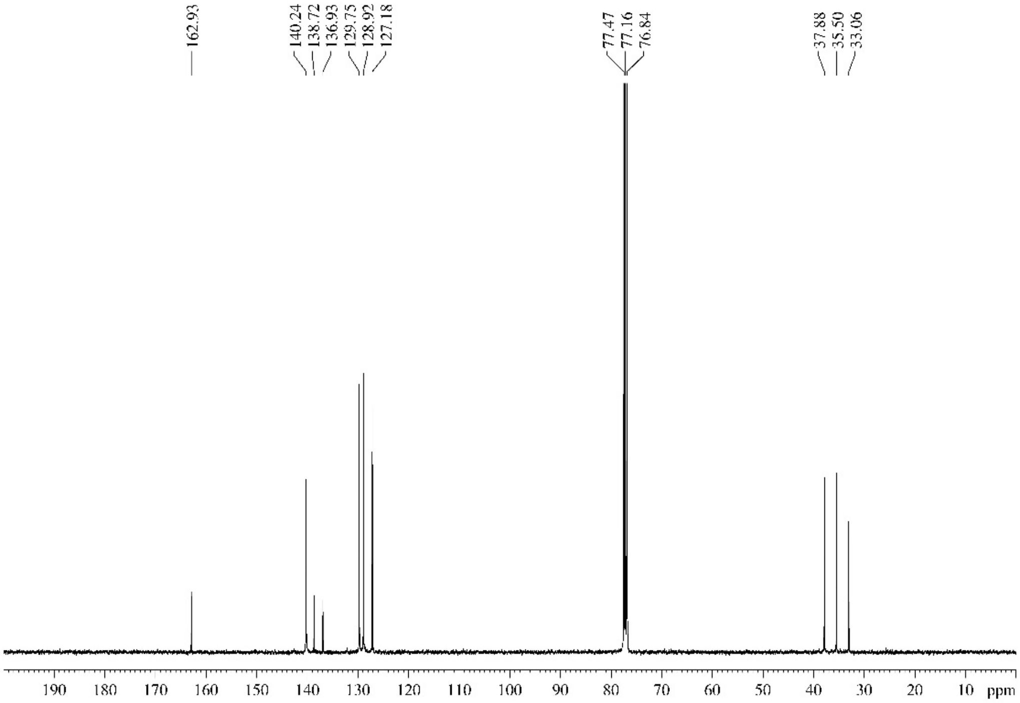

^{1}H (400 MHz, $CDCl_3$) and ^{13}C (101 MHz, $CDCl_3$) – NMR spectra of **28**

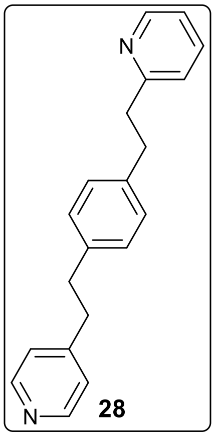

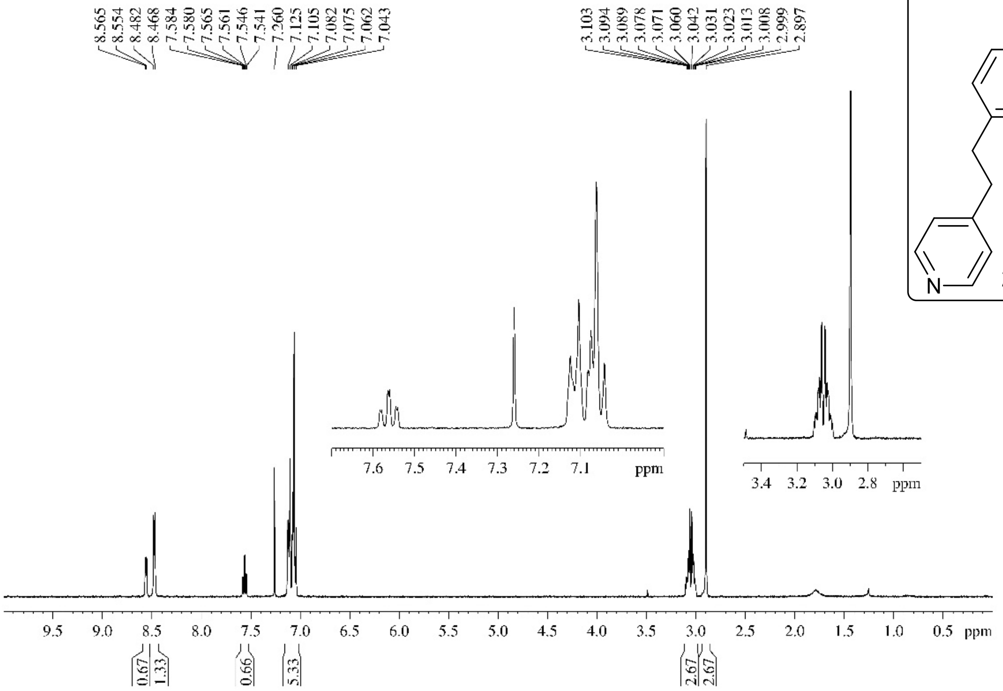

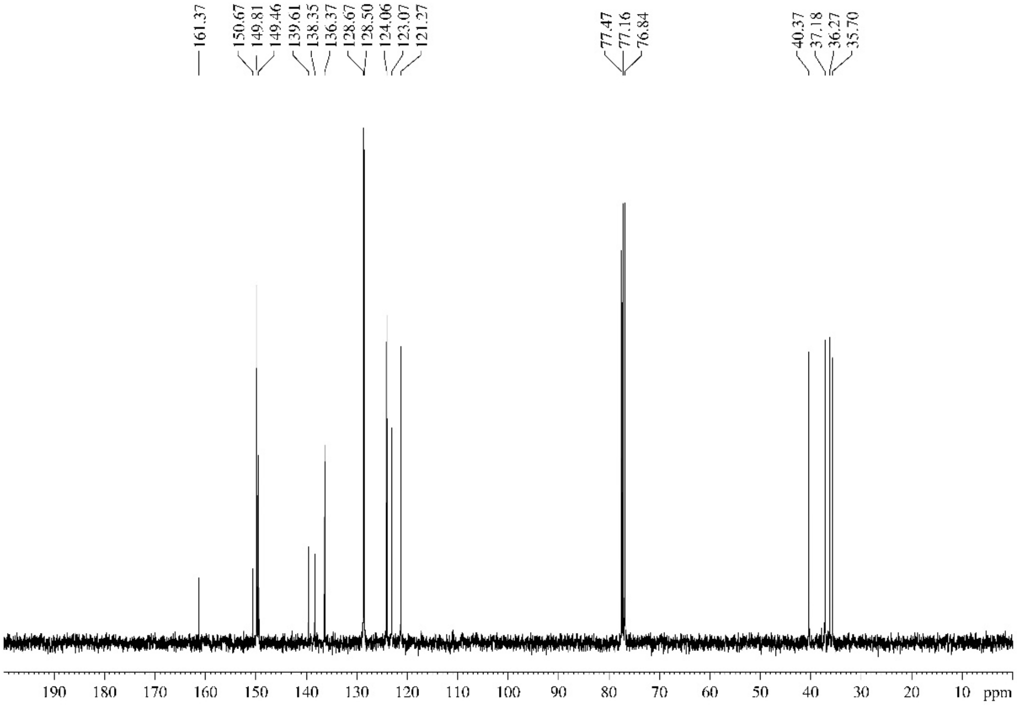

^{1}H (400 MHz, $CDCl_3$) and ^{13}C (101 MHz, $CDCl_3$) – NMR spectra of **29**

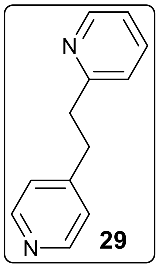

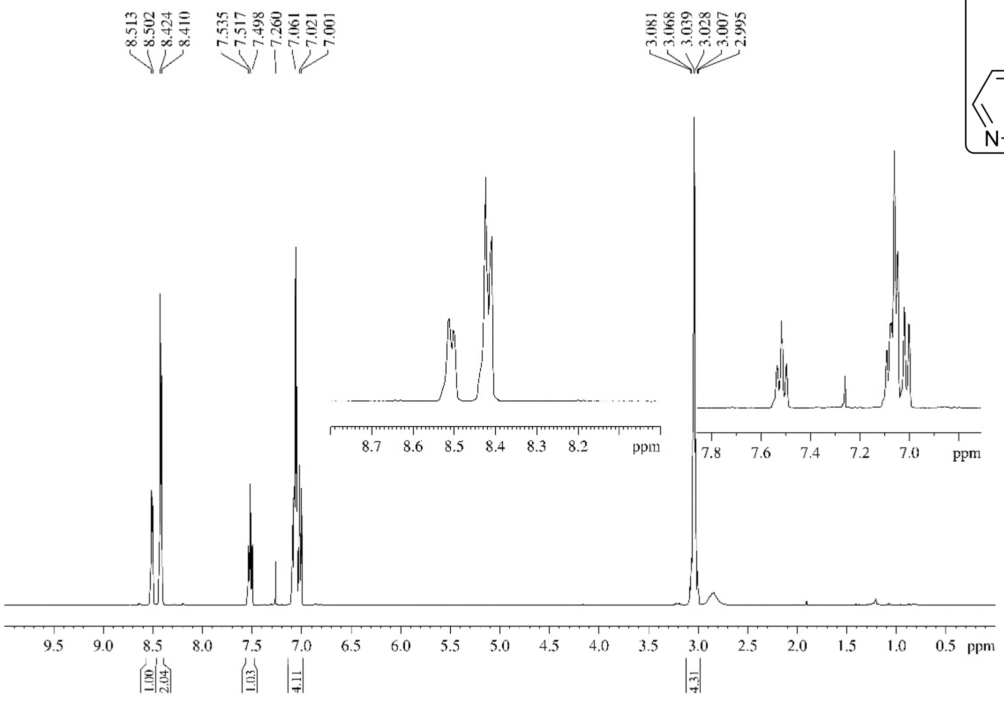

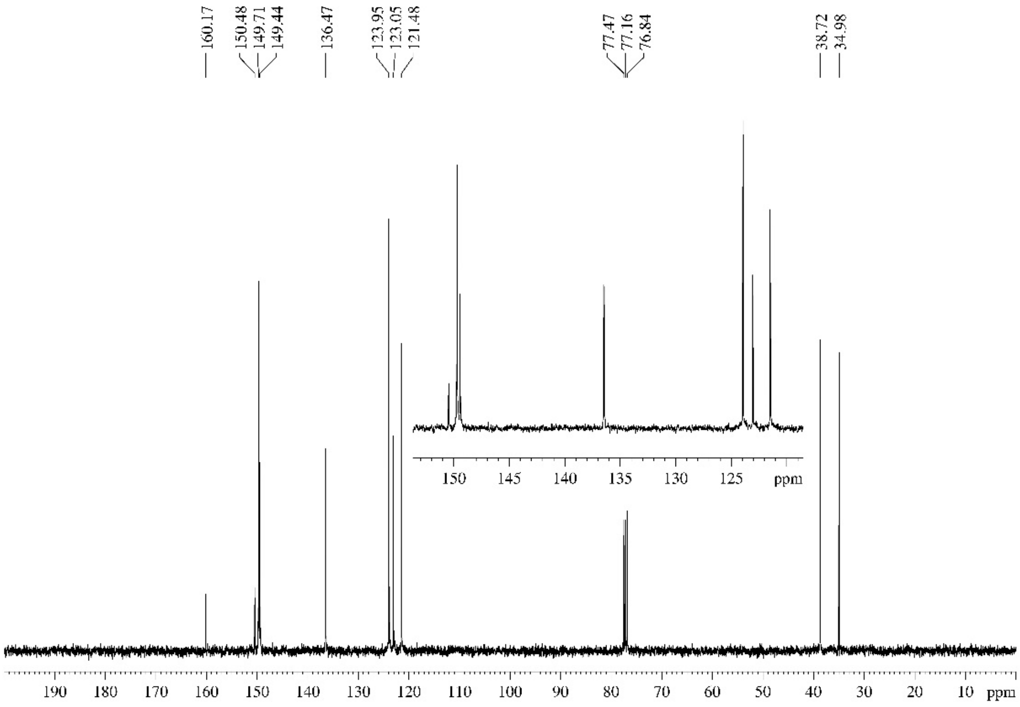

^{1}H (400 MHz, $CDCl_3$) and ^{13}C (76 MHz, $CDCl_3$) – NMR spectra of **31**

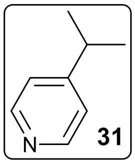

31

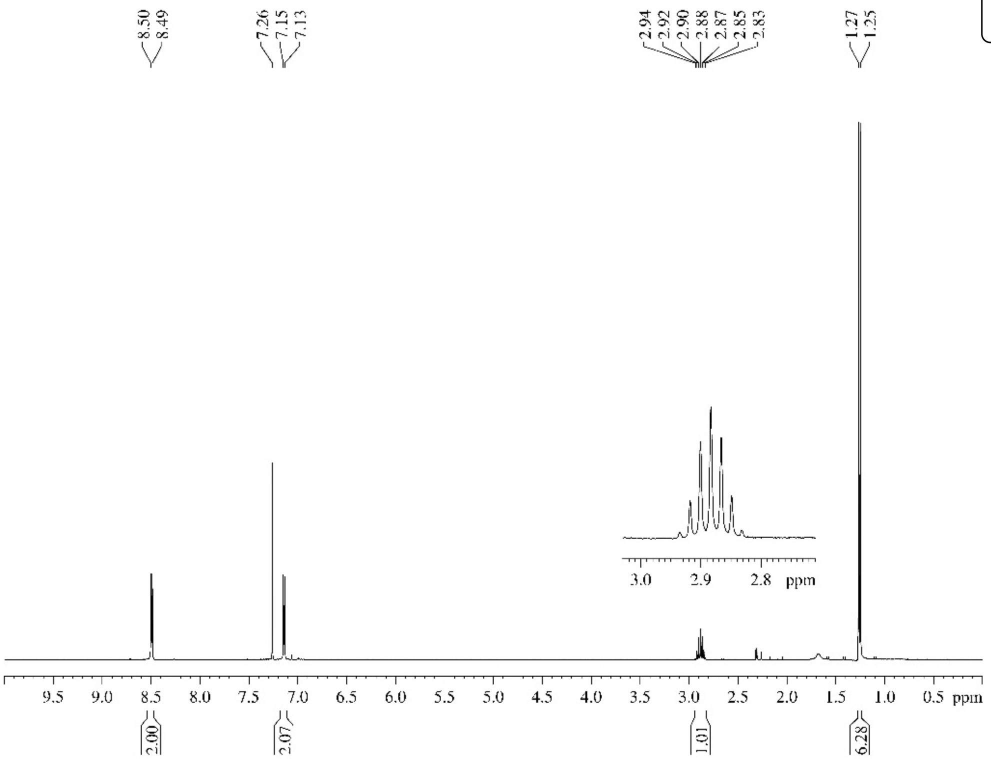

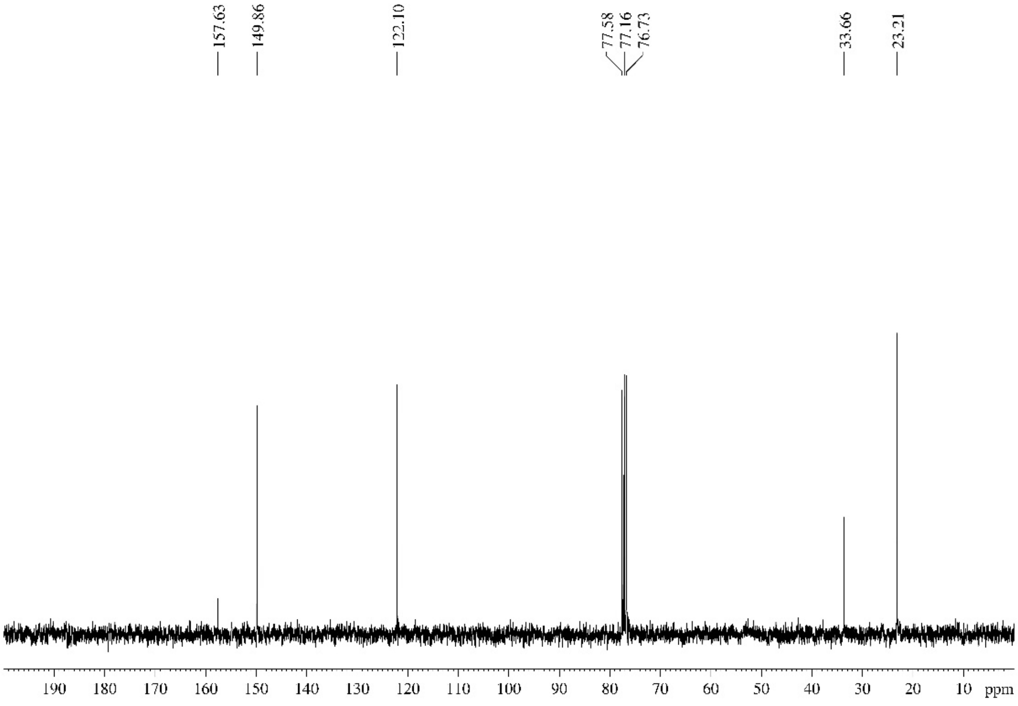

^{1}H (300 MHz, $CDCl_3$) and ^{13}C (101 MHz, $CDCl_3$) – NMR spectra of **32**

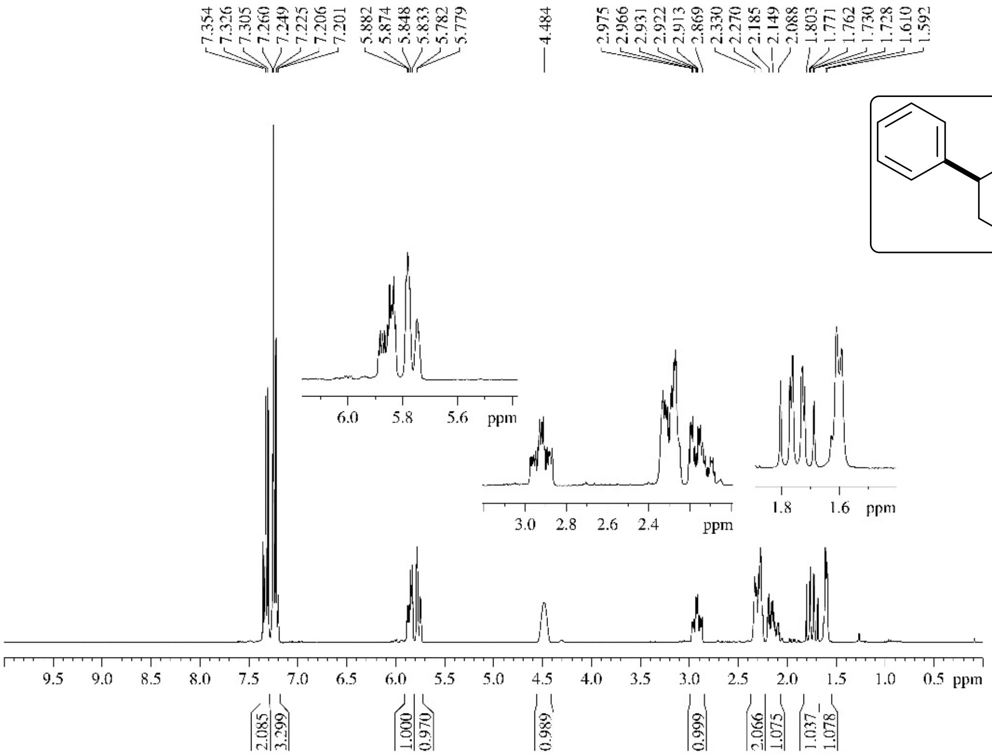

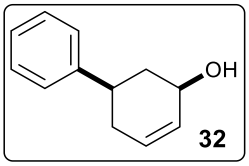

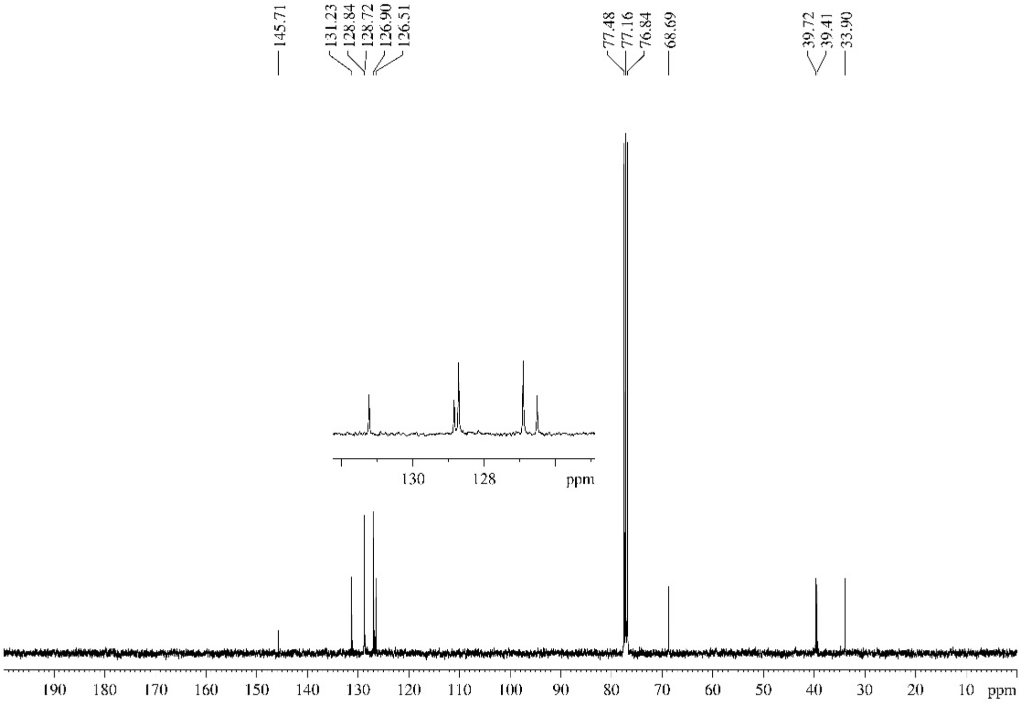

2D-COSY (600 MHz, $CDCl_3$) – NMR spectra of **32**

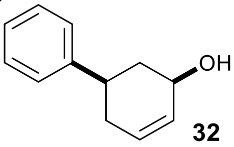

32

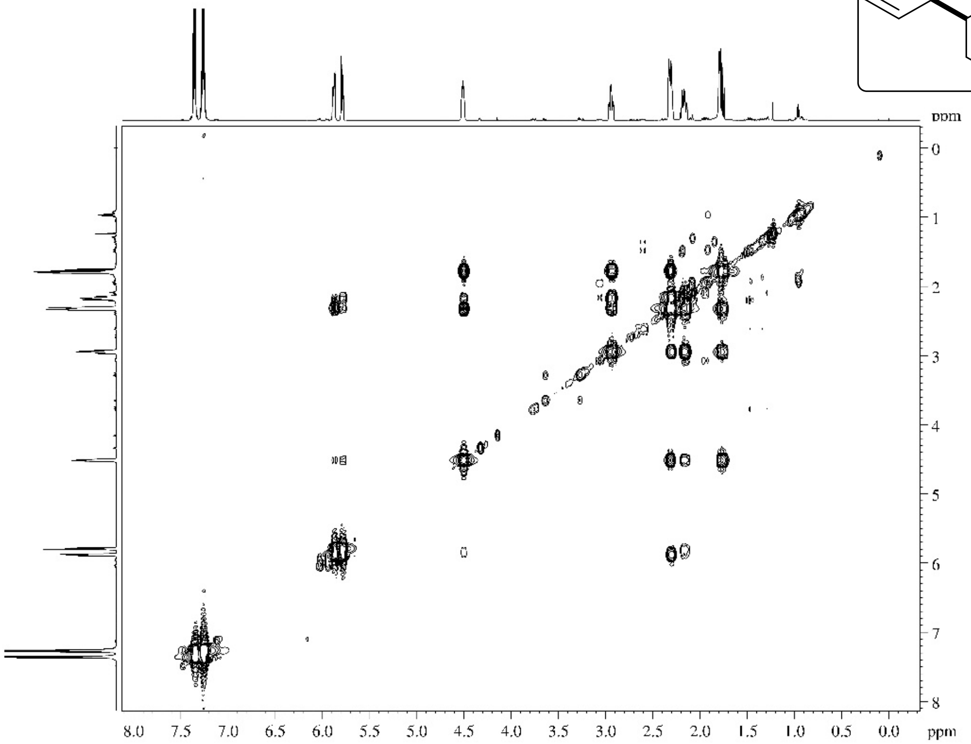

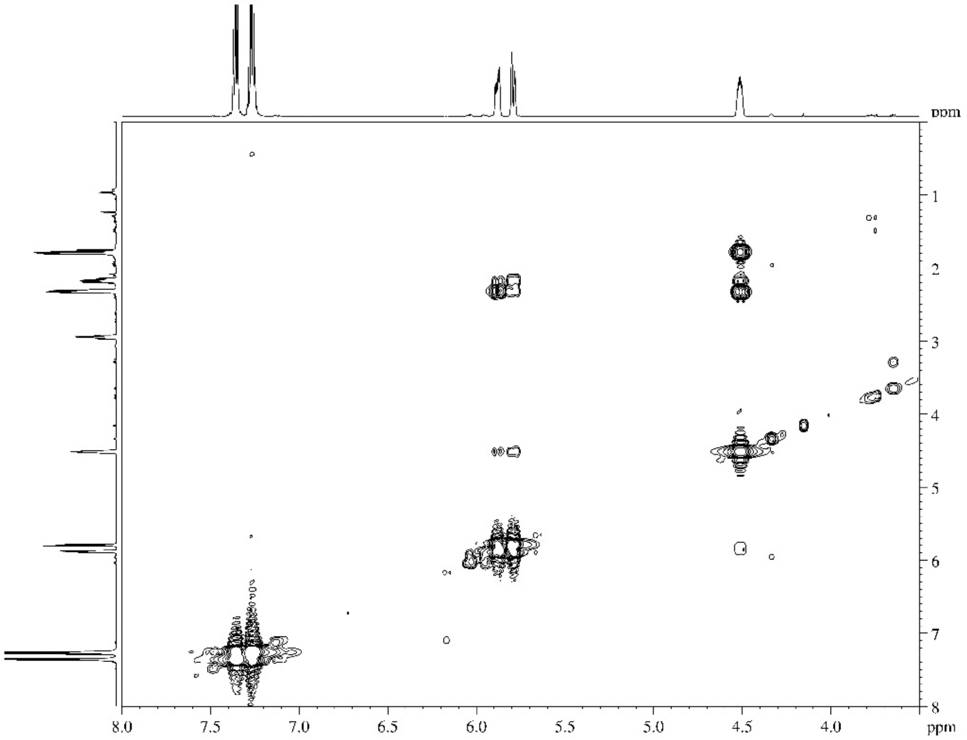

2D-COSY (600 MHz, $CDCl_3$) – NMR spectra of **32**

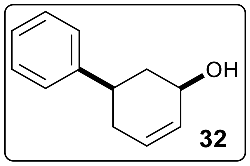

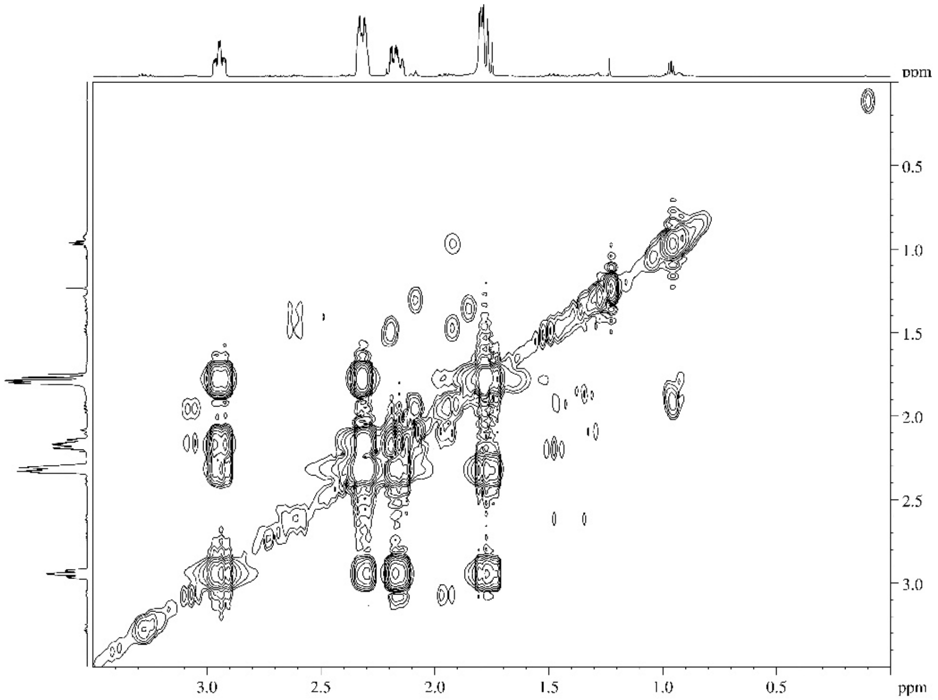

2D-NOESY (600 MHz, $CDCl_3$) – NMR spectra of **32**

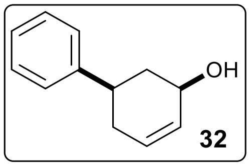

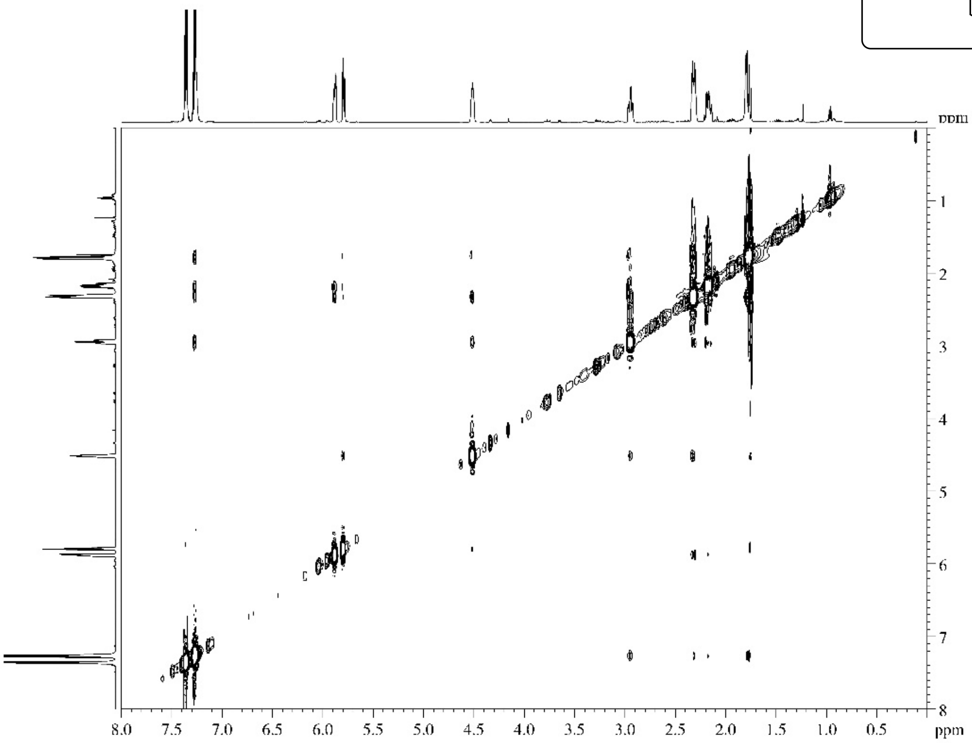

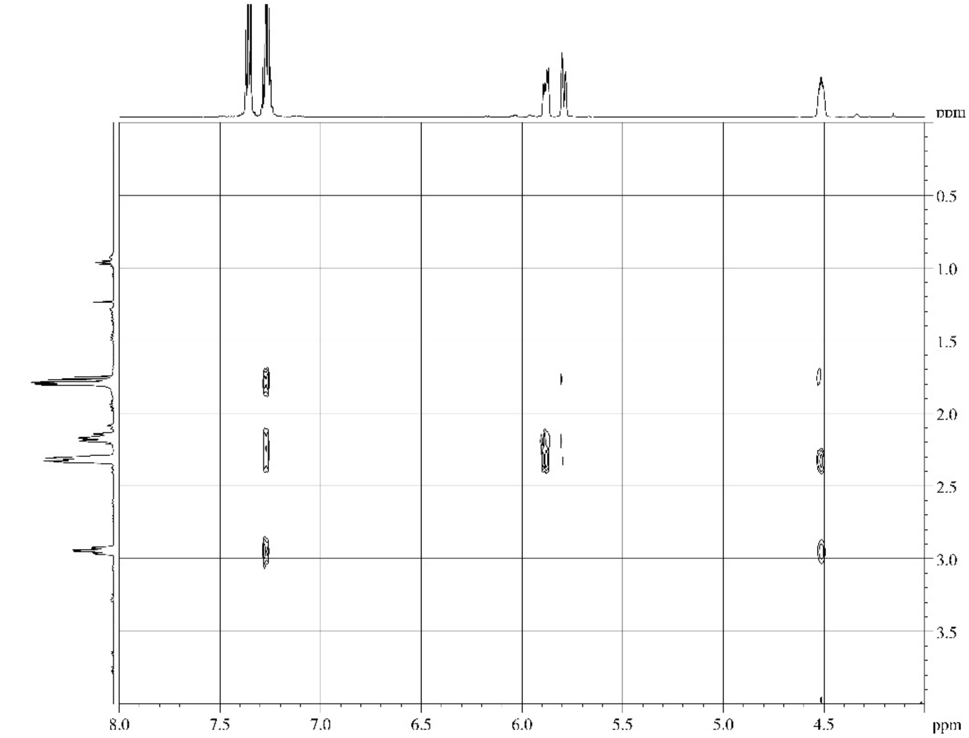

^{1}H (300 MHz, CD_2Cl_2) and ^{13}C (101 MHz, CD_2Cl_2) – NMR spectra of **33**

[(Xantphos)Pd(η3-allyl)]OTf
33

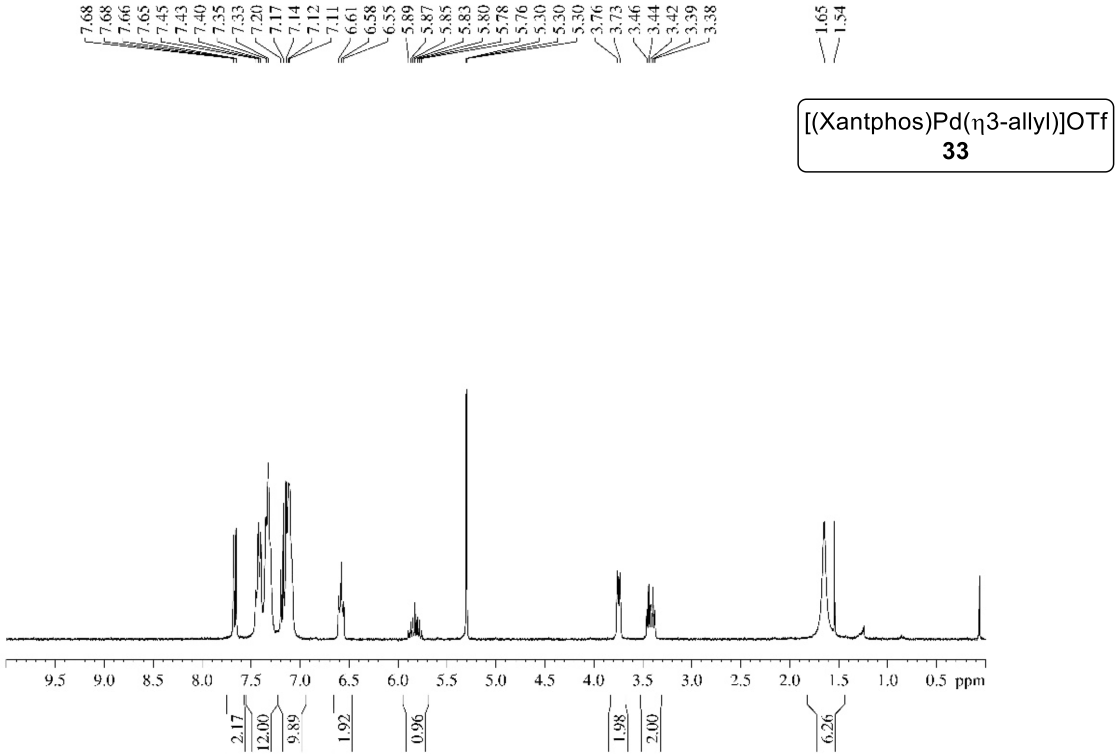

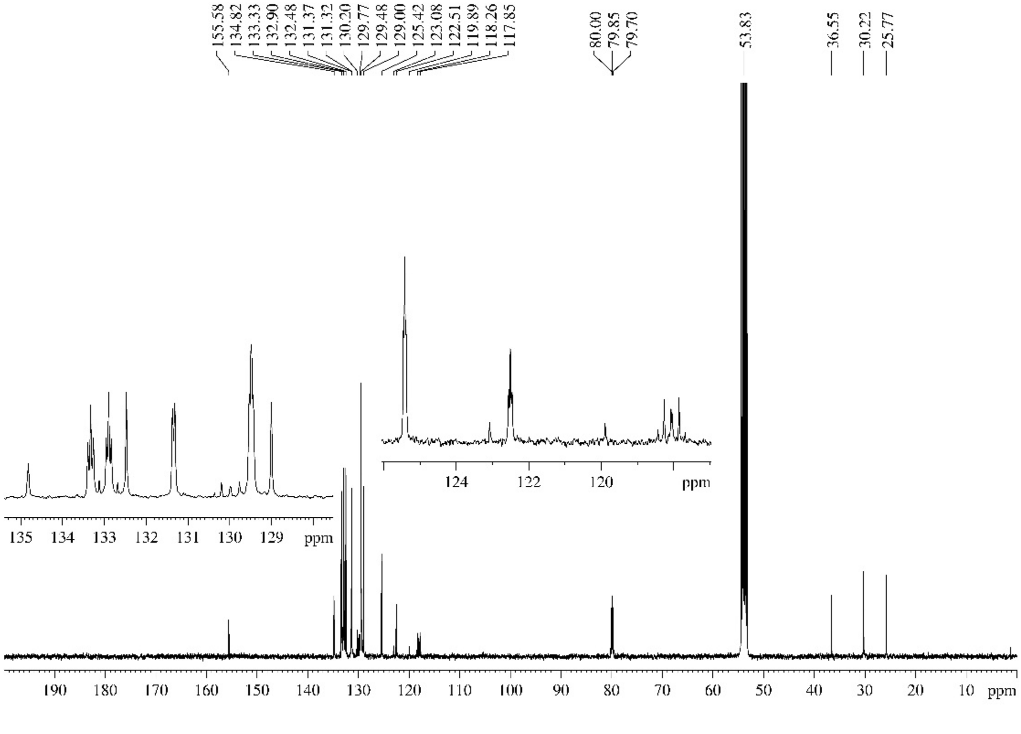

^{31}P (122 MHz, CD_2Cl_2) – NMR spectrum of **33**

[(Xantphos)Pd(η3-allyl)]OTf
33

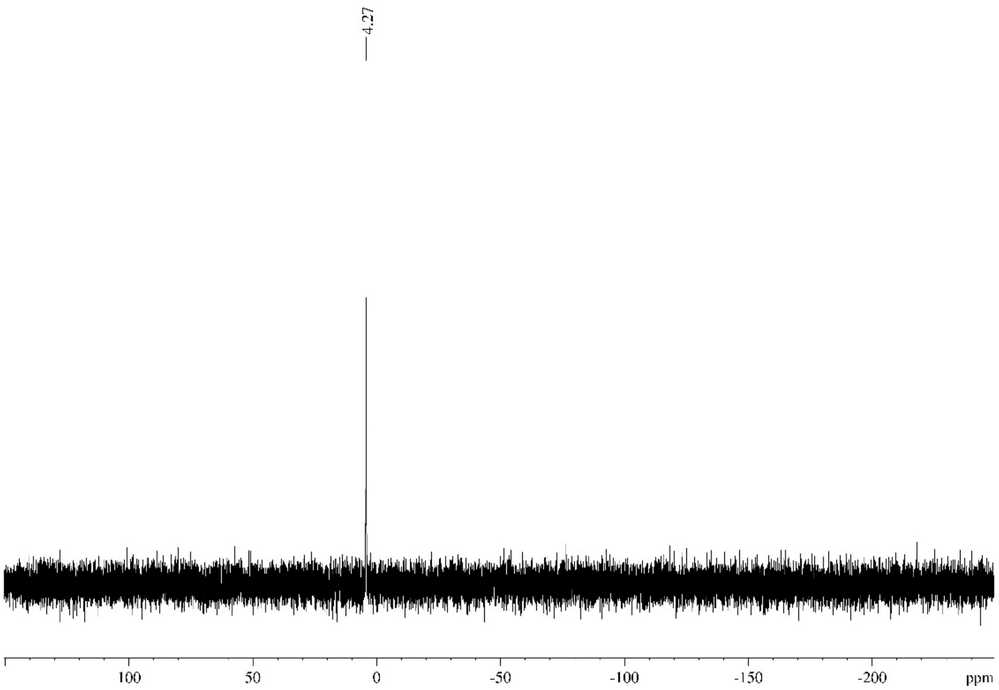

^{1}H (400 MHz, $CDCl_3$) and ^{13}C (76 MHz, $CDCl_3$) – NMR spectra of **34.1**

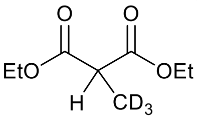

34.1

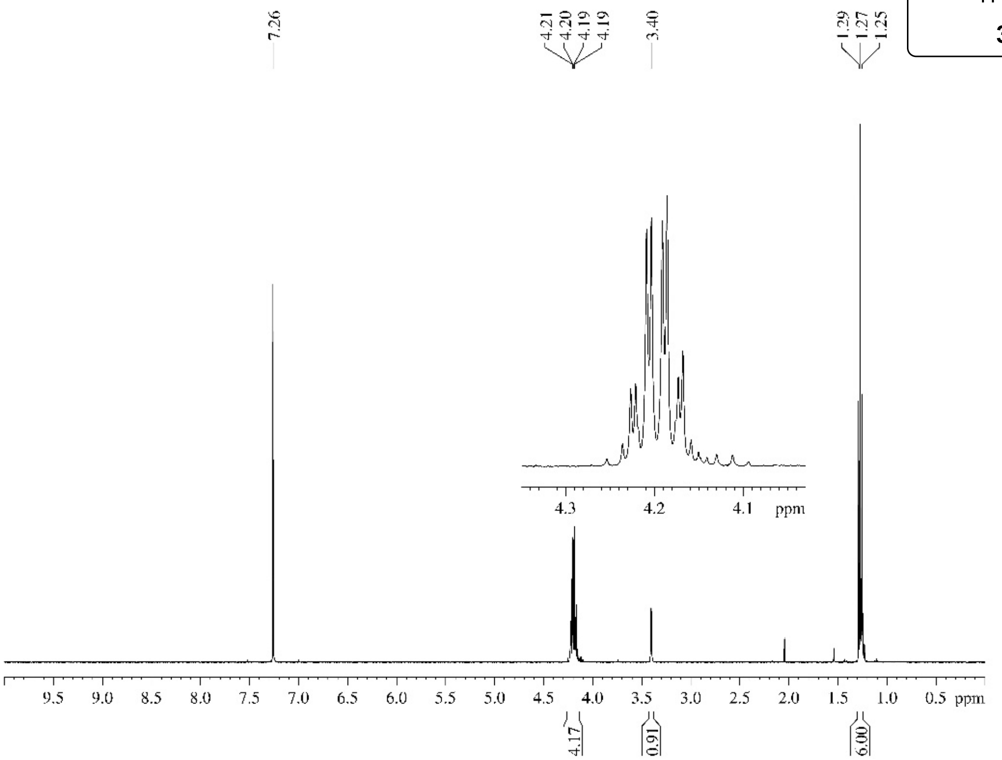

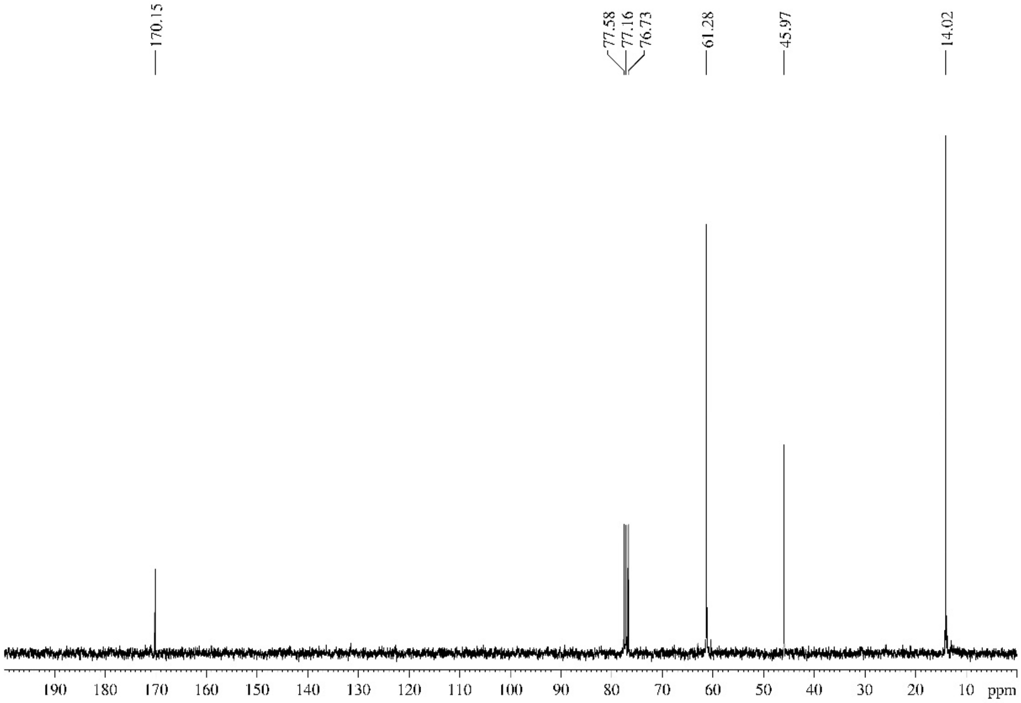

^{1}H (300 MHz, $CDCl_3$) and ^{2}H (46 MHz, $CDCl_3$) – NMR spectra of **34** as a solution in Et_2O

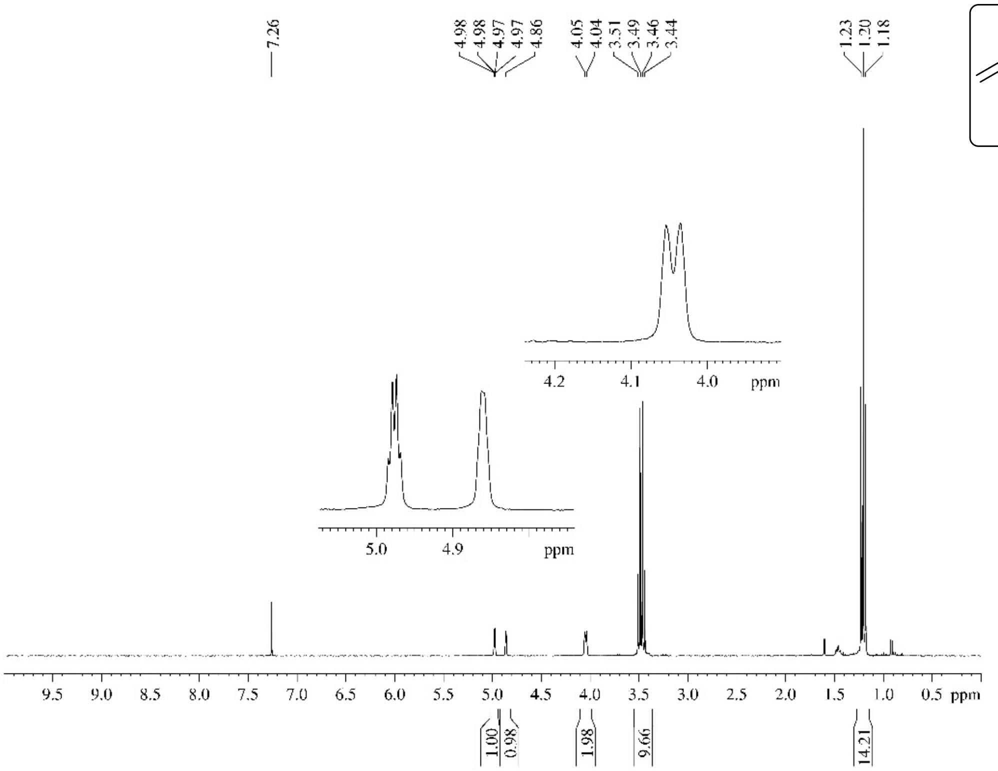

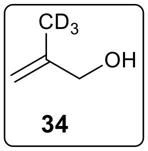

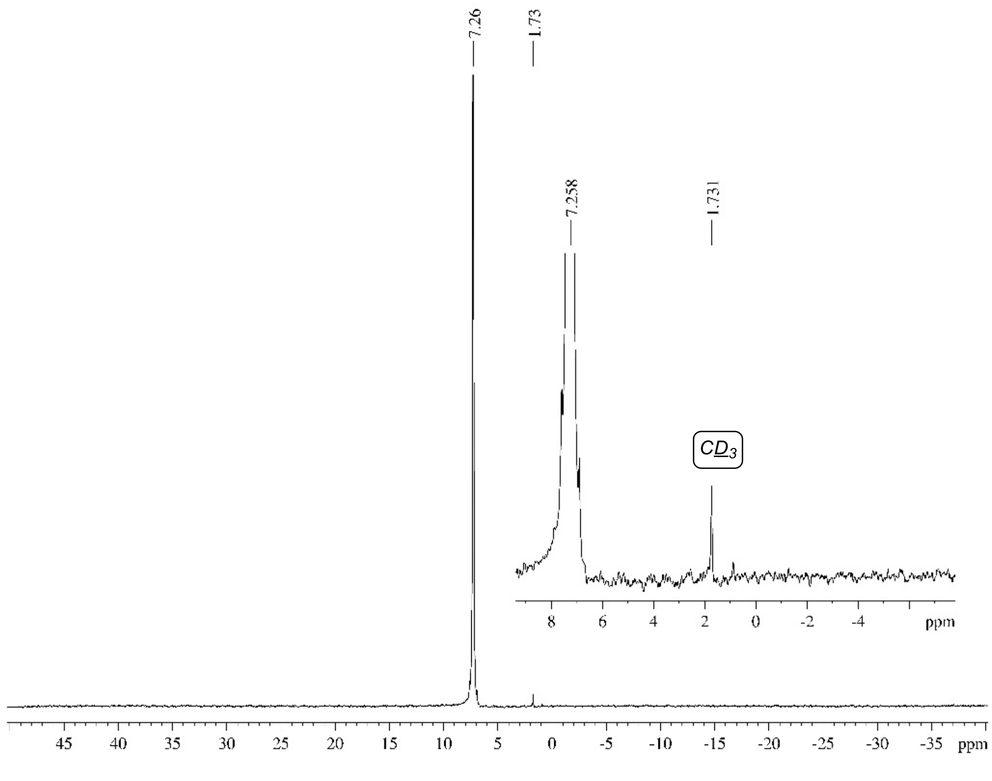

Diethyl ether peaks

^{13}C (151 MHz, $CDCl_3$) – NMR spectra of **34** as a solution in Et_2O

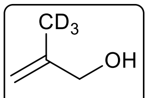

34

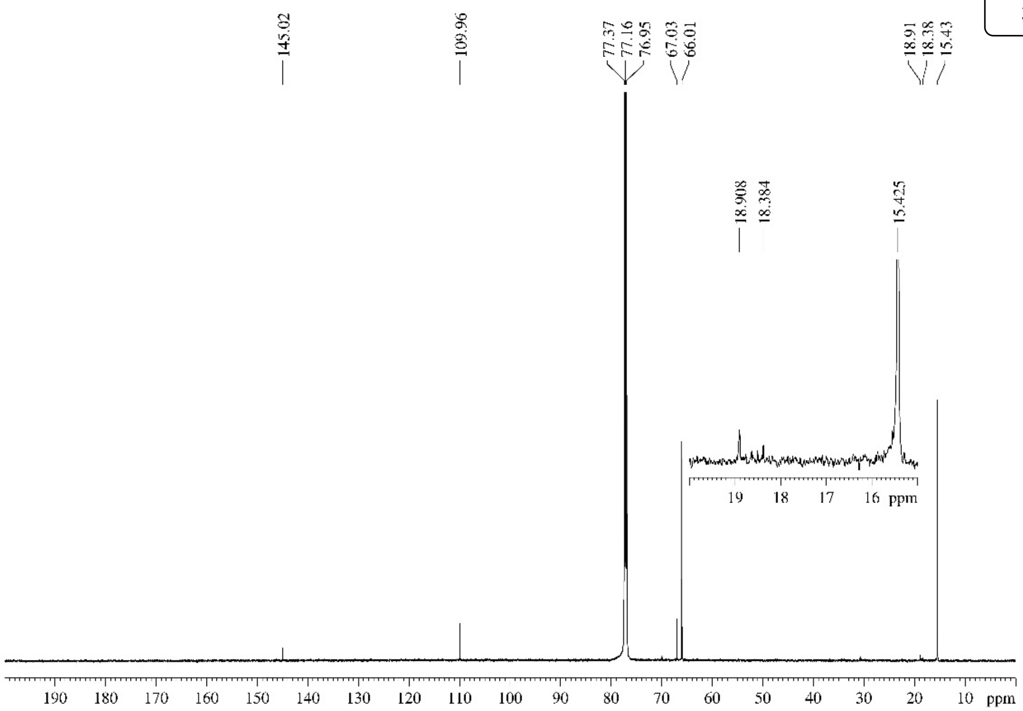

Diethyl ether peaks

2D-HMBC (600 MHz, $CDCl_3$) – NMR spectra of **34** as a solution in Et_2O

The $\underline{C}D_3$ *signal at 18.3 ppm correlated with the allyl & vinyl protons as shown by HMBC.*

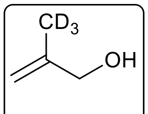

34

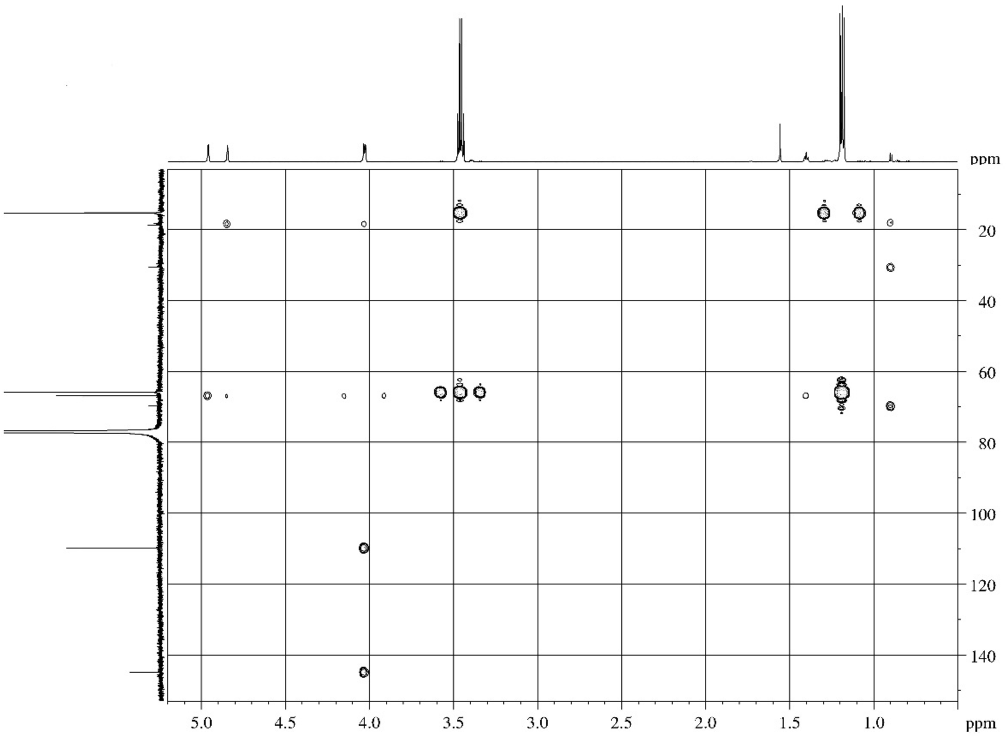

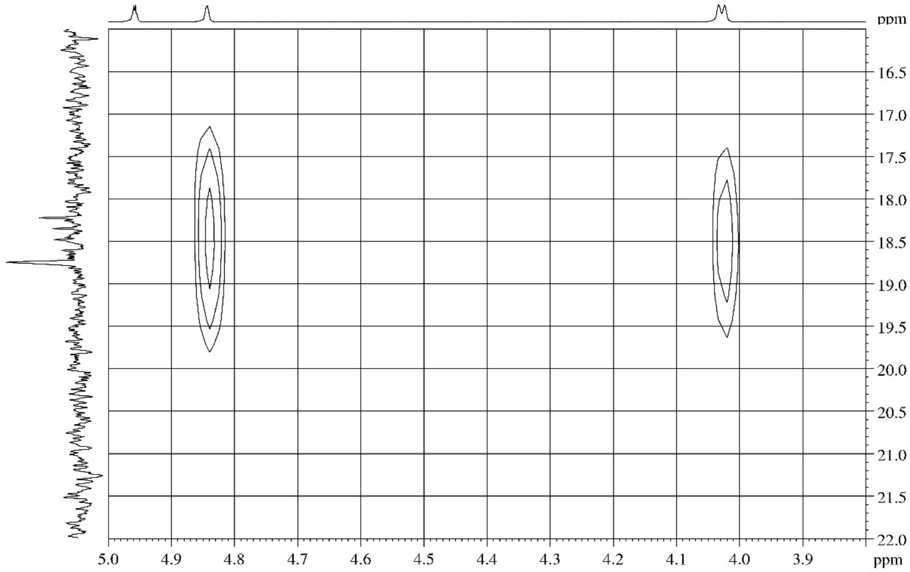

^{1}H (300 MHz, $CDCl_3$) and ^{13}C (76 MHz, $CDCl_3$) – NMR spectra of **L1**

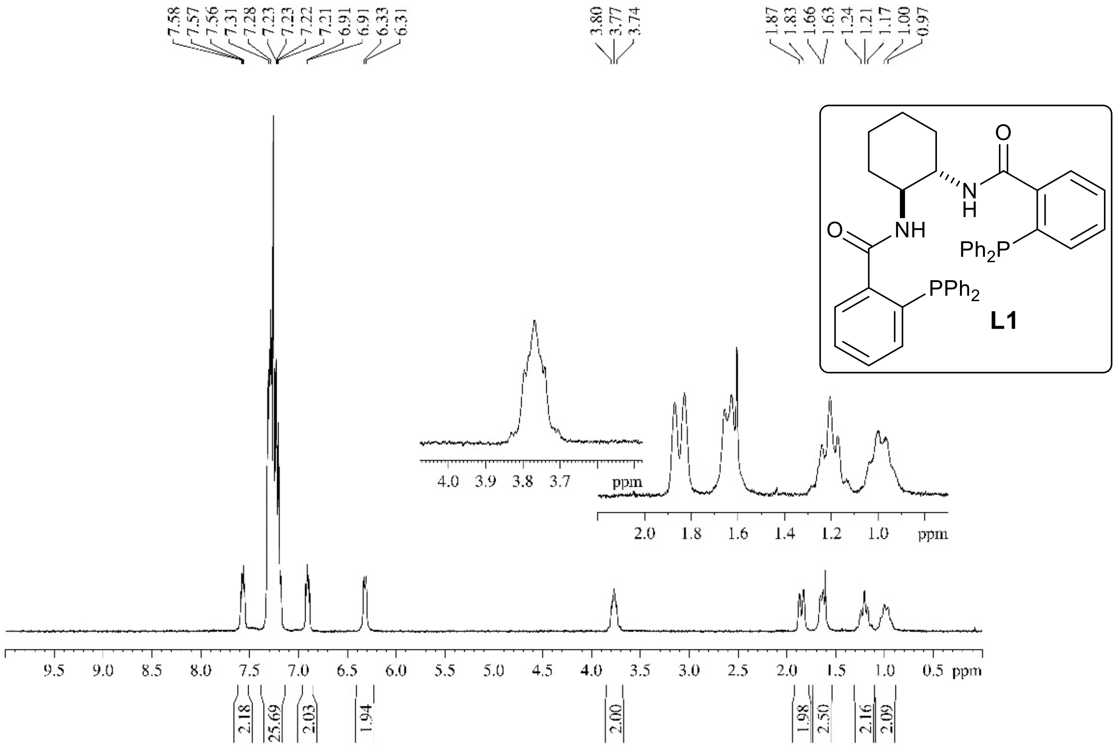

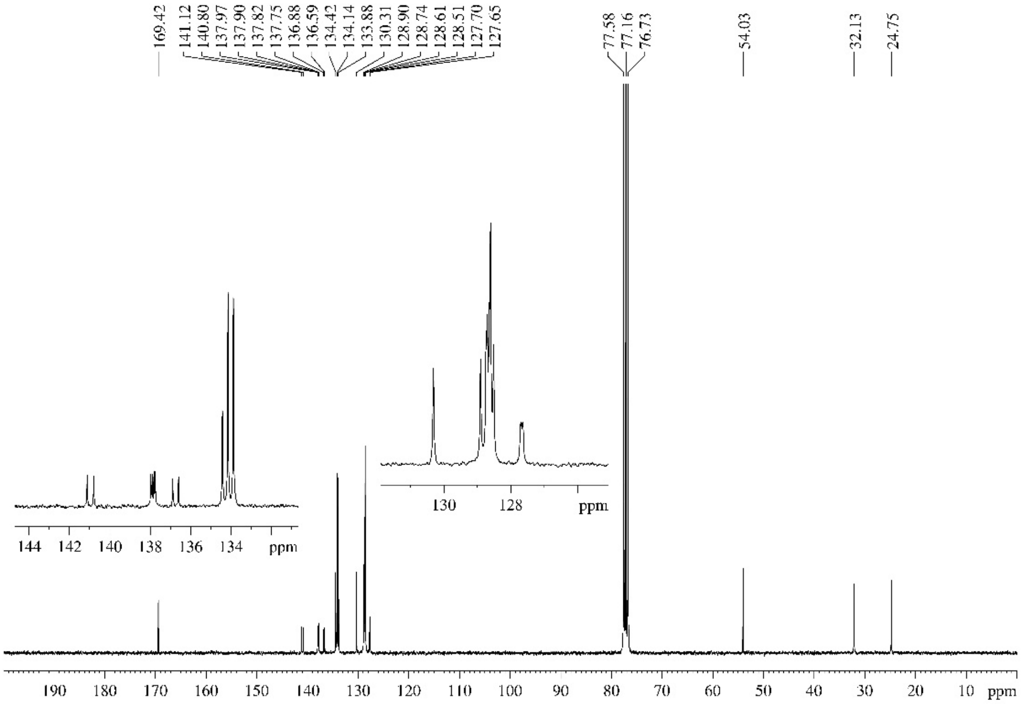

^{1}H (400 MHz, $CDCl_3$) and ^{13}C (101 MHz, $CDCl_3$) – NMR spectra of **L2**

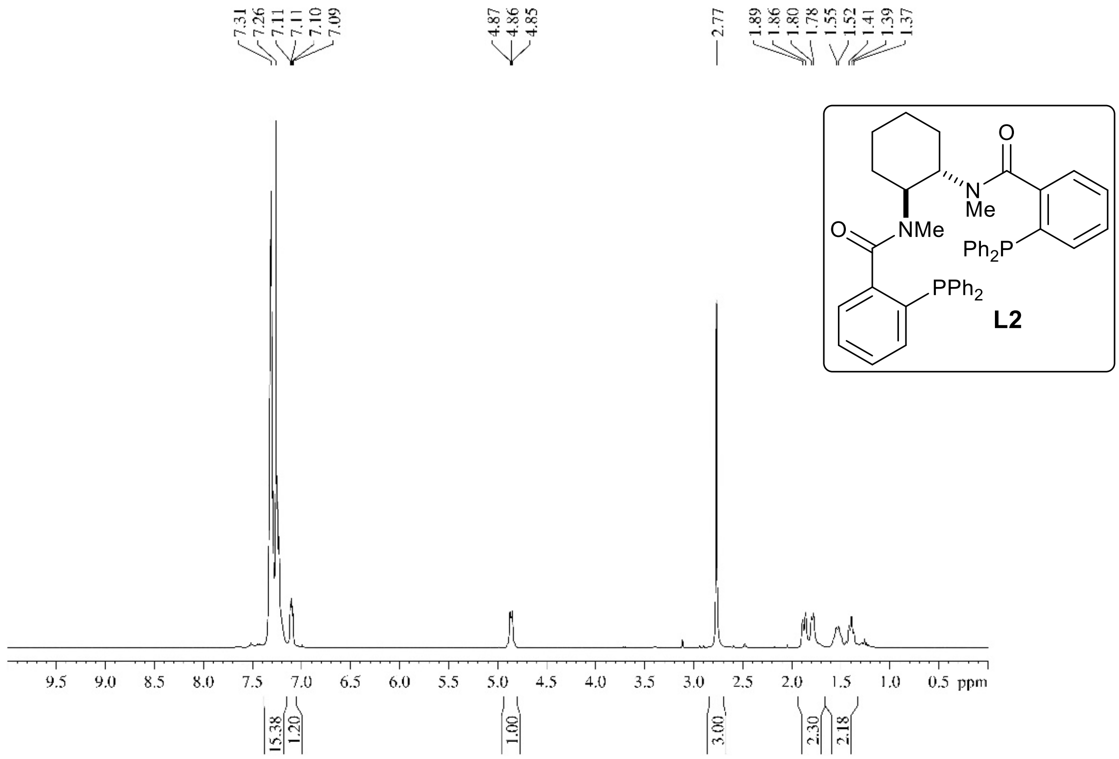

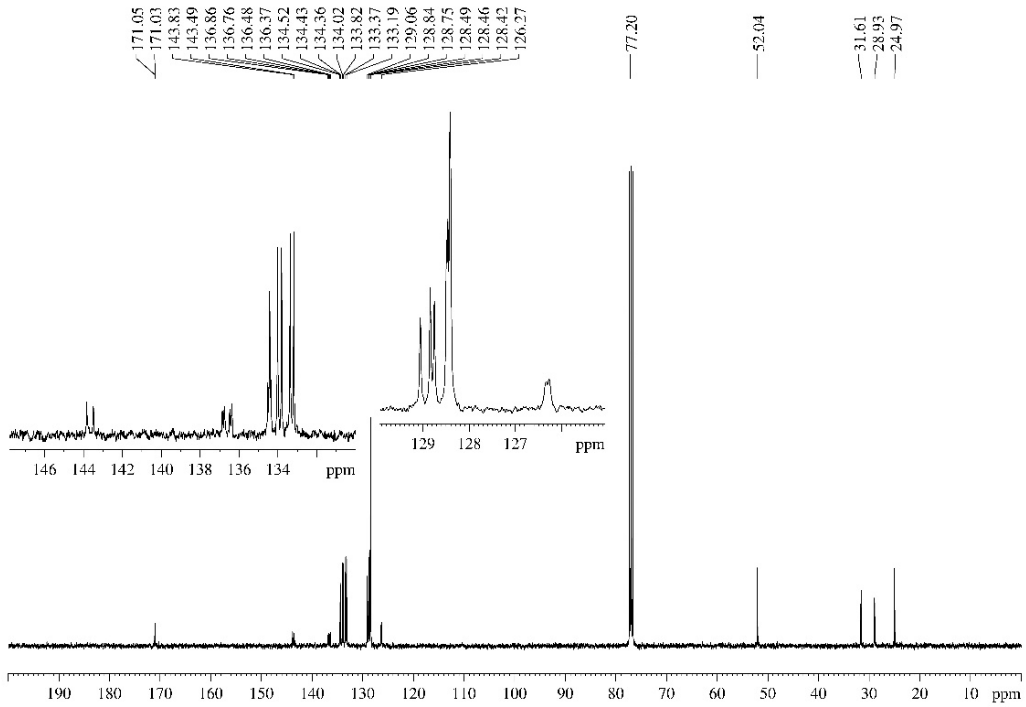

^{1}H (300 MHz, $CDCl_3$, 25 °C) and ^{1}H (300 MHz, $CDCl_3$, 45 °C) – NMR spectra of **1’**

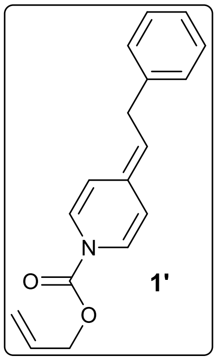

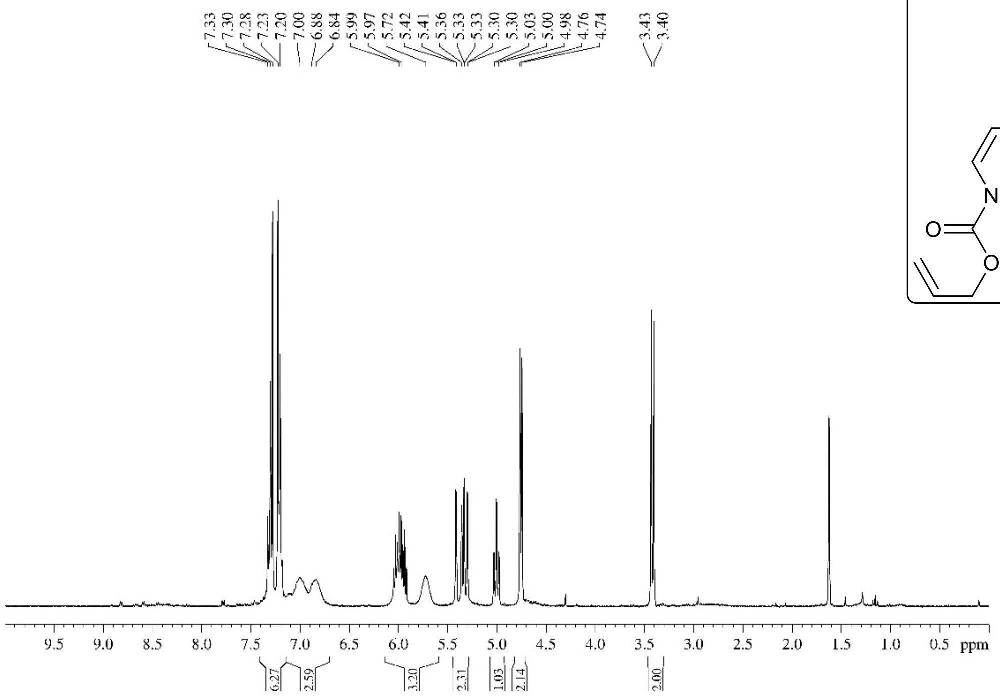

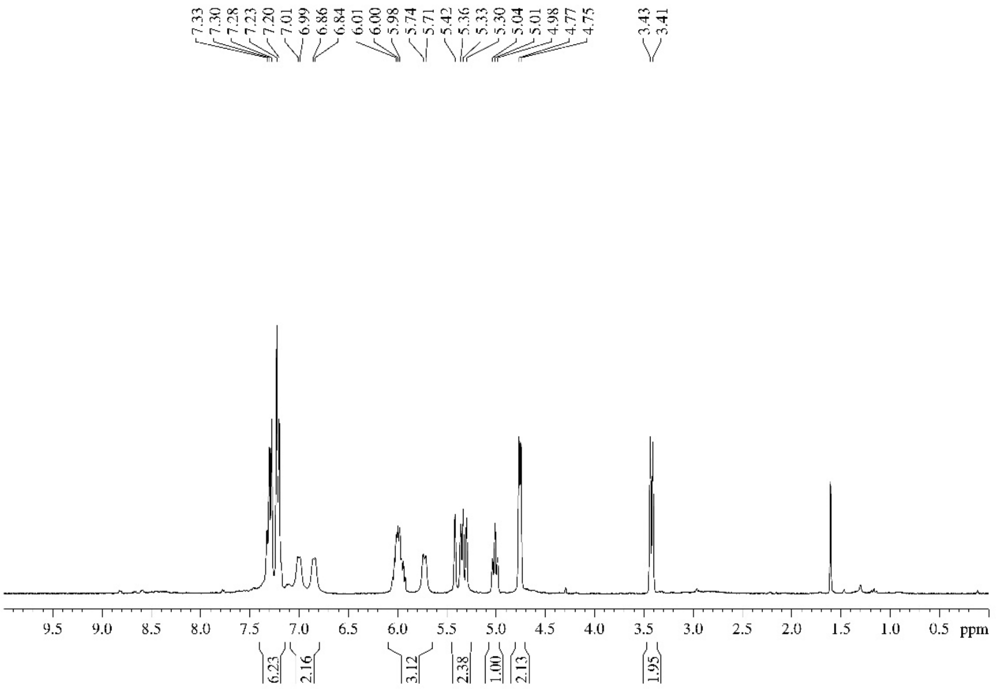

^{13}C (76 MHz, $CDCl_3$, 52 °C) – NMR spectrum of **1’**

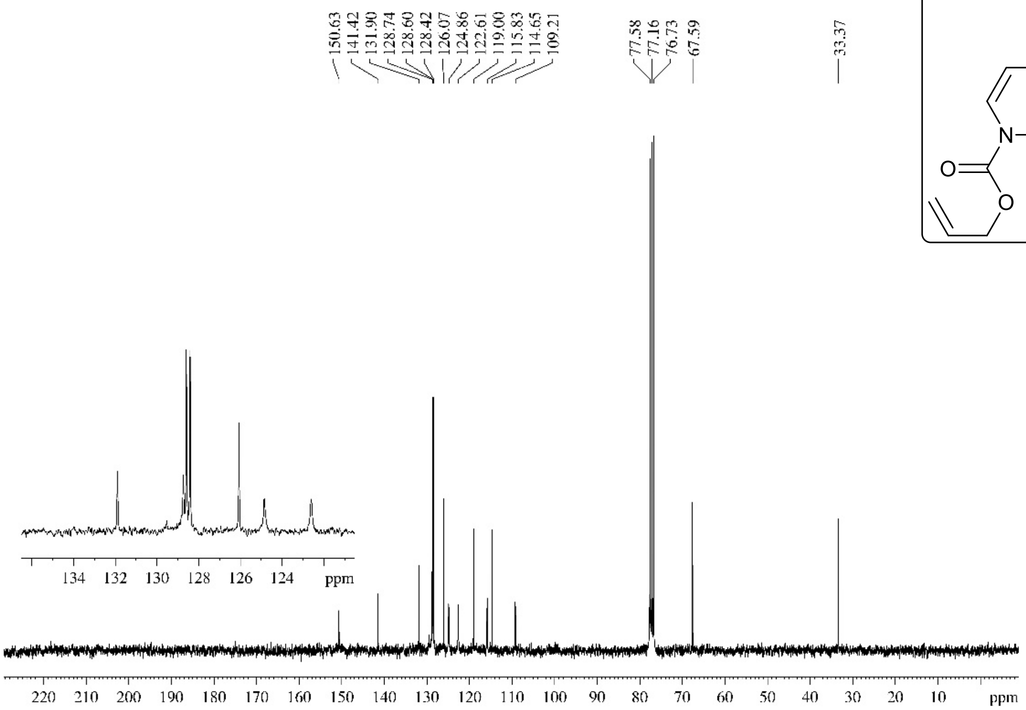

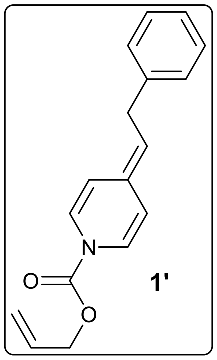

1'

^{1}H (400 MHz, $CDCl_3$) – NMR spectra of **1''**

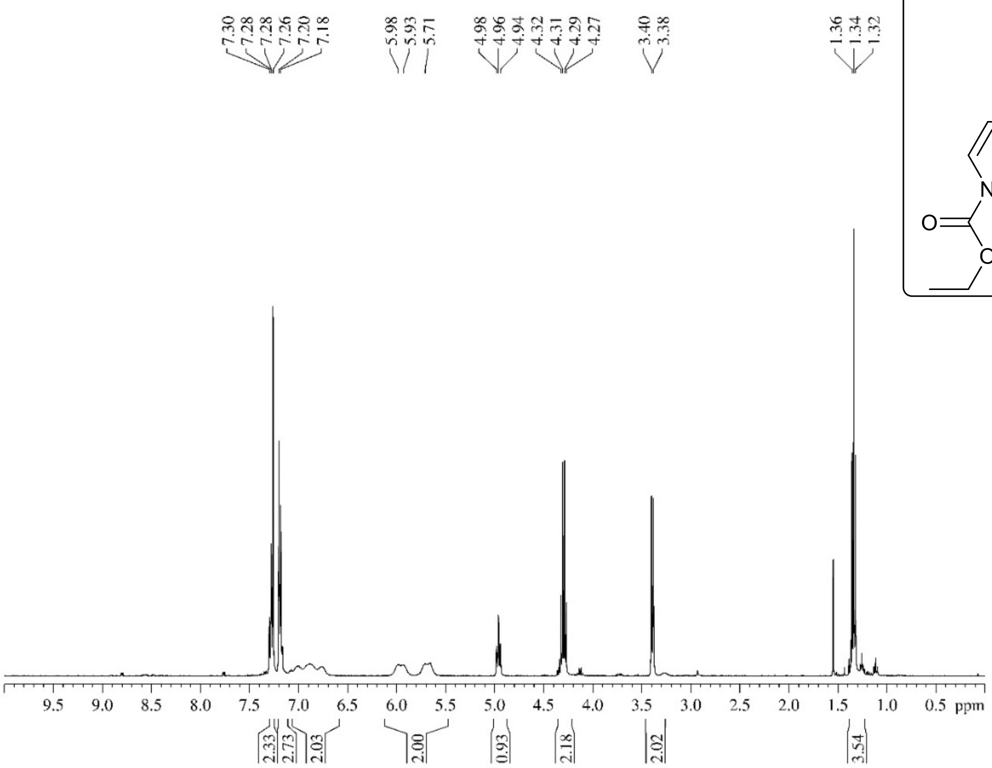

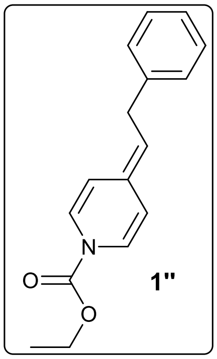

1''

^{1}H (400 MHz, $CDCl_3$) and ^{13}C (101 MHz, $CDCl_3$) – NMR spectra of **1A**

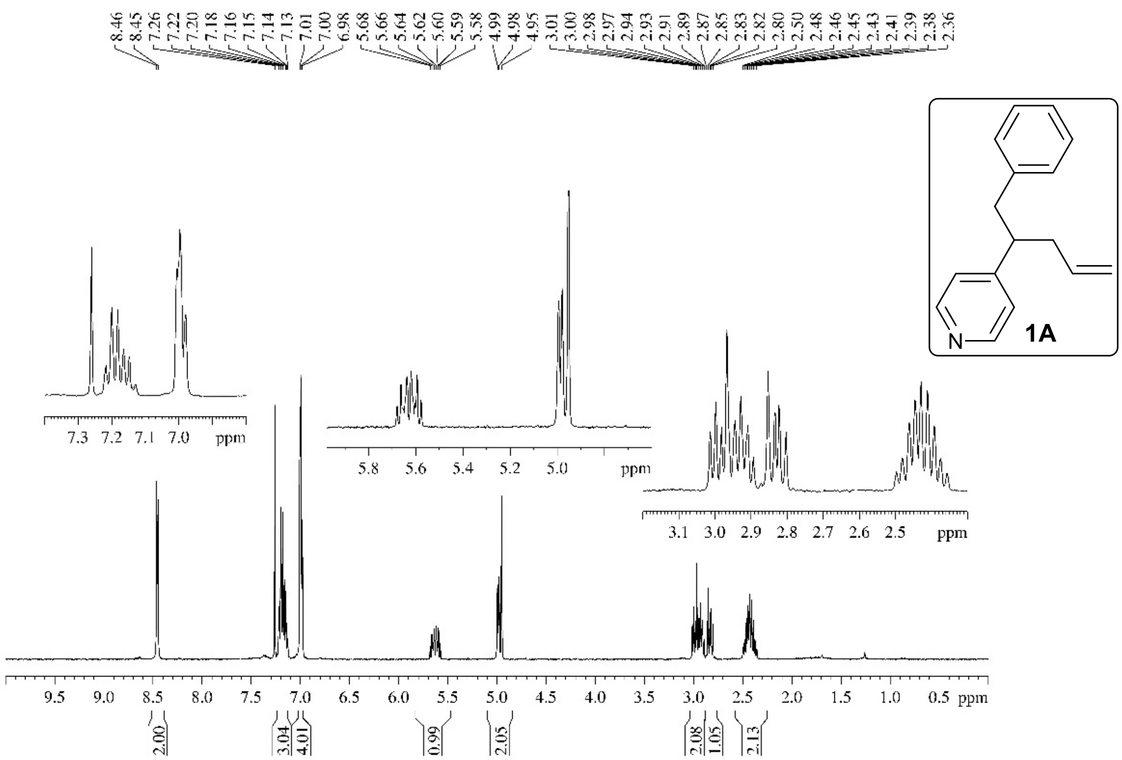

1A

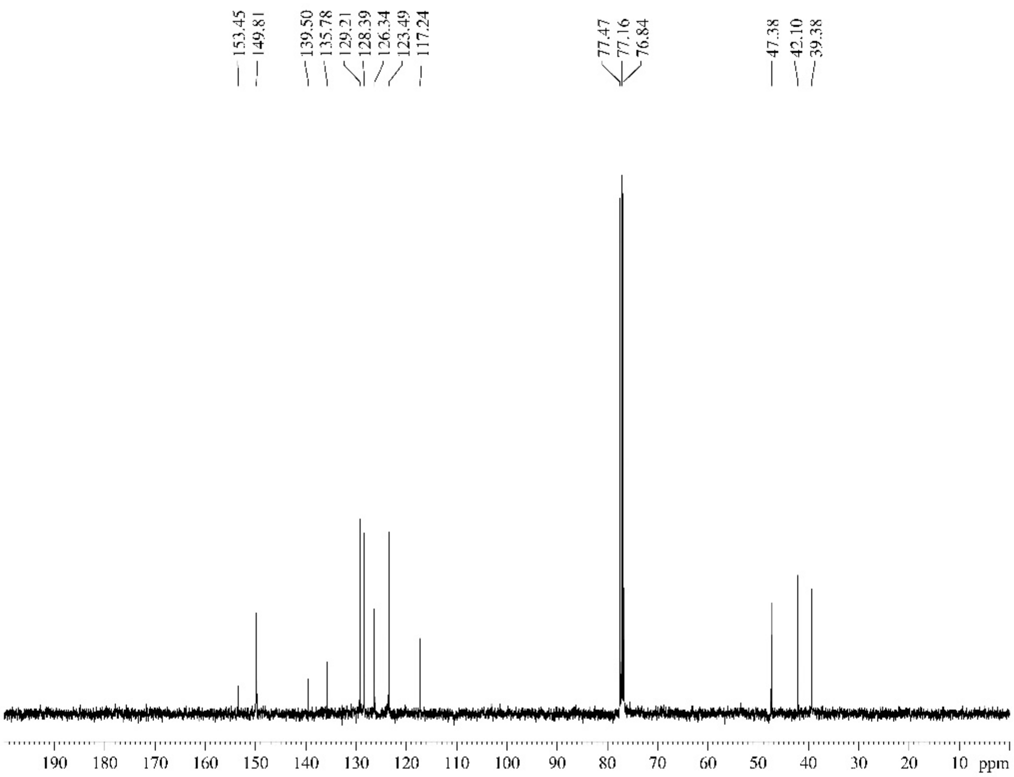

^{1}H (400 MHz, $CDCl_3$) and ^{13}C (101 MHz, $CDCl_3$) – NMR spectra of **2A**

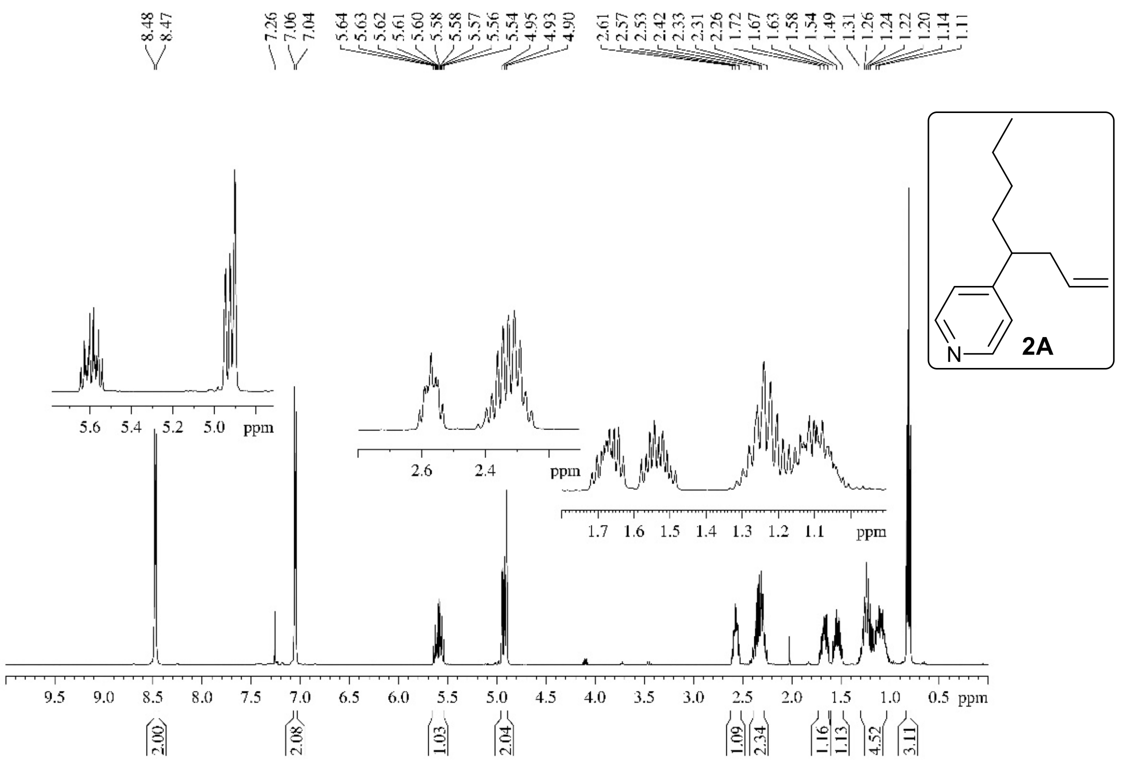

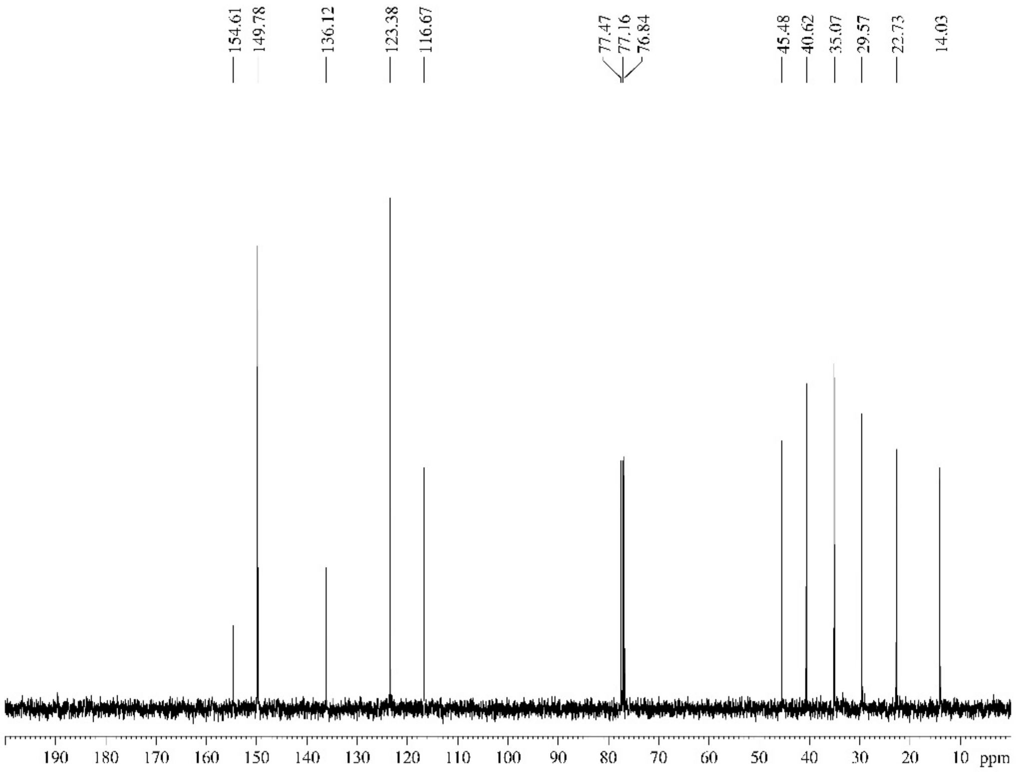

^{1}H (400 MHz, $CDCl_3$) and ^{13}C (101 MHz, $CDCl_3$) – NMR spectra of **3A**

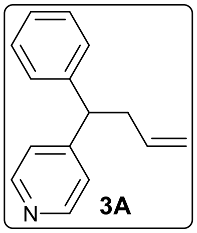

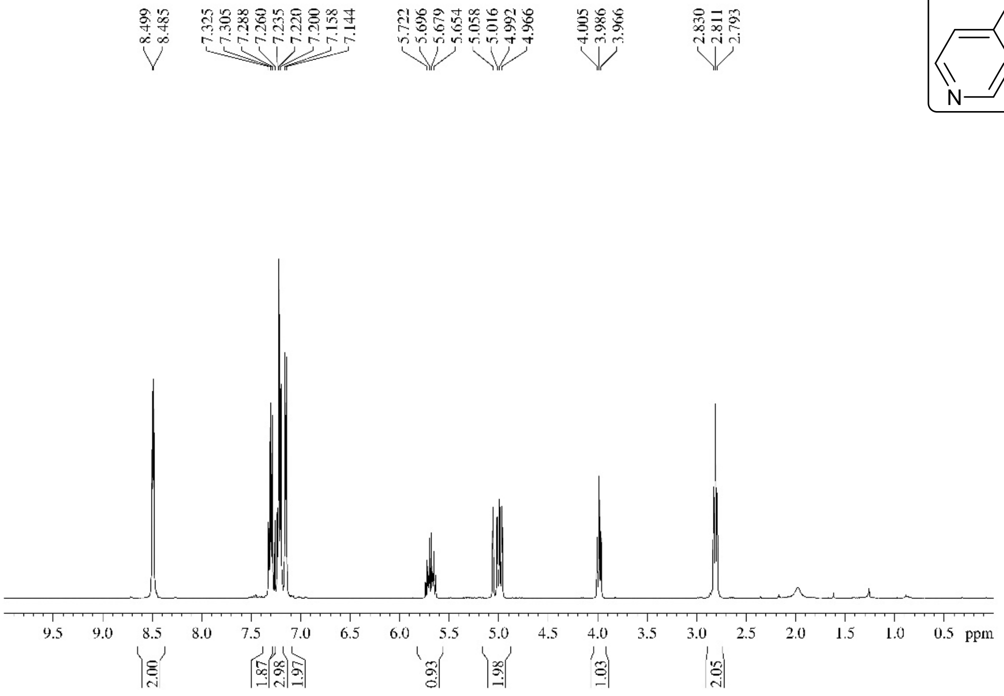

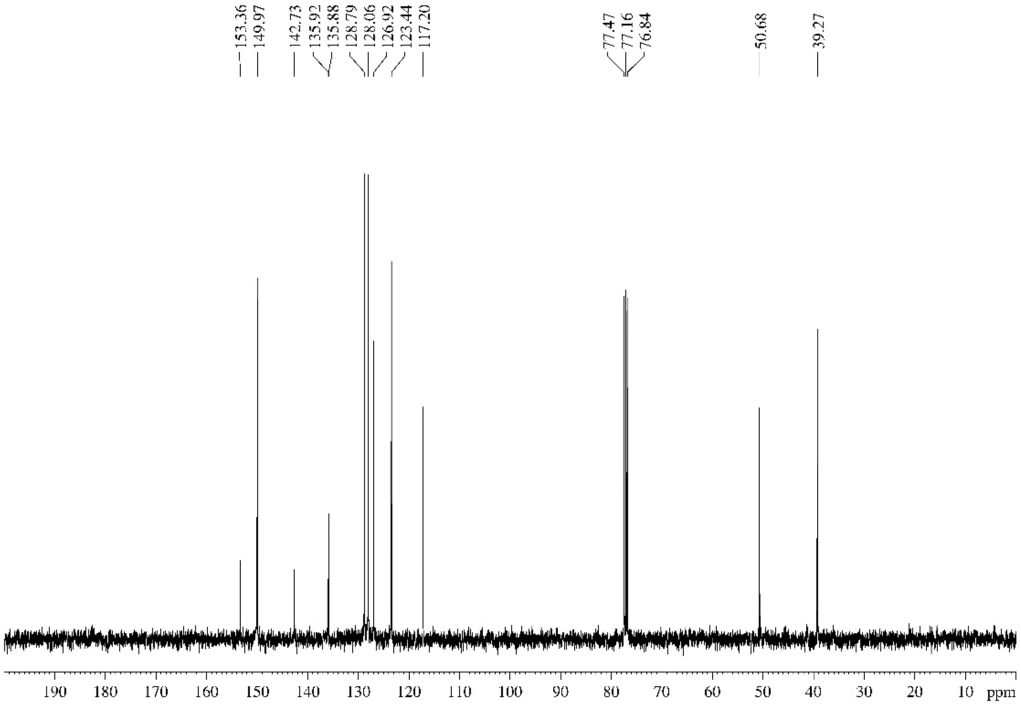

^{1}H (300 MHz, $CDCl_3$) and ^{13}C (76 MHz, $CDCl_3$) – NMR spectra of **4A**

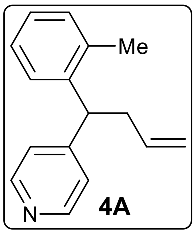

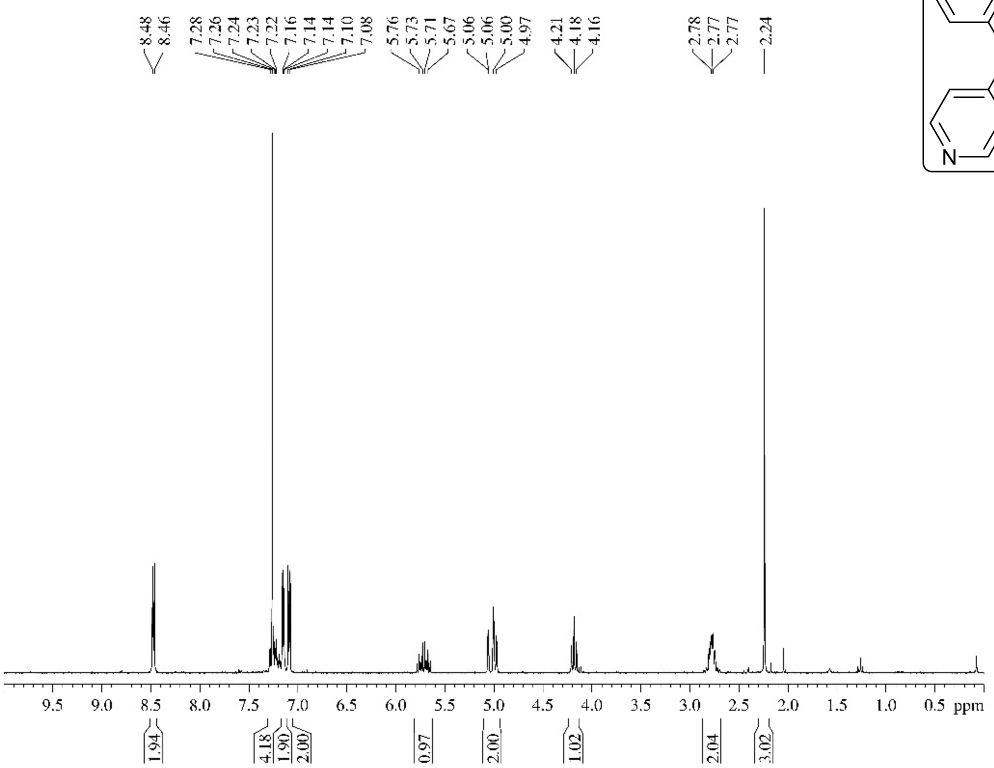

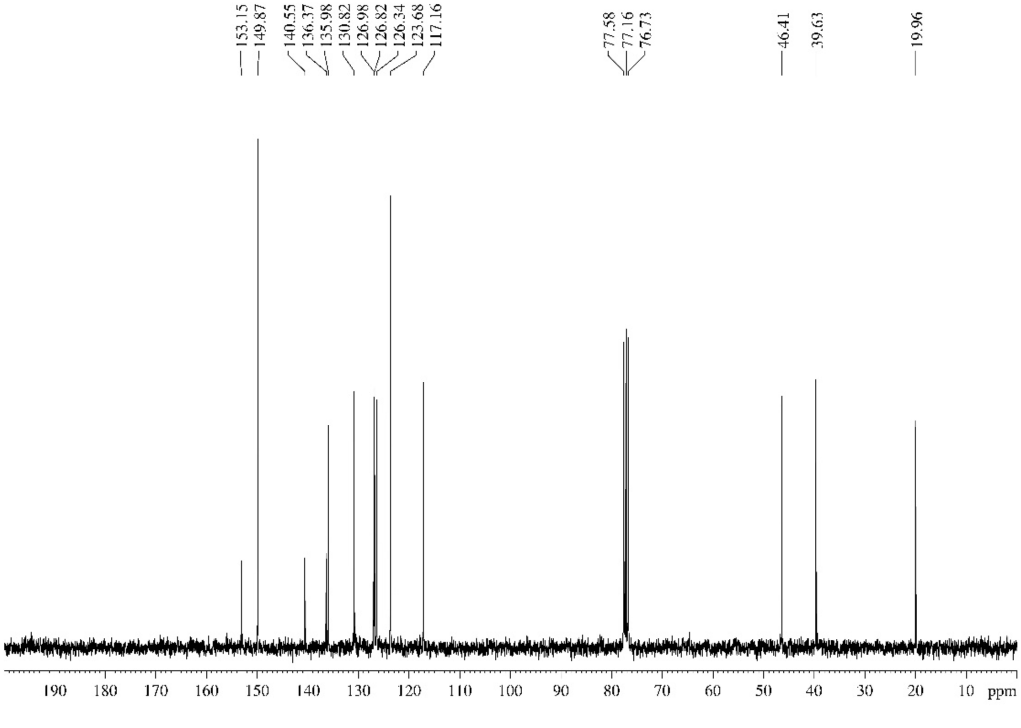

^{1}H (400 MHz, $CDCl_3$) and ^{13}C (101 MHz, $CDCl_3$) – NMR spectra of **5A**

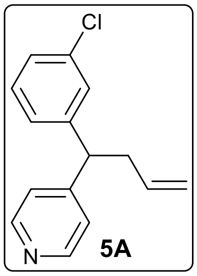

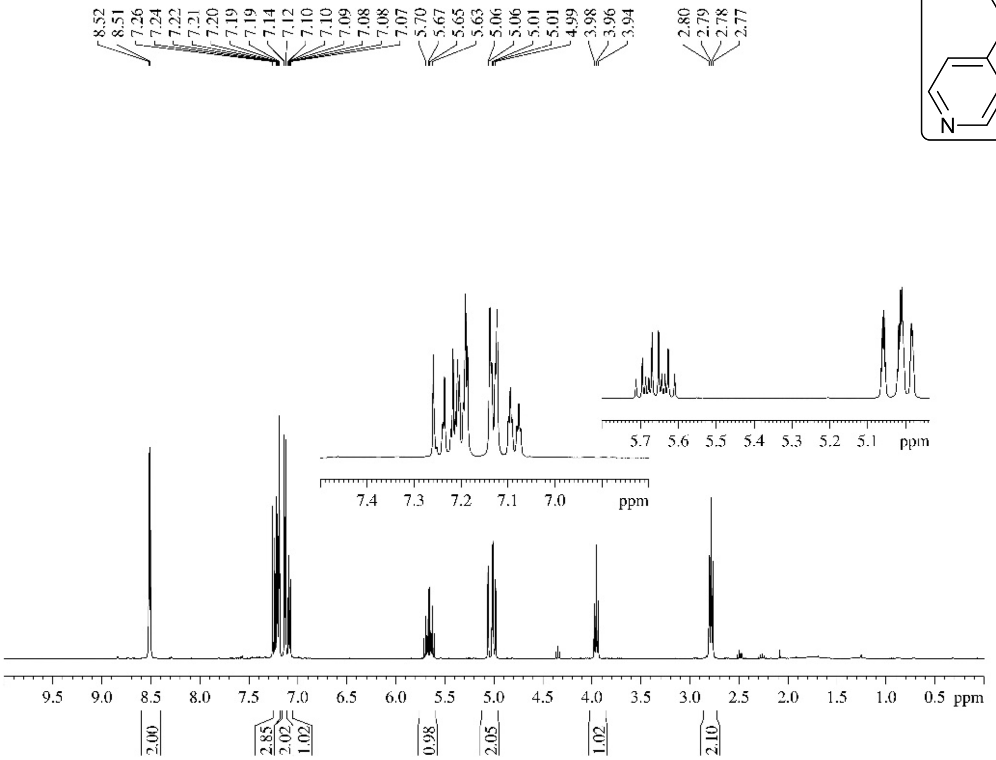

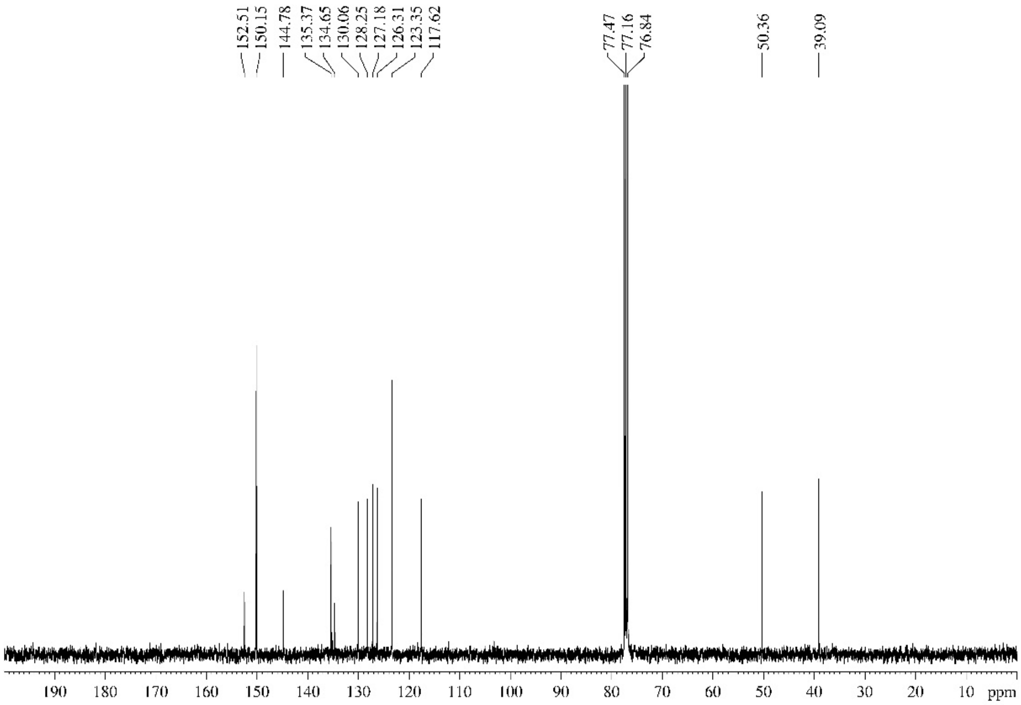

^{1}H (300 MHz, $CDCl_3$) and ^{13}C (76 MHz, $CDCl_3$) – NMR spectra of **6A**

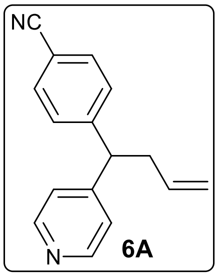

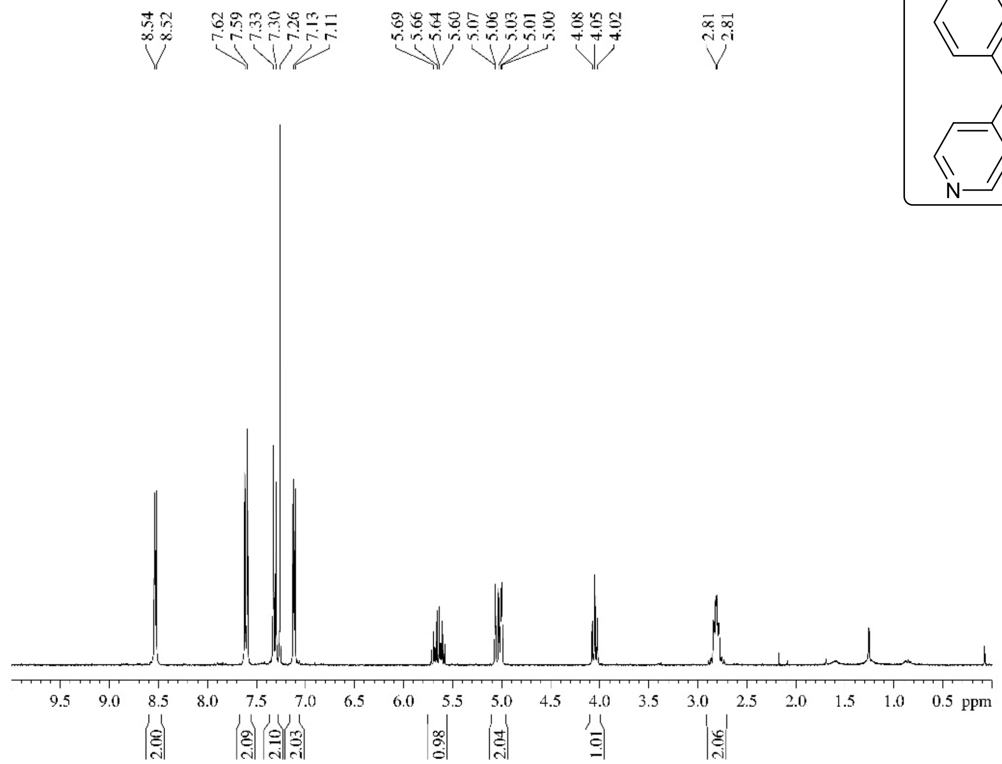

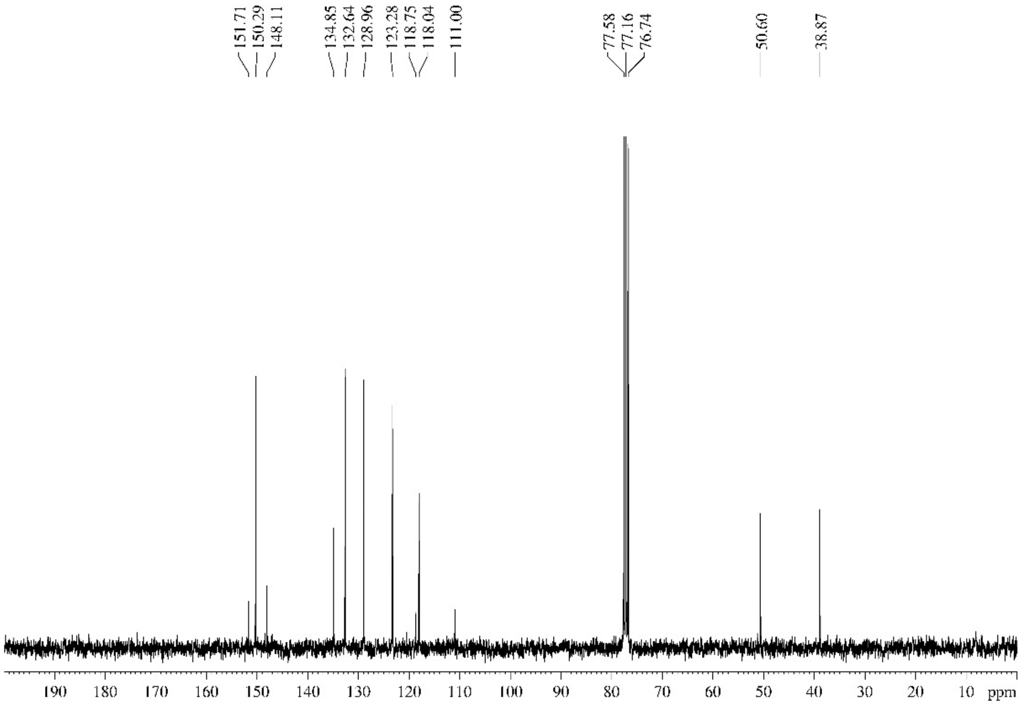

^{1}H (300 MHz, $CDCl_3$) and ^{13}C (76 MHz, $CDCl_3$) – NMR spectra of **7A**

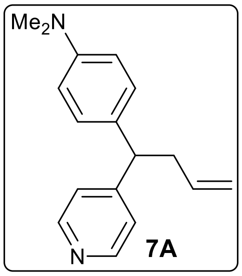

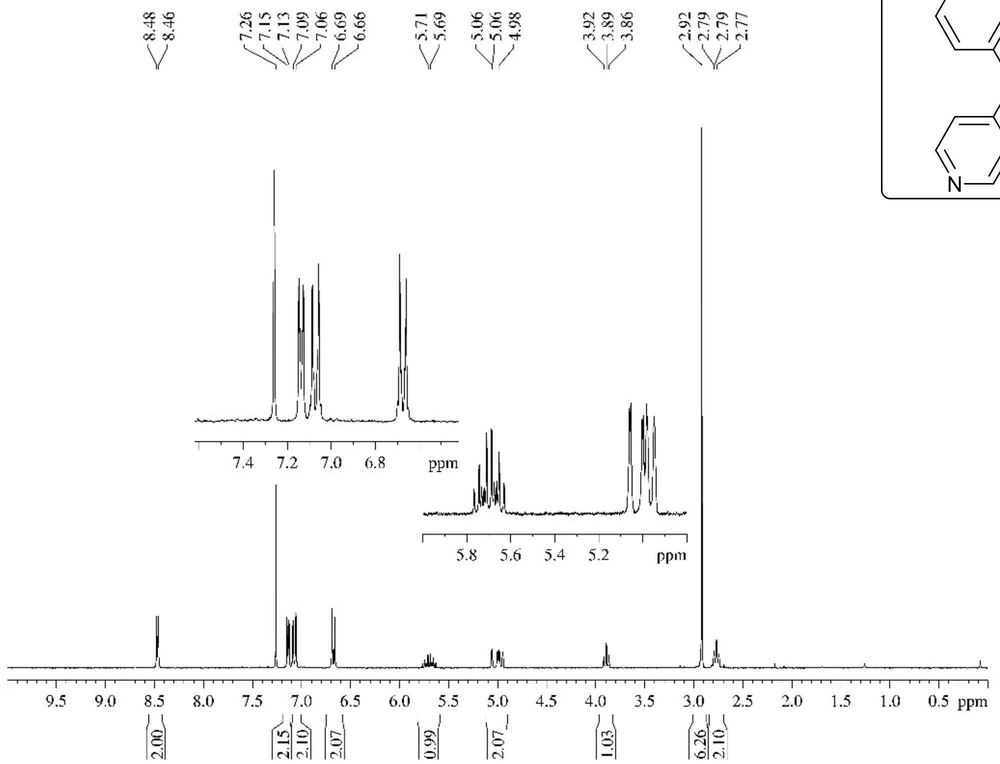

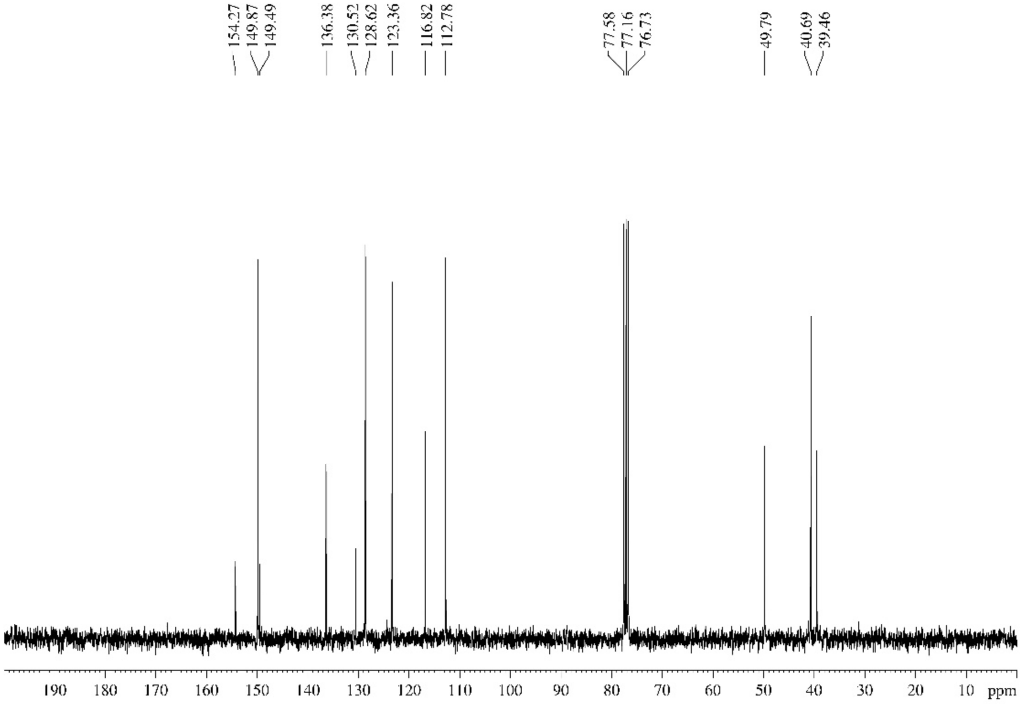

^{1}H (400 MHz, $CDCl_3$) and ^{13}C (101 MHz, $CDCl_3$) – NMR spectra of **8A**

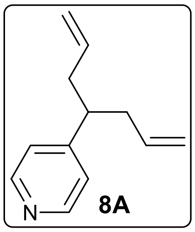

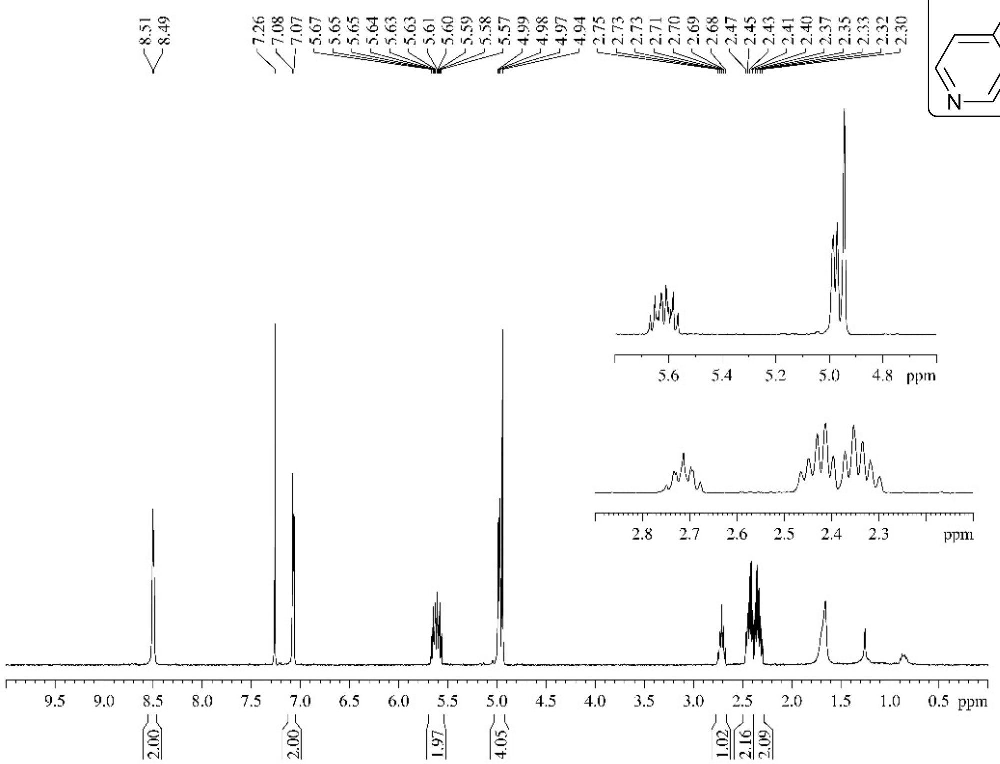

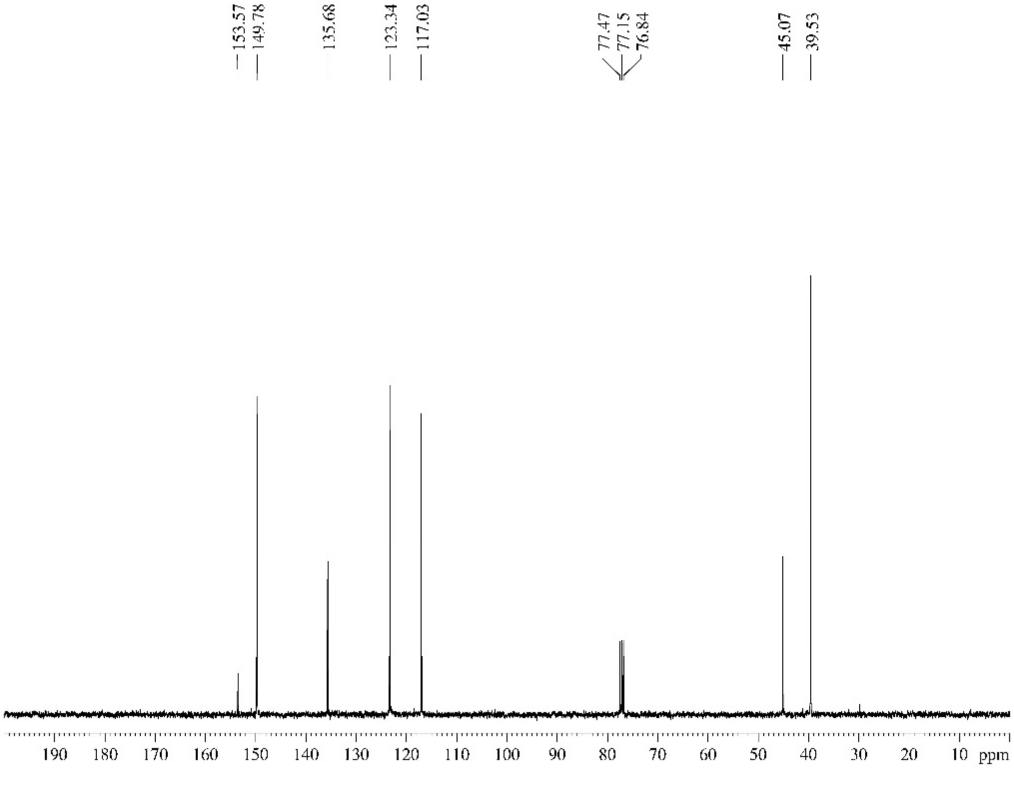

^{1}H (400 MHz, $CDCl_3$) and ^{13}C (101 MHz, $CDCl_3$) – NMR spectra of **9A**

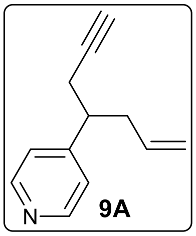

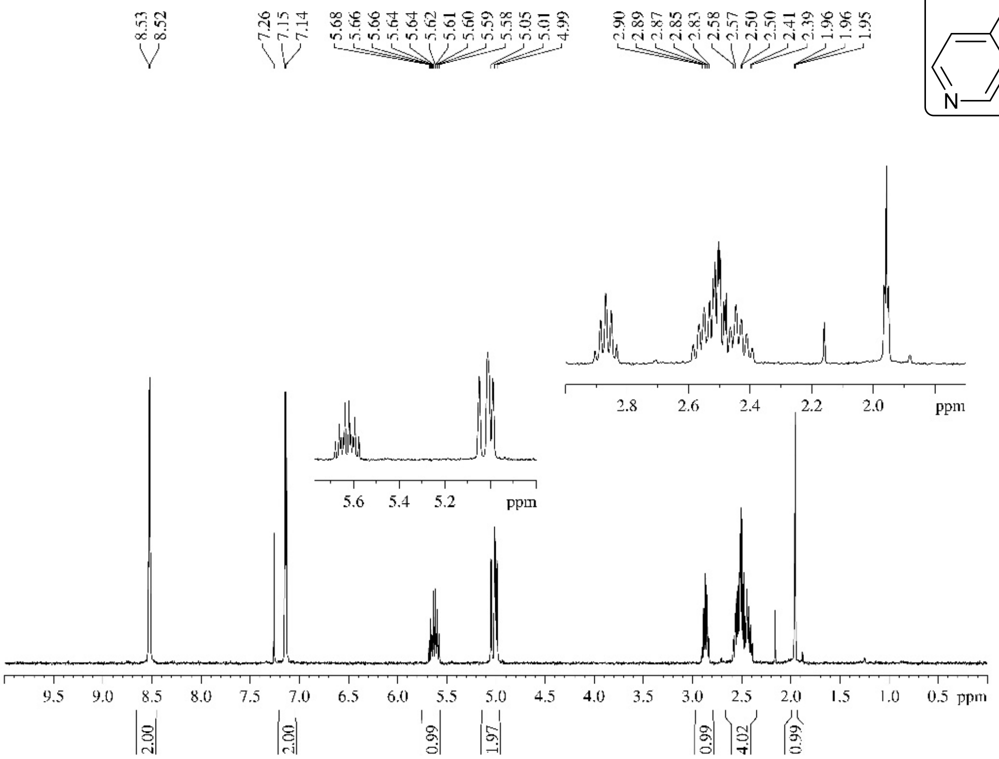

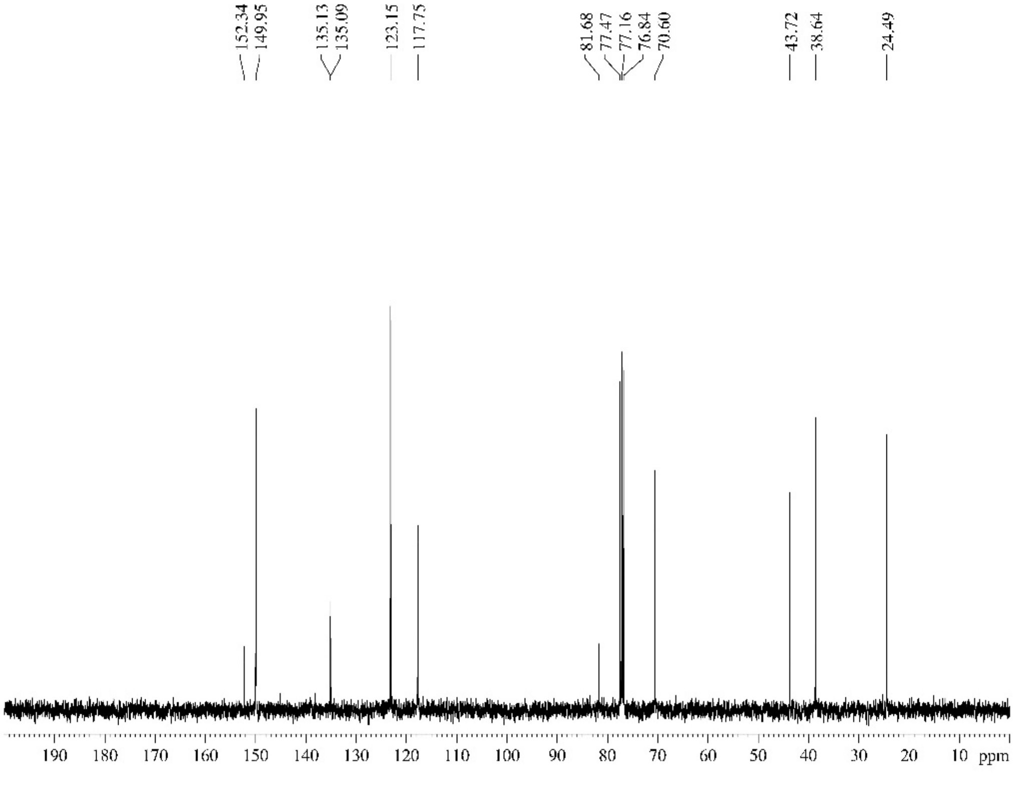

^{1}H (600 MHz, $CDCl_3$) and ^{13}C (101 MHz, $CDCl_3$) – NMR spectra of **10A**

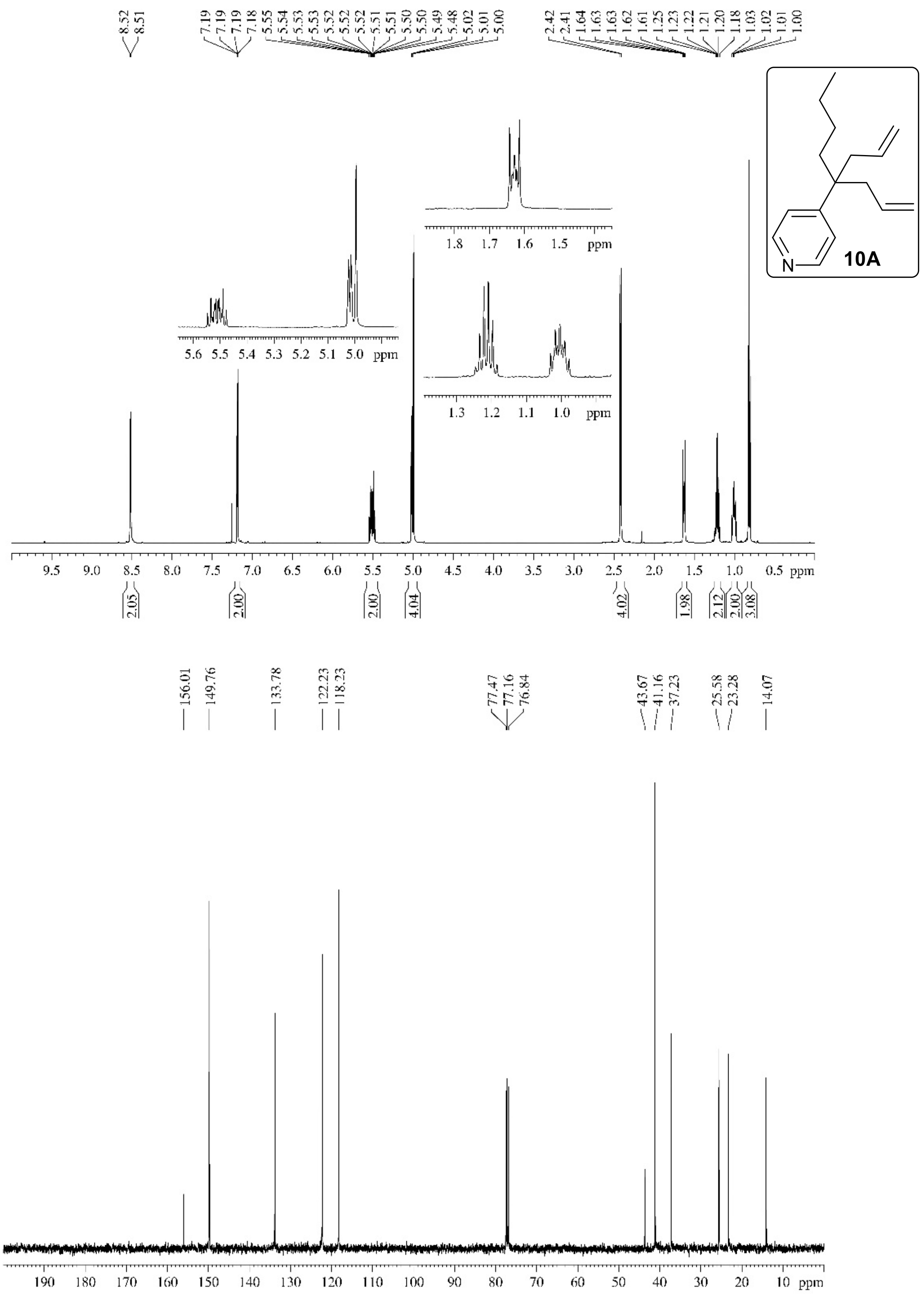

^{1}H (400 MHz, $CDCl_3$) – NMR spectra of **11’**

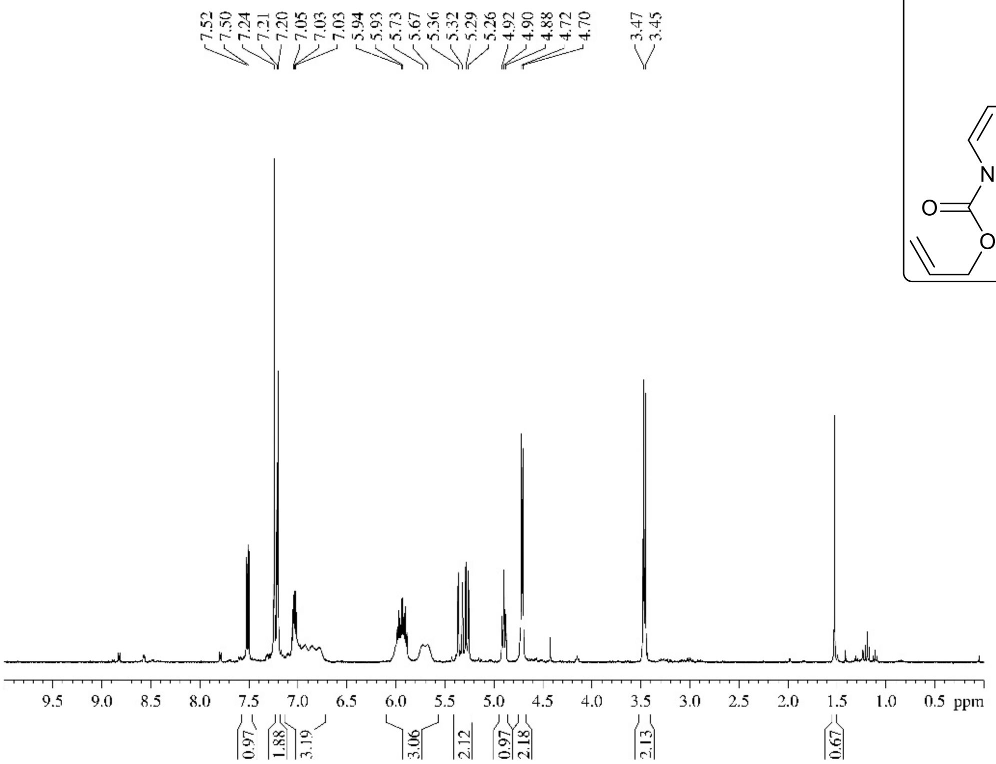

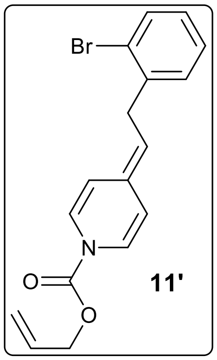

^{1}H (400 MHz, $CDCl_3$) and ^{13}C (101 MHz, $CDCl_3$) – NMR spectra of **11A**

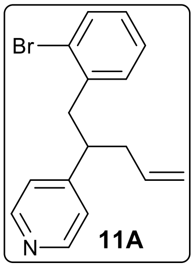

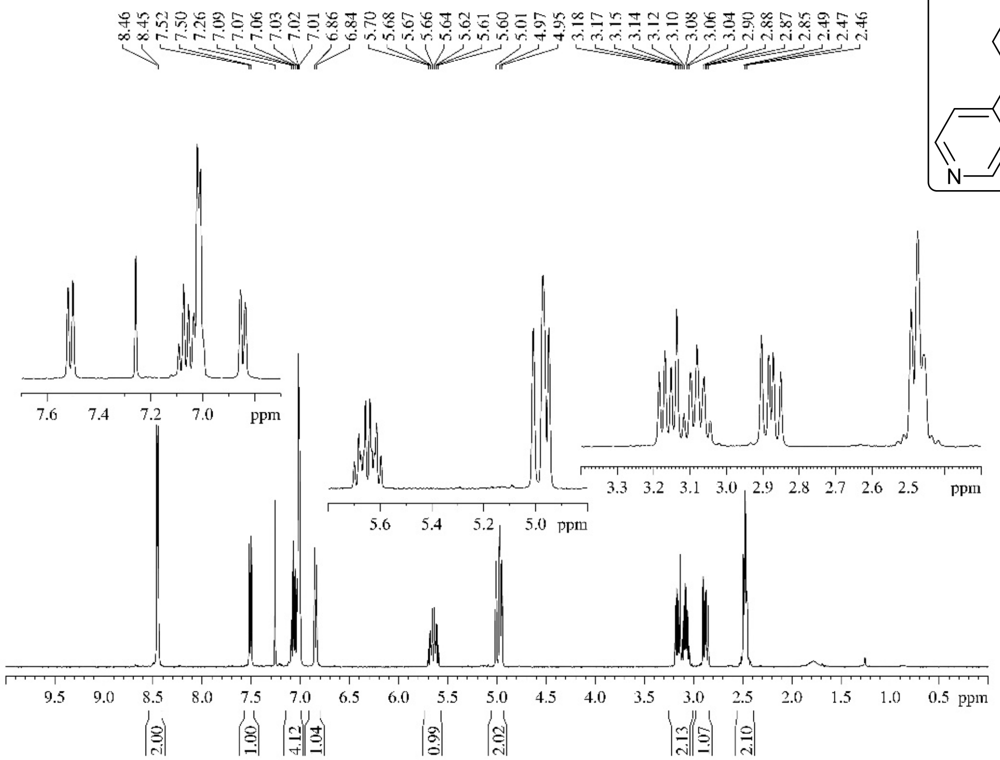

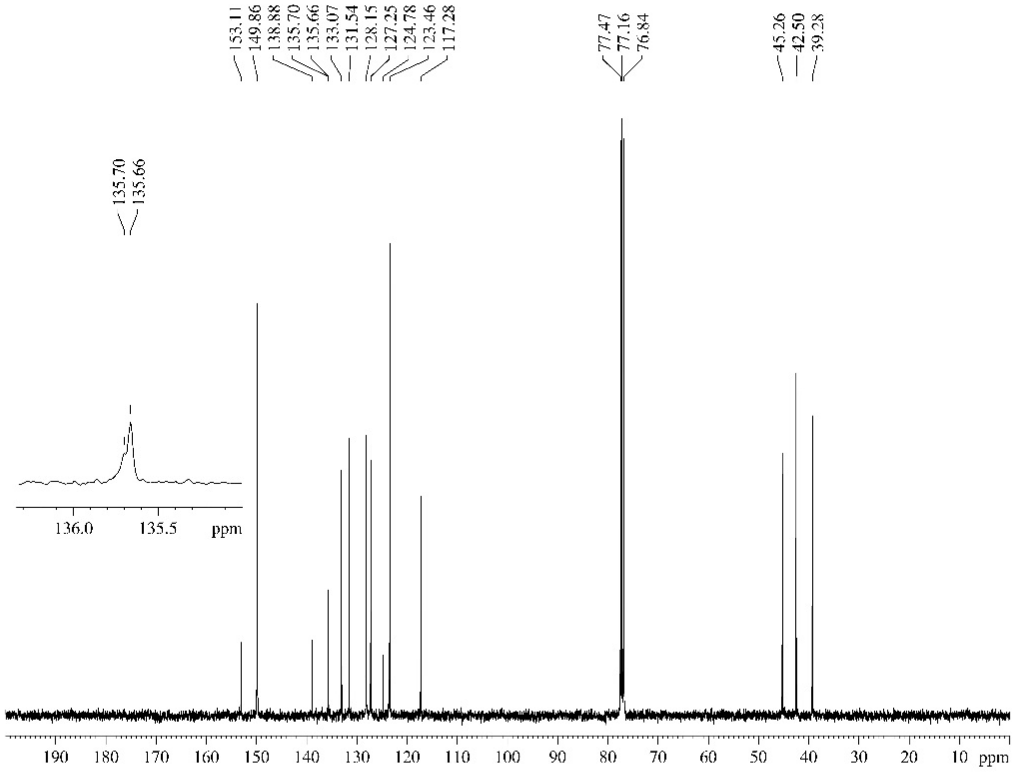

^{1}H (300 MHz, $CDCl_3$) and ^{13}C (76 MHz, $CDCl_3$) – NMR spectra of **12A**

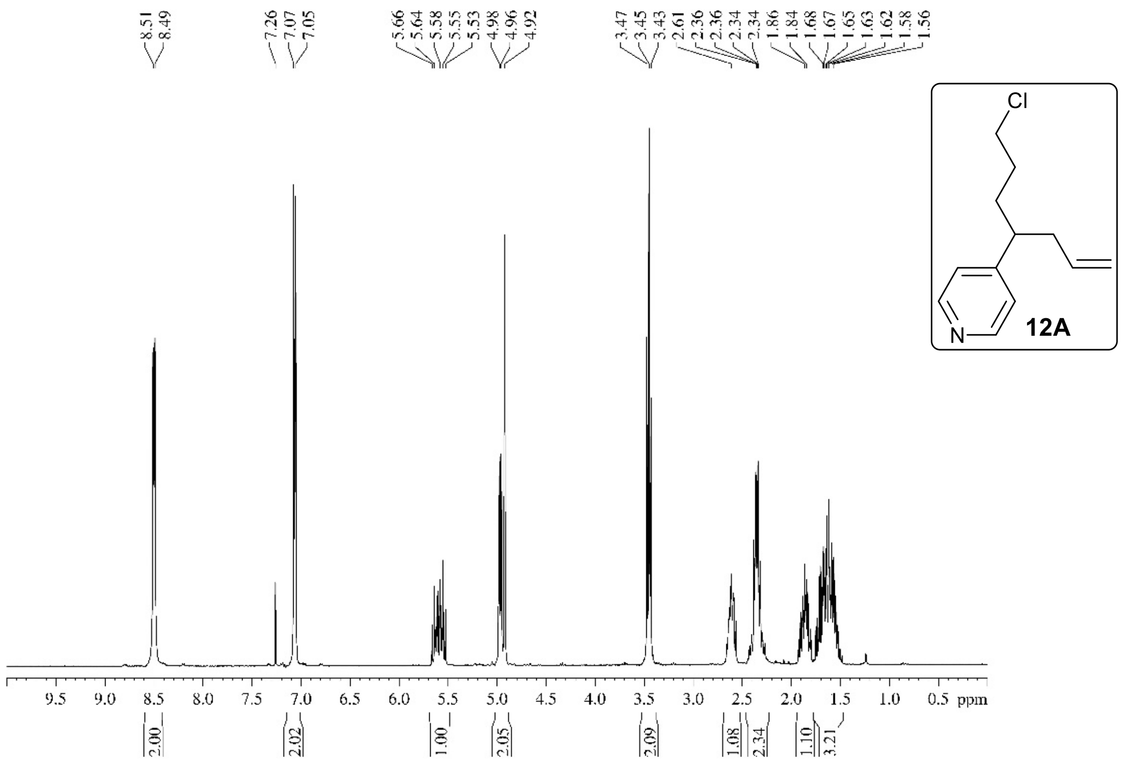

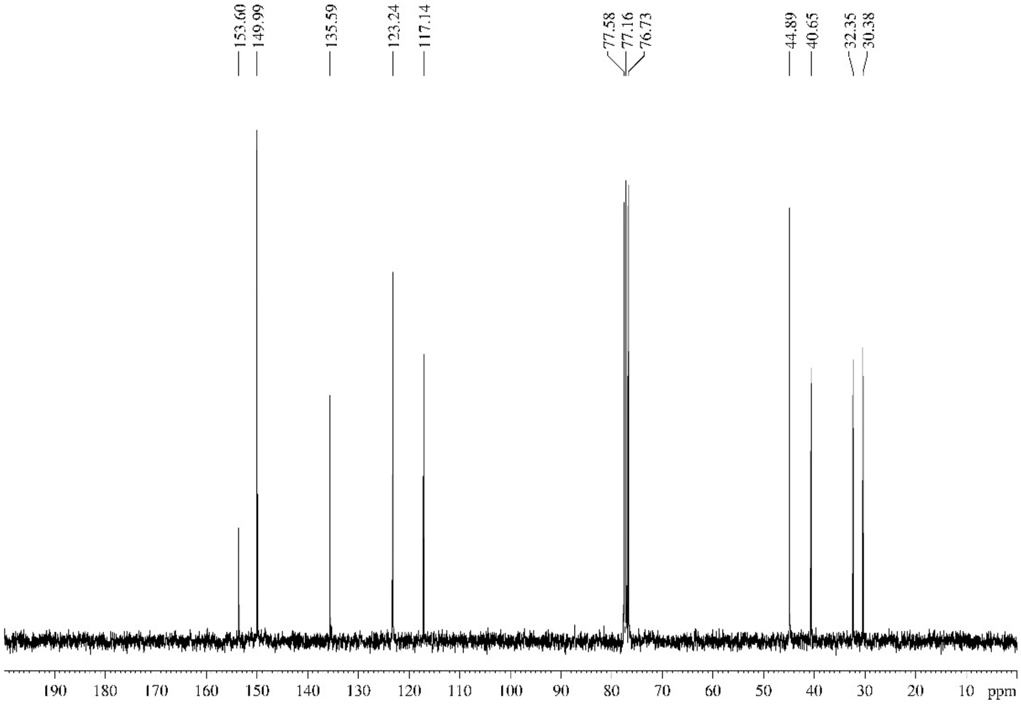

^{1}H (400 MHz, $CDCl_3$) and ^{13}C (101 MHz, $CDCl_3$) – NMR spectra of **13A**

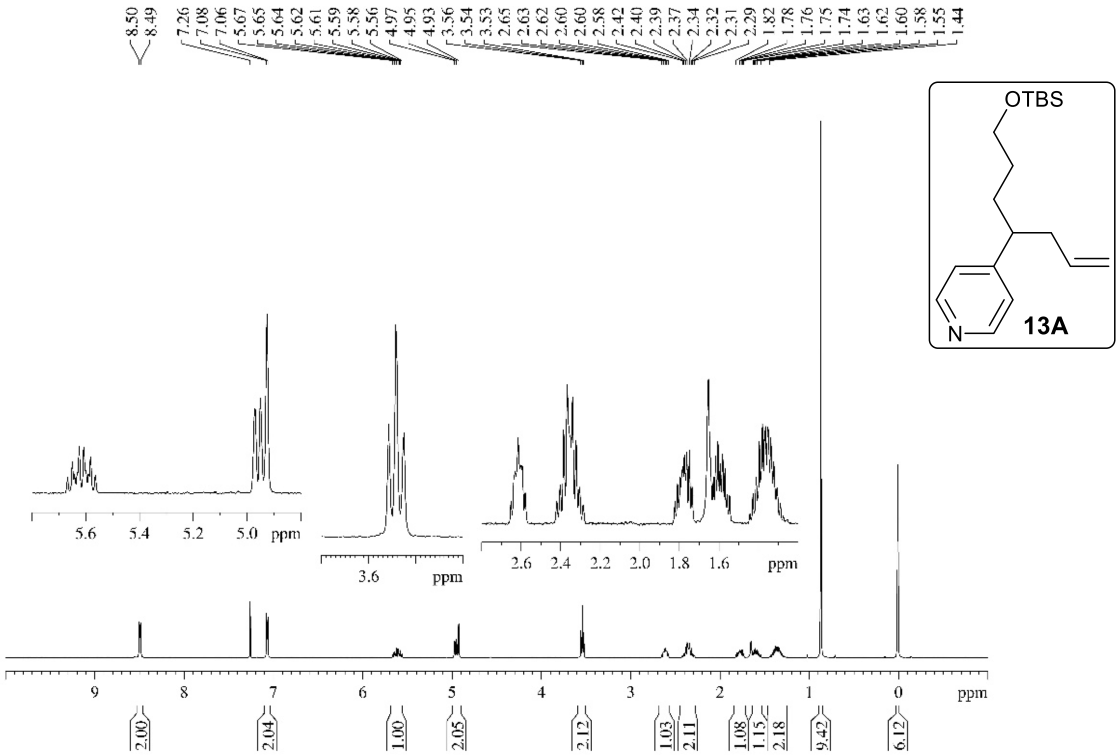

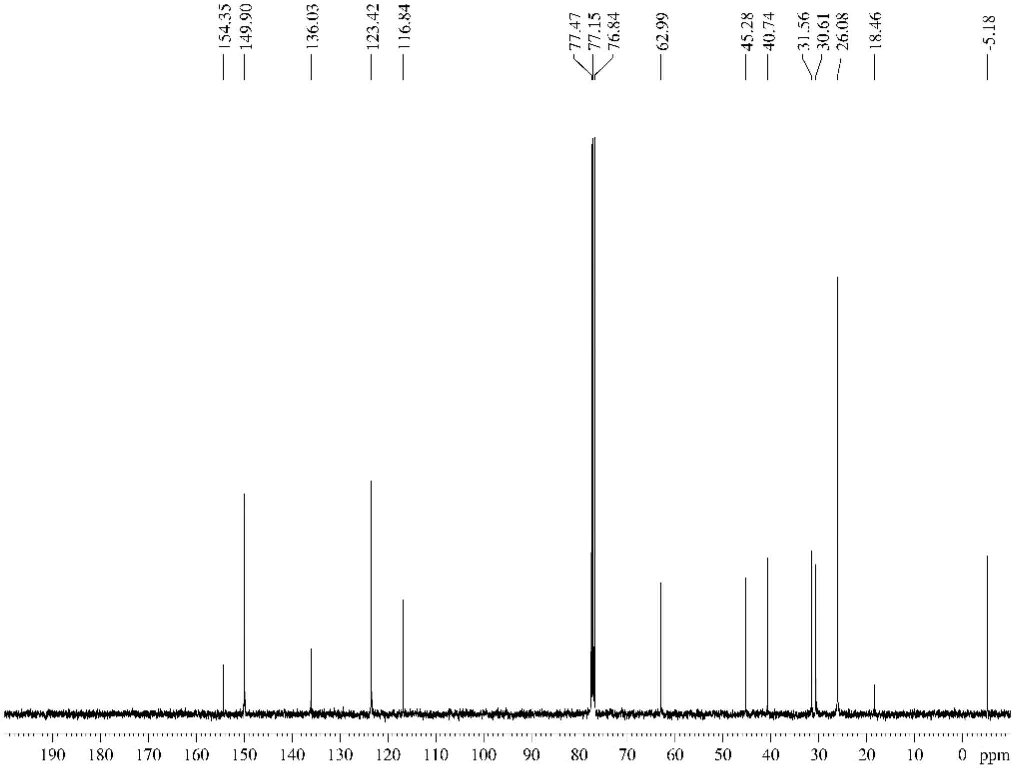

^{29}Si (57 MHz, $CDCl_3$) – NMR spectrum of **13A**

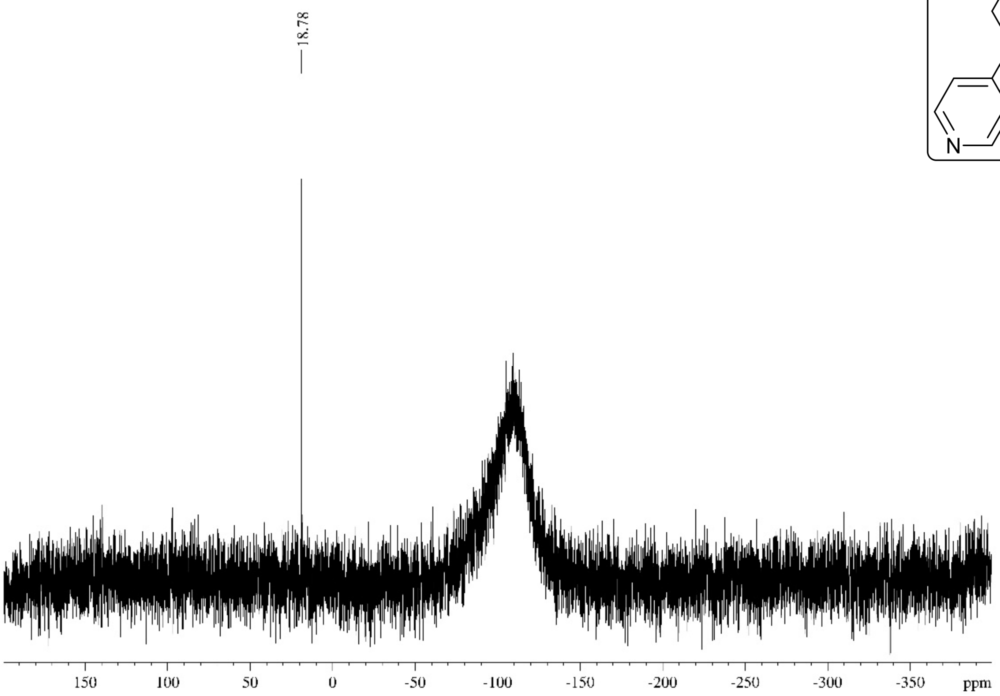

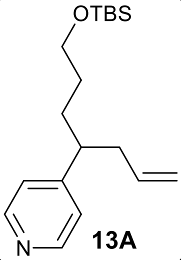

13A

^{1}H (400 MHz, $CDCl_3$) and ^{13}C (101 MHz, $CDCl_3$) – NMR spectra of **14A**

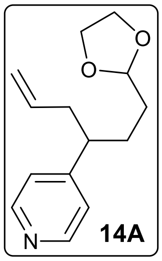

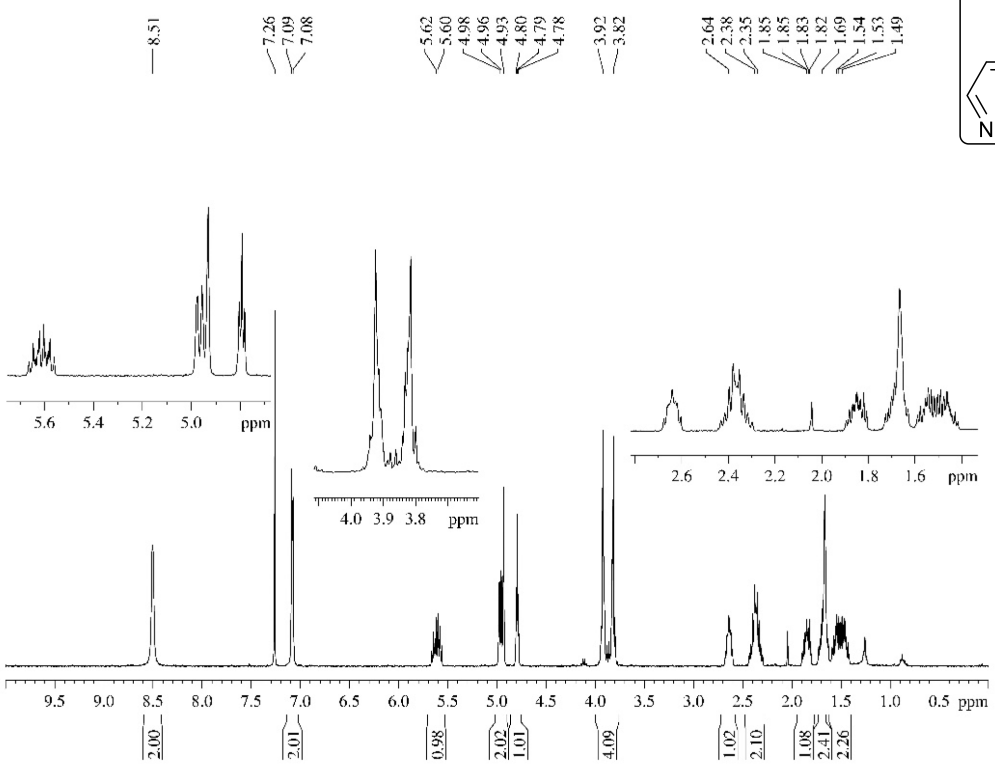

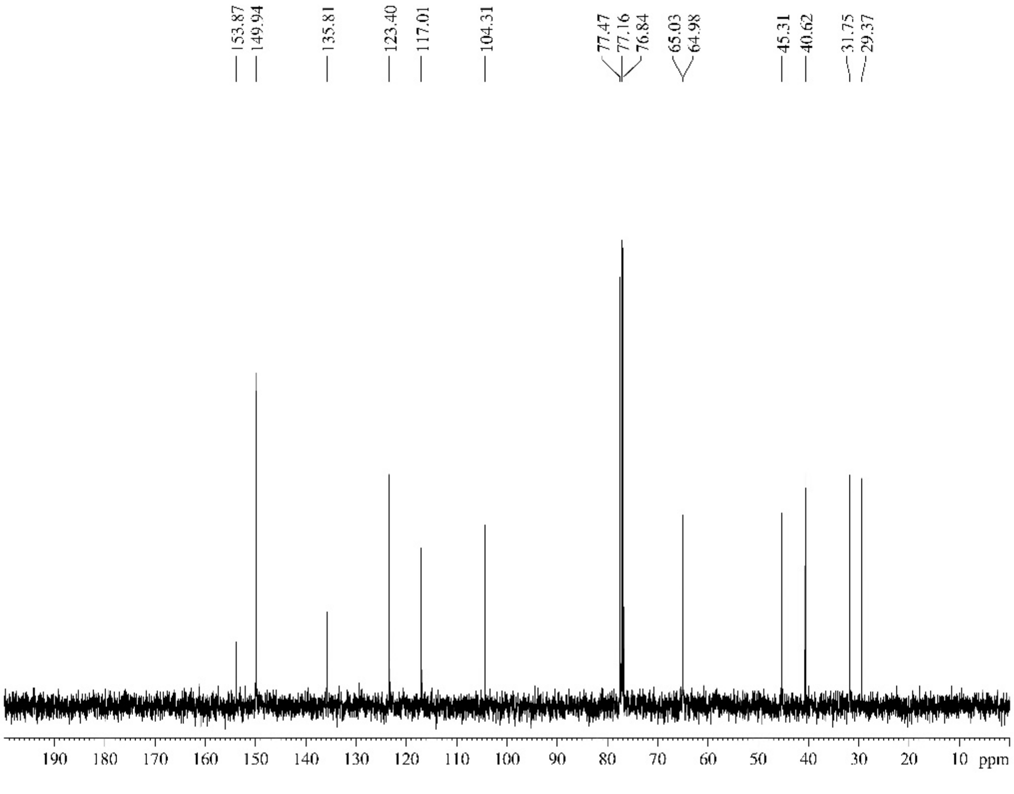

^{1}H (400 MHz, $CDCl_3$) and ^{13}C (101 MHz, $CDCl_3$) – NMR spectra of **15A**

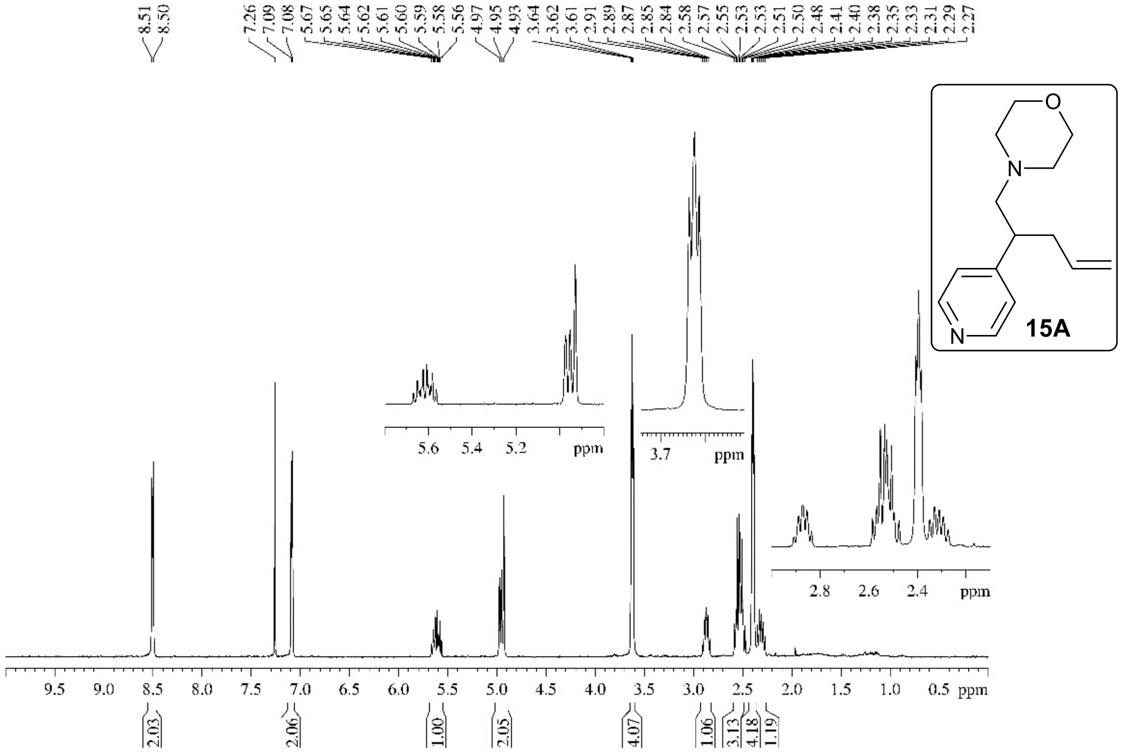

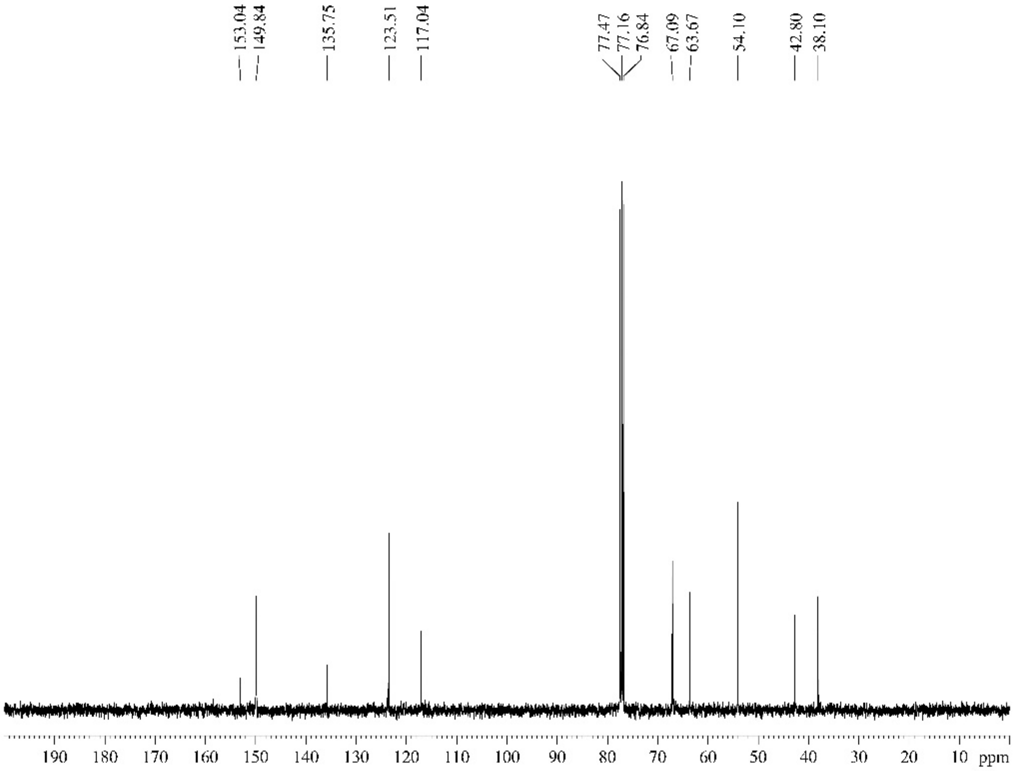

^{1}H (400 MHz, $CDCl_3$) and ^{13}C (101 MHz, $CDCl_3$) – NMR spectra of **16A**

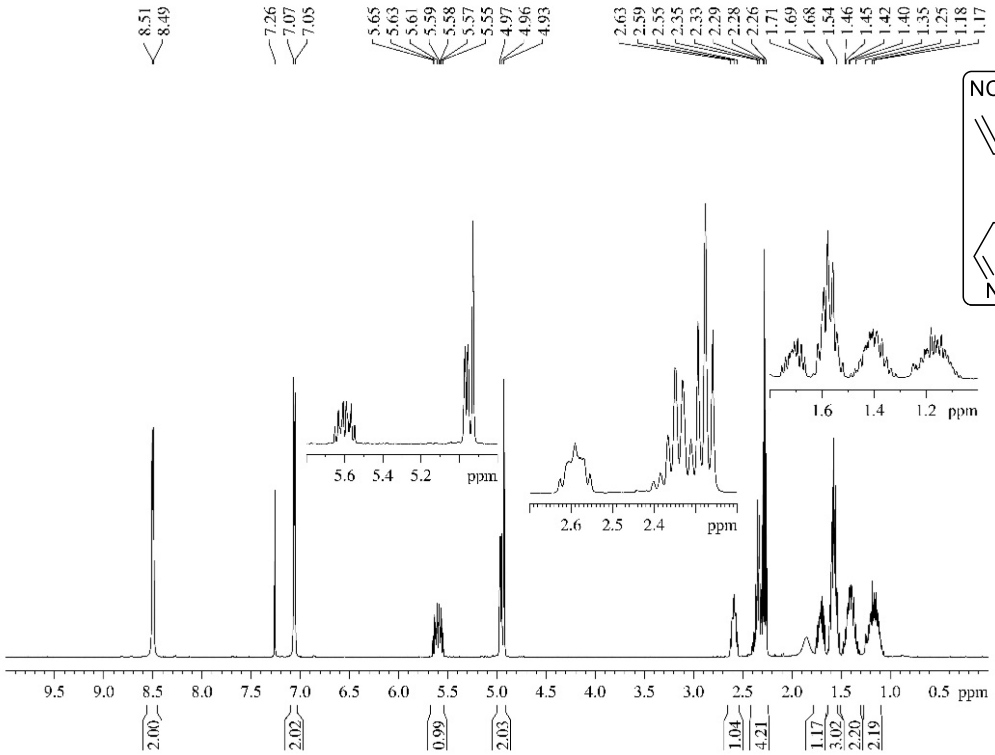

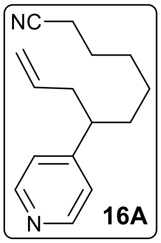

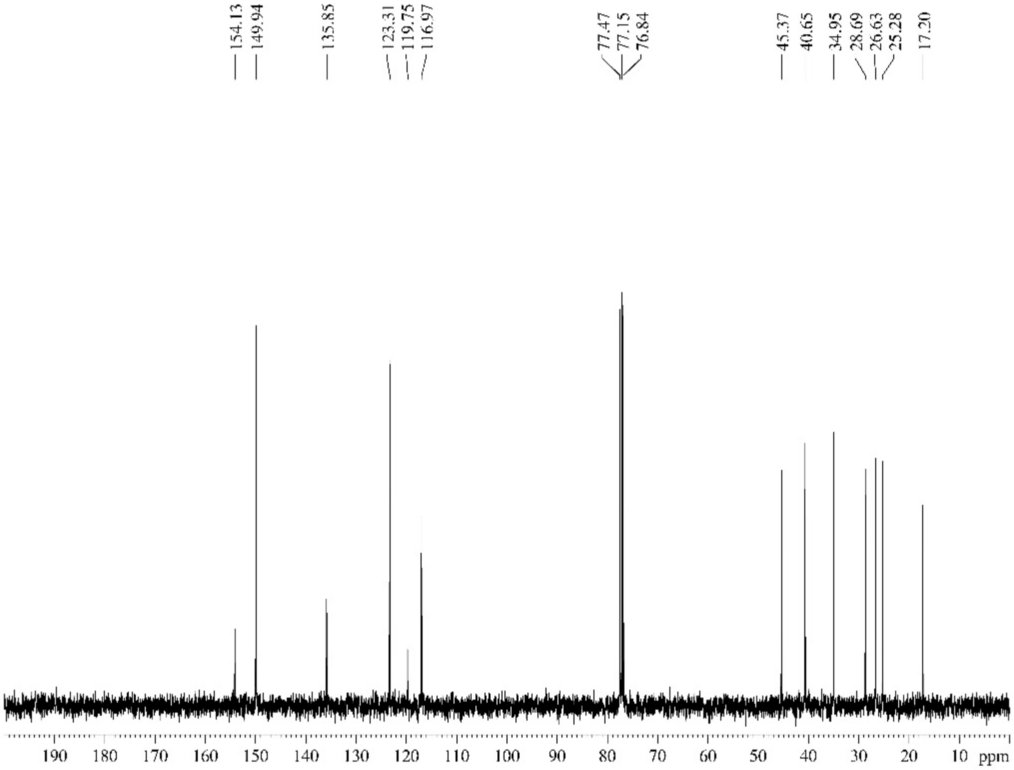

^1H (400 MHz, $CDCl_3$) and ^{13}C (101 MHz, $CDCl_3$) – NMR spectra of **17A**

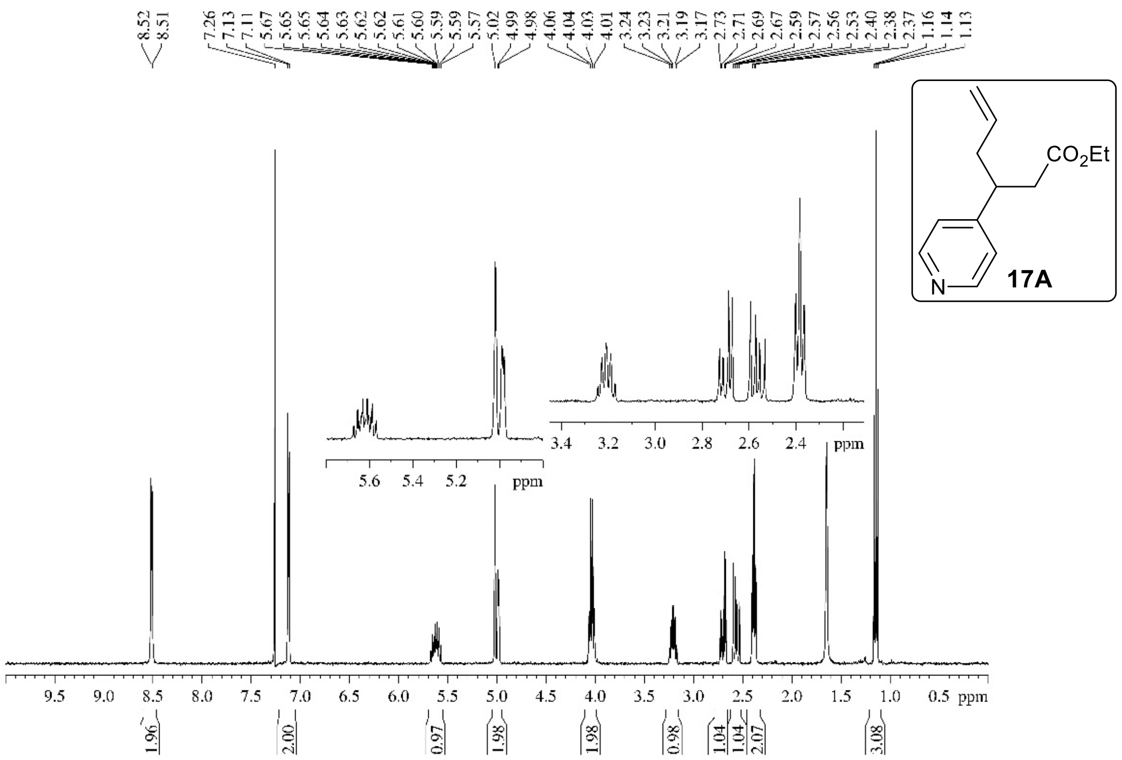

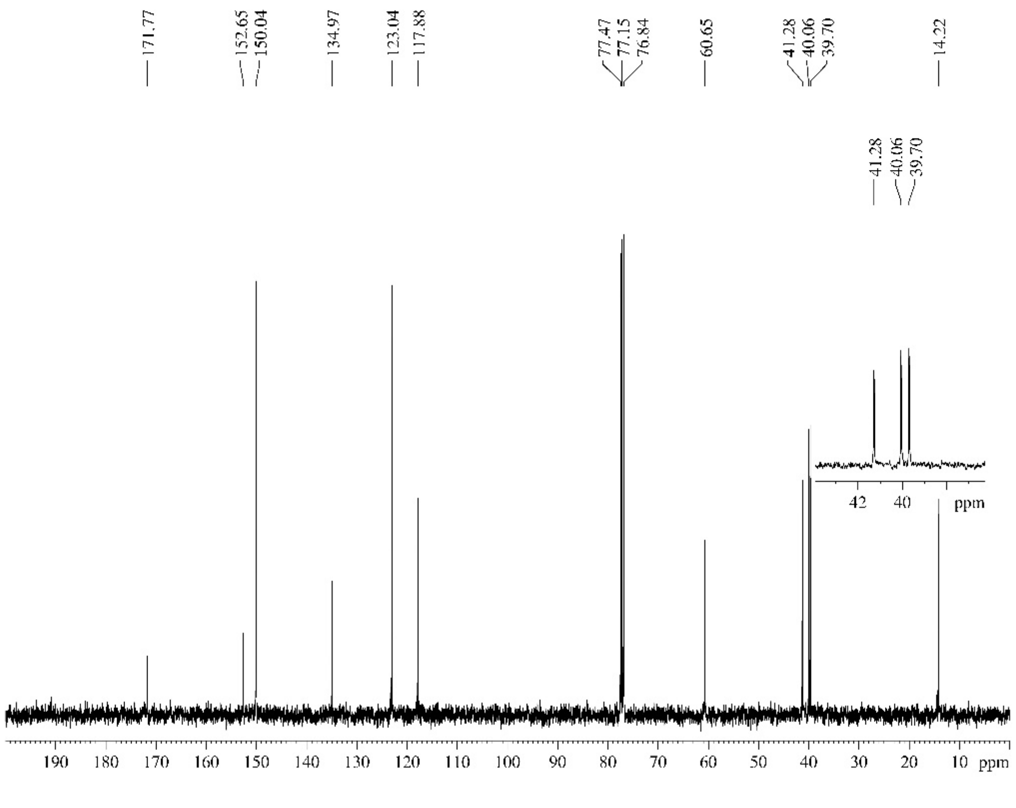

^{1}H (400 MHz, CDCl$_3$) and ^{13}C (101 MHz, CDCl$_3$) – NMR spectra of **18A**

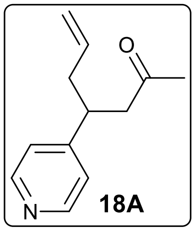

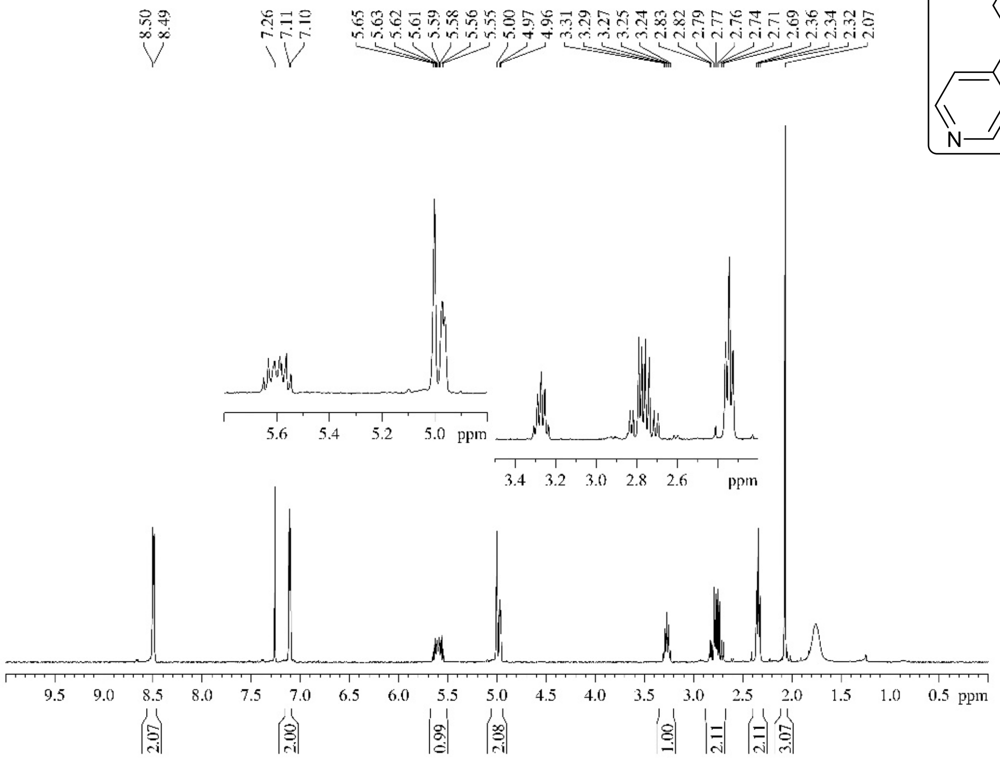

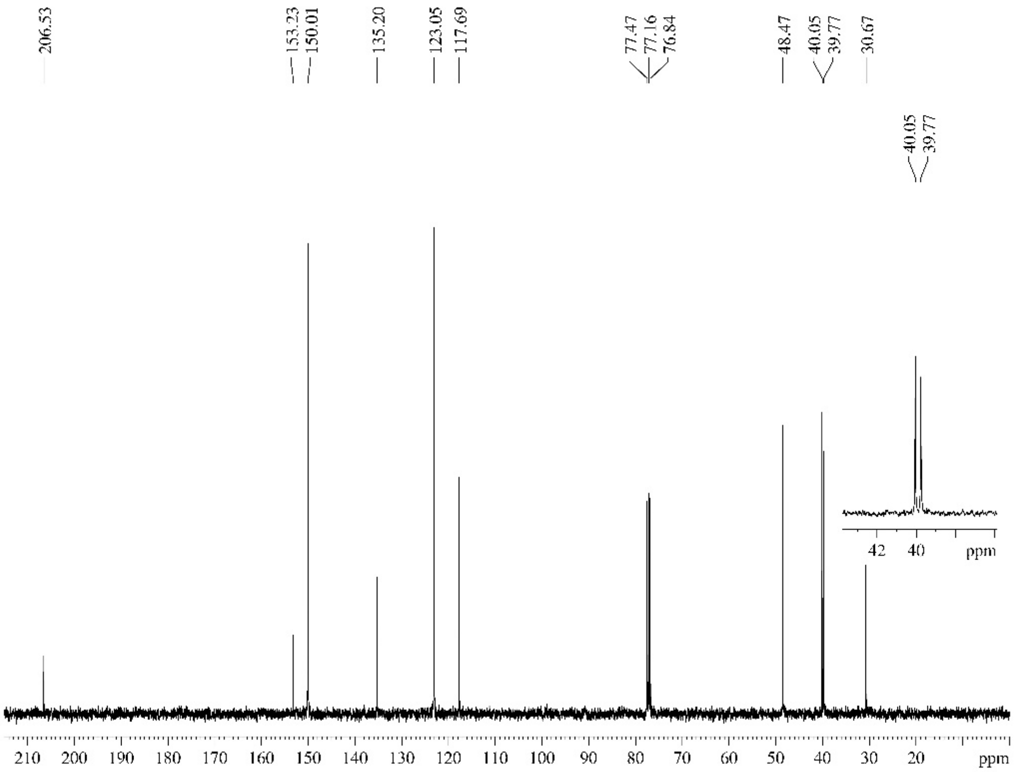

^{1}H (400 MHz, $CDCl_3$) and ^{13}C (101 MHz, $CDCl_3$) – NMR spectra of **19A**

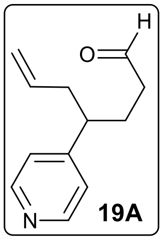

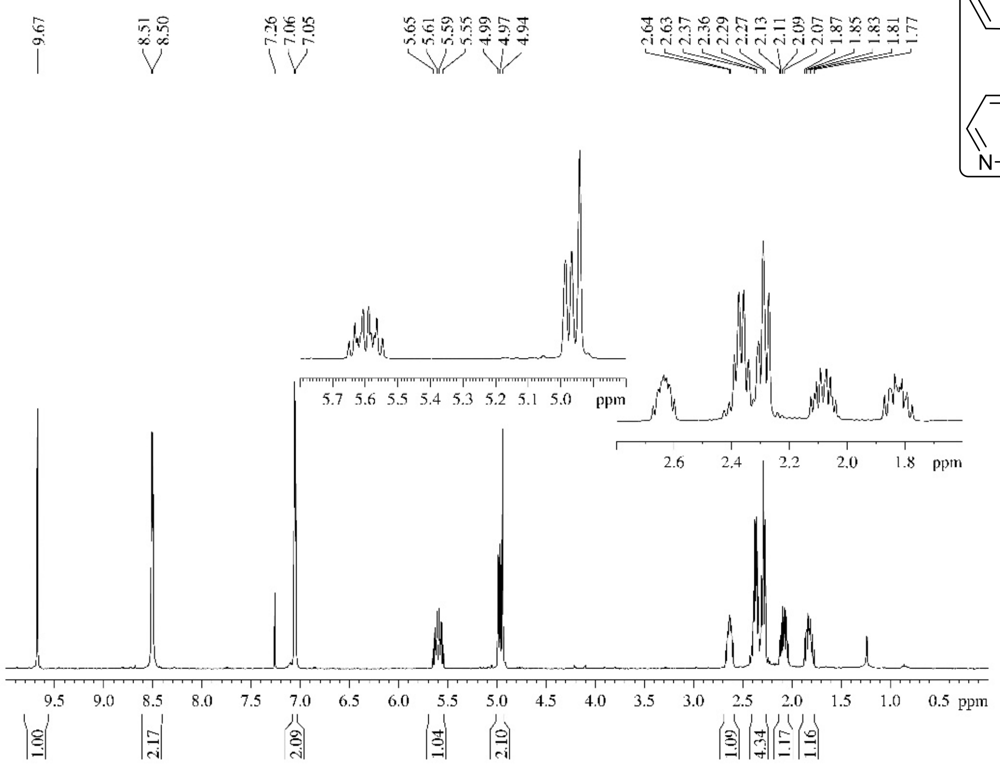

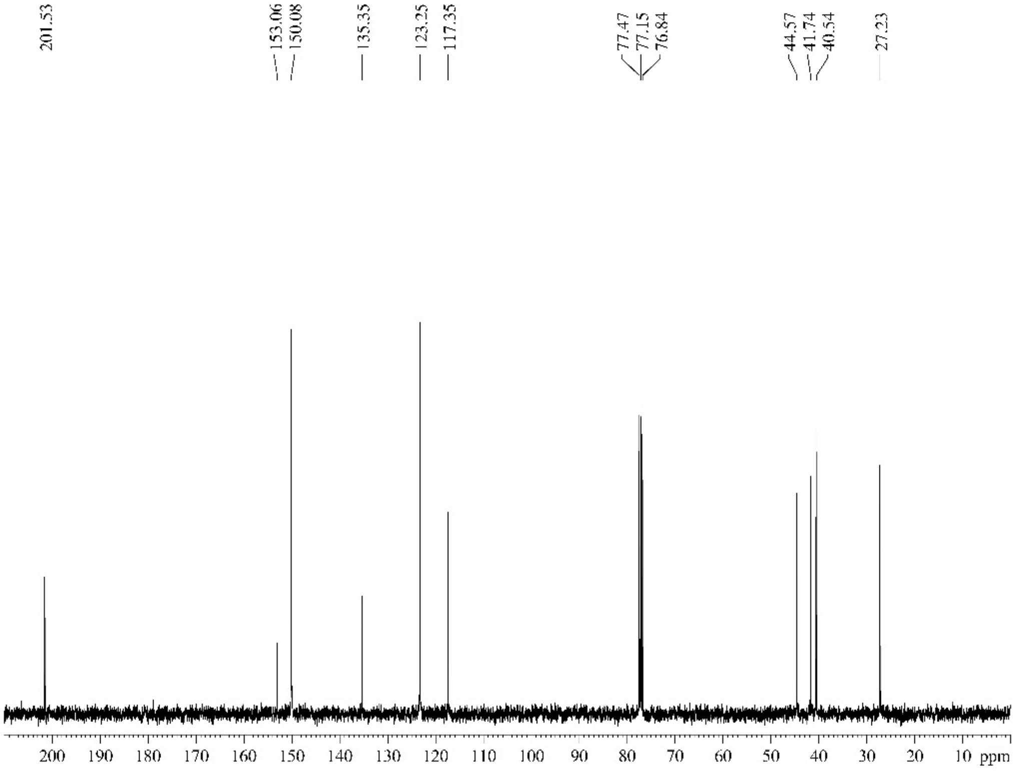

^{1}H (400 MHz, $CDCl_3$) and ^{13}C (101 MHz, $CDCl_3$) – NMR spectra of **20A**

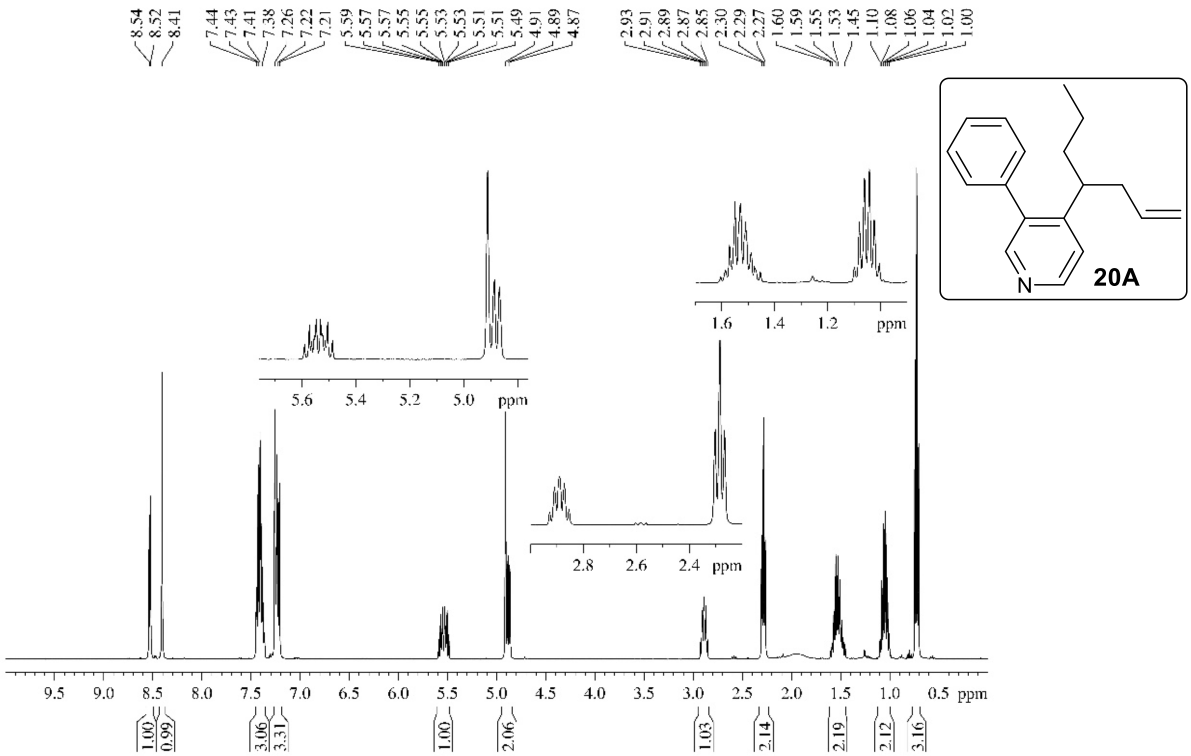

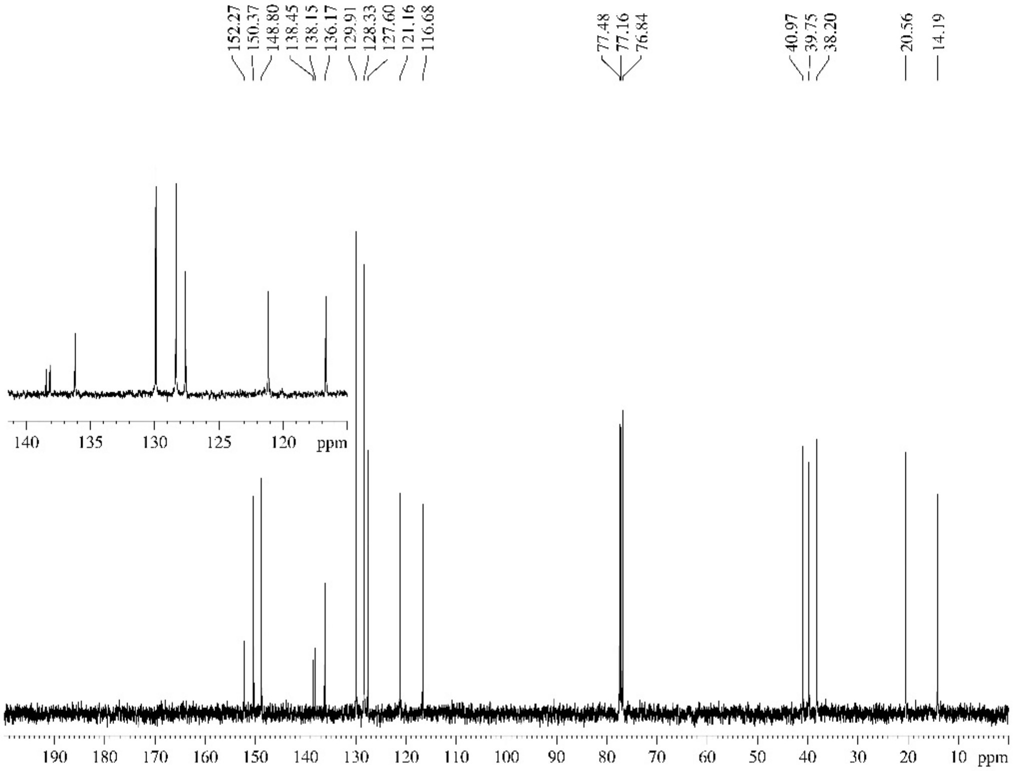

^{1}H (300 MHz, $CDCl_3$) and ^{13}C (76 MHz, $CDCl_3$) – NMR spectra of **21A**

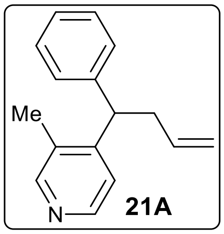

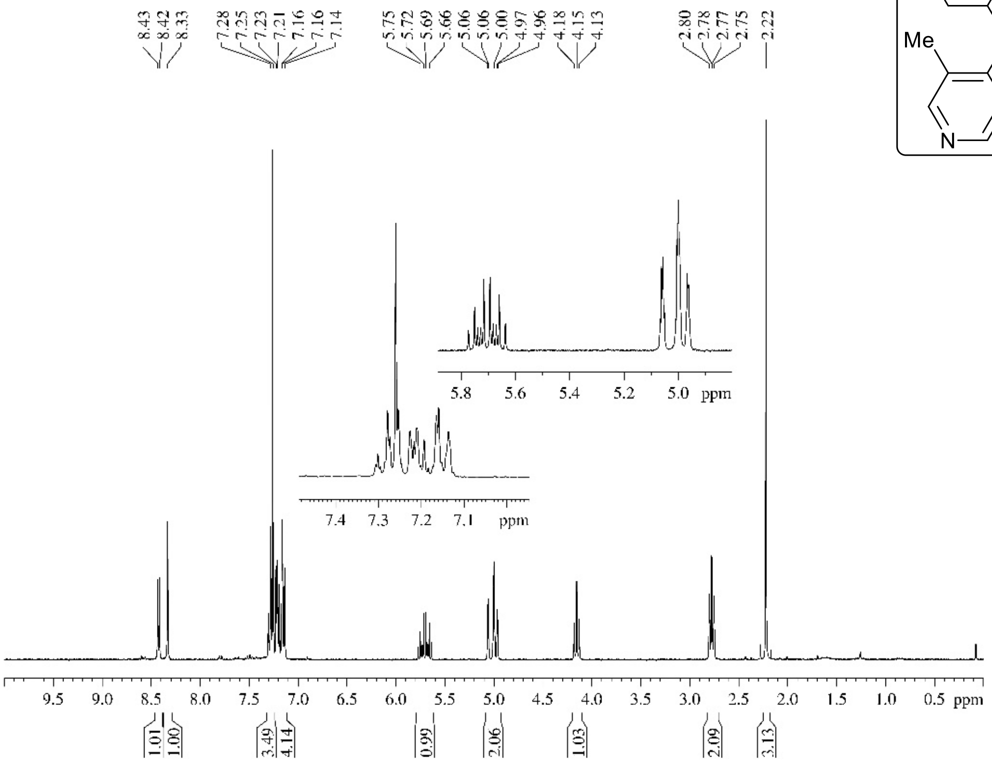

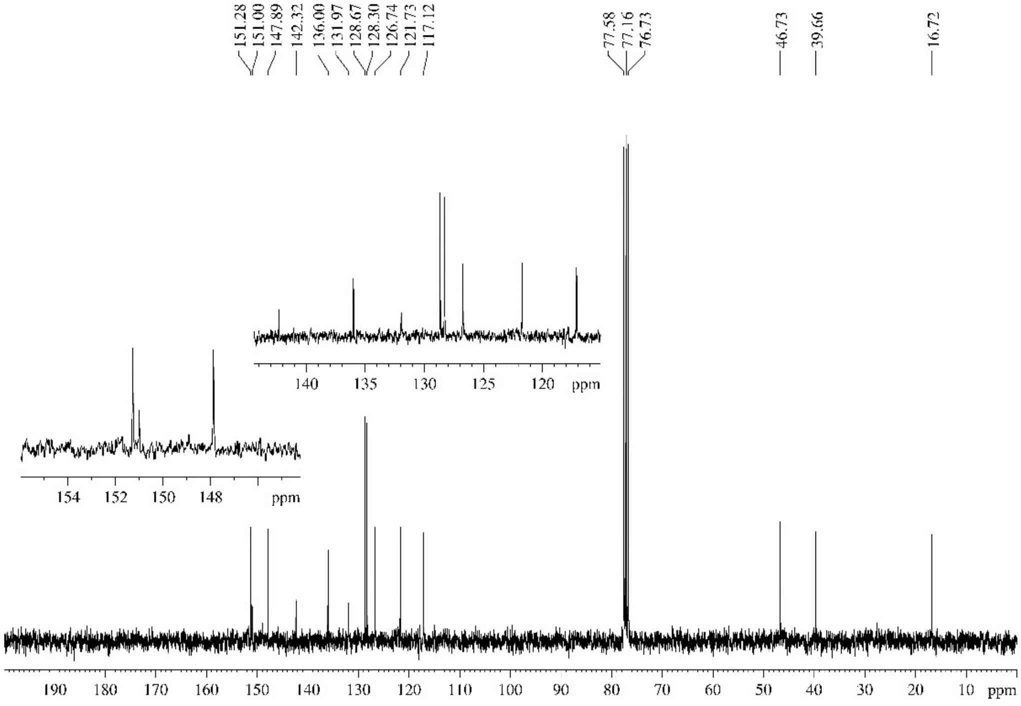

^{1}H (400 MHz, $CDCl_3$) and ^{13}C (101 MHz, $CDCl_3$) – NMR spectra of **22A**

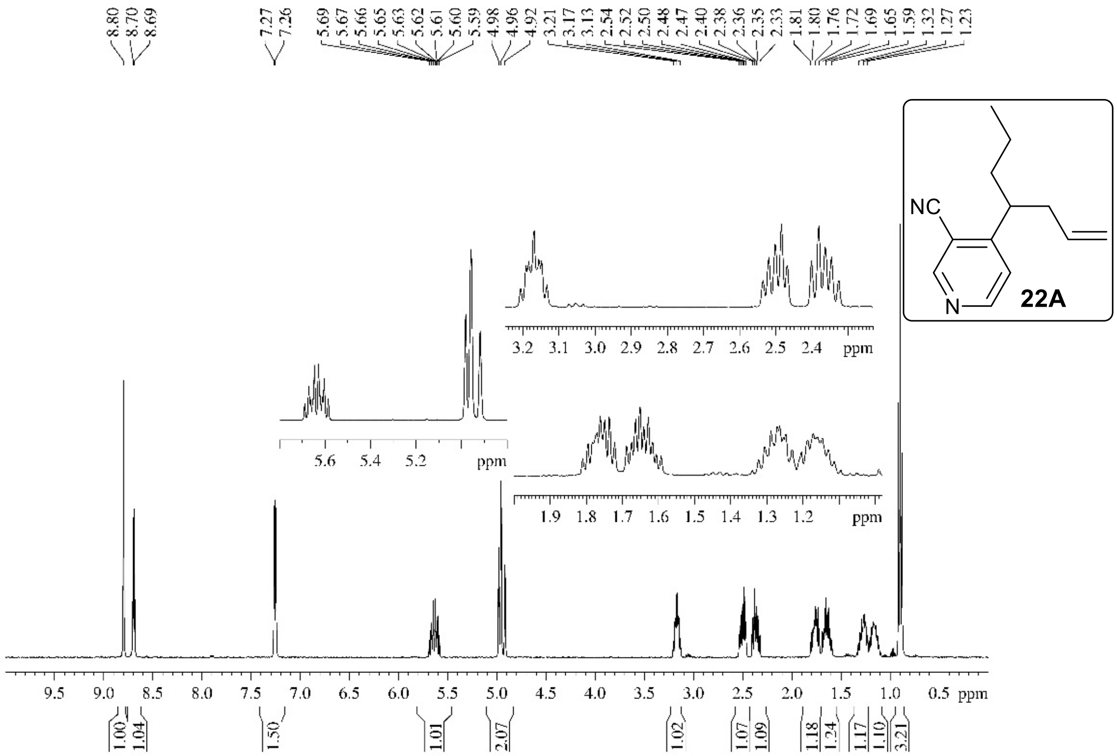

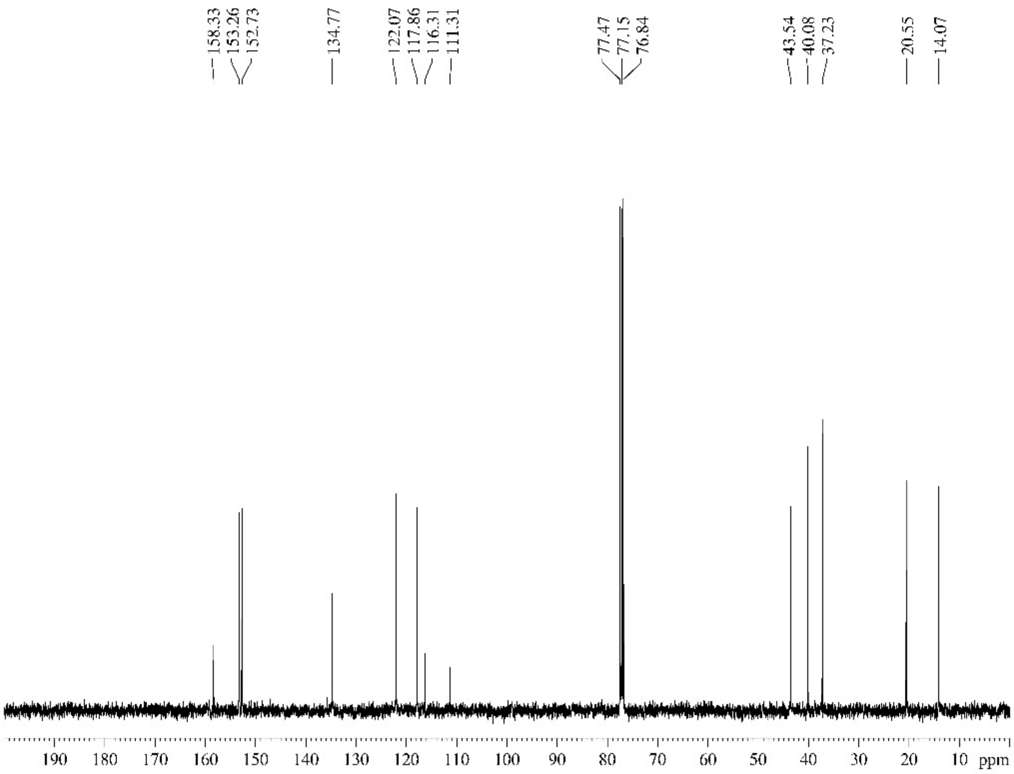

^{1}H (400 MHz, $CDCl_3$) and ^{13}C (101 MHz, $CDCl_3$) – NMR spectra of **23A**

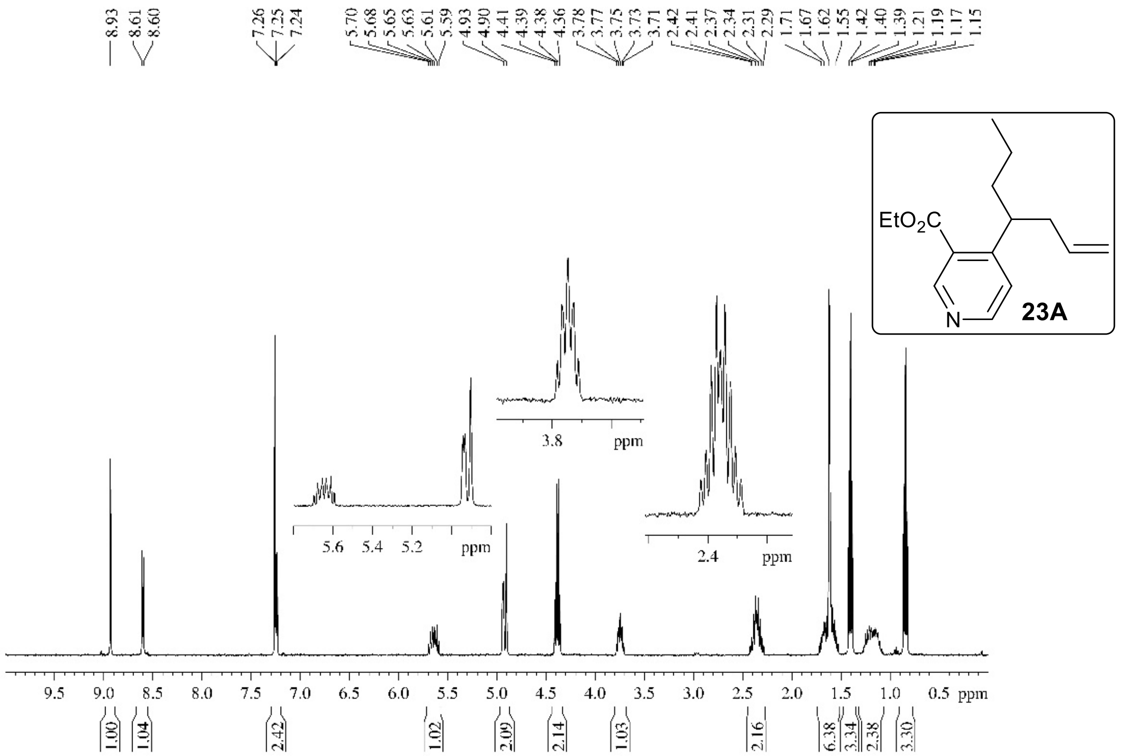

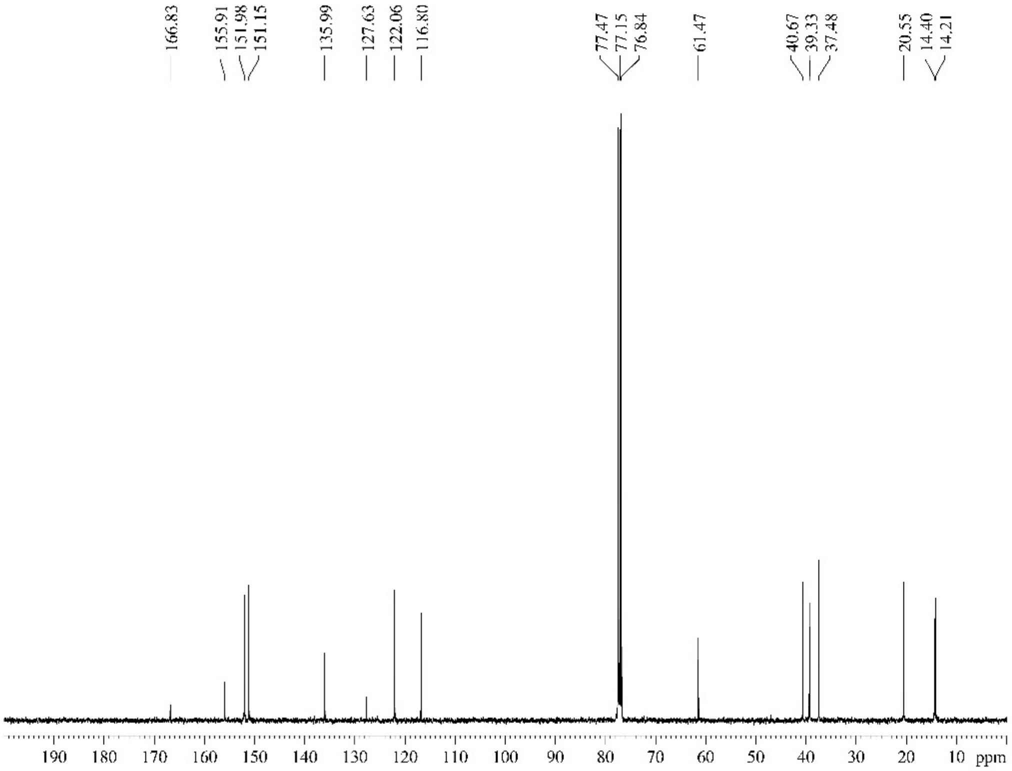

^{1}H (400 MHz, $CDCl_3$) and ^{13}C (101 MHz, $CDCl_3$) – NMR spectra of **24A**

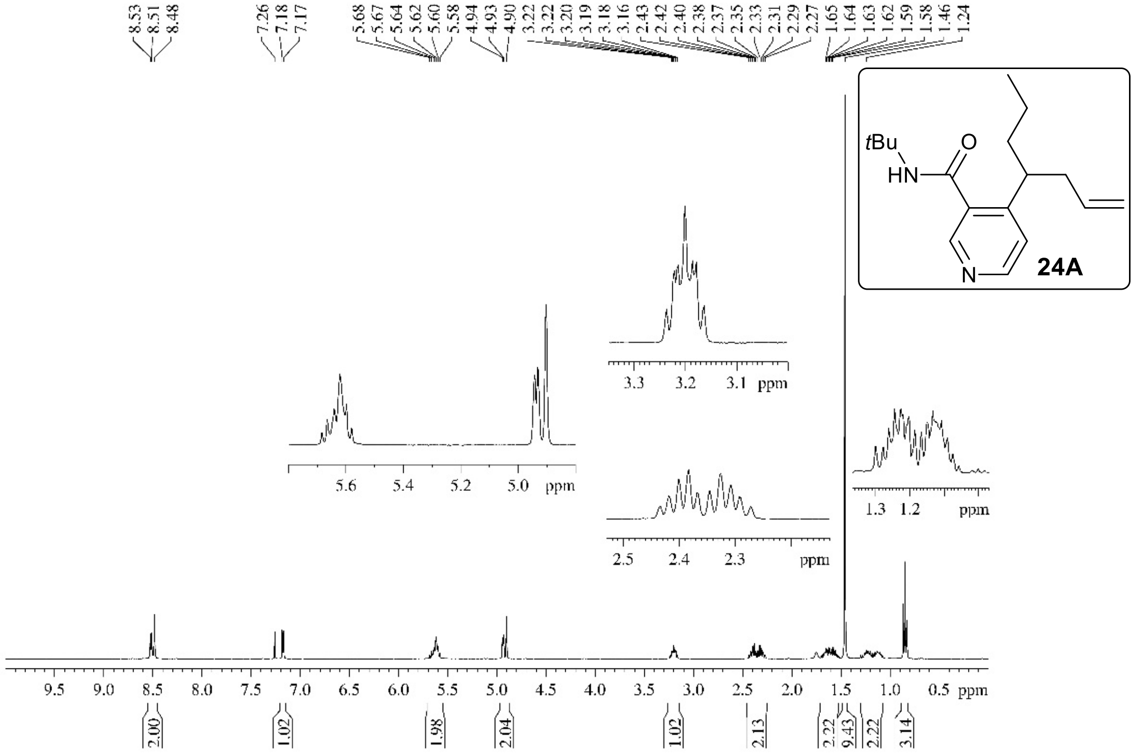

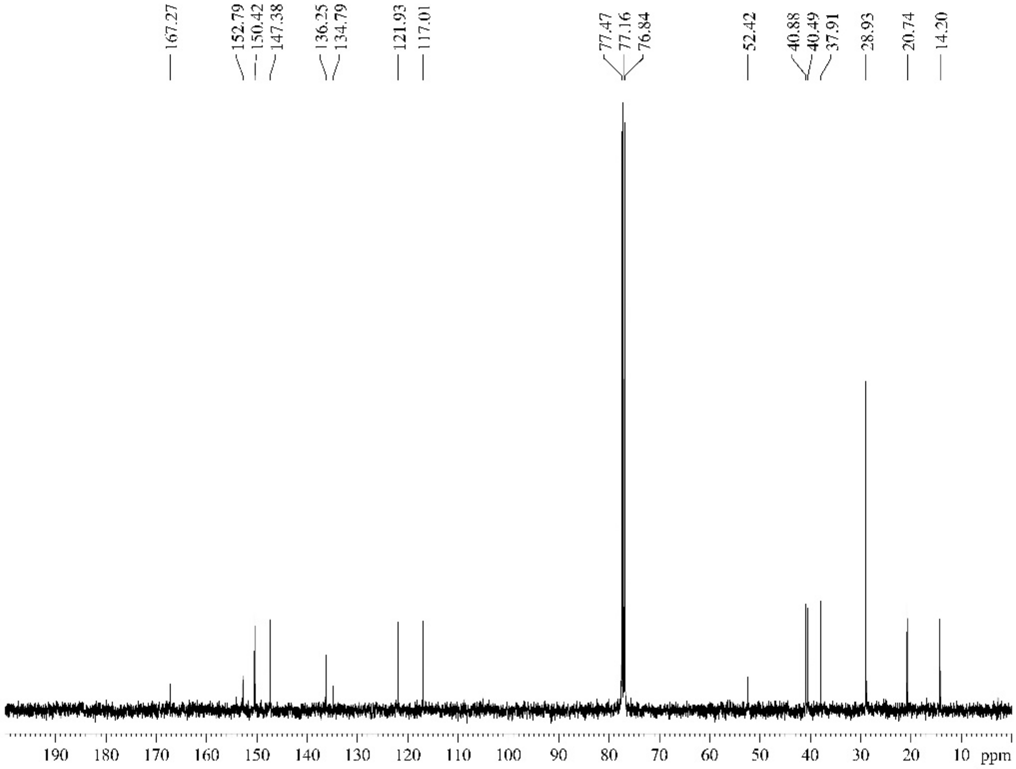

^{1}H (400 MHz, $CDCl_3$) and ^{13}C (101 MHz, $CDCl_3$) – NMR spectra of **25A**

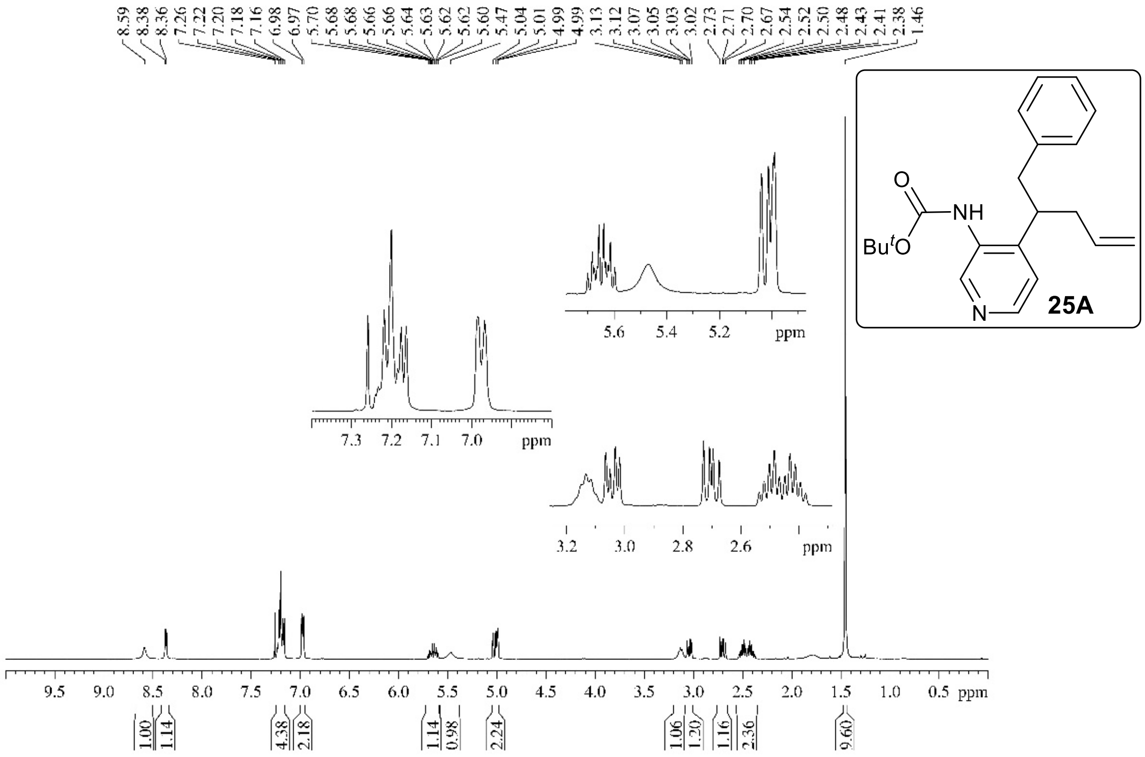

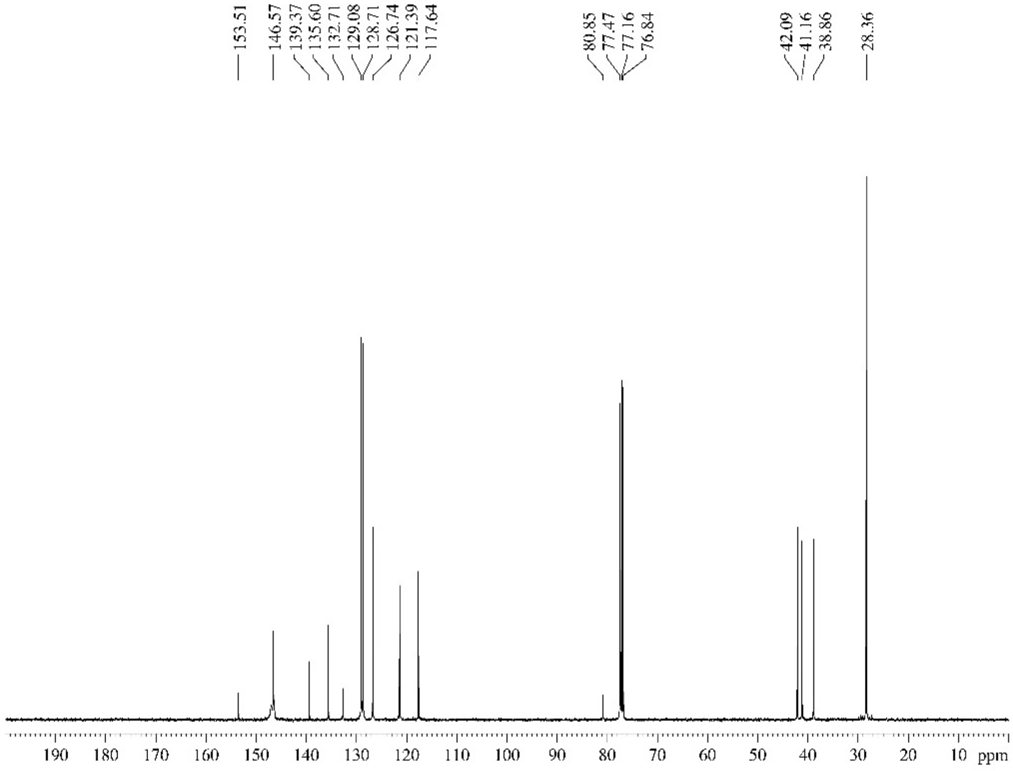

^{1}H (400 MHz, $CDCl_3$) and ^{13}C (101 MHz, $CDCl_3$) – NMR spectra of **26A**

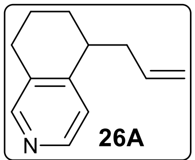

26A

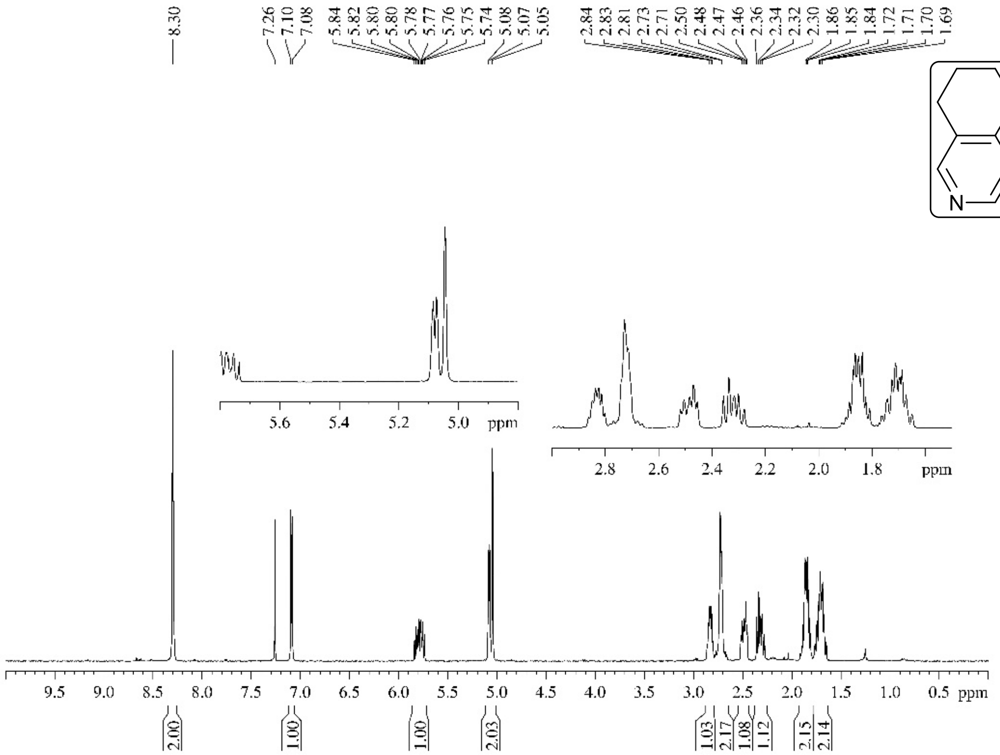

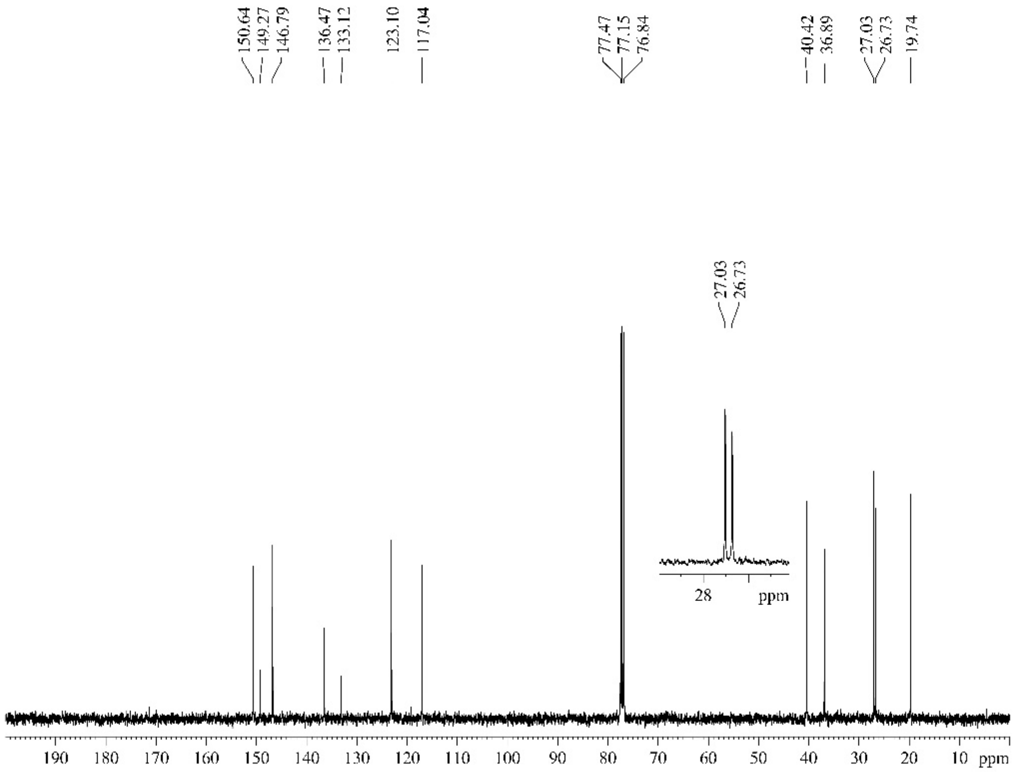

^{1}H (400 MHz, $CDCl_3$) and ^{13}C (101 MHz, $CDCl_3$) – NMR spectra of **27A**

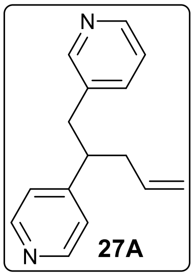

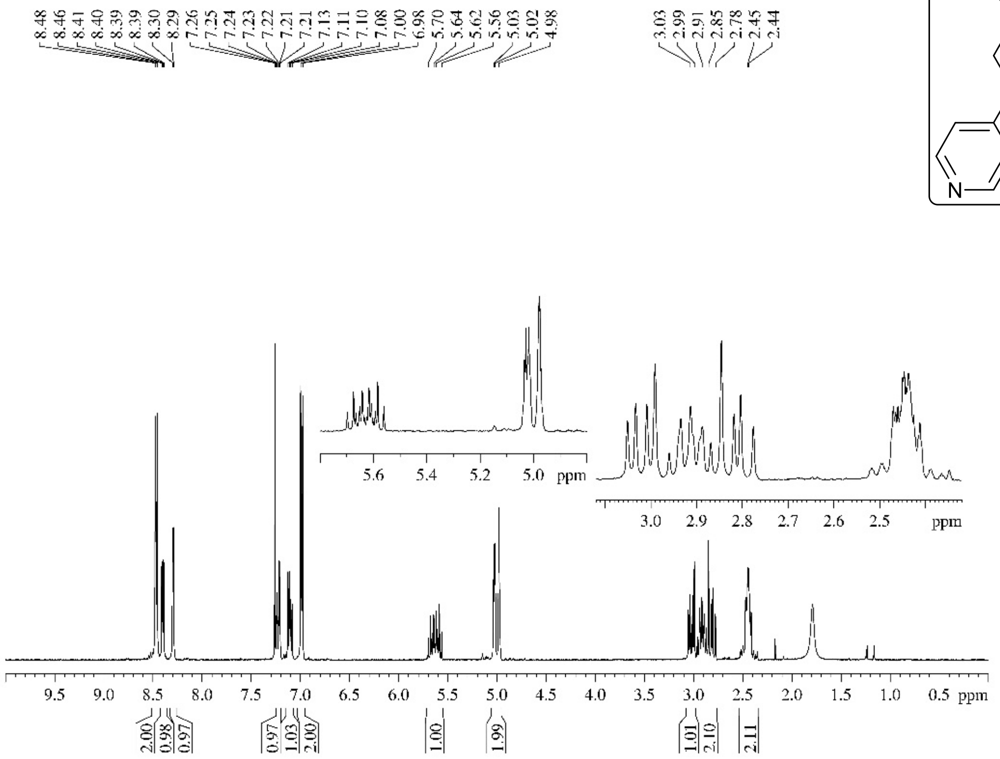

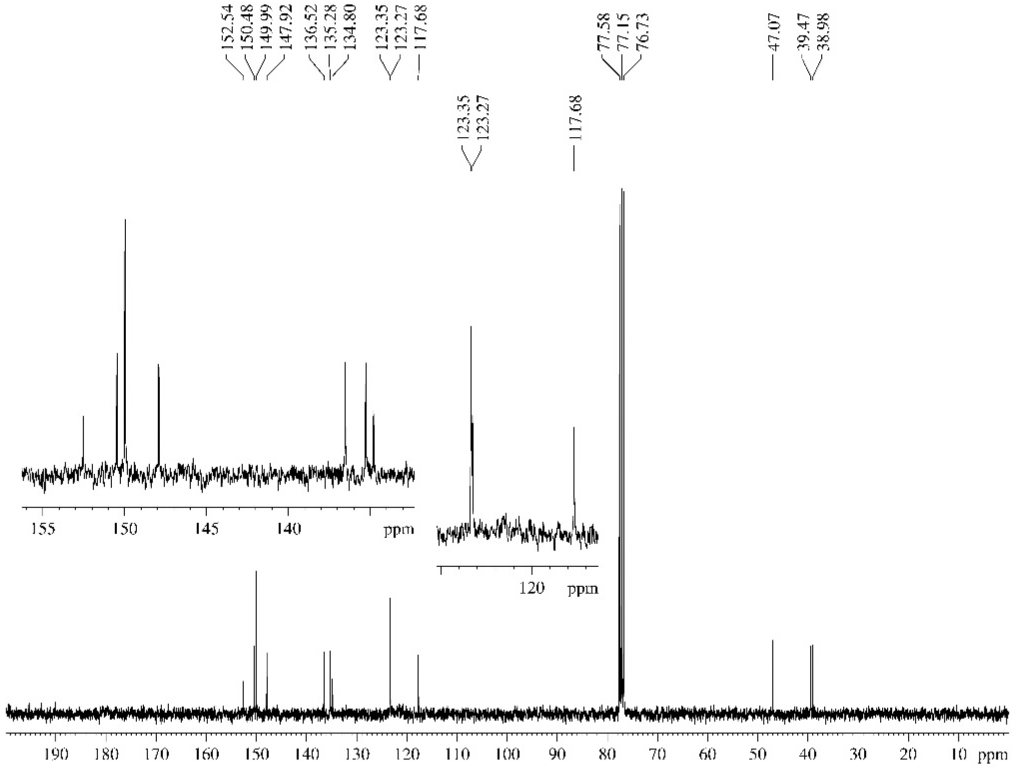

^{1}H (600 MHz, $CDCl_3$) and ^{13}C (151 MHz, $CDCl_3$) – NMR spectra of **28A**

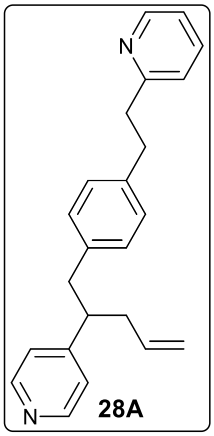

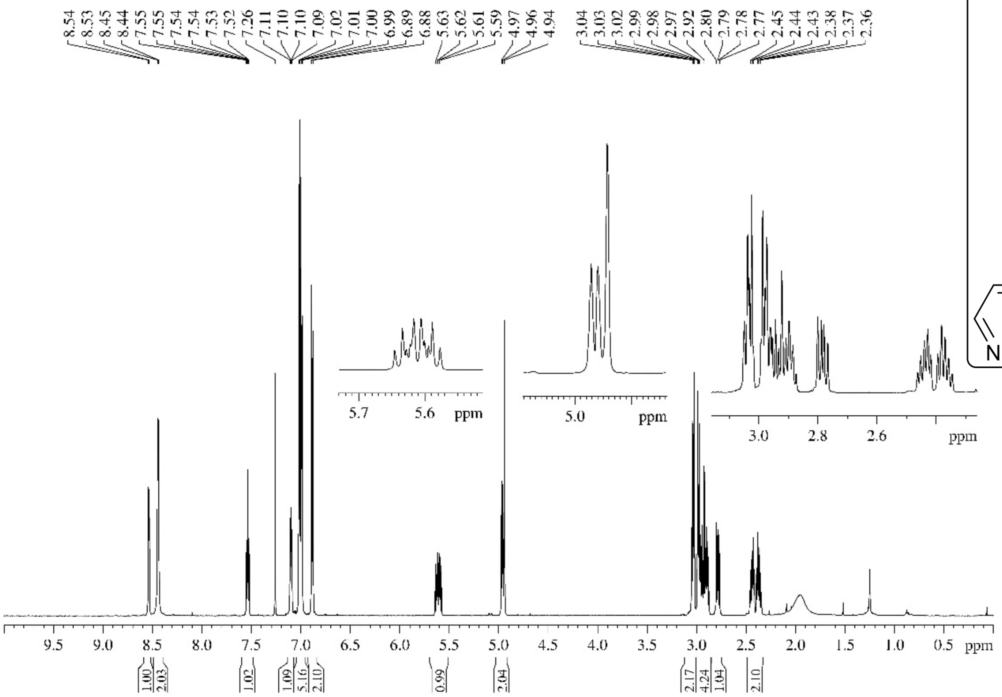

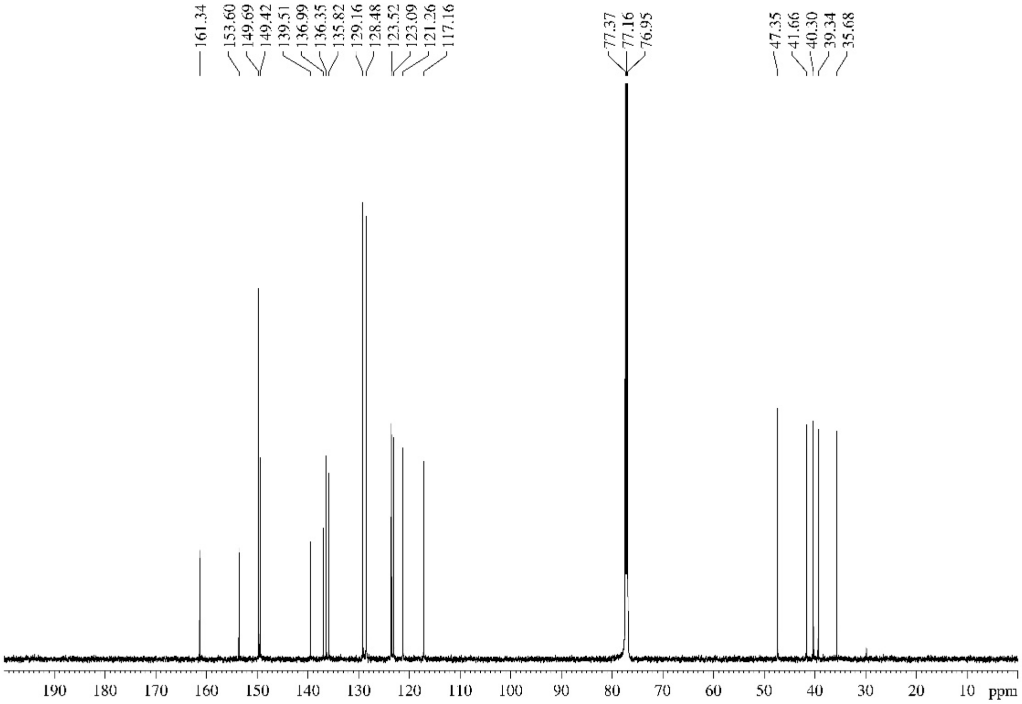

2D-COSY (600 MHz, $CDCl_3$) and 2D-HSQC (600 MHz, $CDCl_3$) – NMR spectra of **28A**

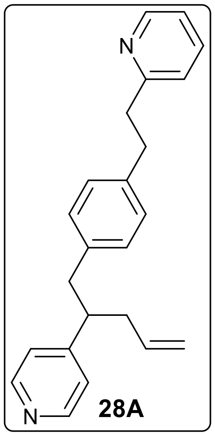

28A

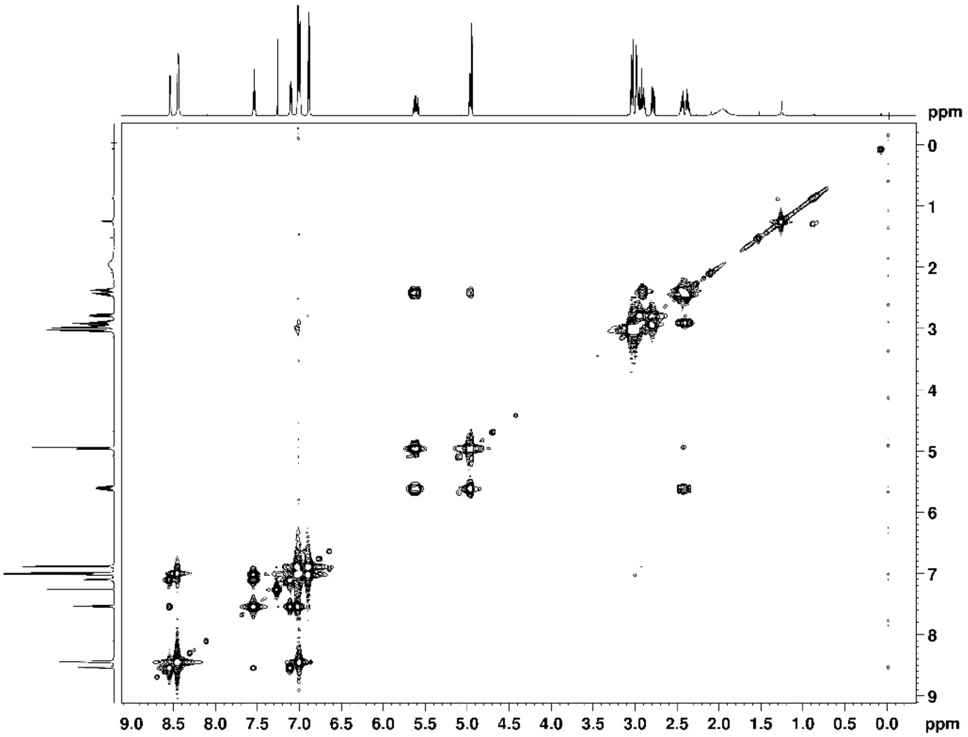

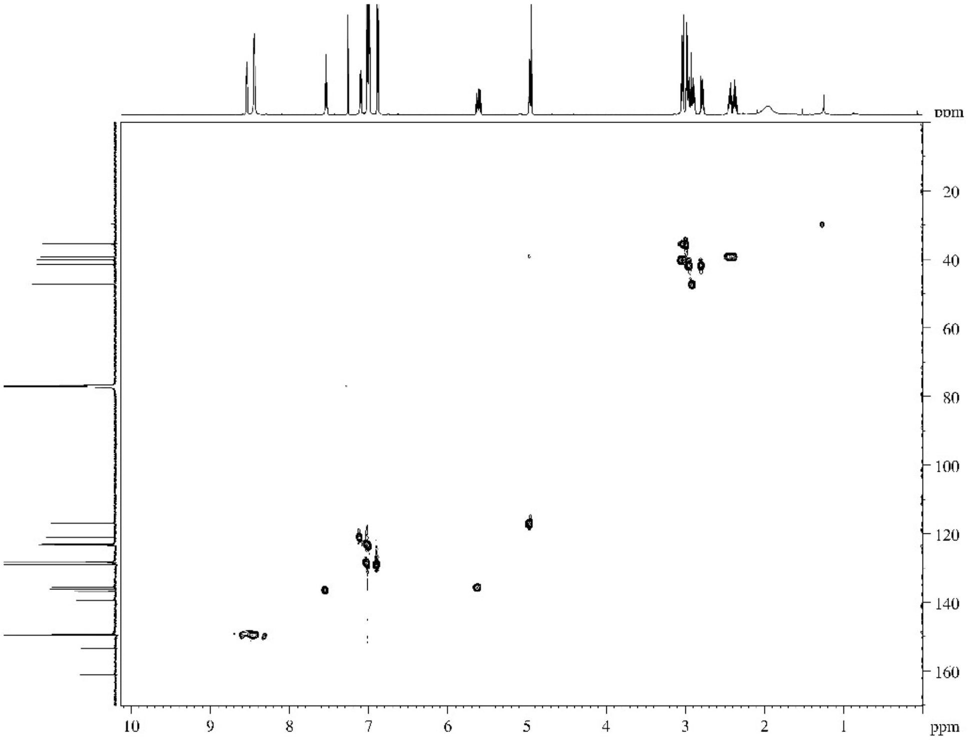

2D-HSQC (600 MHz, $CDCl_3$) – NMR spectra of **28A**

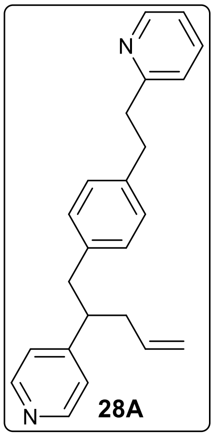

28A

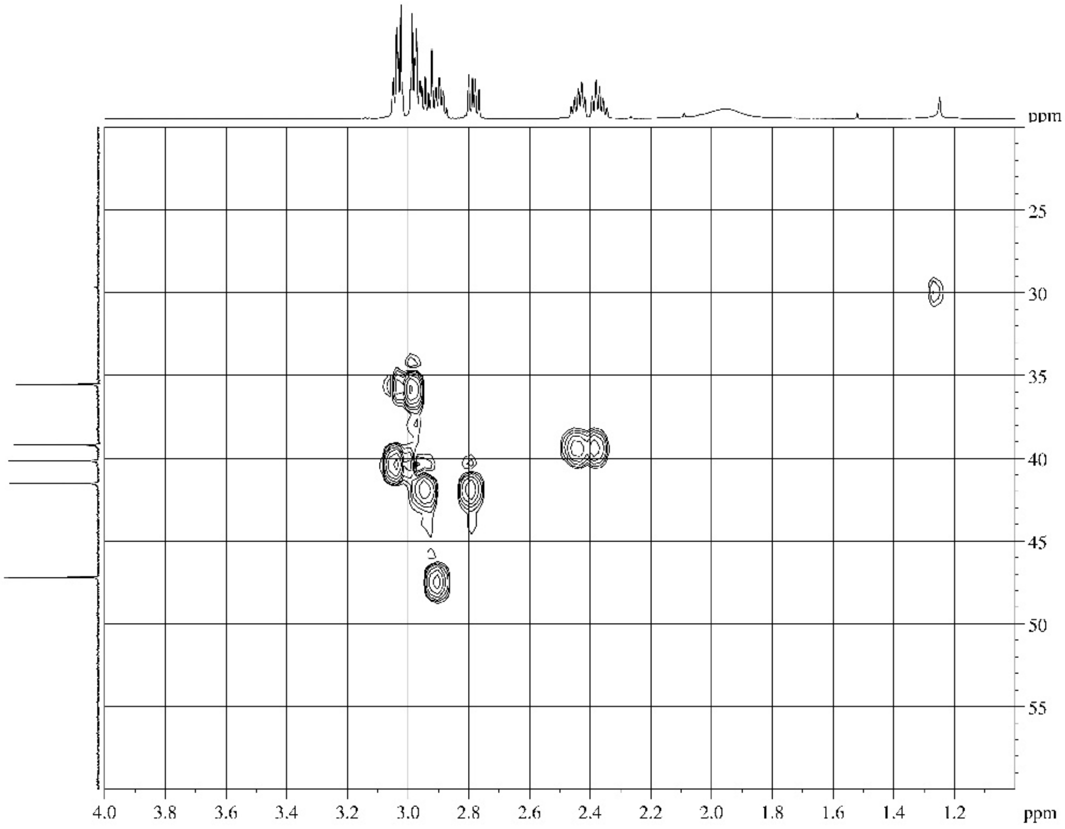

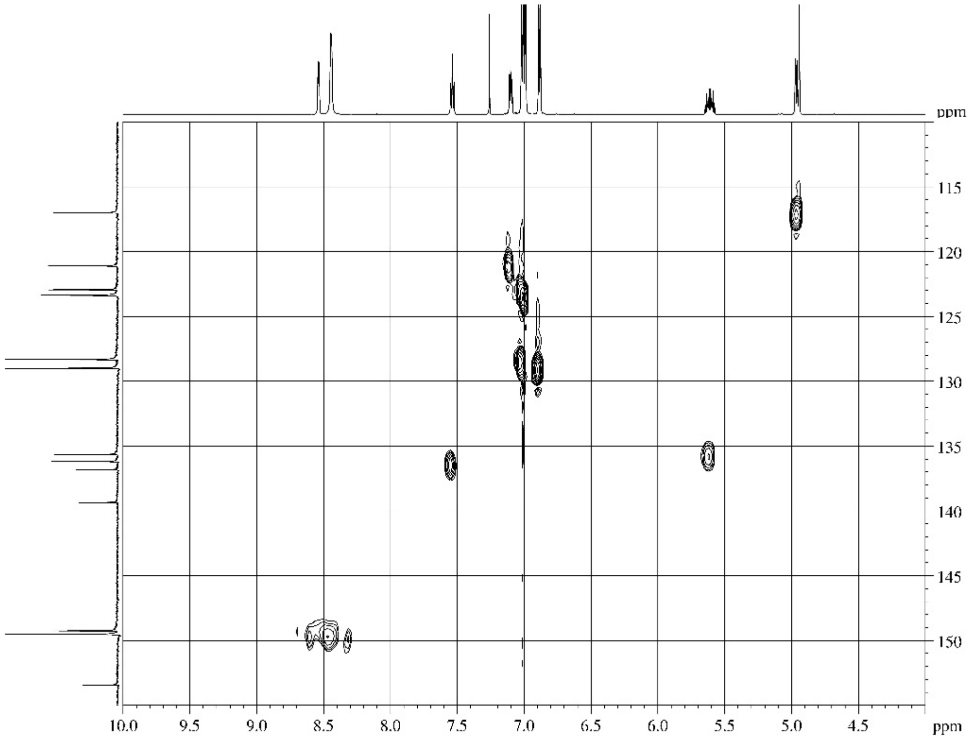

2D-HMBC (600 MHz, $CDCl_3$) – NMR spectra of **28A**

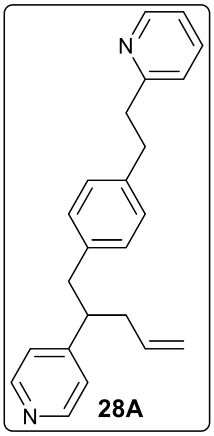

28A

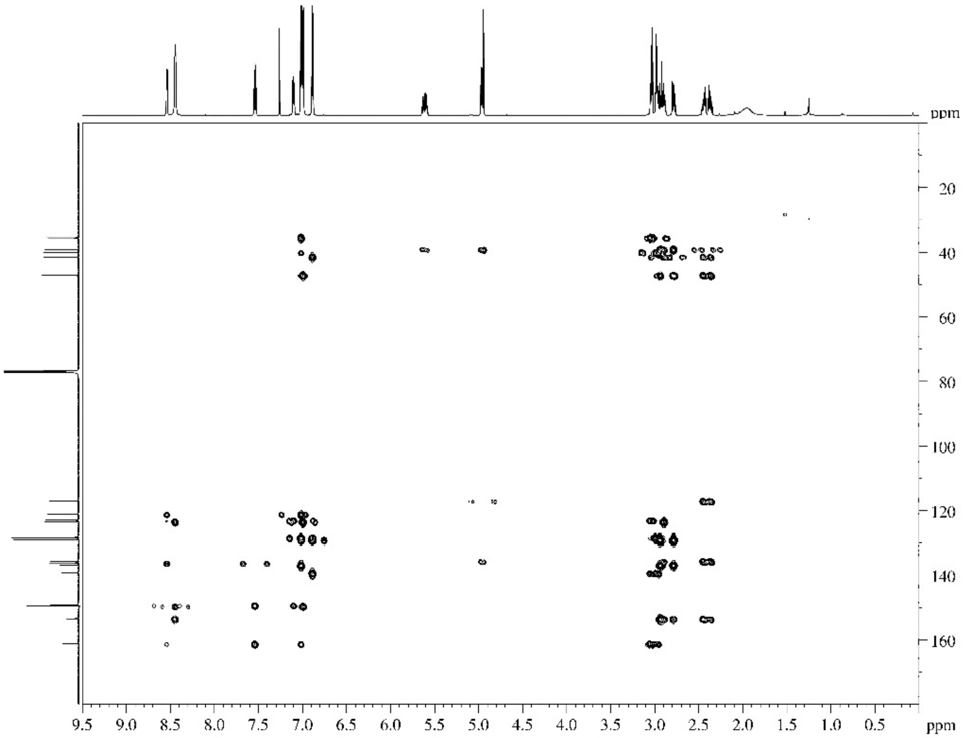

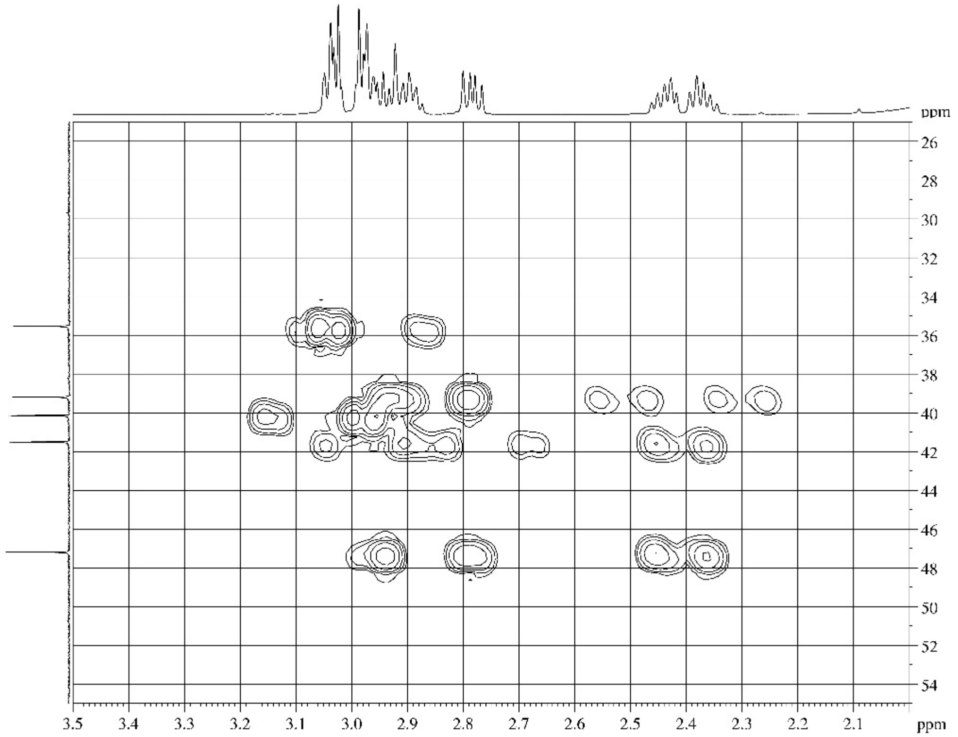

2D-HMBC (600 MHz, $CDCl_3$) – NMR spectra of **28A**

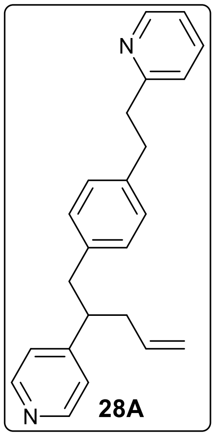

28A

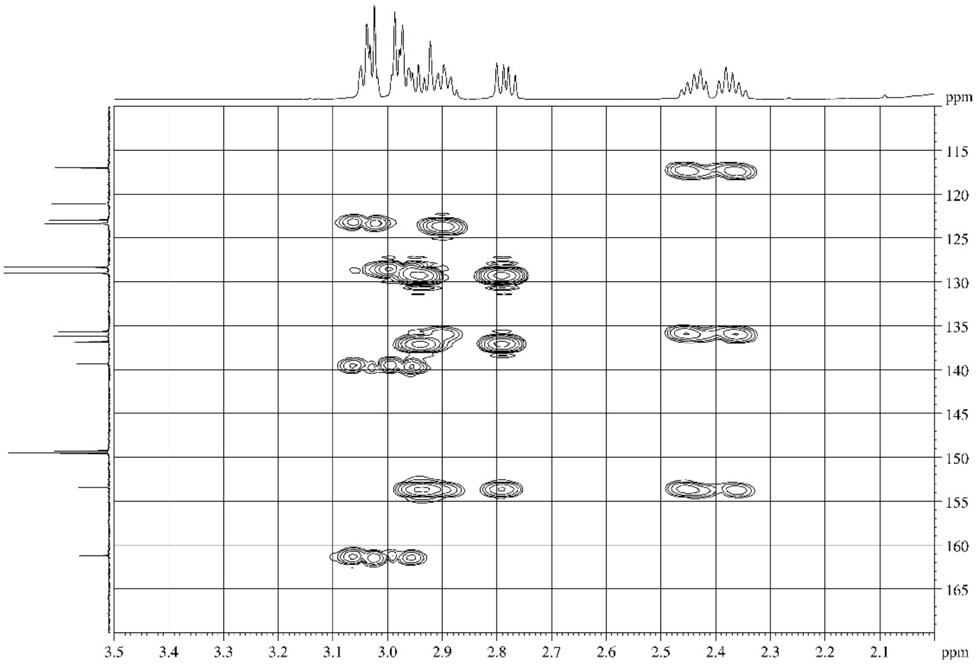

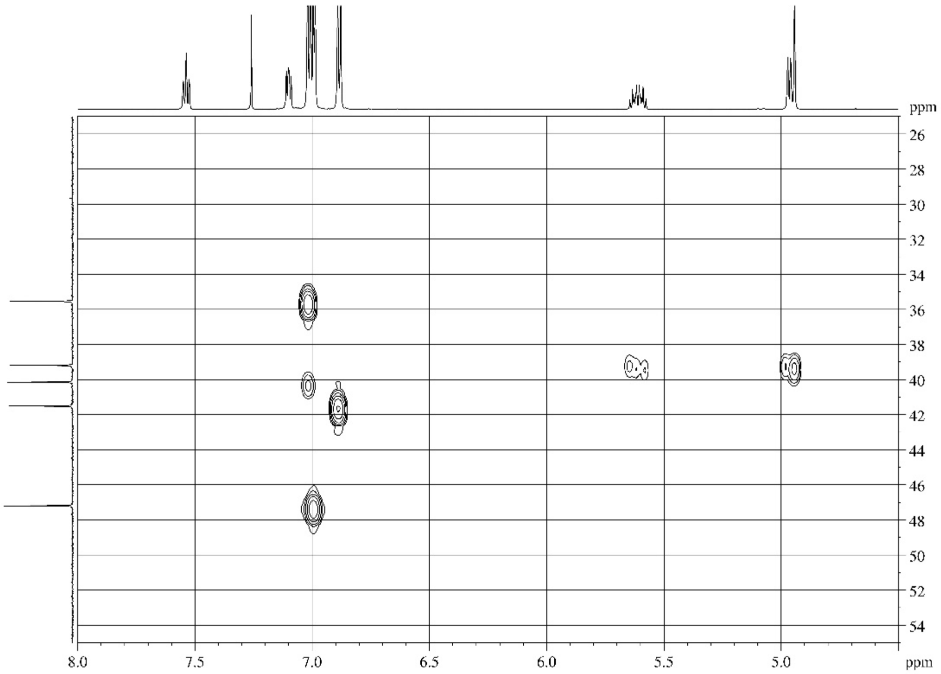

2D-HMBC (600 MHz, $CDCl_3$) – NMR spectra of **28A**

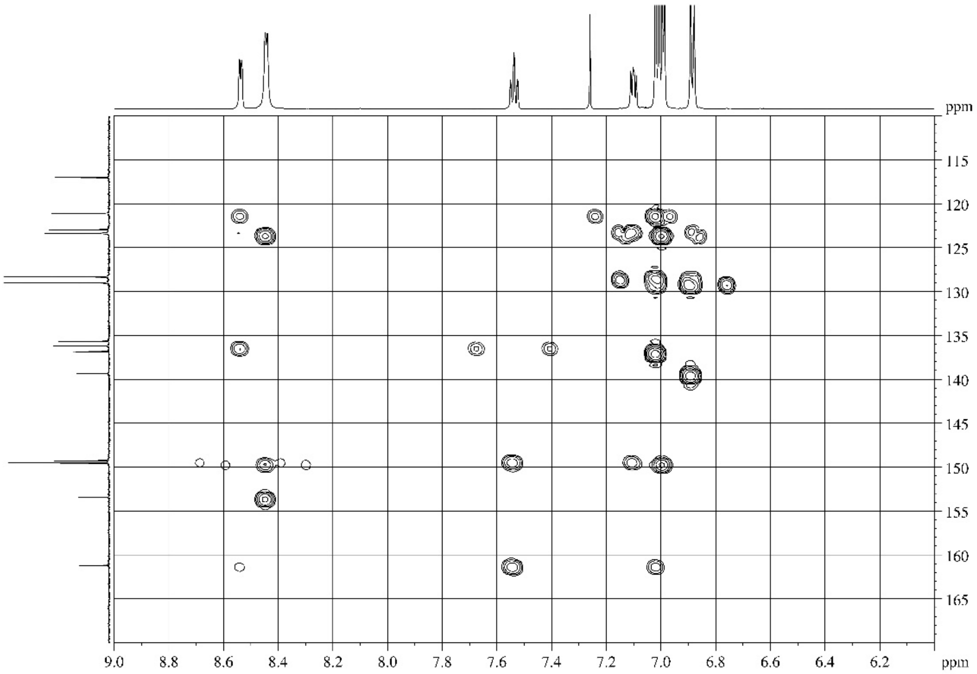

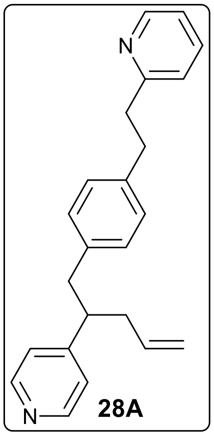

28A

^{1}H (300 MHz, $CDCl_3$, 45 °C) and ^{13}C (76 MHz, $CDCl_3$, 45 °C) – NMR spectra of **29’**

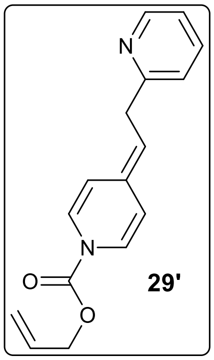

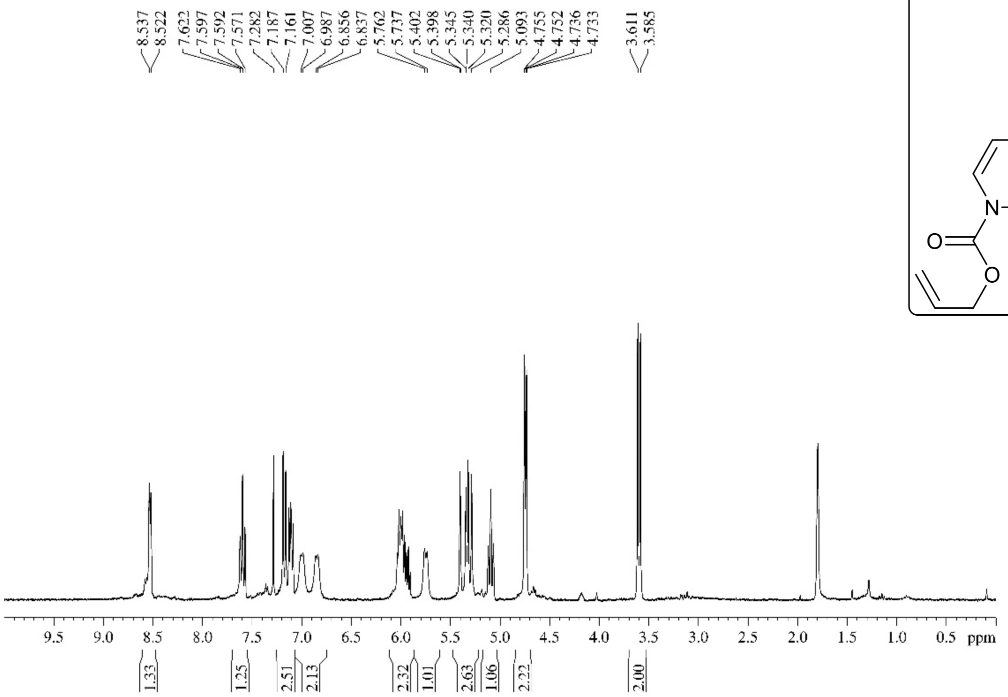

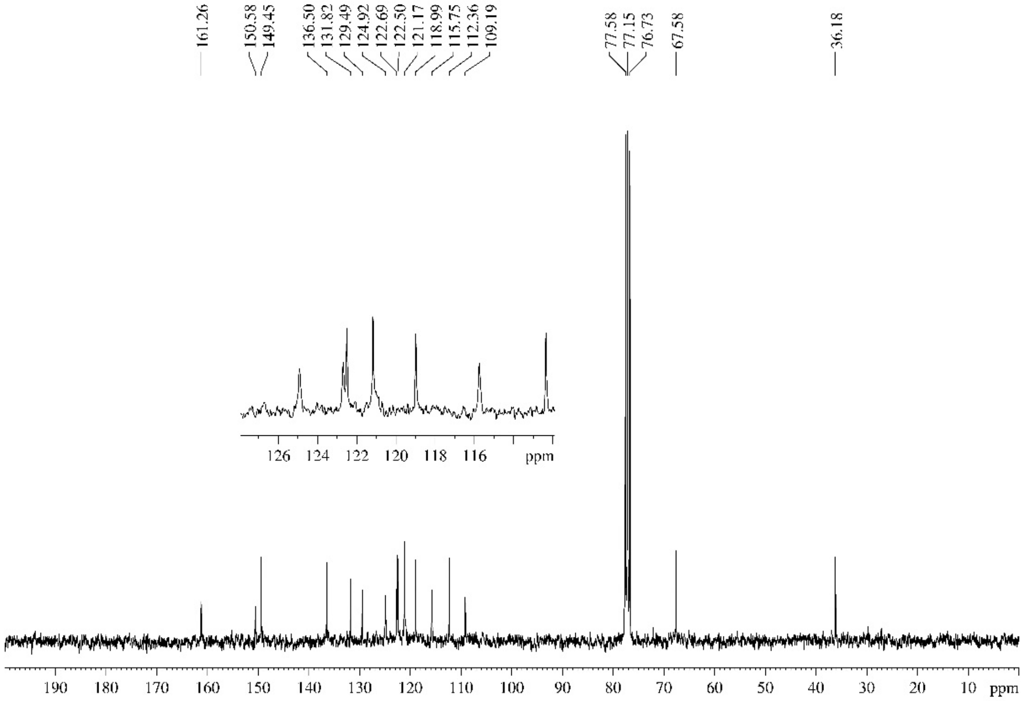

^{1}H (300 MHz, $CDCl_3$) and ^{13}C (76 MHz, $CDCl_3$) – NMR spectra of **30A**

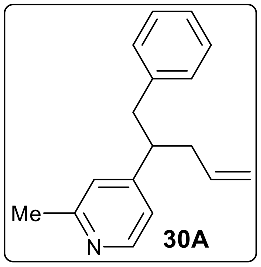

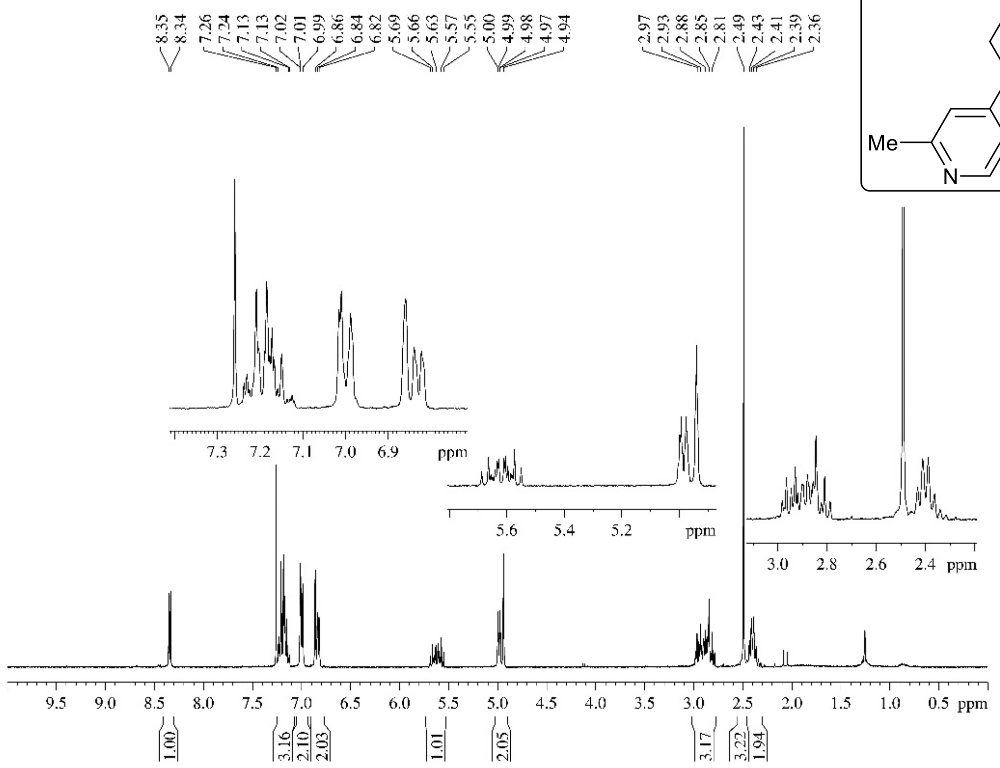

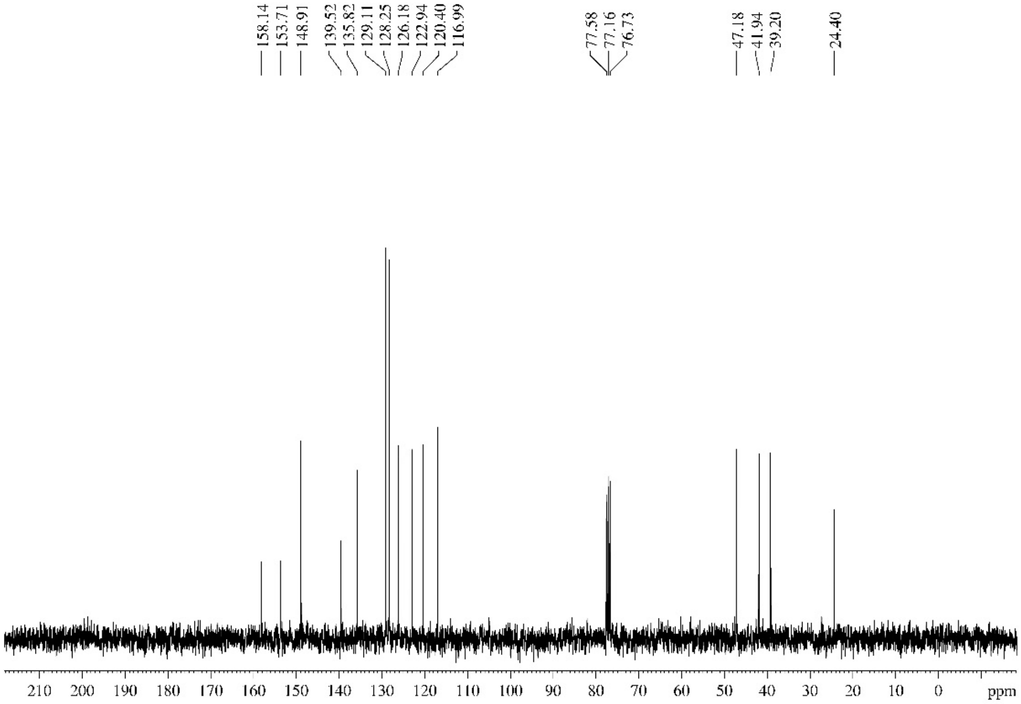

^{1}H (400 MHz, $CDCl_3$) and ^{13}C (101 MHz, $CDCl_3$) – NMR spectra of **1A (CH_3)**

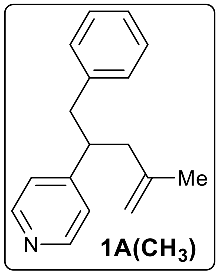

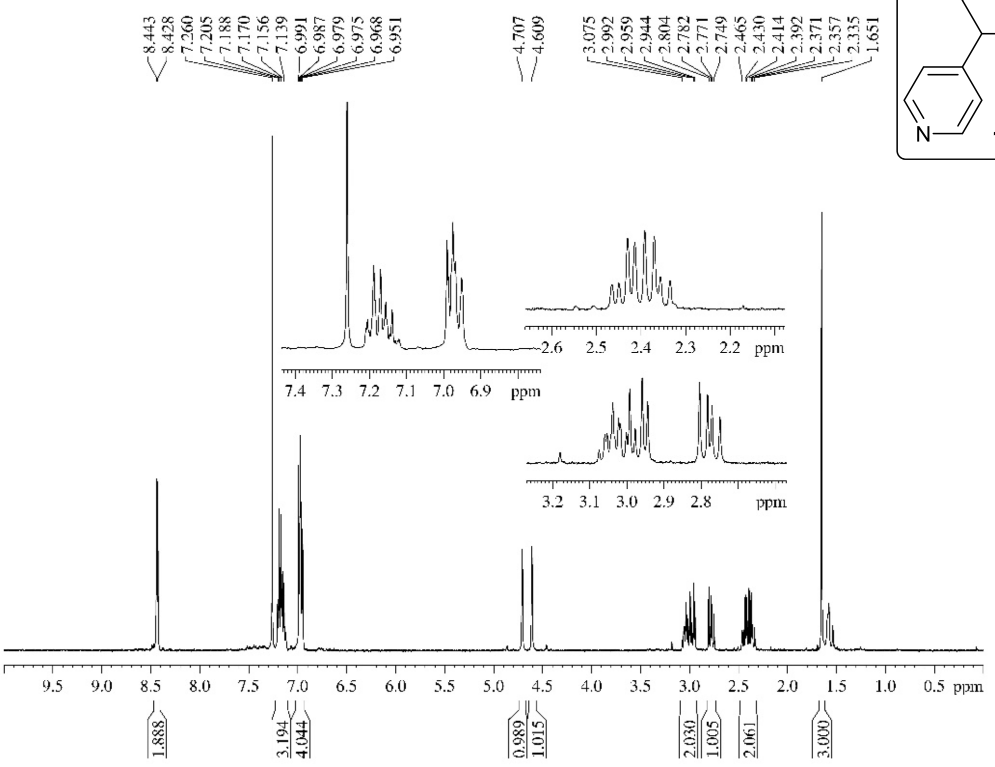

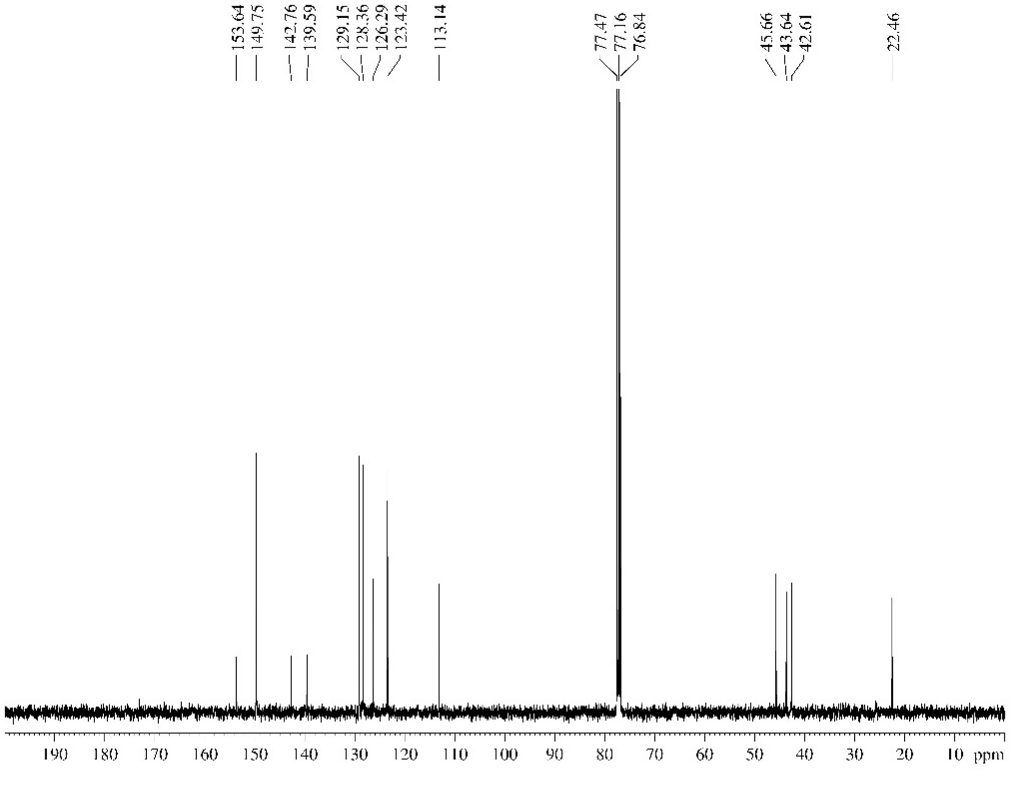

^{1}H (400 MHz, $CDCl_3$) and ^{2}H (61 MHz, $CDCl_3$) – NMR spectra of **1A (CD$_3$)**

C<u>D</u>$_3$ observed as a singlet in ^{2}H NMR at 1.62 ppm

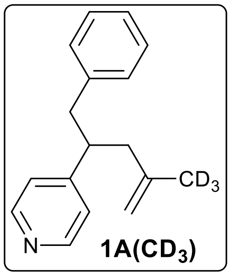

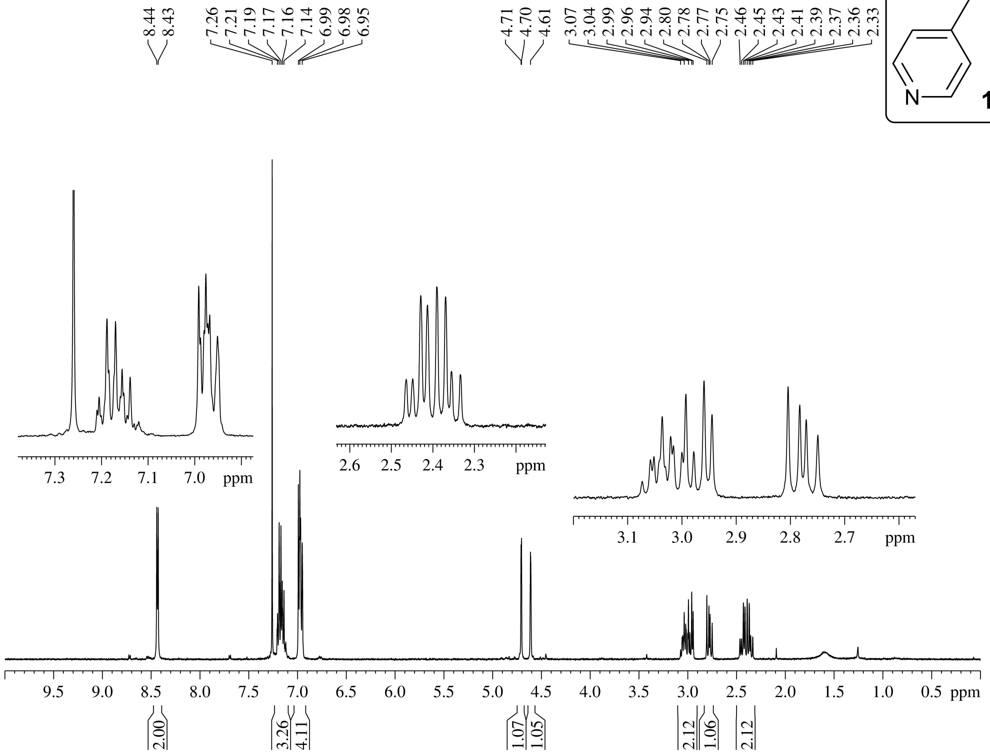

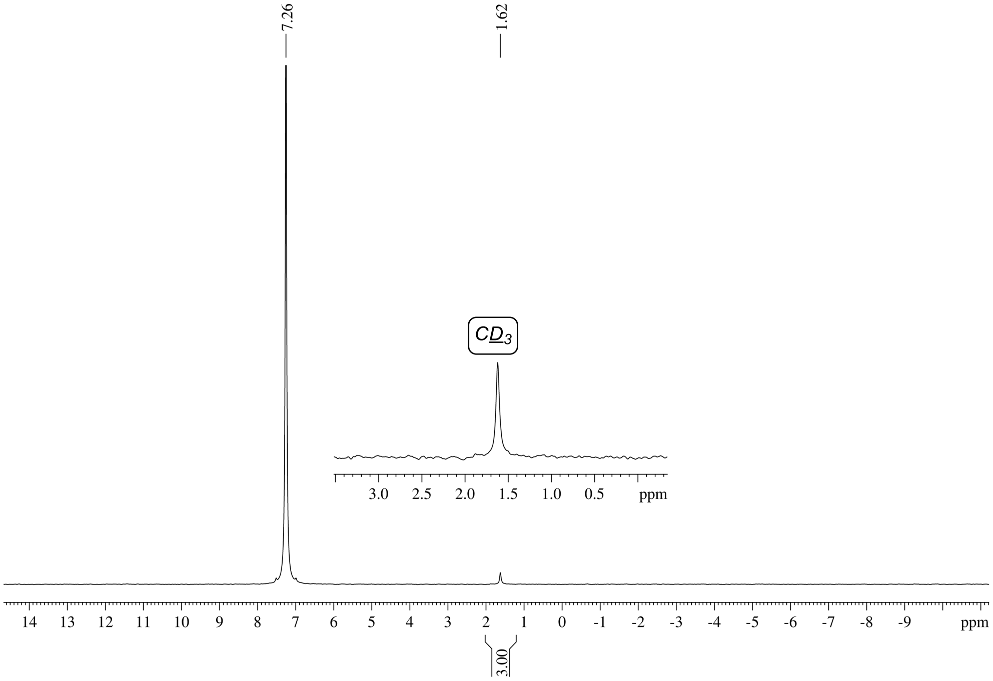

^{13}C (101 MHz, $CDCl_3$) and ^{13}C (151 MHz, $CDCl_3$) – NMR spectra of **1A (CD_3)**

<u>*C*</u>*D*$_3$ observed as a septet at 21.6 ppm with a coupling constant J = 18.1 Hz.

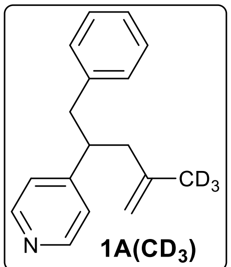

1A(CD$_3$)

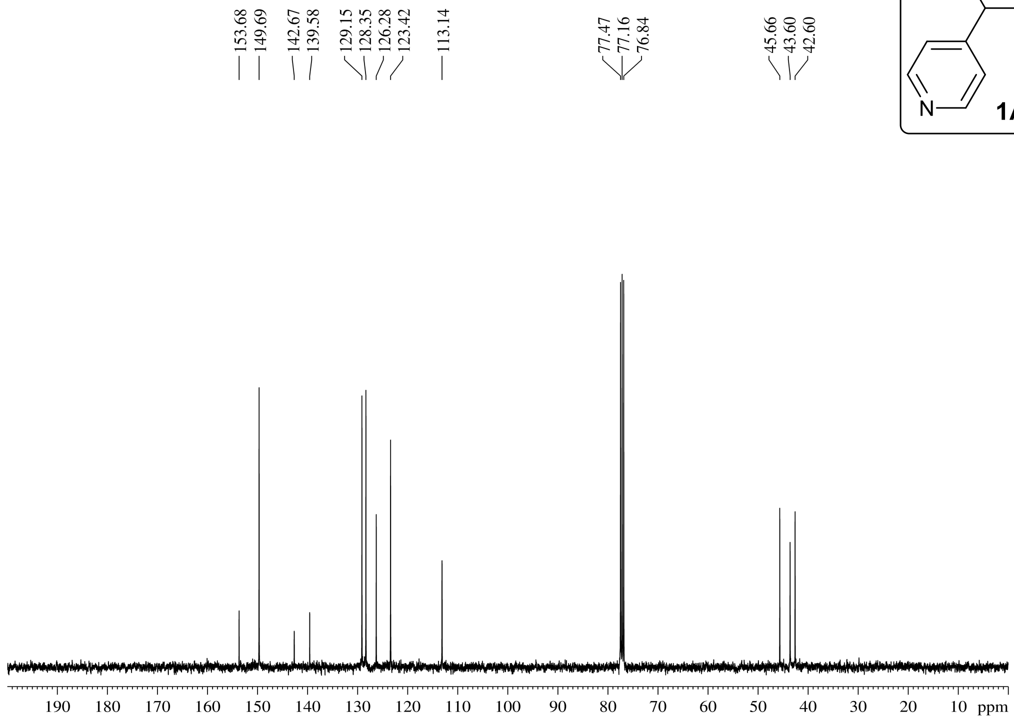

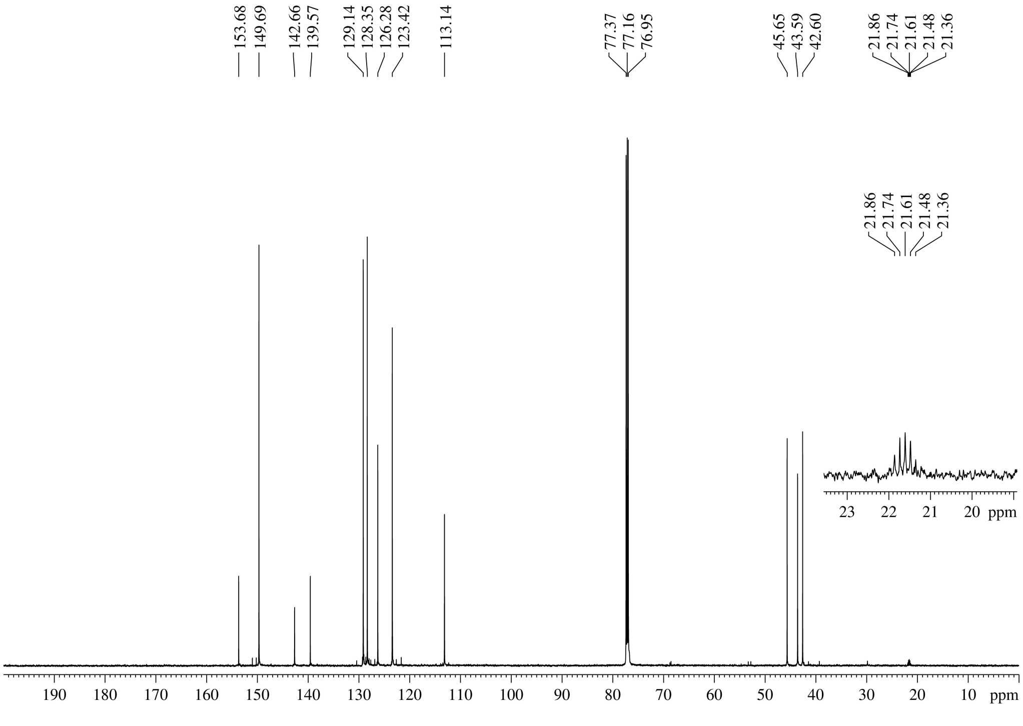

2D HMBC (600 MHz, $CDCl_3$) – NMR spectra of **1A (CD_3)**

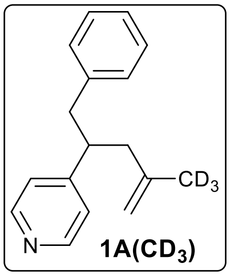

The *CD_3* peak at 21.6 ppm correlated to the allylic & vinylic protons as shown by HMBC.

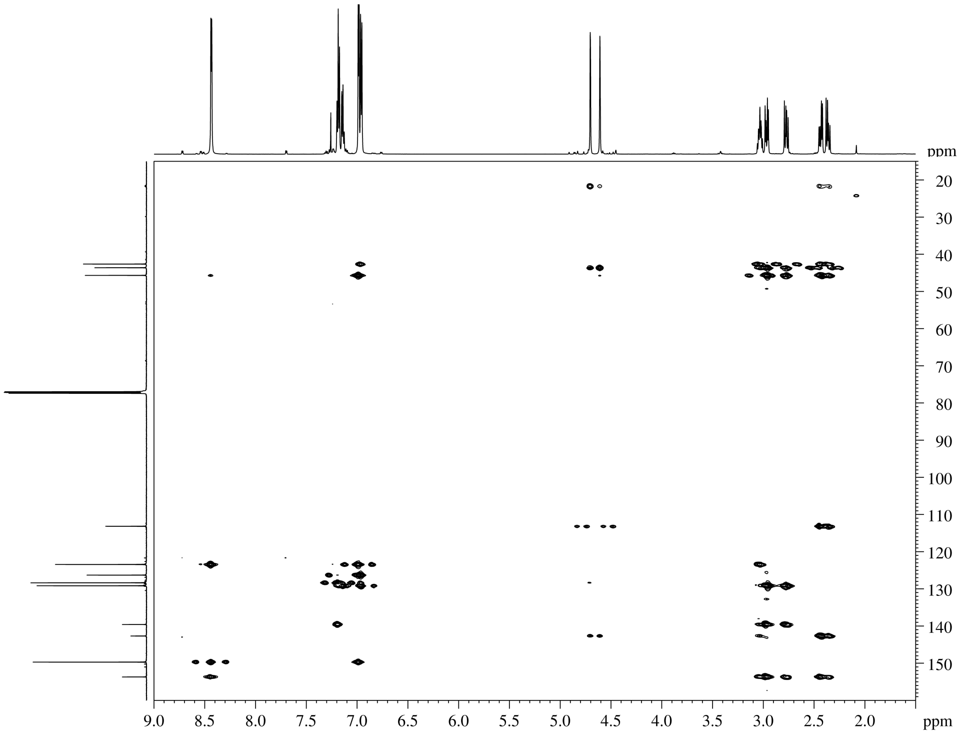

5

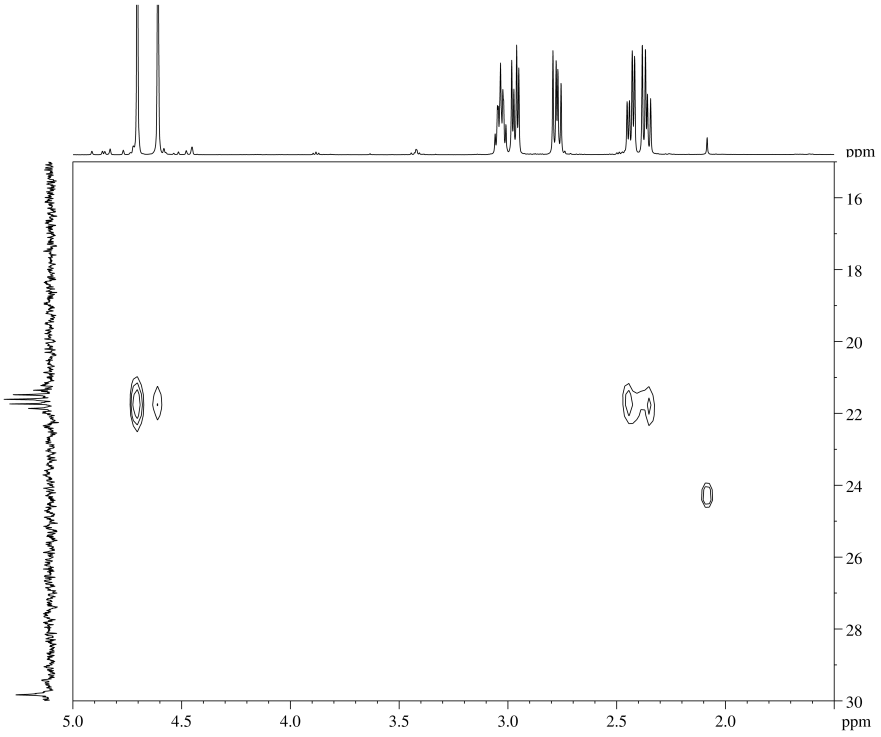

^{1}H (400 MHz, $CDCl_3$) and ^{13}C (101 MHz, $CDCl_3$) – NMR spectra of **2A (CH$_3$)**

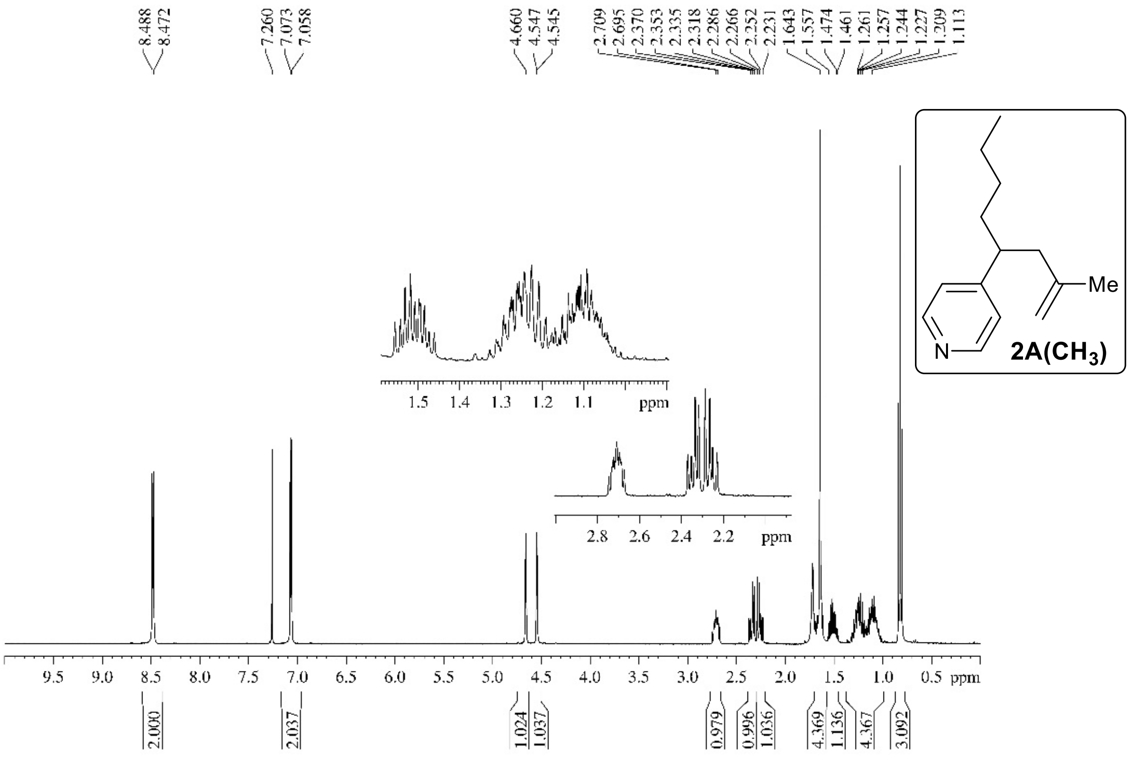

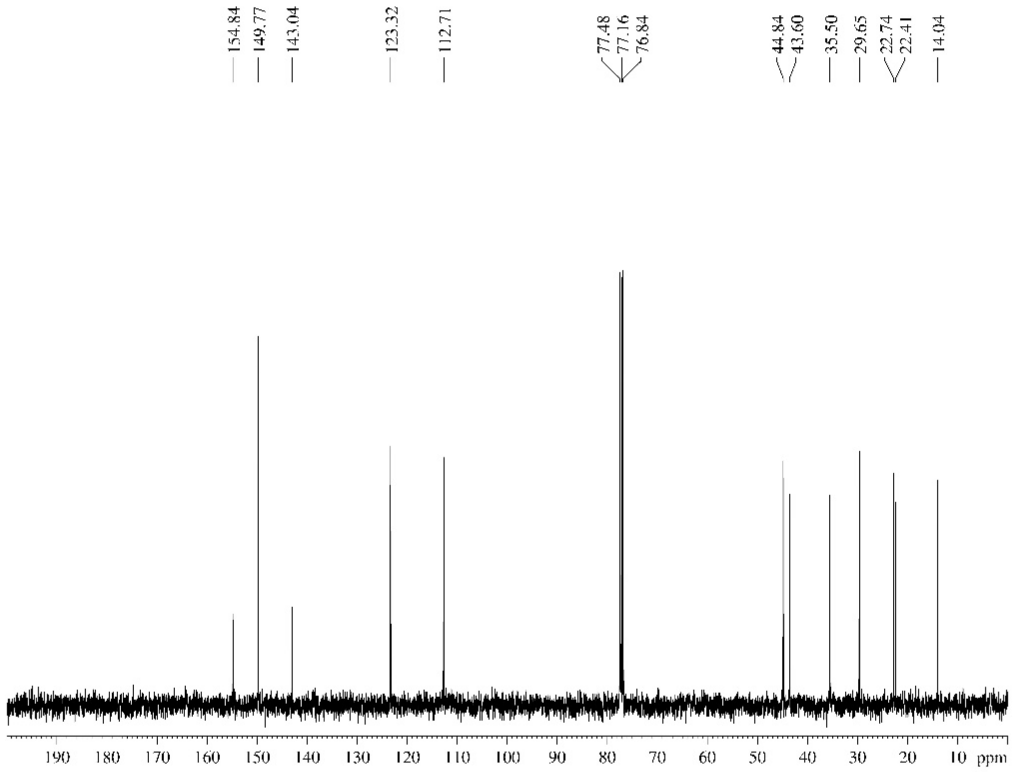

^{1}H (300 MHz, $CDCl_3$) and ^{2}H (46 MHz, $CDCl_3$) – NMR spectra of **2A (CD_3)**

$C\underline{D}_3$ observed as a singlet in ^{2}H NMR at 1.62 ppm

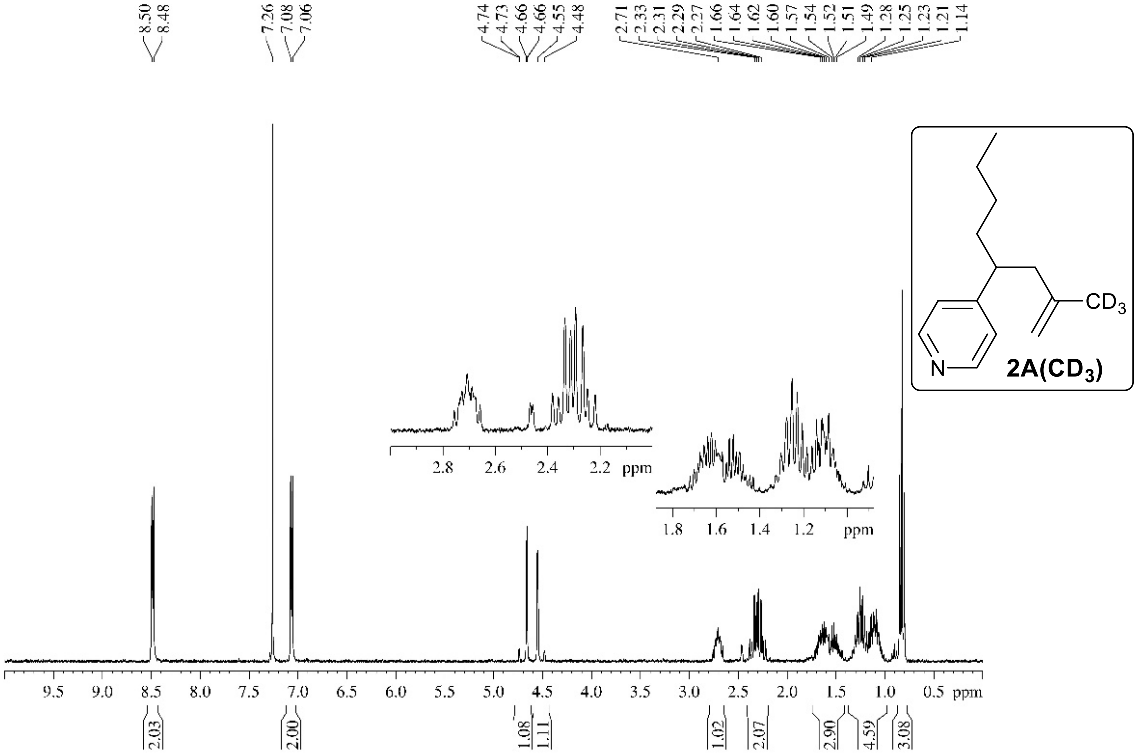

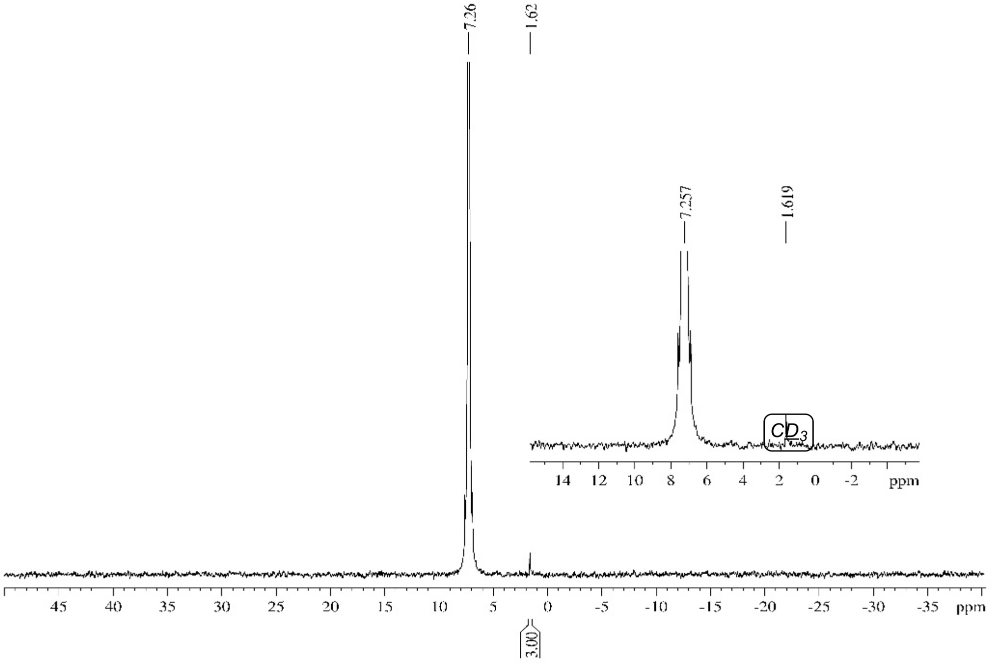

^{13}C (101 MHz, $CDCl_3$) and ^{13}C (151 MHz, $CDCl_3$) – NMR spectra of **2A (CD_3)**

$\underline{C}D_3$ observed as a septet at 21.8 ppm with a coupling constant J = 19.6 Hz.

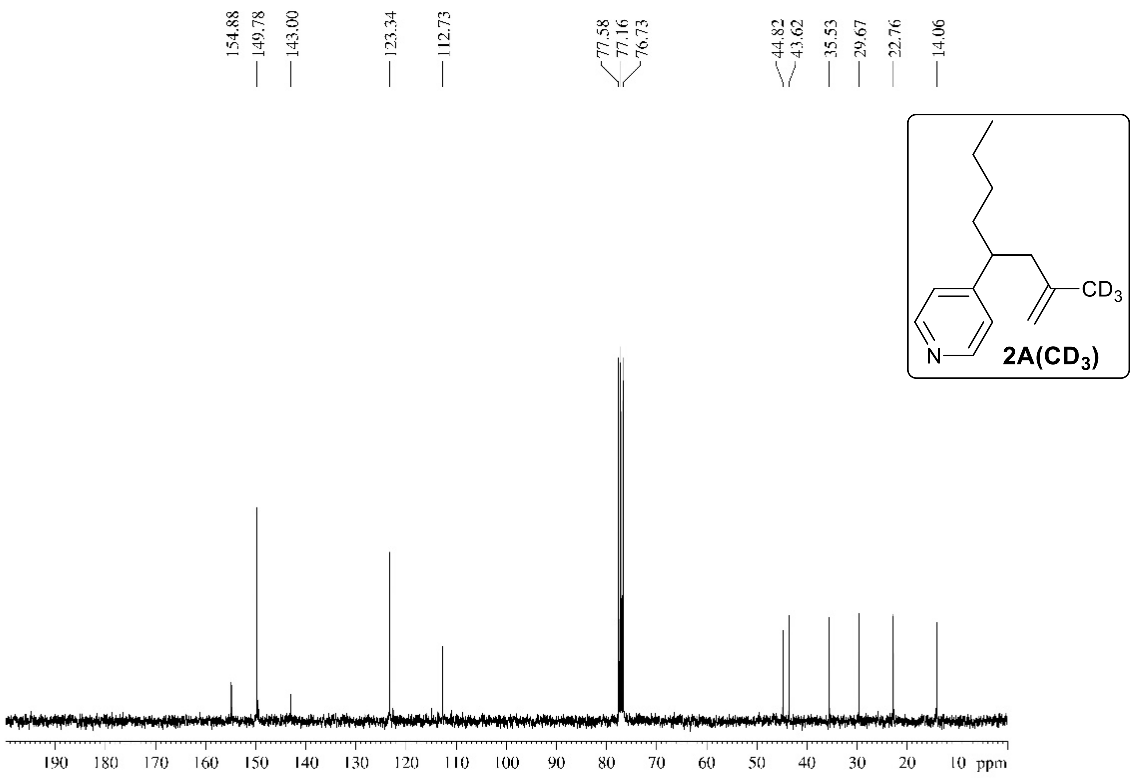

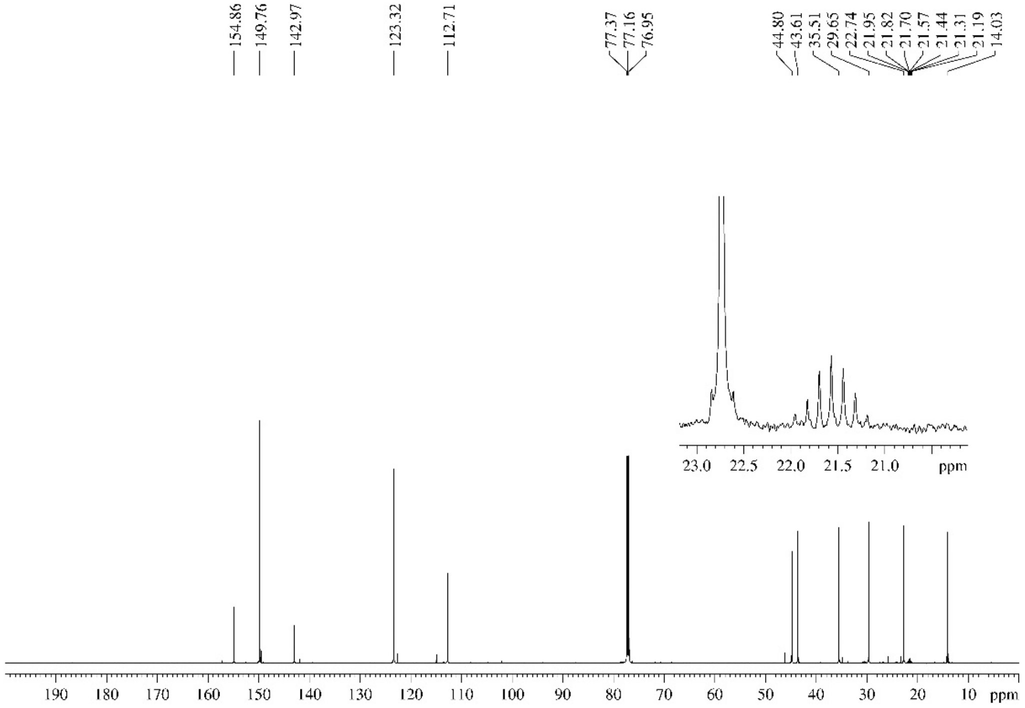

2D HMBC (600 MHz, $CDCl_3$) – NMR spectra of **2A (CD_3)**

The <u>*C*</u>*D_3* peak at 21.8 ppm correlated to the allylic & vinylic protons as shown by HMBC.

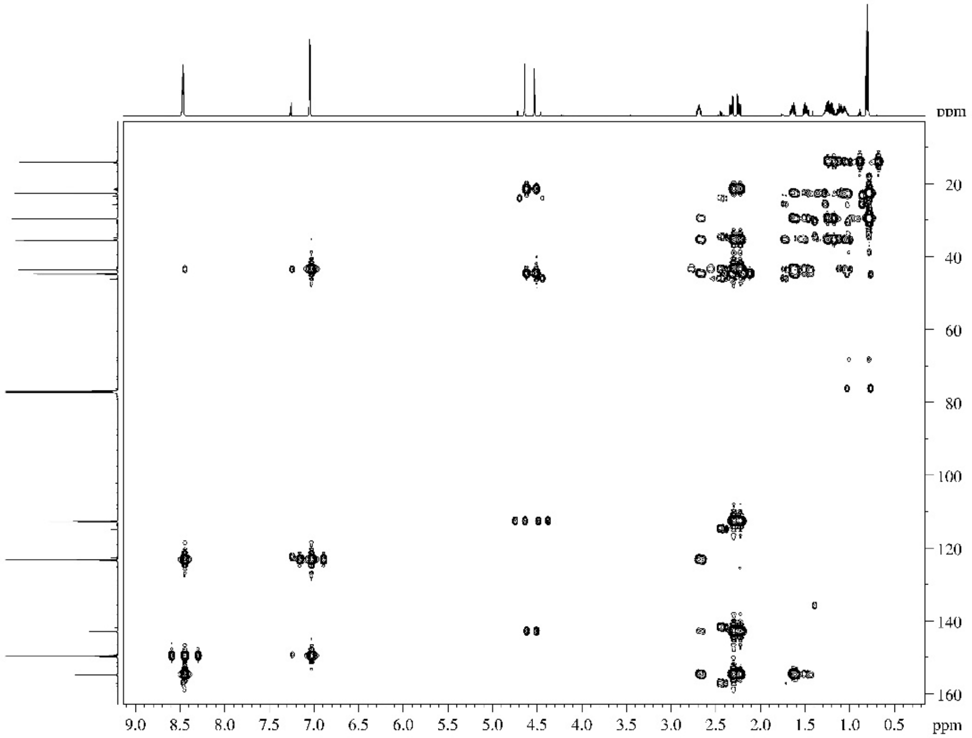

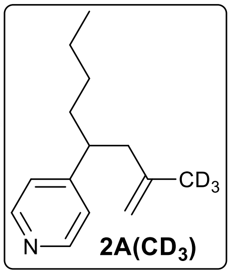

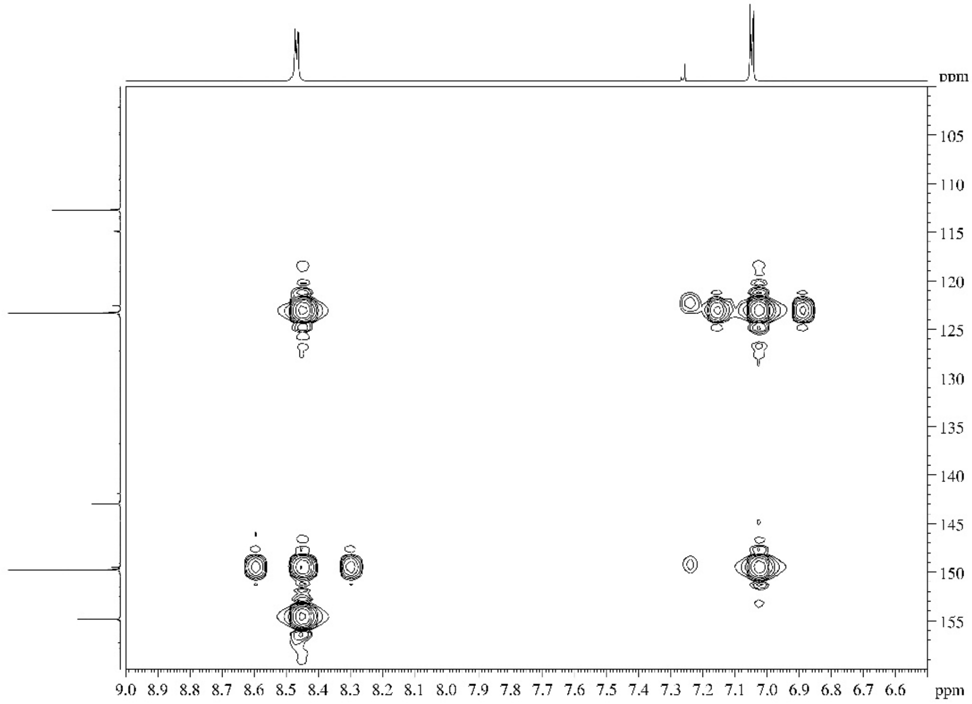

2D HMBC (600 MHz, $CDCl_3$) – NMR spectra of **2A (CD_3)**

The *$\underline{C}D_3$* peak at 21.8 ppm correlated to the allylic & vinylic protons as shown by HMBC.

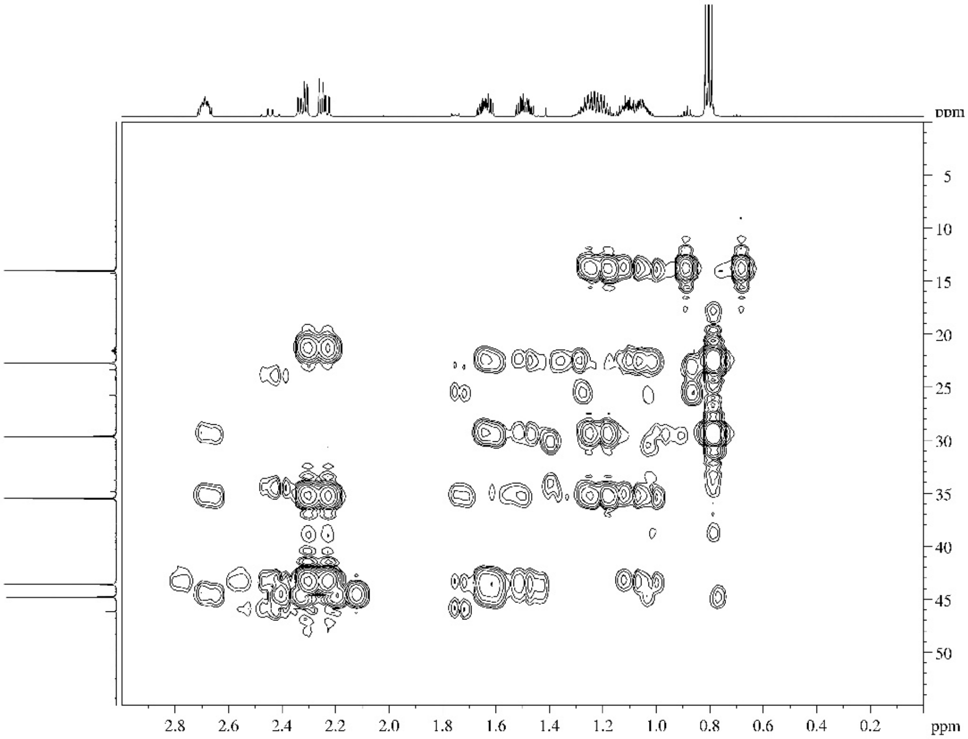

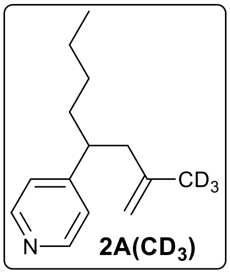

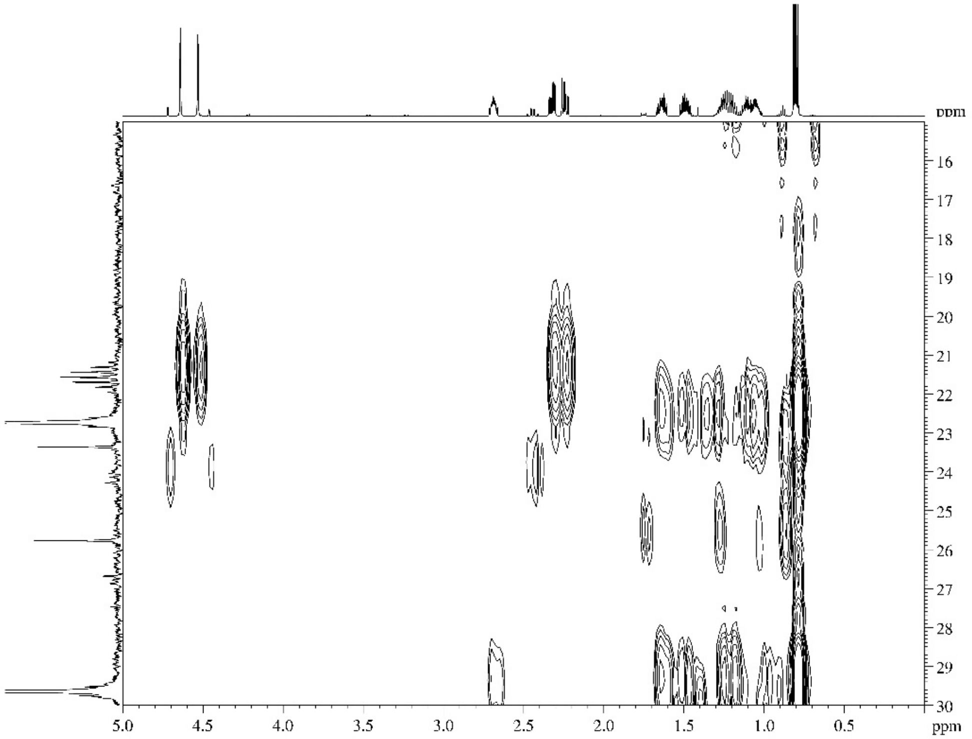

^{1}H (300 MHz, $CDCl_3$) – NMR spectra of the cross-over experiment (ADHP **1' (CH_3)** & **2' (CD_3)**)

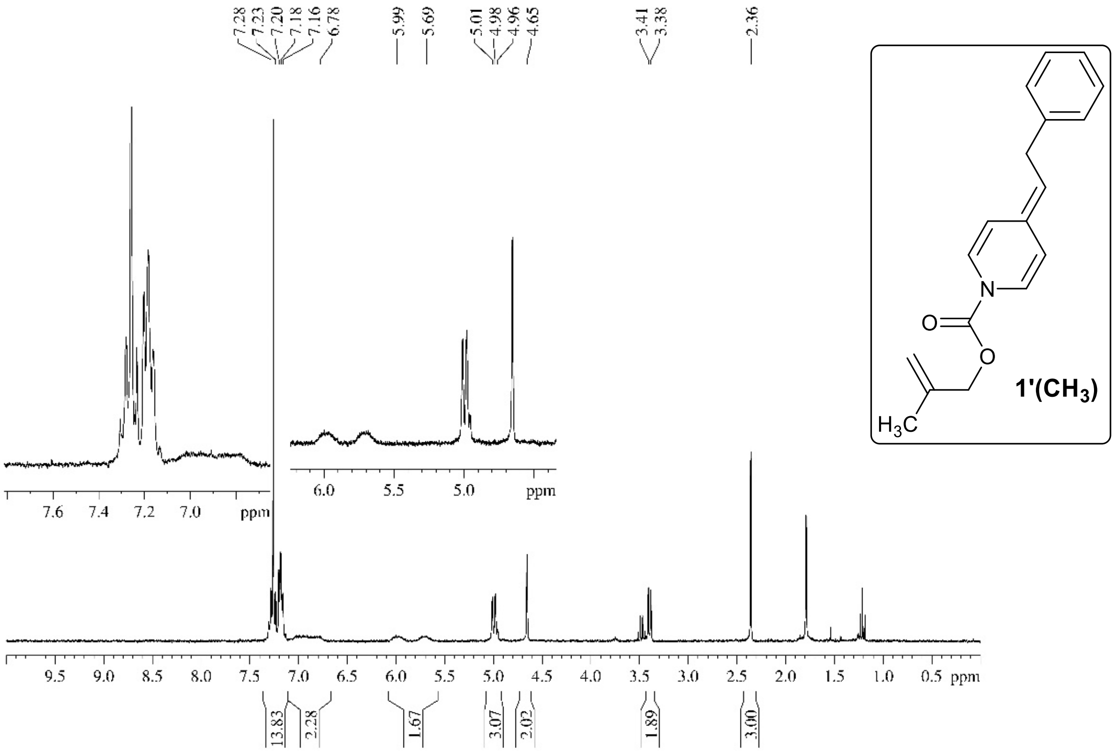

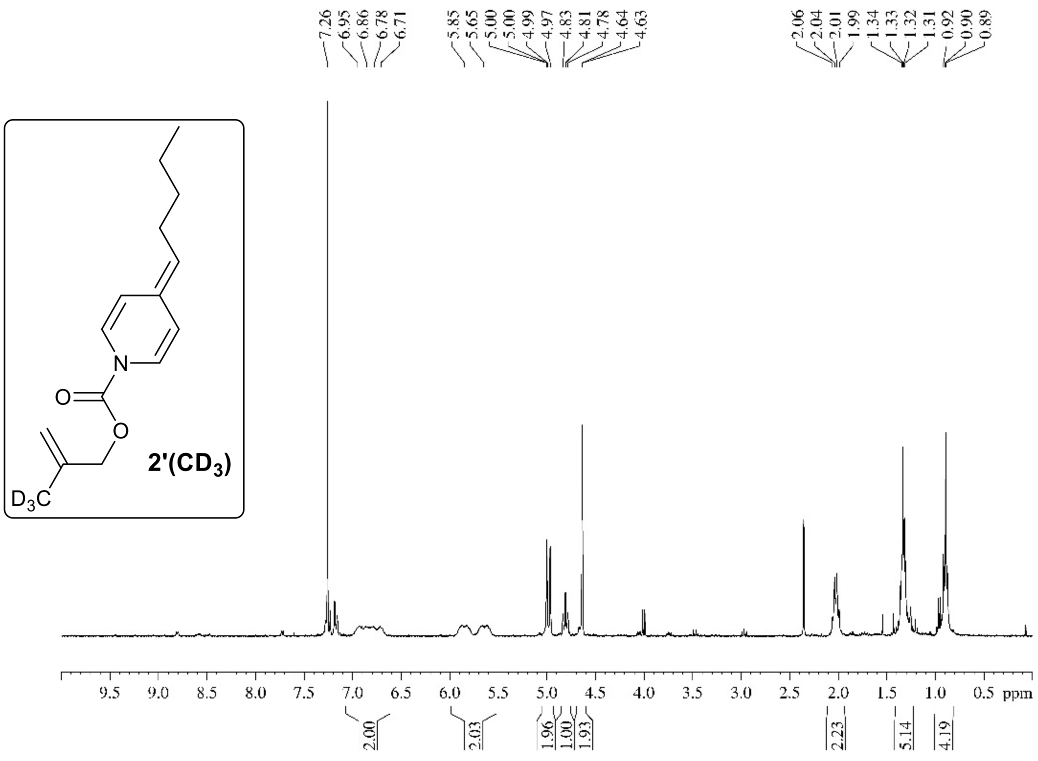

Residual diethyl ether signals

Residual toluene signals

^{1}H (400 MHz, $CDCl_3$) and ^{2}H (61 MHz, $CDCl_3$) – NMR spectra of the cross-over products **1A (CH_3) + 1A (CD_3)**

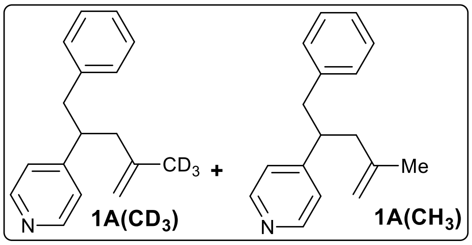

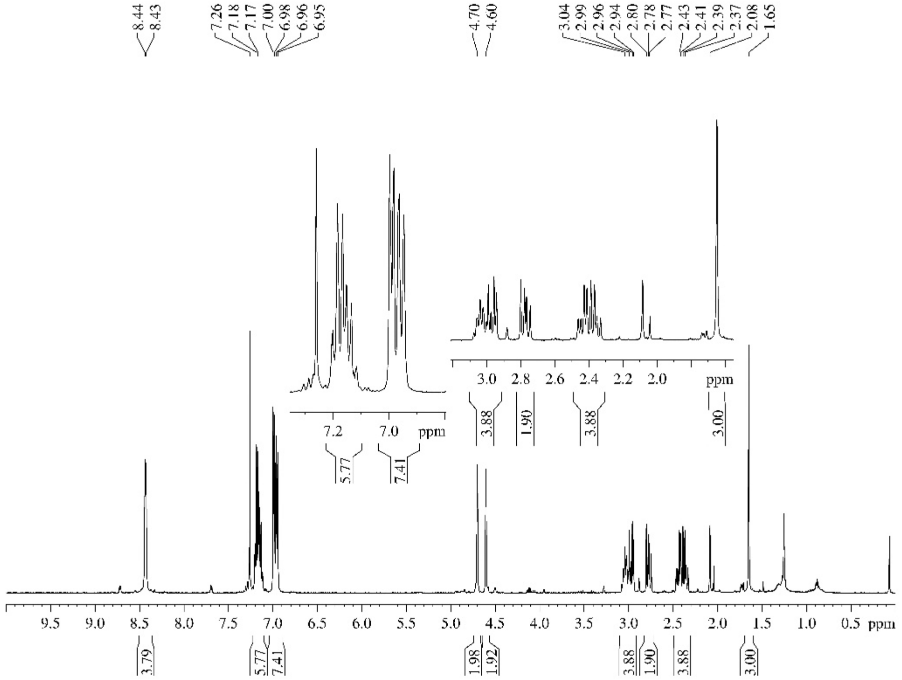

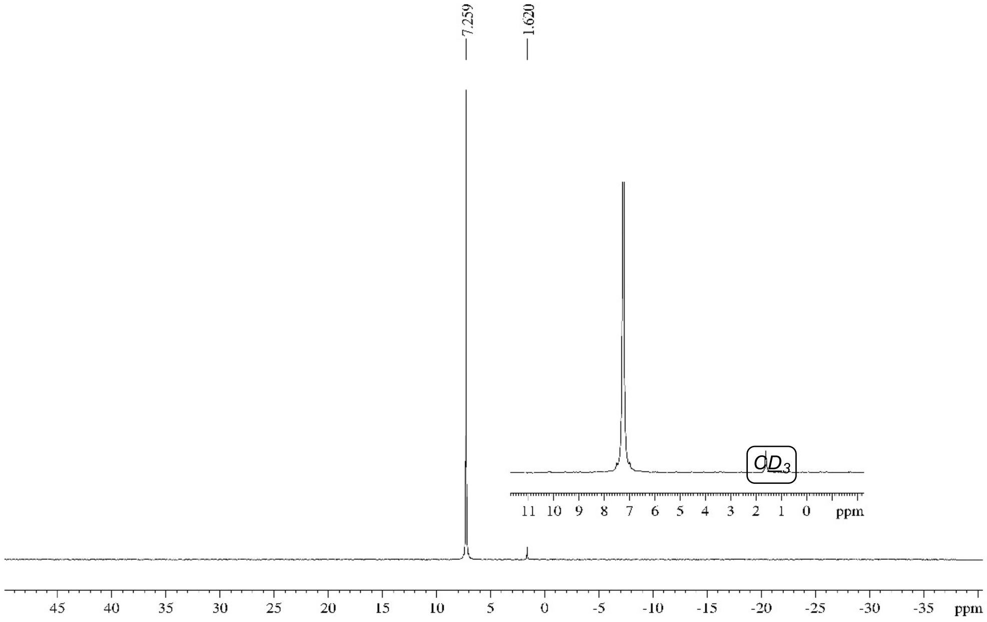

CH$_3$ peak integration used to determine product ratio.

^{1}H (400 MHz, $CDCl_3$) and ^{2}H (61 MHz, $CDCl_3$) – NMR spectra of the cross-over products **2A (CD_3) + 2A (CH_3)**

The relative amounts of **2A (CD_3)** was established. by integrating the *C<u>D</u>₃* peak at 1.62 ppm in ^{2}H NMR relative to a known amount of C<u>D</u>₃CN,

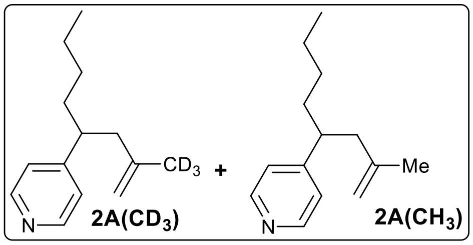

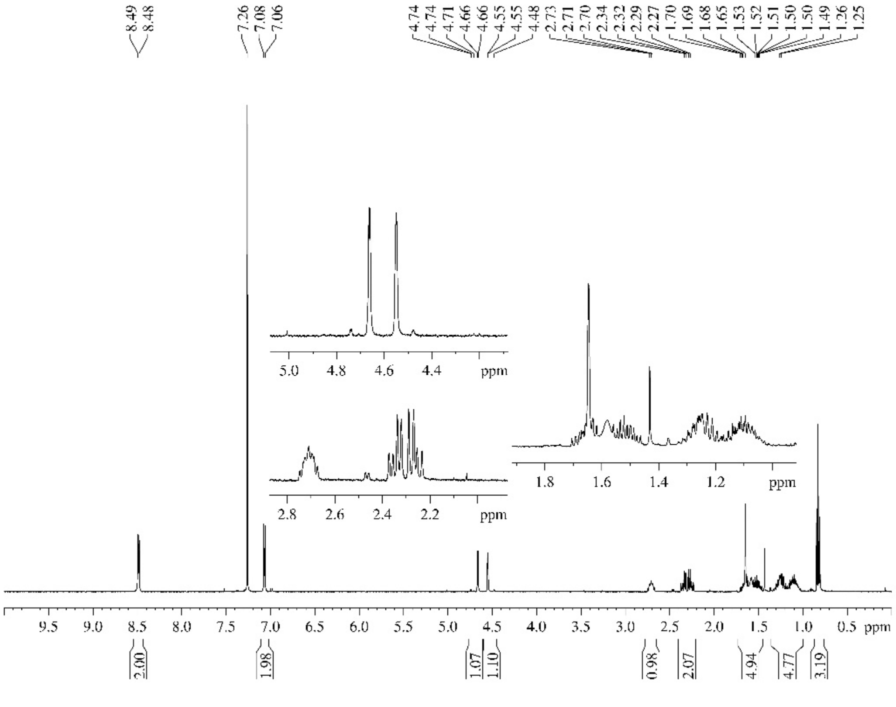

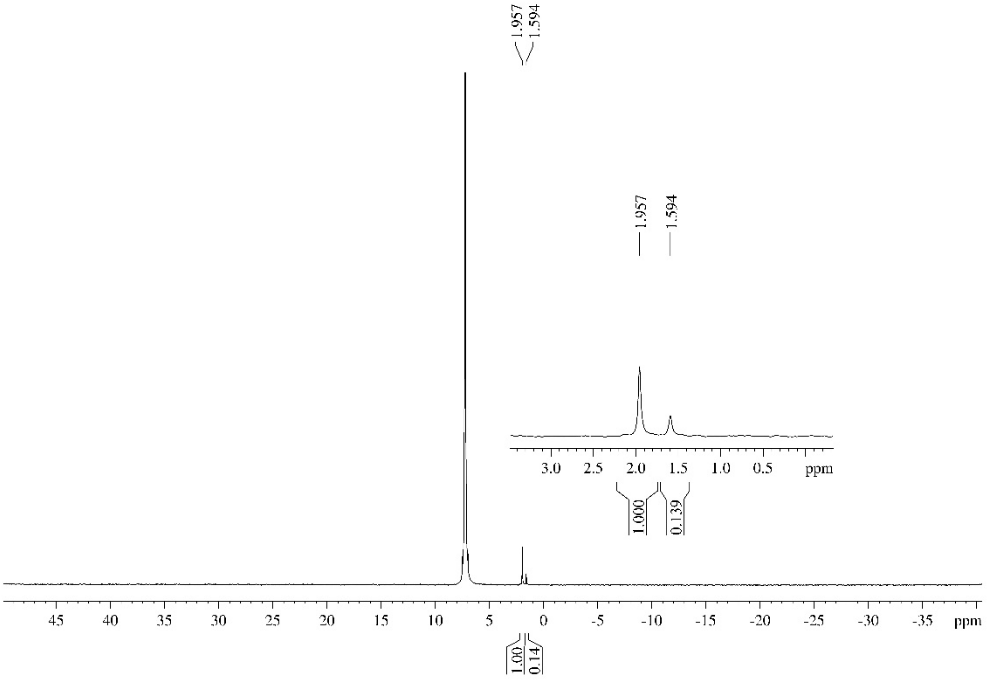

CD₃CN used as an internal standard.

^{1}H (600 MHz, $CDCl_3$) and ^{13}C (151 MHz, $CDCl_3$) – NMR spectra of **31A**

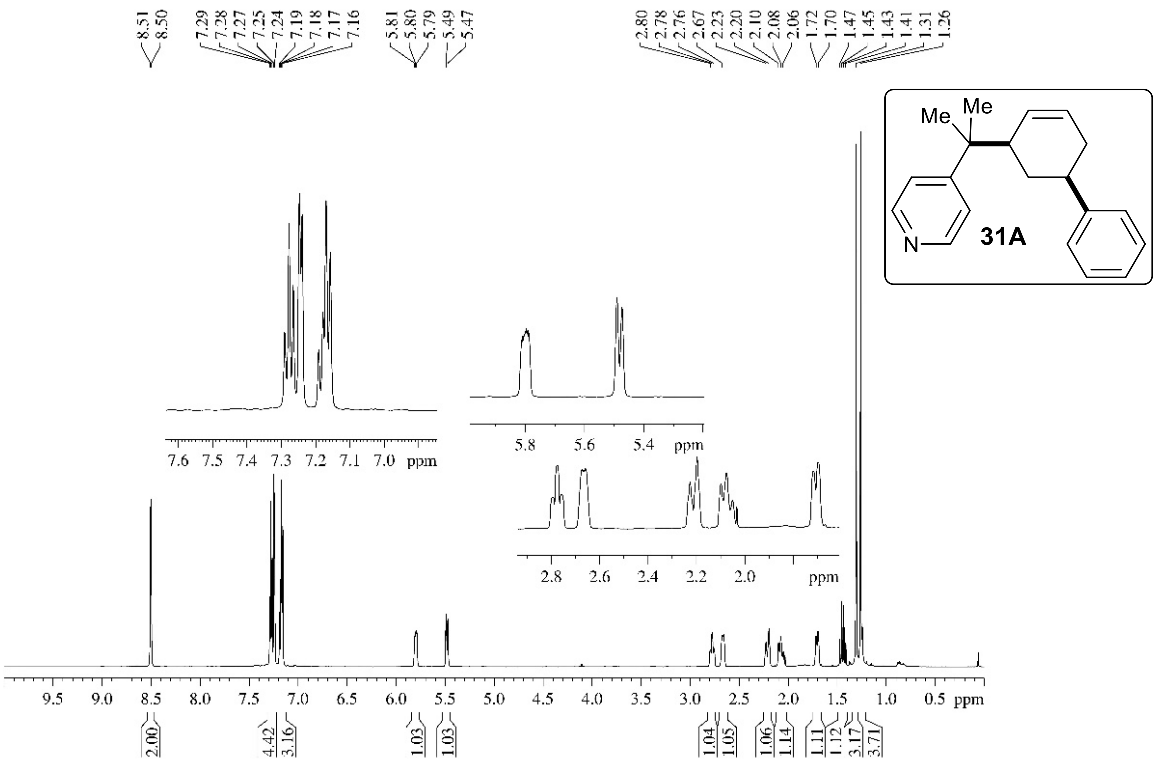

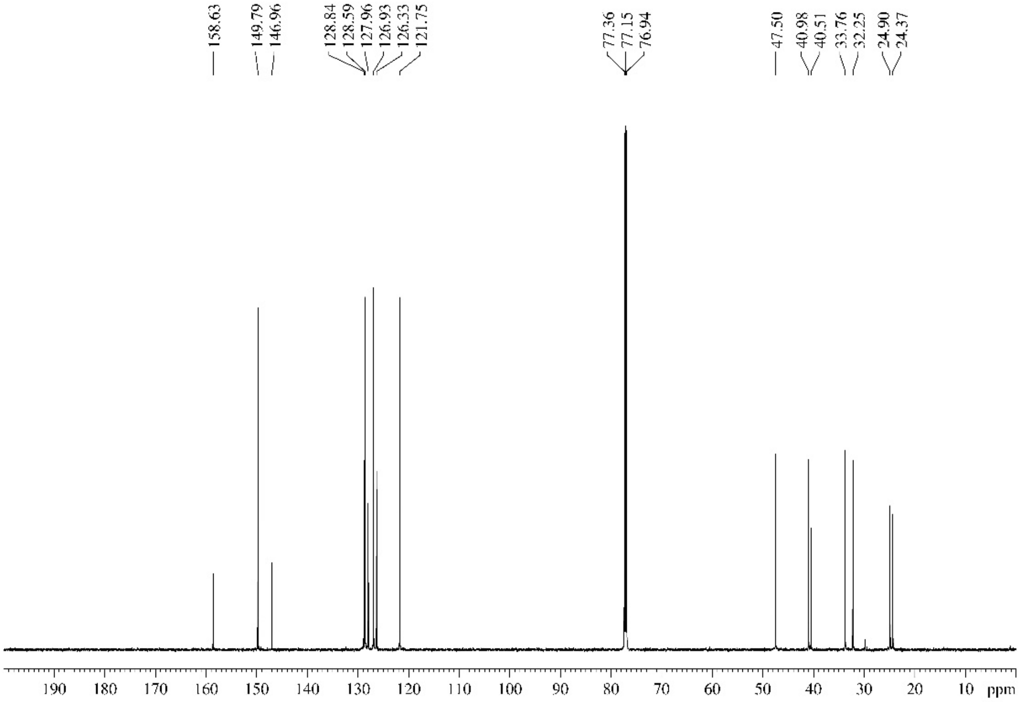

2D-COSY (600 MHz, $CDCl_3$) – NMR spectra of **31A**

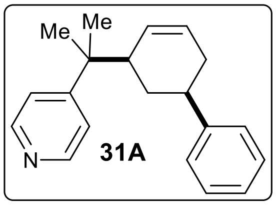

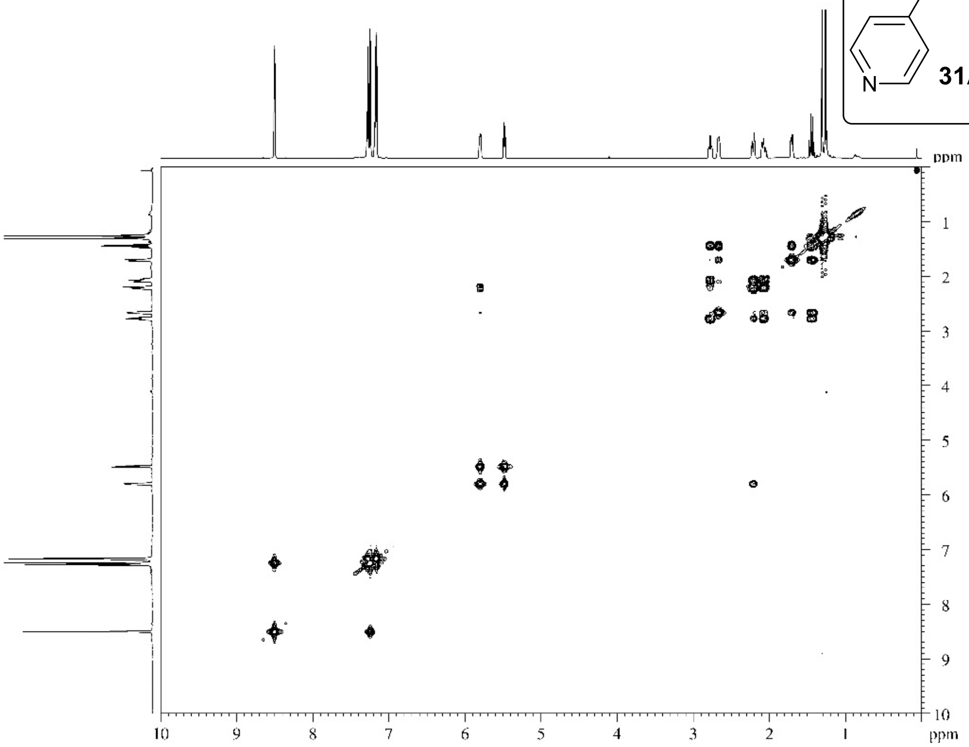

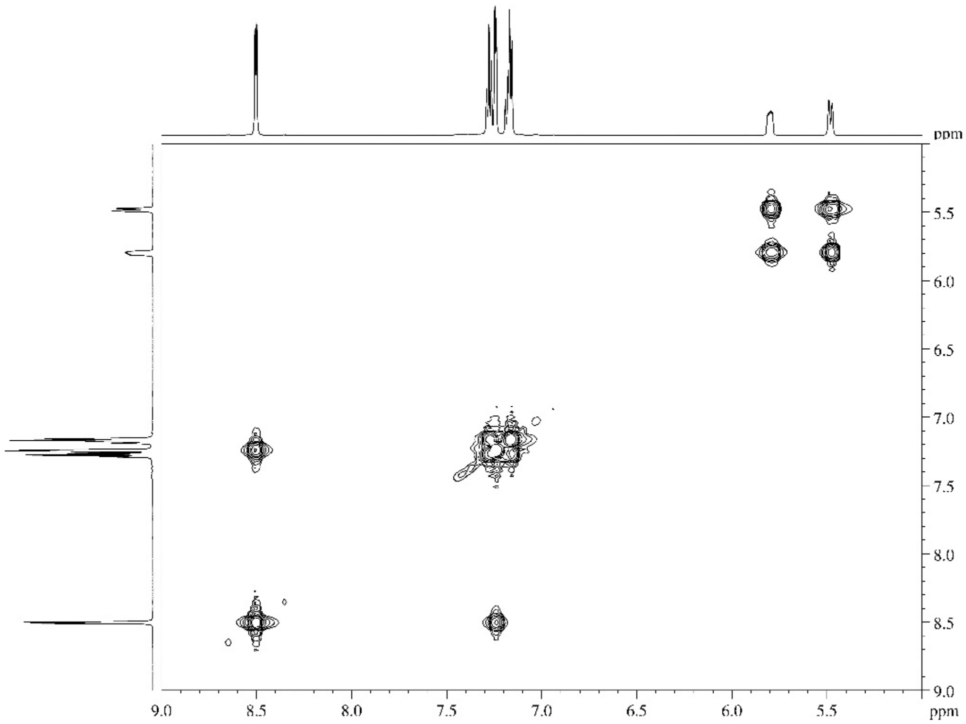

2D-COSY (600 MHz, $CDCl_3$) – NMR spectra of **31A**

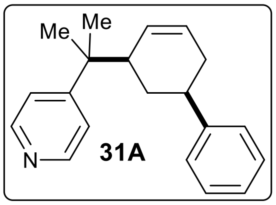

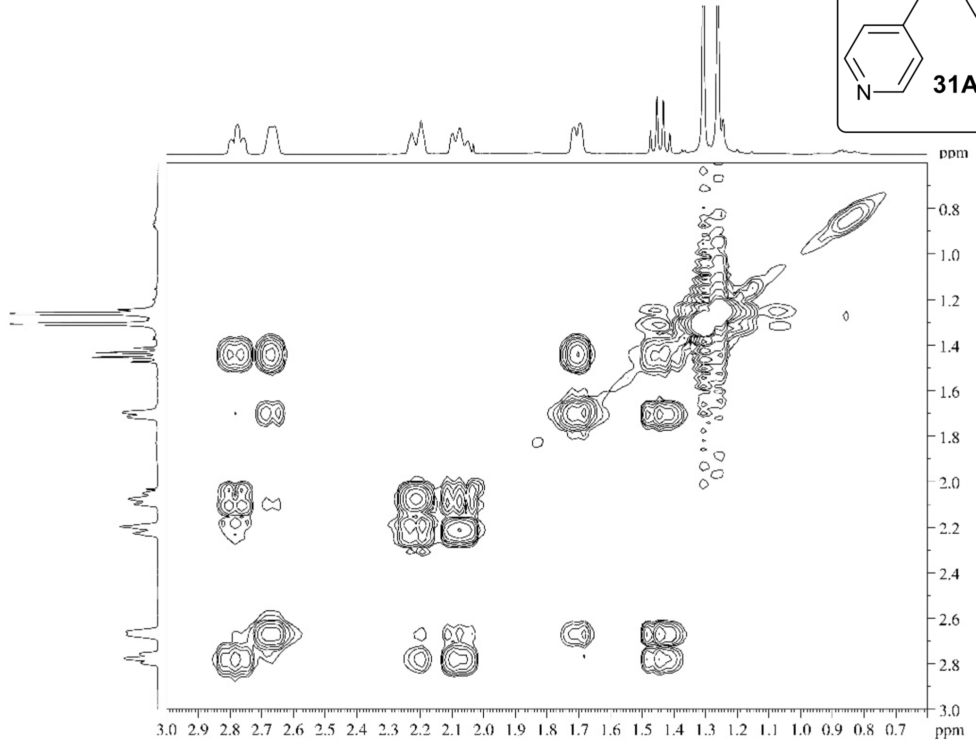

2D-HMBC (600 MHz, $CDCl_3$) – NMR spectra of **31A**

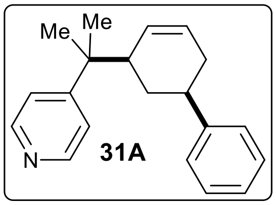

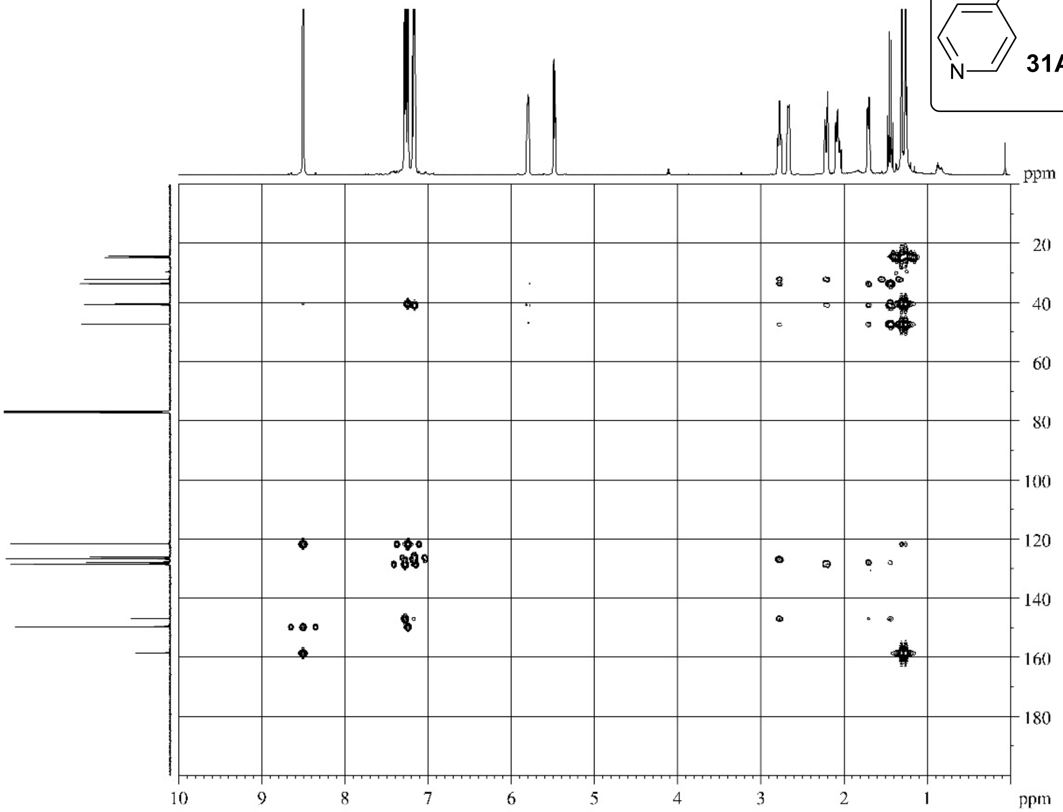

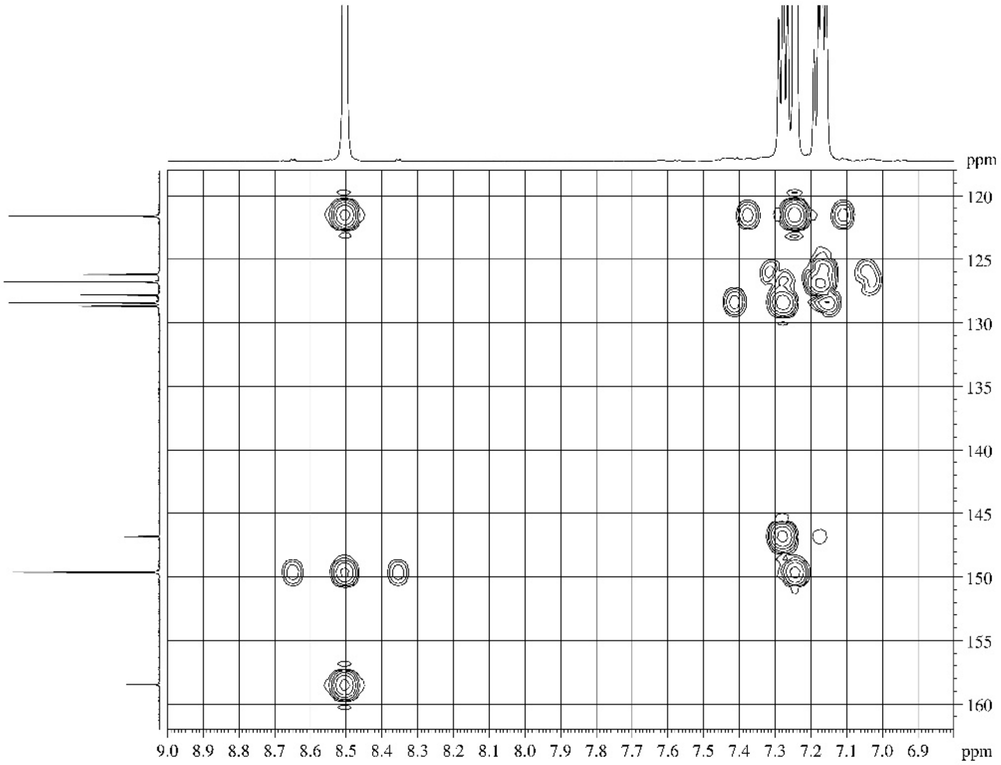

2D-HMBC (600 MHz, $CDCl_3$) – NMR spectra of **31A**

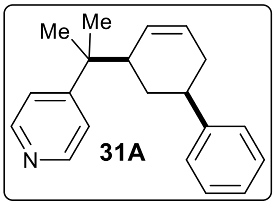

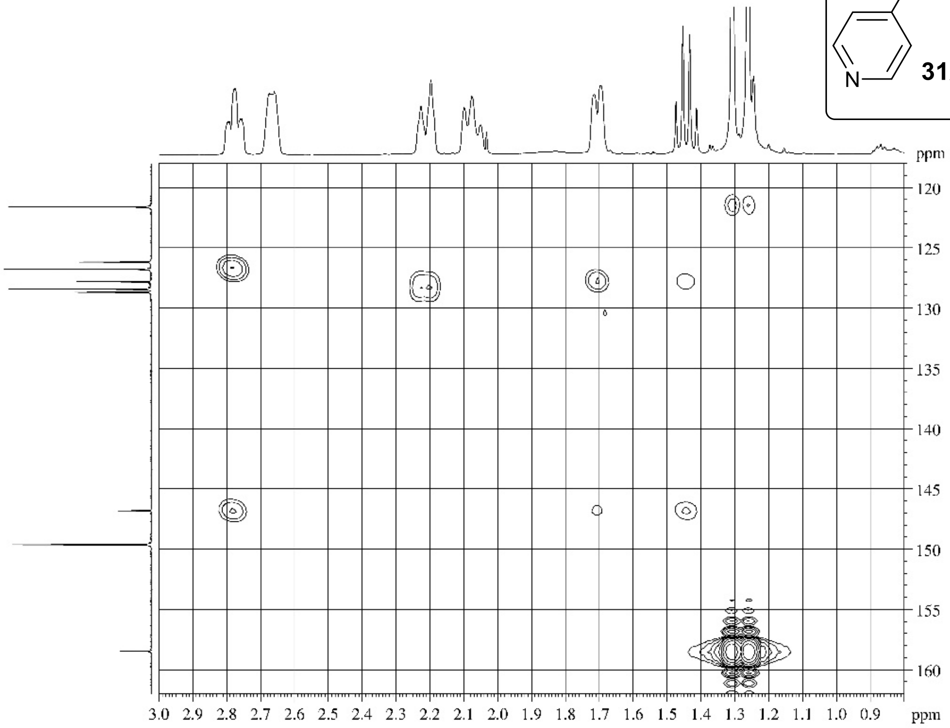

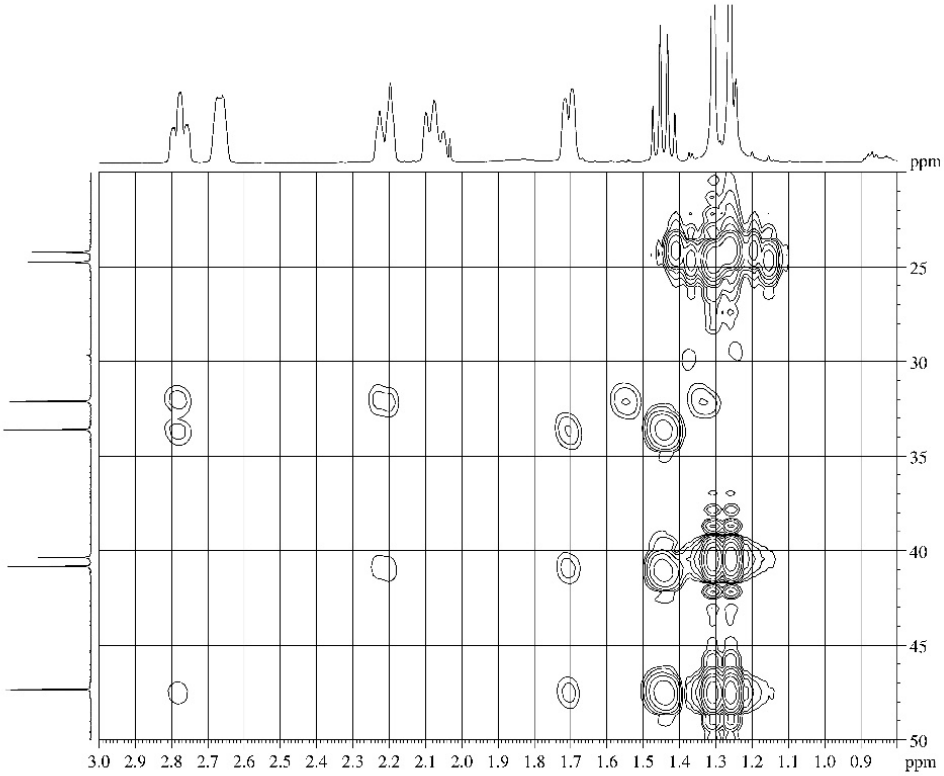

2D-NOESY (600 MHz, $CDCl_3$) – NMR spectra of **31A**

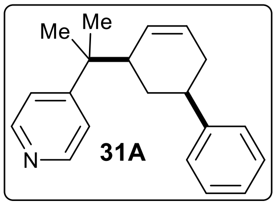

31A

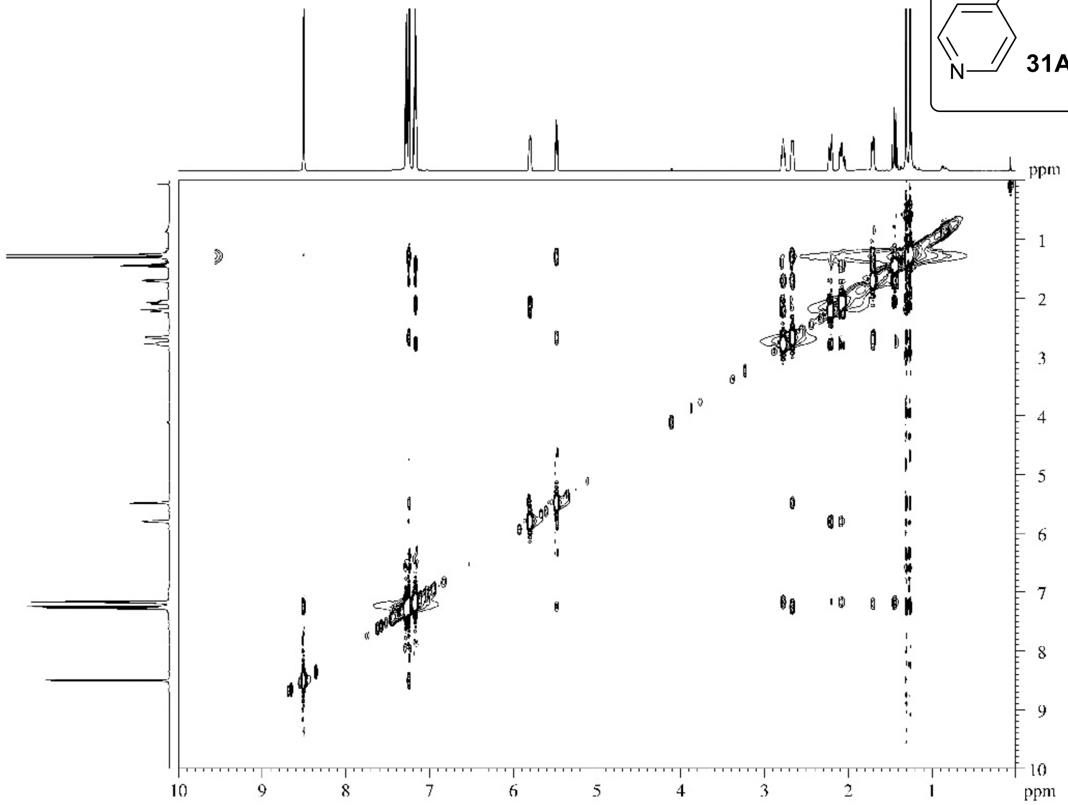

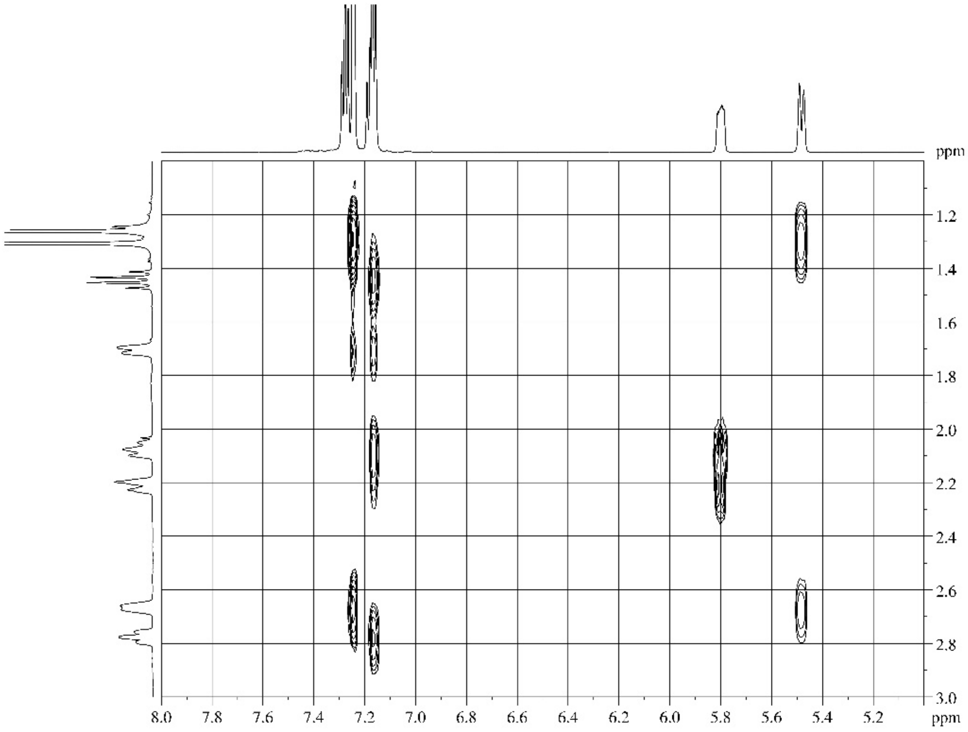

2D-NOESY (600 MHz, $CDCl_3$) – NMR spectra of **31A**

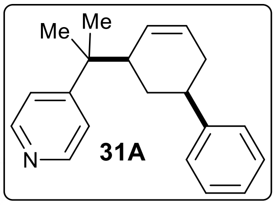

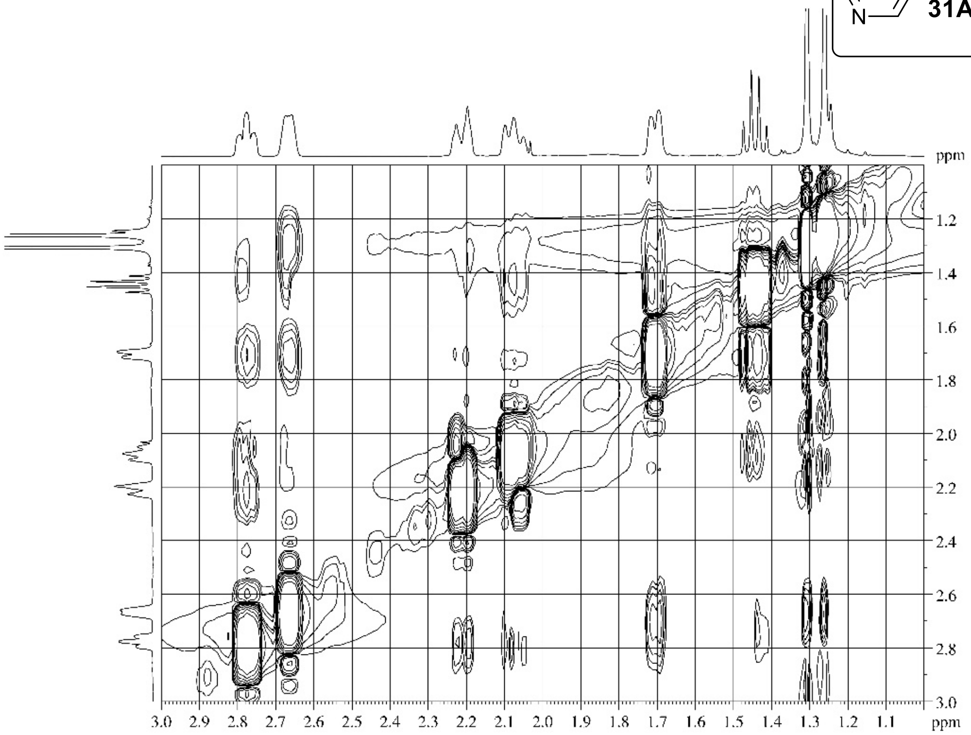

Computational Method.

The calculations were performed using the Gaussian 16[30] and CREST 2.10.2 software packages.[31,32] Prior to the DFT geometry optimization, conformational sampling of all stable species was performed using CREST with the default parameters. Due to the large number of conformers in some cases, up to 11 unique conformers were chosen as starting points for further DFT calculations. The geometry optimizations were performed with the ωB97XD density functional,[33] in combination with the 6-31+G(d,p) basis set[34] for all atoms except palladium, for which SDD[35] and corresponding ECP were used. Single-point calculations were performed with the M06 density functional[36], in combination with the 6-311++G(d,p) basis set[37] for all atoms except palladium, for which SDD and corresponding ECP were used. The D3 empirical dispersion was also applied for these calculations. The SMD implicit solvation model for tetrahydrofuran was used in all cases.[38] Substrate **1’** was selected for the study. Harmonic vibrational frequencies were computed for all optimized structures to verify that they were either minima or transition states, possessing zero or one imaginary frequency, respectively. All the free energies (SMD(THF)/M06-D3/6-311++G(d,p)+SDD(Pd)//SMD(THF)/ ωB97XD/6-31+G(d,p)+SDD(Pd)) are reported in kJ/mol, incorporate unscaled thermodynamic corrections based on the vibrational analyses and temperature of 298.15 K, using the correction to vibrational entropy reported by Grimme.[39] Additionally, the free energies at 1 atm concentration were corrected to a 1M standard state using a +7.91 kJ/mol correction. In the case of flexible species, with multiple conformers with similar free energies, the Boltzmann-averaged free energy was used for the calculation of relative energies.

Computational Study – Additional Details.

Alternative Forms of the Pi-Allyl Complex

To study inner-sphere mechanisms, we first calculated the free energies associated with pi-allyl complexes possessing a free binding site, to enable association of the pyridylic anion. We make the approximation that these forms will represent the barrier to formation of inner-sphere adducts (**Int-F** and **Int-G**, see next Scheme).

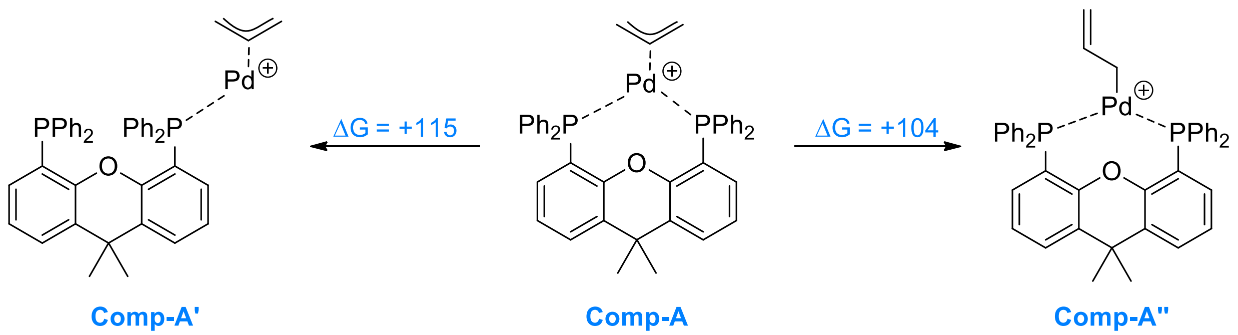

Both alternative forms of the pi-allyl complex are much higher in free energy. Using the previously mentioned approximation, **Int-F** and **Int-G** would never be formed, as the barrier leading to them is greater than the allylation barriers for both **Int-B** and **Int-C**. This fits well with the experimental observation that the reaction proceeds via an outer-sphere mechanism.

Extended Reaction Map

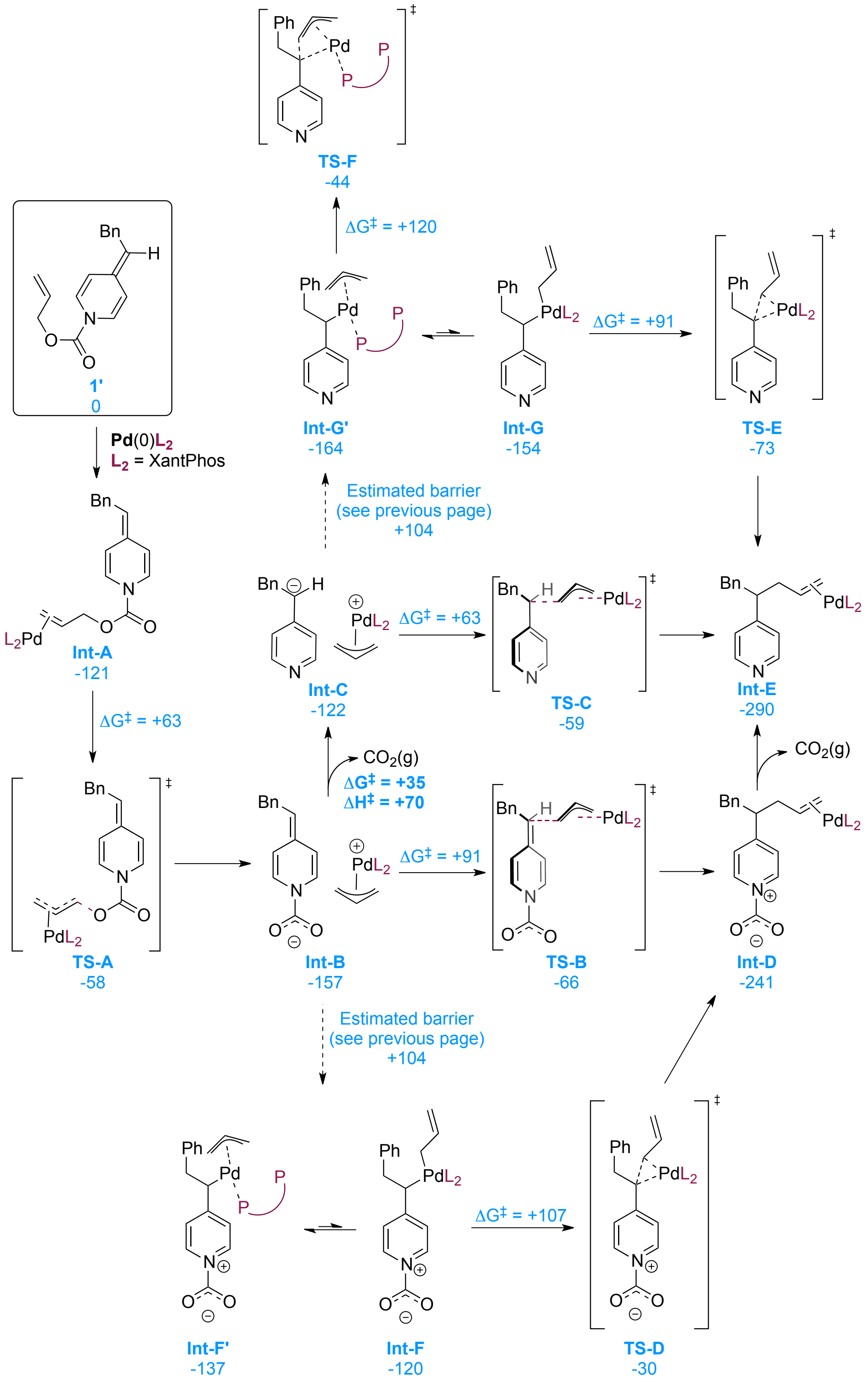

As previously discussed, **Int-F** will never form due to the large barrier. Moreover, the formation of **Int-F** is endergonic and its corresponding allylation barrier is even greater than for **Int-B**. This strongly suggests that no inner-sphere allylation can proceed here.

On the other hand, the formation of **Int-G** is exergonic and **TS-E** is actually lower in energy than **TS-C**. This entails that the reaction of **Int-C** via **TS-C** is faster than the formation of **Int-G**. This can be explained by the important barrier of a dissociative mechanism, once again.

Cartesian Coordinates of Computed Structures

1'_conf1
C 0.616432 1.102070 0.000657
C 1.882525 1.552522 0.146823
C 2.925557 1.009111 1.084095
C 3.993181 0.195828 0.371157
C 3.639870 -0.932847 -0.379500
C 4.611676 -1.693123 -1.025582
C 5.958018 -1.332374 -0.938700
C 6.320427 -0.208263 -0.199528
C 5.343044 0.548240 0.450505
H 5.633901 1.423831 1.026291
H 7.364468 0.083642 -0.127619
H 6.716230 -1.922600 -1.445159
H 4.318964 -2.567350 -1.600492
H 2.592718 -1.215279 -0.460274
H 3.411996 1.842611 1.603851
H 2.465568 0.388520 1.860677
H 2.203680 2.373425 -0.493572
C -0.308126 1.708321 -0.961367
C -1.567424 1.278382 -1.122382
N -2.082811 0.210329 -0.372443
C -1.240338 -0.414264 0.557736
C 0.026389 -0.012212 0.747968
H 0.615291 -0.552786 1.480222
H -1.678014 -1.236974 1.104302
C -3.396071 -0.180267 -0.591621
O -4.131200 0.363467 -1.391344
O -3.729217 -1.223499 0.174571
C -5.092441 -1.664257 0.114746
C -5.980346 -0.929495 1.072093
C -5.604379 0.042705 1.900131
H -4.577851 0.398076 1.949141
H -6.322714 0.518148 2.561269
H -7.017053 -1.262548 1.050401
H -5.465560 -1.568639 -0.908778
H -5.053329 -2.726758 0.369063
H -2.262465 1.714482 -1.826784
H 0.034028 2.538721 -1.570994
SCF: -862.984249
ZPE: 0.305440
H: -862.659932
G: -862.721237
SP SCF: -862.836241

1'_conf2
C -0.828010 -1.751515 -0.013372
C -2.118169 -2.153621 0.022682
C -3.236127 -1.528897 -0.776447
C -3.685951 -0.164201 -0.277949
C -3.942395 0.057116 1.080616
C -4.371819 1.302857 1.532976
C -4.549065 2.355086 0.632610
C -4.293795 2.147645 -0.721489
C -3.865368 0.896938 -1.169875
H -3.664032 0.744938 -2.228095
H -4.423290 2.959560 -1.431803
H -4.880255 3.327488 0.985378
H -4.566478 1.454222 2.591143
H -3.799338 -0.752946 1.791610
H -2.947716 -1.433466 -1.830726
H -4.097742 -2.206091 -0.759345
H -2.375868 -2.977001 0.685701
C 0.214707 -2.389206 0.791108
C 1.494396 -1.989878 0.756949
N 1.912877 -0.928196 -0.058618
C 0.955191 -0.278049 -0.847838
C -0.335526 -0.648322 -0.840859
H -1.021458 -0.091824 -1.469327
H 1.323596 0.537723 -1.452712
C 3.252216 -0.561671 -0.033504
O 4.080494 -1.104027 0.669973
O 3.498878 0.439770 -0.882972
C 4.816108 1.005711 -0.858684
C 4.953650 2.097889 0.158014
C 3.998138 2.527124 0.979798
H 2.997612 2.100910 0.980194
H 4.192148 3.328547 1.686354
H 5.944287 2.549742 0.183425
H 5.553137 0.217420 -0.682362
H 4.969124 1.400722 -1.866462
H 2.278846 -2.447240 1.344185
H -0.049763 -3.216572 1.442072
SCF: -862.983343
ZPE: 0.305535
H: -862.658996
G: -862.719762
SP SCF: -862.835493

1'_conf3
C 0.760932 1.140479 0.323570
C 2.043717 1.460082 0.606211
C 3.009792 0.660584 1.436206
C 4.073989 -0.031299 0.600664
C 3.707308 -0.925716 -0.412987
C 4.674644 -1.577719 -1.173962
C 6.030576 -1.340051 -0.938974
C 6.406800 -0.447913 0.063024
C 5.433471 0.200113 0.826383
H 5.735318 0.894142 1.607385
H 7.458706 -0.252904 0.252506
H 6.785707 -1.845251 -1.534213
H 4.370986 -2.270309 -1.954049
H 2.653259 -1.109252 -0.608254
H 3.507142 1.326421 2.151071
H 2.483135 -0.092100 2.032620
H 2.442765 2.375683 0.170590
C -0.076329 2.002820 -0.515337
C -1.348887 1.705800 -0.813919
N -1.964544 0.539103 -0.336474
C -1.210260 -0.326242 0.467583
C 0.066714 -0.063786 0.788742
H 0.580750 -0.788920 1.409219
H -1.724058 -1.214156 0.806330
C -3.282853 0.290303 -0.692163
O -3.934547 1.032283 -1.402225
O -3.727279 -0.850839 -0.163771
C -5.096239 -1.218692 -0.460222
C -6.056959 -0.514865 0.447673
C -6.881115 -1.158857 1.273186
H -6.880634 -2.244901 1.345459
H -7.589525 -0.621103 1.897254
H -6.073770 0.571780 0.386461
H -5.297845 -0.990763 -1.510158
H -5.123873 -2.298994 -0.309948
H -1.979045 2.331091 -1.431335
H 0.345057 2.920075 -0.914589
SCF: -862.983840
ZPE: 0.305417
H: -862.659423
G: -862.720975
SP SCF: -862.834952

1'_conf4
C 0.735035 -1.129750 -0.079467
C 2.006069 -1.549189 -0.269578
C 3.003484 -0.982311 -1.242040
C 4.085938 -0.159645 -0.563210
C 5.436628 -0.492470 -0.695532
C 6.426996 0.272255 -0.075441
C 6.076834 1.384932 0.686584
C 4.729820 1.726221 0.826170
C 3.745284 0.957889 0.209600
H 2.697959 1.224817 0.331053
H 4.446701 2.591477 1.419177
H 6.845102 1.981354 1.170123
H 7.471583 -0.004336 -0.188445
H 5.717811 -1.359222 -1.289240
H 3.481672 -1.803878 -1.787987
H 2.505145 -0.361837 -1.994695
H 2.368917 -2.361780 0.359125
C -0.140405 -1.757554 0.914235
C -1.405722 -1.362724 1.114852
N -1.976077 -0.313529 0.378954
C -1.180370 0.334081 -0.575391
C 0.090979 -0.031526 -0.805466
H 0.640699 0.526402 -1.554864
H -1.656725 1.144479 -1.107574
C -3.295351 0.038090 0.635837
O -3.986462 -0.525634 1.461154
O -3.683583 1.066604 -0.121382
C -5.073963 1.457582 -0.027956
C -5.919311 0.635161 -0.951486
C -6.982780 -0.059115 -0.549945
H -7.279362 -0.088413 0.496419
H -7.591877 -0.617846 -1.255237
H -5.631324 0.648458 -2.001894
H -5.407882 1.379776 1.008560
H -5.066663 2.506904 -0.330731
H -2.064001 -1.816637 1.842851
H 0.244664 -2.575582 1.514945
SCF: -862.983704
ZPE: 0.305381
H: -862.659355
G: -862.720868
SP SCF: -862.835076

1'_conf5
C -0.676018 -1.407392 -0.054923
C -1.994146 -1.707588 -0.058661
C -3.020648 -1.260801 0.945718
C -3.929287 -0.162983 0.417277
C -5.317178 -0.323591 0.394071
C -6.148623 0.693732 -0.078819
C -5.599523 1.890147 -0.535378
C -4.213649 2.060847 -0.518126
C -3.388065 1.041617 -0.049594
H -2.309341 1.179641 -0.047577
H -3.775693 2.989601 -0.873183
H -6.243749 2.683267 -0.903716
H -7.225209 0.548050 -0.090617
H -5.753577 -1.254105 0.749708
H -3.640971 -2.118314 1.231202
H -2.540399 -0.909843 1.865566
H -2.372088 -2.310478 -0.883794
C 0.225823 -1.894715 -1.102413
C 1.535702 -1.610425 -1.118391
N 2.130120 -0.820308 -0.122822
C 1.313193 -0.316230 0.898743
C -0.002339 -0.579195 0.949147
H -0.565016 -0.142907 1.766396
H 1.811649 0.298075 1.634420
C 3.490931 -0.561749 -0.204683
O 4.197296 -0.972150 -1.103734
O 3.909769 0.174388 0.829466
C 5.271621 0.621984 0.801447
C 5.434059 1.923153 0.076129
C 4.461083 2.620947 -0.506021
H 3.425932 2.287777 -0.501442
H 4.675190 3.558019 -1.011257
H 6.460199 2.287455 0.054816
H 5.551611 0.732605 1.852472
H 5.904507 -0.150611 0.355955
H 2.213809 -1.961948 -1.883924
H -0.176484 -2.510871 -1.900404
SCF: -862.984261
ZPE: 0.305442
H: -862.659985
G: -862.720849
SP SCF: -862.836187

1'_conf6
C -0.781183 -1.356236 -0.087883
C -2.098673 -1.657762 -0.122739
C -3.141730 -1.247858 0.880162
C -4.048875 -0.137291 0.376089
C -3.506565 1.076374 -0.065209
C -4.331384 2.107280 -0.509061
C -5.717582 1.939657 -0.526681
C -6.267855 0.734387 -0.095381
C -5.437215 -0.294669 0.352740
H -5.874582 -1.232118 0.688451
H -7.344734 0.590905 -0.107977
H -6.361157 2.741854 -0.876024
H -3.892452 3.042748 -0.844780
H -2.427590 1.212230 -0.063030
H -3.761919 -2.116765 1.129324
H -2.676605 -0.924836 1.817938
H -2.462202 -2.232763 -0.973912
C 0.137879 -1.807638 -1.136426
C 1.448282 -1.525045 -1.119931
N 2.026488 -0.770561 -0.088573
C 1.192838 -0.299936 0.934504
C -0.123911 -0.562322 0.954131
H -0.699845 -0.152770 1.776000
H 1.679211 0.288259 1.699044
C 3.392253 -0.519905 -0.133610
O 4.112603 -0.912506 -1.029902
O 3.795819 0.180888 0.928352
C 5.182993 0.593101 0.948243
C 5.370495 1.870546 0.188997
C 6.249550 2.011953 -0.801635
H 6.865271 1.179916 -1.136904
H 6.383454 2.964656 -1.306632
H 4.756324 2.711493 0.508626
H 5.393722 0.732700 2.010848
H 5.809425 -0.210128 0.555227
H 2.138801 -1.851740 -1.885306
H -0.251211 -2.395372 -1.961943
SCF: -862.983695
ZPE: 0.305419
H: -862.659325
G: -862.720591
SP SCF: -862.835029

1'_conf7
C 0.650930 -1.133883 0.139991
C 1.915951 -1.583991 -0.016349
C 2.897397 -1.151644 -1.070276
C 3.983001 -0.232673 -0.533920
C 5.335926 -0.533392 -0.713911
C 6.330069 0.322638 -0.235793
C 5.981865 1.495704 0.430753
C 4.633116 1.805224 0.617705
C 3.644828 0.946185 0.142610
H 2.596964 1.190204 0.300969
H 4.351282 2.716457 1.137974
H 6.753053 2.163091 0.804425
H 7.376287 0.069656 -0.383793
H 5.615952 -1.446801 -1.233613
H 3.375058 -2.038566 -1.502488
H 2.383888 -0.647448 -1.896336
H 2.284972 -2.314358 0.702985
C -0.212716 -1.626258 1.216756
C -1.472514 -1.200042 1.384176
N -2.046459 -0.244644 0.531832
C -1.260932 0.274406 -0.506750
C 0.003997 -0.128167 -0.707400
H 0.547292 0.328391 -1.526856
H -1.740671 1.016818 -1.127977
C -3.359757 0.142035 0.758030
O -4.048364 -0.316680 1.647498
O -3.748520 1.078346 -0.112975
C -5.124254 1.480152 -0.068424
C -6.002011 0.620575 -0.926301
C -5.605178 -0.420647 -1.654912
H -4.567473 -0.744312 -1.686197
H -6.316624 -0.987149 -2.248283
H -7.049224 0.919613 -0.919253
H -5.475320 1.479494 0.967269
H -5.126491 2.511074 -0.432170
H -2.123683 -1.554882 2.171240
H 0.176490 -2.366119 1.909178
SCF: -862.984248
ZPE: 0.305413
H: -862.659957
G: -862.721040
SP SCF: -862.836219

1'_conf8
C 0.760945 1.140528 0.323628
C 2.043730 1.460120 0.606279
C 3.009805 0.660590 1.436243
C 4.073974 -0.031303 0.600673
C 5.433464 0.200094 0.826360
C 6.406767 -0.447942 0.062976
C 6.030509 -1.340076 -0.939013
C 4.674569 -1.577729 -1.173967
C 3.707259 -0.925716 -0.412968
H 2.653202 -1.109241 -0.608210
H 4.370883 -2.270315 -1.954047
H 6.785619 -1.845285 -1.534270
H 7.458680 -0.252945 0.252433
H 5.735338 0.894119 1.607355
H 3.507179 1.326405 2.151110
H 2.483143 -0.092094 2.032655
H 2.442777 2.375738 0.170693
C -0.076321 2.002901 -0.515240
C -1.348877 1.705889 -0.813834
N -1.964529 0.539167 -0.336441
C -1.210240 -0.326208 0.467581
C 0.066732 -0.063758 0.788752
H 0.580770 -0.788915 1.409200
H -1.724035 -1.214138 0.806291
C -3.282834 0.290372 -0.692146
O -3.934539 1.032390 -1.402159
O -3.727242 -0.850814 -0.163833
C -5.096195 -1.218669 -0.460308
C -6.056918 -0.514979 0.447688
C -6.881057 -1.159094 1.273121
H -6.880558 -2.245148 1.345248
H -7.589471 -0.621437 1.897268
H -6.073747 0.571674 0.386622
H -5.297823 -0.990618 -1.510213
H -5.123798 -2.298990 -0.310166
H -1.979039 2.331204 -1.431222
H 0.345061 2.920175 -0.914453
SCF: -862.983840
ZPE: 0.305417
H: -862.659423
G: -862.720978
SP SCF: -862.834952

1'_conf9
C -0.650839 -1.133461 0.140189
C -1.915847 -1.583608 -0.016139
C -2.897266 -1.151437 -1.070162
C -3.983070 -0.232654 -0.533894
C -3.645148 0.946298 0.142599
C -4.633619 1.805160 0.617632
C -5.982302 1.495364 0.430654
C -6.330256 0.322204 -0.235856
C -5.335930 -0.533648 -0.713912
H -5.615759 -1.447134 -1.233586
H -7.376419 0.069010 -0.383877
H -6.753634 2.162613 0.804279
H -4.351981 2.716469 1.137874
H -2.597335 1.190529 0.300975
H -2.383771 -0.647159 -1.896179
H -3.374739 -2.038444 -1.502410
H -2.284881 -2.313888 0.703279
C 0.212767 -1.625684 1.217056
C 1.472585 -1.199505 1.384414
N 2.046591 -0.244291 0.531905
C 1.261076 0.274677 -0.506726
C -0.003876 -0.127858 -0.707313
H -0.547161 0.328641 -1.526809
H 1.740838 1.017003 -1.128038
C 3.359954 0.142246 0.757961
O 4.048535 -0.316382 1.647495
O 3.748825 1.078310 -0.113263
C 5.124656 1.479803 -0.068933
C 6.002144 0.619756 -0.926617
C 5.605002 -0.421581 -1.654894
H 4.567215 -0.745001 -1.686014
H 6.316265 -0.988432 -2.248150
H 7.049431 0.918536 -0.919730
H 5.127114 2.510614 -0.432990
H 5.475799 1.479375 0.966734
H 2.123730 -1.554245 2.171544
H -0.176485 -2.365406 1.909602
SCF: -862.984248
ZPE: 0.305412
H: -862.659957
G: -862.721044
SP SCF: -862.836220

1A_conf1
C 1.820743 -0.306326 0.103685
C 2.456393 -0.662111 -1.090181
C 3.797914 -1.026023 -1.067663
N 4.540642 -1.058290 0.046906
C 3.932237 -0.716863 1.188094
C 2.594012 -0.339442 1.264858
H 2.162800 -0.069845 2.224712
H 4.543887 -0.743899 2.086905
H 4.303261 -1.304688 -1.989360
H 1.920118 -0.656347 -2.034589
C 0.362008 0.097756 0.140062
H 0.107142 0.321541 1.183224
C -0.531718 -1.075808 -0.326459
C -2.005872 -0.815078 -0.126676
C -2.561471 -0.854685 1.158044
C -3.913876 -0.587814 1.361411
C -4.736958 -0.276829 0.277907
C -4.196461 -0.237662 -1.006443
C -2.841282 -0.504885 -1.204223
H -2.426113 -0.470248 -2.208713
H -4.828869 0.001206 -1.856875
H -5.791692 -0.070140 0.434346
H -4.326888 -0.626155 2.365495
H -1.926962 -1.100380 2.006826
H -0.241766 -1.971660 0.234405
H -0.332962 -1.280789 -1.384644
C 0.099848 1.373473 -0.688574
C 0.907415 2.552799 -0.226698
C 0.390153 3.657309 0.314122
H 1.022161 4.480404 0.637757
H -0.684088 3.774488 0.448683
H 1.989384 2.476990 -0.340838
H -0.966564 1.615616 -0.630656
H 0.326707 1.160820 -1.742547
SCF: -674.504854
ZPE: 0.290973
H: -674.197911
G: -674.252208
SP SCF: -674.328739

1A_conf2
C -0.926505 0.539659 0.023619
C -1.222810 1.411579 -1.028438
C -1.158679 2.783814 -0.816975
N -0.822210 3.340241 0.354430
C -0.537906 2.505191 1.359686
C -0.577212 1.118092 1.243652
H -0.329036 0.496747 2.099138
H -0.263483 2.965985 2.305709
H -1.386677 3.471927 -1.627619
H -1.496860 1.035680 -2.009898
C -0.941822 -0.963695 -0.158733
H -0.768960 -1.417375 0.824843
C 0.206642 -1.413015 -1.092678
C 1.568615 -1.025786 -0.569755
C 2.240451 0.097308 -1.062472
C 3.473724 0.481582 -0.536102
C 4.054531 -0.254890 0.495441
C 3.394963 -1.379162 0.993922
C 2.162895 -1.758560 0.464333
H 1.654011 -2.635918 0.857762
H 3.841789 -1.961887 1.794599
H 5.014696 0.042930 0.906631
H 3.979078 1.358192 -0.931483
H 1.790303 0.680123 -1.862385
H 0.050843 -0.979884 -2.087474
H 0.146774 -2.502046 -1.200254
C -2.295665 -1.484238 -0.684015
C -3.447607 -1.148802 0.219583
C -4.184978 -2.048596 0.872741
H -5.005028 -1.748045 1.519839
H -3.994284 -3.116612 0.778651
H -3.673481 -0.089169 0.344160
H -2.224359 -2.571478 -0.803304
H -2.477005 -1.062819 -1.681975
SCF: -674.506228
ZPE: 0.290929
H: -674.199342
G: -674.253709
SP SCF: -674.330887

1A_conf3
C -1.409370 -0.858263 0.072784
C -2.528818 -0.902015 0.909075
C -3.702866 -0.265590 0.523063
N -3.837353 0.403380 -0.629531
C -2.767567 0.442047 -1.431804
C -1.550502 -0.162757 -1.130071
H -0.728325 -0.072926 -1.832047
H -2.885380 0.988803 -2.364423
H -4.578404 -0.295091 1.167450
H -2.488092 -1.428024 1.859105
C -0.108850 -1.496065 0.514262
H -0.375872 -2.359169 1.137503
C 0.681289 -0.535093 1.448765
C 1.085356 0.773878 0.814154
C 2.309654 0.901929 0.148109
C 2.668522 2.099282 -0.470416
C 1.805533 3.194155 -0.429195
C 0.586871 3.083595 0.240550
C 0.233256 1.884201 0.856452
H -0.719320 1.806233 1.374951
H -0.089883 3.932309 0.284427
H 2.082950 4.128028 -0.909555
H 3.624255 2.177761 -0.981087
H 2.989049 0.053829 0.118537
H 1.574068 -1.066675 1.795382
H 0.061848 -0.336549 2.329895
C 0.736393 -2.023273 -0.659165
C 1.859892 -2.912070 -0.206731
C 3.137664 -2.778727 -0.565300
H 3.899599 -3.471089 -0.216559
H 3.462491 -1.976949 -1.226257
H 1.579845 -3.731425 0.457944
H 1.138562 -1.198110 -1.257273
H 0.075723 -2.603041 -1.317706
SCF: -674.505711
ZPE: 0.291216
H: -674.198765
G: -674.252288
SP SCF: -674.330307

1A_conf4
C -0.602258 1.175960 0.292761
C -0.799181 2.036422 -0.791671
C -0.135840 3.257821 -0.824110
N 0.701540 3.674629 0.134774
C 0.890770 2.849996 1.170837
C 0.269393 1.610083 1.292076
H 0.474931 0.985785 2.156742
H 1.575051 3.194987 1.942417
H -0.281321 3.935451 -1.662137
H -1.455127 1.764764 -1.613324
C -1.261950 -0.184193 0.361695
H -1.059569 -0.598542 1.357732
C -0.639328 -1.142852 -0.680621
C 0.838348 -1.358940 -0.463384
C 1.791148 -0.651578 -1.203499
C 3.154372 -0.815463 -0.957685
C 3.586024 -1.692366 0.036685
C 2.645013 -2.405884 0.780251
C 1.284110 -2.238491 0.530196
H 0.555393 -2.797864 1.113035
H 2.971717 -3.095025 1.554027
H 4.647096 -1.821311 0.229677
H 3.878832 -0.255688 -1.542511
H 1.461780 0.039330 -1.975735
H -0.817376 -0.739332 -1.684411
H -1.165747 -2.101416 -0.620267
C -2.793289 -0.087569 0.188777
C -3.494309 -1.372654 0.523893
C -4.248843 -2.076804 -0.321088
H -4.736244 -2.998676 -0.014279
H -4.406278 -1.750891 -1.347983
H -3.357511 -1.736861 1.543588
H -3.036633 0.211687 -0.837792
H -3.163701 0.702947 0.854015
SCF: -674.506177
ZPE: 0.290885
H: -674.199345
G: -674.253530
SP SCF: -674.330760

1A_conf5
C -0.949088 0.317275 0.110566
C -1.435950 1.043865 -0.979792
C -1.527101 2.427932 -0.892995
N -1.169858 3.129892 0.190854
C -0.704361 2.433830 1.233607
C -0.578718 1.047118 1.239693
H -0.186341 0.544405 2.118929
H -0.414873 3.011714 2.108197
H -1.904245 3.003780 -1.734926
H -1.740711 0.546706 -1.895786
C -0.784551 -1.186934 0.058382
H -0.504879 -1.522360 1.065101
C 0.363058 -1.582403 -0.900934
C 1.688712 -0.975745 -0.510002
C 2.173154 0.172102 -1.145116
C 3.370078 0.762837 -0.740575
C 4.102760 0.211476 0.309740
C 3.631582 -0.935891 0.949163
C 2.434703 -1.522047 0.540784
H 2.073141 -2.416367 1.043850
H 4.197754 -1.375932 1.765330
H 5.034828 0.670245 0.626675
H 3.728387 1.655600 -1.245276
H 1.603315 0.612086 -1.959860
H 0.098626 -1.277495 -1.920172
H 0.440085 -2.675715 -0.899531
C -2.088556 -1.908622 -0.337802
C -3.211479 -1.648690 0.623787
C -4.365444 -1.058687 0.309158
H -5.139853 -0.891857 1.053366
H -4.572636 -0.722663 -0.705520
H -3.038313 -1.961657 1.654698
H -1.874443 -2.985011 -0.362836
H -2.387106 -1.618123 -1.352271
SCF: -674.506683
ZPE: 0.290858
H: -674.199857
G: -674.254130
SP SCF: -674.331783

1A_conf6
```
C   -1.714432 -0.402377 -0.076594
C   -2.362189 -0.264240  1.154911
C   -3.685886 -0.669271  1.279693
N   -4.400303 -1.195010  0.275473
C   -3.780338 -1.325927 -0.902203
C   -2.457706 -0.949730 -1.122612
H   -2.015970 -1.080584 -2.106379
H   -4.368819 -1.751332 -1.711809
H   -4.200552 -0.566048  2.232042
H   -1.849985  0.158277  2.014299
C   -0.272536  0.016681 -0.268523
H   -0.017593 -0.148987 -1.322994
C    0.660165 -0.871513  0.589247
C    2.126318 -0.625358  0.324250
C    2.921409  0.072757  1.238480
C    4.268795  0.320356  0.973613
C    4.841927 -0.128188 -0.215302
C    4.059273 -0.828074 -1.134961
C    2.714639 -1.073915 -0.864637
H    2.112347 -1.624320 -1.584158
H    4.498231 -1.186420 -2.061877
H    5.890710  0.062577 -0.423329
H    4.869779  0.863808  1.697285
H    2.480322  0.428116  2.166806
H    0.422890 -1.920745  0.379270
H    0.442868 -0.699593  1.649667
C   -0.071982  1.515341  0.038102
C   -0.899781  2.403512 -0.843911
C   -1.843746  3.242376 -0.414749
H   -2.413188  3.858937 -1.105427
H   -2.077783  3.339027  0.644204
H   -0.701979  2.328606 -1.914484
H    0.988930  1.750305 -0.111926
H   -0.299338  1.711649  1.093247
```
SCF: -674.505322
ZPE: 0.290932
H: -674.198439
G: -674.252766
SP SCF: -674.329631

1A_conf7
```
C   -0.949083  0.317323  0.110597
C   -1.435897  1.043929 -0.979773
C   -1.526934  2.428005 -0.893007
N   -1.169624  3.129960  0.190824
C   -0.704173  2.433883  1.233588
C   -0.578643  1.047161  1.239704
H   -0.186299  0.544436  2.118946
H   -0.414631  3.011763  2.108161
H   -1.904039  3.003864 -1.734948
H   -1.740704  0.546775 -1.895755
C   -0.784673 -1.186901  0.058446
H   -0.504971 -1.522321  1.065158
C    0.362847 -1.582496 -0.900923
C    1.688573 -0.975931 -0.510092
C    2.434575 -1.522246  0.540680
C    3.631529 -0.936175  0.948961
C    4.102772  0.211118  0.309454
C    3.370081  0.762492 -0.740847
C    2.173081  0.171843 -1.145290
H    1.603235  0.611834 -1.960025
H    3.728440  1.655196 -1.245614
H    5.034900  0.669819  0.626313
H    4.197708 -1.376224  1.765119
H    2.072963 -2.416509  1.043810
H    0.098381 -1.277606 -1.920158
H    0.439787 -2.675814 -0.899484
C   -2.088763 -1.908489 -0.337640
C   -3.211599 -1.648448  0.624018
C   -4.365527 -1.058333  0.309463
H   -5.139872 -0.891427  1.053720
H   -4.572751 -0.722291 -0.705203
H   -3.038398 -1.961429  1.654920
H   -1.874739 -2.984896 -0.362672
H   -2.387356 -1.617980 -1.352094
```
SCF: -674.506683
ZPE: 0.290858
H: -674.199857
G: -674.254130
SP SCF: -674.331783

1A_conf8
C 1.260196 -0.482261 0.307378
C 0.847948 -0.908404 -0.957821
C 1.170403 -2.192295 -1.386055
N 1.864364 -3.069923 -0.651549
C 2.261041 -2.661461 0.560057
C 1.987853 -1.397494 1.072304
H 2.341214 -1.129706 2.064381
H 2.827472 -3.379261 1.148784
H 0.851096 -2.533209 -2.368083
H 0.274855 -0.261157 -1.613578
C 0.900785 0.873005 0.882361
H 1.661907 1.112779 1.634676
C -0.445538 0.798794 1.658853
C -1.647263 0.429320 0.823086
C -2.032092 -0.908819 0.676969
C -3.117951 -1.259896 -0.123593
C -3.841608 -0.273374 -0.793303
C -3.475606 1.064837 -0.648906
C -2.389570 1.410166 0.154719
H -2.115206 2.456282 0.267174
H -4.038200 1.841894 -1.158720
H -4.688464 -0.544547 -1.417052
H -3.398534 -2.304594 -0.223776
H -1.471592 -1.684400 1.193222
H -0.608330 1.776361 2.126295
H -0.327511 0.069794 2.467765
C 0.907422 2.015130 -0.150509
C 2.248436 2.216314 -0.797108
C 2.989693 3.319074 -0.676758
H 3.956447 3.408772 -1.165497
H 2.652012 4.170462 -0.087702
H 2.628329 1.388784 -1.397128
H 0.605212 2.937292 0.359184
H 0.153745 1.821975 -0.923890
SCF: -674.504935
ZPE: 0.291174
H: -674.197957
G: -674.251719
SP SCF: -674.329156

1A_conf9
C -0.670293 1.079429 0.173067
C -0.782834 2.026088 -0.849601
C -0.168088 3.264847 -0.704713
N 0.544004 3.619808 0.372696
C 0.651811 2.713018 1.350293
C 0.069585 1.449763 1.296858
H 0.203759 0.757825 2.123190
H 1.234516 3.008522 2.219623
H -0.249189 4.009122 -1.493513
H -1.336674 1.809617 -1.758236
C -1.275282 -0.303229 0.054623
H -1.191020 -0.785371 1.036308
C -0.472223 -1.154918 -0.958804
C 0.978311 -1.306634 -0.568943
C 1.973335 -0.519291 -1.156438
C 3.303315 -0.625908 -0.749567
C 3.657917 -1.524953 0.255358
C 2.674126 -2.318334 0.847249
C 1.346901 -2.208082 0.436438
H 0.584300 -2.828575 0.902350
H 2.941217 -3.024588 1.628393
H 4.692815 -1.608763 0.574147
H 4.061633 -0.003921 -1.216761
H 1.702654 0.189934 -1.934887
H -0.549053 -0.692787 -1.950061
H -0.933625 -2.145500 -1.025335
C -2.775965 -0.235523 -0.309248
C -3.477128 -1.559144 -0.184973
C -4.403643 -1.835108 0.734791
H -4.879459 -2.811068 0.786182
H -4.719526 -1.091119 1.464453
H -3.198707 -2.335065 -0.898009
H -2.877233 0.132567 -1.338792
H -3.264193 0.494831 0.345849
SCF: -674.504996
ZPE: 0.290956
H: -674.198094
G: -674.252266
SP SCF: -674.329499

CO2_conf1
C 0.000000 -0.000000 -0.000000
O -0.000000 0.000000 1.164992
O -0.000000 0.000000 -1.164992
SCF: -188.526209
ZPE: 0.011722
H: -188.510911
G: -188.532175
SP SCF: -188.560465

Comp-A"_conf1
C -3.207346 3.233461 1.131617
C -3.114746 1.886311 0.810301
C -1.954460 1.382038 0.203336
C -0.942852 2.287929 -0.096480
C -1.008900 3.654666 0.197024
C -2.159627 4.109953 0.832996
H -2.261246 5.158777 1.091211
C 0.161710 4.533563 -0.262913
C 1.439239 3.715706 -0.060407
C -0.010672 4.822836 -1.773720
H -0.939610 5.376666 -1.944101
H -0.047037 3.900750 -2.361518
H 0.828006 5.426806 -2.134985
C 2.682570 4.211864 0.330555
C 3.778968 3.358647 0.485344
C 3.638620 1.983976 0.317980
C 2.397491 1.453039 -0.046044
C 1.355547 2.343420 -0.284253
O 0.171946 1.804847 -0.715580
Pd 0.040660 -1.221414 -1.198362
P -1.845771 -0.407599 -0.195250
C -3.445331 -0.614424 -1.059506
C -3.729419 0.281291 -2.098991
C -4.930451 0.190379 -2.796355
C -5.848423 -0.809643 -2.478817
C -5.561077 -1.715013 -1.458804
C -4.365427 -1.618525 -0.747676
H -4.171917 -2.319310 0.057472
H -6.272897 -2.494102 -1.203677
H -6.784780 -0.882635 -3.023553
H -5.143027 0.896228 -3.593285
H -3.019326 1.061618 -2.359450
C -1.972812 -1.283163 1.405870
C -1.982236 -0.610167 2.630696
C -2.059354 -1.329995 3.822213
C -2.128428 -2.720146 3.801175
C -2.094722 -3.397909 2.582439
C -2.002191 -2.685284 1.391680
H -1.952368 -3.225297 0.450044
H -2.128741 -4.482844 2.557989
H -2.190140 -3.276462 4.731538
H -2.058623 -0.796631 4.767687
H -1.922938 0.472075 2.669297
P 1.971766 -0.321169 0.009356
C 3.509889 -1.160324 -0.515125
C 3.700771 -1.341255 -1.891352
C 4.857699 -1.952878 -2.367245
C 5.828182 -2.398610 -1.469742
C 5.640776 -2.226246 -0.098946
C 4.486883 -1.607165 0.379828
H 4.352132 -1.476128 1.449477
H 6.394348 -2.572748 0.601797
H 6.727777 -2.882071 -1.838524
H 4.998655 -2.086385 -3.435484
H 2.945590 -0.995962 -2.595017
C 1.820753 -0.665940 1.800695
C 1.533132 -1.985588 2.176595
C 1.390271 -2.319350 3.518176
C 1.505608 -1.333216 4.499403
C 1.763757 -0.016110 4.130638
C 1.925388 0.319138 2.785186
H 2.133654 1.349812 2.515439
H 1.844836 0.757480 4.888369
H 1.382916 -1.591953 5.546860
H 1.173837 -3.345732 3.797645
H 1.422229 -2.756521 1.417263
H 4.480848 1.323531 0.502724
H 4.741322 3.770444 0.771963
H 2.811518 5.272766 0.517870
C 0.212656 5.862962 0.493311
H 1.045545 6.473905 0.134464
H -0.699027 6.440066 0.316332
H 0.328694 5.713310 1.571536
H -3.940377 1.219627 1.041387
H -4.102916 3.609979 1.614960
C -1.118697 -2.487199 -2.302092
C -1.246515 -1.948010 -3.664067
H -0.324828 -1.696785 -4.189873
C -2.420370 -1.749867 -4.282771
H -3.359690 -2.020613 -3.806537
H -2.471495 -1.332431 -5.284683
H -0.340759 -3.266666 -2.205396
H -2.065621 -2.834583 -1.886162
SCF: -2508.020992
ZPE: 0.675282
H: -2507.303627
G: -2507.408642
SP SCF: -2507.613379

Comp-A'_conf1

C	-3.487868	3.136206	0.567171
C	-3.412330	1.757404	0.406718
C	-2.177771	1.123700	0.244166
C	-1.008708	1.910435	0.249423
C	-1.058513	3.292186	0.437305
C	-2.317847	3.882630	0.586215
H	-2.384185	4.958043	0.720656
C	0.201010	4.150813	0.482768
C	1.441755	3.276332	0.337416
C	0.163087	5.174898	-0.671274
H	-0.710881	5.827834	-0.584390
H	0.120588	4.667376	-1.640207
H	1.054551	5.808995	-0.652821
C	2.717129	3.849147	0.395327
C	3.865689	3.080405	0.269212
C	3.756246	1.705573	0.077999
C	2.506760	1.089069	0.007084
C	1.366006	1.898854	0.139799
O	0.160633	1.242376	0.050328
Pd	-4.319239	-1.622952	0.384430
P	-2.207829	-0.684590	0.040360
C	-0.993423	-1.409055	1.196666
C	-0.591905	-0.713186	2.339242
C	0.268667	-1.313383	3.256208
C	0.718676	-2.616043	3.047682
C	0.299906	-3.320874	1.920227
C	-0.554829	-2.721735	0.999152
H	-0.856677	-3.278655	0.117147
H	0.648003	-4.335163	1.749839
H	1.394053	-3.079835	3.760254
H	0.588980	-0.758251	4.132829
H	-0.932378	0.303009	2.516207
C	-1.710329	-1.061623	-1.678734
C	-1.141793	-0.113876	-2.535847
C	-0.899429	-0.433053	-3.869221
C	-1.210465	-1.699972	-4.358017
C	-1.776786	-2.651173	-3.511550
C	-2.038402	-2.329310	-2.182439
H	-2.506931	-3.070206	-1.537406
H	-2.027275	-3.638904	-3.886229
H	-1.011856	-1.945054	-5.396858
H	-0.456172	0.310087	-4.524277
H	-0.889862	0.877793	-2.178307
P	2.295369	-0.730155	-0.215392
C	2.351737	-0.906425	-2.046695
C	2.673469	0.123354	-2.936566
C	2.737054	-0.121172	-4.308779
C	2.488799	-1.398920	-4.806478
C	2.155621	-2.429795	-3.927579
C	2.074549	-2.180934	-2.560234
H	1.801951	-2.987774	-1.883254
H	1.946124	-3.425220	-4.308271
H	2.544928	-1.589505	-5.874322
H	2.989055	0.688778	-4.987503
H	2.886060	1.120958	-2.563233
C	3.973854	-1.327252	0.261162
C	5.007023	-1.605592	-0.640387
C	6.241042	-2.068965	-0.182573
C	6.460700	-2.252387	1.181617
C	5.436530	-1.978875	2.089176
C	4.200514	-1.530272	1.629861
H	3.402559	-1.332132	2.342318
H	5.597438	-2.124233	3.153549
H	7.422652	-2.610721	1.536234
H	7.033052	-2.281029	-0.895143
H	4.856260	-1.457688	-1.705798
H	4.655365	1.105392	-0.018431
H	4.843873	3.548107	0.318568
H	2.811390	4.921042	0.540536
C	0.256450	4.891172	1.836561
H	1.144825	5.526495	1.894952
H	-0.617834	5.535400	1.967546
H	0.288025	4.178749	2.667012
H	-4.319488	1.158779	0.408719
H	-4.450345	3.622410	0.685297
C	-6.091550	-2.704875	1.245558
C	-5.694682	-1.669351	2.077943
H	-6.325362	-0.791927	2.199182
C	-4.314050	-1.613136	2.455200
H	-3.786472	-2.532689	2.710451
H	-3.937860	-0.714752	2.937880
H	-7.058385	-2.667585	0.752310
H	-5.589801	-3.671748	1.275555

SCF: -2508.013350
ZPE: 0.675288
H: -2507.295864
G: -2507.401382
SP SCF: -2507.608977

Comp-A_conf1

C	-3.203290	3.291098	1.226282
C	-3.134219	1.935226	0.932582
C	-2.006249	1.410282	0.287751
C	-1.005648	2.301808	-0.080957
C	-1.040230	3.671709	0.195483
C	-2.159647	4.151068	0.869938
H	-2.238088	5.205023	1.115489
C	0.132523	4.520138	-0.309432
C	1.396227	3.674795	-0.130863
C	-0.073651	4.793476	-1.818999
H	-0.993946	5.366426	-1.971949
H	-0.147876	3.864813	-2.392588
H	0.768425	5.372999	-2.211128
C	2.654443	4.148398	0.236477
C	3.738677	3.275961	0.363595
C	3.573666	1.907173	0.178543
C	2.316628	1.395951	-0.165049
C	1.280787	2.303571	-0.357294
O	0.073982	1.790747	-0.746310
Pd	0.023895	-1.281936	-1.273932
P	-1.859519	-0.376504	-0.104541
C	-3.450423	-0.619590	-0.989273
C	-3.504292	-0.198685	-2.324252
C	-4.689550	-0.295465	-3.045884
C	-5.832352	-0.824210	-2.442748
C	-5.783201	-1.246119	-1.116466
C	-4.597472	-1.140209	-0.387408
H	-4.578563	-1.464292	0.648440
H	-6.669126	-1.656138	-0.640926
H	-6.756664	-0.906170	-3.006527
H	-4.720725	0.036383	-4.079236
H	-2.613764	0.202963	-2.803198
C	-2.062453	-1.239706	1.493994
C	-1.964101	-0.591058	2.726652
C	-2.094232	-1.316855	3.910478
C	-2.319881	-2.689954	3.872316
C	-2.397511	-3.346423	2.643137
C	-2.259773	-2.627525	1.460400
H	-2.320732	-3.146872	0.506861
H	-2.564021	-4.418765	2.605478
H	-2.424158	-3.250629	4.796324
H	-2.012348	-0.803039	4.863342
H	-1.781972	0.477459	2.775778
P	1.926319	-0.391220	-0.147847
C	3.541428	-1.121563	-0.619345
C	4.037359	-0.823930	-1.896313
C	5.255894	-1.344613	-2.317580
C	5.990082	-2.177881	-1.470923
C	5.501527	-2.477780	-0.202508
C	4.282468	-1.948556	0.226339
H	3.925312	-2.180369	1.224630
H	6.069966	-3.119502	0.463904
H	6.940104	-2.587423	-1.800480
H	5.632429	-1.101826	-3.306640
H	3.472792	-0.175197	-2.562257
C	1.729017	-0.778699	1.631343
C	1.359465	-2.088427	1.969827
C	1.213895	-2.456833	3.302104
C	1.406598	-1.512836	4.311370
C	1.744057	-0.203331	3.980849
C	1.910715	0.164964	2.645178
H	2.182026	1.188260	2.405897
H	1.882363	0.538572	4.761519
H	1.279908	-1.796553	5.351798
H	0.934105	-3.475354	3.551977
H	1.189980	-2.825221	1.187984
H	4.412173	1.234352	0.330384
H	4.713247	3.667606	0.636678
H	2.803968	5.205937	0.427091
C	0.232404	5.857901	0.426379
H	1.065546	6.447832	0.034258
H	-0.673710	6.449238	0.268700
H	0.377626	5.720757	1.502742
H	-3.950996	1.278355	1.217369
H	-4.074839	3.687138	1.737478
C	1.205558	-2.596285	-2.530665
C	-0.081048	-2.487395	-3.095536
H	-0.220481	-1.916677	-4.011162
C	-1.192473	-2.789061	-2.293885
H	-1.121343	-3.569617	-1.536505
H	-2.191078	-2.555961	-2.647926
H	2.058477	-2.198954	-3.071025
H	1.426772	-3.392707	-1.820464

SCF: -2508.065398
ZPE: 0.676499
H: -2507.347346
G: -2507.450859
SP SCF: -2507.655163

Int-A_conf2

C	-2.656215	2.724050	1.708697
C	-1.499021	2.932934	0.960282
C	-0.485013	1.972043	0.949593
C	-0.676617	0.831743	1.727442
C	-1.816174	0.590233	2.491514
C	-2.816780	1.561195	2.462353
H	-3.728657	1.420386	3.032971
C	-1.827688	-0.682730	3.335522
C	-1.152643	-1.769765	2.499074
C	-0.962815	-0.432525	4.596581
H	-1.410041	0.361921	5.203675
H	0.056649	-0.130937	4.336484
H	-0.908339	-1.344784	5.200170
C	-1.522251	-3.112987	2.470295
C	-0.773431	-4.037003	1.742266
C	0.352407	-3.634387	1.028855
C	0.740472	-2.289877	1.009390
C	-0.037728	-1.397685	1.747216
O	0.346089	-0.082049	1.728519
Pd	1.827092	0.243516	-1.408065
H	3.911104	-0.209529	-2.989437
C	2.845196	-0.261236	-3.212605
C	2.123592	0.923308	-3.454957
H	2.642603	1.880409	-3.425849
C	0.883676	0.912497	-4.328476
H	0.962872	0.178229	-5.133638
H	0.702586	1.896326	-4.762063
O	-0.349700	0.640260	-3.602524
C	-0.580774	-0.627605	-3.267336
O	-0.058930	-1.600181	-3.781599
N	-1.536758	-0.730089	-2.264489
C	-1.849527	-1.996582	-1.753719
C	-2.824832	-2.169881	-0.849828
C	-3.647343	-1.061415	-0.360103
C	-3.207117	0.244445	-0.851948
C	-2.210482	0.377653	-1.739797
H	-1.876065	1.340467	-2.096512
H	-3.675455	1.152416	-0.492374
C	-4.705024	-1.264872	0.456965
H	-4.938390	-2.291822	0.736716
C	-5.642770	-0.211077	0.981748
H	-5.186228	0.782065	0.913613
H	-5.832396	-0.389081	2.047081
C	-6.973827	-0.193236	0.249824
C	-7.020602	0.086720	-1.122065
C	-8.234167	0.112134	-1.804349
C	-9.426751	-0.149398	-1.125857
C	-9.391987	-0.433319	0.237648
C	-8.172663	-0.453377	0.918538
H	-8.154049	-0.674708	1.983247
H	-10.313239	-0.640796	0.775037
H	-10.373368	-0.133005	-1.658193
H	-8.250719	0.333659	-2.867864
H	-6.095009	0.281803	-1.658846
H	-3.008767	-3.171941	-0.476046
H	-1.239263	-2.803958	-2.135053
H	2.511906	-1.197998	-3.652667
P	1.057881	2.049254	-0.048908
C	2.323358	2.408196	1.238906
C	3.669206	2.327449	0.863597
C	4.678606	2.610362	1.779765
C	4.355236	2.958387	3.090635
C	3.017980	3.028987	3.476592
C	2.007188	2.761282	2.554267
H	0.969993	2.830907	2.868658
H	2.757473	3.294301	4.497218
H	5.142637	3.164525	3.809814
H	5.718204	2.541238	1.473858
H	3.927462	2.031330	-0.150687
C	0.854610	3.685340	-0.868527
C	0.213644	3.706937	-2.112519
C	-0.011051	4.910250	-2.777199
C	0.421600	6.109611	-2.211002
C	1.071929	6.096968	-0.977664
C	1.283365	4.892334	-0.306765
H	1.784570	4.899625	0.656699
H	1.415259	7.026754	-0.533348
H	0.257169	7.048991	-2.730781
H	-0.514793	4.909723	-3.739597
H	-0.107172	2.771442	-2.561184
P	2.139104	-1.628196	0.003982
C	3.470925	-1.319426	1.233451
C	4.747568	-1.031530	0.730165
C	5.805946	-0.766449	1.592207
C	5.596549	-0.753004	2.972158
C	4.326568	-1.015478	3.478781
C	3.269488	-1.305939	2.614546
H	2.291472	-1.525781	3.031222
H	4.152319	-0.999742	4.550750
H	6.418371	-0.531413	3.646766
H	6.791318	-0.555814	1.186650
H	4.915257	-1.021516	-0.344297
C	2.709276	-3.176504	-0.812229
C	2.123869	-3.510305	-2.039074
C	2.464312	-4.696410	-2.686625
C	3.405049	-5.556682	-2.120749
C	3.995132	-5.229640	-0.900133
C	3.644576	-4.049280	-0.245592
H	4.101012	-3.815475	0.711739
H	4.726368	-5.896203	-0.451929
H	3.678271	-6.476907	-2.629021
H	1.998976	-4.942624	-3.636795
H	1.395588	-2.841199	-2.490748
H	0.917837	-4.372272	0.470208
H	-1.068713	-5.081741	1.732577
H	-2.393248	-3.450937	3.021943
C	-3.234789	-1.082891	3.778733
H	-3.697418	-0.281885	4.362296
H	-3.873457	-1.309373	2.920938
H	-3.194409	-1.963415	4.426490
H	-1.394538	3.840597	0.374180
H	-3.442820	3.472650	1.701922

SCF: -3253.949041
ZPE: 0.911172
H: -3252.981694
G: -3253.113462
SP SCF: -3253.441003

Int-A_conf3

C	1.712772	-3.810672	0.938066
C	0.388534	-3.555798	0.591411
C	-0.159512	-2.279127	0.760442
C	0.674027	-1.301579	1.304192
C	1.994652	-1.528792	1.692998
C	2.511583	-2.805331	1.482192
H	3.540110	-3.026803	1.747489
C	2.731985	-0.375746	2.373756
C	2.318874	0.900337	1.643389
C	2.234696	-0.278053	3.837816
H	2.473859	-1.200963	4.376783
H	1.152907	-0.119943	3.889875
H	2.727420	0.559291	4.343631
C	3.136576	2.003173	1.398606
C	2.624679	3.144754	0.781365
C	1.288047	3.202524	0.390340
C	0.443530	2.110781	0.606774
C	0.990059	0.994412	1.236897
O	0.135315	-0.051977	1.471150
Pd	-2.213810	0.127813	-1.098321
H	-4.601513	-0.557434	-2.036043
C	-3.650237	-0.463078	-2.560238
C	-3.178411	0.812538	-2.925144
H	-3.777308	1.690726	-2.687703
C	-2.255669	0.991604	-4.116045
H	-2.326164	2.003461	-4.515871
H	-2.479588	0.278198	-4.912638
O	-0.840547	0.862424	-3.792344
C	-0.380858	-0.374593	-3.613936
O	-0.912099	-1.389676	-4.026614
N	0.822241	-0.387023	-2.918726
C	1.388375	-1.622599	-2.574634
C	2.592839	-1.707000	-1.991943
C	3.410406	-0.525056	-1.719077
C	2.721412	0.736676	-1.990890
C	1.500301	0.778421	-2.546713
H	0.979664	1.705109	-2.738487
H	3.185148	1.679099	-1.723223
C	4.681139	-0.610106	-1.268340
H	5.097599	-1.600095	-1.088978
C	5.584856	0.576615	-1.026774
H	5.062711	1.338196	-0.435393
H	5.823928	1.056828	-1.987654
C	6.879418	0.218981	-0.331685
C	7.212554	0.782354	0.903251
C	8.405230	0.449912	1.546829
C	9.286798	-0.456102	0.960003
C	8.967971	-1.022622	-0.275404
C	7.775604	-0.686545	-0.913386
H	7.536568	-1.131283	-1.876836
H	9.650681	-1.726344	-0.743384
H	10.215292	-0.718156	1.458937
H	8.643006	0.898185	2.507545
H	6.527431	1.486446	1.369282
H	2.972027	-2.689025	-1.728761
H	0.774689	-2.483322	-2.802222
H	-3.353575	-1.336448	-3.135822
P	-1.860007	-1.800607	0.226633
C	-2.814390	-1.706013	1.795481
C	-4.208103	-1.597845	1.686491
C	-5.002108	-1.494768	2.823342
C	-4.411603	-1.466717	4.087826
C	-3.026812	-1.552189	4.203188
C	-2.231244	-1.680295	3.063330
H	-1.154909	-1.763616	3.176860
H	-2.557982	-1.523884	5.182530
H	-5.028642	-1.371386	4.976517
H	-6.081104	-1.421602	2.722035
H	-4.674648	-1.599952	0.704101
C	-2.437331	-3.396540	-0.485807
C	-3.032465	-4.409904	0.273831
C	-3.403843	-5.613840	-0.323417
C	-3.172804	-5.822988	-1.682899
C	-2.571119	-4.821360	-2.444221
C	-2.211914	-3.612855	-1.850021
H	-1.751587	-2.833781	-2.452447
H	-2.387317	-4.974883	-3.503749
H	-3.461852	-6.761736	-2.146617
H	-3.869599	-6.390443	0.276478
H	-3.204714	-4.267359	1.336620
P	-1.316873	1.995007	0.086951
C	-2.196813	2.152295	1.696427
C	-3.569888	1.881979	1.712098
C	-4.303637	2.006845	2.888719
C	-3.669251	2.386559	4.070868
C	-2.299801	2.645389	4.067294
C	-1.568310	2.534050	2.885543
H	-0.503767	2.749038	2.898099
H	-1.796536	2.936164	4.984982
H	-4.238257	2.470808	4.992187
H	-5.367819	1.790872	2.885074
H	-4.064927	1.562741	0.797692
C	-1.564822	3.660383	-0.658674
C	-1.322702	3.795132	-2.030441
C	-1.455296	5.030866	-2.659448
C	-1.850375	6.147815	-1.922994
C	-2.104445	6.021323	-0.557817
C	-1.958179	4.785781	0.072853
H	-2.153236	4.705095	1.138169
H	-2.415905	6.886327	0.020641
H	-1.964707	7.111017	-2.411638
H	-1.259043	5.118671	-3.724262
H	-1.030843	2.921693	-2.605842
H	0.907448	4.096683	-0.093341
H	3.274354	3.996115	0.602127
H	4.180104	1.981878	1.695639
C	4.246076	-0.579475	2.381850
H	4.509008	-1.482138	2.940980
H	4.643775	-0.669664	1.367766
H	4.743172	0.256548	2.881538
H	-0.212041	-4.354047	0.168803
H	2.125765	-4.802922	0.784213

SCF: -3253.948919
ZPE: 0.910769
H: -3252.981932
G: -3253.113539
SP SCF: -3253.441567

Int-B_conf1

C	3.112812	0.703305	-2.110814
C	4.335655	1.025747	-1.621837
C	5.597718	0.205535	-1.732411
C	6.377837	0.146419	-0.434002
C	6.556903	-1.059522	0.249271
C	7.237837	-1.103399	1.467035
C	7.757107	0.065645	2.020274
C	7.599184	1.274636	1.340143
C	6.920004	1.310844	0.124494
H	6.801846	2.258547	-0.396072
H	8.007787	2.190812	1.757647
H	8.285454	0.035861	2.968829
H	7.360698	-2.052128	1.982371
H	6.151731	-1.976197	-0.173242
H	5.372539	-0.815102	-2.055778
H	6.251440	0.635875	-2.506661
H	4.439758	1.995382	-1.135274
C	1.973944	1.614689	-1.988925
C	0.787897	1.364969	-2.577644
N	0.544942	0.208510	-3.297117
C	-0.693404	0.044366	-4.063378
O	-0.847849	-1.072920	-4.594924
O	-1.436469	1.046907	-4.086366
C	1.554714	-0.730178	-3.364257
C	2.773489	-0.533492	-2.816459
H	3.506490	-1.325537	-2.921213
H	1.292519	-1.631938	-3.902719
H	-0.048256	2.048284	-2.523333
H	2.086975	2.535071	-1.423642
C	0.909820	4.104394	0.927627
C	-0.350229	3.697859	0.498217
C	-0.719126	2.349667	0.571896
C	0.212677	1.461737	1.100531
C	1.483492	1.832427	1.539682
C	1.821216	3.179927	1.438315
H	2.802212	3.519577	1.753359
C	2.382433	0.730176	2.098310
C	2.136109	-0.501964	1.229516
C	3.855872	1.138102	2.119346
H	4.473809	0.331833	2.524928
H	4.005257	2.008029	2.765621
H	4.218848	1.375165	1.116723
C	3.096616	-1.438691	0.854161
C	2.739515	-2.572143	0.125639
C	1.415586	-2.790155	-0.248278
C	0.433014	-1.857014	0.089835
C	0.829161	-0.739661	0.817488
O	-0.162216	0.144570	1.159719
Pd	-2.639216	-0.298645	-1.330881
P	-2.320568	1.696837	-0.048106
C	-3.023241	3.200658	-0.822944
C	-2.721288	3.443838	-2.168220
C	-3.171841	4.608412	-2.785450
C	-3.934033	5.531846	-2.069712
C	-4.240853	5.290017	-0.731340
C	-3.782331	4.130908	-0.106384
H	-4.014201	3.960955	0.940854
H	-4.833325	6.005488	-0.168781
H	-4.290517	6.435871	-2.554690
H	-2.933444	4.787622	-3.829660
H	-2.150220	2.716788	-2.743435
C	-3.339390	1.438925	1.455622
C	-4.706674	1.186413	1.275166
C	-5.528901	0.942236	2.369405
C	-4.989728	0.921216	3.656824
C	-3.630052	1.155969	3.840689
C	-2.806874	1.421510	2.745457
H	-1.752192	1.616423	2.911539
H	-3.202460	1.136226	4.838571
H	-5.627980	0.717332	4.511393
H	-6.588348	0.758677	2.216893
H	-5.131891	1.190638	0.274379
P	-1.345869	-2.013726	-0.305245
C	-1.373946	-3.628894	-1.169029
C	-1.679921	-4.824230	-0.513329
C	-1.650153	-6.032541	-1.210611
C	-1.306067	-6.053482	-2.560769
C	-0.998576	-4.860802	-3.217391
C	-1.037234	-3.651615	-2.528519
H	-0.821178	-2.726790	-3.062370
H	-0.737069	-4.867853	-4.271460
H	-1.282441	-6.994801	-3.101967
H	-1.894128	-6.956311	-0.694240
H	-1.942338	-4.822237	0.540345
C	-2.113943	-2.350584	1.326570
C	-3.503919	-2.229286	1.434580
C	-4.142120	-2.477463	2.646470
C	-3.394559	-2.836564	3.767215
C	-2.009390	-2.955907	3.667605
C	-1.370595	-2.719028	2.451578
H	-0.291218	-2.820817	2.387973
H	-1.421462	-3.233638	4.537433
H	-3.889938	-3.016876	4.716722
H	-5.220573	-2.375257	2.718251
H	-4.088334	-1.930530	0.568028
H	1.155642	-3.679104	-0.812719
H	3.501142	-3.292641	-0.156017
H	4.133343	-1.289609	1.132504
C	1.928650	0.407442	3.543108
H	0.878566	0.100703	3.581464
H	2.538139	-0.405515	3.951630
H	2.052681	1.290064	4.179586
H	-1.034985	4.432274	0.089100
H	1.187512	5.151120	0.854851
C	-4.200329	0.630547	-2.558035
C	-4.532401	-0.719012	-2.366994
H	-5.368382	-0.985315	-1.722672
C	-3.577970	-1.695425	-2.707558
H	-2.903117	-1.542279	-3.552724
H	-3.746855	-2.724614	-2.405543
H	-4.843113	1.401960	-2.144934
H	-3.539352	0.917654	-3.376702

SCF: -3253.962299
ZPE: 0.909733
H: -3252.996045
G: -3253.128217
SP SCF: -3253.454072

Int-B_conf11
C 3.936807 -1.558626 -2.694604
C 5.286659 -1.414602 -2.678189
C 6.200354 -1.775940 -1.532222
C 6.455124 -0.609210 -0.595445
C 7.372156 0.394653 -0.928876
C 7.580389 1.483095 -0.083192
C 6.870229 1.588738 1.113666
C 5.952819 0.596910 1.455355
C 5.753213 -0.492080 0.607430
H 5.039985 -1.265853 0.877680
H 5.389975 0.670078 2.382295
H 7.027545 2.439267 1.770635
H 8.299006 2.250451 -0.358265
H 7.930621 0.320175 -1.859510
H 5.788297 -2.610988 -0.954673
H 7.160247 -2.120140 -1.935471
H 5.759622 -0.961645 -3.548919
C 3.132014 -1.156318 -3.848319
C 1.790177 -1.297506 -3.873840
N 1.079458 -1.808957 -2.804675
C -0.379459 -1.996492 -2.888694
O -0.920564 -2.413247 -1.850482
O -0.878393 -1.707128 -3.996525
C 1.784467 -2.201491 -1.686534
C 3.130107 -2.102795 -1.601207
H 3.600431 -2.413217 -0.675149
H 1.167565 -2.581526 -0.881379
H 1.179819 -1.019763 -4.723419
H 3.627286 -0.736971 -4.719413
C -4.122372 -3.776030 0.807596
C -4.201197 -2.572917 0.112872
C -3.251314 -1.571164 0.332366
C -2.270502 -1.815414 1.287571
C -2.147349 -3.013867 1.988250
C -3.095904 -4.000819 1.723751
H -3.046219 -4.954678 2.237769
C -1.023739 -3.130657 3.018188
C 0.166197 -2.334648 2.483097
C -1.495825 -2.463715 4.333374
H -1.768606 -1.414944 4.176959
H -0.697225 -2.502625 5.081913
H -2.371227 -2.990743 4.727468
C 1.508444 -2.639304 2.711111
C 2.520100 -1.787369 2.269216
C 2.214014 -0.621332 1.572901
C 0.883020 -0.303398 1.299151
C -0.100918 -1.165953 1.773843
O -1.392594 -0.791698 1.525862
Pd -1.170501 0.984413 -1.481116
P -3.183861 -0.013788 -0.633149
C -3.982181 1.245753 0.432487
C -4.296124 1.020193 1.773448
C -4.876252 2.033424 2.538165
C -5.161863 3.269557 1.965007
C -4.849654 3.501162 0.624449
C -4.248629 2.501195 -0.131605
H -3.996008 2.693934 -1.171881
H -5.063279 4.464890 0.171585
H -5.619407 4.053760 2.560852
H -5.108167 1.849387 3.582908
H -4.097234 0.056168 2.231664
C -4.441444 -0.368351 -1.922293
C -4.045857 -1.165293 -3.004628
C -4.966170 -1.507366 -3.991344
C -6.284411 -1.055413 -3.912109
C -6.681115 -0.265207 -2.835314
C -5.764518 0.073588 -1.838864
H -6.093001 0.677121 -0.998518
H -7.706253 0.086219 -2.763074
H -6.998842 -1.319282 -4.686502
H -4.648916 -2.125463 -4.826192
H -3.018295 -1.517373 -3.082306
P 0.327931 1.171124 0.368708
C -0.401658 2.246137 1.659935
C -0.184448 2.034424 3.024534
C -0.736262 2.902398 3.964849
C -1.497487 3.993778 3.549286
C -1.711657 4.212803 2.189348
C -1.173396 3.338049 1.249605
H -1.357065 3.503472 0.190929
H -2.310362 5.056088 1.859444
H -1.927213 4.669053 4.283273
H -0.567840 2.725130 5.022926
H 0.415484 1.194249 3.361443
C 1.921685 1.986158 -0.031870
C 2.362582 3.143897 0.613671
C 3.574442 3.731717 0.246609
C 4.358275 3.160266 -0.752504
C 3.930633 1.994691 -1.388578
C 2.715022 1.419287 -1.037286
H 2.384495 0.519341 -1.546618
H 4.541410 1.528397 -2.155447
H 5.304187 3.614861 -1.030309
H 3.905380 4.635149 0.750478
H 1.767837 3.597958 1.400147
H 3.011941 0.025754 1.226578
H 3.557024 -2.036834 2.472370
H 1.777131 -3.540700 3.251447
C -0.656009 -4.589465 3.302627
H -0.313238 -5.102596 2.398596
H 0.132522 -4.647300 4.058090
H -1.515330 -5.131410 3.706613
H -4.989349 -2.430685 -0.617799
H -4.858320 -4.551848 0.621849
C 0.160999 2.034771 -2.839202
C -1.160836 2.336836 -3.214758
H -1.553583 3.335362 -3.032386
C -2.043230 1.275639 -3.466471
H -1.681427 0.338268 -3.896390
H -3.102836 1.484729 -3.576323
H 0.802777 2.828868 -2.471792
H 0.669027 1.171361 -3.268607
SCF: -3253.959613
ZPE: 0.909162
H: -3252.993646
G: -3253.126169
SP SCF: -3253.451807

Int-B_conf15
C -3.229584 -2.180479 1.904761
C -4.566046 -2.115435 1.670684
C -5.368743 -0.872270 1.418604
C -6.120506 -0.812694 0.096629
C -6.759419 0.380397 -0.268280
C -7.431770 0.497708 -1.481722
C -7.487989 -0.588013 -2.358883
C -6.869882 -1.784085 -2.001898
C -6.192720 -1.893341 -0.785170
H -5.696701 -2.825451 -0.533682
H -6.905679 -2.637426 -2.673930
H -8.009019 -0.500017 -3.307881
H -7.910257 1.436905 -1.745895
H -6.715591 1.233178 0.406445
H -6.113348 -0.747378 2.219888
H -4.731364 0.016792 1.475470
H -5.124029 -3.051020 1.708871
C -2.559751 -3.452500 2.182972
C -1.251113 -3.524083 2.503983
N -0.446555 -2.403818 2.577698
C 0.924726 -2.492631 3.091695
O 1.518285 -1.395224 3.191269
O 1.319118 -3.636028 3.373852
C -1.005584 -1.187557 2.246480
C -2.307332 -1.043716 1.918226
H -2.650771 -0.051848 1.646124
H -0.308487 -0.358439 2.264249
H -0.744526 -4.453127 2.731246
H -3.135299 -4.372946 2.142079
C 0.857127 -4.273959 -0.446064
C 1.885637 -3.397965 -0.108113
C 1.735061 -2.028982 -0.334303
C 0.547345 -1.599744 -0.920071
C -0.480733 -2.448396 -1.317444
C -0.311120 -3.806199 -1.045665
H -1.090296 -4.514331 -1.306313
C -1.656299 -1.835810 -2.080005
C -1.918759 -0.456378 -1.477226
C -1.231143 -1.655494 -3.557270
H -1.000882 -2.628797 -4.003584
H -0.344942 -1.018703 -3.646593
H -2.044645 -1.193087 -4.126522
C -3.167671 0.151762 -1.383390
C -3.305046 1.416927 -0.812048
C -2.203258 2.077802 -0.281035
C -0.933919 1.489674 -0.344263
C -0.827637 0.261637 -0.989322
O 0.434685 -0.247610 -1.115551
Pd 2.306821 1.008368 1.561972
P 2.964767 -0.737619 0.078348
C 3.477719 -0.075918 -1.550333
C 4.138177 1.156276 -1.576378
C 4.583566 1.692091 -2.782091
C 4.356498 1.007224 -3.974845
C 3.693449 -0.219448 -3.955882
C 3.261793 -0.763717 -2.748004
H 2.755654 -1.724836 -2.744732
H 3.513398 -0.756940 -4.882221
H 4.692126 1.429776 -4.917309
H 5.094955 2.649549 -2.790104
H 4.300154 1.702059 -0.650324
C 4.412223 -1.729846 0.614802
C 5.560221 -1.860428 -0.172077
C 6.639622 -2.619797 0.282083
C 6.575834 -3.260409 1.517439
C 5.428896 -3.136533 2.303089
C 4.354784 -2.369997 1.860818
H 3.465818 -2.269620 2.480829
H 5.368176 -3.634952 3.266101
H 7.415253 -3.854305 1.867256
H 7.527923 -2.711061 -0.336109
H 5.624041 -1.374585 -1.140610
P 0.549213 2.209629 0.461899
C 1.350848 3.212219 -0.843787
C 0.945353 3.167045 -2.178640
C 1.614420 3.925377 -3.140122
C 2.680296 4.742023 -2.772377
C 3.090831 4.789932 -1.438921
C 2.439155 4.018670 -0.482943
H 2.773938 4.049374 0.551240
H 3.925607 5.419858 -1.146220
H 3.196369 5.334196 -3.522242
H 1.296644 3.875822 -4.177161
H 0.108151 2.544325 -2.479107
C -0.258461 3.431942 1.566859
C -0.843799 2.939638 2.740801
C -1.509814 3.797112 3.609543
C -1.587614 5.161135 3.320441
C -1.004645 5.656619 2.156818
C -0.346339 4.794544 1.277429
H 0.088813 5.193154 0.366243
H -1.064543 6.715763 1.924553
H -2.100928 5.833199 4.001612
H -1.962340 3.402240 4.514118
H -0.778599 1.880315 2.978558
H -2.338119 3.040335 0.200128
H -4.287692 1.874240 -0.755476
H -4.052492 -0.359820 -1.745299
C -2.899488 -2.725855 -2.018407
H -2.697488 -3.699115 -2.474556
H -3.226728 -2.878440 -0.985513
H -3.722918 -2.278226 -2.581199
H 2.789716 -3.783892 0.348214
H 0.969125 -5.333686 -0.240389
C 2.304447 2.345314 3.283631
C 3.636298 1.988969 3.010224
H 4.316395 2.730167 2.595072
C 3.960798 0.624531 2.927753
H 3.440471 -0.108726 3.543035
H 4.932098 0.335143 2.539032
H 1.997600 3.381739 3.185029
H 1.699992 1.730061 3.949309
SCF: -3253.959106
ZPE: 0.907683
H: -3252.994056
G: -3253.127953
SP SCF: -3253.450129

Int-B_conf4
C -4.117185 -1.378234 -2.210575
C -5.448666 -1.593373 -2.046932
C -6.486165 -0.511861 -1.859452
C -6.710826 -0.094701 -0.415843
C -7.197092 -1.013297 0.524098
C -7.407063 -0.640366 1.848878
C -7.131625 0.665079 2.263459
C -6.647665 1.587807 1.338932
C -6.440232 1.207648 0.010865
H -6.058630 1.934654 -0.702799
H -6.427526 2.606081 1.648600
H -7.293482 0.957244 3.297066
H -7.785956 -1.368213 2.561260
H -7.410099 -2.033144 0.211878
H -6.223472 0.379552 -2.441518
H -7.440933 -0.869224 -2.264749
H -5.804080 -2.622606 -2.055101
C -3.168138 -2.468122 -2.428372
C -1.856955 -2.244221 -2.653566
N -1.316460 -0.972919 -2.671011
C 0.086408 -0.759501 -3.049979
O 0.450639 0.433816 -3.061458
O 0.722950 -1.793901 -3.321742
C -2.153819 0.090254 -2.410815
C -3.477682 -0.063710 -2.185539
H -4.059988 0.822195 -1.958054
H -1.660661 1.054206 -2.388896
H -1.139271 -3.034834 -2.828607
H -3.525395 -3.493879 -2.415921
C -0.792483 -4.069954 0.313359
C 0.498526 -3.649756 0.004581
C 0.868021 -2.319173 0.215074
C -0.083888 -1.466645 0.764117
C -1.381385 -1.850790 1.091029
C -1.725422 -3.178608 0.842407
H -2.728089 -3.529470 1.062457
C -2.285405 -0.810934 1.750684
C -1.940821 0.542066 1.129988
C -3.767073 -1.152658 1.583277
H -4.050154 -1.210551 0.528739
H -4.394455 -0.401136 2.069702
H -3.997476 -2.109912 2.059843
C -2.849793 1.573804 0.909962
C -2.431966 2.790134 0.372578
C -1.099192 2.991812 0.026592
C -0.160240 1.972126 0.218016
C -0.612895 0.783655 0.783379
O 0.331152 -0.182431 1.002077
Pd 2.834384 0.397783 -1.383200
P 2.521353 -1.600844 -0.113091
C 3.446238 -3.054974 -0.740965
C 3.108386 -3.547188 -2.010103
C 3.782109 -4.646234 -2.533970
C 4.807678 -5.254751 -1.807116
C 5.151720 -4.761685 -0.551049
C 4.470314 -3.666971 -0.015043
H 4.744700 -3.299497 0.969015
H 5.948980 -5.228680 0.019750
H 5.336233 -6.108621 -2.220849
H 3.509532 -5.024527 -3.514954
H 2.316938 -3.061150 -2.580032
C 3.194302 -1.347796 1.574371
C 2.697264 -2.033317 2.687587
C 3.251693 -1.820334 3.947774
C 4.313410 -0.929815 4.105011
C 4.816932 -0.248869 2.998282
C 4.254991 -0.452327 1.740353
H 4.636738 0.096814 0.883272
H 5.637226 0.452545 3.114588
H 4.742855 -0.763077 5.088501
H 2.852988 -2.351595 4.806953
H 1.874005 -2.733236 2.576827
P 1.607118 2.120619 -0.256461
C 1.545746 3.677550 -1.226210
C 1.081435 3.582011 -2.545708
C 0.961305 4.726504 -3.327910
C 1.312570 5.973509 -2.806372
C 1.774964 6.070743 -1.496186
C 1.885538 4.926529 -0.703434
H 2.230742 5.020426 0.321545
H 2.045832 7.037796 -1.082705
H 1.225070 6.865091 -3.420368
H 0.597826 4.644110 -4.348035
H 0.816223 2.606720 -2.950440
C 2.461471 2.574156 1.300686
C 1.846631 2.496384 2.551622
C 2.564639 2.805549 3.707700
C 3.893262 3.212093 3.621550
C 4.512790 3.295009 2.373460
C 3.806037 2.962414 1.223137
H 4.301243 3.013164 0.256806
H 5.550255 3.606953 2.297379
H 4.447657 3.458075 4.522376
H 2.077846 2.730985 4.675438
H 0.806685 2.197236 2.638222
H -0.798678 3.939596 -0.405768
H -3.154569 3.584203 0.211457
H -3.897153 1.432108 1.150462
C -1.942709 -0.756480 3.259375
H -2.147888 -1.726158 3.725426
H -0.888374 -0.512446 3.426378
H -2.554748 0.005278 3.754111
H 1.208159 -4.358208 -0.407737
H -1.077272 -5.101677 0.132611
C 3.994713 1.861576 -2.545406
C 3.947919 0.648576 -3.246431
H 3.316877 0.564084 -4.127622
C 4.386613 -0.520300 -2.596294
H 5.232771 -0.483936 -1.910663
H 4.194013 -1.482016 -3.058021
H 3.511203 2.739213 -2.959458
H 4.797373 2.047303 -1.832396
SCF: -3253.957246
ZPE: 0.908710
H: -3252.991469
G: -3253.124744
SP SCF: -3253.450177

Int-C_conf1
C -4.087380 2.597082 -3.935832
C -5.188328 1.900473 -4.579767
C -6.149768 1.248771 -3.851516
N -6.194018 1.158774 -2.493098
C -5.158090 1.771269 -1.876305
C -4.139422 2.465972 -2.489420
H -3.358907 2.901689 -1.869643
H -5.156906 1.691916 -0.788441
H -6.965689 0.752828 -4.378536
H -5.260793 1.910863 -5.665759
C -3.141303 3.309414 -4.634301
C -2.030481 4.115803 -4.011752
C -0.689562 3.416445 -3.848865
C -0.605611 2.146587 -3.262450
C 0.627003 1.527890 -3.063900
C 1.806572 2.163154 -3.458864
C 1.736884 3.419921 -4.057515
C 0.498790 4.036976 -4.247055
H 0.454307 5.019779 -4.711677
H 2.645626 3.921263 -4.380426
H 2.767308 1.678764 -3.308531
H 0.667703 0.544256 -2.603524
H -1.518558 1.637047 -2.965590
H -2.331917 4.470757 -3.014223
H -1.860923 5.027300 -4.600028
H -3.199845 3.312800 -5.722594
C -10.088162 7.378567 3.091582
C -9.736581 6.411128 2.158740
C -8.620691 6.598743 1.332116
C -7.932826 7.801472 1.451429
C -8.257171 8.799904 2.374995
C -9.347211 8.558821 3.206962
H -9.643721 9.294545 3.947088
C -7.421543 10.085793 2.346393
C -5.977392 9.674570 2.046824
C -7.933605 10.974886 1.187249
H -8.978254 11.252083 1.362461
H -7.872091 10.458604 0.224485
H -7.334337 11.889284 1.125750
C -4.829370 10.270810 2.567117
C -3.559580 9.807458 2.213354
C -3.412691 8.704136 1.377572
C -4.544700 8.067487 0.857666
C -5.788265 8.607976 1.169489
O -6.878693 8.005136 0.606147
Pd -6.293113 5.769276 -1.404260
P -8.102902 5.330290 0.105200
C -9.711718 5.099231 -0.755734
C -10.120642 6.128922 -1.613766
C -11.340766 6.052293 -2.276359
C -12.164132 4.938821 -2.095693
C -11.761560 3.912317 -1.245877
C -10.540832 3.992164 -0.572204
H -10.246809 3.185337 0.091347
H -12.396132 3.043175 -1.100720
H -13.113562 4.873255 -2.618529
H -11.646571 6.856919 -2.938377
H -9.477623 6.992716 -1.769954
C -7.864007 3.798776 1.071620
C -7.831985 3.759843 2.467732
C -7.632296 2.547567 3.127434
C -7.459626 1.371461 2.401190
C -7.465605 1.410659 1.006776
C -7.657389 2.618924 0.345243
H -7.643869 2.631140 -0.742298
H -7.310932 0.505743 0.426400
H -7.305218 0.429261 2.918767
H -7.604699 2.528384 4.212866
H -7.955191 4.667287 3.049702
P -4.502930 6.438106 0.021022
C -2.819988 6.430859 -0.709715
C -2.526858 7.400282 -1.677591
C -1.263134 7.460501 -2.255983
C -0.279174 6.544994 -1.880971
C -0.566955 5.572198 -0.928221
C -1.830892 5.515760 -0.339874
H -2.031850 4.756904 0.408975
H 0.190093 4.848322 -0.642145
H 0.705336 6.585057 -2.336356
H -1.048406 8.219961 -3.001774
H -3.284996 8.121901 -1.970998
C -4.393394 5.262895 1.421653
C -4.398062 3.899245 1.103155
C -4.270548 2.940312 2.100624
C -4.172454 3.334843 3.435179
C -4.201718 4.688299 3.762462
C -4.304708 5.653659 2.759787
H -4.313101 6.704478 3.031653
H -4.139176 5.000054 4.800818
H -4.086900 2.587054 4.217989
H -4.267431 1.887184 1.837198
H -4.498930 3.591379 0.066597
H -2.421786 8.318957 1.157124
H -2.678719 10.294487 2.619356
H -4.912501 11.105395 3.255412
C -7.530170 10.868306 3.657135
H -6.939819 11.787288 3.605205
H -8.565174 11.171050 3.837883
H -7.183921 10.279022 4.512338
H -10.320660 5.498579 2.080479
H -10.945624 7.217336 3.737056
C -5.139246 5.914914 -3.234091
C -6.505061 5.870261 -3.581212
H -6.998914 6.765521 -3.953960
C -7.280330 4.803309 -3.104578
H -6.834705 3.816762 -2.973955
H -8.359330 4.831946 -3.214875
H -4.572500 6.816822 -3.442515
H -4.566795 4.991937 -3.177997
SCF: -3065.379517
ZPE: 0.892080
H: -3064.432816
G: -3064.561650
SP SCF: -3064.845543

Int-C_conf2
C 3.023021 -1.565478 -2.765720
C 4.377913 -1.721864 -2.926256
C 5.319508 -2.271397 -1.880960
C 5.896159 -1.208388 -0.961081
C 6.930718 -0.370016 -1.397665
C 7.445291 0.627953 -0.572527
C 6.932868 0.808984 0.713786
C 5.904308 -0.017631 1.160690
C 5.393573 -1.015273 0.329217
H 4.588454 -1.653213 0.684099
H 5.495092 0.115814 2.159227
H 7.330004 1.588271 1.358027
H 8.249603 1.264664 -0.931758
H 7.336457 -0.503562 -2.398428
H 4.819275 -3.030039 -1.264864
H 6.154462 -2.783308 -2.377178
H 4.828248 -1.370453 -3.854554
C 2.176316 -0.990813 -3.799349
C 0.831608 -0.823559 -3.609458
N 0.137007 -1.153612 -2.486258
C 0.904083 -1.720228 -1.520087
C 2.258827 -1.937711 -1.585657
H 2.753596 -2.372823 -0.722086
H 0.369956 -2.004792 -0.612904
H 0.239731 -0.379328 -4.411737
H 2.622131 -0.684599 -4.743965
C -3.472476 -3.964970 0.557564
C -3.609942 -2.766467 -0.135504
C -2.838637 -1.654016 0.218967
C -1.968464 -1.795434 1.296760
C -1.802165 -2.981645 2.012299
C -2.569944 -4.075064 1.614892
H -2.473948 -5.024186 2.131129
C -0.829133 -2.966717 3.191577
C 0.341219 -2.074263 2.781483
C -1.538993 -2.318188 4.405133
H -1.882028 -1.303828 4.178295
H -0.851019 -2.264722 5.255497
H -2.407845 -2.918361 4.695352
C 1.676290 -2.275566 3.133154
C 2.658345 -1.364267 2.748022
C 2.330651 -0.247058 1.984413
C 1.008371 -0.028852 1.594334
C 0.051233 -0.942051 2.023935
O -1.243443 -0.685987 1.647536
Pd -0.922380 1.128246 -1.394749
P -2.873399 -0.063729 -0.696705
C -3.907330 1.051623 0.325267
C -4.285005 0.756625 1.635928
C -5.012634 1.684727 2.382689
C -5.382738 2.903182 1.820288
C -5.015640 3.200157 0.506496
C -4.269583 2.287009 -0.229650
H -3.969863 2.533220 -1.245435
H -5.298700 4.149218 0.060810
H -5.951582 3.621904 2.402651
H -5.291141 1.448178 3.405222
H -4.019011 -0.194499 2.086687
C -3.937280 -0.517920 -2.118746
C -3.313806 -1.159417 -3.197251
C -4.064377 -1.566718 -4.296084
C -5.439567 -1.328247 -4.332666
C -6.062064 -0.690368 -3.261546
C -5.314798 -0.290641 -2.152517
H -5.813930 0.193497 -1.318502
H -7.132077 -0.505612 -3.282817
H -6.023305 -1.639731 -5.193976
H -3.574302 -2.066458 -5.126470
H -2.239013 -1.335455 -3.164502
P 0.449109 1.370118 0.550977
C -0.382324 2.465023 1.764128
C -0.128762 2.385738 3.137870
C -0.782906 3.239831 4.023160
C -1.690057 4.184592 3.544252
C -1.942928 4.271876 2.176431
C -1.296371 3.412000 1.291849
H -1.513260 3.465354 0.228242
H -2.654629 4.998844 1.797395
H -2.202370 4.846665 4.236099
H -0.583495 3.165248 5.088098
H 0.576831 1.655404 3.523112
C 2.045164 2.192032 0.183855
C 2.471189 3.354745 0.827919
C 3.699973 3.928445 0.493202
C 4.508933 3.337905 -0.474732
C 4.087018 2.172659 -1.117563
C 2.858939 1.607339 -0.796110
H 2.537919 0.699958 -1.302126
H 4.716551 1.694577 -1.861813
H 5.468739 3.779685 -0.726298
H 4.023167 4.835142 0.996385
H 1.852798 3.820566 1.589586
H 3.110482 0.438594 1.673539
H 3.691341 -1.532850 3.036995
H 1.960239 -3.145291 3.715912
C -0.370914 -4.374479 3.579288
H 0.139574 -4.877277 2.751729
H 0.308341 -4.333783 4.435371
H -1.224765 -4.985455 3.885079
H -4.304960 -2.707756 -0.965646
H -4.067418 -4.824473 0.265541
C 0.268550 2.559875 -2.516927
C -1.092328 2.799914 -2.786666
H -1.577599 3.684308 -2.379341
C -1.868914 1.732728 -3.270770
H -1.433371 0.998337 -3.944132
H -2.948287 1.837947 -3.323514
H 0.842597 3.314829 -1.989133
H 0.843846 1.873543 -3.137160
SCF: -3065.390450
ZPE: 0.892868
H: -3064.443221
G: -3064.571205
SP SCF: -3064.859010

Int-D_conf1
C 0.836583 4.203241 2.124001
C 0.693897 3.741278 0.819551
C 1.035098 2.425347 0.489928
C 1.535495 1.622026 1.518467
C 1.674634 2.046964 2.839103
C 1.309000 3.361112 3.126710
H 1.382236 3.736166 4.141421
C 2.258274 1.062278 3.852942
C 1.870619 1.425932 5.288297
H 2.257096 2.414712 5.551438
H 0.783265 1.421978 5.412040
H 2.314976 0.719972 5.995858
C 1.771234 -0.334187 3.466816
C 1.486887 -1.364232 4.364576
C 1.135119 -2.632465 3.905946
C 1.057290 -2.897422 2.539528
C 1.313838 -1.886523 1.611207
C 1.659510 -0.629399 2.110552
O 1.906207 0.345083 1.177170
P 1.057039 -2.035486 -0.201089
C 2.757330 -1.889300 -0.885081
C 3.911712 -1.863448 -0.098268
C 5.172288 -1.850053 -0.693869
C 5.290617 -1.873668 -2.081701
C 4.142627 -1.891137 -2.873518
C 2.884637 -1.887222 -2.277989
H 1.993096 -1.887765 -2.901167
H 4.226646 -1.892784 -3.956138
H 6.272419 -1.865916 -2.546084
H 6.061348 -1.827711 -0.070083
H 3.836123 -1.868159 0.985087
C 0.813189 -3.857568 -0.371251
C -0.488663 -4.345935 -0.497409
C -0.738917 -5.714902 -0.585440
C 0.322669 -6.616535 -0.556586
C 1.629637 -6.141043 -0.438558
C 1.873845 -4.772773 -0.345596
H 2.896866 -4.421413 -0.250223
H 2.462656 -6.837737 -0.417600
H 0.135316 -7.683850 -0.629306
H -1.759983 -6.072747 -0.681627
H -1.312355 -3.643980 -0.527926
Pd -0.589723 -0.383853 -0.883254
P 0.681130 1.643149 -1.140672
C 2.313003 1.580862 -1.987056
C 3.534639 1.703247 -1.323560
C 4.732149 1.672886 -2.039545
C 4.720620 1.528529 -3.424116
C 3.503634 1.390428 -4.092851
C 2.310934 1.404619 -3.377734
H 1.368568 1.299661 -3.910131
H 3.483512 1.266888 -5.171860
H 5.653858 1.512328 -3.979430
H 5.674822 1.767039 -1.508371
H 3.566299 1.835631 -0.246827
C -0.084049 3.044135 -2.066325
C -1.455402 3.025949 -2.327909
C -2.070015 4.080579 -3.004143
C -1.312004 5.166678 -3.433638
C 0.061563 5.193998 -3.183809
C 0.670124 4.141730 -2.506132
H 1.739092 4.178041 -2.315497
H 0.659655 6.037527 -3.515974
H -1.785755 5.987962 -3.963314
H -3.138175 4.046863 -3.197630
H -2.047652 2.176532 -2.009403
C -1.245237 -0.573377 -3.386742
C -1.879902 -1.591188 -2.768869
C -3.291617 -1.518369 -2.253324
C -3.386912 -1.903934 -0.771526
C -2.509607 -1.032774 0.117072
C -2.516880 0.390728 -0.034493
C -2.226326 1.200765 1.071258
N -1.923058 0.690413 2.262845
C -1.853120 -0.666571 2.441419
C -2.144151 -1.515487 1.416568
H -2.118129 -2.585151 1.601176
H -1.572469 -0.983802 3.436307
C -1.790691 1.637034 3.494906
O -1.454398 1.037422 4.515964
O -2.080772 2.801859 3.231317
H -2.301874 2.281094 1.037495
H -3.041882 0.861562 -0.856063
H -3.033748 -2.936000 -0.679241
C -4.851414 -1.932721 -0.244343
C -5.517955 -0.582894 -0.140194
C -5.484025 0.134776 1.061231
C -6.027426 1.414851 1.148943
C -6.622514 1.999439 0.030989
C -6.680698 1.288323 -1.167838
C -6.135763 0.006987 -1.248860
H -6.188602 -0.541699 -2.186137
H -7.153171 1.730219 -2.040727
H -7.043243 2.998701 0.095454
H -5.978610 1.958015 2.088425
H -5.006901 -0.308005 1.932874
H -4.845289 -2.408384 0.742182
H -5.427371 -2.582830 -0.912790
H -3.691481 -0.512549 -2.422449
H -3.924021 -2.208402 -2.826534
H -1.388798 -2.561611 -2.704999
H -1.740012 0.379444 -3.560444
H -0.262324 -0.706790 -3.829365
H 0.777136 -3.889742 2.200334
H 0.920590 -3.422104 4.619618
H 1.539148 -1.183834 5.432701
C 3.800806 1.086167 3.720539
H 4.181722 2.084639 3.960807
H 4.122674 0.832089 2.705230
H 4.248623 0.365223 4.413078
H 0.287526 4.404859 0.064363
H 0.549943 5.221472 2.366904
SCF: -3253.989376
ZPE: 0.912255
H: -3253.021431
G: -3253.151104
SP SCF: -3253.483140

Int-D_conf15
C -3.692905 -1.392520 -3.247076
C -3.275657 -1.725899 -1.961559
C -2.691788 -0.762623 -1.135176
C -2.565650 0.532414 -1.640103
C -3.001245 0.907197 -2.911312
C -3.556701 -0.087181 -3.717275
H -3.895817 0.152046 -4.719487
C -2.881529 2.381670 -3.290967
C -4.042886 3.153374 -2.617138
H -3.962888 4.221801 -2.843887
H -5.003691 2.786638 -2.993498
H -4.031990 3.032142 -1.529677
C -1.562609 2.884185 -2.705290
C -0.748402 3.846870 -3.299111
C 0.399649 4.304500 -2.654698
C 0.769851 3.776954 -1.422901
C 0.004805 2.775446 -0.812398
C -1.169349 2.381898 -1.461401
O -1.966798 1.463475 -0.827287
P 0.484901 1.959840 0.765663
C 2.262283 2.396866 0.926381
C 2.790181 2.815037 2.152123
C 4.169671 2.942047 2.322931
C 5.038521 2.659466 1.271072
C 4.520074 2.246373 0.042734
C 3.145839 2.106214 -0.123345
H 2.761997 1.762148 -1.079317
H 5.186364 2.020581 -0.784968
H 6.111387 2.759231 1.406054
H 4.561887 3.264377 3.282985
H 2.129867 3.037569 2.985423
C -0.345592 2.997532 2.034316
C -0.662809 4.342649 1.813154
C -1.281534 5.091613 2.811784
C -1.584653 4.505579 4.041355
C -1.267544 3.167689 4.268382
C -0.655111 2.414016 3.268172
H -0.417667 1.368212 3.454280
H -1.503371 2.701609 5.220658
H -2.070428 5.090124 4.817297
H -1.529049 6.133054 2.627902
H -0.435278 4.810725 0.859825
Pd 0.107561 -0.305564 1.220819
P -2.036347 -1.108681 0.541206
C -2.130680 -2.935482 0.621335
C -3.151061 -3.640694 1.265124
C -3.125192 -5.034854 1.299050
C -2.088641 -5.733915 0.681474
C -1.067234 -5.034725 0.037371
C -1.081458 -3.643198 0.020104
H -0.272430 -3.101247 -0.463741
H -0.250612 -5.570173 -0.438690
H -2.072887 -6.819616 0.708729
H -3.917905 -5.574252 1.809124
H -3.964718 -3.107461 1.748009
C -3.390829 -0.520823 1.628998
C -3.084618 -0.253692 2.968073
C -4.074696 0.185759 3.843956
C -5.378022 0.375634 3.386711
C -5.689121 0.118057 2.051696
C -4.701359 -0.330987 1.176744
H -4.956452 -0.529817 0.139733
H -6.702068 0.266565 1.688880
H -6.148310 0.727523 4.066830
H -3.821593 0.388319 4.880412
H -2.068765 -0.382269 3.332577
C 7.572741 -1.267149 -1.091556
C 6.743627 -2.161547 -0.551122
C 5.619189 -1.834597 0.388406
C 4.244064 -2.308583 -0.129968
C 3.160810 -2.139206 0.911715
C 2.335916 -3.243698 1.287359
C 1.322338 -3.076027 2.174680
N 1.029945 -1.834992 2.704166
C 1.829576 -0.734077 2.401083
C 2.928887 -0.931090 1.514561
H 3.558996 -0.071588 1.317013
H 1.844973 0.045320 3.156082
C 0.104473 -1.775028 3.957210
O 0.089192 -0.659474 4.485664
O -0.444781 -2.845797 4.202996
H 0.669053 -3.867696 2.514699
H 2.515765 -4.230389 0.873618
H 4.322074 -3.378770 -0.359172
C 3.862043 -1.561791 -1.427100
C 2.541084 -1.978062 -2.032365
C 1.501968 -1.056986 -2.199328
C 0.304175 -1.427584 -2.811240
C 0.115862 -2.737478 -3.244892
C 1.135373 -3.673216 -3.063948
C 2.336201 -3.293929 -2.467106
H 3.130188 -4.027583 -2.346322
H 0.996873 -4.699325 -3.393390
H -0.821180 -3.029370 -3.709841
H -0.484606 -0.692976 -2.941419
H 1.632768 -0.033851 -1.856662
H 4.659406 -1.724553 -2.162040
H 3.842132 -0.486682 -1.214610
H 5.805932 -2.323921 1.352932
H 5.594804 -0.753632 0.572801
H 6.871114 -3.218199 -0.793275
H 7.476232 -0.203670 -0.878175
H 8.375895 -1.567067 -1.759794
H 1.670459 4.138775 -0.938828
H 1.010289 5.073254 -3.117763
H -1.013209 4.261338 -4.265777
C -2.960607 2.595962 -4.804517
H -3.917805 2.236299 -5.191874
H -2.153662 2.077020 -5.332030
H -2.907308 3.661023 -5.046218
H -3.386747 -2.745870 -1.607885
H -4.131417 -2.152885 -3.885913
SCF: -3253.980085
ZPE: 0.910085
H: -3253.013122
G: -3253.146148
SP SCF: -3253.474444

Int-D_conf4
C -5.283834 -0.838200 -2.209749
C -4.200257 -1.536030 -1.682626
C -3.270110 -0.890868 -0.861369
C -3.489791 0.462250 -0.589230
C -4.570702 1.189120 -1.089766
C -5.465415 0.512619 -1.919401
H -6.319278 1.033395 -2.339416
C -4.726730 2.640600 -0.636167
C -5.378140 2.639647 0.768791
H -5.476243 3.666273 1.137339
H -6.374615 2.188005 0.719532
H -4.779449 2.073320 1.489324
C -3.323609 3.232287 -0.510425
C -2.993620 4.566588 -0.752589
C -1.703601 5.035758 -0.511461
C -0.712331 4.174985 -0.045399
C -0.993023 2.823602 0.172009
C -2.305406 2.397926 -0.051674
O -2.575912 1.081912 0.224434
P 0.224146 1.533612 0.655744
C 1.798752 2.474735 0.823293
C 2.361212 3.071716 -0.314319
C 3.621748 3.658635 -0.266742
C 4.356576 3.642394 0.919355
C 3.808329 3.055114 2.056542
C 2.536962 2.481367 2.011621
H 2.135106 2.024789 2.910122
H 4.369629 3.037348 2.986134
H 5.347983 4.084086 0.954652
H 4.034970 4.118152 -1.160050
H 1.811560 3.080401 -1.250510
C -0.208190 1.216374 2.410773
C -0.898802 2.141822 3.199577
C -1.130529 1.880641 4.548825
C -0.655865 0.702608 5.125252
C 0.034980 -0.222092 4.344210
C 0.243127 0.027182 2.990293
H 0.764669 -0.705158 2.379917
H 0.400014 -1.146032 4.781324
H -0.830428 0.502614 6.178505
H -1.675357 2.602077 5.151065
H -1.256218 3.071638 2.765730
Pd 0.304989 -0.447743 -0.714436
P -1.713341 -1.672228 -0.260613
C -1.837092 -3.345144 -1.023273
C -1.182797 -3.568862 -2.238859
C -1.265409 -4.804540 -2.878362
C -2.000651 -5.838547 -2.301458
C -2.658882 -5.626210 -1.090201
C -2.582011 -4.387194 -0.457497
H -3.106708 -4.236908 0.481055
H -3.234693 -6.426496 -0.634768
H -2.059123 -6.806097 -2.791182
H -0.749518 -4.960058 -3.821417
H -0.603712 -2.768863 -2.688710
C -2.042463 -2.049166 1.506550
C -1.184287 -2.958088 2.141007
C -1.366812 -3.287090 3.479485
C -2.394842 -2.692315 4.212087
C -3.236605 -1.770786 3.594798
C -3.066139 -1.454143 2.246303
H -3.744513 -0.745719 1.781739
H -4.033760 -1.295420 4.158998
H -2.531633 -2.939616 5.260808
H -0.696382 -3.999301 3.951308
H -0.374517 -3.430573 1.590640
C -0.642430 0.237271 -3.135543
C 0.550088 0.844503 -2.999805
C 1.859946 0.271004 -3.461868
C 2.921944 0.187867 -2.354367
C 2.483804 -0.643080 -1.153889
C 1.854393 -1.920818 -1.331269
C 1.845980 -2.833095 -0.269061
N 2.469414 -2.590036 0.886263
C 3.150442 -1.411091 1.072199
C 3.172219 -0.463598 0.098776
H 3.734884 0.447089 0.271414
H 3.644474 -1.311055 2.028091
C 2.408755 -3.635634 2.022413
O 1.796312 -4.657852 1.703485
O 2.982413 -3.265620 3.046894
H 1.368056 -3.802710 -0.343107
H 1.564366 -2.289950 -2.308568
H 3.111224 1.202733 -1.986523
C 4.245472 -0.353890 -2.952742
C 5.414358 -0.308122 -2.000843
C 5.924378 -1.473110 -1.422172
C 6.960211 -1.414951 -0.489893
C 7.500936 -0.184086 -0.121485
C 7.006211 0.986189 -0.699984
C 5.975060 0.920842 -1.633790
H 5.588744 1.837069 -2.074750
H 7.423181 1.949972 -0.421236
H 8.302569 -0.135698 0.609775
H 7.336896 -2.331104 -0.044010
H 5.491977 -2.434753 -1.688667
H 4.476230 0.246936 -3.840557
H 4.082702 -1.383163 -3.293897
H 1.695952 -0.718610 -3.906123
H 2.262722 0.906606 -4.260852
H 0.576547 1.856296 -2.598886
H -0.732830 -0.736561 -3.611031
H -1.565914 0.733289 -2.849059
H 0.283653 4.560975 0.142634
H -1.469589 6.081674 -0.684397
H -3.747828 5.256301 -1.115992
C -5.608101 3.450730 -1.590390
H -5.725710 4.475594 -1.227963
H -6.612272 3.021814 -1.644288
H -5.188384 3.483939 -2.601030
H -4.072245 -2.584568 -1.929351
H -5.992613 -1.351643 -2.852019
SCF: -3253.993466
ZPE: 0.910772
H: -3253.026338
G: -3253.158084
SP SCF: -3253.487154

Int-E_conf1
C -1.870170 2.740485 3.176470
C -1.675305 2.730811 1.796407
C -0.573921 2.071740 1.245042
C 0.313107 1.456781 2.128578
C 0.160126 1.458771 3.513182
C -0.963595 2.107516 4.026758
H -1.139559 2.128095 5.097019
C 1.254274 0.789180 4.343450
C 1.714646 -0.443281 3.564689
C 2.446213 1.770799 4.456876
H 2.135921 2.678368 4.985853
H 2.823860 2.061210 3.471247
H 3.265151 1.303582 5.014238
C 2.103536 -1.651965 4.138776
C 2.552334 -2.703418 3.341760
C 2.602018 -2.571102 1.957018
C 2.197936 -1.382434 1.338730
C 1.773220 -0.348374 2.174446
O 1.388376 0.818797 1.564816
Pd -0.003756 -0.376052 -1.389591
P -0.270298 1.835766 -0.553237
C 1.228307 2.864435 -0.842045
C 1.826657 2.784442 -2.104343
C 2.923855 3.580685 -2.419973
C 3.452180 4.452396 -1.468516
C 2.873760 4.525387 -0.202969
C 1.763682 3.740300 0.106402
H 1.314348 3.822109 1.091813
H 3.282460 5.197280 0.546480
H 4.316787 5.063901 -1.710071
H 3.376793 3.507260 -3.404092
H 1.432235 2.090336 -2.842958
C -1.580212 2.926318 -1.260855
C -1.405792 4.295009 -1.495811
C -2.452990 5.063975 -2.002777
C -3.690083 4.478904 -2.272535
C -3.874485 3.116704 -2.038693
C -2.821755 2.349845 -1.544774
H -2.963145 1.289457 -1.372665
H -4.832260 2.647647 -2.245506
H -4.503885 5.081115 -2.665869
H -2.300973 6.124014 -2.184761
H -0.452049 4.769298 -1.284614
P 2.045324 -1.173533 -0.489800
C 3.489971 -0.132194 -0.953025
C 4.234068 0.618237 -0.041454
C 5.305198 1.399674 -0.477811
C 5.652603 1.426973 -1.825667
C 4.908273 0.685703 -2.744602
C 3.825863 -0.073196 -2.313095
H 3.243926 -0.636738 -3.038679
H 5.163048 0.708872 -3.800301
H 6.490596 2.030873 -2.161469
H 5.869517 1.983880 0.243362
H 3.993003 0.596561 1.016559
C 2.615322 -2.839908 -1.041960
C 3.961870 -3.174924 -1.227296
C 4.325283 -4.469485 -1.595596
C 3.349264 -5.450334 -1.770822
C 2.006363 -5.129536 -1.576860
C 1.645496 -3.831475 -1.220872
H 0.600145 -3.582631 -1.069983
H 1.238627 -5.886736 -1.708011
H 3.634419 -6.458615 -2.056756
H 5.374178 -4.712290 -1.739732
H 4.736534 -2.428727 -1.078879
C -1.563340 -0.835270 -2.857393
C -0.470608 -1.695646 -3.029878
H -0.534423 -2.744353 -2.749856
H 0.276318 -1.480916 -3.793605
H -1.637536 0.035381 -3.510279
C -2.915863 -1.304959 -2.344934
H -3.456987 -0.467156 -1.896962
H -3.516663 -1.594319 -3.220047
H 2.937300 -3.409984 1.357159
H 2.858043 -3.637517 3.803121
H 2.059951 -1.783496 5.214441
C 0.770591 0.430847 5.750378
H 0.463205 1.330241 6.291323
H -0.072904 -0.266169 5.721285
H 1.579147 -0.022736 6.330642
H -2.394206 3.223265 1.149385
H -2.736417 3.244971 3.593446
N -1.313223 -1.913610 2.603625
C -2.025708 -0.956932 1.998126
C -2.528821 -1.064636 0.705966
C -2.312247 -2.237230 -0.022412
C -1.563363 -3.233497 0.610109
C -1.084314 -3.028404 1.899365
H -0.493970 -3.797342 2.392450
H -1.358192 -4.172928 0.103670
C -2.934433 -2.497033 -1.378789
H -2.370786 -3.317510 -1.837304
C -4.379856 -3.055052 -1.201593
H -4.319156 -3.973935 -0.608593
H -4.746181 -3.334785 -2.195850
C -5.355738 -2.104523 -0.550134
C -6.038033 -1.140462 -1.302595
C -6.889467 -0.221913 -0.690206
C -7.081929 -0.258362 0.691048
C -6.424517 -1.225937 1.450202
C -5.571560 -2.139361 0.832362
H -5.055010 -2.884072 1.432976
H -6.572514 -1.268683 2.525603
H -7.744673 0.456512 1.170110
H -7.405763 0.520272 -1.292851
H -5.898672 -1.107323 -2.380231
H -3.109205 -0.245083 0.297248
H -2.211316 -0.055790 2.577442
SCF: -3065.463918
ZPE: 0.897342
H: -3064.513560
G: -3064.638768
SP SCF: -3064.927739

Int-E_conf2
C 0.792670 3.307058 3.165047
C 0.501604 3.255828 1.804579
C -0.261694 2.208512 1.276301
C -0.720831 1.244594 2.177317
C -0.457412 1.268318 3.547804
C 0.321627 2.319507 4.028768
H 0.566171 2.378870 5.083794
C -1.077287 0.171034 4.415332
C -1.063557 -1.117801 3.594875
C -0.338333 0.007759 5.745785
H -0.812855 -0.765928 6.355746
H -0.380284 0.933116 6.326923
H 0.710851 -0.263222 5.590669
C -0.884816 -2.401608 4.111198
C -0.944085 -3.518422 3.278905
C -1.163112 -3.372742 1.910511
C -1.320273 -2.100993 1.355290
C -1.279889 -1.011481 2.224413
O -1.471142 0.223511 1.656809
Pd -0.022557 -0.161035 -1.476747
P -0.542809 1.954321 -0.532680
C 0.363373 3.415952 -1.203348
C 1.689598 3.235813 -1.608759
C 2.450940 4.310663 -2.064841
C 1.886447 5.583166 -2.134423
C 0.562798 5.774432 -1.737030
C -0.191579 4.699988 -1.269290
H -1.215357 4.869033 -0.948693
H 0.116844 6.763639 -1.785724
H 2.473314 6.422020 -2.497129
H 3.480677 4.150791 -2.371344
H 2.129081 2.244477 -1.565607
C -2.290700 2.461989 -0.800067
C -3.236869 2.565292 0.220999
C -4.554007 2.923640 -0.070960
C -4.934398 3.197301 -1.381772
C -3.995844 3.089056 -2.409072
C -2.689742 2.708018 -2.121554
H -1.969314 2.612964 -2.930617
H -4.284420 3.289453 -3.436943
H -5.958421 3.482126 -1.605167
H -5.280152 2.992344 0.733901
H -2.958591 2.374444 1.252452
P -1.470360 -1.731605 -0.438423
C -1.542203 -3.435905 -1.135632
C -0.325103 -4.063972 -1.421257
C -0.290240 -5.360543 -1.927643
C -1.482128 -6.043342 -2.172537
C -2.700897 -5.423742 -1.900022
C -2.732049 -4.129361 -1.379947
H -3.689988 -3.664490 -1.167084
H -3.632933 -5.948309 -2.090038
H -1.460999 -7.051263 -2.576581
H 0.664778 -5.833450 -2.138442
H 0.600025 -3.527436 -1.237356
C -3.227316 -1.201995 -0.584391
C -4.162993 -1.336761 0.445279
C -5.486070 -0.939918 0.254616
C -5.890221 -0.417050 -0.972115
C -4.964021 -0.284222 -2.005796
C -3.639400 -0.663802 -1.808602
H -2.915084 -0.535048 -2.609680
H -5.268284 0.132901 -2.961031
H -6.920438 -0.106185 -1.120155
H -6.200464 -1.042665 1.066539
H -3.866369 -1.754770 1.402918
C 1.107261 -1.165866 -3.047301
C 1.023558 0.207532 -3.322289
H 1.896416 0.850410 -3.232242
H 0.267977 0.576233 -4.015780
H 0.420955 -1.838128 -3.563435
C 2.419649 -1.828022 -2.648964
H 2.229680 -2.832667 -2.256245
H 3.004568 -1.982417 -3.567521
H -1.194028 -4.251397 1.274094
H -0.811976 -4.510536 3.699920
H -0.700223 -2.541726 5.170839
C -2.550820 0.551725 4.699284
H -3.032246 -0.230955 5.295383
H -2.595192 1.493511 5.256835
H -3.119813 0.674234 3.772008
H 0.892020 4.028344 1.151405
H 1.395407 4.121655 3.554628
N 2.181023 -1.129898 2.526864
C 2.238666 -2.251341 1.797595
C 2.550367 -2.278085 0.442827
C 2.821441 -1.081556 -0.226608
C 2.748084 0.087485 0.532363
C 2.430160 0.016272 1.884365
H 2.375971 0.925259 2.479400
H 2.960739 1.052644 0.084138
C 3.308095 -1.053285 -1.661637
H 3.342119 -0.002736 -1.971527
C 4.765431 -1.580308 -1.719379
H 5.100452 -1.528690 -2.761532
H 4.779374 -2.636262 -1.424487
C 5.703301 -0.784255 -0.845210
C 6.099068 -1.251022 0.411810
C 6.905586 -0.473531 1.242838
C 7.329506 0.787790 0.826668
C 6.945460 1.262952 -0.428049
C 6.139746 0.481521 -1.254067
H 5.840426 0.859251 -2.229629
H 7.274514 2.242649 -0.763390
H 7.955507 1.395689 1.473417
H 7.198186 -0.852265 2.218129
H 5.761366 -2.228218 0.748453
H 2.601841 -3.233738 -0.070434
H 2.029084 -3.178124 2.326469
SCF: -3065.464512
ZPE: 0.896982
H: -3064.514429
G: -3064.639728
SP SCF: -3064.928871

Int-F'_conf1
C -5.008277 0.271572 -1.500091
C -4.538117 -0.762984 -2.301396
C -3.269658 -1.320097 -2.113251
C -2.494462 -0.798606 -1.077132
C -2.940692 0.233821 -0.240824
C -4.208182 0.766462 -0.474407
H -5.995019 0.689316 -1.673361
H -5.174274 -1.146530 -3.093434
H -4.572292 1.575490 0.151964
O -1.231434 -1.253232 -0.817106
C -0.659854 -2.213787 -1.594239
C 0.639423 -2.587598 -1.208506
C -1.323939 -2.795020 -2.672825
C 1.300224 -3.543060 -1.979197
C -0.619013 -3.745268 -3.418597
C 0.679309 -4.110959 -3.088991
H 2.307829 -3.846962 -1.721649
H -1.101381 -4.218805 -4.268021
H 1.206069 -4.849740 -3.684349
C -2.779668 -2.465751 -2.996456
C -2.919438 -2.083899 -4.483985
H -2.597090 -2.906340 -5.129000
H -3.962462 -1.869446 -4.732563
H -2.319678 -1.201159 -4.724844
C -3.639976 -3.716216 -2.708117
H -4.693810 -3.515143 -2.926826
H -3.317861 -4.557390 -3.330565
H -3.556291 -4.012166 -1.657304
P 1.425703 -1.750392 0.226347
P -1.817168 0.836779 1.088767
C 2.921356 -2.759348 0.573957
C 2.843067 -4.144855 0.773573
C 4.154166 -2.121918 0.718421
C 3.988092 -4.878450 1.064604
H 1.885403 -4.653191 0.697276
C 5.302215 -2.855057 1.018926
H 4.218988 -1.045370 0.590686
C 5.222776 -4.234913 1.180328
H 3.918548 -5.953103 1.204096
H 6.256949 -2.347036 1.115748
H 6.117633 -4.809413 1.400026
C 0.401899 -2.269209 1.674585
C 0.912052 -1.974810 2.945630
C -0.788397 -2.992565 1.580965
C 0.236682 -2.373803 4.093922
H 1.855658 -1.446311 3.043555
C -1.470215 -3.386105 2.732760
H -1.197177 -3.271305 0.616862
C -0.964153 -3.075848 3.990843
H 0.648181 -2.133769 5.069879
H -2.400900 -3.937718 2.638953
H -1.499368 -3.378393 4.885647
C -2.608281 0.171487 2.611382
C -3.740593 -0.648681 2.627097
C -1.993275 0.491264 3.830734
C -4.255643 -1.124379 3.833540
H -4.228454 -0.923357 1.696744
C -2.516575 0.032093 5.035915
H -1.099088 1.110994 3.838888
C -3.651486 -0.779677 5.040409
H -5.133867 -1.763866 3.826486
H -2.030215 0.296828 5.970407
H -4.055827 -1.147373 5.979006
C -2.301826 2.614187 1.138946
C -3.190817 3.185044 2.055623
C -1.696891 3.439276 0.180447
C -3.470395 4.551630 2.012133
H -3.673344 2.566238 2.806813
C -1.986517 4.800052 0.127218
H -0.998952 3.011026 -0.536411
C -2.871887 5.361670 1.048035
H -4.162181 4.981226 2.731201
H -1.514701 5.421595 -0.628606
H -3.092045 6.424759 1.015596
Pd 2.009605 0.538645 -0.003949
C 2.678438 2.562653 0.374258
H 2.481341 3.344190 -0.353997
H 3.728656 2.378133 0.600807
C 1.683792 2.243148 1.320374
H 0.700770 2.698463 1.223970
C 1.828289 1.105411 2.128193
H 2.813991 0.802348 2.480349
H 0.982467 0.769749 2.719629
N 6.097782 -1.803458 -2.108524
C 6.168470 -0.542162 -1.618346
C 4.947202 -2.240128 -2.661459
C 5.082635 0.287928 -1.633083
H 7.134582 -0.260737 -1.219356
C 3.829097 -1.448192 -2.715808
H 4.981582 -3.250958 -3.048473
C 3.830792 -0.146974 -2.151871
H 5.180073 1.287183 -1.221424
H 2.936745 -1.849047 -3.178471
C 1.566057 0.440453 -3.121993
H 1.106112 -0.540518 -2.965799
H 2.014487 0.405204 -4.129720
C 0.465530 1.479754 -3.147079
C -0.866578 1.074265 -3.250121
C 0.731698 2.855166 -3.131721
C -1.908702 1.996223 -3.328823
H -1.086147 0.012553 -3.276375
C -0.303349 3.785382 -3.219899
H 1.756636 3.210139 -3.063989
C -1.629210 3.360782 -3.317645
H -2.934943 1.643547 -3.390029
H -0.072683 4.847316 -3.210115
H -2.434968 4.086971 -3.372433
C 2.663297 0.703947 -2.095105
H 2.981156 1.747184 -2.123952
C 7.325381 -2.745486 -2.015077
O 7.108909 -3.858374 -2.493936
O 8.295192 -2.209438 -1.478091
SCF: -3253.949556
ZPE: 0.909089
H: -3252.983851
G: -3253.115848
SP SCF: -3253.446031

Int-F_conf1
C 0.894005 -5.076624 -0.085571
C 2.177680 -4.693124 -0.471649
C 2.582988 -3.362867 -0.374566
C 1.640287 -2.448130 0.092271
C 0.330235 -2.779469 0.428403
C -0.022832 -4.131554 0.364441
H 0.603709 -6.121048 -0.141564
H 2.867917 -5.448057 -0.832803
H -1.018383 -4.452506 0.647645
O 1.979181 -1.134128 0.259055
C 2.867884 -0.613406 -0.639839
C 2.717313 0.743574 -0.917646
C 3.865719 -1.418927 -1.184868
C 3.631732 1.317136 -1.804865
C 4.732544 -0.819210 -2.097895
C 4.616764 0.535395 -2.403714
H 3.585934 2.374212 -2.036393
H 5.520347 -1.401011 -2.564264
H 5.310761 0.991656 -3.102500
C 3.991173 -2.854108 -0.675529
C 4.724417 -3.754618 -1.673538
H 5.736123 -3.383080 -1.856537
H 4.832218 -4.765956 -1.272938
H 4.195527 -3.812500 -2.630279
C 4.786556 -2.820315 0.652633
H 4.873234 -3.831740 1.063632
H 5.793069 -2.425210 0.478445
H 4.294121 -2.187953 1.398594
P 1.364719 1.633331 -0.033734
P -0.826943 -1.397842 0.803947
C 1.676532 3.361718 -0.596549
C 2.056551 4.382396 0.279369
C 1.498582 3.673444 -1.952623
C 2.250758 5.683002 -0.188338
H 2.205151 4.176551 1.334157
C 1.702091 4.966946 -2.421694
H 1.206140 2.896625 -2.654330
C 2.075501 5.979257 -1.537194
H 2.542198 6.462930 0.508804
H 1.562197 5.184210 -3.476373
H 2.227138 6.991797 -1.899057
C 2.004014 1.681662 1.687464
C 1.132598 2.089570 2.700576
C 3.336626 1.400272 2.007462
C 1.586850 2.229241 4.009817
H 0.093976 2.302827 2.468957
C 3.785975 1.521920 3.320334
H 4.033792 1.089288 1.235455
C 2.913389 1.942201 4.323584
H 0.898502 2.549900 4.785395
H 4.821237 1.294050 3.556650
H 3.265480 2.041096 5.346207
C -0.538152 -0.987976 2.565995
C 0.385104 -1.654664 3.372688
C -1.339698 0.015981 3.126044
C 0.494062 -1.332592 4.726069
H 1.018794 -2.431864 2.955254
C -1.254317 0.312059 4.481976
H -2.042269 0.562853 2.499763
C -0.335016 -0.362738 5.285405
H 1.222745 -1.849424 5.343521
H -1.892365 1.081727 4.906218
H -0.256008 -0.123085 6.341740
C -2.419286 -2.330407 0.897478
C -3.194527 -2.421018 2.057780
C -2.894440 -2.944746 -0.270503
C -4.426861 -3.075917 2.040583
H -2.852723 -1.979881 2.987113
C -4.121577 -3.599332 -0.289014
H -2.289919 -2.928979 -1.172191
C -4.900029 -3.656817 0.867208
H -5.015195 -3.127967 2.951898
H -4.468793 -4.063226 -1.207512
H -5.862721 -4.158901 0.854288
Pd -0.915483 0.786219 -0.437845
C -2.821644 3.678840 0.977868
H -2.835261 4.164528 1.950424
H -3.784119 3.438045 0.532512
C -1.663964 3.411577 0.348747
H -0.741049 3.708899 0.849751
C -1.509700 2.730713 -0.953070
H -2.438811 2.733996 -1.518895
H -0.731133 3.187162 -1.561924
N -6.221426 2.095962 -0.134554
C -5.597444 1.170129 0.629846
C -5.742465 2.389169 -1.357820
C -4.468077 0.533203 0.190243
H -6.054432 0.985593 1.593554
C -4.614104 1.776549 -1.852107
H -6.307955 3.131441 -1.907044
C -3.912099 0.823745 -1.078829
H -3.993214 -0.193536 0.838749
H -4.279938 2.045869 -2.847067
C -2.363303 0.227268 -3.016104
H -2.138922 1.253871 -3.327230
H -3.237376 -0.098607 -3.601150
C -1.188980 -0.668809 -3.340663
C 0.109851 -0.158031 -3.430740
C -1.367054 -2.050541 -3.465823
C 1.203533 -1.003944 -3.607050
H 0.265164 0.914365 -3.344962
C -0.276660 -2.904499 -3.630374
H -2.373066 -2.462416 -3.420250
C 1.015190 -2.382833 -3.693033
H 2.205008 -0.586323 -3.662337
H -0.436000 -3.976603 -3.706377
H 1.867739 -3.045081 -3.812898
C -2.688684 0.163659 -1.518399
H -2.748458 -0.875050 -1.207967
C -7.501796 2.817264 0.400060
O -7.810611 2.444991 1.529874
O -7.959447 3.621011 -0.409067
SCF: -3253.943190
ZPE: 0.910137
H: -3252.976493
G: -3253.108547
SP SCF: -3253.440450

Int-G'_conf1
C -5.016507 0.256108 -1.511029
C -4.536906 -0.775607 -2.310693
C -3.268647 -1.329796 -2.112836
C -2.504179 -0.808367 -1.068596
C -2.959332 0.222076 -0.234627
C -4.226213 0.751658 -0.477869
H -6.002748 0.671811 -1.692058
H -5.165207 -1.158723 -3.109318
H -4.597511 1.559355 0.146036
O -1.243275 -1.259733 -0.798225
C -0.662246 -2.218416 -1.570628
C 0.634702 -2.586509 -1.173363
C -1.314755 -2.801288 -2.654840
C 1.306146 -3.539891 -1.936550
C -0.600379 -3.751104 -3.392595
C 0.695957 -4.112483 -3.050415
H 2.314531 -3.835254 -1.670766
H -1.073328 -4.226070 -4.246565
H 1.230661 -4.849333 -3.641168
C -2.765789 -2.469722 -2.996765
C -2.881816 -2.075222 -4.483140
H -2.550511 -2.892903 -5.129833
H -3.920250 -1.855616 -4.746265
H -2.276130 -1.192031 -4.706718
C -3.630677 -3.721850 -2.731761
H -4.681146 -3.519396 -2.965000
H -3.299290 -4.558303 -3.355754
H -3.563120 -4.026171 -1.682176
P 1.410304 -1.739915 0.262144
P -1.843061 0.826955 1.101515
C 2.891603 -2.768254 0.621337
C 2.779529 -4.139699 0.890613
C 4.145497 -2.161451 0.697690
C 3.908534 -4.890417 1.199287
H 1.806445 -4.622882 0.858395
C 5.279052 -2.912386 1.011158
H 4.238193 -1.096941 0.505357
C 5.162915 -4.277400 1.256346
H 3.811098 -5.953327 1.399800
H 6.249681 -2.427793 1.056021
H 6.044316 -4.864915 1.496122
C 0.370250 -2.256519 1.701693
C 0.858595 -1.945285 2.977033
C -0.810481 -2.994299 1.598749
C 0.171921 -2.341938 4.119595
H 1.793513 -1.403088 3.082063
C -1.504762 -3.384672 2.744234
H -1.201855 -3.286487 0.631271
C -1.019970 -3.057685 4.006485
H 0.566467 -2.087691 5.099020
H -2.428618 -3.946409 2.642386
H -1.564831 -3.357215 4.896528
C -2.650902 0.173009 2.620543
C -3.774851 -0.658609 2.629670
C -2.054839 0.510167 3.844697
C -4.300559 -1.128554 3.833871
H -4.247662 -0.946836 1.695619
C -2.588252 0.055972 5.047253
H -1.167536 1.139637 3.858079
C -3.714872 -0.767468 5.044992
H -5.172253 -1.776880 3.821548
H -2.116545 0.334242 5.985336
H -4.127250 -1.130956 5.981765
C -2.322757 2.606257 1.139356
C -3.230706 3.181192 2.034870
C -1.694599 3.427854 0.193005
C -3.506145 4.548204 1.982680
H -3.730914 2.565026 2.776635
C -1.979574 4.789362 0.131204
H -0.981225 2.996496 -0.506552
C -2.884092 5.354896 1.030620
H -4.212668 4.980881 2.685423
H -1.488906 5.408283 -0.614681
H -3.100658 6.418524 0.991325
Pd 1.990115 0.542615 0.050335
C 2.650204 2.565065 0.400754
H 2.437098 3.339618 -0.330691
H 3.705590 2.404781 0.622828
C 1.668949 2.255562 1.370945
H 0.683341 2.706423 1.276967
C 1.820114 1.144684 2.207530
H 2.809780 0.833367 2.540819
H 0.983761 0.829178 2.823058
N 6.217631 -1.794041 -2.338908
C 6.231377 -0.575245 -1.771176
C 5.037603 -2.198133 -2.824821
C 5.117332 0.238346 -1.655540
H 7.192709 -0.237355 -1.388865
C 3.867103 -1.450226 -2.765561
H 5.024607 -3.180626 -3.293539
C 3.865167 -0.189565 -2.144461
H 5.209339 1.208863 -1.174395
H 2.960625 -1.858984 -3.197052
C 1.581998 0.419104 -3.053458
H 1.109673 -0.559701 -2.921146
H 2.051019 0.391251 -4.052790
C 0.491586 1.468035 -3.093616
C -0.844990 1.078208 -3.200385
C 0.772186 2.840950 -3.088300
C -1.876505 2.011022 -3.292420
H -1.076724 0.019081 -3.219176
C -0.251368 3.782268 -3.190552
H 1.801006 3.183603 -3.016409
C -1.581777 3.372424 -3.292201
H -2.906404 1.669085 -3.355732
H -0.008462 4.841613 -3.188338
H -2.378823 4.107343 -3.358010
C 2.664361 0.659458 -1.999348
H 3.000777 1.696723 -2.073832
SCF: -3065.406457
ZPE: 0.894145
H: -3064.458488
G: -3064.584944
SP SCF: -3064.877240

Int-G_conf1
C 0.752251 -5.025120 -0.549864
C 2.062967 -4.645308 -0.832866
C 2.499255 -3.346259 -0.574699
C 1.558664 -2.456134 -0.063354
C 0.230346 -2.784545 0.195428
C -0.156606 -4.107646 -0.029338
H 0.436347 -6.047487 -0.732849
H 2.749365 -5.380109 -1.239808
H -1.165701 -4.432179 0.194657
O 1.918057 -1.173528 0.252214
C 2.871330 -0.591395 -0.533973
C 2.767060 0.789345 -0.696732
C 3.885175 -1.371486 -1.091631
C 3.766999 1.414833 -1.449710
C 4.832333 -0.714710 -1.875084
C 4.778307 0.667182 -2.045273
H 3.760180 2.489149 -1.586245
H 5.635471 -1.273961 -2.342855
H 5.536406 1.168457 -2.638772
C 3.936089 -2.856965 -0.735548
C 4.704755 -3.671011 -1.780295
H 5.740458 -3.328484 -1.851142
H 4.745143 -4.725114 -1.493450
H 4.243509 -3.596165 -2.770361
C 4.646009 -2.996174 0.633683
H 4.678536 -4.048981 0.933856
H 5.671933 -2.618767 0.565547
H 4.123731 -2.433699 1.414543
P 1.334264 1.669664 0.073858
P -0.830426 -1.408279 0.799442
C 1.656778 3.371409 -0.572195
C 2.049624 4.445150 0.231029
C 1.511456 3.587408 -1.950419
C 2.270670 5.706211 -0.325494
H 2.192198 4.313649 1.297801
C 1.739367 4.840686 -2.508326
H 1.237670 2.761733 -2.600711
C 2.113069 5.909729 -1.693442
H 2.571152 6.527810 0.318061
H 1.621438 4.981728 -3.578537
H 2.284053 6.891744 -2.124314
C 1.838303 1.830788 1.833886
C 1.006500 2.570010 2.684383
C 3.018524 1.287347 2.347078
C 1.369822 2.801101 4.006694
H 0.069934 2.974625 2.312155
C 3.364312 1.488185 3.683527
H 3.682728 0.711194 1.710504
C 2.549179 2.255875 4.512182
H 0.720170 3.390085 4.647272
H 4.279670 1.050771 4.071153
H 2.826588 2.421172 5.549112
C -0.280124 -1.217918 2.539241
C 0.481431 -2.185422 3.202581
C -0.722150 -0.095408 3.244080
C 0.793870 -2.030770 4.551025
H 0.830573 -3.066764 2.672729
C -0.431762 0.045502 4.599143
H -1.303575 0.668525 2.732282
C 0.330565 -0.918828 5.254100
H 1.393250 -2.784055 5.053979
H -0.787441 0.918677 5.137073
H 0.568773 -0.801814 6.307287
C -2.455028 -2.255619 1.036035
C -3.136952 -2.217316 2.257942
C -3.084880 -2.873391 -0.055342
C -4.417073 -2.757698 2.378633
H -2.682648 -1.757851 3.128654
C -4.362005 -3.413335 0.064241
H -2.574633 -2.944232 -1.010525
C -5.038192 -3.349271 1.281819
H -4.927747 -2.709967 3.335878
H -4.828528 -3.880674 -0.797839
H -6.038476 -3.761488 1.374640
Pd -0.931395 0.774931 -0.421846
C -2.890090 3.800767 0.687581
H -2.923640 4.406988 1.589795
H -3.840877 3.454188 0.287606
C -1.721489 3.504215 0.091673
H -0.813317 3.908425 0.542165
C -1.535156 2.671188 -1.113457
H -2.456558 2.607808 -1.688186
H -0.750427 3.067672 -1.757359
N -6.379615 2.002015 -0.263990
C -5.680479 1.186265 0.544398
C -5.872467 2.191739 -1.487040
C -4.494780 0.569554 0.183354
H -6.094483 1.026304 1.537823
C -4.694728 1.607063 -1.944723
H -6.438360 2.849495 -2.143992
C -3.950045 0.769598 -1.098848
H -3.980272 -0.059942 0.901946
H -4.367719 1.815749 -2.958287
C -2.353319 0.121981 -2.985822
H -2.200252 1.142209 -3.355868
H -3.207135 -0.291616 -3.545630
C -1.122618 -0.704438 -3.282142
C 0.129486 -0.100701 -3.440594
C -1.193424 -2.100362 -3.332097
C 1.281694 -0.864938 -3.613851
H 0.197617 0.983192 -3.415525
C -0.045028 -2.872704 -3.505505
H -2.160408 -2.587798 -3.227764
C 1.198252 -2.256719 -3.640446
H 2.245922 -0.373590 -3.716967
H -0.120620 -3.956163 -3.529159
H 2.095903 -2.856198 -3.761276
C -2.673373 0.120385 -1.484012
H -2.749963 -0.915765 -1.167434
SCF: -3065.402672
ZPE: 0.895326
H: -3064.453590
G: -3064.580156
SP SCF: -3064.874416

Int-G_conf2
C -4.857901 0.979295 -1.836815
C -4.594701 -0.198621 -2.530268
C -3.517008 -1.017811 -2.182124
C -2.716032 -0.597513 -1.120973
C -2.944197 0.584372 -0.408039
C -4.037964 1.366779 -0.780614
H -5.703515 1.597346 -2.122337
H -5.248204 -0.483243 -3.347502
H -4.242537 2.292174 -0.251349
O -1.646233 -1.349690 -0.701252
C -1.054663 -2.212347 -1.583448
C 0.255446 -2.611283 -1.278250
C -1.751713 -2.681093 -2.699969
C 0.902135 -3.455651 -2.185768
C -1.062244 -3.514282 -3.583221
C 0.254853 -3.888804 -3.338473
H 1.922591 -3.771956 -2.000301
H -1.559202 -3.893803 -4.468971
H 0.773914 -4.536051 -4.038186
C -3.242698 -2.371896 -2.830004
C -3.707271 -2.404816 -4.291084
H -3.539653 -3.390840 -4.731181
H -4.781826 -2.218154 -4.359239
H -3.183663 -1.657608 -4.896112
C -4.016737 -3.449384 -2.031539
H -5.093142 -3.252593 -2.079698
H -3.822041 -4.442857 -2.449697
H -3.716997 -3.455011 -0.978175
P 1.131173 -1.924915 0.189638
P -1.734764 1.031166 0.902034
C 2.682325 -2.907593 0.236337
C 2.682166 -4.271986 0.555984
C 3.894999 -2.273410 -0.046673
C 3.875098 -4.987809 0.580604
H 1.747601 -4.776885 0.785765
C 5.091403 -2.990946 -0.023189
H 3.903555 -1.209347 -0.272024
C 5.082230 -4.348117 0.289307
H 3.864608 -6.044780 0.829590
H 6.027360 -2.484533 -0.239748
H 6.012615 -4.907862 0.313116
C 0.258590 -2.579320 1.667366
C 0.928012 -2.462726 2.894349
C -1.009772 -3.163305 1.643380
C 0.334246 -2.909131 4.070543
H 1.926035 -2.035211 2.933294
C -1.606385 -3.604238 2.824183
H -1.545932 -3.286303 0.710113
C -0.939651 -3.475446 4.039239
H 0.866437 -2.809132 5.011822
H -2.597018 -4.047577 2.788636
H -1.409633 -3.813303 4.958046
C -2.472302 0.242908 2.391986
C -3.704959 -0.418202 2.404560
C -1.728876 0.305593 3.579059
C -4.189288 -0.992919 3.579894
H -4.297424 -0.485071 1.496669
C -2.218776 -0.254866 4.755408
H -0.761238 0.801491 3.586414
C -3.452132 -0.906559 4.758932
H -5.147315 -1.505237 3.571934
H -1.629741 -0.194561 5.665931
H -3.830654 -1.353220 5.673826
C -2.127954 2.810705 1.147664
C -2.968833 3.318108 2.143203
C -1.499941 3.705005 0.269123
C -3.181815 4.692984 2.253636
H -3.463146 2.642953 2.836115
C -1.724249 5.075311 0.369102
H -0.833639 3.324195 -0.501838
C -2.564695 5.573378 1.365848
H -3.835507 5.074763 3.032804
H -1.235349 5.752920 -0.325151
H -2.734110 6.642735 1.452812
Pd 1.657164 0.384540 -0.007912
C 2.599913 2.948885 2.978965
H 2.361024 3.974739 3.249584
H 3.620270 2.613225 3.155269
C 1.678337 2.133925 2.442503
H 0.670937 2.528595 2.292638
C 1.891528 0.733443 2.028259
H 2.885030 0.369167 2.319267
H 1.121775 0.091402 2.465590
N 6.527254 2.855715 -0.023824
C 5.589422 3.675806 0.465999
C 6.087003 1.829205 -0.770298
C 4.225037 3.517231 0.248792
H 5.947767 4.512394 1.062451
C 4.750146 1.581731 -1.037657
H 6.851428 1.167675 -1.172643
C 3.757591 2.433944 -0.513202
H 3.526588 4.236015 0.663077
H 4.481463 0.717261 -1.638359
C 1.873131 1.646475 -2.061618
H 1.663368 0.522994 -1.988396
H 2.684539 1.662883 -2.799313
C 0.622367 2.273142 -2.628642
C -0.519993 1.510225 -2.872394
C 0.587954 3.645336 -2.903771
C -1.679433 2.099943 -3.373841
H -0.505276 0.445024 -2.654455
C -0.568894 4.238898 -3.403140
H 1.470383 4.251640 -2.713902
C -1.709286 3.467462 -3.636085
H -2.561986 1.490049 -3.542069
H -0.583109 5.306228 -3.605169
H -2.615162 3.931546 -4.014996
C 2.319672 2.216227 -0.726801
H 1.711954 3.052401 -0.379621
SCF: -3065.379433
ZPE: 0.892136
H: -3064.432635
G: -3064.561611
SP SCF: -3064.851424

PdL2
C 3.633557 3.071450 -0.972267
C 3.560882 1.773523 -0.478150
C 2.341133 1.216846 -0.078213
C 1.199044 2.024844 -0.184362
C 1.248055 3.344779 -0.645462
C 2.483220 3.849612 -1.052782
H 2.557282 4.865361 -1.425087
C -0.028435 4.178178 -0.586172
C -1.195118 3.250561 -0.907274
C -0.206979 4.682676 0.867912
H -1.134126 5.259769 0.953187
H 0.633873 5.326320 1.147834
H -0.253550 3.851749 1.579131
C -2.356929 3.653417 -1.569123
C -3.437663 2.787942 -1.705444
C -3.379344 1.507730 -1.161447
C -2.227832 1.060152 -0.509967
C -1.140444 1.941768 -0.424239
O -0.004513 1.478842 0.194514
Pd -0.300120 -1.807168 -0.601229
P 2.214284 -0.497708 0.606212
C 3.995364 -0.865486 0.934623
C 4.501024 -0.480035 2.183765
C 5.830568 -0.724233 2.521032
C 6.669894 -1.379858 1.619591
C 6.172647 -1.783528 0.381303
C 4.845075 -1.525025 0.039185
H 4.477594 -1.837553 -0.934215
H 6.819144 -2.297541 -0.324359
H 7.704247 -1.579986 1.883689
H 6.208024 -0.412147 3.490551
H 3.849252 0.018590 2.898131
C 1.924029 -1.489384 -0.930399
C 2.063332 -0.976363 -2.243485
C 1.990983 -1.814128 -3.347759
C 1.755281 -3.187233 -3.189028
C 1.597717 -3.716087 -1.915116
C 1.687155 -2.884056 -0.779102
H 1.722543 -3.340910 0.208698
H 1.428230 -4.779813 -1.777354
H 1.695546 -3.834054 -4.059083
H 2.113310 -1.396796 -4.343049
H 2.247188 0.084037 -2.386175
P -2.070563 -0.634003 0.189029
C -1.965864 -0.293603 1.995106
C -1.394900 -1.272379 2.815700
C -1.300974 -1.076883 4.192147
C -1.765256 0.108061 4.762140
C -2.326401 1.093406 3.950282
C -2.428505 0.892854 2.574668
H -2.867355 1.669816 1.954592
H -2.684875 2.021345 4.386561
H -1.683331 0.266559 5.833540
H -0.853176 -1.844611 4.816358
H -1.008358 -2.185648 2.369341
C -3.772512 -1.302886 -0.012748
C -4.005601 -2.158214 -1.095703
C -5.277476 -2.679912 -1.327325
C -6.327486 -2.362690 -0.466787
C -6.101232 -1.521362 0.622937
C -4.831994 -0.991836 0.847601
H -4.672099 -0.335063 1.697545
H -6.914334 -1.275953 1.299931
H -7.317476 -2.774606 -0.639669
H -5.444471 -3.340175 -2.173249
H -3.182235 -2.417386 -1.757671
H -4.237098 0.849608 -1.253049
H -4.334678 3.116351 -2.221139
H -2.430835 4.657386 -1.972485
C 0.023678 5.390199 -1.520661
H -0.893864 5.978465 -1.436881
H 0.843795 6.057216 -1.242395
H 0.153769 5.093044 -2.566309
H 4.467351 1.181058 -0.413051
H 4.589102 3.478600 -1.288003
SCF: -2390.886813
ZPE: 0.601895
H: -2390.246674
G: -2390.344815
SP SCF: -2390.529083

TS-A

```
C   -2.766677  2.687767  1.638545
C   -1.611508  2.915806  0.892898
C   -0.577997  1.976983  0.903950
C   -0.747190  0.841572  1.690847
C   -1.882709  0.582031  2.452814
C   -2.903404  1.531069  2.407174
H   -3.814547  1.376992  2.975516
C   -1.864347 -0.680651  3.311451
C   -1.161385 -1.759985  2.487338
C   -1.005478 -0.395238  4.569328
H   -1.471271  0.395942  5.166571
H    0.007135 -0.073451  4.306342
H   -0.930780 -1.298681  5.183867
C   -1.495353 -3.112305  2.474805
C   -0.723311 -4.026290  1.758294
C    0.391595 -3.605166  1.039620
C    0.743361 -2.250918  1.004888
C   -0.055635 -1.369355  1.731495
O    0.298798 -0.046587  1.701336
Pd   1.890764  0.249729 -1.370203
H    4.173412 -0.446043 -2.503169
C    3.151625 -0.404137 -2.880631
C    2.650772  0.841907 -3.390204
H    3.134905  1.785658 -3.142549
C    1.623457  0.866807 -4.340573
H    1.406402 -0.026219 -4.913155
H    1.310712  1.811321 -4.770725
O   -0.297546  0.634081 -3.578386
C   -0.516660 -0.578275 -3.253227
O    0.009026 -1.598776 -3.714696
N   -1.502770 -0.745777 -2.222508
C   -1.773545 -2.006591 -1.707080
C   -2.762817 -2.219079 -0.819608
C   -3.645116 -1.145603 -0.361097
C   -3.269422  0.165808 -0.888237
C   -2.253936  0.321775 -1.758894
H   -1.967242  1.286063 -2.155753
H   -3.808148  1.054676 -0.582073
C   -4.702812 -1.381545  0.452714
H   -4.892323 -2.411539  0.753991
C   -5.684409 -0.354126  0.948491
H   -5.240957  0.647878  0.908399
H   -5.907740 -0.539797  2.006530
C   -6.998127 -0.328661  0.183296
C   -7.008984 -0.249346 -1.215240
C   -8.210123 -0.194911 -1.918692
C   -9.427646 -0.225434 -1.235453
C   -9.429985 -0.310168  0.155433
C   -8.223203 -0.360011  0.856089
H   -8.234746 -0.426611  1.941799
H   -10.370613 -0.340151  0.698580
H   -10.364308 -0.187527 -1.784169
H   -8.196652 -0.131506 -3.003320
H   -6.066143 -0.236072 -1.756505
H   -2.912036 -3.225730 -0.441900
H   -1.125465 -2.792510 -2.071343
H    2.818047 -1.315673 -3.372566
P    0.978224  2.075321 -0.057086
C    2.214055  2.494022  1.238515
C    3.567631  2.442217  0.886286
C    4.553800  2.759961  1.815814
C    4.198841  3.116322  3.116346
C    2.853883  3.160867  3.478181
C    1.865657  2.856513  2.542878
H    0.821676  2.902901  2.838582
H    2.569537  3.433490  4.490371
H    4.968366  3.350201  3.846167
H    5.599927  2.712682  1.528769
H    3.852757  2.142535 -0.119841
C    0.766910  3.657667 -0.963125
C    0.217311  3.597314 -2.249182
C   -0.006973  4.767133 -2.973182
C    0.329282  6.004719 -2.424903
C    0.886651  6.069409 -1.147885
C    1.101751  4.901623 -0.417223
H    1.530488  4.965476  0.578774
H    1.153622  7.030188 -0.717401
H    0.161473  6.915958 -2.991663
H   -0.437711  4.709302 -3.968735
H   -0.030859  2.630891 -2.683124
P    2.157550 -1.590532  0.027678
C    3.457316 -1.235586  1.274371
C    4.735617 -0.913412  0.797950
C    5.763858 -0.610241  1.683309
C    5.520677 -0.595448  3.057539
C    4.248843 -0.897126  3.536660
C    3.221927 -1.224790  2.650084
H    2.241909 -1.473663  3.044729
H    4.049521 -0.882546  4.604087
H    6.318908 -0.343976  3.749601
H    6.751672 -0.371610  1.300166
H    4.929778 -0.905010 -0.271705
C    2.747282 -3.123282 -0.791134
C    2.129790 -3.491768 -1.992889
C    2.483640 -4.680020 -2.627894
C    3.464435 -5.504697 -2.076148
C    4.084443 -5.140167 -0.881925
C    3.723217 -3.957313 -0.237047
H    4.202380 -3.694246  0.701196
H    4.846813 -5.778778 -0.445351
H    3.745213 -6.427156 -2.576076
H    1.995831 -4.956496 -3.558118
H    1.371260 -2.849621 -2.438143
H    0.976111 -4.335540  0.491374
H   -0.992362 -5.077946  1.760982
H   -2.358422 -3.465613  3.029040
C   -3.261684 -1.108264  3.759231
H   -3.740494 -0.311307  4.335355
H   -3.894737 -1.355530  2.903148
H   -3.201793 -1.981374  4.415432
H   -1.524274  3.816224  0.292887
H   -3.572099  3.415578  1.616928
```

SCF: -3253.918690
ZPE: 0.907983
H: -3252.954488
G: -3253.086177
SP SCF: -3253.414501

TS-B_conf1
C 6.076806 3.022830 -1.090748
C 4.813452 3.263974 -0.559085
C 3.890760 2.218930 -0.441317
C 4.271079 0.967122 -0.920721
C 5.543351 0.677040 -1.413962
C 6.446791 1.736457 -1.490109
H 7.449844 1.571342 -1.869021
C 5.817924 -0.765879 -1.846656
C 5.044191 -1.680898 -0.890365
C 5.256156 -0.967242 -3.275049
H 5.771048 -0.302182 -3.976267
H 4.184363 -0.752107 -3.321935
H 5.412042 -2.002595 -3.595533
C 5.463506 -2.935653 -0.455593
C 4.642884 -3.723001 0.357416
C 3.397435 -3.259576 0.763536
C 2.952395 -1.990522 0.372937
C 3.786347 -1.248562 -0.459242
O 3.333786 -0.025356 -0.866166
Pd 0.539066 0.837925 0.155451
P 2.309288 2.362313 0.479350
C 1.919537 4.141888 0.259474
C 1.450190 4.547732 -0.996814
C 1.145334 5.883338 -1.239210
C 1.293090 6.828988 -0.222663
C 1.754840 6.431603 1.029746
C 2.072237 5.093690 1.270764
H 2.442077 4.802144 2.248973
H 1.875215 7.162299 1.824135
H 1.048300 7.870572 -0.408222
H 0.786508 6.185623 -2.218576
H 1.326277 3.814955 -1.790378
C 2.836873 2.235905 2.233154
C 4.169789 2.133076 2.637659
C 4.486831 2.040453 3.994023
C 3.478256 2.059013 4.953364
C 2.143718 2.150894 4.555353
C 1.825009 2.224804 3.204303
H 0.782384 2.277922 2.898839
H 1.350524 2.147571 5.296706
H 3.727322 1.987617 6.007966
H 5.526496 1.956096 4.296348
H 4.969302 2.124845 1.903452
P 1.339831 -1.296200 0.925210
C 0.208387 -2.671910 0.471747
C -0.350141 -2.636522 -0.812525
C -1.183440 -3.661458 -1.250099
C -1.480132 -4.729130 -0.402794
C -0.932237 -4.768306 0.877760
C -0.087281 -3.747336 1.314149
H 0.332130 -3.792859 2.314990
H -1.164921 -5.593934 1.544009
H -2.158893 -5.508652 -0.735415
H -1.616764 -3.619526 -2.244977
H -0.138210 -1.795858 -1.469304
C 1.443795 -1.361792 2.753297
C 0.254621 -1.255257 3.488139
C 0.283795 -1.234697 4.879011
C 1.503327 -1.303185 5.554061
C 2.689412 -1.384335 4.829371
C 2.662296 -1.410563 3.435077
H 3.597031 -1.467693 2.886095
H 3.643634 -1.421788 5.346245
H 1.527132 -1.285294 6.639652
H -0.646104 -1.165330 5.435682
H -0.701242 -1.204169 2.973078
H 2.774119 -3.879079 1.401789
H 4.982621 -4.704024 0.674091
H 6.431822 -3.322118 -0.755803
C 7.315700 -1.082475 -1.853901
H 7.489313 -2.109680 -2.185734
H 7.841941 -0.433395 -2.559136
H 7.762459 -0.955638 -0.862463
H 4.554042 4.258795 -0.209008
H 6.789993 3.836802 -1.174418
C -1.845564 0.205216 0.660595
C -1.512666 0.845821 -0.546352
H -1.625268 0.294008 -1.476987
C -0.959778 2.156474 -0.548420
H -1.182449 2.837764 0.272488
H -0.772332 2.641936 -1.502885
H -1.929582 -0.872981 0.703899
H -1.775809 0.741539 1.602129
C -4.405926 -0.762437 0.159910
C -4.399759 -2.083777 0.740345
C -4.484558 -3.200012 -0.028207
N -4.581578 -3.134490 -1.390280
C -4.605250 -1.909421 -1.987392
C -4.536935 -0.750294 -1.276246
H -4.573153 0.185827 -1.820878
H -4.687659 -1.937443 -3.066585
H -4.479390 -4.203979 0.375455
H -4.320304 -2.196180 1.817285
C -4.182310 0.366460 0.932415
C -4.391328 1.782507 0.455156
C -5.843724 2.210884 0.526516
C -6.411799 2.591127 1.748762
C -7.756054 2.947752 1.834158
C -8.559616 2.930124 0.692852
C -8.004719 2.557482 -0.530527
C -6.657486 2.202913 -0.611059
H -6.233273 1.916669 -1.570237
H -8.619837 2.543995 -1.426020
H -9.607766 3.207523 0.757088
H -8.177100 3.242179 2.791599
H -5.791942 2.607258 2.642707
H -4.026865 1.902740 -0.571272
H -3.788222 2.456786 1.075267
H -4.175583 0.223492 2.011962
C -4.642516 -4.396605 -2.220447
O -4.650724 -5.435800 -1.547269
O -4.673333 -4.189740 -3.440417
SCF: -3253.922782
ZPE: 0.907012
H: -3252.958930
G: -3253.093084
SP SCF: -3253.414485

TS-C
C 5.464387 2.568666 -1.444402
C 4.176253 2.784152 -0.969358
C 3.240898 1.741919 -0.969836
C 3.643524 0.518105 -1.495954
C 4.928411 0.263835 -1.984029
C 5.839818 1.315796 -1.938699
H 6.853201 1.175192 -2.299757
C 5.196780 -1.126508 -2.570464
C 4.423671 -2.133112 -1.713399
C 4.628202 -1.166539 -4.009890
H 5.143028 -0.430377 -4.636046
H 3.557186 -0.943319 -4.027916
H 4.778133 -2.160914 -4.443077
C 4.833748 -3.430498 -1.411514
C 4.025711 -4.276669 -0.647655
C 2.817425 -3.823152 -0.128771
C 2.389788 -2.514436 -0.382128
C 3.185679 -1.728827 -1.210986
O 2.716133 -0.483507 -1.519972
Pd -0.104710 0.235386 -0.375681
P 1.546942 1.936118 -0.285424
C 1.090379 3.559071 -1.016715
C 0.682108 3.555821 -2.356766
C 0.363391 4.746630 -3.001943
C 0.438106 5.956738 -2.310329
C 0.842326 5.967362 -0.977440
C 1.173253 4.774479 -0.332684
H 1.499334 4.803480 0.702249
H 0.906510 6.905386 -0.433941
H 0.181558 6.886149 -2.809960
H 0.049513 4.729292 -4.041297
H 0.612734 2.615585 -2.898806
C 1.816584 2.281220 1.493867
C 3.021783 2.001841 2.142365
C 3.148793 2.224693 3.513636
C 2.077668 2.730005 4.245560
C 0.864925 2.992614 3.607123
C 0.730637 2.756788 2.242927
H -0.221549 2.954117 1.756254
H 0.020064 3.373620 4.173108
H 2.180844 2.905491 5.312242
H 4.088922 1.998000 4.007500
H 3.867210 1.608325 1.586392
P 0.938169 -1.761480 0.453557
C -0.181520 -3.211595 0.560578
C -0.776729 -3.641313 -0.633173
C -1.648736 -4.724223 -0.635550
C -1.950173 -5.380775 0.559344
C -1.369815 -4.951388 1.749991
C -0.482595 -3.872650 1.752442
H -0.030846 -3.555006 2.687338
H -1.601771 -5.456340 2.683162
H -2.641437 -6.218310 0.560137
H -2.104599 -5.045671 -1.566739
H -0.566159 -3.118848 -1.563944
C 1.520280 -1.502027 2.172839
C 0.637194 -0.855077 3.048847
C 0.992290 -0.636451 4.375284
C 2.246458 -1.037405 4.837058
C 3.139853 -1.654633 3.965707
C 2.778800 -1.890563 2.638555
H 3.486308 -2.376557 1.974442
H 4.122563 -1.957289 4.315208
H 2.528989 -0.856349 5.869905
H 0.296310 -0.139784 5.044286
H -0.335280 -0.525102 2.691044
H 2.216861 -4.476865 0.496999
H 4.353675 -5.290481 -0.441027
H 5.785729 -3.800473 -1.777462
C 6.692739 -1.447273 -2.619429
H 6.860184 -2.430445 -3.067622
H 7.218898 -0.723351 -3.247820
H 7.143654 -1.436364 -1.621844
H 3.899116 3.760299 -0.581798
H 6.186465 3.378942 -1.430536
C -2.283614 -0.538486 0.195764
C -2.189835 0.171490 -1.007706
H -2.335979 -0.359132 -1.945050
C -1.705874 1.503556 -1.005108
H -1.895673 2.145383 -0.145341
H -1.595926 2.025504 -1.951393
H -2.388689 -1.614587 0.198788
H -2.251548 -0.027829 1.153074
C -5.031956 -1.355306 -0.444630
C -4.864605 -2.782753 -0.514244
C -4.985937 -3.453204 -1.708307
N -5.271214 -2.879075 -2.903531
C -5.447227 -1.543342 -2.851803
C -5.352738 -0.763260 -1.714701
H -5.534587 0.304756 -1.795588
H -5.687275 -1.063792 -3.801268
H -4.844716 -4.533876 -1.727930
H -4.618907 -3.339721 0.387899
C -4.816472 -0.625441 0.720364
C -5.021029 0.858086 0.810118
C -6.451533 1.394055 0.805376
C -7.569605 0.557774 0.828881
C -8.862505 1.086423 0.832555
C -9.059448 2.465599 0.812693
C -7.949998 3.313814 0.786202
C -6.663704 2.779643 0.780407
H -5.804439 3.447802 0.754968
H -8.088873 4.391582 0.767527
H -10.064758 2.877297 0.814174
H -9.717074 0.415190 0.846959
H -7.422054 -0.518620 0.837773
H -4.492883 1.362857 -0.016553
H -4.539058 1.230267 1.724858
H -4.658542 -1.175974 1.646073
SCF: -3065.362857
ZPE: 0.890368
H: -3064.417926
G: -3064.548199
SP SCF: -3064.830501

TS-D_conf1
C 0.835245 -4.700144 -1.960731
C 2.119169 -4.201221 -2.175532
C 2.543641 -3.030528 -1.548072
C 1.622400 -2.374042 -0.729382
C 0.314783 -2.820656 -0.520159
C -0.056343 -4.021088 -1.136146
H 0.528012 -5.624445 -2.440025
H 2.794910 -4.745785 -2.826073
H -1.052819 -4.422161 -0.986611
O 1.995068 -1.236142 -0.067880
C 2.869783 -0.404777 -0.718261
C 2.717306 0.960998 -0.475697
C 3.856108 -0.941512 -1.543769
C 3.625787 1.826585 -1.089058
C 4.725055 -0.042475 -2.163427
C 4.615111 1.327623 -1.932531
H 3.559404 2.895616 -0.918555
H 5.507369 -0.406249 -2.821115
H 5.308536 2.012215 -2.411242
C 3.960491 -2.463237 -1.636042
C 4.679444 -2.911556 -2.911621
H 5.697402 -2.513932 -2.938888
H 4.770626 -4.000659 -2.941790
H 4.151320 -2.580507 -3.811857
C 4.757489 -2.965679 -0.407039
H 4.823981 -4.058817 -0.423884
H 5.771708 -2.552587 -0.423073
H 4.279702 -2.665114 0.530976
P 1.321287 1.465714 0.609338
P -0.840286 -1.760272 0.446890
C 1.503001 3.296115 0.640526
C 1.826751 4.020173 1.792985
C 1.204057 4.000595 -0.534492
C 1.854766 5.415030 1.767927
H 2.059994 3.501659 2.717879
C 1.244221 5.391585 -0.563428
H 0.954332 3.451828 -1.438823
C 1.565362 6.104659 0.592054
H 2.107155 5.961665 2.672000
H 1.017548 5.917810 -1.486394
H 1.588527 7.190320 0.575560
C 1.943918 0.990813 2.272148
C 1.015193 0.892253 3.312398
C 3.296879 0.761778 2.544906
C 1.430368 0.585357 4.606444
H -0.040252 1.050751 3.106142
C 3.711026 0.438177 3.835401
H 4.034512 0.835294 1.750806
C 2.779064 0.353531 4.869788
H 0.697484 0.511255 5.404046
H 4.763315 0.254847 4.032813
H 3.102320 0.101857 5.875727
C -0.500321 -2.198281 2.194365
C 0.493304 -3.097454 2.586708
C -1.270969 -1.563377 3.178124
C 0.708144 -3.362417 3.940105
H 1.106457 -3.596729 1.841914
C -1.080130 -1.853440 4.524550
H -2.026264 -0.836583 2.886312
C -0.083655 -2.751440 4.909552
H 1.492639 -4.053937 4.233161
H -1.694246 -1.363070 5.274312
H 0.080589 -2.965113 5.961662
C -2.451002 -2.593085 0.110680
C -3.245612 -3.180715 1.100715
C -2.966628 -2.506175 -1.192562
C -4.529833 -3.638365 0.803796
H -2.874460 -3.280807 2.115189
C -4.243498 -2.969924 -1.491705
H -2.363325 -2.080425 -1.989596
C -5.037630 -3.525971 -0.488518
H -5.133235 -4.084122 1.589311
H -4.621392 -2.887453 -2.506601
H -6.041311 -3.873448 -0.714380
Pd -0.855868 0.630874 0.038872
C -3.092332 2.819434 1.969752
H -3.015347 2.939022 3.047174
H -4.092284 2.746062 1.549377
C -1.991294 2.783095 1.198315
H -1.022535 2.891485 1.682817
C -1.992925 2.633362 -0.273097
H -2.893300 3.081660 -0.692750
H -1.127405 3.075073 -0.763881
N -6.674392 0.864858 0.155154
C -5.693230 0.172406 0.770915
C -6.400912 1.607223 -0.930898
C -4.401814 0.216760 0.309682
H -6.001974 -0.397712 1.637674
C -5.124859 1.675150 -1.448114
H -7.247632 2.130595 -1.357336
C -4.074647 0.961796 -0.840220
H -3.632582 -0.327492 0.842317
H -4.960559 2.280168 -2.331616
C -2.465868 1.538235 -2.746872
H -2.742164 2.596999 -2.816505
H -3.133775 0.996206 -3.432081
C -1.036879 1.369362 -3.215590
C -0.264345 2.476187 -3.573835
C -0.446905 0.100317 -3.284377
C 1.063466 2.324736 -3.978936
H -0.701394 3.470927 -3.522520
C 0.877926 -0.056897 -3.680807
H -1.025599 -0.779586 -3.018551
C 1.640221 1.058186 -4.029446
H 1.649430 3.201006 -4.242514
H 1.316074 -1.050742 -3.710781
H 2.676813 0.939061 -4.329153
C -2.695279 0.994978 -1.337790
H -2.336885 -0.040735 -1.308137
C -8.148824 0.801693 0.714324
O -8.235561 0.077381 1.700548
O -8.921510 1.488878 0.054146
SCF: -3253.899206
ZPE: 0.907400
H: -3252.934932
G: -3253.068389
SP SCF: -3253.402156

TS-E

C	0.839289	-4.764539	-1.814133
C	2.125146	-4.281388	-2.051608
C	2.557082	-3.089646	-1.470824
C	1.641552	-2.397942	-0.674226
C	0.328023	-2.821339	-0.454574
C	-0.048646	-4.044756	-1.020894
H	0.526112	-5.706210	-2.254264
H	2.796226	-4.854114	-2.682532
H	-1.048742	-4.431580	-0.859057
O	2.025900	-1.244647	-0.048769
C	2.907407	-0.441598	-0.724469
C	2.771030	0.930495	-0.512349
C	3.883935	-1.009203	-1.540163
C	3.682866	1.772458	-1.152053
C	4.757955	-0.133638	-2.186543
C	4.661719	1.242828	-1.989621
H	3.627073	2.845931	-1.006505
H	5.533792	-0.520952	-2.838403
H	5.358119	1.908551	-2.490294
C	3.976587	-2.533778	-1.588770
C	4.679305	-3.023864	-2.858304
H	5.697668	-2.629538	-2.910661
H	4.767801	-4.113632	-2.856007
H	4.140573	-2.719147	-3.761569
C	4.783147	-3.004561	-0.353582
H	4.842523	-4.098164	-0.338108
H	5.799802	-2.598886	-0.392146
H	4.316709	-2.672697	0.579599
P	1.390355	1.463740	0.578688
P	-0.835423	-1.706154	0.446174
C	1.627507	3.288993	0.614018
C	2.026071	3.997188	1.752909
C	1.302969	4.008091	-0.544913
C	2.101702	5.390390	1.730052
H	2.279636	3.467522	2.666063
C	1.390497	5.396992	-0.572628
H	0.993685	3.470850	-1.437268
C	1.786473	6.094183	0.569275
H	2.410940	5.924523	2.623924
H	1.142077	5.934249	-1.483581
H	1.846626	7.178458	0.554367
C	2.021763	0.970878	2.234814
C	1.099675	0.871437	3.280541
C	3.374221	0.726259	2.496843
C	1.520465	0.547054	4.568600
H	0.044672	1.042501	3.083213
C	3.794201	0.385541	3.781052
H	4.107616	0.801078	1.698892
C	2.868392	0.298922	4.820861
H	0.792034	0.472728	5.370276
H	4.846117	0.190495	3.969337
H	3.195845	0.033682	5.821997
C	-0.566419	-2.111294	2.215719
C	0.377365	-3.038446	2.662017
C	-1.345912	-1.426232	3.158062
C	0.533454	-3.283173	4.027468
H	0.996845	-3.576175	1.949991
C	-1.213885	-1.694390	4.516174
H	-2.059055	-0.675123	2.823651
C	-0.268091	-2.622259	4.955253
H	1.279699	-3.997616	4.362597
H	-1.833948	-1.163947	5.233164
H	-0.149766	-2.819856	6.016705
C	-2.438354	-2.538820	0.075482
C	-3.265653	-3.110916	1.046779
C	-2.902076	-2.484274	-1.248227
C	-4.531943	-3.590618	0.708924
H	-2.933850	-3.182802	2.077266
C	-4.160808	-2.968060	-1.587663
H	-2.268893	-2.070245	-2.028088
C	-4.987545	-3.513736	-0.605022
H	-5.161663	-4.026055	1.479528
H	-4.499784	-2.909957	-2.617807
H	-5.977025	-3.878602	-0.863842
Pd	-0.822859	0.677972	0.043315
C	-2.832628	2.570507	2.117275
H	-2.668011	2.657423	3.188189
H	-3.840572	2.336463	1.784285
C	-1.819598	2.752045	1.248305
H	-0.842500	3.011953	1.652053
C	-1.924681	2.663890	-0.227683
H	-2.867659	3.093312	-0.563453
H	-1.107978	3.161385	-0.749081
N	-6.750069	1.081522	-0.047498
C	-5.818336	0.294352	0.514307
C	-6.336884	1.870673	-1.044492
C	-4.489414	0.270802	0.122234
H	-6.155783	-0.347513	1.325023
C	-5.026925	1.909425	-1.516008
H	-7.092760	2.509032	-1.497119
C	-4.053837	1.087294	-0.931674
H	-3.788214	-0.375341	0.637406
H	-4.784578	2.577410	-2.335932
C	-2.348492	1.678094	-2.745092
H	-2.560874	2.753639	-2.763568
H	-3.041089	1.215586	-3.463927
C	-0.930683	1.451426	-3.222594
C	-0.118308	2.526485	-3.590792
C	-0.394127	0.159913	-3.309541
C	1.193876	2.323163	-4.023170
H	-0.513232	3.538211	-3.528648
C	0.914804	-0.049961	-3.733883
H	-1.004514	-0.696371	-3.039242
C	1.716051	1.034101	-4.092979
H	1.810207	3.176329	-4.293413
H	1.310409	-1.061002	-3.779387
H	2.740065	0.873377	-4.415777
C	-2.633915	1.077206	-1.368260
H	-2.333198	0.026026	-1.397459

SCF: -3065.362713
ZPE: 0.892495
H: -3064.416161
G: -3064.543924
SP SCF: -3064.839897

TS-F_conf1
C -4.857363 0.584946 -1.068520
C -4.379109 -0.411853 -1.911150
C -3.098812 -0.951795 -1.757353
C -2.321822 -0.465262 -0.704342
C -2.758352 0.564950 0.143217
C -4.040351 1.077936 -0.055682
H -5.855726 0.986768 -1.210278
H -5.015574 -0.775802 -2.712474
H -4.401804 1.877226 0.584314
O -1.071132 -0.950681 -0.446556
C -0.487940 -1.862673 -1.277734
C 0.810385 -2.255904 -0.905427
C -1.137467 -2.373649 -2.398495
C 1.484744 -3.152018 -1.732833
C -0.423047 -3.274779 -3.197292
C 0.874982 -3.653350 -2.882006
H 2.493793 -3.462542 -1.483298
H -0.896791 -3.695177 -4.079439
H 1.410202 -4.349317 -3.520336
C -2.581352 -2.008257 -2.731465
C -2.655586 -1.451398 -4.168864
H -2.297956 -2.189776 -4.892325
H -3.686557 -1.202916 -4.436950
H -2.046367 -0.547939 -4.269452
C -3.453434 -3.277284 -2.622447
H -4.497639 -3.051394 -2.860925
H -3.107371 -4.044508 -3.322529
H -3.412424 -3.690519 -1.609441
P 1.592986 -1.473596 0.563402
P -1.598331 1.242479 1.410170
C 3.029164 -2.574866 0.905617
C 2.878042 -3.945383 1.158930
C 4.303999 -2.010158 0.973524
C 3.983920 -4.733367 1.460693
H 1.891794 -4.400331 1.121111
C 5.414475 -2.797779 1.280123
H 4.427793 -0.947081 0.782432
C 5.256456 -4.159409 1.521868
H 3.854333 -5.794466 1.653218
H 6.400512 -2.344846 1.321106
H 6.119453 -4.774574 1.759759
C 0.490224 -1.988202 1.950403
C 0.710976 -1.393932 3.195898
C -0.483182 -2.985632 1.838810
C -0.010660 -1.803268 4.314927
H 1.449839 -0.601664 3.287318
C -1.218580 -3.382816 2.953729
H -0.673038 -3.463308 0.881824
C -0.978895 -2.798158 4.196475
H 0.173101 -1.332463 5.275933
H -1.978775 -4.151946 2.849909
H -1.552443 -3.107313 5.065216
C -2.350367 0.660450 2.985871
C -3.383608 -0.277296 3.062490
C -1.802906 1.155720 4.179270
C -3.873003 -0.695103 4.301468
H -3.814036 -0.690304 2.155206
C -2.301907 0.753628 5.413943
H -0.983097 1.870042 4.141809
C -3.341172 -0.176308 5.479040
H -4.673832 -1.428205 4.341123
H -1.872257 1.157686 6.326202
H -3.726712 -0.498298 6.442064
C -2.098297 3.020181 1.382122
C -3.019623 3.611321 2.254767
C -1.482316 3.822440 0.411628
C -3.315965 4.970949 2.160366
H -3.515038 3.012201 3.013158
C -1.785398 5.178312 0.309725
H -0.766152 3.381609 -0.278858
C -2.700404 5.758222 1.187871
H -4.033063 5.413110 2.846287
H -1.301408 5.780450 -0.453785
H -2.931362 6.817118 1.115705
Pd 2.075734 0.763031 0.394752
C 2.718203 2.910854 -0.135410
H 2.254255 3.660420 -0.768255
H 3.793832 3.048596 -0.052373
C 2.024926 2.764240 1.147673
H 1.000970 3.128283 1.202739
C 2.532955 2.086441 2.240856
H 3.594397 1.860614 2.330920
H 1.943546 2.020305 3.150916
N 6.250908 -1.135027 -1.773442
C 6.333004 0.085591 -1.227102
C 5.059214 -1.467308 -2.288567
C 5.279603 0.988364 -1.176661
H 7.300411 0.362621 -0.813195
C 3.945718 -0.639308 -2.282195
H 4.989140 -2.456642 -2.736007
C 4.022829 0.640281 -1.704153
H 5.447813 1.964705 -0.733989
H 3.025738 -1.006264 -2.721231
C 1.734267 1.227515 -2.646667
H 1.337081 0.251569 -2.355689
H 2.145250 1.104379 -3.663226
C 0.570312 2.185269 -2.748374
C -0.728833 1.673861 -2.715389
C 0.731538 3.559262 -2.964549
C -1.841547 2.494665 -2.884056
H -0.864308 0.608363 -2.566976
C -0.376586 4.389437 -3.130208
H 1.729387 3.987698 -3.019401
C -1.668065 3.861180 -3.090785
H -2.838627 2.064467 -2.840519
H -0.230257 5.453541 -3.294607
H -2.529161 4.511848 -3.211659
C 2.901614 1.632471 -1.741484
H 3.333365 2.545798 -2.151468
SCF: -3065.355770
ZPE: 0.893166
H: -3064.408772
G: -3064.535807
SP SCF: -3064.830262

References

[1] Still, W. C.; Kahn, M.; Mitra, A. *J. Org. Chem.* **1978**, *43*, 2923–2925.
[2] Hoye, T. R.; Zhao, H. *J. Org. Chem.* **2002**, *67*, 4014–4016.
[3] Duez, S.; Steib, A. K.; Manolikakes, S. M.; Knochel, P. *Angew. Chem. Int. Ed*. **2011**, *50*, 7686–7690.
[4] Chen, Q.; Mollat, X.; Knochel, P. *J. Am. Chem. Soc*. **2013**, 135, 2–5.
[5] Schmaunz, C. E.; Pabel, J.; Wanner, K. T. *Synthesis.* **2010**, 2147-2160.
[6] Howell, J. M.; Feng, K.; Clark, J. R.; Trzepkowski, L. J.; White, M. C. *J. Am. Chem. Soc.* **2015**, *137*, 14590–14593.
[7] Gao, K.; Yamamoto, K.; Nogi, K.; Yorimitsu, H. *Synlett.* **2017**, *28*, 2956-2960.
[8] Chia, W.; Shiao, M. *Tet. Lett*. **1991**, *32*, 2033-2034.
[9] Lebel, H.; Davi, M.; Díez-González, S.; Nolan, S. P. *J. Org. Chem.* **2007**, *72*, 144–149.
[10] Jiang, J.; Wang, W.; Sane, D. C.; Wang, B. *Bioorg. Chem*. **2001**, *29*, 357–379.
[11] Kitbunnadaj, R.; Zuiderveld, O. P.; Christophe, B.; Hulscher, S.; Menge, W. M. P. B.; Gelens, E.; Snip, E.; Bakker, R. A.; Celanire, S.; Gillard, M.; Talaga, P.; Timmerman, H.; Leurs, R. *J. Med. Chem.* **2004**, *47*, 2414–2417.
[12] Massaro, A.; Mordini, A.; Mingardi, A.; Klein, J.; Andreotti, D. *Eur. J. Org. Chem.* **2011**, 271–279.
[13] Brundish, D.; Bull, A.; Donovan, V.; Fullerton, J. D.; Garman, S. M.; Hayler, J. F.; Janus, D.; Kane, P. D.; McDonnell, M.; Smith, G. P.; Wakeford, R.; Walker, C. V.; Howarth, G.; Hoyle, W.; Allen, M. C.; Ambler, J.; Butler, K.; Talbot, M. D. *J. Med. Chem.* **1999**, *42*, 4584–4603.
[14] Evans, O. R.; Lin, W. *Chem. Mater.* **2001**, *13*, 2705–2712.
[15] Hunter, C. A.; Misuraca, M. C.; Turega, S. M. *J. Am. Chem. Soc.* **2011**, *133*, 582–594.
[16] Benington, F.; Morin, R. D.; Khaled, M. A. *Synthesis* **1984**, 619–620.
[17] Konishi, H.; Tanaka, H.; Manabe, K. *Org. Lett.* **2017**, *19*, 1578–1581.
[18] Kuriyama, M.; Matsuo, S.; Shinozawa, M.; Onomura, O. *Org. Lett.* **2013**, *15*, 2716–2719.
[19] Bonnet, V.; Mongin, F.; Trécourt, F.; Quéguiner, G. *J. Chem. Soc. Perkin Trans.* **2000**, 4245–4249.
[20] Huestis, M. P.; Fagnou, K. *Org. Lett.* **2009**, *11*, 1357–1360.
[21] Maekawa, H.; Nishiyama, Y. *Tetrahedron* **2015**, *71*, 6694–6700.
[22] Oyama,T.; Yatabe, T.; Jin, X.; Mizuno, N.; Yamaguchi, K. *Chem. Lett*. **2019**, 48, 517–520.
[23] Rideau, E.; You, H.; Sidera, M.; Claridge, T. D. W.; Fletcher, S. P. *J. Am. Chem. Soc*. **2017**, *139*, 5614–5624.
[24] Johns, A. M.; Utsunomiya, M.; Incarvito, C. D.; Hartwig, J. F. *J. Am. Chem. Soc*. **2006**, *128*, 1828-1839.
[25] Kobayashi, Y.; Czechtizky, W.; Kishi, Y. *Org. Lett*. **2003**, 5, 93-96.
[26] Wu, H.; Yang, B.; Zhu, L.; Lu, R.; Li, G.; Lu, H. *Org. Lett*. **2016**, *18*, 5804–5807.
[27] Trost, B. M.; O'Boyle, B. M. *Org. Lett.* **2008**, *10*, 1369-1372.
[28] Huang, D.; Liu, X.; Li, L.; Cai, Y.; Liu, W.; Shi, Y. *J. Am. Chem. Soc*. **2013**, *135*, 8101–8104.
[29] Sha, S.; Zhang, J.; Carroll, P. J.; Walsh, P. J. *J. Am. Chem. Soc*. **2013**, *135*, 17602–17609.
[30] Gaussian 16, Revision A.03, M. J. Frisch, G. W. Trucks, H. B. Schlegel, G. E. Scuseria, M. A. Robb, J. R. Cheeseman, G. Scalmani, V. Barone, B. Mennucci, G. A. Petersson, H. Nakatsuji, M. Caricato, X. Li, H. P. Hratchian, A. F. Izmaylov, J. Bloino, G. Zheng, J. L. Sonnenberg, M. Hada, M. Ehara, K. Toyota, R. Fukuda, J. Hasegawa, M. Ishida, T. Nakajima, Y. Honda, O. Kitao, H. Nakai, T. Vreven, J. A. Montgomery, Jr., J. E. Peralta, F. Ogliaro, M. Bearpark, J. J. Heyd, E. Brothers, K. N. Kudin, V. N. Staroverov, R. Kobayashi, J. Normand, K. Raghavachari, A. Rendell, J. C. Burant, S. S. Iyengar, J. Tomasi, M. Cossi, N. Rega, J. M. Millam, M. Klene, J.

E. Knox, J. B. Cross, V. Bakken, C. Adamo, J. Jaramillo, R. Gomperts, R. E. Stratmann, O. Yazyev, A. J. Austin, R. Cammi, C. Pomelli, J. W. Ochterski, R. L. Martin, K. Morokuma, V. G. Zakrzewski, G. A. Voth, P. Salvador, J. J. Dannenberg, S. Dapprich, A. D. Daniels, Ö. Farkas, J. B. Foresman, J. V. Ortiz, J. Cioslowski, and D. J. Fox, Gaussian, Inc., Wallingford CT, 2016.
[31] Grimme, S. Exploration of Chemical Compound, Conformer, and Reaction Space with Meta-Dynamics Simulations Based on Tight-Binding Quantum Chemical Calculations. *Journal of Chemical Theory and Computation* **2019**, *15*, 2847–2862.
[32] Pracht, P.; Bohle, F.; Grimme, S. Automated Exploration of the Low-Energy Chemical Space with Fast Quantum Chemical Methods. *Physical Chemistry Chemical Physics* **2020**, *22*, 7169–7192.
[33] Chai, J.; Head-Gordon, M. Long-Range Corrected Hybrid Density Functionals With Damped Atom–Atom Dispersion Corrections. *Physical Chemistry Chemical Physics* **2008**, *10*, 6615–6620.
[34] Rassolov, V.; Ratner, M.; Pople, J.; Redfern, P.; Curtiss, L. 6-31G* Basis Set For Third-Row Atoms. *Journal of Computational Chemistry* 2001, *22*, 976-984.
[35] Dolg, M.; Wedig, U.; Stoll, H.; Preuss, H. Energy-Adjusted ab initio pseudopotentials for the First Row Transition Elements. *The Journal of Chemical Physics* **1987**, *86*, 866–872.
[36] Zhao, Y.; Truhlar, D. G. The M06 Suite of Density Functionals for Main Group Thermochemistry, Thermochemical Kinetics, Noncovalent Interactions, Excited States, and Transition Elements: Two New Functionals and Systematic Testing of Four M06-Class Functionals and 12 Other Functionals. *Theoretical Chemistry Accounts* **2008**, *120*, 215–241.
[37] McLean, A.; Chandler, G. Contracted Gaussian Basis Sets for Molecular Calculations. I. Second Row Atoms, Z=11–18. *The Journal of Chemical Physics* 1980, *72*, 5639-5648.
[38] Marenich, A. V.; Cramer, C. J.; Truhlar, D. G. Universal Solvation Model Based on Solute Electron Density and on a Continuum Model of the Solvent Defined by the Bulk Dielectric Constant and Atomic Surface Tensions. *The Journal of Physical Chemistry B* **2009**, *113*, 6378–6396.
[39] Grimme, S. Supramolecular Binding Thermodynamics by Dispersion-Corrected Density Functional Theory. *Chemistry - A European Journal* **2012**, *18*, 9955–9964.
